# Quantitative Proteomic Profiling Identifies SOX8 as Novel Regulator of Drug Resistance in Gestational Trophoblastic Neoplasia

**DOI:** 10.3389/fonc.2020.00557

**Published:** 2020-04-28

**Authors:** Fu Jun, Zheng Peng, Yi Zhang, Dazun Shi

**Affiliations:** ^1^Department of Oncology, Xiangya Hospital, Central South University, Changsha, China; ^2^Department of Neurosurgery, Sanbo Brain Hospital, Capital Medical University, Beijing, China; ^3^Department of Gynecology and Obstetrics, Xiangya Hospital, Central South University, Changsha, China; ^4^Gynecological Oncology Research and Engineering Center of Hunan Province, Changsha, China

**Keywords:** gestational trophoblastic neoplasia, drug resistance, quantitative proteomics, SOX8, reactive oxygen species

## Abstract

The development of drug resistance remains one of the major challenges to current chemotherapeutic regimens in gestational trophoblastic neoplasia (GTN). Further understanding on the mechanisms of drug resistance would help to develop more effective therapy to treat GTN. Herein, tandem mass tag-based (TMT) quantitative proteomic technique was used to establish drug resistance-related proteomic profiles in chemoresistant GTN cell models (JEG3/MTX, JEG3/VP16, JEG3/5-Fu). In total, we identified 5,704 protein groups, among which 4,997 proteins were quantified in JEG3 and its chemoresistant sublines. Bioinformatics analysis revealed that multiple biological processes/molecular pathways/signaling networks were involved in the regulation of drug resistance in chemoresistant JEG3 sublines. SOX8 was upregulated in all the three chemoresistant sublines, and its function was further investigated. Knockdown of SOX8 significantly reduced cell viability, impaired soft agar clonogenesis, and increased caspase-3 activities after drug treatment in JEG3 chemoresistant sublines. In addition, over-expression of SOX8 promoted cell survival, enhanced soft agar clonogenesis, and attenuated caspase-3 activities after drug treatment in GTN cells. Importantly, SOX8 might be a potential regulator of reactive oxygen species (ROS) homeostasis, as SOX8 regulated the expression of antioxidant enzymes (GPX1, HMOX1) and reduced drug-induced ROS accumulation in GTN cell models. Collectively, SOX8 might promote drug resistance through attenuating the accumulation of ROS induced by chemotherapeutic drugs in GTN cells. Targeting SOX8 might be useful to sensitize GTN cells to chemotherapy.

## Introduction

Gestational trophoblastic neoplasia (GTN, including invasive mole, placental site trophoblastic tumor, choriocarcinoma, and epithelioid trophoblastic tumor) is one of the most successfully treated cancers due to its intrinsic sensitivity to chemotherapy (1). Low-risk GTNs could be treated with single-agent methotrexate (MTX) or actinomycin D (ACT-D); while high-risk GTNs are routinely treated with multi-agent regimens such as EMA/CO (etoposide, methotrexate, actinomycin D, cyclophosphamide, vincristine) (2, 3). However, resistance to chemotherapy represents a major problem to the successful management of high-risk GTN. About 20~30% of low-risk GTN fails to achieve complete remission after treatment with single-agent MTX or ACT-D (4, 5). Nevertheless, about 10~20% of high-risk GTN patients fails to render a complete response to routinely used EMA/CO regimen, which always results in cancer recurrence and distant metastasis (6, 7). Therefore, further understanding on the mechanisms of drug resistance would help to develop more effective therapy for GTN.

The phenomenon of drug resistance in GTN is well-recognized but yet not fully understood. Some earlier studies showed that membrane ATP-binding-cassette (ABC) transporter including MRP1 and ABCG2 contributed to drug efflux in GTN cell lines (8–10). However, extended lists of mechanisms responsible for drug resistance were also elucidated in GTN, including upregulation of drug target gene (11), inhibition of cell death/apoptosis (12), activation of interferon signaling (13), involvement of cancer stem cells (14), and dysregulation of Long non-coding RNAs (15). Recently, we showed that STAT3/NFIL3 signaling axis might regulate drug resistance to 5-Fu, MTX, and VP16 in GTN cells (16). Therefore, multiple genes/pathways might be involved in regulating drug resistance in GTN, which needs to be comprehensively characterized and functionally validated in future study.

Quantitative proteomic techniques have been developed to precisely determine the global protein expression in body fluids, cells, and tissues (17). New generations of quantitative proteomic techniques such as tandem mass tag (TMT) based tandem mass spectrometry have been utilized to quantify proteins in different biological samples (18). Recently, TMT labeling and liquid chromatography-tandem mass spectrometry (LC-MS/MS) was used to search for potential proteins involved in drug resistance in cancer cells. Zhao et al. (19) identified potential signaling determinants of acquired resistance to nanoparticle Abraxane in lung cancer cells. Wang et al. (20) established functional proteomic profiles associated with resistance to cisplatin in ovarian cancer cells. Our recent study obtained the proteomic profiles associated with radioresistance in nasopharyngeal cancer cells (21). In this study, TMT-based quantitative proteomic technique was used to establish drug resistance related proteomic profiles, and to identify novel regulators of drug resistance in chemoresistant GTN cell models.

## Materials and Methods

### Reagents and Cell Lines

Primary antibodies against SLAMF1, TTN, GRIA2, UBIAD1, SOX8, and β-actin were obtained from Abcam (Cambridge, MA, USA). Five-Fu, MTX, VP16, N-Acetyl-L-cysteine (NAC), and 2,7-Dichlorodi-hydrofluorescein diacetate (DCFDA) were obtained from Sigma-Aldrich. Human GTN cell line JAR and JEG3 were provided by ATCC culture collection (Manassas, VA, USA). JEG3 chemoresistant sublines (JEG3/MTX, JEG3/5-Fu, and JEG3/VP16) were established as we described previously (16). All the GTN cell lines were grown in DMEM media supplemented with 10% fetal bovine serum. Lentiviral plasmids expressing non-targeting scramble and shSOX8 (shRNA sequences are provided in Supplementary File) were purchased from Genecopoeia Inc. (Rockville, MD, USA). Lentiviral plasmids LV105 expressing empty vector and SOX8 open reading frames (ORFs) were also provided by Genecopoeia Inc. The production of lentiviral particles was performed as described previously (22).

### TMT Labeling, LC-MS/MS Analysis, and Database Search

JEG3 and its chemoresistant sublines (JEG3/MTX, JEG3/5-Fu, and JEG3/VP16) were used for quantitative proteomic analysis with two biological replicates for each cell line. Protein extraction, trypsin digestion, and TMT labeling of the peptides were conducted as we described previously (21). The resulting peptides were analyzed by tandem mass spectrometry (MS/MS) coupled with high performance liquid chromatography (HPLC). Tandem mass spectra were searched against the reference Human Swissprot database.

### Pathway and Process Enrichment Analysis

Pathway and process enrichment analysis was conducted in *Metascape* (http://metascape.org/gp/index.html) with the following ontology sources: gene ontology (GO) biological processes, kyoto encyclopedia of genes and genomes (KEGG) pathway, canonical pathways and CORUM, and reactome gene sets (21).

### Soft Agar Assay

Soft agar assay was used to examine the *in vitro* clonogenesis of GTN cells after drug treatment (23). Briefly, the 2 mL culture medium with 0.5% agar was first plated into each well of a 6 cm culture dish. After the agar solidified, each well-received another 2 mL of 0.35% agar in culture medium containing 1 × 10^5^ cells with or without drugs. After 10~12 days, colonies were fixed by 4% paraformaldehyde, stained with 0.1% crystal violet and counted.

### Cell Viability Analysis

Cell viability was evaluated with CCK-8 assay as we described previously (16). Briefly, GTN cells were seeded in a 24-well-culture plates in triplicate (2 × 10^4^/well). Cell viability was monitored by CCK-8 viability assay at 48 h after drug treatment. IC_50_-values (the concentration of a drug that is required to suppress 50% of the cell viability) were calculated in SPSS software as described previously (16).

### Western Blotting

Western blotting was conducted as we described previously (21). Protein lysates (15 μg) was separated by Sodium dodecyl sulfate-polyacrylamide gel electrophoresis (SDS-PAGE) and transferred to polyvinylidene fluoride (PVDF) membranes. Blots were blocked and incubated with diluted antibodies, followed by incubation with horseradish peroxidase (HRP)-conjugated secondary antibody for 1 h at room temperature. The signal was visualized by enhanced chemiluminescence (ECL).

### Caspase-3 Activity Assay

Cell apoptosis was determined by Caspase-3 Colorimetric Assay (21). Briefly, GTN cells (5 × 10^5^ cells) were lysed and centrifuged, followed by enzyme reactions with chromogen. The absorbance was measured at 405 nm wavelength.

### Real-Time PCR

The mRNA levels of antioxidant enzymes (GPX1, HMOX1) were analyzed using real-time PCR (22). Briefly, total RNA was extracted from GTN cells using TRIzol reagent and further purified using the RNeasy kit (QIAGEN, USA). Total RNA (1 μg) was used to generate cDNA, which was then used for the quantitative PCR using SYBR Green PCR expression assays (Invitrogen, USA). Relative gene expression was determined based on the threshold cycles (Ct-values) of GPX1/HMOX1 and of the internal reference gene β-Actin. PCR Primers for GPX1, HMOX1, and β-Actin genes are listed (in Supplementary File).

### ROS Measurement

Intracellular ROS levels were evaluated by DCFDA fluorescence as we described previously (21). Briefly, GTN cells were incubated with 25 μM DCFDA for 30 min after drug treatment for 48 h. Florescence DCF was measured using F97Pro fluorospectrometer (Lengguang Technology, Shanghai, China).

### Statistical Analysis

Statistical analysis was conducted with SPSS 16.0 (SPSS Inc., Chicago, IL, USA). Error bars throughout the figures indicate standard deviation. The means of two groups were compared by Student's *t*-test. The means of three or more groups were compared by One-way ANOVA analysis. The difference was considered statistically significant when *P* < 0.05 in all the tests.

## Results

### Drug Resistance-Associated Proteomic Profiles in Chemoresistant JEG3 Sublines

**Figure 1:**
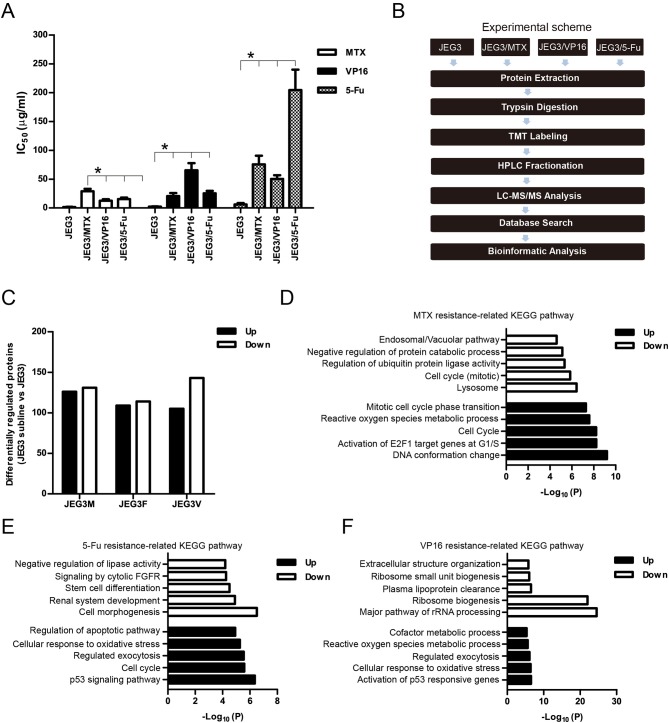
**(A)** IC_50_ concentrations of JEG3 and its chemoresistant sublines treated with MTX, 5-Fu, or VP16, respectively. *n* = 3, *P < 0.05, JEG3 sublines vs. JEG3. **(B)** Experimental scheme for the quantitative proteomic analysis on JEG3 and its chemoresistant sublines. **(C)** The number of differentially expressed proteins identified by TMT labeling and LC-MS/MS in JEG3 and its chemoresistant sublines. Criteria were set for up-regulation (JEG3 sublines vs. JEG3, fold change ≥ 1.5) and down-regulation (JEG3 sublines vs. JEG3, fold change ≤ 0.67). **(D)** Top enriched molecular pathways/biological processes of up-regulated or down-regulated proteins in JEG3/MTX. **(E)** Top enriched molecular pathways/biological processes of up-regulated or down-regulated proteins in JEG3/5-Fu. **(F)** Top enriched molecular pathways/biological processes of up-regulated or down-regulated proteins in JEG3/VP16.

In our previous study, we established three chemoresistant JEG3 sublines JEG3/MTX (JEG3M), JEG3/5-Fu (JEG3F), and JEG3/VP16 (JEG3V) (16). These chemoresistant sublines exhibited cross-resistance to chemotherapeutic drugs MTX, 5-Fu, and VP16, respectively (Figure 1A). TMT-based quantitative proteomic analysis was conducted on JEG3 and its chemoresistant sublines as illustrated in (Figure 1B). In total, we identified 5,704 protein groups, among which 4,997 proteins were quantified (Table S1). The distribution of mass error is near zero and most of them are <10 ppm, suggesting the mass accuracy of the MS data fit the requirement (Figure S1A). The detection and quantification of proteins showed good distribution of peptide length and peptide sequence coverage (Figures S1B,C).

**Figure 2:**
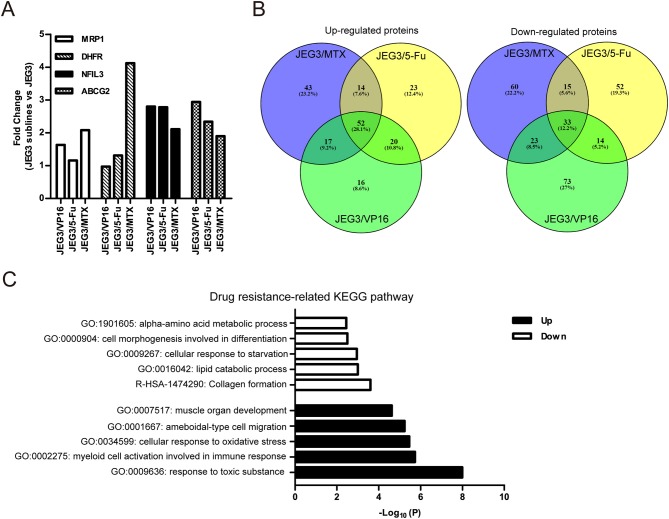
**(A)** Relative expression levels of known drug resistance-related proteins in JEG3 and its chemoresistant sublines (JEG3 sublines vs. JEG3). **(B)** Venn diagram of up- or down-regulated drug resistance-related proteins across JEG3 chemoresistant sublines. **(C)** Top five commonly enriched molecular pathways/biological processes of up-regulated (*n* = 52) or down-regulated (*n* = 33) drug resistance-related proteins in JEG3 chemoresistant sublines.

Among the 4,997 quantifiable proteins, criteria were set for up-regulation (JEG3 sublines vs. JEG3, fold change ≥ 1.5) and down-regulation (JEG3 sublines vs. JEG3, fold change ≤ 0.67). The number of proteins up- or down-regulated in JEG3/MTX, JEG3/5-Fu, and JEG3/VP16 was 126/131, 109/114, and 105/143, respectively (Figure 1C, Table S2). KEGG pathway analysis revealed differential patterns of pathway activation in three chemoresistant JEG3 sublines (Figures 1D–F). Our proteomic analysis also revealed differential patterns of expression of some known drug resistance-related proteins in these chemoresistant sublines compared to JEG3. As shown in (Figure 2A), ABC transporter MRP1/ABCC1 expression increased in both JEG3/VP16 and JEG3/MTX, while ABCG2 were up-regulated in all the three chemoresistant JEG3 sublines. Dihydrofolate reductase (DHFR), a well-known regulator of MTX resistance (24), was only up-regulated in JEG3/MTX. Consistent with our previous study (16), increased NFIL3 expression was observed in all the three chemoresistant JEG3 sublines.

Our study also identified 52 up-regulated and 33 down-regulated proteins across three chemoresistant JEG3 sublines (Figure 2B, Table S2). Pathway and process enrichment analysis for these 85 differentially expressed proteins was carried out in *Metascape* in order to identify common biological processes and signaling pathways associated with drug resistance in these sublines. Our findings showed that the GO biological processes such as response to toxic substance (ASS1, CES1, GPX1, GPX3, GSN, HMOX1, MAPK1, ABCG2, SETX, UBIAD1, SRXN1, TP53, AKR1C1, C1QTNF6), myeloid cell activation involved in immune response (ANXA3, GSN, HMOX1, CXCR2, MAPK1, S100P, SLAMF1, MVP, METTL7A, TP53, PFN2, TTN), cellular response to oxidative stress (GPX1, GPX3, HMOX1, MAPK1, TP53, SETX, SRXN1), ameboidal-type cell migration (ANXA3, ARHGDIB, DPP4, GPX1, HMOX1, PFN2, S100P, SOX8), and muscle organ development (ASS1, FHL1, GPX1, MAPK1, TTN, AKAP6, SOX8) were highly enriched in the up-regulated proteins (Figure 2C). In contrast, the down-regulated proteins were shown to enrich reactome gene set such as collagen formation (COL12A1, CTSV, COL21A1, SLC7A7, CRIM1), and GO biological processes such as lipid catabolic process (LIPA, SMPDL3A, PLCH1, ABHD6, GOT1, VLDLR), cellular response to starvation (CTSV, DSC2, GABARAPL2, GOT1, VLDLR, ISG15, N4BP1, ACSL1), cell morphogenesis involved in differentiation (COL12A1, VLDLR, EVL, ENAH, COL21A1), and alpha-amino acid metabolic process (GOT1, SLC7A7, RIDA; Figure 2C).

### Drug Resistance-Associated Signaling Networks in Chemoresistant JEG3 Sublines

**Figure 3:**
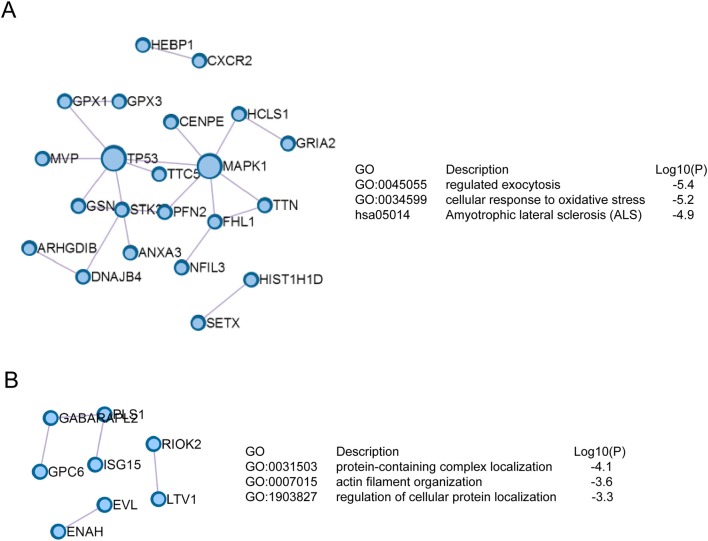
Signaling networks based on Protein-Protein interaction enrichment analysis on drug resistance-related proteins. **(A)** Signaling networks identified in up-regulated drug resistance-related proteins in JEG3 chemoresistant sublines. **(B)** Signaling networks identified in down-regulated drug resistance-related proteins in JEG3 chemoresistant sublines.

The signaling networks encoded by drug resistance-related proteins were revealed by Protein-Protein interaction enrichment analysis. As shown in (Figure 3A), proteins up-regulated in JEG3 chemoresistant sublines usually interacted with each other to constitute a signaling network. These proteins might have important functions in exocytosis, cellular response to oxidative stress, and amyotrophic lateral sclerosis. In contrast, proteins down-regulated in JEG3 sublines exhibited the enriched signaling networks involved in protein-containing complex localization, actin filament organization, and regulation of cellular protein localization (Figure 3B).

### SOX8 Might Be a Candidate Regulator of Drug Resistance in Chemoresistant JEG3 Sublines

**Figure 4:**
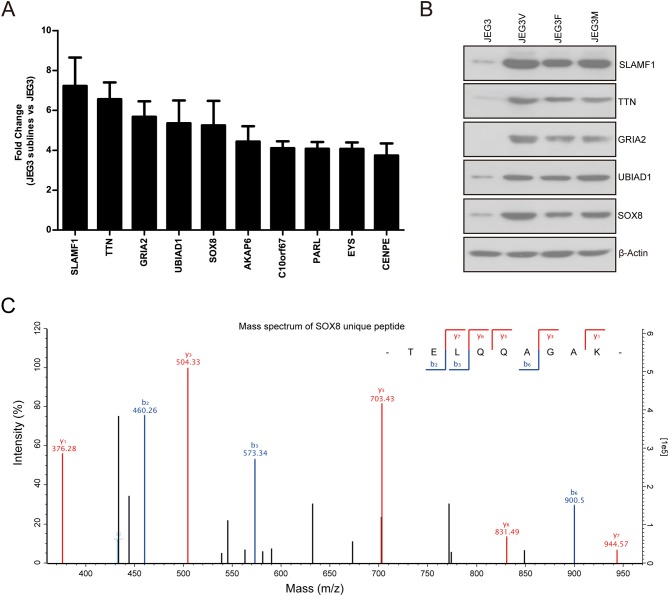
**(A)** Relative protein levels of top 10 up-regulated drug resistance-related proteins in JEG3 and its chemoresistant sublines (JEG3 sublines vs. JEG3). **(B)** Western blotting validation on protein levels of SLAMF1, TTN, GRIA2, UBIAD1, and SOX8 in JEG3 and its chemoresistant sublines. β-actin was used as loading control. **(C)** The mass spectrum of SOX8 unique peptide (TELQQAGAK) identified by TMT labeling and LC-MS/MS.

Our proteomic analysis identified several proteins highly expressed in JEG3 chemoresistant sublines compared to JEG3 (Figure 4A). Consistently, western blotting validation showed that protein levels of SLAMF1, TTN, GRIA2, UBIAD1, and SOX8 were markedly higher in three JEG3 chemoresistant sublines than those in JEG3 (Figure 4B). SLAMF1, TTN, GRIA2, UBIAD1 genes have important physiological function in normal tissues. SLAMF1 is involved in modulating the activation and differentiation of immune cells (25); TTN is a key component in the assembly and functioning of vertebrate striated muscles (26); GRIA2 is the predominant excitatory neurotransmitter receptors in the mammalian brain and are required for normal neurophysiologic processes (27); UBIAD1 presents at high concentrations in the brain, kidney, and pancreas, and is required for vitamin K metabolism (28). SOX8 exhibits low expression in normal adult human tissues, and has recently been proven to be a potential oncogene in several cancers, despite its function in the regulation of drug resistance still remains poorly understood (29). Therefore, we further investigated the function of SOX8 in drug resistance in GTN cells. The mass spectrum of SOX8 unique peptide (TELQQAGAK) was shown in Figure 4C.

### Knockdown of SOX8 Attenuated Drug Resistance Phenotype in Chemoresistant JEG3 Sublines

**Figure 5:**
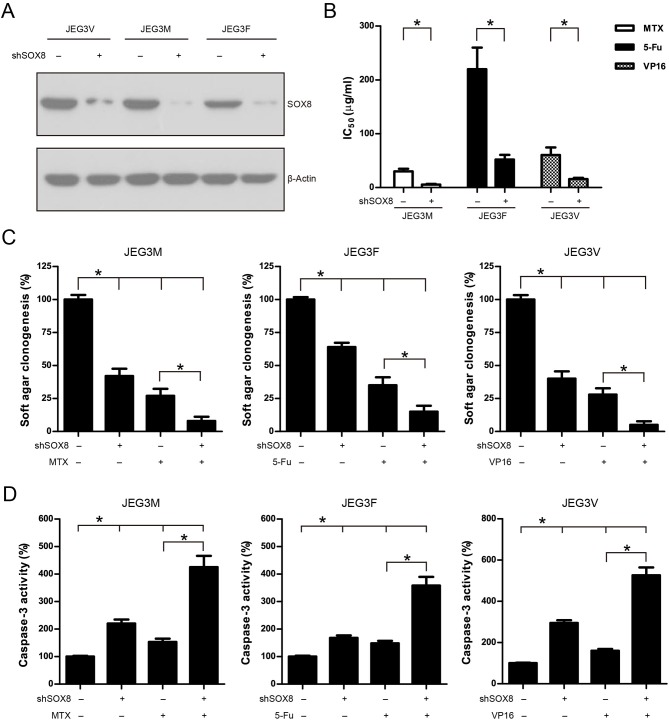
Knockdown of SOX8 attenuated drug resistance in JEG3 chemoresistant sublines. **(A)** Western blotting analysis on protein expression of SOX8 following lentivirus-mediated shRNA knockdown in JEG3 sublines. β-actin was used as loading control. **(B)** IC_50_-values of chemotherapeutic drugs in JEG3 chemoresistant sublines expressing Scr or shSOX8 shRNAs. *n* = 3, *P < 0.05. **(C)** Knockdown of SOX8 impaired soft agar clonogenesis after drug treatment (MTX: 10 μg/mL; 5-Fu: 50 μg/mL; VP16: 10 μg/mL) in JEG3 sublines. The colony formation in Scr group without drug treatment was regarded as 100%, respectively. *n* = 4, *P < 0.05. **(D)** Knockdown of SOX8 increased caspase-3 activity following drug treatment (MTX: 10 μg/mL; 5-Fu: 50 μg/mL; VP16: 10 μg/mL) for 48 h in JEG3 sublines. The caspase-3 activity in Scr group without drug treatment was regarded as 100%, respectively. *n* = 4, *P < 0.05.

To address the function of SOX8 in regulating drug resistance phenotype, JEG3 chemoresistant sublines were transduced with scramble (Scr) or specific shRNA targeting SOX8. The SOX8 expression was significantly decreased in three JEG3 sublines infected by SOX8 shRNA lentiviruses, as compared with Scr control (Figure 5A). The effect of SOX8 expression on the chemosensitivity of JEG3 sublines was assessed by CCK-8 assay. As shown in (Figure 5B), knockdown of SOX8 exhibited lower IC_50_-values, as compared with Scr in three sublines. Soft agar clonogenesis of JEG3 sublines was decreased in shSOX8 groups compared to Scr control groups (Figure 5C). Moreover, compared with Scr, knockdown of SOX8 reduced soft agar clonogenesis of JEG3 sublines after drug treatment (Figure 5C). Knockdown of SOX8 increased apoptosis-related caspase-3 activities in three sublines (Figure 5D). Impressively, more caspase-3 activities were detected in JEG3 sublines transduced with shSOX8 than in JEG3 cells transduced with Scr shRNA after drug treatment (Figure 5D).

### Over-Expression of SOX8 Promoted Drug Resistance in GTN Cell Lines

**Figure 6:**
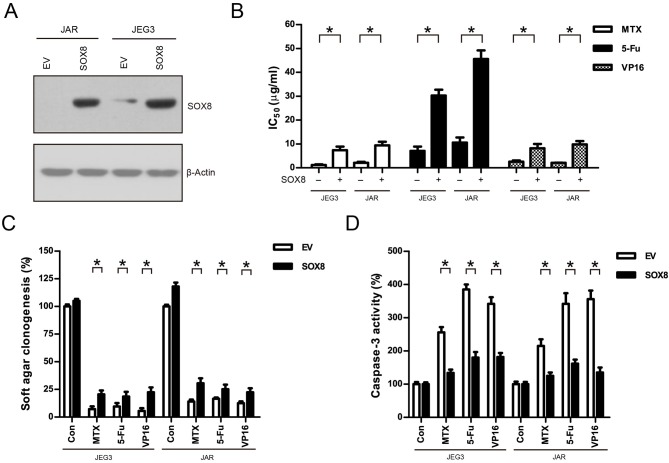
Over-expression of SOX8 promoted drug resistance in GTN cell lines. **(A)** Lentiviral transduction of SOX8 considerably increased their protein expression in JEG3 and JAR cells. β-actin was used as loading control. **(B)** IC_50_-values of chemotherapeutic drugs in JEG3 and JAR cells expressing SOX8 or EV. *n* = 3, *P < 0.05. **(C)** Over-expression of SOX8 rescued soft agar clonogenesis after drug treatment (MTX: 1 μg/mL; 5-Fu: 5 μg/mL; VP16: 2 μg/mL) in JEG3 and JAR cells. The colony formation in EV of each cell line without drug treatment was regarded as 100%, respectively. *n* = 4, *P < 0.05. **(D)** Over-expression of SOX8 attenuated caspase-3 activity following drug treatment (MTX: 1 μg/mL; 5-Fu: 5 μg/mL; VP16: 2 μg/mL) for 48 h in JEG3 and JAR cells. The caspase-3 activity in EV of each cell line without drug treatment was regarded as 100%, respectively. *n* = 4, *P < 0.05.

We also constitutively expressed SOX8 in JEG3 and JAR cells in order to evaluate the effect of SOX8 expression on the chemosensitivity of GTN cells (Figure 6A). As shown in (Figure 6B), higher IC_50_ was observed 48 h after drug treatment in SOX8 groups than in EV groups in JEG3 and JAR cells, respectively. Further, SOX8-expressing GTN cells exhibited increased soft agar clonogenesis compared with EV following drug treatment (Figure 6C). Nevertheless, less caspase-3 activities were detected in cells expressing SOX8 than in cells expressing EV 48 h after drug treatment (Figure 6D).

### Attenuation of ROS Induced by Drugs Might Be Associated With Drug Resistance in GTN Cells

**Figure 7:**
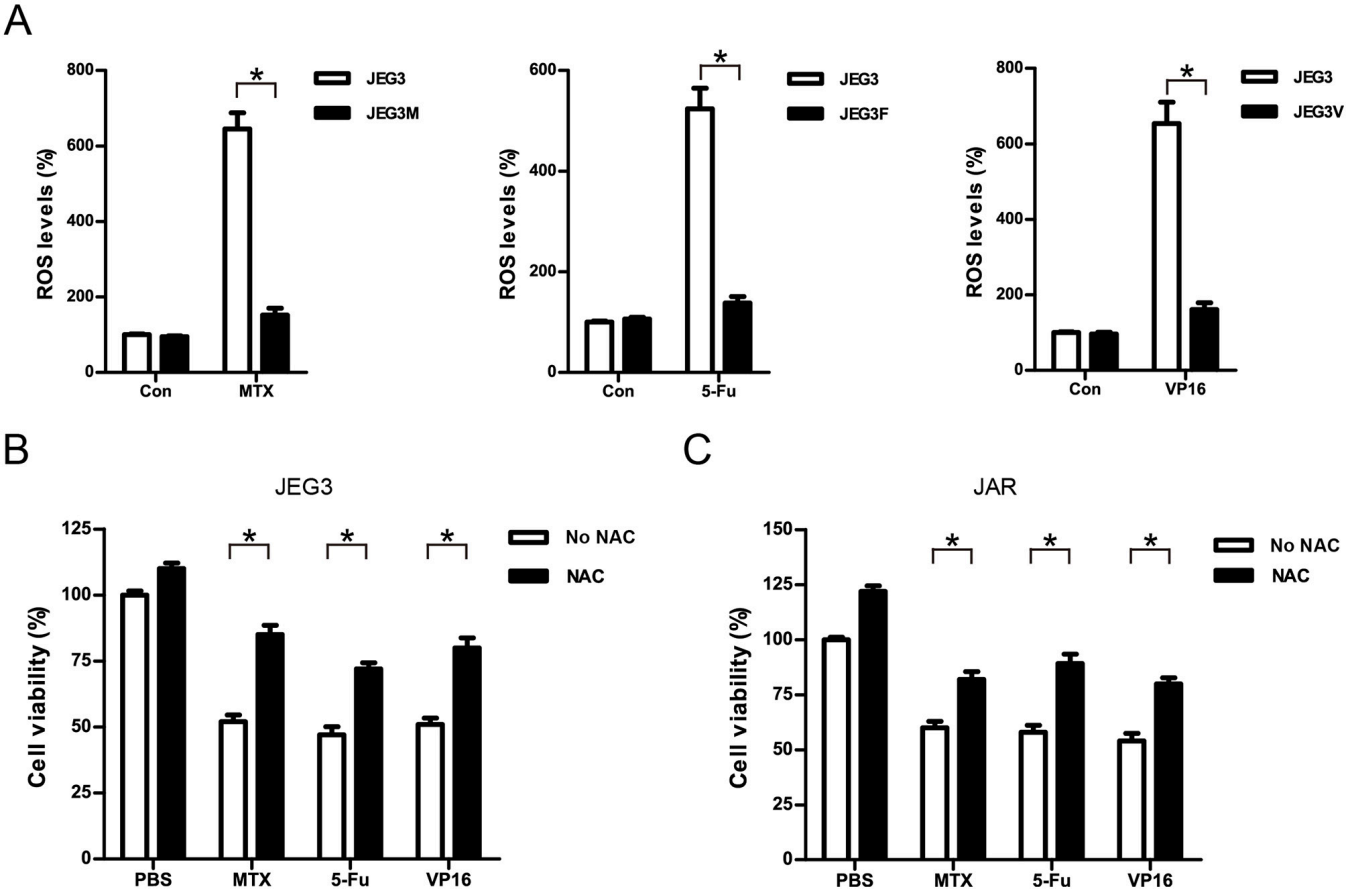
Attenuation of ROS induced by drugs might be associated with drug resistance in GTN cells. **(A)** ROS levels of JEG3 and JEG3 chemoresistant sublines after drug treatment. JEG3 and JEG3 sublines were treated with MTX (1 μg/mL), 5-Fu (5 μg/mL), or VP16 (2 μg/mL) for 48 h, respectively. ROS levels in JEG3 without drug treatment were regarded as 100%. *n* = 4, *P < 0.05. **(B)** Cytotoxicity induced by drugs was associated with ROS accumulation in JEG3 cells. JEG3 cells were treated with MTX (1 μg/mL), 5-Fu (5 μg/mL), or VP16 (2 μg/mL) alone or with NAC (5 mM) for 48 h, followed by CCK-8 assay to assess the cell viability. Cell viability in JEG3 without drug treatment was regarded as 100%. *n* = 4, *P < 0.05. **(C)** Cytotoxicity induced by drugs was associated with ROS accumulation in JAR cells. JAR cells were treated with MTX (1 μg/mL), 5-Fu (5 μg/mL), or VP16 (2 μg/mL) alone or with NAC (5 mM) for 48 h, followed by CCK-8 assay to assess the cell viability. Cell viability in JAR without drug treatment was regarded as 100%. *n* = 4, *P < 0.05.

Most of chemotherapeutic drugs could induce the accumulation of intracellular ROS to kill cancer cells (30). Attenuation of drug-induced ROS generation might be one of the important mechanisms of drug resistance in cancer cells (30). Our KEGG pathway and protein-protein interaction analysis indicated that signaling networks associated with cellular response to oxidative stress were commonly enriched in all the three chemoresistant JEG3 sublines (Figures 2C, 3A). Consistently, higher levels of ROS were detected in JEG3 than those in chemoresistant JEG3 sublines following drug treatment for 48 h (Figure 7A). To confirm the role of ROS on drug-induced cytotoxicity, we co-incubated drugs with ROS scavenger NAC for 48 h in JEG3 and JAR cells. The addition of NAC greatly increased cell viability following drug treatment in both JEG3 and JAR cells (Figures 7B,C).

### SOX8 Regulated the Expression of Antioxidant Enzymes and Reduced the Drug-Induced ROS Accumulation in GTN Cells

**Figure 8:**
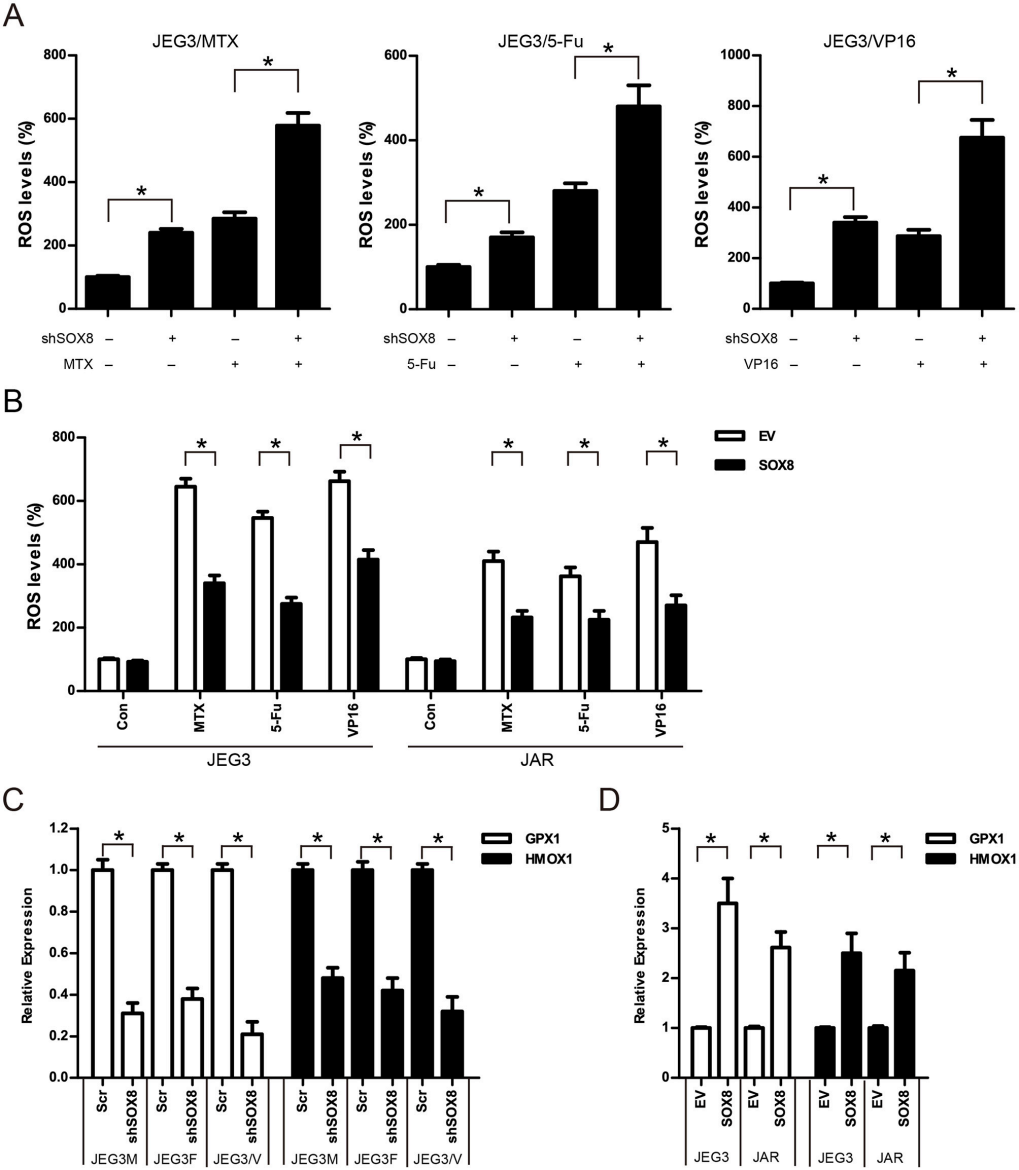
SOX8 regulated the expression of antioxidant enzymes and reduced the drug-induced ROS accumulation in GTN cells **(A)** Knockdown of SOX8 increased drug-induced ROS in JEG3 sublines. JEG3 sublines expressing Scr or shSOX8 shRNAs were treated with MTX (10 μg/mL), 5-Fu (50 μg/mL), or VP16 (10 μg/mL) for 48 h. The DCFDA fluorescence in Scr group of three JEG3 sublines without drug treatment was regarded as 100%, respectively. *n* = 4, *P < 0.05. **(B)** Over-expression of SOX8 attenuated drug-induced ROS in GTN cell lines. GTN cells (JEG3, JAR) expressing EV or SOX8 were treated with MTX (1 μg/mL), 5-Fu (5 μg/mL), or VP16 (2 μg/mL) for 48 h. The DCFDA fluorescence in EV group of JEG3 or JAR cells without drug treatment was regarded as 100%, respectively. *n* = 4, *P < 0.05. **(C)** Knockdown of SOX8 reduced the expression of antioxidant enzymes GPX1 and HMOX1 in JEG3 chemoresistant sublines. The gene expression of GPX1 or HMOX1 in Scr group of three JEG3 sublines was regarded as 100%, respectively. *n* = 3, *P < 0.05. **(D)** over-expression of SOX8 increased GPX1 and HMOX1 expression in JEG3 and JAR cells. The gene expression of GPX1 or HMOX1 in EV group of JEG3 or JAR cells was regarded as 100%, respectively. *n* = 3, *P < 0.05.

Further studies were conducted to evaluate the effect of SOX8 on intracellular ROS accumulation in GTN cells. As shown in (Figure 8A), knockdown of SOX8 markedly increased intracellular ROS levels compared to Scr control in JEG3 chemoresistant sublines. Higher ROS levels were also detected in JEG3 chemoresistant sublines expressing shSOX8 than those in Scr after drug treatment for 48 h (Figure 8A). In contrast, lower ROS levels were detected in GTN cells (JEG3, JAR) expressing SOX8 than those in EV after drug treatment for 48 h (Figure 8B). Glutathione peroxidase (GPXs) and heme oxygenase (HMOXs) are important antioxidant enzymes which metabolize intracellular ROS and promote drug resistance in cancer cells (31). Our proteomic analysis identified several upregulated antioxidant enzymes (GPX1, HMOX1, etc.) in chemoresistant JEG3 sublines (Figures 2C, 3A, Table S1). We further evaluated the effect of SOX8 on the expression of two antioxidant enzymes (GPX1, HMOX1) in GTN cells. As shown in (Figure 8C), knockdown of SOX8 reduced the expression of GPX1 and HMOX1 in JEG3 chemoresistant sublines. In contrast, over-expression of SOX8 increased GPX1 and HMOX1 expression in JEG3 and JAR cells (Figure 8D).

## Discussion

Resistance to chemotherapeutic protocols poses a significant challenge for GTN treatment (2, 32, 33). Currently, the mechanism of drug resistance in GTN still remains poorly understood. Recently, the advent of label-free quantitative proteomics has enabled robust quantification of proteomic experiments, albeit lack of data on drug resistance in GTN cell model. Herein, using TMT-based quantitative proteomic technique, we identified 5,704 proteins and quantified 4,997 proteins in JEG3 and its chemoresistant sublines and established drug resistance-related proteomic profiles, highlighting the advantage of quantitative proteomic technique in identifying drug resistance-related proteins. Although KEGG pathway analysis revealed differential patterns of pathway activation associated with drug resistance to individual drugs (MTX, 5-Fu, VP16), some common genes/pathways/signaling networks were indeed activated in all the three chemoresistant sublines. Several earlier reports indicated that ABCG2 could confer drug resistance to GTN cells by facilitating drug efflux (10, 34, 35). Consistently, increased ABCG2 expression was observed in our drug resistance-related proteomic dataset. Nevertheless, multiple molecular pathways/biological processes (response to toxic substance, cellular response to oxidative stress, myeloid cell activation involved in immune response, ameboidal-type cell migration, etc.) were also identified in all the three chemoresistant GTN cells. Therefore, multiple regulatory mechanisms might potentially contribute to the development of drug resistance in GTN cells. Elucidating these mechanisms may help to develop more effective therapeutic strategies for GTN treatment in the future.

SOX8 exerts an important biological function during embryonic brain development, despite its expression is low in normal adult human tissues (36, 37). Recently, SOX8 has been shown to be highly expressed in several cancers, and has been shown to be a potential oncogene. Zhang et al. (38) showed that SOX8 promoted cellular proliferation and enhanced tumor growth in hepatocellular carcinomas. SOX8 is also found to be a signature of basal-like immune-suppressed triple-negative breast cancer; amplification of SOX8 significantly shortens the survival of patients with breast cancer (39, 40). Xie et al. (41) indicated that SOX8 could activate Wnt/β-catenin pathway to promote cisplatin-induced EMT in tongue squamous cell carcinoma. However, the regulatory role of SOX8 on drug resistance of cancer cells still awaits further investigation. In this study, we showed that SOX8 might play an important role in regulating drug resistance in GTN cells, as knockdown of SOX8 could attenuate drug resistance through reducing cell viability, impairing soft agar clonogenesis, and inducing apoptosis following drug treatment in JEG3 chemoresistant sublines. In contrast, over-expression of SOX8 promoted drug resistance through enhancing cell survival, promoting soft agar clonogenesis, and attenuating apoptosis in JEG3 and JAR cells. Therefore, targeting SOX8 could potentially sensitize GTN cells to chemotherapeutic drugs, which may warrant further investigation as potential therapeutic targets for GTN.

ROS production plays an important role in mediating cytotoxicity induced by chemotherapy (30, 42, 43). Lim et al. (43, 44) showed that chrysophanol and coumestrol could induce apoptosis through regulation of ROS in GTN cells. Ham et al. (45) showed that silibinin stimluated apoptosis partly by inducing ROS in GTN cells. Zhao et al. (46) showed that selenocystine inhibits GTN cell line JEG3 growth by inducing ROS-mediated cell cycle arrest and apoptosis. Our bioinformatics analysis indicated that ROS-related mechanisms might be associated with drug resistance in GTN cells. Consistently, compared with JEG3, reduced ROS generation was observed in JEG3 chemoresistant sublines after drug treatment. Therefore, attenuation of ROS accumulation induced by chemotherapeutic drugs might be crucial for the development of drug resistance and cell survival in GTN cells. Our further findings also suggested that SOX8 might be a potential regulator of ROS homeostasis, as SOX8 expression affected ROS accumulation following drug treatment in GTN cell models. Therefore, SOX8 might promote drug resistance through modulating ROS homeostasis in GTN cells. Further, our findings showed that SOX8 regulated the expression of antioxidant enzymes (GPX1, HMOX1) in GTN cell models, which might help to attenuate drug-induced ROS accumulation and promote GTN cell survival after drug treatment. Future studies on SOX8 function might be valuable toward the development of novel drugs for GTN.

In summary, our study established drug resistance related proteomic profiles, and revealed multiple genes/molecular pathways/signaling networks potentially involved in regulating drug resistance in GTN cells. We further showed that SOX8 might be a potential regulator of drug resistance through attenuating the accumulation of ROS induced by chemotherapeutic drugs in GTN cells. Our findings might have potential clinical value in targeting SOX8-regulated signaling pathway to overcome drug resistance in GTN.

## Data Availability Statement

The original contributions presented in the study are included in the article/Supplementary Material, further inquiries can be directed to the corresponding author/s.

## Ethics Statement

The research protocols were approved by the research Ethics Committee in Xiangya Hospital, Central South University.

## Author Contributions

DS designed the study: FJ performed the experiments: ZP, YZ, and FJ analyzed the data: DS wrote the manuscript. All authors have read and approved the final version of the manuscript.

## Conflict of Interest

The authors declare that the research was conducted in the absence of any commercial or financial relationships that could be construed as a potential conflict of interest.

## Abbreviations

GTN: Gestational trophoblastic neoplasia
TMT: Tandem mass tag
MTX: Methotrexate
5-Fu: Fluorouracil
VP16: Etoposide
ROS: Reactive oxygen species
LC-MS/MS: Liquid chromatography-tandem mass spectrometry
DCFDA: 2: 7-Dichlorodi-hydrofluorescein diacetate
NAC: N-Acetyl-L-cysteine
SOX8: SRY-Box Transcription Factor 8
GPX1: Glutathione Peroxidase 1
HMOX1: Heme Oxygenase 1.
