# Supplementary material for: Quantitative Proteomic Profiling Identifies SOX8 as Novel Regulator of Drug Resistance in Gestational Trophoblastic Neoplasia
Source: Front Oncol. 2020 Apr 28;10:557. doi: 10.3389/fonc.2020.00557 (PMC7198745; doi:10.3389/fonc.2020.00557)
Supplement: Supplementary file 1 [file Data_Sheet_1.PDF]

Supplementary files

Supplementary Methods

Oligonucleotide sequences of SOX8 and scramble shRNAs:

shSOX8: 5’ TTTGAGGTGGTGGGATTA 3’

Scramble: 5’GTCACGATAAGACAATGAT 3’

Primer sequences for Real-time PCR:

GPX1:

Forward: 5’ CAGTCGGTGTATGCCTTCTCG 3’

Reverse: 5’ GAGGGACGCCACATTCTCG 3’

HMOX1:

Forward: 5’ AAGACTGCGTTCCTGCTCAAC 3’

Reverse: 5’ AAAGCCCTACAGCAACTGTCG 3’

β-Actin:

Forward: 5’ CATGTACGTTGCTATCCAGGC 3’

Reverse: 5’ CTCCTTAATGTCACGCACGAT 3’

Supplementary Figures

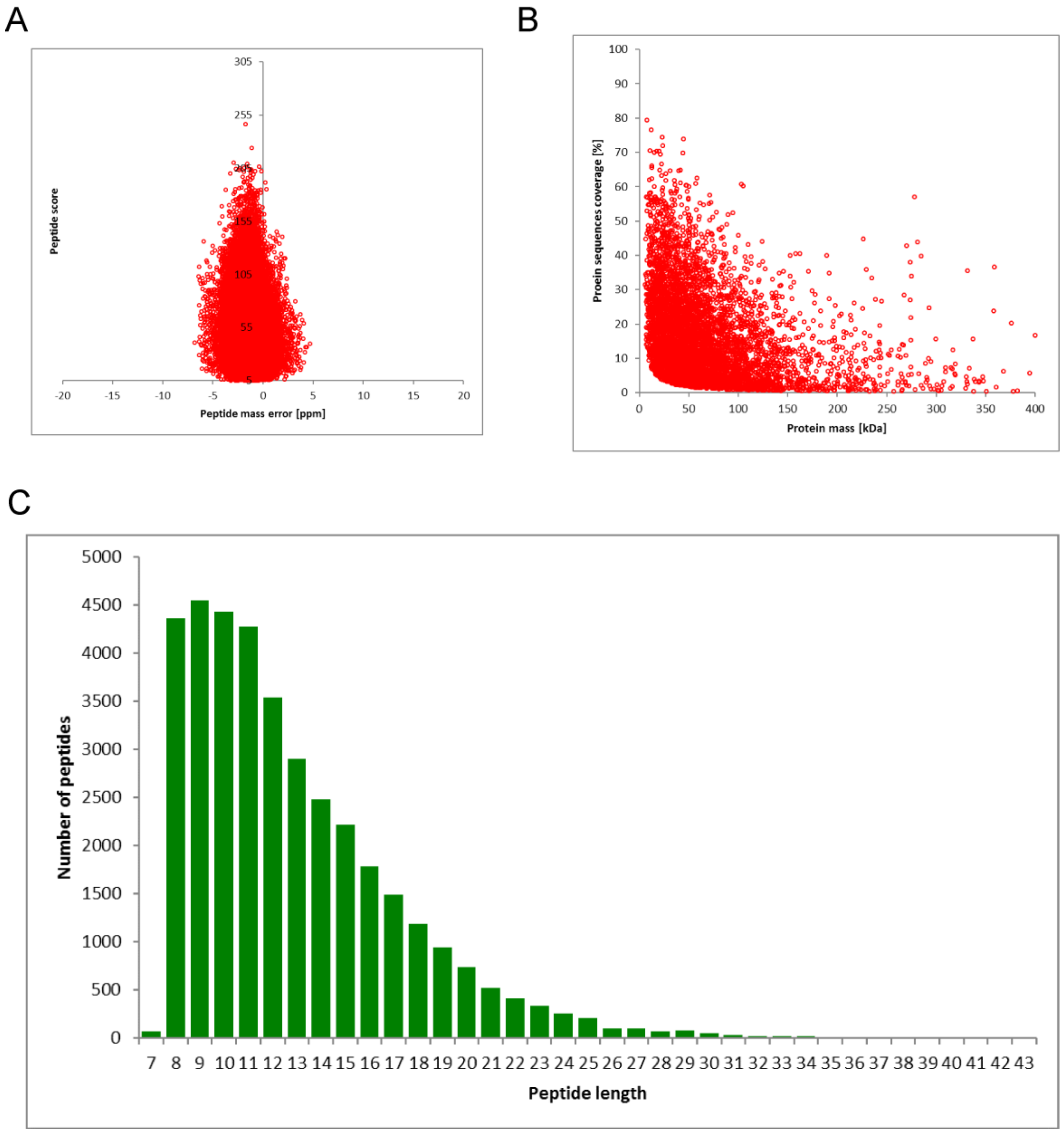

Figure S1. **A**. Distribution of mass error of the MS data **B**. Peptide sequence coverage of the MS data. **C.** Distribution of peptide length of the MS data.

Table S1

Protein accession	Protein description	Gene name	MW [kDa]	Score	Coverage [%]	Peptides	PSMs	Unique peptides	JEG3-1	JEG3-2	JEG3/VP 16-1	JEG3/VP 16-2	JEG3/5-Fu-1	JEG3/5-Fu-2	JEG3/MTX-1	JEG3/MTX-2	JEG3/VP 16/JEG3 Ratio	JEG3/5-Fu/JEG3 Ratio	JEG3/MTX/JEG3 Ratio
Q6PD74	Alpha- and gamma-adaptin-binding prote	AAGAB	34.593	6.7855	2.9	1	1	1									#DIV/0!	#DIV/0!	#DIV/0!
Q96IU4	Protein ABHD14B OS=Homo sapiens O	ABHD14B	22.345	8.311	4.8	1	1	1									#DIV/0!	#DIV/0!	#DIV/0!
Q5VST6	Alpha/beta hydrolase domain-containing	ABHD17B	32.214	12.304	8	2	2	2									#DIV/0!	#DIV/0!	#DIV/0!
Q9BR61	Acyl-CoA-binding domain-containing pr	ACBD6	31.15	13.535	9.2	2	2	2									#DIV/0!	#DIV/0!	#DIV/0!
O14734	Acyl-coenzyme A thioesterase 8 OS=Hor	ACOT8	35.914	19.878	11	3	4	3									#DIV/0!	#DIV/0!	#DIV/0!
P13686	Tartrate-resistant acid phosphatase type 5	ACP5	36.598	8.3089	5.8	1	1	1									#DIV/0!	#DIV/0!	#DIV/0!
Q9NPH0	Lysophosphatidic acid phosphatase type	ACP6	48.886	6.894	2.6	1	1	1									#DIV/0!	#DIV/0!	#DIV/0!
Q04771	Activin receptor type-1 OS=Homo sapien	ACVR1	57.152	7.2188	2.4	1	1	1									#DIV/0!	#DIV/0!	#DIV/0!
Q9H013	Disintegrin and metalloproteinase domai	ADAM19	105	6.4887	1.4	1	1	1									#DIV/0!	#DIV/0!	#DIV/0!
Q6UY14	ADAMTS-like protein 4 OS=Homo sapie	ADAMTSL	116.54	12.32	2.6	2	2	2									#DIV/0!	#DIV/0!	#DIV/0!
Q96EY9	Probable inactive tRNA-specific adenosi	ADAT3	38.07	6.6358	3.7	1	1	1									#DIV/0!	#DIV/0!	#DIV/0!
O43306	Adenylate cyclase type 6 OS=Homo sapi	ADCY6	130.61	6.1454	0.8	1	1	1									#DIV/0!	#DIV/0!	#DIV/0!
Q8TED9	Actin filament-associated protein 1-like 1	AFAP1L1	86.431	12.427	2.3	2	2	2									#DIV/0!	#DIV/0!	#DIV/0!
Q96P47	Arf-GAP with GTPase, ANK repeat and I	AGAP3	95.043	7.1249	2.2	1	1	1									#DIV/0!	#DIV/0!	#DIV/0!
O95081	Arf-GAP domain and FG repeat-containi	AGFG2	48.962	6.8986	4.4	2	2	1									#DIV/0!	#DIV/0!	#DIV/0!
Q9NUQ2	1-acyl-sn-glycerol-3-phosphate acyltrans	AGPAT5	42.072	12.222	4.9	2	2	2									#DIV/0!	#DIV/0!	#DIV/0!
Q9UPW5	Cytosolic carboxypeptidase 1 OS=Homo	AGTPBP1	138.45	22.624	2.2	2	2	2									#DIV/0!	#DIV/0!	#DIV/0!
Q96HN2	Adenosylhomocysteinase 3 OS=Homo sa	AHCYL2	66.72	11.942	15.9	10	14	2									#DIV/0!	#DIV/0!	#DIV/0!
Q96IF1	LIM domain-containing protein ajuba OS	AJUBA	56.933	7.2519	1.9	1	1	1									#DIV/0!	#DIV/0!	#DIV/0!
O60218	Aldo-keto reductase family 1 member B1	AKR1B10	36.019	5.9955	5.7	1	1	1									#DIV/0!	#DIV/0!	#DIV/0!
Q96JD6	1,5-anhydro-D-fructose reductase OS=H	AKR1E2	36.589	6.1563	2.5	1	1	1									#DIV/0!	#DIV/0!	#DIV/0!
P05091	Aldehyde dehydrogenase, mitochondrial	ALDH2	56.381	21.874	7.5	3	3	3									#DIV/0!	#DIV/0!	#DIV/0!
Q96BT7	Alkylated DNA repair protein alkB homo	ALKBH8	75.208	6.056	1.2	1	1	1									#DIV/0!	#DIV/0!	#DIV/0!
P09917	Arachidonate 5-lipoxygenase OS=Homo	ALOX5	77.982	15.332	4.2	2	3	2									#DIV/0!	#DIV/0!	#DIV/0!
P05186	Alkaline phosphatase, tissue-nonspecific	ALPL	57.304	6.5339	2.3	1	1	1									#DIV/0!	#DIV/0!	#DIV/0!
P05187	Alkaline phosphatase, placental type OS=	ALPP	57.953	8.8076	21.3	10	13	1									#DIV/0!	#DIV/0!	#DIV/0!
P17707	S-adenosylmethionine decarboxylase pro	AMD1	38.339	6.4365	2.4	1	1	1									#DIV/0!	#DIV/0!	#DIV/0!
Q9Y2J4	Angiomotin-like protein 2 OS=Homo sap	AMOTL2	85.763	6.4235	1.8	1	1	1									#DIV/0!	#DIV/0!	#DIV/0!
Q01433	AMP deaminase 2 OS=Homo sapiens OX	AMPD2	100.69	7.4533	1.1	1	1	1									#DIV/0!	#DIV/0!	#DIV/0!
Q9UJX5	Anaphase-promoting complex subunit 4	ANAPC4	92.115	16.404	2.6	2	3	2									#DIV/0!	#DIV/0!	#DIV/0!
Q9UPM8	AP-4 complex subunit epsilon-1 OS=Ho	AP4E1	127.29	6.3661	0.9	1	1	1									#DIV/0!	#DIV/0!	#DIV/0!
Q9Y587	AP-4 complex subunit sigma-1 OS=Hom	AP4S1	17.005	6.33	6.9	1	1	1									#DIV/0!	#DIV/0!	#DIV/0!
Q9UBZ4	DNA-(apurinic or apyrimidinic site) lyase	APEX2	57.4	14.553	4.1	1	1	1									#DIV/0!	#DIV/0!	#DIV/0!
Q9BQE5	Apolipoprotein L2 OS=Homo sapiens O	APOL2	37.092	9.7732	6.5	2	1	1									#DIV/0!	#DIV/0!	#DIV/0!
Q9NWB6	Arginine and glutamate-rich protein 1 OS	ARGLU1	33.216	11.975	5.9	2	2	2									#DIV/0!	#DIV/0!	#DIV/0!
Q8IWW6	Rho GTPase-activating protein 12 OS=H	ARHGAP1	96.253	22.745	4.3	4	4	4									#DIV/0!	#DIV/0!	#DIV/0!
P85298	Rho GTPase-activating protein 8 OS=Ho	ARHGAP8	53.483	10.856	4.1	2	2	2									#DIV/0!	#DIV/0!	#DIV/0!
Q6ZSZ5	Rho guanine nucleotide exchange factor	ARHGEF1	130.78	23.674	4.9	4	4	4									#DIV/0!	#DIV/0!	#DIV/0!
Q66PJ3	ADP-ribosylation factor-like protein 6-in	ARL6IP4	44.915	12.411	4.5	1	1	1									#DIV/0!	#DIV/0!	#DIV/0!
Q96BM9	ADP-ribosylation factor-like protein 8A	ARL8A	21.416	6.5558	14	2	3	1									#DIV/0!	#DIV/0!	#DIV/0!
Q5T2E6	Armadillo-like helical domain-containing	ARMH3	78.71	18.987	4.8	3	3	3									#DIV/0!	#DIV/0!	#DIV/0!
P32121	Beta-arrestin-2 OS=Homo sapiens OX=9	ARRB2	46.105	17.104	7.3	2	2	2									#DIV/0!	#DIV/0!	#DIV/0!
Q93070	Ecto-ADP-ribosyltransferase 4 OS=Hom	ART4	35.877	6.4457	2.9	1	1	1									#DIV/0!	#DIV/0!	#DIV/0!
Q9ULH1	Arf-GAP with SH3 domain, ANK repeat	ASAP1	125.5	16.386	2	2	2	2									#DIV/0!	#DIV/0!	#DIV/0!
Q9NWX5	Ankyrin repeat and SOCS box protein 6	ASB6	47.135	23.959	11.6	4	4	4									#DIV/0!	#DIV/0!	#DIV/0!
Q8IZT6	Abnormal spindle-like microcephaly-assc	ASPM	409.8	12.605	0.9	2	2	2									#DIV/0!	#DIV/0!	#DIV/0!
Q76L83	Putative Polycomb group protein ASXL2	ASXL2	153.82	17.549	3.3	3	3	3									#DIV/0!	#DIV/0!	#DIV/0!
Q96QE3	ATPase family AAA domain-containing	ATAD5	207.57	17.437	1.7	3	2	2									#DIV/0!	#DIV/0!	#DIV/0!
P18846	Cyclic AMP-dependent transcription fact	ATF1	29.232	6.7248	12.9	2	2	1									#DIV/0!	#DIV/0!	#DIV/0!
Q6VMQ6	Activating transcription factor 7-interacti	ATF7IP	136.39	8.4227	0.9	1	1	1									#DIV/0!	#DIV/0!	#DIV/0!
O94817	Ubiquitin-like protein ATG12 OS=Homo	ATG12	15.113	5.9588	6.4	1	1	1									#DIV/0!	#DIV/0!	#DIV/0!
O75143	Autophagy-related protein 13 OS=Homo	ATG13	56.571	6.4629	1.9	1	1	1									#DIV/0!	#DIV/0!	#DIV/0!
Q2TAZ0	Autophagy-related protein 2 homolog A	ATG2A	212.86	13.882	1	2	2	2									#DIV/0!	#DIV/0!	#DIV/0!
Q9Y4P1	Cysteine protease ATG4B OS=Homo sap	ATG4B	44.294	7.1188	3.8	1	1	1									#DIV/0!	#DIV/0!	#DIV/0!
Q8WXE1	ATR-interacting protein OS=Homo sapie	ATRIP	85.837	7.5016	1.6	1	1	1									#DIV/0!	#DIV/0!	#DIV/0!
O60513	Beta-1,4-galactosyltransferase 4 OS=Ho	B4GALT4	40.041	24.511	9.3	3	3	3									#DIV/0!	#DIV/0!	#DIV/0!
Q9NWV8	BRISC and BRCA1-A complex member	BABAM1	36.56	22.204	8.2	2	3	2									#DIV/0!	#DIV/0!	#DIV/0!
Q9NXR7	BRISC and BRCA1-A complex member	BABAM2	43.551	25.327	12	4	4	4									#DIV/0!	#DIV/0!	#DIV/0!
O75531	Barrier-to-autointegration factor OS=Ho	BANF1	10.058	26.259	27	1	1	1									#DIV/0!	#DIV/0!	#DIV/0!
Q9H6U6	Breast carcinoma-amplified sequence 3 C	BCAS3	101.24	11.586	2.4	2	2	2									#DIV/0!	#DIV/0!	#DIV/0!
Q8WUZ0	B-cell CLL/lymphoma 7 protein family m	BCL7C	23.468	13.799	10.1	2	2	1									#DIV/0!	#DIV/0!	#DIV/0!
Q9BUT1	3-hydroxybutyrate dehydrogenase type 2	BDH2	26.724	6.5803	4.9	1	1	1									#DIV/0!	#DIV/0!	#DIV/0!
O15392	Baculoviral IAP repeat-containing protei	BIRC5	16.389	14.548	9.2	1	1	1									#DIV/0!	#DIV/0!	#DIV/0!
P78537	Biogenesis of lysosome-related organelle	BLOC1S1	17.262	13.219	13.7	2	2	2									#DIV/0!	#DIV/0!	#DIV/0!
Q6QNY0	Biogenesis of lysosome-related organelle	BLOC1S3	21.255	8.5241	7.9	1	1	1									#DIV/0!	#DIV/0!	#DIV/0!
Q12982	BCL2/adenovirus E1B 19 kDa protein-in	BNIP2	36.017	6.0551	4.1	1	1	1									#DIV/0!	#DIV/0!	#DIV/0!
Q96IK1	Biorientation of chromosomes in cell div	BOD1	19.196	7.9621	21.6	3	3	1									#DIV/0!	#DIV/0!	#DIV/0!
Q969J3	BLOC-1-related complex subunit 5 OS=	BORCS5	22.222	7.0872	6.1	1	1	1									#DIV/0!	#DIV/0!	#DIV/0!
Q96GS4	BLOC-1-related complex subunit 6 OS=	BORCS6	37.226	12.263	5	2	2	2									#DIV/0!	#DIV/0!	#DIV/0!
Q86WA6	Valacyclovir hydrolase OS=Homo sapien	BPHL	32.542	11.273	6.2	2	1	1									#DIV/0!	#DIV/0!	#DIV/0!
Q9ULD4	Bromodomain and PHD finger-containin	BRPF3	135.74	13.096	1.7	2	2	1									#DIV/0!	#DIV/0!	#DIV/0!
Q9BRD0	BUD13 homolog OS=Homo sapiens OX	BUD13	70.52	10.941	3.9	2	1	1									#DIV/0!	#DIV/0!	#DIV/0!
Q96A22	Uncharacterized protein C11orf52 OS=H	C11orf52	13.92	6.3029	11.4	1	1	1									#DIV/0!	#DIV/0!	#DIV/0!
E9PRG8	Uncharacterized protein C11orf98 OS=H	C11orf98	13.798	7.491	10.7	1	1	1									#DIV/0!	#DIV/0!	#DIV/0!
Q9NWQ9	Uncharacterized protein C14orf119 OS=	C14orf119	16.009	6.0156	5.7	1	1	1									#DIV/0!	#DIV/0!	#DIV/0!
Q5BKX5	UPF0692 protein C19orf54 OS=Homo s	C19orf54	37.778	7.6774	4.8	1	1	1									#DIV/0!	#DIV/0!	#DIV/0!
Q9NSG2	Uncharacterized protein C1orf112 OS=H	C1orf112	96.553	17.417	5	3	3	3									#DIV/0!	#DIV/0!	#DIV/0!
Q9GZN8	UPF0687 protein C20orf27 OS=Homo s	C20orf27	19.291	10.652	12.1	1	1	1									#DIV/0!	#DIV/0!	#DIV/0!
Q86YS7	C2 domain-containing protein 5 OS=Ho	C2CD5	110.45	6.894	1.1	1	1	1									#DIV/0!	#DIV/0!	#DIV/0!
Q9BVC5	Ashwin OS=Homo sapiens OX=9606 GN	C2orf49	25.858	13.671	12.1	2	2	2									#DIV/0!	#DIV/0!	#DIV/0!
Q2NKX9	UPF0561 protein C2orf68 OS=Homo sa	C2orf68	18.751	6.436	6	1	1	1									#DIV/0!	#DIV/0!	#DIV/0!
Q8N8R5	UPF0565 protein C2orf69 OS=Homo sa	C2orf69	43.448	6.24	2.1	1	1	1									#DIV/0!	#DIV/0!	#DIV/0!
Q8NDZ4	Deleted in autism protein 1 OS=Homo sa	C3orf58	49.481	9.4732	3.3	1	1	1									#DIV/0!	#DIV/0!	#DIV/0!
Q5BLP8	Neuropeptide-like protein C4orf48 OS=	C4orf48	10.17	12.209	18.9	2	2	2									#DIV/0!	#DIV/0!	#DIV/0!
Q8N0T1	Uncharacterized protein C8orf59 OS=Ho	C8orf59	11.456	12.985	18	2	3	2									#DIV/0!	#DIV/0!	#DIV/0!
Q8IXQ3	Uncharacterized protein C9orf40 OS=Ho	C9orf40	21.063	17.314	14.4	2	2	2									#DIV/0!	#DIV/0!	#DIV/0!
Q9H9S4	Calcium-binding protein 39-like OS=Ho	CAB39L	39.087	26.1	11.9	4	5	4									#DIV/0!	#DIV/0!	#DIV/0!
Q9BY67	Cell adhesion molecule 1 OS=Homo sapi	CADM1	48.509	21.988	11.1	3	3	3									#DIV/0!	#DIV/0!	#DIV/0!
Q8WVQ1	Soluble calcium-activated nucleotidase 1	CANT1	44.839	14.019	6.2	2	2	2									#DIV/0!	#DIV/0!	#DIV/0!
Q13938	Calcyphosin OS=Homo sapiens OX=960	CAPS	20.967	14.243	12.2	2	2	2									#DIV/0!	#DIV/0!	#DIV/0!
Q92851	Caspase-10 OS=Homo sapiens OX=960	CASP10	58.95	7.0285	2.7	1	1	1									#DIV/0!	#DIV/0!	#DIV/0!
P55210	Caspase-7 OS=Homo sapiens OX=9606	CASP7	34.276	6.6978	3.3	1	1	1									#DIV/0!	#DIV/0!	#DIV/0!
Q8WTX7	Cytosolic arginine sensor for mTORC1 s	CASTOR1	36.275	6.7411	3.3	1	1	1									#DIV/0!	#DIV/0!	#DIV/0!
Q9ULV8	E3 ubiquitin-protein ligase CBL-C OS=H	CBLC	52.455	7.1911	3.6	1	1	1									#DIV/0!	#DIV/0!	#DIV/0!
P16152	Carbonyl reductase [NADPH] 1 OS=Hon	CBR1	30.375	7.5666	5.4	1	1	1									#DIV/0!	#DIV/0!	#DIV/0!
Q7Z3E2	Coiled-coil domain-containing protein 1	CCDC186	103.69	13.079	2.4	2	2	2									#DIV/0!	#DIV/0!	#DIV/0!
Q567U6	Coiled-coil domain-containing protein 9	CCDC93	73.197	33.035	7.3	5	5	5									#DIV/0!	#DIV/0!	#DIV/0!
P24863	Cyclin-C OS=Homo sapiens OX=9606 C	CCNC	33.242	7.9322	6	1	1	1									#DIV/0!	#DIV/0!	#DIV/0!
P30281	G1/S-specific cyclin-D3 OS=Homo sapie	CCND3	32.519	7.577	3.1	1	1	1									#DIV/0!	#DIV/0!	#DIV/0!
P24864	G1/S-specific cyclin-E1 OS=Homo sapie	CCNE1	47.077	12.099	4.9	2	2	2									#DIV/0!	#DIV/0!	#DIV/0!
O60583	Cyclin-T2 OS=Homo sapiens OX=9606	CCNT2	81.028	11.217	2.5	2	2	2									#DIV/0!	#DIV/0!	#DIV/0!
P29016	T-cell surface glycoprotein CD1b OS=H	CD1B	36.939	6.1442	2.4	1	1	1									#DIV/0!	#DIV/0!	#DIV/0!
P15529	Membrane cofactor protein OS=Homo sa	CD46	43.747	6.8589	3.8	1	1	1									#DIV/0!	#DIV/0!	#DIV/0!
P21926	CD9 antigen OS=Homo sapiens OX=960	CD9	25.416	7.5038	4.4	1	1	1									#DIV/0!	#DIV/0!	#DIV/0!
P30304	M-phase inducer phosphatase 1 OS=Hon	CDC25A	59.086	6.4121	2.1	1	1	1									#DIV/0!	#DIV/0!	#DIV/0!
Q96SN8	CDK5 regulatory subunit-associated prot	CDK5RAP	215.04	14.413	1.1	2	2	2									#DIV/0!	#DIV/0!	#DIV/0!
P49336	Cyclin-dependent kinase 8 OS=Homo sa	CDK8	53.283	6.4788	1.7	1	1	1									#DIV/0!	#DIV/0!	#DIV/0!
Q8N0S6	Centromere protein L OS=Homo sapiens	CENPL	38.997	7.9532	3.5	1	1	1									#DIV/0!	#DIV/0!	#DIV/0!
Q9UPN4	Centrosomal protein of 131 kDa OS=Ho	CEP131	122.15	28.878	5.1	4	4	4									#DIV/0!	#DIV/0!	#DIV/0!
Q9Y4F5	Centrosomal protein of 170 kDa protein	CEP170B	171.69	23.974	3.1	4	4	3									#DIV/0!	#DIV/0!	#DIV/0!
Q12873	Chromodomain-helicase-DNA-binding p	CHD3	226.59	23.692	3.2	6	7	2									#DIV/0!	#DIV/0!	#DIV/0!
Q96EP1	E3 ubiquitin-protein ligase CHFR OS=H	CHFR	73.386	6.1192	1.4	1	1	1									#DIV/0!	#DIV/0!	#DIV/0!
O15111	Inhibitor of nuclear factor kappa-B kinas	CHUK	84.639	18.683	3.2	2	2	2									#DIV/0!	#DIV/0!	#DIV/0!
Q86X95	Corepressor interacting with RBPJ 1 OS	CIR1	52.313	17.251	6.2	3	3	3									#DIV/0!	#DIV/0!	#DIV/0!
Q8N2M8	CLK4-associating serine/arginine rich pr	CLASRP	77.16	32.056	8.6	4	4	4									#DIV/0!	#DIV/0!	#DIV/0!
Q96S66	Chloride channel CLIC-like protein 1 OS	CLCC1	62.022	13.039	3.8	2	2	2									#DIV/0!	#DIV/0!	#DIV/0!
P51790	H(+)/Cl(-) exchange transporter 3 OS=H	CLCN3	90.965	8.3144	2.2	1	1	1									#DIV/0!	#DIV/0!	#DIV/0!
Q9NY35	Claudin domain-containing protein 1 OS	CLDND1	28.603	6.184	3.6	1	1	1									#DIV/0!	#DIV/0!	#DIV/0!
P49759	Dual specificity protein kinase CLK1 OS	CLK1	57.29	7.5168	2.5	1	1	1									#DIV/0!	#DIV/0!	#DIV/0!
Q9NWW5	Ceroid-lipofuscinosis neuronal protein 6	CLN6	35.919	8.8713	6.8	1	1	1									#DIV/0!	#DIV/0!	#DIV/0!
Q9UBY8	Protein CLN8 OS=Homo sapiens OX=9	CLN8	32.787	6.8558	6.3	1	1	1									#DIV/0!	#DIV/0!	#DIV/0!
Q92989	Polyribonucleotide 5'-hydroxyl-kinase Cl	CLP1	47.645	12.034	4.5	2	2	2									#DIV/0!	#DIV/0!	#DIV/0!
Q7Z7K0	COX assembly mitochondrial protein ho	CMC1	12.49	14.147	24.5	2	2	2									#DIV/0!	#DIV/0!	#DIV/0!
Q8IYT2	Cap-specific mRNA (nucleoside-2'-O-)-m	CMTR2	88.119	11.891	2.2	2	2	2									#DIV/0!	#DIV/0!	#DIV/0!
Q8N9A8	Nuclear envelope phosphatase-regulatory	CNEP1R1	14.267	8.2222	12	1	1	1									#DIV/0!	#DIV/0!	#DIV/0!
Q9H8M5	Metal transporter CNNM2 OS=Homo sa	CNNM2	96.622	24.674	5	4	5	1									#DIV/0!	#DIV/0!	#DIV/0!
Q9Y2R0	Cytochrome c oxidase assembly factor 3	COA3	11.731	12.963	17	2	2	2									#DIV/0!	#DIV/0!	#DIV/0!
O75128	Protein cordon-bleu OS=Homo sapiens C	COBL	135.62	22.836	2.3	2	2	2									#DIV/0!	#DIV/0!	#DIV/0!
Q9GZQ3	COMM domain-containing protein 5 OS	COMMD5	24.67	8.7091	4.9	1	1	1									#DIV/0!	#DIV/0!	#DIV/0!
Q7Z4G1	COMM domain-containing protein 6 OS	COMMD6	9.638	6.868	12.9	1	1	1									#DIV/0!	#DIV/0!	#DIV/0!
P21964	Catechol O-methyltransferase OS=Homo	COMT	30.037	10.8	6.3	1	1	1									#DIV/0!	#DIV/0!	#DIV/0!
Q9NZJ6	Ubiquinone biosynthesis O-methyltransfe	COQ3	41.054	8.1731	4.3	1	1	1									#DIV/0!	#DIV/0!	#DIV/0!
Q5HYK3	2-methoxy-6-polyprenyl-1,4-benzoquinol	COQ5	37.14	30.47	19.6	4	4	4									#DIV/0!	#DIV/0!	#DIV/0!
Q9Y2Z9	Ubiquinone biosynthesis monooxygenase	COQ6	50.869	6.144	2.1	1	1	1									#DIV/0!	#DIV/0!	#DIV/0!
Q17RY0	Cytoplasmic polyadenylation element-bir	CPEB4	80.151	11.012	3	2	2	2									#DIV/0!	#DIV/0!	#DIV/0!
Q96FN4	Copine-2 OS=Homo sapiens OX=9606 C	CPNE2	61.189	6.6275	3.1	2	3	1									#DIV/0!	#DIV/0!	#DIV/0!
Q86YQ8	Copine-8 OS=Homo sapiens OX=9606 C	CPNE8	63.107	8.1515	4.3	2	3	1									#DIV/0!	#DIV/0!	#DIV/0!
Q9BRF8	Serine/threonine-protein phosphatase CP	CPPED1	35.548	6.4192	3.5	1	1	1									#DIV/0!	#DIV/0!	#DIV/0!
Q5TA50	Ceramide-1-phosphate transfer protein O	CPTP	24.365	6.747	5.1	1	1	1									#DIV/0!	#DIV/0!	#DIV/0!
Q9H0B8	Cysteine-rich secretory protein LCCL do	CRISPLD2	55.919	6.5273	2.2	1	1	1									#DIV/0!	#DIV/0!	#DIV/0!
Q9UKG9	Peroxisomal carnitine O-octanoyltransfer	CROT	70.178	6.2382	2.8	1	1	1									#DIV/0!	#DIV/0!	#DIV/0!
P0DP91	Chimeric ERCC6-PGBD3 protein OS=H	CSB-PGBI	119.49	6.0963	0.8	1	1	1									#DIV/0!	#DIV/0!	#DIV/0!
O95476	CTD nuclear envelope phosphatase 1 OS	CTDNEP1	28.377	6.4553	4.5	1	1	1									#DIV/0!	#DIV/0!	#DIV/0!
Q9Y5B0	RNA polymerase II subunit A C-terminal	CTDP1	104.4	17.652	2.7	3	3	3									#DIV/0!	#DIV/0!	#DIV/0!
O15194	CTD small phosphatase-like protein OS=	CTDSPL	31.128	14.252	7.6	2	2	2									#DIV/0!	#DIV/0!	#DIV/0!
Q8TB03	Uncharacterized protein CXorf38 OS=H	CXorf38	36.67	11.462	5.6	2	2	2									#DIV/0!	#DIV/0!	#DIV/0!
P49447	Cytochrome b561 OS=Homo sapiens OX	CYB561	27.559	6.4137	6	1	1	1									#DIV/0!	#DIV/0!	#DIV/0!
O14569	Cytochrome b561 domain-containing pro	CYB561D2	23.973	11.815	5.4	1	1	1									#DIV/0!	#DIV/0!	#DIV/0!
P13498	Cytochrome b-245 light chain OS=Homo	CYBA	21.012	7.5964	7.7	1	2	1									#DIV/0!	#DIV/0!	#DIV/0!
Q6UW02	Cytochrome P450 20A1 OS=Homo sapie	CYP20A1	52.432	33.68	12.8	5	5	5									#DIV/0!	#DIV/0!	#DIV/0!
Q99418	Cytohesin-2 OS=Homo sapiens OX=960	CYTH2	46.546	5.9779	15.5	4	4	1									#DIV/0!	#DIV/0!	#DIV/0!
Q8N465	D-2-hydroxyglutarate dehydrogenase, mit	D2HGDH	56.415	11.071	3.1	2	2	2									#DIV/0!	#DIV/0!	#DIV/0!
P53355	Death-associated protein kinase 1 OS=H	DAPK1	160.04	6.7922	0.8	1	1	1									#DIV/0!	#DIV/0!	#DIV/0!

Q8WVC6	Dephospho-CoA kinase domain-containi	DCAKD	26.55	11.459	5.6	2	2	2	#DIV/0!	#DIV/0!	#DIV/0!
Q9Y6V7	Probable ATP-dependent RNA helicase I	DDX49	54.226	12.533	4.1	2	2	2	#DIV/0!	#DIV/0!	#DIV/0!
Q9Y2R4	Probable ATP-dependent RNA helicase I	DDX52	67.497	18.391	6.5	3	3	3	#DIV/0!	#DIV/0!	#DIV/0!
Q8TEH3	DENN domain-containing protein 1A OS	DENND1A	110.58	6.2772	1	1	1	1	#DIV/0!	#DIV/0!	#DIV/0!
O76075	DNA fragmentation factor subunit beta C	DFFB	39.109	9.3744	3.3	1	1	1	#DIV/0!	#DIV/0!	#DIV/0!
Q7Z3D6	D-glutamate cyclase, mitochondrial OS=I	DGLUCY	66.436	5.964	3.1	1	1	1	#DIV/0!	#DIV/0!	#DIV/0!
Q7L7V1	Putative pre-mRNA-splicing factor ATP-	DHX32	84.418	13.377	2.6	2	2	2	#DIV/0!	#DIV/0!	#DIV/0!
Q9H5Z1	Probable ATP-dependent RNA helicase I	DHX35	78.909	27.756	6.4	4	5	4	#DIV/0!	#DIV/0!	#DIV/0!
Q8TF46	DIS3-like exonuclease 1 OS=Homo sapie	DIS3L	120.79	6.3197	0.9	1	1	1	#DIV/0!	#DIV/0!	#DIV/0!
Q92796	Disks large homolog 3 OS=Homo sapiens	DLG3	90.313	11.136	3.4	3	3	2	#DIV/0!	#DIV/0!	#DIV/0!
Q9Y2H0	Disks large-associated protein 4 OS=Hor	DLGAP4	108.01	12.832	3.2	2	3	2	#DIV/0!	#DIV/0!	#DIV/0!
P56179	Homeobox protein DLX-6 OS=Homo sap	DLX6	19.708	13.897	15.4	2	2	2	#DIV/0!	#DIV/0!	#DIV/0!
Q9Y485	DmX-like protein 1 OS=Homo sapiens C	DMXL1	337.83	6.5409	0.3	1	1	1	#DIV/0!	#DIV/0!	#DIV/0!
P51530	DNA replication ATP-dependent helicase	DNA2	120.41	8.2803	1.3	1	1	1	#DIV/0!	#DIV/0!	#DIV/0!
Q9UFH2	Dynein heavy chain 17, axonemal OS=Ho	DNAH17	511.78	10.51	0.4	2	2	2	#DIV/0!	#DIV/0!	#DIV/0!
O75190	DnaJ homolog subfamily B member 6 OS	DNAJB6	36.087	8.4227	3.4	1	1	1	#DIV/0!	#DIV/0!	#DIV/0!
Q9NVM6	DnaJ homolog subfamily C member 17 O	DNAJC17	34.687	14.954	8.2	2	2	2	#DIV/0!	#DIV/0!	#DIV/0!
Q96DA6	Mitochondrial import inner membrane tra	DNAJC19	12.498	11.548	14.7	2	2	2	#DIV/0!	#DIV/0!	#DIV/0!
O75061	Putative tyrosine-protein phosphatase au	DNAJC6	99.995	14.136	2.2	2	2	2	#DIV/0!	#DIV/0!	#DIV/0!
Q14185	Dedicator of cytokinesis protein 1 OS=H	DOCK1	215.34	31.005	2.5	5	5	5	#DIV/0!	#DIV/0!	#DIV/0!
Q5JSL3	Dedicator of cytokinesis protein 11 OS=I	DOCK11	237.67	6.7705	0.5	1	1	1	#DIV/0!	#DIV/0!	#DIV/0!
Q8NF50	Dedicator of cytokinesis protein 8 OS=H	DOCK8	238.53	19.159	2.1	4	4	3	#DIV/0!	#DIV/0!	#DIV/0!
Q9BQC3	2-(3-amino-3-carboxypropyl)histidine sy	DPH2	52.082	6.2898	2.9	1	1	1	#DIV/0!	#DIV/0!	#DIV/0!
Q86TI2	Dipeptidyl peptidase 9 OS=Homo sapien	DPP9	98.262	19.637	3.7	3	3	3	#DIV/0!	#DIV/0!	#DIV/0!
Q2PZI1	Probable C-mannosyltransferase DPY19I	DPY19L1	77.318	13.126	2.8	2	3	2	#DIV/0!	#DIV/0!	#DIV/0!
Q9BPU6	Dihydropyrimidinase-related protein 5 O	DPYSL5	61.421	10.835	2.8	2	2	2	#DIV/0!	#DIV/0!	#DIV/0!
Q03001	Dystonin OS=Homo sapiens OX=9606 C	DST	860.65	16.724	0.4	3	4	3	#DIV/0!	#DIV/0!	#DIV/0!
Q96EV8	Dysbindin OS=Homo sapiens OX=9606	DTNBP1	39.493	5.9889	2.8	1	1	1	#DIV/0!	#DIV/0!	#DIV/0!
Q6P1R4	tRNA-dihydrouridine(16/17) synthase [N	DUS1L	53.23	7.0125	2.7	1	2	1	#DIV/0!	#DIV/0!	#DIV/0!
O14641	Segment polarity protein dishevelled hon	DVL2	78.947	7.8439	3.7	2	2	1	#DIV/0!	#DIV/0!	#DIV/0!
Q15125	3-beta-hydroxysteroid-Delta(8),Delta(7)-	EBP	26.352	7.55	5.2	1	1	1	#DIV/0!	#DIV/0!	#DIV/0!
Q92611	ER degradation-enhancing alpha-mannos	EDEM1	73.767	8.3522	2.4	1	1	1	#DIV/0!	#DIV/0!	#DIV/0!
Q9BV94	ER degradation-enhancing alpha-mannos	EDEM2	64.752	8.5238	2.8	1	1	1	#DIV/0!	#DIV/0!	#DIV/0!
P98172	Ephrin-B1 OS=Homo sapiens OX=9606	EFNB1	38.006	5.9728	3.5	1	1	1	#DIV/0!	#DIV/0!	#DIV/0!
Q8N3D4	EH domain-binding protein 1-like protein	EHBP1L1	161.85	11.519	1.7	2	3	2	#DIV/0!	#DIV/0!	#DIV/0!
Q9BQI3	Eukaryotic translation initiation factor 2-	EIF2AK1	71.106	6.2363	2.2	1	1	1	#DIV/0!	#DIV/0!	#DIV/0!
Q9NRA8	Eukaryotic translation initiation factor 4I	EIF4ENIF1	108.2	6.729	1	1	1	1	#DIV/0!	#DIV/0!	#DIV/0!
Q8IZ81	ELMO domain-containing protein 2 OS=	ELMOD2	34.96	14.587	3.4	1	1	1	#DIV/0!	#DIV/0!	#DIV/0!
Q8NFI3	Cytosolic endo-beta-N-acetylglucosamini	ENGASE	83.986	11.602	2.8	2	2	2	#DIV/0!	#DIV/0!	#DIV/0!
Q07075	Glutamyl aminopeptidase OS=Homo sapi	ENPEP	109.24	11.742	3.2	2	2	2	#DIV/0!	#DIV/0!	#DIV/0!
Q5NDL2	EGF domain-specific O-linked N-acetylg	EOGT	62.01	7.0391	2.1	1	1	1	#DIV/0!	#DIV/0!	#DIV/0!
Q9HCM4	Band 4.1-like protein 5 OS=Homo sapier	EPB41L5	81.855	8.313	1.6	1	1	1	#DIV/0!	#DIV/0!	#DIV/0!
Q92889	DNA repair endonuclease XPF OS=Hom	ERCC4	104.48	14.227	3.2	2	2	2	#DIV/0!	#DIV/0!	#DIV/0!
P28715	DNA repair protein complementing XP-C	ERCC5	133.11	13.339	2.1	2	2	2	#DIV/0!	#DIV/0!	#DIV/0!
P50548	ETS domain-containing transcription fact	ERF	58.702	7.2271	2.6	1	1	1	#DIV/0!	#DIV/0!	#DIV/0!
Q969X5	Endoplasmic reticulum-Golgi intermediat	ERGIC1	32.592	26.812	12.8	4	5	4	#DIV/0!	#DIV/0!	#DIV/0!
O43414	ERI1 exoribonuclease 3 OS=Homo sapie	ERI3	37.238	10.643	3.6	1	2	1	#DIV/0!	#DIV/0!	#DIV/0!
Q9HBU6	Ethanolamine kinase 1 OS=Homo sapien	ETNK1	50.968	6.7423	3.8	1	1	1	#DIV/0!	#DIV/0!	#DIV/0!
O60447	Ecotropic viral integration site 5 protein I	EVI5	92.948	12.335	2.6	2	3	2	#DIV/0!	#DIV/0!	#DIV/0!
Q96CN4	EVI5-like protein OS=Homo sapiens OX	EVI5L	91.375	6.6796	1.9	1	1	1	#DIV/0!	#DIV/0!	#DIV/0!
Q8TAG9	Exocyst complex component 6 OS=Hom	EXOC6	93.721	11.681	1.5	1	1	1	#DIV/0!	#DIV/0!	#DIV/0!
Q13868	Exosome complex component RRP4 OS=	EXOSC2	32.789	7.8411	4.1	1	1	1	#DIV/0!	#DIV/0!	#DIV/0!
O15540	Fatty acid-binding protein, brain OS=Ho	FABP7	14.889	6.7974	6.8	1	1	1	#DIV/0!	#DIV/0!	#DIV/0!
Q9H098	Protein FAM107B OS=Homo sapiens O	FAM107B	15.558	13.956	16	2	2	2	#DIV/0!	#DIV/0!	#DIV/0!
Q9BQ89	Protein FAM110A OS=Homo sapiens O	FAM110A	31.27	6.6433	4.4	1	1	1	#DIV/0!	#DIV/0!	#DIV/0!
Q6P1L5	Protein FAM117B OS=Homo sapiens O	FAM117B	61.967	7.2916	2.9	1	1	1	#DIV/0!	#DIV/0!	#DIV/0!
Q96PV7	Protein FAM193B OS=Homo sapiens O	FAM193B	96.542	6.0921	1.8	1	1	1	#DIV/0!	#DIV/0!	#DIV/0!
P98173	Protein FAM3A OS=Homo sapiens OX=	FAM3A	25.152	6.7254	5.2	1	1	1	#DIV/0!	#DIV/0!	#DIV/0!
Q5HYJ3	Protein FAM76B OS=Homo sapiens OX	FAM76B	38.708	11.228	5.6	2	2	2	#DIV/0!	#DIV/0!	#DIV/0!
Q9UBU6	Protein FAM8A1 OS=Homo sapiens OX	FAM8A1	44.123	6.9797	4.1	1	1	1	#DIV/0!	#DIV/0!	#DIV/0!
Q9NVF7	F-box only protein 28 OS=Homo sapiens	FBXO28	41.149	12.84	6.2	2	2	2	#DIV/0!	#DIV/0!	#DIV/0!
Q9UK99	F-box only protein 3 OS=Homo sapiens (	FBXO3	54.56	12.289	6.6	2	2	2	#DIV/0!	#DIV/0!	#DIV/0!
Q9UKT4	F-box only protein 5 OS=Homo sapiens (	FBXO5	50.146	6.676	2.2	1	1	1	#DIV/0!	#DIV/0!	#DIV/0!
Q9UKB1	F-box/WD repeat-containing protein 11 (	FBXW11	62.09	13.411	3.9	2	2	2	#DIV/0!	#DIV/0!	#DIV/0!
Q96M96	FYVE, RhoGEF and PH domain-contain	FGD4	86.625	6.7705	1.4	1	1	1	#DIV/0!	#DIV/0!	#DIV/0!
P22455	Fibroblast growth factor receptor 4 OS=I	FGFR4	87.953	6.2727	1.2	1	1	1	#DIV/0!	#DIV/0!	#DIV/0!
Q9C0D6	FH2 domain-containing protein 1 OS=Ho	FHDC1	124.76	6.4235	1.2	1	1	1	#DIV/0!	#DIV/0!	#DIV/0!
Q9UIM3	FK506-binding protein-like OS=Homo sa	FKBPL	38.176	7.1028	3.7	1	1	1	#DIV/0!	#DIV/0!	#DIV/0!
Q14315	Filamin-C OS=Homo sapiens OX=9606	FLNC	291.02	-2	3.7	9	18	1	#DIV/0!	#DIV/0!	#DIV/0!
Q96HJ9	Protein FMC1 homolog OS=Homo sapie	FMC1	12.749	15.747	17.7	2	3	2	#DIV/0!	#DIV/0!	#DIV/0!
Q9H479	Fructosamine-3-kinase OS=Homo sapien	FN3K	35.171	16.408	10	2	2	2	#DIV/0!	#DIV/0!	#DIV/0!
Q5VW36	Focadhesin OS=Homo sapiens OX=9606	FOCAD	200.07	31.868	2.7	5	6	5	#DIV/0!	#DIV/0!	#DIV/0!
P15408	Fos-related antigen 2 OS=Homo sapiens	FOSL2	35.193	10.052	3.7	1	1	1	#DIV/0!	#DIV/0!	#DIV/0!
Q01167	Forkhead box protein K2 OS=Homo sapi	FOXK2	69.061	11.9	2.4	2	2	2	#DIV/0!	#DIV/0!	#DIV/0!
P98177	Forkhead box protein O4 OS=Homo sapi	FOXO4	53.684	10.738	5.9	2	2	2	#DIV/0!	#DIV/0!	#DIV/0!
Q9BZ67	FERM domain-containing protein 8 OS=	FRMD8	51.217	15.581	6	2	2	2	#DIV/0!	#DIV/0!	#DIV/0!
Q495W5	Alpha-(1,3)-fucosyltransferase 11 OS=Ho	FUT11	55.815	7.2376	4.1	1	1	1	#DIV/0!	#DIV/0!	#DIV/0!
O15117	FYN-binding protein 1 OS=Homo sapier	FYB1	85.386	13.629	3.2	2	2	2	#DIV/0!	#DIV/0!	#DIV/0!
Q14353	Guanidinoacetate N-methyltransferase O	GAMT	26.318	15.836	12.3	2	4	2	#DIV/0!	#DIV/0!	#DIV/0!
Q9HCG7	Non-lysosomal glucosylceramidase OS=I	GBA2	104.65	13.903	4.1	2	2	2	#DIV/0!	#DIV/0!	#DIV/0!
P28676	Grancalcin OS=Homo sapiens OX=9606	GCA	24.01	13.29	8.3	2	2	2	#DIV/0!	#DIV/0!	#DIV/0!
Q9BZD3	Putative GRINL1B complex locus protein	GCOM2	41.712	12.327	5.2	1	1	1	#DIV/0!	#DIV/0!	#DIV/0!
Q9NXN4	Ganglioside-induced differentiation-asso	GDAP2	56.224	10.969	4.2	1	1	1	#DIV/0!	#DIV/0!	#DIV/0!
Q9H840	Gem-associated protein 7 OS=Homo sapi	GEMIN7	14.536	14.223	20.6	1	1	1	#DIV/0!	#DIV/0!	#DIV/0!
Q9NWZ8	Gem-associated protein 8 OS=Homo sapi	GEMIN8	28.636	13.778	9.9	2	2	2	#DIV/0!	#DIV/0!	#DIV/0!
O95749	Geranylgeranyl pyrophosphate synthase (	GGPS1	34.87	6.32	3.7	1	1	1	#DIV/0!	#DIV/0!	#DIV/0!
Q9Y2X7	ARF GTPase-activating protein GIT1 OS	GIT1	84.34	6.2795	1.6	1	1	1	#DIV/0!	#DIV/0!	#DIV/0!
O75496	Geminin OS=Homo sapiens OX=9606 G	GMNN	23.565	11.062	9.1	2	2	2	#DIV/0!	#DIV/0!	#DIV/0!
Q03113	Guanine nucleotide-binding protein subu	GNA12	44.279	19.339	9.2	4	4	3	#DIV/0!	#DIV/0!	#DIV/0!
Q9BYB4	Guanine nucleotide-binding protein subu	GNB1L	35.618	14.807	3.1	1	1	1	#DIV/0!	#DIV/0!	#DIV/0!
Q92805	Golgin subfamily A member 1 OS=Homo	GOLGA1	88.183	18.005	4.3	3	3	3	#DIV/0!	#DIV/0!	#DIV/0!
O95249	Golgi SNAP receptor complex member 1	GOSR1	28.612	6.1322	3.6	1	1	1	#DIV/0!	#DIV/0!	#DIV/0!
Q9HCL2	Glycerol-3-phosphate acyltransferase 1, n	GPAM	93.794	15.352	3.4	2	2	2	#DIV/0!	#DIV/0!	#DIV/0!
Q9HCN4	GPN-loop GTPase 1 OS=Homo sapiens (	GPN1	41.74	7.3459	2.9	1	1	1	#DIV/0!	#DIV/0!	#DIV/0!
Q9NPR9	Protein GPR108 OS=Homo sapiens OX=	GPR108	60.632	6.2562	2	1	1	1	#DIV/0!	#DIV/0!	#DIV/0!
Q13227	G protein pathway suppressor 2 OS=Hon	GPS2	36.688	6.689	2.4	1	1	1	#DIV/0!	#DIV/0!	#DIV/0!
Q8TED1	Probable glutathione peroxidase 8 OS=H	GPX8	23.881	6.4107	5.3	1	1	1	#DIV/0!	#DIV/0!	#DIV/0!
Q8IYS0	GRAM domain-containing protein 1C OS	GRAMD1C	76.034	6.3702	1.5	1	1	1	#DIV/0!	#DIV/0!	#DIV/0!
Q8IUY3	GRAM domain-containing protein 2A OS	GRAMD2A	40.249	6.0517	2.8	1	1	1	#DIV/0!	#DIV/0!	#DIV/0!
Q6ISB3	Grainyhead-like protein 2 homolog OS=I	GRHL2	71.104	19.813	5.9	3	3	3	#DIV/0!	#DIV/0!	#DIV/0!
Q9Y3R0	Glutamate receptor-interacting protein 1 (	GRIP1	122.42	11.448	1.6	2	2	2	#DIV/0!	#DIV/0!	#DIV/0!
Q8NEC7	Glutathione S-transferase C-terminal don	GSTCD	71.078	12.116	4.3	2	2	2	#DIV/0!	#DIV/0!	#DIV/0!
P21266	Glutathione S-transferase Mu 3 OS=Hon	GSTM3	26.559	26.623	13.8	3	3	3	#DIV/0!	#DIV/0!	#DIV/0!
Q8N3Z3	GTP-binding protein 8 OS=Homo sapien	GTPBP8	32.146	6.4014	3.9	1	1	1	#DIV/0!	#DIV/0!	#DIV/0!
Q9NVX0	HAUS augmin-like complex subunit 2 O	HAUS2	26.933	6.4722	5.5	1	1	1	#DIV/0!	#DIV/0!	#DIV/0!
Q969S8	Histone deacetylase 10 OS=Homo sapien	HDAC10	71.444	6.4455	3	1	1	1	#DIV/0!	#DIV/0!	#DIV/0!
O15379	Histone deacetylase 3 OS=Homo sapiens	HDAC3	48.847	8.2416	3.5	1	1	1	#DIV/0!	#DIV/0!	#DIV/0!
Q9BYK8	Helicase with zinc finger domain 2 OS=I	HELZ2	294.65	16.238	1.1	2	2	2	#DIV/0!	#DIV/0!	#DIV/0!
Q9BTY7	Protein HGH1 homolog OS=Homo sapier	HGH1	42.129	7.0189	3.1	1	1	1	#DIV/0!	#DIV/0!	#DIV/0!
Q9NQE9	Histidine triad nucleotide-binding protein	HINT3	20.361	8.1869	8.2	1	1	1	#DIV/0!	#DIV/0!	#DIV/0!
P10321	HLA class I histocompatibility antigen, C	HLA-C	40.648	7.4048	21.3	6	9	0	#DIV/0!	#DIV/0!	#DIV/0!
Q07000	HLA class I histocompatibility antigen, C	HLA-C	40.862	6.8387	28.1	7	9	0	#DIV/0!	#DIV/0!	#DIV/0!
Q9P0W2	SWI/SNF-related matrix-associated actin	HMG20B	35.812	11.923	6.3	2	2	2	#DIV/0!	#DIV/0!	#DIV/0!
P48723	Heat shock 70 kDa protein 13 OS=Homo	HSPA13	51.927	13.376	6.4	2	3	2	#DIV/0!	#DIV/0!	#DIV/0!
Q8NDH6	Islet cell autoantigen 1-like protein OS=I	ICA1L	54.406	6.2607	2.5	1	1	1	#DIV/0!	#DIV/0!	#DIV/0!
P05362	Intercellular adhesion molecule 1 OS=Ho	ICAM1	57.825	14.93	4.9	2	3	2	#DIV/0!	#DIV/0!	#DIV/0!
P41134	DNA-binding protein inhibitor ID-1 OS=	ID1	16.132	6.48	6.5	1	1	1	#DIV/0!	#DIV/0!	#DIV/0!
Q8IY31	Intraflagellar transport protein 20 homolo	IFT20	15.28	7.2827	10.6	1	1	1	#DIV/0!	#DIV/0!	#DIV/0!
Q9BW83	Intraflagellar transport protein 27 homolo	IFT27	20.48	8.0586	7.5	1	1	1	#DIV/0!	#DIV/0!	#DIV/0!
Q96F46	Interleukin-17 receptor A OS=Homo sapi	IL17RA	96.121	6.2338	1.3	1	1	1	#DIV/0!	#DIV/0!	#DIV/0!
Q9NPH3	Interleukin-1 receptor accessory protein (	IL1RAP	65.418	13.585	3.5	2	2	2	#DIV/0!	#DIV/0!	#DIV/0!
Q9NXR8	Inhibitor of growth protein 3 OS=Homo s	ING3	46.742	6.0574	2.2	1	1	1	#DIV/0!	#DIV/0!	#DIV/0!
Q9ULG1	Chromatin-remodeling ATPase INO80 O	INO80	176.75	11.598	1.3	2	2	2	#DIV/0!	#DIV/0!	#DIV/0!
Q9UL03	Integrator complex subunit 6 OS=Homo s	INTS6	100.39	10.991	1.4	1	1	1	#DIV/0!	#DIV/0!	#DIV/0!
Q9UI26	Importin-11 OS=Homo sapiens OX=9606	IPO11	112.53	53.402	7.6	6	6	6	#DIV/0!	#DIV/0!	#DIV/0!
O94829	Importin-13 OS=Homo sapiens OX=9606	IPO13	108.19	9.5514	1.5	1	1	1	#DIV/0!	#DIV/0!	#DIV/0!
Q9BZI1	Iroquois-class homeodomain protein IRX	IRX2	49.129	6.4536	2.3	1	1	1	#DIV/0!	#DIV/0!	#DIV/0!
Q9H9L3	Interferon-stimulated 20 kDa exonuclease	ISG20L2	39.153	6.5125	3.4	1	1	1	#DIV/0!	#DIV/0!	#DIV/0!
P18084	Integrin beta-5 OS=Homo sapiens OX=9	ITGB5	88.053	6.7763	1	1	1	1	#DIV/0!	#DIV/0!	#DIV/0!
Q9Y287	Integral membrane protein 2B OS=Homo	ITM2B	30.338	8.1772	3.8	1	1	1	#DIV/0!	#DIV/0!	#DIV/0!
Q8IWB1	Inositol 1,4,5-trisphosphate receptor-inte	ITPRIP	62.059	7.8058	2.2	1	1	1	#DIV/0!	#DIV/0!	#DIV/0!
Q3MIP1	Inositol 1,4,5-trisphosphate receptor-inte	ITPRIPL2	58.446	13.088	3	1	1	1	#DIV/0!	#DIV/0!	#DIV/0!
Q9NQC1	E3 ubiquitin-protein ligase Jade-2 OS=H	JADE2	87.465	7.4839	1.4	1	1	1	#DIV/0!	#DIV/0!	#DIV/0!
Q14678	KN motif and ankyrin repeat domain-con	KANK1	147.29	11.882	1.5	2	2	2	#DIV/0!	#DIV/0!	#DIV/0!
Q63ZY3	KN motif and ankyrin repeat domain-con	KANK2	91.173	6.1574	0.9	1	2	1	#DIV/0!	#DIV/0!	#DIV/0!
O15554	Intermediate conductance calcium-activat	KCNN4	47.695	6.581	2.6	1	1	1	#DIV/0!	#DIV/0!	#DIV/0!
Q8NC54	Keratinocyte-associated transmembrane p	KCT2	29.234	9.3518	3.8	1	1	1	#DIV/0!	#DIV/0!	#DIV/0!
Q9H3F6	BTB/POZ domain-containing adapter for	KCTD10	35.432	11.949	6.4	2	2	2	#DIV/0!	#DIV/0!	#DIV/0!
Q8N371	JmjC domain-containing protein 5 OS=H	KDM8	47.27	6.0517	2.4	1	1	1	#DIV/0!	#DIV/0!	#DIV/0!
Q5JSQ8	Putative KHDC1-like protein OS=Homo	KHDC1L	14.546	7.2726	7.8	1	1	1	#DIV/0!	#DIV/0!	#DIV/0!
Q7Z7F0	KH homology domain-containing protein	KHDC4	64.845	6.6904	1.6	1	1	1	#DIV/0!	#DIV/0!	#DIV/0!
Q8IZA0	Dyslexia-associated protein KIAA0319-li	KIAA0319I	115.66	39.485	2.5	2	2	2	#DIV/0!	#DIV/0!	#DIV/0!
Q9P260	LisH domain and HEAT repeat-containin	KIAA1468	134.63	12.324	2.1	2	2	2	#DIV/0!	#DIV/0!	#DIV/0!
Q9ULH0	Kinase D-interacting substrate of 220 kD	KIDINS220	196.54	12.682	1.5	2	2	2	#DIV/0!	#DIV/0!	#DIV/0!
Q2VIQ3	Chromosome-associated kinesin KIF4B (	KIF4B	140.03	-2	8.8	10	12	1	#DIV/0!	#DIV/0!	#DIV/0!
Q8TBB5	Kelch domain-containing protein 4 OS=I	KLHDC4	57.891	7.2805	1.9	1	1	1	#DIV/0!	#DIV/0!	#DIV/0!
Q03164	Histone-lysine N-methyltransferase 2A O	KMT2A	431.76	18.236	1	3	3	3	#DIV/0!	#DIV/0!	#DIV/0!
Q9Y448	Small kinetochore-associated protein OS	KNSTRN	35.438	12.447	7.3	2	2	2	#DIV/0!	#DIV/0!	#DIV/0!
Q96JM7	Lethal(3)malignant brain tumor-like prote	L3MBTL3	88.336	11.981	3.1	2	2	2	#DIV/0!	#DIV/0!	#DIV/0!
Q9Y2U8	Inner nuclear membrane protein Man1 OS	LEMD3	99.996	6.1675	1	1	1	1	#DIV/0!	#DIV/0!	#DIV/0!
Q3ZCW2	Galectin-related protein OS=Homo sapier	LGALSL	18.986	14.846	12.2	2	3	2	#DIV/0!	#DIV/0!	#DIV/0!
Q15334	Lethal(2) giant larvae protein homolog 1 (	LLGL1	115.42	20.144	3.8	3	3	3	#DIV/0!	#DIV/0!	#DIV/0!
Q86WA8	Lon protease homolog 2, peroxisomal OS	LONP2	94.615	8.0004	1.6	1	1	1	#DIV/0!	#DIV/0!	#DIV/0!
Q643R3	Lysophospholipid acyltransferase LPCAT	LPCAT4	57.218	7.259	2.3	1	1	1	#DIV/0!	#DIV/0!	#DIV/0!
Q9Y2L9	Leucine-rich repeat and calponin homolo	LRCH1	80.874	22.503	5.5	3	4	2	#DIV/0!	#DIV/0!	#DIV/0!
Q9Y546	Leucine-rich repeat-containing protein 42	LRRC42	48.571	10.6	5.1	2	2	2	#DIV/0!	#DIV/0!	#DIV/0!

Q9Y608	Leucine-rich repeat flightless-interacting	LRRFIP2	82.17	6.3019	3.5	2	4	1	#DIV/0!	#DIV/0!	#DIV/0!
Q9BS40	Latexin OS=Homo sapiens OX=9606 GN	LXN	25.75	6.4156	4.1	1	1	1	#DIV/0!	#DIV/0!	#DIV/0!
Q9HD34	LYR motif-containing protein 4 OS=Hon	LYRM4	10.758	8.0468	15.4	1	1	1	#DIV/0!	#DIV/0!	#DIV/0!
Q7Z3D4	LysM and putative peptidoglycan-binding	LYSMD3	34.537	6.4728	2.9	1	1	1	#DIV/0!	#DIV/0!	#DIV/0!
Q8N653	Leucine-zipper-like transcriptional regula	LZTR1	94.718	6.1288	1	1	1	1	#DIV/0!	#DIV/0!	#DIV/0!
Q9BRK4	Leucine zipper putative tumor suppressor	LZTS2	72.759	22.558	5.8	3	3	3	#DIV/0!	#DIV/0!	#DIV/0!
Q9BQ69	O-acetyl-ADP-ribose deacetylase MACR	MACROD	35.505	7.4286	3.4	1	1	1	#DIV/0!	#DIV/0!	#DIV/0!
O15525	Transcription factor MafG OS=Homo sap	MAFG	17.849	11.951	9.9	2	2	2	#DIV/0!	#DIV/0!	#DIV/0!
Q969L2	Protein MAL2 OS=Homo sapiens OX=9	MAL2	19.125	8.1634	6.2	1	1	1	#DIV/0!	#DIV/0!	#DIV/0!
Q9Y2E5	Epididymis-specific alpha-mannosidase (	MAN2B2	113.98	7.3141	1	1	1	1	#DIV/0!	#DIV/0!	#DIV/0!
O00462	Beta-mannosidase OS=Homo sapiens OX	MANBA	100.89	-2	1	1	1	1	#DIV/0!	#DIV/0!	#DIV/0!
Q9NQG1	Protein MANBAL OS=Homo sapiens OX	MANBAL	9.467	6.1015	9.4	1	1	1	#DIV/0!	#DIV/0!	#DIV/0!
Q8TD08	Mitogen-activated protein kinase 15 OS=	MAPK15	59.831	6.617	1.5	1	2	1	#DIV/0!	#DIV/0!	#DIV/0!
Q13387	C-Jun-amino-terminal kinase-interacting	MAPK8IP2	87.974	6.6759	1	1	1	1	#DIV/0!	#DIV/0!	#DIV/0!
P45984	Mitogen-activated protein kinase 9 OS=H	MAPK9	48.139	12.647	5	2	2	2	#DIV/0!	#DIV/0!	#DIV/0!
Q15555	Microtubule-associated protein RP/EB fa	MAPRE2	37.031	6.1033	2.4	1	1	1	#DIV/0!	#DIV/0!	#DIV/0!
Q9NX47	E3 ubiquitin-protein ligase MARCH5 OS	MARCH5	31.231	12.441	10.4	2	2	2	#DIV/0!	#DIV/0!	#DIV/0!
Q9P0L2	Serine/threonine-protein kinase MARK1	MARK1	89.002	7.5492	6.3	4	4	1	#DIV/0!	#DIV/0!	#DIV/0!
Q14703	Membrane-bound transcription factor site	MBTPS1	117.75	6.0821	1.2	1	1	1	#DIV/0!	#DIV/0!	#DIV/0!
Q8IVS2	Malonyl-CoA-acyl carrier protein transac	MCAT	42.961	6.9049	2.8	1	1	1	#DIV/0!	#DIV/0!	#DIV/0!
Q96RQ3	Methylcrotonoyl-CoA carboxylase subun	MCCC1	80.472	14.989	4	2	2	2	#DIV/0!	#DIV/0!	#DIV/0!
Q7L590	Protein MCM10 homolog OS=Homo sap	MCM10	98.182	6.7389	1.3	1	1	1	#DIV/0!	#DIV/0!	#DIV/0!
Q96EZ8	Microspherule protein 1 OS=Homo sapie	MCRS1	51.803	5.9846	3.2	1	1	1	#DIV/0!	#DIV/0!	#DIV/0!
Q00987	E3 ubiquitin-protein ligase Mdm2 OS=H	MDM2	55.232	7.3457	2	1	1	1	#DIV/0!	#DIV/0!	#DIV/0!
Q9BTT4	Mediator of RNA polymerase II transcrip	MED10	15.688	20.387	20	3	4	3	#DIV/0!	#DIV/0!	#DIV/0!
Q13503	Mediator of RNA polymerase II transcrip	MED21	15.564	6.1587	7.6	1	1	1	#DIV/0!	#DIV/0!	#DIV/0!
O95402	Mediator of RNA polymerase II transcrip	MED26	65.446	6.4567	2.2	1	1	1	#DIV/0!	#DIV/0!	#DIV/0!
Q14680	Maternal embryonic leucine zipper kinas	MELK	74.641	19.865	5.7	3	3	3	#DIV/0!	#DIV/0!	#DIV/0!
Q9H1A3	Methyltransferase-like protein 9 OS=Hor	METTL9	36.536	6.3311	4.1	1	2	1	#DIV/0!	#DIV/0!	#DIV/0!
A1L020	RNA-binding protein MEX3A OS=Hom	MEX3A	54.173	6.7921	1.7	1	1	1	#DIV/0!	#DIV/0!	#DIV/0!
P26572	Alpha-1,3-mannosyl-glycoprotein 2-beta-	MGAT1	50.878	12.192	5.4	2	3	2	#DIV/0!	#DIV/0!	#DIV/0!
Q9UQ53	Alpha-1,3-mannosyl-glycoprotein 4-beta-	MGAT4B	63.198	7.0304	3.3	1	1	1	#DIV/0!	#DIV/0!	#DIV/0!
Q09328	Alpha-1,6-mannosylglycoprotein 6-beta-l	MGAT5	84.542	13.627	3.9	2	2	2	#DIV/0!	#DIV/0!	#DIV/0!
Q99735	Microsomal glutathione S-transferase 2 (	MGST2	16.62	8.6916	9.5	1	1	1	#DIV/0!	#DIV/0!	#DIV/0!
O15344	E3 ubiquitin-protein ligase Midline-1 OS	MID1	75.25	6.179	1.6	1	1	1	#DIV/0!	#DIV/0!	#DIV/0!
Q9BRT3	Migration and invasion enhancer 1 OS=H	MIEN1	12.403	6.0151	7.8	1	1	1	#DIV/0!	#DIV/0!	#DIV/0!
Q9H081	Protein MIS12 homolog OS=Homo sapie	MIS12	24.14	12.927	10.7	2	2	2	#DIV/0!	#DIV/0!	#DIV/0!
Q96FF7	Uncharacterized protein MISP3 OS=Hon	MISP3	24.028	7.3343	8.7	1	1	1	#DIV/0!	#DIV/0!	#DIV/0!
Q8WV92	MIT domain-containing protein 1 OS=Hc	MITD1	29.314	6.4961	4	1	1	1	#DIV/0!	#DIV/0!	#DIV/0!
Q9BUB5	MAP kinase-interacting serine/threonine-	MKNK1	51.342	14.893	5.6	2	2	2	#DIV/0!	#DIV/0!	#DIV/0!
Q9BVC4	Target of rapamycin complex subunit LS	MLST8	35.876	8.3499	5.5	1	1	1	#DIV/0!	#DIV/0!	#DIV/0!
Q9Y4U1	Methylmalonic aciduria and homocystinu	MMACHC	31.728	9.8733	5.7	1	1	1	#DIV/0!	#DIV/0!	#DIV/0!
O95396	Adenylyltransferase and sulfurtransferase	MOCS3	49.669	19.103	7	3	3	3	#DIV/0!	#DIV/0!	#DIV/0!
Q9UJG1	Motile sperm domain-containing protein	MOSPD1	24.086	7.7635	5.2	1	1	1	#DIV/0!	#DIV/0!	#DIV/0!
O75352	Mannose-P-dolichol utilization defect 1	MPDU1	26.638	6.33	4	1	2	1	#DIV/0!	#DIV/0!	#DIV/0!
Q99547	M-phase phosphoprotein 6 OS=Homo sa	MPHOSPH	19.024	6.2214	6.9	1	1	1	#DIV/0!	#DIV/0!	#DIV/0!
Q8N3R9	MAGUK p55 subfamily member 5 OS=H	MPP5	77.293	23.827	5.6	3	3	3	#DIV/0!	#DIV/0!	#DIV/0!
Q6IN84	rRNA methyltransferase 1, mitochondrial	MRM1	38.638	17.352	5.4	2	2	2	#DIV/0!	#DIV/0!	#DIV/0!
Q9H0U6	39S ribosomal protein L18, mitochondria	MRPL18	20.576	9.6045	13.9	2	1	1	#DIV/0!	#DIV/0!	#DIV/0!
Q9P0M9	39S ribosomal protein L27, mitochondria	MRPL27	16.073	27.624	30.4	4	4	4	#DIV/0!	#DIV/0!	#DIV/0!
Q13084	39S ribosomal protein L28, mitochondria	MRPL28	30.156	6.6124	4.7	1	1	1	#DIV/0!	#DIV/0!	#DIV/0!
Q8N5N7	39S ribosomal protein L50, mitochondria	MRPL50	18.325	33.351	34.2	4	4	4	#DIV/0!	#DIV/0!	#DIV/0!
O60783	28S ribosomal protein S14, mitochondria	MRPS14	15.139	13.591	18	2	2	2	#DIV/0!	#DIV/0!	#DIV/0!
Q9Y2Q9	28S ribosomal protein S28, mitochondria	MRPS28	20.843	6.4098	5.3	1	1	1	#DIV/0!	#DIV/0!	#DIV/0!
Q9HD23	Magnesium transporter MRS2 homolog,	MRS2	50.317	12.346	4.5	1	1	1	#DIV/0!	#DIV/0!	#DIV/0!
Q9BUK6	Protein misato homolog 1 OS=Homo sap	MSTO1	61.835	7.3848	2.1	1	1	1	#DIV/0!	#DIV/0!	#DIV/0!
Q96E29	Transcription termination factor 3, mitocl	MTERF3	47.971	11.207	6.7	2	2	2	#DIV/0!	#DIV/0!	#DIV/0!
Q7Z6M4	Transcription termination factor 4, mitocl	MTERF4	43.957	7.2261	3.1	1	1	1	#DIV/0!	#DIV/0!	#DIV/0!
Q9H4K7	Mitochondrial ribosome-associated GTPa	MTG2	43.955	12.077	6.9	2	2	2	#DIV/0!	#DIV/0!	#DIV/0!
Q13496	Myotubularin OS=Homo sapiens OX=96	MTM1	69.931	13.292	3	2	2	2	#DIV/0!	#DIV/0!	#DIV/0!
P03923	NADH-ubiquinone oxidoreductase chain	MT-ND6	18.622	7.1546	8	1	1	1	#DIV/0!	#DIV/0!	#DIV/0!
Q99707	Methionine synthase OS=Homo sapiens (	MTR	140.53	23.024	4.3	4	4	4	#DIV/0!	#DIV/0!	#DIV/0!
Q9UGC7	Peptide chain release factor 1-like, mitocl	MTRF1L	43.599	11.919	6.3	2	2	2	#DIV/0!	#DIV/0!	#DIV/0!
Q9ULD2	Microtubule-associated tumor suppressor	MTUS1	141.4	14.196	2.4	2	2	2	#DIV/0!	#DIV/0!	#DIV/0!
Q8N387	Mucin-15 OS=Homo sapiens OX=9606 (	MUC15	36.294	11.684	6.9	2	2	2	#DIV/0!	#DIV/0!	#DIV/0!
Q96S97	Myeloid-associated differentiation marke	MYADM	35.273	9.9962	7.1	1	2	1	#DIV/0!	#DIV/0!	#DIV/0!
O75592	E3 ubiquitin-protein ligase MYCBP2 OS	MYCBP2	513.63	8.3953	0.3	1	1	1	#DIV/0!	#DIV/0!	#DIV/0!
P24844	Myosin regulatory light polypeptide 9 OS	MYL9	19.827	6.7714	44.2	6	13	1	#DIV/0!	#DIV/0!	#DIV/0!
Q6NSJ0	Myogenesis-regulating glycosidase OS=H	MYORG	81.086	8.4302	1.5	1	1	1	#DIV/0!	#DIV/0!	#DIV/0!
Q9BRA0	N-alpha-acetyltransferase 38, NatC auxili	NAA38	13.514	6.2077	7.2	1	1	1	#DIV/0!	#DIV/0!	#DIV/0!
P54802	Alpha-N-acetylglucosaminidase OS=Hon	NAGLU	82.265	10.353	2.7	1	1	1	#DIV/0!	#DIV/0!	#DIV/0!
Q8TBE9	N-acylneuraminate-9-phosphatase OS=H	NANP	27.813	7.8896	5.6	1	1	1	#DIV/0!	#DIV/0!	#DIV/0!
Q8WUY8	N-acetyltransferase 14 OS=Homo sapiens	NAT14	21.65	6.0745	3.9	1	1	1	#DIV/0!	#DIV/0!	#DIV/0!
Q8N6N6	Protein NATD1 OS=Homo sapiens OX=	NATD1	13.032	7.8557	12.4	1	1	1	#DIV/0!	#DIV/0!	#DIV/0!
Q6IBW4	Condensin-2 complex subunit H2 OS=H	NCAPH2	68.226	6.2338	1.8	1	1	1	#DIV/0!	#DIV/0!	#DIV/0!
P56556	NADH dehydrogenase [ubiquinone] 1 alp	NDUFA6	15.136	23.791	18.8	3	4	3	#DIV/0!	#DIV/0!	#DIV/0!
O95182	NADH dehydrogenase [ubiquinone] 1 alp	NDUFA7	12.551	24.31	23.9	2	2	2	#DIV/0!	#DIV/0!	#DIV/0!
O43674	NADH dehydrogenase [ubiquinone] 1 be	NDUFB5	21.75	9.6183	12.7	2	1	1	#DIV/0!	#DIV/0!	#DIV/0!
Q15223	Nectin-1 OS=Homo sapiens OX=9606 G	NECTIN1	57.157	7.8849	2.3	1	1	1	#DIV/0!	#DIV/0!	#DIV/0!
Q8NHV4	Protein NEDD1 OS=Homo sapiens OX=	NEDD1	71.965	22.974	6.2	3	3	3	#DIV/0!	#DIV/0!	#DIV/0!
Q9UMX5	Neudesin OS=Homo sapiens OX=9606 (	NENF	18.856	14.867	14.5	2	2	2	#DIV/0!	#DIV/0!	#DIV/0!
Q7Z628	Neuroepithelial cell-transforming gene 1	NET1	67.739	6.8441	1.7	1	1	1	#DIV/0!	#DIV/0!	#DIV/0!
P35240	Merlin OS=Homo sapiens OX=9606 GN	NF2	69.689	6.1238	1.7	1	1	1	#DIV/0!	#DIV/0!	#DIV/0!
P23511	Nuclear transcription factor Y subunit alp	NFYA	36.876	8.0516	5.5	1	1	1	#DIV/0!	#DIV/0!	#DIV/0!
Q96IV0	Peptide-N(4)-(N-acetyl-beta-glucosaminy	NGLY1	74.389	8.5409	1.7	1	1	1	#DIV/0!	#DIV/0!	#DIV/0!
Q6T4R5	Nance-Horan syndrome protein OS=Hom	NHS	179.13	7.8434	1	1	1	1	#DIV/0!	#DIV/0!	#DIV/0!
Q9NYS0	NF-kappa-B inhibitor-interacting Ras-lik	NKIRAS1	21.643	7.0189	6.2	1	1	1	#DIV/0!	#DIV/0!	#DIV/0!
P59047	NACHT, LRR and PYD domains-contair	NLRP5	134.34	6.3846	0.8	1	1	1	#DIV/0!	#DIV/0!	#DIV/0!
Q9HAN9	Nicotinamide/nicotinic acid mononucleot	NMNAT1	31.932	13.032	6.5	2	2	2	#DIV/0!	#DIV/0!	#DIV/0!
O60551	Glycylpeptide N-tetradecanoyltransferase	NMT2	56.98	5.9925	8.8	3	4	1	#DIV/0!	#DIV/0!	#DIV/0!
O60936	Nucleolar protein 3 OS=Homo sapiens O	NOL3	22.629	35.442	23.1	3	3	3	#DIV/0!	#DIV/0!	#DIV/0!
Q8IVI9	Nostrin OS=Homo sapiens OX=9606 GN	NOSTRIN	57.659	6.4766	1.8	1	1	1	#DIV/0!	#DIV/0!	#DIV/0!
Q04721	Neurogenic locus notch homolog protein	NOTCH2	265.4	6.4154	1	2	2	1	#DIV/0!	#DIV/0!	#DIV/0!
P13056	Nuclear receptor subfamily 2 group C me	NR2C1	67.314	9.1148	1.8	1	1	1	#DIV/0!	#DIV/0!	#DIV/0!
Q86WQ0	Nuclear receptor 2C2-associated protein	NR2C2AP	15.876	14.296	17.3	1	1	1	#DIV/0!	#DIV/0!	#DIV/0!
Q9H7Z3	Protein NRDE2 homolog OS=Homo sapi	NRDE2	132.67	10.464	1.4	2	2	2	#DIV/0!	#DIV/0!	#DIV/0!
Q16656	Nuclear respiratory factor 1 OS=Homo sa	NRF1	53.54	7.814	2.4	1	1	1	#DIV/0!	#DIV/0!	#DIV/0!
Q96IY1	Kinetochore-associated protein NSL1 ho	NSL1	32.162	21.458	8.2	1	1	1	#DIV/0!	#DIV/0!	#DIV/0!
Q9NXX6	Non-structural maintenance of chromoso	NSMCE4A	44.301	8.0057	3.6	1	2	1	#DIV/0!	#DIV/0!	#DIV/0!
Q8TEA1	Putative methyltransferase NSUN6 OS=H	NSUN6	51.77	20.306	9.6	3	3	3	#DIV/0!	#DIV/0!	#DIV/0!
Q969T7	7-methylguanosine phosphate-specific 5'-	NT5C3B	34.389	15.375	9	2	2	2	#DIV/0!	#DIV/0!	#DIV/0!
Q8TB37	Iron-sulfur protein NUBPL OS=Homo sa	NUBPL	34.082	6.0842	2.8	1	1	1	#DIV/0!	#DIV/0!	#DIV/0!
Q96DE0	U8 snoRNA-decapping enzyme OS=Hom	NUDT16	21.273	14.047	13.3	3	3	2	#DIV/0!	#DIV/0!	#DIV/0!
A0A024RE	Diphosphoinositol polyphosphate phospl	NUDT4B	20.434	6.792	27.6	3	3	1	#DIV/0!	#DIV/0!	#DIV/0!
Q8WV74	Nucleoside diphosphate-linked moiety X	NUDT8	25.37	6.1601	4.7	1	1	1	#DIV/0!	#DIV/0!	#DIV/0!
Q9BZD4	Kinetochore protein Nuf2 OS=Homo sap	NUF2	54.303	6.6621	3.9	1	1	1	#DIV/0!	#DIV/0!	#DIV/0!
Q9UHK0	Nuclear fragile X mental retardation-inter	NUFIP1	56.299	16.8	4.6	2	3	2	#DIV/0!	#DIV/0!	#DIV/0!
Q96E22	Dehydrodolichyl diphosphate synthase co	NUS1	33.224	11.195	4.1	1	2	1	#DIV/0!	#DIV/0!	#DIV/0!
Q9UKK6	NTF2-related export protein 1 OS=Homo	NXT1	15.847	7.2159	11.4	1	1	1	#DIV/0!	#DIV/0!	#DIV/0!
Q01968	Inositol polyphosphate 5-phosphatase OC	OCRL	104.2	33.06	5.3	4	5	4	#DIV/0!	#DIV/0!	#DIV/0!
Q96D31	Calcium release-activated calcium channe	ORAI1	32.668	6.2095	2.7	1	1	1	#DIV/0!	#DIV/0!	#DIV/0!
Q9H1P3	Oxysterol-binding protein-related protein	OSBPL2	55.201	15.328	5.6	2	2	2	#DIV/0!	#DIV/0!	#DIV/0!
Q9H4B0	Probable tRNA N6-adenosine threonylca	OSGEPL1	45.122	6.2085	3.1	1	1	1	#DIV/0!	#DIV/0!	#DIV/0!
Q99650	Oncostatin-M-specific receptor subunit b	OSMR	110.51	6.5752	0.8	1	1	1	#DIV/0!	#DIV/0!	#DIV/0!
Q8N6M0	Deubiquitinase OTUD6B OS=Homo sap	OTUD6B	33.812	6.0359	6.5	1	1	1	#DIV/0!	#DIV/0!	#DIV/0!
P68402	Platelet-activating factor acetylhydrolase	PAFAH1B	25.569	20.659	12.7	2	1	1	#DIV/0!	#DIV/0!	#DIV/0!
Q504Q3	PAN2-PAN3 deadenylation complex cata	PAN2	135.37	10.875	2	2	2	2	#DIV/0!	#DIV/0!	#DIV/0!
Q96RD7	Pannexin-1 OS=Homo sapiens OX=960	PANX1	48.05	6.4034	4.7	1	1	1	#DIV/0!	#DIV/0!	#DIV/0!
Q8NI35	InaD-like protein OS=Homo sapiens OX	PATJ	196.37	6.8727	0.7	1	1	1	#DIV/0!	#DIV/0!	#DIV/0!
O14917	Protocadherin-17 OS=Homo sapiens OX	PCDH17	126.23	5.9851	0.8	1	1	1	#DIV/0!	#DIV/0!	#DIV/0!
Q9Y5F0	Protocadherin beta-13 OS=Homo sapiens	PCDHB13	87.551	12.738	3	2	2	2	#DIV/0!	#DIV/0!	#DIV/0!
Q9H4Z3	Phosphorylated CTD-interacting factor 1	PCIF1	80.669	13.305	3.6	2	2	2	#DIV/0!	#DIV/0!	#DIV/0!
O95613	Pericentrin OS=Homo sapiens OX=9606	PCNT	378.03	6.2724	0.3	1	1	1	#DIV/0!	#DIV/0!	#DIV/0!
Q16342	Programmed cell death protein 2 OS=Ho	PDCD2	38.592	13.953	7.6	2	2	2	#DIV/0!	#DIV/0!	#DIV/0!
Q53EL6	Programmed cell death protein 4 OS=Ho	PDCD4	51.735	16.531	5.8	2	2	2	#DIV/0!	#DIV/0!	#DIV/0!
Q9P2J9	[Pyruvate dehydrogenase [acetyl-transfer	PDP2	59.977	7.7423	2.6	1	1	1	#DIV/0!	#DIV/0!	#DIV/0!
Q86TG7	Retrotransposon-derived protein PEG10 (	PEG10	80.172	6.0793	1.1	1	1	1	#DIV/0!	#DIV/0!	#DIV/0!
Q9UBM1	Phosphatidylethanolamine N-methyltrans	PEMT	22.134	12.668	6.5	1	1	1	#DIV/0!	#DIV/0!	#DIV/0!
O15055	Period circadian protein homolog 2 OS=I	PER2	136.58	6.6759	0.7	1	1	1	#DIV/0!	#DIV/0!	#DIV/0!
P56589	Peroxisomal biogenesis factor 3 OS=Hon	PEX3	42.139	15.736	6.7	2	2	2	#DIV/0!	#DIV/0!	#DIV/0!
O15173	Membrane-associated progesterone recep	PGRMC2	23.818	-2	7.6	2	3	1	#DIV/0!	#DIV/0!	#DIV/0!
Q8IZ21	Phosphatase and actin regulator 4 OS=H	PHACTR4	78.21	7.2827	2	1	1	1	#DIV/0!	#DIV/0!	#DIV/0!
Q8IXK0	Polyhomeotic-like protein 2 OS=Homo s	PHC2	90.712	25.205	4.7	4	5	3	#DIV/0!	#DIV/0!	#DIV/0!
Q8WUB8	PHD finger protein 10 OS=Homo sapiens	PHF10	56.05	19.3	6.8	3	4	3	#DIV/0!	#DIV/0!	#DIV/0!
Q96QT6	PHD finger protein 12 OS=Homo sapiens	PHF12	109.7	7.7635	1.1	1	1	1	#DIV/0!	#DIV/0!	#DIV/0!
O94880	PHD finger protein 14 OS=Homo sapiens	PHF14	100.05	7.635	1.6	1	1	1	#DIV/0!	#DIV/0!	#DIV/0!
Q9BVI0	PHD finger protein 20 OS=Homo sapiens	PHF20	115.38	10.594	2	2	2	2	#DIV/0!	#DIV/0!	#DIV/0!
A8MW92	PHD finger protein 20-like protein 1 OS=	PHF20L1	115.01	6.0199	0.9	1	1	1	#DIV/0!	#DIV/0!	#DIV/0!
P46019	Phosphorylase b kinase regulatory subun	PHKA2	138.41	6.5115	0.7	1	1	1	#DIV/0!	#DIV/0!	#DIV/0!
Q9P1Y6	PHD and RING finger domain-containing	PHRF1	178.66	6.6194	1.6	2	2	1	#DIV/0!	#DIV/0!	#DIV/0!
Q5SRE7	Phytanoyl-CoA dioxygenase domain-con	PHYHD1	32.41	18.237	9.6	2	2	2	#DIV/0!	#DIV/0!	#DIV/0!
Q8N2W9	E3 SUMO-protein ligase PIAS4 OS=Hor	PIAS4	56.503	13.032	4.7	2	3	2	#DIV/0!	#DIV/0!	#DIV/0!
Q14442	Phosphatidylinositol N-acetylglucosamin	PIGH	21.08	6.9387	5.3	1	1	1	#DIV/0!	#DIV/0!	#DIV/0!
Q86T03	Type 1 phosphatidylinositol 4,5-bisphos	PIP4P1	29.469	6.5832	4	1	2	1	#DIV/0!	#DIV/0!	#DIV/0!
Q99755	Phosphatidylinositol 4-phosphate 5-kina	PIP5K1A	62.633	7.2309	6.9	3	3	1	#DIV/0!	#DIV/0!	#DIV/0!
A2A3N6	Putative PIP5K1A and PSMD4-like prot	PIPSL	95.047	19.751	6.8	5	5	0	#DIV/0!	#DIV/0!	#DIV/0!
O00625	Pirin OS=Homo sapiens OX=9606 GN=I	PIR	32.113	7.2762	3.4	1	2	1	#DIV/0!	#DIV/0!	#DIV/0!
Q6P5Z2	Serine/threonine-protein kinase N3 OS=I	PKN3	99.419	6.7922	1.3	1	1	1	#DIV/0!	#DIV/0!	#DIV/0!
Q9HAU0	Pleckstrin homology domain-containing f	PLEKHA5	127.46	12.963	1.5	2	2	2	#DIV/0!	#DIV/0!	#DIV/0!
O95397	Putative protein PLEKHA9 OS=Homo sa	PLEKHA8I	43.538	6.4762	2.6	1	1	1	#DIV/0!	#DIV/0!	#DIV/0!
O43157	Plexin-B1 OS=Homo sapiens OX=9606	PLXNB1	232.3	6.2375	0.4	1	1	1	#DIV/0!	#DIV/0!	#DIV/0!
Q9Y2G5	GDP-fucose protein O-fucosyltransferase	POFUT2	49.975	7.4433	2.8	1	1	1	#DIV/0!	#DIV/0!	#DIV/0!
Q8NBL1	Protein O-glucosyltransferase 1 OS=Hon	POGLUT1	46.189	11.22	4.1	2	2	2	#DIV/0!	#DIV/0!	#DIV/0!
P06746	DNA polymerase beta OS=Homo sapiens	POLB	38.177	22.215	10.4	3	3	3	#DIV/0!	#DIV/0!	#DIV/0!
P56282	DNA polymerase epsilon subunit 2 OS=I	POLE2	59.536	6.5802	2.1	1	1	1	#DIV/0!	#DIV/0!	#DIV/0!
P0DPB6	DNA-directed RNA polymerases I and III	POLR1D	15.237	22.12	29.3	3	3	3	#DIV/0!	#DIV/0!	#DIV/0!

O00411	DNA-directed RNA polymerase, mitocho	POLRMT	138.62	13.589	1.9	2	2	2	#DIV/0!	#DIV/0!	#DIV/0!
O95707	Ribonuclease P protein subunit p29 OS=	POP4	25.424	7.9102	5.5	1	1	1	#DIV/0!	#DIV/0!	#DIV/0!
O60927	E3 ubiquitin-protein ligase PPP1R11 OS	PPP1R11	13.952	8.6098	12.7	1	1	1	#DIV/0!	#DIV/0!	#DIV/0!
Q9UD71	Protein phosphatase 1 regulatory subunit	PPP1R1B	22.963	6.411	4.4	1	1	1	#DIV/0!	#DIV/0!	#DIV/0!
P16298	Serine/threonine-protein phosphatase 2B	PPP3CB	59.024	6.2223	6.7	3	2	1	#DIV/0!	#DIV/0!	#DIV/0!
Q92733	Proline-rich protein PRCC OS=Homo sa	PRCC	52.417	38.754	12.8	4	5	4	#DIV/0!	#DIV/0!	#DIV/0!
Q9Y3B1	PRELI domain containing protein 3B OS	PRELID3B	21.494	7.4057	7.2	1	1	1	#DIV/0!	#DIV/0!	#DIV/0!
Q9Y478	5'-AMP-activated protein kinase subunit	PRKAB1	30.382	6.8129	3.7	1	2	1	#DIV/0!	#DIV/0!	#DIV/0!
Q6P2P2	Protein arginine N-methyltransferase 9 O	PRMT9	94.5	17.589	3.6	3	3	3	#DIV/0!	#DIV/0!	#DIV/0!
O43272	Proline dehydrogenase 1, mitochondrial (	PRODH	68.001	6.0372	1.7	1	1	1	#DIV/0!	#DIV/0!	#DIV/0!
Q99633	Pre-mRNA-splicing factor 18 OS=Homo	PRPF18	39.859	7.2725	3.5	1	1	1	#DIV/0!	#DIV/0!	#DIV/0!
Q5VTL8	Pre-mRNA-splicing factor 38B OS=Hom	PRPF38B	64.467	18.056	4	3	3	3	#DIV/0!	#DIV/0!	#DIV/0!
P78330	Phosphoserine phosphatase OS=Homo sa	PSPH	25.007	6.4158	5.8	1	1	1	#DIV/0!	#DIV/0!	#DIV/0!
Q05209	Tyrosine-protein phosphatase non-recept	PTPN12	88.105	24.47	4.7	3	3	3	#DIV/0!	#DIV/0!	#DIV/0!
P17706	Tyrosine-protein phosphatase non-recept	PTPN2	48.473	7.5407	2.7	1	1	1	#DIV/0!	#DIV/0!	#DIV/0!
Q16825	Tyrosine-protein phosphatase non-recept	PTPN21	133.28	7.527	1.1	1	1	1	#DIV/0!	#DIV/0!	#DIV/0!
Q92729	Receptor-type tyrosine-protein phosphata	PTPRU	162.42	6.7844	0.8	1	1	1	#DIV/0!	#DIV/0!	#DIV/0!
Q3MIT2	Putative tRNA pseudouridine synthase Pu	PUS10	60.243	6.6374	1.5	1	1	1	#DIV/0!	#DIV/0!	#DIV/0!
P32322	Pyrroline-5-carboxylate reductase 1, mito	PYCR1	33.36	8.3001	9.7	2	3	1	#DIV/0!	#DIV/0!	#DIV/0!
Q8WU10	Pyridine nucleotide-disulfide oxidoreduc	PYROXD1	55.792	11.51	3.6	1	1	1	#DIV/0!	#DIV/0!	#DIV/0!
Q2KHR3	Glutamine and serine-rich protein 1 OS=]	QSER1	189.97	8.4227	0.6	1	1	1	#DIV/0!	#DIV/0!	#DIV/0!
Q9BXR0	Queuine tRNA-ribosyltransferase catalyti	QTRT1	44.047	7.0125	3.2	1	1	1	#DIV/0!	#DIV/0!	#DIV/0!
Q9H974	Queuine tRNA-ribosyltransferase access	QTRT2	46.712	6.329	2.7	1	1	1	#DIV/0!	#DIV/0!	#DIV/0!
Q15032	R3H domain-containing protein 1 OS=Ho	R3HDM1	120.69	6.9352	1.3	1	1	1	#DIV/0!	#DIV/0!	#DIV/0!
Q8WUD1	Ras-related protein Rab-2B OS=Homo sa	RAB2B	24.214	7.8557	34.3	7	14	1	#DIV/0!	#DIV/0!	#DIV/0!
P20336	Ras-related protein Rab-3A OS=Homo sa	RAB3A	24.984	8.0698	6.8	1	1	1	#DIV/0!	#DIV/0!	#DIV/0!
Q9NRW1	Ras-related protein Rab-6B OS=Homo sa	RAB6B	23.461	-2	17.8	3	7	1	#DIV/0!	#DIV/0!	#DIV/0!
Q9H5N1	Rab GTPase-binding effector protein 2 O	RABEP2	63.542	6.6877	1.6	1	1	1	#DIV/0!	#DIV/0!	#DIV/0!
Q7Z6M1	Rab9 effector protein with kelch motifs (	RABEPK	40.564	13.821	6.2	2	2	2	#DIV/0!	#DIV/0!	#DIV/0!
B7ZAP0	Rab GTPase-activating protein 1-like, iso	RABGAP1	29.038	6.3241	13.4	3	4	1	#DIV/0!	#DIV/0!	#DIV/0!
Q06609	DNA repair protein RAD51 homolog 1 C	RAD51	36.966	6.2129	3.5	1	1	1	#DIV/0!	#DIV/0!	#DIV/0!
O43502	DNA repair protein RAD51 homolog 3 C	RAD51C	42.189	14.278	8	2	2	2	#DIV/0!	#DIV/0!	#DIV/0!
Q2PPJ7	Ral GTPase-activating protein subunit al	RALGAPA	210.77	5.9764	0.5	1	1	1	#DIV/0!	#DIV/0!	#DIV/0!
Q9Y3L5	Ras-related protein Rap-2c OS=Homo sa	RAP2C	20.745	8.5256	20.2	3	4	1	#DIV/0!	#DIV/0!	#DIV/0!
Q8TEU7	Rap guanine nucleotide exchange factor (	RAPGEF6	179.42	14.517	1.4	2	2	1	#DIV/0!	#DIV/0!	#DIV/0!
Q15283	Ras GTPase-activating protein 2 OS=Hor	RASA2	96.613	7.7099	1.8	1	1	1	#DIV/0!	#DIV/0!	#DIV/0!
O75884	Putative hydrolase RBBP9 OS=Homo sa	RBBP9	21	7.316	10.8	1	1	1	#DIV/0!	#DIV/0!	#DIV/0!
P28749	Retinoblastoma-like protein 1 OS=Homo	RBL1	120.85	6.2743	0.9	1	1	1	#DIV/0!	#DIV/0!	#DIV/0!
Q9H1K0	Rabenosyn-5 OS=Homo sapiens OX=96(	RBSN	88.869	6.3403	1.3	1	1	1	#DIV/0!	#DIV/0!	#DIV/0!
Q8IZ40	REST corepressor 2 OS=Homo sapiens (	RCOR2	58.011	12.968	6.1	3	3	2	#DIV/0!	#DIV/0!	#DIV/0!
O94762	ATP-dependent DNA helicase Q5 OS=H	RECQL5	108.86	7.0872	1.2	1	1	1	#DIV/0!	#DIV/0!	#DIV/0!
Q9P2R6	Arginine-glutamic acid dipeptide repeats	RERE	172.42	6.4209	0.8	1	1	1	#DIV/0!	#DIV/0!	#DIV/0!
O15211	Ral guanine nucleotide dissociation stim	RGL2	83.548	7.5168	1.5	1	1	1	#DIV/0!	#DIV/0!	#DIV/0!
Q92546	RAB6A-GEF complex partner protein 2 (	RGP1	42.455	8.3419	2.8	1	1	1	#DIV/0!	#DIV/0!	#DIV/0!
A6NKT7	RanBP2-like and GRIP domain-containir	RGPD3	197.48	21.993	15.1	24	32	0	#DIV/0!	#DIV/0!	#DIV/0!
Q99666	RANBP2-like and GRIP domain-contain	RGPD5	198.92	-2	12.2	20	25	0	#DIV/0!	#DIV/0!	#DIV/0!
Q9Y3P4	Rhomboid domain-containing protein 3 (	RHBDD3	40.484	6.8945	3.1	1	1	1	#DIV/0!	#DIV/0!	#DIV/0!
Q8IXI2	Mitochondrial Rho GTPase 1 OS=Homo	RHOT1	70.783	8.5252	2.6	1	1	1	#DIV/0!	#DIV/0!	#DIV/0!
Q8IUC4	Rhophilin-2 OS=Homo sapiens OX=960	RHPN2	76.992	24.557	5.4	4	4	4	#DIV/0!	#DIV/0!	#DIV/0!
Q9NVN3	Synembryn-B OS=Homo sapiens OX=96	RIC8B	58.824	7.9708	5	2	2	1	#DIV/0!	#DIV/0!	#DIV/0!
Q6R327	Rapamycin-insensitive companion of mT	RICTOR	192.22	17.58	1.5	3	3	3	#DIV/0!	#DIV/0!	#DIV/0!
Q6NUQ1	RAD50-interacting protein 1 OS=Homo s	RINT1	90.631	23.553	4	2	2	2	#DIV/0!	#DIV/0!	#DIV/0!
Q9H9A7	RecQ-mediated genome instability protei	RMI1	70.144	12.422	4.8	2	2	2	#DIV/0!	#DIV/0!	#DIV/0!
Q9H871	Protein RMD5 homolog A OS=Homo sa	RMND5A	43.992	14.646	5.4	2	2	2	#DIV/0!	#DIV/0!	#DIV/0!
Q8TDP1	Ribonuclease H2 subunit C OS=Homo sa	RNASEH2	17.84	14.983	23.8	2	2	2	#DIV/0!	#DIV/0!	#DIV/0!
Q05823	2-5A-dependent ribonuclease OS=Homo	RNASEL	83.532	6.2077	1.2	1	1	1	#DIV/0!	#DIV/0!	#DIV/0!
O00584	Ribonuclease T2 OS=Homo sapiens OX=	RNASET2	29.48	7.058	5.5	1	1	1	#DIV/0!	#DIV/0!	#DIV/0!
Q9H6Y7	E3 ubiquitin-protein ligase RNF167 OS=	RNF167	38.298	19.169	8.3	2	2	2	#DIV/0!	#DIV/0!	#DIV/0!
Q9UNX3	60S ribosomal protein L26-like 1 OS=Ho	RPL26L1	17.256	6.534	24.8	5	10	1	#DIV/0!	#DIV/0!	#DIV/0!
Q9BUL9	Ribonuclease P protein subunit p25 OS=	RPP25	20.632	6.379	4.5	1	1	1	#DIV/0!	#DIV/0!	#DIV/0!
Q86WX3	Active regulator of SIRT1 OS=Homo sap	RPS19BP1	15.434	15.926	19.1	2	2	2	#DIV/0!	#DIV/0!	#DIV/0!
P22090	40S ribosomal protein S4, Y isoform 1 O	RPS4Y1	29.455	18.877	27.4	7	11	1	#DIV/0!	#DIV/0!	#DIV/0!
Q6P087	Mitochondrial mRNA pseudouridine syn	RPUSD3	38.46	20.234	12.5	3	3	3	#DIV/0!	#DIV/0!	#DIV/0!
Q9NYV6	RNA polymerase I-specific transcription	RRN3	74.106	6.0468	1.2	1	1	1	#DIV/0!	#DIV/0!	#DIV/0!
Q8WWV3	Reticulon-4-interacting protein 1, mitoch	RTN4IP1	43.589	12.585	5.3	2	2	2	#DIV/0!	#DIV/0!	#DIV/0!
P28702	Retinoic acid receptor RXR-beta OS=Ho	RXRB	56.921	10.342	8.4	4	4	1	#DIV/0!	#DIV/0!	#DIV/0!
Q9H4B6	Protein salvador homolog 1 OS=Homo sa	SAV1	44.633	12.384	7.3	2	2	2	#DIV/0!	#DIV/0!	#DIV/0!
Q9UQR0	Sex comb on midleg-like protein 2 OS=H	SCML2	77.256	11.051	4.3	2	2	2	#DIV/0!	#DIV/0!	#DIV/0!
Q96FV2	Secernin-2 OS=Homo sapiens OX=9606	SCRN2	46.596	9.0245	4.5	1	1	1	#DIV/0!	#DIV/0!	#DIV/0!
Q8IZQ5	Selenoprotein H OS=Homo sapiens OX=	SELENOH	13.453	7.0888	9.8	1	1	1	#DIV/0!	#DIV/0!	#DIV/0!
Q9NZV5	Selenoprotein N OS=Homo sapiens OX=	SELENON	65.812	9.7457	3.7	2	1	1	#DIV/0!	#DIV/0!	#DIV/0!
Q9P0U3	Sentrin-specific protease 1 OS=Homo sa	SENP1	73.48	6.4337	1.2	1	1	1	#DIV/0!	#DIV/0!	#DIV/0!
Q9HC62	Sentrin-specific protease 2 OS=Homo sa	SENP2	67.854	6.0081	1.9	1	1	1	#DIV/0!	#DIV/0!	#DIV/0!
Q9GZR1	Sentrin-specific protease 6 OS=Homo sa	SENP6	126.14	12.42	1.7	2	2	2	#DIV/0!	#DIV/0!	#DIV/0!
Q86VW0	SEC14 domain and spectrin repeat-conta	SESTD1	79.347	11.557	2.4	2	2	2	#DIV/0!	#DIV/0!	#DIV/0!
Q8WTS6	Histone-lysine N-methyltransferase SETI	SETD7	40.72	6.0814	2.7	1	1	1	#DIV/0!	#DIV/0!	#DIV/0!
Q8N474	Secreted frizzled-related protein 1 OS=H	SFRP1	35.385	8.6264	3.8	1	1	1	#DIV/0!	#DIV/0!	#DIV/0!
O95562	Vesicle transport protein SFT2B OS=Ho	SFT2D2	17.779	6.6538	6.9	1	1	1	#DIV/0!	#DIV/0!	#DIV/0!
Q96BR1	Serine/threonine-protein kinase Sgk3 OS	SGK3	57.107	6.2276	2.4	1	1	1	#DIV/0!	#DIV/0!	#DIV/0!
P29353	SHC-transforming protein 1 OS=Homo s	SHC1	62.821	15.829	5.5	2	2	2	#DIV/0!	#DIV/0!	#DIV/0!
Q13796	Protein Shroom2 OS=Homo sapiens OX=	SHROOM2	176.41	12.397	1.1	2	2	2	#DIV/0!	#DIV/0!	#DIV/0!
Q8IUQ4	E3 ubiquitin-protein ligase SIAH1 OS=H	SIAH1	31.123	7.987	5.3	1	1	1	#DIV/0!	#DIV/0!	#DIV/0!
Q9NTG7	NAD-dependent protein deacetylase sirtu	SIRT3	43.573	6.8105	2.8	1	1	1	#DIV/0!	#DIV/0!	#DIV/0!
P41440	Folate transporter 1 OS=Homo sapiens C	SLC19A1	64.868	12.98	6.8	2	2	2	#DIV/0!	#DIV/0!	#DIV/0!
P43003	Excitatory amino acid transporter 1 OS=I	SLC1A3	59.572	17.956	5.2	2	2	2	#DIV/0!	#DIV/0!	#DIV/0!
Q9UGH3	Solute carrier family 23 member 2 OS=H	SLC23A2	70.336	6.4394	2.2	1	2	1	#DIV/0!	#DIV/0!	#DIV/0!
Q96CQ1	Solute carrier family 25 member 36 OS=I	SLC25A36	34.282	11.436	5.5	2	2	2	#DIV/0!	#DIV/0!	#DIV/0!
Q9BZD2	Equilibrative nucleoside transporter 3 OS	SLC29A3	51.814	11.506	3.8	2	2	2	#DIV/0!	#DIV/0!	#DIV/0!
Q6PML9	Zinc transporter 9 OS=Homo sapiens OX	SLC30A9	63.514	6.7705	1.9	1	1	1	#DIV/0!	#DIV/0!	#DIV/0!
Q9ULF5	Zinc transporter ZIP10 OS=Homo sapien	SLC39A10	94.131	11.904	1.9	2	2	2	#DIV/0!	#DIV/0!	#DIV/0!
Q8N1S5	Zinc transporter ZIP11 OS=Homo sapien	SLC39A11	35.396	8.0753	5	1	1	1	#DIV/0!	#DIV/0!	#DIV/0!
Q8N370	Large neutral amino acids transporter sma	SLC43A2	62.746	23.205	7	4	3	3	#DIV/0!	#DIV/0!	#DIV/0!
Q96NT5	Proton-coupled folate transporter OS=Ho	SLC46A1	49.77	6.4235	3.1	1	1	1	#DIV/0!	#DIV/0!	#DIV/0!
O43246	Cationic amino acid transporter 4 OS=Ho	SLC7A4	68.267	6.1303	2.4	1	1	1	#DIV/0!	#DIV/0!	#DIV/0!
Q96CW6	Probable RNA polymerase II nuclear loca	SLC7A6OS	35.027	7.8576	4.2	1	1	1	#DIV/0!	#DIV/0!	#DIV/0!
Q14BN4	Sarcolemmal membrane-associated protei	SLMAP	95.197	19.657	2.5	2	2	2	#DIV/0!	#DIV/0!	#DIV/0!
Q9H0W8	Protein SMG9 OS=Homo sapiens OX=9	SMG9	57.65	13.778	4.6	2	2	2	#DIV/0!	#DIV/0!	#DIV/0!
O60906	Sphingomyelin phosphodiesterase 2 OS=	SMPD2	47.645	14.303	8.3	2	2	2	#DIV/0!	#DIV/0!	#DIV/0!
P53814	Smoothelin OS=Homo sapiens OX=960(	SMTN	99.058	30.227	5.9	5	5	5	#DIV/0!	#DIV/0!	#DIV/0!
Q9H7B4	Histone-lysine N-methyltransferase SMY	SMYD3	49.097	12.686	4.9	2	2	2	#DIV/0!	#DIV/0!	#DIV/0!
P37840	Alpha-synuclein OS=Homo sapiens OX=	SNCA	14.46	7.4781	9.3	1	1	1	#DIV/0!	#DIV/0!	#DIV/0!
Q8TAD8	Smad nuclear-interacting protein 1 OS=H	SNIP1	45.777	7.1584	3.3	1	1	1	#DIV/0!	#DIV/0!	#DIV/0!
Q9Y5W8	Sorting nexin-13 OS=Homo sapiens OX=	SNX13	112.19	11.964	2.3	2	2	2	#DIV/0!	#DIV/0!	#DIV/0!
Q8WV41	Sorting nexin-33 OS=Homo sapiens OX=	SNX33	65.264	6.6803	2.1	1	1	1	#DIV/0!	#DIV/0!	#DIV/0!
Q07889	Son of sevenless homolog 1 OS=Homo sa	SOS1	152.46	11.201	1.4	2	2	2	#DIV/0!	#DIV/0!	#DIV/0!
Q07617	Sperm-associated antigen 1 OS=Homo sa	SPAG1	103.64	6.9533	1.3	1	1	1	#DIV/0!	#DIV/0!	#DIV/0!
Q8NB90	Spermatogenesis-associated protein 5 OS	SPATA5	97.903	24.147	6	5	6	4	#DIV/0!	#DIV/0!	#DIV/0!
P78362	SRSF protein kinase 2 OS=Homo sapien	SRPK2	77.526	-2	3.5	2	3	1	#DIV/0!	#DIV/0!	#DIV/0!
Q15532	Protein SSXT OS=Homo sapiens OX=96	SS18	45.929	14.914	7.2	2	2	2	#DIV/0!	#DIV/0!	#DIV/0!
Q9UHA2	SS18-like protein 2 OS=Homo sapiens C	SS18L2	8.8349	8.1418	15.6	1	1	1	#DIV/0!	#DIV/0!	#DIV/0!
Q658P3	Metalloreductase STEAP3 OS=Homo sa	STEAP3	54.6	7.4547	2.5	1	1	1	#DIV/0!	#DIV/0!	#DIV/0!
O94768	Serine/threonine-protein kinase 17B OS=	STK17B	42.344	6.303	2.4	1	1	1	#DIV/0!	#DIV/0!	#DIV/0!
Q9Y2H1	Serine/threonine-protein kinase 38-like C	STK38L	54.002	11.749	6.2	3	3	2	#DIV/0!	#DIV/0!	#DIV/0!
Q12846	Syntaxin-4 OS=Homo sapiens OX=9606	STX4	34.18	22.334	12.1	3	3	3	#DIV/0!	#DIV/0!	#DIV/0!
O43463	Histone-lysine N-methyltransferase SUV	SUV39H1	47.907	6.4351	2.2	1	1	1	#DIV/0!	#DIV/0!	#DIV/0!
Q8N300	Small vasohibin-binding protein OS=Hor	SVBP	7.8078	6.398	22.7	1	1	1	#DIV/0!	#DIV/0!	#DIV/0!
O95425	Supervillin OS=Homo sapiens OX=9606	SVIL	247.74	57.652	1.9	3	4	3	#DIV/0!	#DIV/0!	#DIV/0!
Q8NHG7	Small VCP/p97-interacting protein OS=I	SVIP	8.4426	8.2628	14.3	1	1	1	#DIV/0!	#DIV/0!	#DIV/0!
Q8NF91	Nesprin-1 OS=Homo sapiens OX=9606 (	SYNE1	1011.1	6.4411	0.1	1	1	1	#DIV/0!	#DIV/0!	#DIV/0!
Q9NYJ8	TGF-beta-activated kinase 1 and MAP3K	TAB2	76.493	30.996	3.8	2	2	2	#DIV/0!	#DIV/0!	#DIV/0!
Q9BSH4	Translational activator of cytochrome c o	TACO1	32.477	11.731	6.1	2	2	2	#DIV/0!	#DIV/0!	#DIV/0!
O75528	Transcriptional adapter 3 OS=Homo sapi	TADA3	48.902	11.817	4.6	2	2	2	#DIV/0!	#DIV/0!	#DIV/0!
Q6P1X5	Transcription initiation factor TFIID subu	TAF2	136.97	6.0052	1.2	1	1	1	#DIV/0!	#DIV/0!	#DIV/0!
O75529	TAF5-like RNA polymerase II p300/CBF	TAF5L	66.155	18.186	5.9	3	3	3	#DIV/0!	#DIV/0!	#DIV/0!
Q13395	Probable methyltransferase TARBP1 OS	TARBP1	181.67	8.1248	0.7	1	1	1	#DIV/0!	#DIV/0!	#DIV/0!
Q9BXI6	TBC1 domain family member 10A OS=H	TBC1D10A	57.117	19.351	5.5	3	2	2	#DIV/0!	#DIV/0!	#DIV/0!
Q9NUY8	TBC1 domain family member 23 OS=Ho	TBC1D23	78.321	12.194	3.6	2	2	2	#DIV/0!	#DIV/0!	#DIV/0!
Q5QJ74	Tubulin-specific chaperone cofactor E-lik	TBCEL	48.194	12.022	5.7	2	2	2	#DIV/0!	#DIV/0!	#DIV/0!
O60907	F-box-like/WD repeat-containing protein	TBL1X	62.495	21.419	12.1	5	7	3	#DIV/0!	#DIV/0!	#DIV/0!
P20226	TATA-box-binding protein OS=Homo sa	TBP	37.698	6.3403	2.9	1	1	1	#DIV/0!	#DIV/0!	#DIV/0!
Q9UHQ7	Transcription elongation factor A protein	TCEAL9	12.749	7.0844	14.4	1	1	1	#DIV/0!	#DIV/0!	#DIV/0!
Q9NQB0	Transcription factor 7-like 2 OS=Homo s	TCF7L2	67.918	6.046	1.3	1	1	1	#DIV/0!	#DIV/0!	#DIV/0!
Q9H7E2	Tudor domain-containing protein 3 OS=I	TDRD3	73.184	13.276	3.4	2	2	2	#DIV/0!	#DIV/0!	#DIV/0!
Q9Y6I9	Testis-expressed protein 264 OS=Homo s	TEX264	34.188	16.56	7	2	2	2	#DIV/0!	#DIV/0!	#DIV/0!
Q8WVM0	Dimethyladenosine transferase 1, mitoch	TFB1M	39.542	7.6844	4	1	1	1	#DIV/0!	#DIV/0!	#DIV/0!
P01137	Transforming growth factor beta-1 OS=H	TGFB1	44.341	24.101	10.3	4	4	4	#DIV/0!	#DIV/0!	#DIV/0!
Q03167	Transforming growth factor beta receptor	TGFBR3	93.498	14.416	3.5	2	2	2	#DIV/0!	#DIV/0!	#DIV/0!
Q96EK4	THAP domain-containing protein 11 OS=	THAP11	34.455	8.4607	2.9	1	1	1	#DIV/0!	#DIV/0!	#DIV/0!
O43422	52 kDa repressor of the inhibitor of the p	THAP12	87.703	11.761	3	2	2	2	#DIV/0!	#DIV/0!	#DIV/0!
Q9BT49	THAP domain-containing protein 7 OS=I	THAP7	34.414	6.085	3.6	1	1	1	#DIV/0!	#DIV/0!	#DIV/0!
Q9NWX6	Probable tRNA(His) guanylyltransferase	THG1L	34.83	9.6061	5.7	1	1	1	#DIV/0!	#DIV/0!	#DIV/0!
O60830	Mitochondrial import inner membrane tra	TIMM17B	18.273	6.482	8.7	1	1	1	#DIV/0!	#DIV/0!	#DIV/0!
Q9Y5J9	Mitochondrial import inner membrane tra	TIMM8B	9.3435	11.823	22.9	2	2	2	#DIV/0!	#DIV/0!	#DIV/0!
O95049	Tight junction protein ZO-3 OS=Homo s	TJP3	101.4	22.484	4.2	4	4	4	#DIV/0!	#DIV/0!	#DIV/0!
Q96CP7	Calfacilitin OS=Homo sapiens OX=9606	TLCD1	28.548	6.4035	4	1	1	1	#DIV/0!	#DIV/0!	#DIV/0!
Q9UKI8	Serine/threonine-protein kinase tousled-l	TLK1	86.699	7.8598	3.3	2	2	1	#DIV/0!	#DIV/0!	#DIV/0!
Q86UE8	Serine/threonine-protein kinase tousled-l	TLK2	87.66	11.711	2.2	2	2	1	#DIV/0!	#DIV/0!	#DIV/0!
Q9Y2S6	Translation machinery-associated protein	TMA7	7.0662	6.6954	14.1	1	1	1	#DIV/0!	#DIV/0!	#DIV/0!
P55061	Bax inhibitor 1 OS=Homo sapiens OX=9	TMBIM6	26.537	12.744	3.8	2	2	2	#DIV/0!	#DIV/0!	#DIV/0!
Q9BVX2	Transmembrane protein 106C OS=Homo	TMEM106	27.875	6.3223	6.8	1	1	1	#DIV/0!	#DIV/0!	#DIV/0!
Q8IUX1	Complex I assembly factor TMEM126B,	TMEM126	25.943	7.8557	6.1	1	1	1	#DIV/0!	#DIV/0!	#DIV/0!
Q86UB9	Transmembrane protein 135 OS=Homo s	TMEM135	52.29	6.5743	1.7	1	1	1	#DIV/0!	#DIV/0!	#DIV/0!
Q8N511	Transmembrane protein 199 OS=Homo s	TMEM199	23.13	21.817	16.3	3	4	3	#DIV/0!	#DIV/0!	#DIV/0!
Q5SNT2	Transmembrane protein 201 OS=Homo s	TMEM201	72.235	13.263	4.7	2	2	2	#DIV/0!	#DIV/0!	#DIV/0!
Q96A57	Transmembrane protein 230 OS=Homo s	TMEM230	13.188	7.1328	9.2	1	2	1	#DIV/0!	#DIV/0!	#DIV/0!
Q9NQ34	Transmembrane protein 9B OS=Homo sa	TMEM9B	22.531	11.307	14.6	1	1	1	#DIV/0!	#DIV/0!	#DIV/0!

Q9BYE2	Transmembrane protease serine 13 OS=H	TMPRSS13	63.152	6.3116	1.7	1	1	1									#DIV/0!	#DIV/0!	#DIV/0!
Q15025	TNFAIP3-interacting protein 1 OS=Hom	TNIP1	71.863	7.9722	2.2	1	1	1									#DIV/0!	#DIV/0!	#DIV/0!
Q8NFZ5	TNFAIP3-interacting protein 2 OS=Hom	TNIP2	48.699	14.57	6.3	2	2	2									#DIV/0!	#DIV/0!	#DIV/0!
O15417	Trinucleotide repeat-containing gene 18 p	TNRC18	314.52	15.324	1.3	2	2	2									#DIV/0!	#DIV/0!	#DIV/0!
Q96HA7	Tonsoku-like protein OS=Homo sapiens	TONSL	150.93	32.081	3.4	5	5	5									#DIV/0!	#DIV/0!	#DIV/0!
O14656	Torsin-1A OS=Homo sapiens OX=9606	TOR1A	37.808	22.014	6	2	2	2									#DIV/0!	#DIV/0!	#DIV/0!
O60507	Protein-tyrosine sulfotransferase 1 OS=H	TPST1	42.188	6.9387	2.7	1	1	1									#DIV/0!	#DIV/0!	#DIV/0!
Q9Y4K3	TNF receptor-associated factor 6 OS=Ho	TRAF6	59.573	5.9889	1.9	1	1	1									#DIV/0!	#DIV/0!	#DIV/0!
Q86SZ2	Trafficking protein particle complex subu	TRAPPC6B	17.983	6.6698	5.7	1	2	1									#DIV/0!	#DIV/0!	#DIV/0!
O00635	E3 ubiquitin-protein ligase TRIM38 OS=	TRIM38	53.415	23.221	8.2	3	3	3									#DIV/0!	#DIV/0!	#DIV/0!
Q9NUP7	tRNA:m(4)X modification enzyme TRM1	TRMT13	54.246	13.177	4.6	2	2	2									#DIV/0!	#DIV/0!	#DIV/0!
O75648	Mitochondrial tRNA-specific 2-thiouridy	TRMU	47.744	13.945	4.8	2	2	2									#DIV/0!	#DIV/0!	#DIV/0!
Q9NX07	tRNA selenocysteine 1-associated protein	TRNAU1AP	32.498	20.821	8.7	3	3	3									#DIV/0!	#DIV/0!	#DIV/0!
Q8TEL6	Short transient receptor potential channel	TRPC4AP	90.851	6.2201	1.4	1	1	1									#DIV/0!	#DIV/0!	#DIV/0!
Q9UJT2	Testis-specific serine kinase substrate OS	TSKS	65.049	12.388	3.2	2	2	2									#DIV/0!	#DIV/0!	#DIV/0!
O95858	Tetraspanin-15 OS=Homo sapiens OX=9	TSPAN15	33.165	6.4392	2.7	1	2	1									#DIV/0!	#DIV/0!	#DIV/0!
O75954	Tetraspanin-9 OS=Homo sapiens OX=96	TSPAN9	26.779	9.6206	6.3	1	1	1									#DIV/0!	#DIV/0!	#DIV/0!
Q9Y5U2	Protein TSSC4 OS=Homo sapiens OX=9	TSSC4	34.325	8.4386	3.3	1	1	1									#DIV/0!	#DIV/0!	#DIV/0!
Q9UNY4	Transcription termination factor 2 OS=H	TTF2	129.59	12.281	2.2	2	2	2									#DIV/0!	#DIV/0!	#DIV/0!
Q9BTX7	Alpha-tocopherol transfer protein-like OS	TTPAL	38.515	6.9808	5	1	1	1									#DIV/0!	#DIV/0!	#DIV/0!
Q71U36	Tubulin alpha-1A chain OS=Homo sapie	TUBA1A	50.135	291.62	51.7	17	91	0									#DIV/0!	#DIV/0!	#DIV/0!
P04350	Tubulin beta-4A chain OS=Homo sapien	TUBB4A	49.585	14.44	50.9	17	81	0									#DIV/0!	#DIV/0!	#DIV/0!
Q5TAX3	Terminal uridylyltransferase 4 OS=Homo	TUT4	185.16	11.325	1.1	2	2	2									#DIV/0!	#DIV/0!	#DIV/0!
Q9NUQ3	Gamma-taxilin OS=Homo sapiens OX=9	TXLNG	60.585	20.796	5.5	3	3	3									#DIV/0!	#DIV/0!	#DIV/0!
P19971	Thymidine phosphorylase OS=Homo sap	TYMP	49.955	7.2456	3.1	1	1	1									#DIV/0!	#DIV/0!	#DIV/0!
Q6NUM6	S-adenosyl-L-methionine-dependent tRN	TYW1B	76.946	10.674	3.7	2	2	2									#DIV/0!	#DIV/0!	#DIV/0!
Q9Y2X8	Ubiquitin-conjugating enzyme E2 D4 OS	UBE2D4	16.649	7.0398	19.7	2	3	1									#DIV/0!	#DIV/0!	#DIV/0!
Q15819	Ubiquitin-conjugating enzyme E2 variant	UBE2V2	16.363	-2	33.1	4	4	1									#DIV/0!	#DIV/0!	#DIV/0!
O95164	Ubiquitin-like protein 3 OS=Homo sapie	UBL3	13.157	6.7084	7.7	1	1	1									#DIV/0!	#DIV/0!	#DIV/0!
Q9BZL1	Ubiquitin-like protein 5 OS=Homo sapie	UBL5	8.5468	12.29	27.4	2	2	2									#DIV/0!	#DIV/0!	#DIV/0!
Q96S82	Ubiquitin-like protein 7 OS=Homo sapie	UBL7	40.51	7.8017	4.7	1	1	1									#DIV/0!	#DIV/0!	#DIV/0!
Q9NZI7	Upstream-binding protein 1 OS=Homo sa	UBP1	60.49	7.9664	6.5	2	3	1									#DIV/0!	#DIV/0!	#DIV/0!
Q8WUN7	Ubiquitin domain-containing protein 2 O	UBTD2	26.189	7.6581	9.4	1	1	1									#DIV/0!	#DIV/0!	#DIV/0!
Q9NYU1	UDP-glucose:glycoprotein glucosyltransf	UGGT2	174.73	11.558	1.8	3	3	2									#DIV/0!	#DIV/0!	#DIV/0!
O14795	Protein unc-13 homolog B OS=Homo sap	UNC13B	180.68	28.601	1.9	3	4	3									#DIV/0!	#DIV/0!	#DIV/0!
P10746	Uroporphyrinogen-III synthase OS=Homo	UROS	28.627	6.0965	6	1	1	1									#DIV/0!	#DIV/0!	#DIV/0!
Q9UHP3	Ubiquitin carboxyl-terminal hydrolase 25	USP25	122.22	6.4235	1.3	1	1	1									#DIV/0!	#DIV/0!	#DIV/0!
Q86T82	Ubiquitin carboxyl-terminal hydrolase 37	USP37	110.17	7.5932	1.1	1	1	1									#DIV/0!	#DIV/0!	#DIV/0!
Q9H9J4	Ubiquitin carboxyl-terminal hydrolase 42	USP42	145.39	6.8404	1	1	1	1									#DIV/0!	#DIV/0!	#DIV/0!
Q8TAA9	Vang-like protein 1 OS=Homo sapiens O	VANGL1	59.974	15.043	3.6	2	2	2									#DIV/0!	#DIV/0!	#DIV/0!
Q14135	Transcription cofactor vestigial-like prote	VGLL4	30.947	14.238	9.7	2	2	2									#DIV/0!	#DIV/0!	#DIV/0!
Q96JG6	Syndetin OS=Homo sapiens OX=9606 G	VPS50	111.17	18.717	3.5	3	4	3									#DIV/0!	#DIV/0!	#DIV/0!
Q9UID3	Vacuolar protein sorting-associated prote	VPS51	86.041	7.5168	1.5	1	1	1									#DIV/0!	#DIV/0!	#DIV/0!
Q9H7M9	V-type immunoglobulin domain-containi	VSIR	33.908	7.8722	4.2	1	1	1									#DIV/0!	#DIV/0!	#DIV/0!
Q96AJ9	Vesicle transport through interaction wit	VTI1A	25.217	6.5305	4.1	1	1	1									#DIV/0!	#DIV/0!	#DIV/0!
Q9Y3C0	WASH complex subunit 3 OS=Homo sap	WASHC3	21.172	7.4863	7.2	1	1	1									#DIV/0!	#DIV/0!	#DIV/0!
Q5MNZ6	WD repeat domain phosphoinositide-inte	WDR45B	38.121	13.592	7.6	2	2	2									#DIV/0!	#DIV/0!	#DIV/0!
O94967	WD repeat-containing protein 47 OS=Ho	WDR47	101.95	7.4364	1	1	1	1									#DIV/0!	#DIV/0!	#DIV/0!
Q6PJI9	GATOR complex protein WDR59 OS=H	WDR59	109.79	12.311	2.6	2	2	2									#DIV/0!	#DIV/0!	#DIV/0!
O43379	WD repeat-containing protein 62 OS=Ho	WDR62	165.95	19.025	2.4	3	3	3									#DIV/0!	#DIV/0!	#DIV/0!
O96014	Protein Wnt-11 OS=Homo sapiens OX=9	WNT11	39.179	6.6473	5.4	1	1	1									#DIV/0!	#DIV/0!	#DIV/0!
Q9Y6F9	Protein Wnt-6 OS=Homo sapiens OX=96	WNT6	39.72	7.0872	3.3	1	1	1									#DIV/0!	#DIV/0!	#DIV/0!
Q6AWC2	Protein WWC2 OS=Homo sapiens OX=9	WWC2	133.89	11.253	2.1	3	3	2									#DIV/0!	#DIV/0!	#DIV/0!
Q9ULE0	Protein WWC3 OS=Homo sapiens OX=9	WWC3	122.67	6.1951	1	1	1	1									#DIV/0!	#DIV/0!	#DIV/0!
P23025	DNA repair protein complementing XP-A	XPA	31.368	8.0747	5.5	1	1	1									#DIV/0!	#DIV/0!	#DIV/0!
O43543	DNA repair protein XRCC2 OS=Homo s	XRCC2	31.956	6.654	2.9	1	1	1									#DIV/0!	#DIV/0!	#DIV/0!
Q8IZH2	5'-3' exoribonuclease 1 OS=Homo sapien	XRN1	194.1	12.922	1.3	2	2	2									#DIV/0!	#DIV/0!	#DIV/0!
Q8NBI6	Xyloside xylosyltransferase 1 OS=Homo	XXYLT1	43.806	7.5168	3.1	1	1	1									#DIV/0!	#DIV/0!	#DIV/0!
O75191	Xylulose kinase OS=Homo sapiens OX=	XYLB	58.382	18.685	5.2	3	3	3									#DIV/0!	#DIV/0!	#DIV/0!
P58557	Endoribonuclease YbeY OS=Homo sapie	YBEY	19.298	7.1095	6.6	1	1	1									#DIV/0!	#DIV/0!	#DIV/0!
Q86T24	Transcriptional regulator Kaiso OS=Hom	ZBTB33	74.484	7.4364	1.3	1	1	1									#DIV/0!	#DIV/0!	#DIV/0!
Q9NUA8	Zinc finger and BTB domain-containing p	ZBTB40	138.12	6.7647	0.7	1	1	1									#DIV/0!	#DIV/0!	#DIV/0!
O43298	Zinc finger and BTB domain-containing p	ZBTB43	52.63	6.0719	2.1	1	1	1									#DIV/0!	#DIV/0!	#DIV/0!
P0CG32	Zinc finger CCHC domain-containing pro	ZCCHC18	45.16	5.9604	4.2	1	1	1									#DIV/0!	#DIV/0!	#DIV/0!
Q9H5U6	Zinc finger CCHC domain-containing pro	ZCCHC4	59.009	11.092	3.9	2	2	2									#DIV/0!	#DIV/0!	#DIV/0!
Q8IUH5	Palmitoyltransferase ZDHHC17 OS=Hor	ZDHHC17	72.639	13.036	3.5	2	2	2									#DIV/0!	#DIV/0!	#DIV/0!
Q5W0Z9	Palmitoyltransferase ZDHHC20 OS=Hor	ZDHHC20	42.277	20.616	8.2	2	2	2									#DIV/0!	#DIV/0!	#DIV/0!
Q7Z7L7	Protein zer-1 homolog OS=Homo sapiens	ZER1	88.169	14.389	3.8	2	2	2									#DIV/0!	#DIV/0!	#DIV/0!
Q07352	mRNA decay activator protein ZFP36L1	ZFP36L1	36.313	14.082	6.5	2	2	2									#DIV/0!	#DIV/0!	#DIV/0!
Q9BQ24	Zinc finger FYVE domain-containing pro	ZFYVE21	26.505	15.779	13.2	2	3	2									#DIV/0!	#DIV/0!	#DIV/0!
Q9ULJ6	Zinc finger MIZ domain-containing prote	ZMIZ1	115.48	18.725	2.4	3	3	3									#DIV/0!	#DIV/0!	#DIV/0!
Q5VZL5	Zinc finger MYM-type protein 4 OS=Ho	ZMYM4	172.79	13.419	1.6	2	2	2									#DIV/0!	#DIV/0!	#DIV/0!
Q9UQR1	Zinc finger protein 148 OS=Homo sapien	ZNF148	88.975	6.8466	1.5	1	1	1									#DIV/0!	#DIV/0!	#DIV/0!
Q9UDV6	Zinc finger protein 212 OS=Homo sapien	ZNF212	55.447	6.2759	1.6	1	1	1									#DIV/0!	#DIV/0!	#DIV/0!
P17035	Zinc finger protein 28 OS=Homo sapiens	ZNF28	83.658	7.2585	3.8	2	3	1									#DIV/0!	#DIV/0!	#DIV/0!
Q8N184	Zinc finger protein 567 OS=Homo sapien	ZNF567	75.163	11.141	4.9	3	2	0									#DIV/0!	#DIV/0!	#DIV/0!
Q6ZN55	Zinc finger protein 574 OS=Homo sapien	ZNF574	98.899	8.4628	1.5	1	1	1									#DIV/0!	#DIV/0!	#DIV/0!
Q9P0T4	Zinc finger protein 581 OS=Homo sapien	ZNF581	22.01	7.6102	5.1	1	1	1									#DIV/0!	#DIV/0!	#DIV/0!
Q96SQ5	Zinc finger protein 587 OS=Homo sapien	ZNF587	65.621	13.839	3.8	2	2	2									#DIV/0!	#DIV/0!	#DIV/0!
Q9H582	Zinc finger protein 644 OS=Homo sapien	ZNF644	149.56	23.657	3.5	3	3	3									#DIV/0!	#DIV/0!	#DIV/0!
Q9Y5V0	Zinc finger protein 706 OS=Homo sapien	ZNF706	8.4977	12.704	30.3	2	2	2									#DIV/0!	#DIV/0!	#DIV/0!
Q9H5H4	Zinc finger protein 768 OS=Homo sapien	ZNF768	60.228	12.491	3.3	2	2	2									#DIV/0!	#DIV/0!	#DIV/0!
Q6DD87	Zinc finger protein 787 OS=Homo sapien	ZNF787	40.428	22.361	10.7	3	3	3									#DIV/0!	#DIV/0!	#DIV/0!
Q6ZN06	Zinc finger protein 813 OS=Homo sapien	ZNF813	71.72	6.3192	11.7	3	4	1									#DIV/0!	#DIV/0!	#DIV/0!
A7E2V4	Zinc finger SWIM domain-containing pro	ZSWIM8	197.3	6.5659	0.7	1	1	1									#DIV/0!	#DIV/0!	#DIV/0!
Q2QGD7	Zinc finger protein ZXDC OS=Homo sap	ZXDC	89.987	6.2364	1	1	1	1									#DIV/0!	#DIV/0!	#DIV/0!
Q13291	Signaling lymphocytic activation molecul	SLAMF1	37.231	6.0862	2.7	1	1	1	0.133	0.186	1.459	1.577	1.057	0.413	1.39	1.012	9.52	4.61	7.53
Q8WZ42	Titin OS=Homo sapiens OX=9606 GN=T	TTN	3816	12.4	0	2	3	2	0.114	0.245	1.354	1.432	1.025	0.751	1.309	1.195	7.76	4.95	6.97
Q9Y5Z9	UbiA prenyltransferase domain-containin	UBIAD1	36.831	6.5637	3	1	2	1	0.297	0.172	1.873	1.705	1.229	0.816	1.136	0.775	7.63	4.36	4.07
P57073	Transcription factor SOX-8 OS=Homo sa	SOX8	47.314	5.9588	2	1	1	1	0.297	0.18	1.917	1.717	1.085	0.596	1.355	0.846	7.62	3.52	4.61
P42262	Glutamate receptor 2 OS=Homo sapiens	GRIA2	98.82	6.7744	1	1	2	1	0.336	0.115	1.441	1.513	1.339	0.524	1.654	1.209	6.55	4.13	6.35
Q13023	A-kinase anchor protein 6 OS=Homo sap	AKAP6	256.72	6.0862	0.4	1	1	1	0.189	0.324	1.346	1.566	1.139	0.414	1.323	1.03	5.68	3.03	4.59
Q02224	Centromere-associated protein E OS=Ho	CENPE	316.41	32.994	1.8	5	5	5	0.247	0.356	1.512	1.469	1.333	0.633	1.18	0.64	4.94	3.26	3.02
Q15011	Homocysteine-responsive endoplasmic re	HERPUD1	43.719	8.0145	4.1	1	2	1	0.503		1.648	0.784	1.247		0.748		4.83	2.48	1.49
Q8IYJ2	Uncharacterized protein C10orf67, mitoc	C10orf67	63.655	6.3358	1.6	1	2	1	0.444	0.162	1.396	1.501	1.381	0.812	1.242	1.125	4.78	3.62	3.91
Q9H300	Presenilins-associated rhomboid-like pro	PARL	42.19	13.148	4.7	2	2	2	0.387	0.225	1.369	1.516	1.372	0.802	1.268	1.16	4.71	3.55	3.97
Q5T1H1	Protein eyes shut homolog OS=Homo sap	EYS	350.79	6.0331	0.3	1	1	1	0.247	0.318	1.242	1.338	1.321	0.63	1.264	1.099	4.57	3.45	4.18
Q9UK55	Protein Z-dependent protease inhibitor O	SERPINA10	50.706	-2	1.8	1	1	1	0.411	0.264	1.371	1.547	1.199	0.781	1.284	1.208	4.32	2.93	3.69
Q8IWC1	MAP7 domain-containing protein 3 OS=	MAP7D3	98.428	12.795	2.3	2	2	2	0.482	0.286	1.433	1.454	1.308	0.734	1.313	1.059	3.76	2.66	3.09
P35499	Sodium channel protein type 4 subunit al	SCN4A	208.06	17.446	1.6	3	3	3	0.468	0.295	1.456	1.25	1.368	0.579	1.628	1.194	3.55	2.55	3.70
Q2KJY2	Kinesin-like protein KIF26B OS=Homo s	KIF26B	223.88	10.854	0.9	2	2	2	0.325	0.336	0.981	1.224	0.8		1.191	0.828	3.34	1.21	3.05
Q8NCE2	Myotubularin-related protein 14 OS=Hor	MTMR14	72.203	13.204	4.9	2	2	2	0.692	0.325	2.291	1.075	1.74	0.647	0.935	0.46	3.31	2.35	1.37
Q03188	Centromere protein C OS=Homo sapiens	CENPC	106.83	11.567	2.2	2	2	2	0.376	0.48	1.811	0.822	1.742	2.017	0.453	0.365	3.08	4.39	0.96
Q9H8H3	Methyltransferase-like protein 7A OS=H	METTL7A	28.319	13.197	11.1	2	2	2	0.423	0.366	1.173	1.197	1.184	0.94	1.308	1.39	3.00	2.69	3.42
Q9UNQ0	ATP-binding cassette sub-family G memb	ABCG2	72.313	41.522	10.2	6	6	6	0.569	0.562	1.633	1.691	1.378	1.27	1.058	1.088	2.94	2.34	1.90
P43358	Melanoma-associated antigen 4 OS=Hom	MAGEA4	34.899	43.467	21.5	7	8	4	0.674	0.411	1.954	1.22	1.327	0.959	0.576	0.728	2.93	2.11	1.20
P23141	Liver carboxylesterase 1 OS=Homo sapie	CES1	62.52	45.5	12.2	6	7	6	0.512	0.308	1.168	1.187	1.626	1.234	1.083	1.129	2.87	3.49	2.70
P52566	Rho GDP-dissociation inhibitor 2 OS=H	ARHGDIB	22.988	28.987	17.9	3	4	3	0.504	0.353	1.206	1.251	1.38	0.892	1.405	1.217	2.87	2.65	3.06
P27487	Dipeptidyl peptidase 4 OS=Homo sapien	DPP4	88.278	62.511	10.1	7	8	7	0.542	0.312	1.208	1.207	1.403	0.955	1.306	1.203	2.83	2.76	2.94
Q14764	Major vault protein OS=Homo sapiens O	MVP	99.326	73.112	13.2	10	11	10	0.514	0.295	1.05	1.231	1.335	0.789	1.539	1.426	2.82	2.63	3.67
O15479	Melanoma-associated antigen B2 OS=Ho	MAGEB2	35.277	33.203	14.4	3	4	2	0.594	0.419	1.538	1.217	1.356	1.062	1.059	0.925	2.72	2.39	1.96
Q9GZU2	Paternally-expressed gene 3 protein OS=	PEG3	180.83	102.08	10.9	13	15	13	0.625	0.395	1.351	1.349	1.287	0.99	1.103	0.906	2.65	2.23	1.97
P38936	Cyclin-dependent kinase inhibitor 1 OS=	CDKN1A	18.119	13.335	13.4	2	2	2	0.678	0.667	2.064	1.478	0.961	0.676	1.031	0.329	2.63	1.22	1.01
Q96PE3	Type I inositol 3,4-bisphosphate 4-phosp	INPP4A	109.95	17.527	3.3	3	3	3	0.584	0.432	1.509	1.142	1.18	1.006	1.32	1.069	2.61	2.15	2.35
Q04828	Aldo-keto reductase family 1 member C1	AKR1C1	36.788	6.3008	2.5	1	1	1	0.621	0.333	1.201	1.277	1.479	0.877	1.254	1.059	2.60	2.47	2.42
P25025	C-X-C chemokine receptor type 2 OS=H	CXCR2	40.759	6.5027	2.8	1	1	1	0.501	0.364	1.045	1.17	1.632	1.004	1.179	1.028	2.56	3.05	2.55
P22352	Glutathione peroxidase 3 OS=Homo sapi	GPX3	25.552	11.221	5.3	1	1	1	0.604	0.341	1.127	1.231	1.366	0.813	1.429	1.248	2.50	2.31	2.83
P04637	Cellular tumor antigen p53 OS=Homo sa	TP53	43.653	11.819	4.8	2	2	2	0.663	0.521	1.555	1.315	1.264	0.742	1.133	0.961	2.42	1.69	1.77
P07203	Glutathione peroxidase 1 OS=Homo sapi	GPX1	22.088	55.694	44.3	7	8	7	0.717	0.375	1.295	1.232	1.297	0.935	1.17	1.078	2.31	2.04	2.06
Q9UBV7	Beta-1,4-galactosyltransferase 7 OS=Hor	B4GALT7	37.405	13.17	6.7	2	2	2	0.666	0.413	1.563	0.929	1.693	0.864	1.587	0.585	2.31	2.37	2.01
O60829	P antigen family member 4 OS=Homo sap	PAGE4	11.153	8.4718	12.7	1	1	1	0.655	0.584	1.495	1.359	0.908	0.768	1.249	1.007	2.30	1.35	1.82
P12429	Annexin A3 OS=Homo sapiens OX=960	ANXA3	36.375	83.476	30.7	9	10	9	0.624	0.514	1.279	1.287	1.17	1.024	1.205	1.083	2.25	1.93	2.01
P02751	Fibronectin OS=Homo sapiens OX=9606	FN1	262.62	195.46	12.6	21	28	21	0.657	0.649	1.363	1.537	0.919	0.77	0.927	1.132	2.22	1.29	1.58
P09382	Galectin-1 OS=Homo sapiens OX=9606	LGALS1	14.716	32.536	37.8	4	4	4	0.627	0.633	1.424	1.368	0.832	0.66	1.375	1.052	2.22	1.18	1.93
Q8N8N7	Prostaglandin reductase 2 OS=Homo sap	PTGR2	38.499	15.429	7.7	2	2	2	0.619	0.606	1.288	1.38	0.974	0.881	1.267	1.026	2.18	1.51	1.87
Q7Z6K5	Arpin OS=Homo sapiens OX=9606 GN=	ARPIN	24.943	7.4718	5.8	1	1	1	0.669	0.506	1.25	1.309	1.183	0.771	1.291	1.073	2.18	1.66	2.01
Q9BXI9	Complement C1q tumor necrosis factor-r	C1QTNF6	28.668	14.428	9.3	2	2	2	0.581	0.554	1.297	1.17	1.079	0.85	1.464	1.238	2.17	1.70	2.38
O43405	Cochlin OS=Homo sapiens OX=9606 GN	COCH	59.482	35.315	4.4	2	4	2	0.654	0.608	1.532	1.21	1.552	1.722	0.441	0.4	2.17	2.59	0.67
Q68DK2	Zinc finger FYVE domain-containing pro	ZFYVE26	284.57	22.808	2.1	4	4	4	0.925		0.913	1.086	1.485	1.291	0.658	0.44	2.16	3.00	1.19
Q13642	Four and a half LIM domains protein 1 O	FHL1	36.263	11.733	5.9	2	2	2	0.591	0.342	0.851	1.142	1.52	1.037	1.318	1.449	2.14	2.74	2.97
Q13158	FAS-associated death domain protein OS	FADD	23.279	18.752	8.7	1	2	1	0.574	0.606	1.46	1.055	1.84	0.849	0.786	0.507	2.13	2.28	1.10
P14317	Hematopoietic lineage cell-specific protei	HCLS1	54.013	31.039	10.3	4	6	4	0.674	0.467	1.204	1.223	1.11	0.845	1.399	1.286	2.13	1.71	2.35
Q9UDY4	DnaJ homolog subfamily B member 4 OS	DNAJB4	37.806	48.039	18.7	5	7	4	0.715	0.437	1.282	1.142	1.43	1.253	0.932	0.861	2.10	2.33	1.56
P00966	Argininosuccinate synthase OS=Homo sa	ASS1	46.53	19.928	8.5	3	4	3	0.64	0.572	1.253	1.284	1.102	0.993	1.075	1.009	2.09	1.73	1.72
P06396	Gelsolin OS=Homo sapiens OX=9606 G	GSN	85.696	96.187	19.1	10	3	2	0.602	0.585	1.156	1.274	1.054	0.768	1.385	1.235	2.05	1.53	2.21
Q13535	Serine/threonine-protein kinase ATR OS	ATR	301.36	12.05	0.8	2	2	2	0.762	0.624	1.417	1.367	1.158	0.638	1.279	0.981	2.01	1.30	1.63
Q14574	Desmocollin-3 OS=Homo sapiens OX=9	DSC3	99.968	17.524	3.3	3	3	3	0.741	0.681	1.55	1.297	1.059	1.259	0.705	0.714	2.00	1.63	1.00
Q8WV93	AFG1-like ATPase OS=Homo sapiens O	AFG1L	54.844	11.575	4.2	2	3	2	0.535	0.773	1.266	1.347	1.092	0.752	1.145	1.001	2.00	1.41	1.64
P25815	Protein S100-P OS=Homo sapiens OX=9	S100P	10.4	26.204	24.2	2	6	2	0.718	0.509	1.198	1.218	1.213	0.876	1.26	1.096	1.97	1.70	1.92
Q8N0Z6	Tetratricopeptide repeat protein 5 OS=H	TTC5	48.927	13.183	4.3	2	3	2	0.524	0.707	1.182	1.223	1.196	0.751	1.276	1.124	1.95	1.58	1.95
P35080	Profilin-2 OS=Homo sapiens OX=9606	PFN2	15.046	38.848	37.1	4	5	4	0.627	0.608	1.169	1.227	1.029	0.919	1.3	1.246	1.94	1.58	2.06
P09601	Heme oxygenase 1 OS=Homo sapiens OX	HMOX1	32.818	45.781	17	4	5	4	0.59	0.579	1.141	1.109	1.051	0.862	1.444	1.406	1.92	1.64	2.44
P16402	Histone H1.3 OS=Homo sapiens OX=96	HIST1H1D	22.35	13.097	23.1	7	15	2	0.704	0.511	1.253	1.01	1.3	0.685	1.438	1.364	1.86	1.63	2.31
Q9NRV9	Heme-binding protein 1 OS=Homo sapie	HEBP1	21.097	8.47	8.5	1	1	1	0.763	0.589	1.261	1.248	1.135	0.897	1.154	0.981	1.86	1.50	1.58
O60237	Protein phosphatase 1 regulatory subunit	PPP1R12B	110.4	11.236	3	3	3	2	0.766	0.614	1.402	1.146	1.242	0.68	1.327	0.918	1.85	1.39	1.63
Q9UN81	LINE-1 retrotransposable element ORF1	L1RE1	40.055	50.745	27.2	8	8	7	0.741	0.517	1.173	1.139	1.319	1.157	0.995	1.03	1.84	1.97	1.61
Q16649	Nuclear factor interleukin-3-regulated pro	NFIL3	51.471	20.448	8.9	3	3	3	0.473	0.513	1.357	1.405	1.442	1.304	1.13	0.948	2.80	2.78	2.11
A6NED2	RCC1 domain-containing protein 1 OS=H	RCCD1	40.078	7.0069	5.1	1	1	1	0.819	0.443	1.125	1.134	2.777	0.554	0.585	0.585	1.79	2.64	0.93
O75461	Transcription factor E2F6 OS=Homo sap	E2F6	31.844	6.6273	5.7	1	1	1	0.593	0.615	1.071	1.087	1.221	0.984	1.155	1.113	1.79	1.83	1.88
Q8TE85	Grainyhead-like protein 3 homolog OS=H	GRHL3	70.344	13.42	4	2	2	2	0.781	0.412	1.036	1.092	1.531	0.946	1.223	1.107	1.78	2.08	1.95
Q8IU60	m7GpppN-mRNA hydrolase OS=Homo s	DCP2	48.457	20.042	8.1	3	4	3	0.561	0.862	1.237	1.271	1.033	0.607	1.421	0.997	1.76	1.15	1.70
Q8TD26	Chromodomain-helicase-DNA-binding pr	CHD6	305.41	17.868	1.9	4	4	3	0.509	0.925	1.316	1.197	1.163	0.757	1.261	0.993	1.75	1.34	1.57
Q15208	Serine/threonine-protein kinase 38 OS=H	STK38	54.19	46.991	15.7	6	6	5	0.637	0.62	1.059	1.117	1.271	0.922	1.15	1.103	1.73	1.74	1.79
P00441	Superoxide dismutase [Cu-Zn] OS=Hom	SOD1	15.936	33.275	32.5	5	8	5	0.745	0.67	1.258	1.174	1.105	0.905	1.19	1.113	1.72	1.42	1.63
Q01740	Dimethylaniline monooxygenase [N-oxid	FMO1	60.31	5.9893	2.1	1	1	1	0.841	0.717	0.881	1.774	0.823	0.865	0.788	0.888	1.70	1.08	1.08
Q96P16	Regulation of nuclear pre-mRNA domain	RPRD1A	35.719	23.516	14.4	3	4	3	0.82	0.715	1.454	1.158	1.102	0.933	1.041	0.827	1.70	1.33	1.22

Q9BYN0	Sulfiredoxin-1 OS=Homo sapiens OX=9	SRXN1	14.259	7.2456	10.9	1	1	1	0.691	0.587	1.125	1.049	1.302	1.045	1.487	0.94	1.70	1.84	1.90
Q9Y5S1	Transient receptor potential cation chann	TRPV2	85.98	11.506	3.1	2	2	2	0.857	0.62	1.353	1.139	1.287	1.026	1.023	0.723	1.69	1.57	1.18
Q7Z333	Probable helicase senataxin OS=Homo sa	SETX	302.88	10.689	0.6	2	2	2	0.745	0.625	1.138	1.172	1.229	0.874	1.172	1.152	1.69	1.54	1.70
Q14019	Coactosin-like protein OS=Homo sapiens	COTL1	15.945	62.627	57	8	15	8	0.744	0.689	1.221	1.17	1.037	0.951	1.234	1.106	1.67	1.39	1.63
Q96HB5	Coiled-coil domain-containing protein 12	CCDC120	67.567	7.9181	3.8	1	1	1	0.88	0.415	1.241	0.912	1.832	1.328	0.766	0.804	1.66	2.44	1.21
Q04446	1,4-alpha-glucan-branching enzyme OS=	GBE1	80.473	105.81	21.4	12	14	12	0.696	0.751	1.241	1.159	1.181	1.009	1.122	1.033	1.66	1.51	1.49
B2RUZ4	Small integral membrane protein 1 OS=H	SMIM1	8.749	8.2684	20.5	1	1	1	0.734	0.752	1.257	1.205	1.09	0.757	1.285	1.007	1.66	1.24	1.54
O60613	Selenoprotein F OS=Homo sapiens OX=	SELENOF	18.092	6.9106	7.9	1	1	1	0.724	0.665	1.13	1.149	1.364	0.858	1.231	1.011	1.64	1.60	1.61
P33527	Multidrug resistance-associated protein 1	ABCC1	171.59	6.0334	0.6	1	1	1	0.817	0.745	1.242	1.305	0.932	0.882	1.914	1.332	1.63	1.16	2.08
Q01658	Protein Dr1 OS=Homo sapiens OX=960(	DR1	19.443	14.369	11.9	2	4	2	0.773	0.856	1.43	1.216	1.041	0.803	1.078	0.85	1.62	1.13	1.18
Q9UII2	ATPase inhibitor, mitochondrial OS=Hor	ATP5IF1	12.249	13.268	17	2	2	2	1.142	0.259	1.459	0.8	1.401	0.472	0.786	1.793	1.61	1.34	1.84
Q9NVN8	Guanine nucleotide-binding protein-like	GNL3L	65.572	133.37	26.3	12	16	12	0.873	0.898	1.62	1.233	0.848	0.889	0.973	0.598	1.61	0.98	0.89
Q9Y5J5	Pleckstrin homology-like domain family	PHLDA3	13.891	20.158	22	3	5	3	0.823	0.687	1.314	1.109	1.393	1.138	0.89	0.762	1.60	1.68	1.09
Q96GQ5	RUS1 family protein C16orf58 OS=Hom	C16orf58	51.017	14.141	3.8	2	3	2	0.806	0.825	1.452	1.162	0.772	1.868	0.579	0.609	1.60	1.62	0.73
Q5HYI8	Rab-like protein 3 OS=Homo sapiens OX	RABL3	26.422	26.598	14	3	5	3	0.803	0.696	1.2	1.197	1.16	0.928	1.129	0.96	1.60	1.39	1.39
Q53FA7	Quinone oxidoreductase PIG3 OS=Homo	TP53I3	35.536	17.62	5.1	2	2	2	0.752	0.757	1.293	1.119	1.126	1.317	0.863	0.919	1.60	1.62	1.18
O15198	Mothers against decapentaplegic homolo	SMAD9	52.493	7.8732	4.5	2	3	1	0.794	0.788	1.332	1.186	1.082	0.328	1.359	1.192	1.59	0.89	1.61
Q9H6S3	Epidermal growth factor receptor kinase s	EPS8L2	80.62	151.42	28.5	14	17	14	0.789	0.752	1.242	1.207	1.071	1.012	1.03	0.925	1.59	1.35	1.27
Q96PM9	Zinc finger protein 385A OS=Homo sapi	ZNF385A	40.453	30.047	10.9	3	3	3	0.744	0.675	1.122	1.12	1.134	1.394	0.93	1.036	1.58	1.78	1.39
P28482	Mitogen-activated protein kinase 1 OS=H	MAPK1	41.389	35.193	18.3	5	6	4	0.63	0.746	1.086	1.073	1.125	1.003	1.292	1.174	1.57	1.55	1.79
Q8WUH6	Transmembrane protein 263 OS=Homo s	TMEM263	11.748	15.636	27.6	2	2	2	0.664	0.91	1.406	1.063	1.127	1.325	0.856	0.91	1.57	1.56	1.12
Q96AD5	Patatin-like phospholipase domain-conta	PNPLA2	55.315	14.328	4.8	2	2	2	0.71	0.85	1.221	1.221	0.921	0.968	1.215	0.997	1.57	1.21	1.42
Q99541	Perilipin-2 OS=Homo sapiens OX=9606	PLIN2	48.075	78.083	24.9	10	13	10	0.797	0.781	1.235	1.21	0.802	0.678	1.411	1.19	1.55	0.94	1.65
Q9UGJ1	Gamma-tubulin complex component 4 OS	TUBGCP4	76.088	17.784	4	2	2	2	0.983	0.599	1.437	1.005	1.636	0.538	1.133	0.686	1.54	1.37	1.15
P04264	Keratin, type II cytoskeletal 1 OS=Homo	KRT1	66.038	135.44	23.9	18	1	1	1.23	0.633	1.937	0.933	0.996	0.926	0.602	0.648	1.54	1.03	0.67
P33897	ATP-binding cassette sub-family D memb	ABCD1	82.936	15.171	2.7	2	2	2	0.79	0.769	1.299	1.091	1.171	1.133	0.913	0.971	1.53	1.48	1.21
P50151	Guanine nucleotide-binding protein G(I)/	GNG10	7.2053	17.073	26.5	2	2	2	0.82	0.7	1.161	1.163	1.273	0.928	1.071	0.933	1.53	1.45	1.32
P05204	Non-histone chromosomal protein HMG-	HMGN2	9.3926	27.041	47.8	5	5	2	0.89	0.604	0.972	1.308	1.276	0.957	0.787	1.102	1.53	1.49	1.26
P40306	Proteasome subunit beta type-10 OS=Ho	PSMB10	28.936	15.731	11	2	2	2	0.766	0.689	1.058	1.149	1.2	1.105	1.034	1.113	1.52	1.58	1.48
O95757	Heat shock 70 kDa protein 4L OS=Homo	HSPA4L	94.511	124.43	29.3	20	30	16	0.802	0.707	1.127	1.153	1.099	1.04	1.057	1.032	1.51	1.42	1.38
P51570	Galactokinase OS=Homo sapiens OX=96	GALK1	42.272	50.378	23.2	6	7	6	0.765	0.717	1.113	1.126	1.065	0.903	1.285	1.16	1.51	1.33	1.65
Q9NZI6	Transcription factor CP2-like protein 1 C	TFCP2L1	54.626	8.3239	1.9	1	2	1	0.767	0.513	1.109	0.82	1.937	1.7	0.706	0.79	1.51	2.84	1.17
Q9UPN7	Serine/threonine-protein phosphatase 6 r	PPP6R1	96.723	38.722	4	2	2	2	0.9	0.868	1.559	1.091	0.871	1.186	0.942	0.628	1.50	1.16	0.89
P10620	Microsomal glutathione S-transferase 1 C	MGST1	17.598	13.297	9	2	2	2	0.758	0.673	0.996	1.148	1.184	1.363	0.822	1.187	1.50	1.78	1.40
A6NHX0	Cytosolic arginine sensor for mTORC1 s	CASTOR2	36.056	9.9508	3.6	1	2	1	0.867	0.775	1.317	1.14	1.215	1.231	0.789	0.695	1.50	1.49	0.90
Q4KMP7	TBC1 domain family member 10B OS=H	TBC1D10E	87.198	44.903	3.8	2	2	2	0.912	0.689	1.243	1.15	1.201	0.748	1.194	0.846	1.49	1.22	1.27
Q7LBR1	Charged multivesicular body protein 1b C	CHMP1B	22.109	10.907	8.5	2	2	1	0.848	0.724	1.214	1.133	1.203	1.112	0.971	0.847	1.49	1.47	1.16
P16989	Y-box-binding protein 3 OS=Homo sapie	YBX3	40.089	54.195	24.5	6	9	3	0.836	0.8	1.284	1.151	1.066	1.264	0.733	0.928	1.49	1.42	1.02
Q6ZRQ5	Protein MMS22-like OS=Homo sapiens	MMS22L	142.32	22.041	2.5	3	3	3	0.815	0.7	1.202	1.041	1.312	0.849	1.237	0.99	1.48	1.43	1.47
P11488	Guanine nucleotide-binding protein G(t)	GNAT1	40.04	6.1323	6	2	7	1	0.844	0.86	1.335	1.183	1.016	0.694	1.189	0.899	1.48	1.00	1.23
P31947	14-3-3 protein sigma OS=Homo sapiens	SFN	27.774	18.536	18.5	5	17	3	0.591	0.702	0.902	1.007	1.493	0.97	1.27	1.331	1.48	1.90	2.01
P00390	Glutathione reductase, mitochondrial OS	GSR	56.256	119.67	28	12	17	12	0.82	0.785	1.24	1.128	1.1	1.102	1.031	0.979	1.48	1.37	1.25
P15559	NAD(P)H dehydrogenase [quinone] 1 OS	NQO1	30.867	64.616	27	8	12	8	0.767	0.753	1.104	1.133	1.122	1.062	1.173	1.019	1.47	1.44	1.44
Q9UMN6	Histone-lysine N-methyltransferase 2B O	KMT2B	293.51	30.684	2	5	5	5	0.88	0.856	1.323	1.202	0.959	1.018	1.002	0.736	1.45	1.14	1.00
Q8WXE9	Stonin-2 OS=Homo sapiens OX=9606 G	STON2	101.16	6.145	1	1	1	1	0.943	0.631	1.186	1.097	1.287	1.071	0.968	0.818	1.45	1.50	1.13
Q16881	Thioredoxin reductase 1, cytoplasmic OS	TXNRD1	70.905	160.91	24.3	13	28	12	0.821	0.758	1.167	1.12	1.097	0.913	1.186	1.07	1.45	1.27	1.43
Q96RN5	Mediator of RNA polymerase II transcrip	MED15	86.753	54.824	4.9	3	4	3	0.806	0.684	1.072	1.085	1.259	0.751	1.553	0.899	1.45	1.35	1.65
P53985	Monocarboxylate transporter 1 OS=Hom	SLC16A1	53.944	25	5.6	3	4	3	0.905	0.715	1.262	1.078	1.17	0.995	1.062	0.862	1.44	1.34	1.19
Q9BTD8	RNA-binding protein 42 OS=Homo sapie	RBM42	50.413	54.851	11.2	3	4	3	0.9	0.74	1.22	1.142	1.168	1.161	0.899	0.806	1.44	1.42	1.04
O95833	Chloride intracellular channel protein 3 C	CLIC3	26.648	183.84	55.1	11	37	11	0.776	0.797	1.159	1.105	1.07	1.034	1.041	1.076	1.44	1.34	1.35
P58004	Sestrin-2 OS=Homo sapiens OX=9606 C	SESN2	54.493	19.89	7.7	3	3	3	0.82	0.785	1.173	1.133	1.075	0.937	1.24	0.91	1.44	1.25	1.34
Q9P2B4	CTTNBP2 N-terminal-like protein OS=H	CTTNBP2I	70.157	7.1983	1.7	1	1	1	0.868	1.019	1.527	1.182	0.892	0.81	1.064	0.634	1.44	0.90	0.90
Q9H1C7	Cysteine-rich and transmembrane domain	CYSTM1	10.631	6.6441	10.3	1	1	1	0.829	0.682	0.988	1.177	1.085	0.853	1.129	1.313	1.43	1.28	1.62
O94992	Protein HEXIM1 OS=Homo sapiens OX	HEXIM1	40.623	69.536	17	2	3	2	0.76	0.769	1.117	1.07	1.17	1.261	0.932	1.087	1.43	1.59	1.32
Q86YD1	Prostate tumor-overexpressed gene 1 prot	PTOV1	46.868	5.999	2.4	1	1	1	0.834	0.72	1.131	1.082	1.195	1.067	1.026	1.059	1.42	1.46	1.34
P62341	Thioredoxin reductase-like selenoprotein	SELENOT	22.324	12.944	12.8	2	3	2	0.833	0.73	1.133	1.088	1.167	0.85	1.236	1.065	1.42	1.29	1.47
P54753	Ephrin type-B receptor 3 OS=Homo sapi	EPHB3	110.33	18.05	7.7	6	7	2	0.78	0.838	1.245	1.044	1.054	0.876	1.408	0.928	1.41	1.19	1.44
Q9C0H2	Protein tweety homolog 3 OS=Homo sap	TTYH3	57.544	6.193	1.5	1	1	1	0.821	0.766	1.115	1.127	1.096	0.984	1.172	1.004	1.41	1.31	1.37
Q6ZW49	PAX-interacting protein 1 OS=Homo sap	PAXIP1	121.34	20.257	3.2	3	4	3	0.726	0.903	1.04	1.259	0.927	0.778	1.323	1.108	1.41	1.05	1.49
Q07812	Apoptosis regulator BAX OS=Homo sap	BAX	21.184	44.151	36.5	6	11	6	0.864	0.804	1.264	1.086	1.131	0.912	1.164	0.939	1.41	1.22	1.26
P27105	Erythrocyte band 7 integral membrane pr	STOM	31.73	25.688	13.2	3	3	3	0.781	0.803	1.112	1.119	1.189	1.24	0.9	0.981	1.41	1.53	1.19
Q9H8W4	Pleckstrin homology domain-containing f	PLEKHF2	27.797	46.277	20.1	3	3	3	0.869	0.781	1.198	1.119	1.053	0.926	1.175	0.932	1.40	1.20	1.28
Q9UBB5	Methyl-CpG-binding domain protein 2 O	MBD2	43.254	20.518	10.2	3	4	3	0.832	0.806	1.11	1.19	1.057	1.188	0.825	1.031	1.40	1.37	1.13
Q15149	Plectin OS=Homo sapiens OX=9606 GN	PLEC	531.78	323.31	36	139	189	131	0.824	0.797	1.155	1.118	1.064	0.955	1.148	1.02	1.40	1.25	1.34
Q9NXE8	Pre-mRNA-splicing factor CWC25 homo	CWC25	49.647	11.643	4.7	2	2	2	0.833	0.747	1.226	0.986	1.183	1.073	1.155	0.979	1.40	1.43	1.35
Q16134	Electron transfer flavoprotein-ubiquinone	ETFDH	68.495	23.146	6.3	3	3	3	0.82	0.859	1.276	1.069	1.182	1.255	0.822	0.847	1.40	1.45	0.99
P28290	Sperm-specific antigen 2 OS=Homo sapi	SSFA2	138.38	7.2078	1.1	1	1	1	0.792	0.72	1.07	1.04	1.03	1.392	1.034	1.107	1.40	1.60	1.42
Q9NQ88	Fructose-2,6-bisphosphatase TIGAR OS=	TIGAR	30.062	93.198	47	8	19	8	0.855	0.829	1.236	1.114	1.007	0.992	0.985	0.98	1.40	1.19	1.17
P50416	Carnitine O-palmitoyltransferase 1, liver	CPT1A	88.367	12.29	3.1	2	2	2	0.774	0.851	1.136	1.119	1.114	1.208	0.953	0.979	1.39	1.43	1.19
Q2TAM9	Tumor suppressor candidate gene 1 prote	TUSC1	23.39	13.374	14.6	2	2	2	0.841	0.834	1.112	1.212	0.995	1.231	0.782	1.01	1.39	1.33	1.07
O15297	Protein phosphatase 1D OS=Homo sapie	PPM1D	66.674	7.4792	2.3	1	2	1	0.799	0.693	1.024	1.046	1.159	1.104	1.097	1.255	1.39	1.52	1.58
Q9BWE0	Replication initiator 1 OS=Homo sapiens	REPIN1	63.574	23.967	2.8	1	1	1	0.819	0.876	1.171	1.175	0.944	0.814	1.251	1.003	1.38	1.04	1.33
Q9P0U4	CXXC-type zinc finger protein 1 OS=Ho	CXXC1	75.711	13.075	3.7	2	2	2	0.9	0.707	1.129	1.093	1.237	1.318	0.729	0.938	1.38	1.59	1.04
P36969	Phospholipid hydroperoxide glutathione	GPX4	22.174	30.494	26.4	4	5	4	0.848	0.803	1.171	1.109	1.062	0.926	1.152	0.969	1.38	1.20	1.28
Q9NS93	Transmembrane 7 superfamily member 3	TM7SF3	64.166	6.7893	1.6	1	1	1	0.814	0.845	1.13	1.16	1.037	1.026	1.103	0.952	1.38	1.24	1.24
P00533	Epidermal growth factor receptor OS=Ho	EGFR	134.28	238.21	33.1	29	38	29	0.78	0.794	1.052	1.12	1.02	0.882	1.29	1.141	1.38	1.21	1.54
Q96Q06	Perilipin-4 OS=Homo sapiens OX=9606	PLIN4	134.43	19.037	5.3	3	3	3	0.712	0.946	1.259	1.022	0.99	1.309	1.012	1.011	1.38	1.39	1.22
P20338	Ras-related protein Rab-4A OS=Homo sa	RAB4A	24.389	16.861	12.8	2	2	2	0.784	0.875	1.223	1.058	1.159	1.091	1.059	0.917	1.37	1.36	1.19
Q14192	Four and a half LIM domains protein 2 O	FHL2	32.193	105.4	41.6	11	17	11	0.845	0.769	1.07	1.146	0.964	0.837	1.329	1.171	1.37	1.12	1.55
P07738	Bisphosphoglycerate mutase OS=Homo s	BPGM	30.005	26.967	7.3	1	2	1	0.891	0.724	1.103	1.11	1.051	0.896	1.229	1.041	1.37	1.21	1.41
Q7LG56	Ribonucleoside-diphosphate reductase su	RRM2B	40.736	29.1	16.2	5	7	3	0.742	0.867	1.142	1.061	0.994	1.356	1.05	0.996	1.37	1.46	1.27
P00568	Adenylate kinase isoenzyme 1 OS=Homo	AK1	21.635	31.819	27.3	5	6	4	0.924	0.812	1.256	1.119	0.999	0.945	1.007	0.808	1.37	1.12	1.05
Q5VVQ6	Ubiquitin thioesterase OTU1 OS=Homo	YOD1	38.322	12.75	5.5	2	2	2	0.862	0.75	1.101	1.104	1.212	1.029	1.045	0.966	1.37	1.39	1.25
Q15048	Leucine-rich repeat-containing protein 14	LRRC14	54.513	6.5752	1.6	1	2	1	0.773	0.713	0.89	1.14	0.988	1.071	1.175	1.383	1.37	1.39	1.72
Q9H7F0	Probable cation-transporting ATPase 13A	ATP13A3	138.04	22.71	3	3	3	3	0.906	0.84	1.358	1.025	1.229	1.071	0.965	0.69	1.36	1.32	0.95
P13473	Lysosome-associated membrane glycopro	LAMP2	44.96	20.279	5.1	2	3	2	0.884	0.735	1.094	1.104	1.166	0.746	1.272	1.049	1.36	1.18	1.43
P18440	Arylamine N-acetyltransferase 1 OS=Hor	NAT1	33.898	8.5415	3.1	1	2	1	0.887	0.778	1.096	1.163	1.061	0.918	1.154	0.946	1.36	1.19	1.26
P30043	Flavin reductase (NADPH) OS=Homo sa	BLVRB	22.119	111.63	42.7	6	11	6	0.818	0.851	1.132	1.132	0.997	0.979	1.215	1.085	1.36	1.18	1.38
P52788	Spermine synthase OS=Homo sapiens O	SMS	41.268	12.771	6.6	2	1	1	0.854	0.801	1.116	1.129	0.963	1.184	0.954	1.071	1.36	1.30	1.22
Q6P4A7	Sideroflexin-4 OS=Homo sapiens OX=9	SFXN4	37.998	10.529	7.1	2	2	2	0.732	0.686	0.831	1.088	1.152	1.093	1.066	1.565	1.35	1.58	1.86
Q02833	Ras association domain-containing protei	RASSF7	39.945	21.72	12.3	3	3	3	0.955	0.706	1.219	1.028	1.215	1.002	1.054	0.872	1.35	1.33	1.16
Q86W92	Liprin-beta-1 OS=Homo sapiens OX=96	PPFIBP1	114.02	22.385	6.6	6	7	4	0.85	0.783	1.107	1.102	1.036	1.163	0.982	1.069	1.35	1.35	1.26
O60828	Polyglutamine-binding protein 1 OS=Ho	PQBP1	30.472	66.588	29.8	7	9	7	0.883	0.837	1.142	1.184	1.042	0.976	1.004	1.02	1.35	1.17	1.18
Q9UGI8	Testin OS=Homo sapiens OX=9606 GN=	TES	47.996	179.09	44.4	16	26	16	0.817	0.762	1.035	1.1	1.086	0.976	1.179	1.161	1.35	1.31	1.48
Q01664	Transcription factor AP-4 OS=Homo sap	TFAP4	38.725	14.117	5.9	1	1	1	0.799	0.868	1.117	1.13	1.006	0.999	1.137	1.053	1.35	1.20	1.31
Q6P1K2	Polyamine-modulated factor 1 OS=Homo	PMF1	23.339	6.2793	5.9	1	1	1	1.081	0.583	1.011	1.231	1.2	0.842	1.09	0.749	1.35	1.23	1.11
P28161	Glutathione S-transferase Mu 2 OS=Hom	GSTM2	25.744	24.084	19.3	3	3	3	0.866	0.636	0.911	1.112	1.03	0.787	1.427	1.301	1.35	1.21	1.82
Q86W50	U6 small nuclear RNA (adenine-(43)-N(6	METTL16	63.62	9.8873	2.1	1	1	1	1.012	0.717	1.208	1.117	1.189	0.947	1.033	0.705	1.34	1.24	1.01
Q9NS86	LanC-like protein 2 OS=Homo sapiens C	LANCL2	50.854	43.986	17.6	6	6	6	0.841	0.724	1.054	1.049	1.158	1.001	1.081	1.089	1.34	1.38	1.39
Q9NVC3	Putative sodium-coupled neutral amino a	SLC38A7	49.965	8.6454	2.6	1	1	1	0.87	0.783	1.106	1.114	1.113	1.204	0.877	0.998	1.34	1.40	1.13
Q96MW5	Conserved oligomeric Golgi complex sub	COG8	68.423	37.504	9.5	6	7	6	0.8	0.814	1.03	1.137	1.03	0.95	1.171	1.175	1.34	1.23	1.45
Q92599	Septin-8 OS=Homo sapiens OX=9606 G	SEPT8	55.756	33.567	8.9	3	6	1	0.876	0.812	1.188	1.077	1.261	0.726	1.237	0.892	1.34	1.18	1.26
A6NDU8	UPF0600 protein C5orf51 OS=Homo sap	C5orf51	33.62	16.853	15.3	2	2	2	0.926	1.03	1.33	1.292	0.855	0.878	0.914	0.649	1.34	0.89	0.80
Q15185	Prostaglandin E synthase 3 OS=Homo sa	PTGES3	18.697	60.632	23.8	3	8	3	1.118	0.589	1.203	1.081	1.214	0.912	0.622	1.035	1.34	1.25	0.97
Q96D46	60S ribosomal export protein NMD3 OS	NMD3	57.603	35.654	10.9	5	6	5	0.932	0.855	1.189	1.202	0.936	1.109	0.872	0.851	1.34	1.14	0.96
P37268	Squalene synthase OS=Homo sapiens OX	FDFT1	48.115	68.301	26.1	9	11	8	0.864	0.771	1.126	1.061	1.108	1.124	0.978	0.981	1.34	1.37	1.20
P21399	Cytoplasmic aconitate hydratase OS=Hor	ACO1	98.398	71.408	14.8	11	11	10	0.825	0.785	1.102	1.051	1.102	1.112	1	1.053	1.34	1.38	1.28
O60563	Cyclin-T1 OS=Homo sapiens OX=9606	CCNT1	80.684	20.541	5.6	3	4	3	0.899	0.806	1.22	1.057	1.176	1.037	1.013	0.866	1.34	1.30	1.10
Q9NZ45	CDGSH iron-sulfur domain-containing p	CISD1	12.199	21.697	35.2	3	3	3	0.88	0.82	1.147	1.118	1.099	1.056	0.922	1.011	1.33	1.27	1.14
Q99933	BAG family molecular chaperone regulat	BAG1	38.778	6.8326	2.6	1	1	1	0.857	0.789	1.159	1.031	1.202	1.223	0.936	0.934	1.33	1.47	1.14
Q9NQS1	Cell death regulator Aven OS=Homo sap	AVEN	38.506	6.8326	2.5	1	1	1	0.876	0.727	1.046	1.085	1.086	1.061	1.196	0.996	1.33	1.34	1.37
P52943	Cysteine-rich protein 2 OS=Homo sapien	CRIP2	22.492	9.7042	9.1	1	2	1	0.796	0.727	1.042	0.982	1.495	1.505	0.706	0.964	1.33	1.97	1.10
Q5TC82	Roquin-1 OS=Homo sapiens OX=9606 (	RC3H1	125.73	13.241	2	2	2	2	0.876	0.727	0.964	1.166	1.44	0.919	1.046	0.863	1.33	1.47	1.19
Q9Y365	START domain-containing protein 10 OS	STARD10	33.048	77.926	23	6	8	6	0.869	0.807	1.104	1.12	1.128	1.215	0.875	0.992	1.33	1.40	1.11
Q96JH7	Deubiquitinating protein VCIP135 OS=H	VCPIP1	134.32	12.747	2	2	2	2	0.875	0.8	1.053	1.166	1.077	0.905	1.073	1.071	1.32	1.18	1.28
Q5T870	Proline-rich protein 9 OS=Homo sapiens	PRR9	12.904	8.0225	13.8	1	1	1	0.872	0.886	1.213	1.115	1.023	1.069	0.983	0.895	1.32	1.19	1.07
Q9NZQ7	Programmed cell death 1 ligand 1 OS=Ho	CD274	33.275	8.4818	5.5	1	1	1	0.884	0.817	1.155	1.097	0.991	0.883	1.224	1.01	1.32	1.10	1.31
Q86WA9	Sodium-independent sulfate anion transp	SLC26A11	65.298	13.394	3.1	2	2	2	0.817	0.85	1.175	1.028	1.205	0.985	1.291	0.799	1.32	1.31	1.25
Q9NZZ3	Charged multivesicular body protein 5 OS	CHMP5	24.57	59.718	21	4	8	4	0.865	0.878	1.222	1.08	0.993	0.911	1.071	0.972	1.32	1.09	1.17
P13987	CD59 glycoprotein OS=Homo sapiens O	CD59	14.177	22.646	17.2	2	5	2	0.845	0.801	1.05	1.123	1.075	0.912	1.192	1.053	1.32	1.21	1.36
O60306	RNA helicase aquarius OS=Homo sapien	AQR	171.29	82.903	9.2	11	16	11	0.94	0.832	1.267	1.072	1.019	1.022	0.926	0.886	1.32	1.15	1.02
Q03519	Antigen peptide transporter 2 OS=Homo	TAP2	75.663	22.213	6.4	4	4	3	0.94	0.797	1.213	1.079	1.036	0.826	1.238	0.894	1.32	1.07	1.23
Q8NBF6	Late secretory pathway protein AVL9 hor	AVL9	71.947	29.018	5.6	3	5	3	0.993	0.733	1.208	1.068	1.165	1.07	0.974	0.777	1.32	1.29	1.01
Q9NV31	U3 small nucleolar ribonucleoprotein pro	IMP3	21.85	29.621	32.1	4	5	4	0.816	0.881	1.035	1.201	0.945	1.069	1.011	1.086	1.32	1.19	1.24
O00560	Syntenin-1 OS=Homo sapiens OX=9606	SDCBP	32.444	20.097	7	1	1	1	0.768	0.786	0.984	1.061	1.177	1.277	1.04	1.091	1.32	1.58	1.37
Q6GMV2	SET and MYND domain-containing prot	SMYD5	47.34	36.633	13.2	3	3	3	0.721	1.426	1.586	1.239	0.504	1.49	0.622	0.502	1.32	0.93	0.52
Q9BUR5	MICOS complex subunit MIC26 OS=Ho	APOO	22.284	11.204	5.6	1	1	1	0.889	0.792	1.164	1.047	1.224	0.77	1.212	0.99	1.32	1.19	1.31
Q9UNS1	Protein timeless homolog OS=Homo sapi	TIMELESS	138.66	42.301	4.3	4	5	4	0.855	0.842	1.14	1.092	1.132	1.262	0.729	1.048	1.32	1.41	1.05
Q8TB52	F-box only protein 30 OS=Homo sapiens	FBXO30	82.303	17.246	3.5	2	3	2	1.03	0.52	0.953	1.083	1.54	1.132	0.64	1.06	1.31	1.72	1.10
Q92466	DNA damage-binding protein 2 OS=Hom	DDB2	47.863	39.422	13.6	5	6	5	0.838	0.789	1.118	1.019	1.13	0.965	1.148	1.04	1.31	1.29	1.34
Q9H1E3	Nuclear ubiquitous casein and cyclin-dep	NUCKS1	27.296	65.11	19.8	3	4	3	0.889	0.921	1.183	1.193	0.932	0.876	1.088	0.927	1.31	1.00	1.11
Q9HB21	Pleckstrin homology domain-containing f	PLEKHA1	45.553	6.5234	2.7	1	1	1	0.901	0.767	1.113	1.075	1.214	0.849	1.114	1.03	1.31	1.24	1.29
P36871	Phosphoglucomutase-1 OS=Homo sapier	PGM1	61.448	147.7	38.6	18	30	18	0.857	0.78	1.085	1.062	1.045	0.907	1.204	1.055	1.31	1.19	1.38
Q9H6E5	Speckle targeted PIP5K1A-regulated poly	TUT1	93.846	15.489	3.4	2	2	2	0.769	0.952	1.064	1.193	0.987	0.903	1.053	1.172	1.31	1.10	1.29
Q9Y2W6	Tudor and KH domain-containing protein	TDRKH	62.045	13.837	4.1	2	2	2	0.859	0.854	1.114	1.132	1.094	0.916	1.121	0.961	1.31	1.17	1.22
Q8NHS3	Major facilitator superfamily domain-con	MFSD8	57.627	18.999	7.3	3	3	3	0.92	0.806	1.22	1.043	1.251	0.997	0.953	0.879	1.31	1.30	1.06
Q99990	Transcription cofactor vestigial-like prote	VGLL1	28.707	41.751	25.2	4	4	4	0.881	0.867	1.169	1.122	1.05	0.84	1.216	0.895	1.31	1.08	1.21
Q96N66	Lysophospholipid acyltransferase 7 OS=l	MBOAT7	52.764	54.058	11.4	5	7	5	0.94	0.802	1.133	1.15	0.987	0.878	1.115	1.077	1.31	1.07	1.26
P01344	Insulin-like growth factor II OS=Homo sa	IGF2	20.14	17.032	13.9	2	3	2	0.809	0.708	0.979	1.009	1.292	0.979	1.246	1.187	1.31	1.50	1.60
Q8N6N3	UPF0690 protein C1orf52 OS=Homo sap	C1orf52	20.599	10.882	13.2	2	2	2	0.899	0.715	1.062	1.053	1.113	0.963	1.098	1.189	1.31	1.29	1.42
Q9UPZ3	Hermansky-Pudlak syndrome 5 protein O	HPS5	127.45	7.9912	1.2	1	2	1	0.874	0.723	1.016	1.075	1.258	0.973	1.15	1.013	1.31	1.40	1.35
P08727	Keratin, type I cytoskeletal 19 OS=Homo	KRT19	44.105	59.95	69.8	25	3	1	0.854	0.842	1.087	1.133	0.984	1.105	1.022	1.039	1.31	1.23	1.22
P78545	ETS-related transcription factor Elf-3 OS	ELF3	41.454	36.7	20.8	6	6	6	0.771	0.865	0.963	1.177	1.221	1.025	1.072	0.934	1.31	1.37	1.23
Q9Y371	Endophilin-B1 OS=Homo sapiens OX=9	SH3GLB1	40.796	22.938	8.8	4	6	3	0.796	0.77	1.045	1.003	1.165	1.517	0.938	0.984	1.31	1.71	1.23
Q8ND04	Protein SMG8 OS=Homo sapiens OX=9	SMG8	109.68	36.51	8.3	5	5	5	0.897	0.686	1.017	1.053	1.252	0.918	1.089	1.136	1.31	1.37	1.41
Q8IY17	Neuropathy target esterase OS=Homo sap	PNPLA6	149.99	55.574	4	5	5	5	0.879	0.804	1.063	1.137	1.148	0.982	1.043	0.978	1.31	1.27	1.20
Q9BQ95	Evolutionarily conserved signaling intern	ECSIT	49.148	13.209	6.3	2	2	2	0.877	0.755	1.033	1.097	1.358	0.809	1.254	0.866	1.31	1.33	1.30
Q96G03	Phosphoglucomutase-2 OS=Homo sapier	PGM2	68.283	109.72	27.1	15	19	15	0.87	0.833	1.141	1.08	1.071	0.93	1.182	1.009	1.30	1.17	1.29
Q9H3K6	BolA-like protein 2 OS=Homo sapiens C	BOLA2	10.116	20.681	22.1	1	5	1	0.883	0.848	1.138	1.119	1.033	0.909	1.124	1.011	1.30	1.12	1.23
Q8IU81	Interferon regulatory factor 2-binding pro	IRF2BP1	61.687	31.351	9.4	5	6	5	0.793	0.873	1.024	1.146	0.96	1.149	1.023	1.142	1.30	1.27	1.30
Q7Z3K6	Mesoderm induction early response prote	MIER3	61.436	19.518	5.6	3	3	3	0.858	0.739	1.015	1.064	1.329	1.024	1.016	1.061	1.30	1.47	1.30
P49711	Transcriptional repressor CTCF OS=Hon	CTCF	82.785	57.958	13.5	7	12	7	0.865	0.853	1.162	1.073	1.055	1.079	0.977	1.061	1.30	1.24	1.19
O15056	Synaptojanin-2 OS=Homo sapiens OX=9	SYNJ2	165.54	14.158	1.5	2	2	2	0.901	0.821	1.146	1.092	1.127	0.776	1.187	0.999	1.30	1.11	1.27
P53384	Cytosolic Fe-S cluster assembly factor N	NUBP1	34.534	60.72	14.7	3	4	3	0.841	0.906	1.187	1.082	1.055	0.868	1.154	0.941	1.30	1.10	1.20
P02545	Prelamin-A/C OS=Homo sapiens OX=96	LMNA	74.139	323.31	51.1	28	59	27	0.852	0.772	1.053	1.056	1.119	1.049	1.096	1.088	1.30	1.33	1.34
Q9Y232	Chromodomain Y-like protein OS=Homo	CDYL	66.481	26.995	6.2	4	5	4	0.903	0.738	1.02	1.111	1.193	0.826	1.197	1.031	1.30	1.23	1.36

P55199	RNA polymerase II elongation factor ELI	ELL	68.264	28.514	10	5	5	5	0.906	0.841	1.224	1.044	1.198	0.84	0.998	0.939	1.30	1.17	1.11
Q96CP2	FLYWCH family member 2 OS=Homo sa	FLYWCH2	14.563	24.574	30	3	4	3	0.944	0.795	1.203	1.053	1.073	0.954	1.078	0.943	1.30	1.17	1.16
Q86Y07	Serine/threonine-protein kinase VRK2 O	VRK2	58.14	37.728	12.4	5	6	5	0.938	0.756	1.178	1.019	1.139	1.036	1.036	0.931	1.30	1.28	1.16
Q15121	Astrocytic phosphoprotein PEA-15 OS=I	PEA15	15.04	62.91	23.8	2	3	2	0.784	0.929	1.105	1.116	0.885	1.102	1.107	1.095	1.30	1.16	1.29
Q13542	Eukaryotic translation initiation factor 4I	EIF4EBP2	12.939	50.576	37.5	2	2	1	0.839	0.946	1.203	1.11	0.842	1.363	0.774	1.028	1.30	1.24	1.01
Q9Y5P6	Mannose-1-phosphate guanyltransferase	GMPPB	39.834	12.35	5.6	2	2	2	0.821	0.85	1.083	1.082	1.047	1.05	1.113	1.083	1.30	1.25	1.31
P17813	Endoglin OS=Homo sapiens OX=9606 C	ENG	70.577	56.715	13.4	7	11	7	0.869	0.684	0.986	1.026	1.099	1.056	1.076	1.091	1.30	1.39	1.40
Q9NRN7	L-aminoadipate-semialdehyde dehydroge	AASDHPP	35.776	27.946	15.5	4	5	4	0.892	1.066	1.477	1.059	0.841	1.237	0.862	0.646	1.30	1.06	0.77
Q8WWI1	LIM domain only protein 7 OS=Homo sa	LMO7	192.69	55.123	5.3	5	4	4	0.855	0.846	1.12	1.083	1.111	0.989	1.102	1.006	1.30	1.23	1.24
Q5PSV4	Breast cancer metastasis-suppressor 1-lik	BRMS1L	37.629	25.022	14.9	4	4	4	0.914	0.84	1.127	1.144	1.026	1.214	0.72	1.032	1.29	1.28	1.00
O43657	Tetraspanin-6 OS=Homo sapiens OX=96	TSPAN6	27.563	34.81	18.8	4	8	4	0.896	0.853	1.119	1.145	0.973	0.97	1.116	0.987	1.29	1.11	1.20
Q5JSZ5	Protein PRRC2B OS=Homo sapiens OX	PRRC2B	242.96	193.67	14.2	21	22	20	0.903	0.833	1.176	1.071	1.172	1.22	0.953	0.934	1.29	1.38	1.09
Q96C19	EF-hand domain-containing protein D2 C	EFHD2	26.697	65.939	31.2	7	11	5	0.907	0.88	1.168	1.145	0.983	0.879	1.128	0.89	1.29	1.04	1.13
P40763	Signal transducer and activator of transcr	STAT3	88.067	39.589	7.5	5	7	5	0.929	0.874	1.151	1.181	1.039	0.922	0.938	0.932	1.29	1.09	1.04
O95159	Zinc finger protein-like 1 OS=Homo sapi	ZFPL1	34.114	13.527	7.7	2	2	2	0.836	0.875	1.131	1.082	0.925	0.925	1.224	1.121	1.29	1.08	1.37
O43896	Kinesin-like protein KIF1C OS=Homo sa	KIF1C	122.95	32.736	4.8	5	6	2	0.986	0.674	1.153	0.994	1.213	0.961	1.236	0.827	1.29	1.31	1.24
Q92882	Osteoclast-stimulating factor 1 OS=Hom	OSTF1	23.787	92.098	50.5	8	13	7	0.889	0.909	1.187	1.136	0.846	0.969	1.271	0.942	1.29	1.01	1.23
Q9H4M9	EH domain-containing protein 1 OS=Hor	EHD1	60.626	102.83	26.2	11	15	9	0.884	0.854	1.161	1.084	1.123	0.894	1.146	0.978	1.29	1.16	1.22
Q8NEB9	Phosphatidylinositol 3-kinase catalytic su	PIK3C3	101.55	35.301	7.2	6	7	6	0.844	0.95	1.122	1.194	1.075	0.97	1.166	1.11	1.29	1.14	1.27
O75146	Huntingtin-interacting protein 1-related p	HIP1R	119.39	84.639	11.9	11	14	9	0.849	0.803	1.061	1.069	1.172	1.052	1.046	1.071	1.29	1.35	1.28
P35914	Hydroxymethylglutaryl-CoA lyase, mitoc	HMGCL	34.36	13.638	7.4	2	3	2	0.987	0.813	1.301	1.019	1.084	0.979	0.998	0.858	1.29	1.15	1.03
O15254	Peroxisomal acyl-coenzyme A oxidase 3	ACOX3	77.628	14.684	5.3	2	2	2	0.696	0.932	0.978	1.12	1.101	1.05	1.131	1.195	1.29	1.32	1.43
Q7Z4W1	L-xylulose reductase OS=Homo sapiens	DCXR	25.913	53.795	38.9	7	11	7	0.88	0.861	1.259	0.984	1.22	1.193	0.928	0.801	1.29	1.39	0.99
Q96C86	m7GpppX diphosphatase OS=Homo sapi	DCPS	38.608	86.919	26.1	6	8	6	0.84	0.74	1.05	0.984	1.114	1.023	1.226	1.165	1.29	1.35	1.51
Q9UJ41	Rab5 GDP/GTP exchange factor OS=Ho	RABGEF1	79.37	37.249	6.5	3	3	3	0.899	0.803	1.089	1.102	1.1	0.924	1.099	1.032	1.29	1.19	1.25
O14920	Inhibitor of nuclear factor kappa-B kinas	IKBKB	86.563	24.268	3.4	2	2	2	0.858	0.827	1.081	1.085	1.089	0.931	1.207	1.013	1.29	1.20	1.32
P24386	Rab proteins geranylgeranyltransferase co	CHM	73.475	13.651	4.7	2	2	2	1.004	0.706	1.096	1.102	1.042	1.029	1.106	0.873	1.29	1.21	1.16
Q8WU79	Stromal membrane-associated protein 2 C	SMAP2	46.785	6.4692	5.4	1	1	1	0.871	0.966	1.12	1.241	0.728	1.508	0.765	0.773	1.29	1.22	0.84
P62256	Ubiquitin-conjugating enzyme E2 H OS=	UBE2H	20.655	27.51	25.7	4	7	4	0.906	0.915	1.185	1.155	1.054	1.174	0.718	0.93	1.29	1.22	0.90
Q14376	UDP-glucose 4-epimerase OS=Homo sap	GALE	38.281	12.128	6.9	2	2	2	0.877	0.79	1.063	1.078	1.06	0.999	1.086	1.138	1.28	1.24	1.33
Q8TF21	Ankyrin repeat domain-containing protei	ANKRD24	124.19	14.363	2.4	2	2	2	0.725	0.809	0.894	1.076	1.051	1.22	1.154	1.29	1.28	1.48	1.59
P52294	Importin subunit alpha-5 OS=Homo sapi	KPNA1	60.221	20.896	16.9	7	7	3	0.875	0.814	1.051	1.118	1.101	1.277	0.82	1.006	1.28	1.41	1.08
Q9NX57	Ras-related protein Rab-20 OS=Homo sa	RAB20	26.277	6.8078	4.3	1	1	1	0.969	0.931	1.427	1.01	1.015	1.136	0.992	0.567	1.28	1.13	0.82
Q07065	Cytoskeleton-associated protein 4 OS=H	CKAP4	66.022	240.41	35.7	15	20	15	0.864	0.875	1.105	1.125	1.016	0.967	1.059	1.042	1.28	1.14	1.21
Q6P4Q7	Metal transporter CNNM4 OS=Homo sa	CNNM4	86.606	13.856	5.8	4	6	2	0.984	0.789	1.238	1.034	1.229	1.047	0.941	0.759	1.28	1.28	0.96
Q9HA38	Zinc finger matrin-type protein 3 OS=Ho	ZMAT3	32.059	6.8767	6.2	1	1	1	0.894	0.905	1.186	1.115	1.006	0.963	1.079	0.891	1.28	1.09	1.10
O95671	N-acetylserotonin O-methyltransferase-lil	ASMTL	68.856	52.694	13	7	9	7	0.897	0.933	1.219	1.121	0.883	0.989	1.055	0.94	1.28	1.02	1.09
Q13510	Acid ceramidase OS=Homo sapiens OX=	ASAH1	44.659	37.437	14.2	5	6	5	0.885	0.835	1.127	1.072	1.103	1.246	0.852	0.969	1.28	1.37	1.06
Q96FZ2	Embryonic stem cell-specific 5-hydroxym	HMCES	40.574	49.29	22.3	6	8	6	0.813	0.767	0.953	1.066	1.177	1.076	1	1.079	1.28	1.43	1.32
P48507	Glutamate--cysteine ligase regulatory sub	GCLM	30.727	29.285	19.7	4	4	4	0.835	0.769	1.004	1.044	1.144	1.058	1.205	1.065	1.28	1.37	1.42
Q9UHD1	Cysteine and histidine-rich domain-conta	CHORDC1	37.489	221.78	46.4	11	21	11	0.891	0.926	1.207	1.112	0.998	1.176	0.93	0.907	1.28	1.20	1.01
P05549	Transcription factor AP-2-alpha OS=Hon	TFAP2A	48.062	26.568	12.8	4	4	2	0.843	0.991	1.246	1.092	1.029	1.003	1.062	0.829	1.27	1.11	1.03
Q15649	Zinc finger HIT domain-containing protei	ZNHIT3	17.607	7.6032	9	1	1	1	1.016	0.763	1.245	1.022	1.153	0.699	1.206	0.905	1.27	1.04	1.19
P38432	Coilin OS=Homo sapiens OX=9606 GN=	COIL	62.608	39.708	10.1	5	6	5	0.89	0.795	1.094	1.052	1.149	0.921	1.101	1.021	1.27	1.23	1.26
Q0VDG4	Secernin-3 OS=Homo sapiens OX=9606	SCRN3	48.544	6.3483	2.6	1	1	1	0.897	0.902	1.097	1.194	1.002	1.012	0.889	0.993	1.27	1.12	1.05
O94855	Protein transport protein Sec24D OS=Ho	SEC24D	113.01	34.939	6.1	5	5	5	0.913	0.8	1.124	1.057	1.16	1.012	1.004	1	1.27	1.27	1.17
P86452	Zinc finger BED domain-containing prot	ZBED6	109.97	33.076	6.9	5	5	5	0.868	0.802	1.062	1.063	1.064	0.985	1.051	1.183	1.27	1.23	1.34
Q9H2A9	Carbohydrate sulfotransferase 8 OS=Hon	CHST8	48.834	6.0658	3.3	1	1	1	0.976	0.794	1.198	1.054	0.991	0.851	1.282	0.867	1.27	1.04	1.21
Q9Y6X4	Soluble lamin-associated protein of 75 kI	FAM169A	74.954	23.981	8.1	3	3	3	0.932	0.765	1.121	1.038	1.062	0.979	1.017	1.031	1.27	1.20	1.21
Q9Y3B2	Exosome complex component CSL4 OS=	EXOSC1	21.452	26.995	24.6	4	4	4	0.911	0.856	1.116	1.131	1.026	1.02	1.027	0.929	1.27	1.16	1.11
Q15418	Ribosomal protein S6 kinase alpha-1 OS	RPS6KA1	82.722	72.839	19.5	10	10	9	0.854	0.816	1.047	1.076	1.075	0.957	1.193	1.063	1.27	1.22	1.35
Q17RN3	Protein FAM98C OS=Homo sapiens OX	FAM98C	37.329	10.797	4.3	1	1	1	0.812	0.888	1.155	1.005	1.184	1.363	0.893	0.89	1.27	1.50	1.05
Q96JP5	E3 ubiquitin-protein ligase ZFP91 OS=H	ZFP91	63.444	17.084	7.2	3	3	3	0.958	0.804	1.202	1.036	1.015	1.014	1	0.949	1.27	1.15	1.11
P11413	Glucose-6-phosphate 1-dehydrogenase O	G6PD	59.256	209.26	43.9	15	27	15	0.854	0.81	1.058	1.055	1.126	1.007	1.137	1.031	1.27	1.28	1.30
Q92785	Zinc finger protein ubi-d4 OS=Homo sap	DPF2	44.155	38.456	18.2	5	8	5	0.931	0.855	1.19	1.076	1.149	1.035	0.965	0.861	1.27	1.22	1.02
Q9UIS9	Methyl-CpG-binding domain protein 1 O	MBD1	66.606	20.889	6	3	3	3	0.923	0.768	1.13	1.015	1.068	0.942	1.242	1.005	1.27	1.19	1.33
O95714	E3 ubiquitin-protein ligase HERC2 OS=I	HERC2	527.22	8.5771	0.2	1	1	1	0.934	0.74	1.041	1.082	1.358	1.245	0.692	0.942	1.27	1.55	0.98
P11166	Solute carrier family 2, facilitated glucos	SLC2A1	54.083	99.913	12.2	7	12	7	0.861	0.937	1.161	1.116	1.002	1.149	0.919	0.934	1.27	1.20	1.03
Q5JPI3	Uncharacterized protein C3orf38 OS=Ho	C3orf38	37.54	15.473	6.1	2	2	2	0.874	0.811	1.053	1.08	1.22	0.953	1.14	0.946	1.27	1.29	1.24
Q14919	Dr1-associated corepressor OS=Homo sa	DRAP1	22.35	14.743	16.1	2	2	2	0.867	0.947	1.315	0.981	1.073	0.864	1.172	0.944	1.27	1.07	1.17
Q8TBK6	Zinc finger CCHC domain-containing pro	ZCCHC10	20.966	18.811	7.3	1	2	1	0.858	0.837	1.114	1.031	1.132	1.171	1.025	0.966	1.27	1.36	1.17
Q8IV48	3'-5' exoribonuclease 1 OS=Homo sapien	ERI1	40.063	12.021	9.2	2	2	2	0.838	0.842	1.044	1.082	1.164	1.358	0.774	1.014	1.27	1.50	1.06
P29350	Tyrosine-protein phosphatase non-recept	PTPN6	67.56	12.227	3.4	2	2	2	0.826	0.941	1.271	0.965	1.009	1.308	1.016	0.873	1.27	1.31	1.07
Q86VS8	Protein Hook homolog 3 OS=Homo sapi	HOOK3	83.125	41.823	10.7	7	7	7	0.978	0.803	1.177	1.076	1.162	0.674	1.296	0.822	1.27	1.03	1.19
Q9UI09	NADH dehydrogenase [ubiquinone] 1 alp	NDUFA12	17.114	47.674	42.1	4	5	4	0.944	0.779	1.142	1.037	1.217	1.131	0.845	0.969	1.26	1.36	1.05
P14060	3 beta-hydroxysteroid dehydrogenase/De	HSD3B1	42.251	75.298	20.4	5	6	5	0.867	0.834	1.031	1.119	0.942	0.779	1.266	1.197	1.26	1.01	1.45
Q8N5W9	Refilin-B OS=Homo sapiens OX=9606 C	RFLNB	22.882	16.482	21.5	2	2	2	0.922	0.787	1.054	1.104	1.075	0.949	0.986	1.163	1.26	1.18	1.26
Q9H1E5	Thioredoxin-related transmembrane prote	TMX4	38.952	9.7851	3.4	1	1	1	0.883	0.854	1.099	1.094	1.164	0.732	1.239	0.997	1.26	1.09	1.29
P14618	Pyruvate kinase PKM OS=Homo sapiens	PKM	57.936	323.31	62.5	29	96	29	0.911	0.875	1.158	1.096	0.984	0.912	1.105	0.981	1.26	1.06	1.17
Q7Z5L9	Interferon regulatory factor 2-binding pro	IRF2BP2	61.024	99.335	26.4	11	15	8	0.866	0.848	1.073	1.089	1.051	0.963	1.065	1.019	1.26	1.18	1.22
O14735	CDP-diacylglycerol--inositol 3-phosphat	CDIPT	23.539	9.9022	10.3	2	1	1	0.887	0.771	1.071	1.02	1.096	1.049	1.231	0.991	1.26	1.29	1.34
Q9NYB0	Telomeric repeat-binding factor 2-interac	TERF2IP	44.259	13.481	11.8	2	2	2	0.993	0.668	1.075	1.019	1.163	0.985	1.157	0.971	1.26	1.29	1.28
O75832	26S proteasome non-ATPase regulatory s	PSMD10	24.428	65.307	37.6	7	7	6	0.839	0.943	1.155	1.091	0.963	1.174	0.79	0.896	1.26	1.20	0.95
Q13523	Serine/threonine-protein kinase PRP4 ho	PRPF4B	116.99	55.589	8.1	7	10	7	0.916	0.82	1.099	1.088	0.977	0.997	1.103	1.011	1.26	1.14	1.22
Q5T7W7	Thiosulfate sulfurtransferase/rhodanese-l	TSTD2	58.262	6.4952	2.1	1	1	1	0.935	0.844	1.112	1.129	1.032	0.89	1.117	0.936	1.26	1.08	1.15
Q14320	Protein FAM50A OS=Homo sapiens OX	FAM50A	40.241	100.59	26.3	7	13	7	0.859	0.829	1.084	1.042	1.048	1.049	1.098	1.06	1.26	1.24	1.28
O43299	AP-5 complex subunit zeta-1 OS=Homo	AP5Z1	88.604	28.011	5.5	4	4	4	0.872	0.96	1.222	1.085	0.968	1.26	0.826	0.895	1.26	1.22	0.94
Q8NCH0	Carbohydrate sulfotransferase 14 OS=Ho	CHST14	42.996	21.466	10.6	3	4	3	0.95	0.842	1.148	1.108	0.942	1.251	0.835	0.934	1.26	1.22	0.99
Q709C8	Vacuolar protein sorting-associated prote	VPS13C	422.39	148.11	5.4	17	17	17	0.913	0.807	1.119	1.046	1.09	0.929	1.211	0.974	1.26	1.17	1.27
Q9UPN3	Microtubule-actin cross-linking factor 1,	MACF1	838.3	168.73	4	25	26	24	0.864	0.834	1.054	1.083	1.103	0.99	1.111	1.063	1.26	1.23	1.28
Q01831	DNA repair protein complementing XP-C	XPC	105.95	56.493	10.2	7	7	7	0.859	0.91	1.087	1.139	0.999	1.217	0.852	0.961	1.26	1.25	1.02
Q14207	Protein NPAT OS=Homo sapiens OX=96	NPAT	154.29	11.046	1.7	2	2	2	0.872	0.785	0.978	1.107	1.14	0.816	1.277	1.087	1.26	1.18	1.43
O95684	FGFR1 oncogene partner OS=Homo sapi	FGFR1OP	43.064	34.709	15.5	4	4	4	0.849	0.91	1.154	1.059	1.082	0.881	1.174	1.012	1.26	1.12	1.24
Q96HR9	Receptor expression-enhancing protein 6	REEP6	23.418	12.211	10	2	2	2	1.024	0.938	1.274	1.193	0.698	1.132	0.69	0.947	1.26	0.93	0.83
Q9NX70	Mediator of RNA polymerase II transcrip	MED29	21.073	38.581	18.5	3	3	3	0.866	0.826	0.993	1.133	1.122	0.945	1.07	1.1	1.26	1.22	1.28
Q9UBS8	E3 ubiquitin-protein ligase RNF14 OS=I	RNF14	53.837	24.45	8.9	4	4	4	0.873	0.808	1	1.112	1.098	1.028	1.072	1.072	1.26	1.26	1.28
Q6P9B6	TLD domain-containing protein 1 OS=Ho	TLDC1	50.993	49.515	11.8	4	5	4	0.824	0.883	1.068	1.074	0.939	1.031	1.091	1.101	1.25	1.15	1.28
O43639	Cytoplasmic protein NCK2 OS=Homo sa	NCK2	42.915	68.36	24.2	9	13	8	0.916	0.885	1.111	1.148	0.992	0.939	0.994	1.04	1.25	1.07	1.13
Q96MH6	Transmembrane protein 68 OS=Homo sa	TMEM68	37.424	13.05	7.4	2	2	2	0.796	0.947	1.12	1.066	1.034	1.203	1.094	0.896	1.25	1.28	1.14
Q96GT9	X antigen family member 2 OS=Homo sa	XAGE2	12.354	24.948	30.6	2	2	2	0.794	0.99	1.147	1.089	0.893	1.204	0.893	1.15	1.25	1.18	1.15
P43250	G protein-coupled receptor kinase 6 OS=	GRK6	65.99	30.073	11.8	5	5	5	0.563	0.973	0.886	1.038	1.268	0.929	1.315	1.27	1.25	1.43	1.68
P53801	Pituitary tumor-transforming gene 1 prote	PTTG1IP	20.324	11.561	10	1	4	1	0.901	0.926	1.215	1.073	1.054	1.067	0.872	0.912	1.25	1.16	0.98
Q96QR8	Transcriptional activator protein Pur-beta	PURB	33.24	31.473	11.5	3	4	3	0.884	0.901	1.091	1.144	1.053	1.048	0.949	0.959	1.25	1.18	1.07
Q8N392	Rho GTPase-activating protein 18 OS=H	ARHGAP1	74.976	76.701	15.7	8	9	8	0.898	0.798	1.032	1.091	1.123	1.038	1.035	1.01	1.25	1.27	1.21
Q6H9L7	Isthmin-2 OS=Homo sapiens OX=9606 C	ISM2	63.906	37.016	10.2	4	5	4	0.786	0.693	0.868	0.983	1.194	1.026	1.394	1.321	1.25	1.50	1.84
Q9NZB2	Constitutive coactivator of PPAR-gamma	FAM120A	121.89	220.38	27.7	22	28	22	0.887	0.892	1.143	1.082	1.074	1.043	0.934	0.966	1.25	1.19	1.07
Q9NNX1	Tuftelin OS=Homo sapiens OX=9606 GI	TUFT1	44.263	29.673	12.1	4	4	4	0.863	0.858	1.033	1.119	1.031	0.948	1.165	1.049	1.25	1.15	1.29
Q9Y5F9	Protocadherin gamma-B6 OS=Homo sapi	PCDHGB6	101.04	6.5531	1.8	1	1	1	0.932	0.929	1.193	1.131	1.065	0.986	0.937	0.825	1.25	1.10	0.95
Q9C0B0	RING finger protein unkempt homolog O	UNK	88.084	7.3059	1.4	1	1	1	0.891	0.824	1.001	1.14	1.098	0.854	1.147	1.071	1.25	1.14	1.29
P17096	High mobility group protein HMG-I/HM(	HMGA1	11.676	11.635	15.9	2	2	2	0.895	0.755	0.955	1.104	1.072	0.874	1.205	1.196	1.25	1.18	1.46
Q6NSJ2	Pleckstrin homology-like domain family	PHLDB3	71.911	6.9786	1.9	1	2	1	0.875	0.893	1.153	1.052	1.079	0.99	1.141	0.919	1.25	1.17	1.17
O95352	Ubiquitin-like modifier-activating enzym	ATG7	77.959	48.145	10.7	6	9	6	0.908	0.815	1.03	1.118	1.06	0.976	1.029	0.987	1.25	1.18	1.17
Q15836	Vesicle-associated membrane protein 3 C	VAMP3	11.309	134.24	40	3	7	1	0.888	0.861	1.106	1.074	1.173	0.934	1.122	0.916	1.25	1.20	1.17
Q9NP84	Tumor necrosis factor receptor superfami	TNFRSF12	13.911	6.1211	7.8	1	1	1	0.843	0.822	1.014	1.059	1.237	1.148	0.932	1.075	1.25	1.43	1.21
Q9Y6M9	NADH dehydrogenase [ubiquinone] 1 be	NDUFB9	21.831	43.868	26.3	3	4	3	0.908	0.896	1.142	1.104	1.095	0.855	1.099	0.939	1.25	1.08	1.13
Q9NRX4	14 kDa phosphohistidine phosphatase OS	PHPT1	13.832	36.821	28	4	8	4	0.893	0.809	1.053	1.066	1.126	1.099	1.087	1.099	1.25	1.31	1.28
Q9Y666	Solute carrier family 12 member 7 OS=H	SLC12A7	119.1	13.622	1.8	2	2	2	0.873	0.825	0.983	1.13	1.185	0.996	1.134	0.913	1.24	1.28	1.21
Q8N3Z6	Zinc finger CCHC domain-containing pro	ZCCHC7	63.052	6.8683	2	1	2	1	0.797	0.78	0.883	1.079	1.635	0.922	1.015	1.021	1.24	1.62	1.29
P45973	Chromobox protein homolog 5 OS=Hom	CBX5	22.225	77.449	52.9	9	13	8	0.881	0.811	1.068	1.037	1.155	1.312	0.819	1.019	1.24	1.46	1.09
Q9BUR4	Telomerase Cajal body protein 1 OS=Ho	WRAP53	59.309	31.117	12	4	5	4	0.878	0.879	1.149	1.036	1.107	0.863	1.276	0.919	1.24	1.12	1.25
Q15542	Transcription initiation factor TFIID sub	TAF5	86.829	33.988	7.1	5	5	5	0.964	0.929	1.296	1.057	0.953	1.014	1.115	0.693	1.24	1.04	0.96
Q8WWQ0	PH-interacting protein OS=Homo sapiens	PHIP	206.69	58.695	6.1	9	9	9	0.937	0.829	1.045	1.149	0.991	0.946	1.067	1.022	1.24	1.10	1.18
P32456	Guanylate-binding protein 2 OS=Homo s	GBP2	67.208	33.541	13.9	7	10	5	0.887	0.882	1.152	1.045	1.038	0.984	1.17	0.941	1.24	1.14	1.19
Q5T3F8	CSC1-like protein 2 OS=Homo sapiens C	TMEM63B	94.957	17.357	4.9	3	2	2	0.862	0.888	1.157	1.016	1.135	0.858	1.245	0.976	1.24	1.14	1.27
Q7Z7A3	Cytoplasmic tRNA 2-thiolation protein 1	CTU1	36.45	13.094	4.3	1	1	1	0.883	0.876	1.114	1.069	1.099	0.906	1.161	0.975	1.24	1.14	1.21
P51452	Dual specificity protein phosphatase 3 O	DUSP3	20.478	48.5	36.2	5	10	5	0.872	0.883	1.092	1.086	0.995	0.973	1.116	1.017	1.24	1.12	1.22
Q70E73	Ras-associated and pleckstrin homology	RAPH1	135.25	52.036	6.2	6	7	5	0.883	0.89	1.141	1.059	0.975	0.967	1.173	1.007	1.24	1.10	1.23
P51608	Methyl-CpG-binding protein 2 OS=Hom	MECP2	52.44	14.203	6	2	3	2	0.946	0.788	1.109	1.042	1.067	0.995	1	1.121	1.24	1.19	1.22
P29122	Proprotein convertase subtilisin/kexin ty	PCSK6	106.42	54.97	5.6	5	5	5	0.856	1.007	1.218	1.093	0.94	1.163	0.947	0.868	1.24	1.13	0.97
P50583	Bis(5'-nucleosyl)-tetraphosphatase [asym	NUDT2	16.829	14.776	19.7	2	2	2	0.877	0.835	1.059	1.064	1.071	1.19	1.009	0.993	1.24	1.32	1.17
Q9HAJ7	Histone deacetylase complex subunit SA	SAP30L	20.876	10.007	14.2	1	1	1	0.879	0.909	1.276	0.941	0.968	1.055	1.329	0.821	1.24	1.13	1.20
Q9ULS5	Transmembrane and coiled-coil domain p	TMCC3	53.784	13.937	5.5	2	2	2	0.944	0.9	1.48	0.806	1.252	1.19	1.09	0.554	1.24	1.32	0.89
Q9UH65	Switch-associated protein 70 OS=Homo	SWAP70	68.997	154.54	26.8	14	18	14	0.899	0.833	1.076	1.07	1.025	0.985	1.043	1.052	1.24	1.16	1.21
Q5T1V6	Probable ATP-dependent RNA helicase I	DDX59	68.809	93.822	22.5	12	15	12	0.846	0.802	0.998	1.043	1.095	0.996	1.171	1.082	1.24	1.27	1.37
Q9GZT4	Serine racemase OS=Homo sapiens OX=	SRR	36.566	19.609	10	2	2	2	0.867	0.866	1.094	1.052	1.193	0.829	1.174	1.034	1.24	1.17	1.27
P63167	Dynein light chain 1, cytoplasmic OS=Ho	DYNLL1	10.366	82.838	37.1	2	12	1	0.965	0.787	1.083	1.086	1.079	0.905	1.049	1.101	1.24	1.13	1.23
Q9H987	Synaptopodin 2-like protein OS=Homo s	SYNPO2L	102.48	6.2031	1.1	1	1	1	0.889	0.866	1.063	1.109	1.172	1.196	0.839	0.918	1.24	1.35	1.00
O96007	Molybdopterin synthase catalytic subunit	MOCS2	20.944	23.76	17.6	3	3	3	0.828	0.891	1.108	1.019	1.07	1.088	1.119	1.048	1.24	1.26	1.26
O95376	E3 ubiquitin-protein ligase ARIH2 OS=H	ARIH2	57.818	60.838	16.2	5	4	4	0.88	0.858	1.091	1.059	1.006	1.018	1.07	0.941	1.24	1.16	1.16
Q9NR45	Sialic acid synthase OS=Homo sapiens C	NANS	40.307	123.44	32	8	11	8	0.846	0.905	1.093	1.073	1.072	0.993	1.034	1.104	1.24	1.18	1.22
Q5VZK9	F-actin-uncapping protein LRRC16A OS	CARMIL1	151.56	50.692	6.1	7	8	7	0.899	0.82	1.035	1.091	1.055	1.063	1.018	1.02	1.24	1.23	1.19
Q14435	Polypeptide N-acetylgalactosaminyltrans	GALNT3	72.609	22.054	7.6	4	5	3	0.88	0.849	1.068	1.07	1.121	1.012	1.063	1.063	1.24	1.23	1.23
Q96S94	Cyclin-L2 OS=Homo sapiens OX=9606	CCNL2	58.147	29.478	8.5	3	4	3	1.053	1.241	1.945	0.891	0.912	1.187	0.604	0.24	1.24	0.91	0.37
Q03518	Antigen peptide transporter 1 OS=Homo	TAP1	87.217	45.073	10.4	6	6	5	0.885	0.906	1.114	1.1	0.97	1.02	0.988	0.985	1.24	1.11	1.10
O14787	Transportin-2 OS=Homo sapiens OX=96	TNPO2	101.39	12.796	8.9	7	13	2	0.96	0.9	1.124	1.174	0.946	0.783	1.109	0.949	1.24	0.93	1.11
Q9H6F5	Coiled-coil domain-containing protein 86	CCDC86	40.235	50.408	25.3	7	7	7	0.817	0.815	0.938	1.078	1.106	1.022	1.29	1.129	1.24	1.30	1.48
O43396	Thioredoxin-like protein 1 OS=Homo sa	TXNL1	32.251	40.471	24.6	4	6	4	0.899	0.943	1.172	1.103	1.048	1.028	0.935	0.896	1.24	1.13	0.99
Q9NQU5	Serine/threonine-protein kinase PAK 6 O	PAK6	74.868	48.788	11.5	7	7	7	0.824	0.797	0.996	1.006	1.204	1.285	0.92	1.156	1.24	1.54	1.28
Q7Z478	ATP-dependent RNA helicase DHX29 O	DHX29	155.23	118.47	12.5	13	14	12	0.886	0.891	1.142	1.049	1.021	0.992	1.056	1.028	1.23	1.13	1.17
Q9BV40	Vesicle-associated membrane protein 8 C	VAMP8	11.438	38.199	41	5	5	5	0.876	0.98	1.248	1.04	1.126	1.038	0.941	0.921	1.23	1.17	1.00
P00813	Adenosine deaminase OS=Homo sapiens	ADA	40.764	12.402	7.4	2	2	2	0.906	0.862	1.081	1.098	1.038	0.912	1.097	1.056	1.23	1.10	1.22
Q9UHD9	Ubiquilin-2 OS=Homo sapiens OX=9606	UBQLN2	65.695	92.138	19.9	7	11	6	0.985	0.763	1.119	1.035	1.15	0.926	1.191	1	1.23	1.19	1.25
Q9Y6Q1	Calpain-6 OS=Homo sapiens OX=9606 C	CAPN6	74.576	80.602	20	11	19	10	0.868	0.911	1.09	1.102	0.929	0.88	1.175	1.049	1.23	1.02	1.25
Q15642	Cdc42-interacting protein 4 OS=Homo sa	TRIP10	68.351	89.53	28.5	13	15	12	0.932	0.912	1.166	1.106	0.95	0.991	1.065	1.068	1.23	1.05	1.16
P22570	NADPH:adrenodoxin oxidoreductase, mi	FDXR	53.836	104.92	35.8	13	16	13	0.896	0.872	1.091	1.087	1.053	1.065	0.99	1.017	1.23	1.20	1.14
Q9Y5U9	Immediate early response 3-interacting pr	IER3IP1	8.9687	9.902	24.4	1	1	1	0.951	0.718	0.978	1.078	1.234	0.923	1.148	0.989	1.23	1.29	1.28
Q9NZU5	LIM and cysteine-rich domains protein 1	LMCD1	40.832	6.712	2.2	1	1	1	0.871	0.877	1.107	1.046	1.103	0.93	1.326	0.841	1.23	1.16	1.24
Q05655	Protein kinase C delta type OS=Homo sa	PRKCD	77.504	54.394	13.5	8	9	8	0.9	0.89	1.212	0.992	1.076	0.97	0.875	0.967	1.23	1.14	1.03
Q14653	Interferon regulatory factor 3 OS=Homo	IRF3	47.219	23.447	5.4	2	2	2	0.946	0.915	1.244	1.047	1.067	0.781	1.154	0.895	1.23	0.99	1.10
Q13131	5'-AMP-activated protein kinase catalytic	PRKAA1	64.009	31.688	13.2	5	6	5	0.91	0.856	1.122	1.051	1.204	0.888	1.078	1.08	1.23	1.18	1.22
Q15742	NGFI-A-binding protein 2 OS=Homo sap	NAB2	56.593	33.311	12	5	5	5	0.856	0.863	1.017	1.098	1.04	1.25	0.738	1.245	1.23	1.33	1.15
Q86VN1	Vacuolar protein-sorting-associated prote	VPS36	43.816	35.535	13	4	5	4	0.871	0.94	1.122	1.106	0.935	1.153	0.842	0.966	1.23	1.15	1.00
Q9UNI6	Dual specificity protein phosphatase 12 C	DUSP12	37.687	27.806	8.5	2	2	2	0.964	0.873	1.256	1.003	0.972	1.026	1.129	0.846	1.23	1.09	1.08
Q8NBN7	Retinol dehydrogenase 13 OS=Homo sap	RDH13	35.932	13.992	7.3	2	3	2	0.874	1.092	1.284	1.132	1.186	1.069	0.997	0.385	1.23	1.15	0.70
P17535	Transcription factor jun-D OS=Homo sap	JUND	35.173	21.454	11	2	4	2	0.867	0.907	1.079	1.1	0.942	1.106	1.033	1.074	1.23	1.15	1.19
Q9ULT8	E3 ubiquitin-protein ligase HECTD1 OS	HECTD1	289.38	42.316	3	6	6	6	0.902	0.829	1.052	1.074	1.14	0.946	1.141	0.984	1.23	1.21	1.23

Q5VYS8	Terminal uridylyltransferase 7 OS=Homo	TUT7	171.23	32.21	4.1	5	5	5	0.93	0.786	0.968	1.139	1.083	0.983	1.003	1.111	1.23	1.20	1.23
Q86U06	Probable RNA-binding protein 23 OS=H	RBM23	48.73	12.045	8.7	3	6	2	0.818	0.858	0.995	1.062	1.159	1.172	0.992	1.095	1.23	1.39	1.25
Q9BZL6	Serine/threonine-protein kinase D2 OS=I	PRKD2	96.721	56.547	11.3	6	6	6	0.899	0.919	1.111	1.12	1.036	1.014	0.966	0.973	1.23	1.13	1.07
Q01469	Fatty acid-binding protein 5 OS=Homo s	FABP5	15.164	78.874	60	8	14	8	0.94	0.849	1.099	1.096	0.945	0.91	1.09	1.047	1.23	1.04	1.19
P50750	Cyclin-dependent kinase 9 OS=Homo sa	CDK9	42.777	22.046	9.7	3	3	2	0.883	0.745	0.977	1.02	1.207	0.978	1.215	1.097	1.23	1.34	1.42
Q9Y248	DNA replication complex GINS protein I	GINS2	21.427	22.235	23.2	3	4	3	0.911	0.788	1.062	1.021	1.109	0.962	1.189	1.07	1.23	1.22	1.33
Q16576	Histone-binding protein RBBP7 OS=Ho	RBBP7	47.82	121.03	26.4	9	15	6	0.813	0.963	1.096	1.081	0.952	1.205	0.923	1.019	1.23	1.21	1.09
P08243	Asparagine synthetase [glutamine-hydrol	ASNS	64.369	113.06	32.8	15	18	15	0.833	0.793	0.933	1.059	1.065	0.975	1.131	1.177	1.23	1.25	1.42
P45877	Peptidyl-prolyl cis-trans isomerase C OS	PPIC	22.763	15.002	6.1	1	4	1	0.951	0.851	1.141	1.066	1.158	0.95	1.031	0.927	1.22	1.17	1.09
P33991	DNA replication licensing factor MCM4	MCM4	96.557	169.01	30.2	23	28	21	0.877	0.836	1.066	1.03	1.158	0.972	1.091	1.022	1.22	1.24	1.23
Q7L523	Ras-related GTP-binding protein A OS=I	RRAGA	36.566	26.202	10.2	3	5	3	0.921	0.781	1.037	1.045	1.094	0.985	1.152	1.024	1.22	1.22	1.28
P54252	Ataxin-3 OS=Homo sapiens OX=9606 G	ATXN3	41.25	25.739	14.1	4	4	4	0.804	0.999	1.253	0.952	1.152	1.02	1.044	1.016	1.22	1.20	1.14
Q9UQC2	GRB2-associated-binding protein 2 OS=	GAB2	74.457	21.209	6.4	3	3	3	0.973	0.633	0.938	1.026	1.46	0.579	1.312	1.115	1.22	1.27	1.51
P15311	Ezrin OS=Homo sapiens OX=9606 GN=	EZR	69.412	177.54	33.4	22	43	13	0.879	0.833	1.001	1.09	1.046	0.922	1.13	1.132	1.22	1.15	1.32
Q86YN1	Dolichyldiphosphatase 1 OS=Homo sapi	DOLPP1	27.03	13.533	8	1	1	1	1.024	0.728	1.04	1.099	1.026	0.921	1.077	1.039	1.22	1.11	1.21
P42694	Probable helicase with zinc finger domai	HELZ	218.97	36.636	3.1	5	5	5	0.945	0.826	1.078	1.084	1.135	0.959	1.097	0.896	1.22	1.18	1.13
P42575	Caspase-2 OS=Homo sapiens OX=9606	CASP2	50.685	7.2248	2.4	1	1	1	0.856	0.861	1.088	1.007	1.198	0.841	1.281	1.016	1.22	1.19	1.34
O15228	Dihydroxyacetone phosphate acyltransfe	GNPAT	77.187	42.097	10.4	5	5	4	0.929	0.828	0.914	1.229	0.92	0.953	1.064	1.101	1.22	1.07	1.23
Q4KMQ1	Taperin OS=Homo sapiens OX=9606 GN	TPRN	75.555	21.061	6.5	3	3	3	0.883	0.828	1.051	1.035	1.144	0.847	1.229	1.096	1.22	1.16	1.36
Q8N6H7	ADP-ribosylation factor GTPase-activati	ARFGAP2	56.72	44.969	13.4	5	6	5	0.905	0.863	1.121	1.034	1.062	0.96	1.073	0.935	1.22	1.14	1.14
O00170	AH receptor-interacting protein OS=Hom	AIP	37.636	75.009	25.5	7	9	7	0.923	0.893	1.101	1.112	1.065	0.981	1.078	1.03	1.22	1.13	1.16
Q96GM8	Target of EGR1 protein 1 OS=Homo sapi	TOE1	56.547	79.037	16.5	5	5	5	0.914	0.869	1.09	1.082	1.197	1.049	1.012	0.966	1.22	1.26	1.11
O95758	Polypyrimidine tract-binding protein 3 O	PTBP3	59.689	51	15.2	7	9	6	0.973	0.834	1.159	1.042	1.045	0.992	1.123	0.986	1.22	1.13	1.17
P04920	Anion exchange protein 2 OS=Homo sap	SLC4A2	137.01	75.722	11.1	9	11	9	0.897	0.865	1.1	1.046	1.063	0.94	1.078	1.023	1.22	1.14	1.19
P0DJD4	RNA-binding motif protein, Y chromoso	RBMY1C	55.783	89.642	18.5	7	11	7	0.917	0.882	1.147	1.042	1.105	1.094	0.91	0.981	1.22	1.22	1.05
Q9NR19	Acetyl-coenzyme A synthetase, cytoplasn	ACSS2	78.579	31.09	7.3	5	7	5	0.893	0.842	1.048	1.063	1.077	1.148	0.995	1.015	1.22	1.28	1.16
Q8N302	Angiogenic factor with G patch and FHA	AGGF1	80.976	6.2494	1.5	1	1	1	0.91	0.798	0.946	1.132	1.171	1.246	0.769	1.052	1.22	1.42	1.07
O14524	Nuclear envelope integral membrane prot	NEMP1	50.639	13.925	5.2	2	2	2	0.929	0.808	1.062	1.051	1.128	0.93	1.196	0.954	1.22	1.18	1.24
Q13885	Tubulin beta-2A chain OS=Homo sapien	TUBB2A	49.906	26.756	50.1	18	62	2	0.925	0.9	1.163	1.056	0.889	0.79	1.375	1.021	1.22	0.92	1.31
Q9UNP9	Peptidyl-prolyl cis-trans isomerase E OS	PPIE	33.43	21.652	17.6	4	3	3	0.968	0.82	1.054	1.12	1.183	1.007	0.824	1.009	1.22	1.22	1.03
P09972	Fructose-bisphosphate aldolase C OS=H	ALDOC	39.455	48.389	25.5	7	17	4	0.92	0.818	1.038	1.075	0.967	0.858	1.147	1.097	1.22	1.05	1.29
Q6ZRV2	Protein FAM83H OS=Homo sapiens OX	FAM83H	127.12	130.88	17.8	14	18	14	0.836	0.899	1.062	1.047	1.088	0.996	1.104	1.048	1.22	1.20	1.24
Q9H173	Nucleotide exchange factor SIL1 OS=Ho	SIL1	52.084	38.51	12.4	5	6	5	0.974	0.941	1.23	1.097	1.117	0.883	1.239	0.751	1.22	1.04	1.04
P41229	Lysine-specific demethylase 5C OS=Hon	KDM5C	175.72	25.639	4.4	5	5	3	0.843	0.775	0.8	1.166	1.421	0.821	1.332	0.865	1.22	1.39	1.36
P16383	GC-rich sequence DNA-binding factor 2	GCFC2	89.384	12.235	3.2	2	2	2	0.916	0.876	1.117	1.06	1.041	0.963	1.098	0.998	1.21	1.12	1.17
Q9H4A4	Aminopeptidase B OS=Homo sapiens O	RNPEP	72.595	246.92	46.2	23	48	22	0.889	0.871	1.071	1.067	1.057	1.026	1.081	1.007	1.21	1.18	1.19
Q9ULQ0	Striatin-interacting protein 2 OS=Homo s	STRIP2	95.358	30.181	5.5	4	4	2	0.875	0.845	1.171	0.918	1.263	0.893	1.213	1.025	1.21	1.25	1.30
Q96T60	Bifunctional polynucleotide phosphatase	PNKP	57.076	31.897	10.2	5	6	5	0.934	0.905	1.123	1.11	1.123	0.982	1.036	0.858	1.21	1.14	1.03
Q8N573	Oxidation resistance protein 1 OS=Homo	OXR1	97.969	8.3086	1	1	1	1	0.985	0.786	1.172	0.978	1.205	1.01	1.062	0.869	1.21	1.25	1.09
Q14691	DNA replication complex GINS protein I	GINS1	22.988	13.052	14.8	2	2	2	0.831	0.898	1.029	1.069	1.043	1.017	1.135	1.113	1.21	1.19	1.30
Q13049	E3 ubiquitin-protein ligase TRIM32 OS=	TRIM32	71.988	55.18	8.7	5	6	5	0.921	0.851	1.065	1.085	0.992	0.974	1.153	0.964	1.21	1.11	1.19
Q53S08	Ras-related protein Rab-6D OS=Homo s	RAB6D	28.242	21.554	8.7	1	2	1	0.929	0.854	1.082	1.081	1.049	1.005	1.068	0.975	1.21	1.15	1.15
Q9BV86	N-terminal Xaa-Pro-Lys N-methyltransfe	NTMT1	25.387	41.145	30	5	7	5	0.91	0.719	0.987	0.989	1.143	1.033	1.093	1.171	1.21	1.34	1.39
P54619	5'-AMP-activated protein kinase subunit	PRKAG1	37.579	44.406	23	7	10	7	0.93	0.92	1.127	1.116	1.074	0.986	0.898	0.956	1.21	1.11	1.00
P51948	CDK-activating kinase assembly factor M	MNAT1	35.823	29.525	14.9	4	5	4	0.897	0.866	1.116	1.02	0.886	0.901	1.208	0.962	1.21	1.01	1.23
Q66K74	Microtubule-associated protein 1S OS=H	MAP1S	112.21	47.587	7.5	7	8	7	0.91	0.902	1.11	1.085	1.038	0.85	1.194	0.96	1.21	1.04	1.19
Q9UH99	SUN domain-containing protein 2 OS=H	SUN2	80.31	126.09	18.1	8	9	8	0.893	0.944	1.149	1.076	1.044	0.995	1.056	1.01	1.21	1.11	1.12
P51159	Ras-related protein Rab-27A OS=Homo	RAB27A	24.868	21.126	16.3	3	3	3	0.859	0.728	0.948	0.974	1.263	1.007	1.283	1.112	1.21	1.43	1.51
O00515	Ladinin-1 OS=Homo sapiens OX=9606	LAD1	57.13	71.981	21.5	10	14	10	0.918	0.963	1.148	1.13	0.967	0.963	1.012	0.981	1.21	1.03	1.06
P49748	Very long-chain specific acyl-CoA dehyd	ACADVL	70.389	132.75	27	15	25	15	0.95	0.856	1.106	1.081	1.002	0.826	1.181	1.004	1.21	1.01	1.21
Q86VI3	Ras GTPase-activating-like protein IQGA	IQGAP3	184.7	14.23	2	3	4	2	0.839	0.884	1.087	0.999	1.019	0.894	1.372	1.082	1.21	1.11	1.42
P09211	Glutathione S-transferase P OS=Homo s	GSTP1	23.356	228.1	51.4	9	30	9	0.908	0.945	1.128	1.115	0.973	0.94	1.086	0.922	1.21	1.03	1.08
Q9Y5A7	NEDD8 ultimate buster 1 OS=Homo sap	NUB1	70.537	10.282	2	1	1	1	0.947	0.903	1.168	1.071	0.993	0.991	1.028	0.936	1.21	1.07	1.06
Q00013	55 kDa erythrocyte membrane protein OS	MPP1	52.296	52.25	16.1	7	11	7	0.9	0.855	1.068	1.056	1.069	1.244	0.898	0.937	1.21	1.32	1.05
P04818	Thymidylate synthase OS=Homo sapiens	TYMS	35.716	44.352	20.8	5	8	5	0.643	0.533	0.613	0.81	1.571	1.024	1.459	1.737	1.21	2.21	2.72
Q8N490	Probable hydrolase PNKD OS=Homo sa	PNKD	42.875	10.554	6.2	1	1	1	0.878	0.975	1.047	1.194	0.973	0.781	0.977	1.185	1.21	0.95	1.17
P22830	Ferrochelatase, mitochondrial OS=Homo	FECH	47.862	29.008	9.9	3	4	3	0.886	0.801	0.987	1.053	1.131	1.022	1.112	1.107	1.21	1.28	1.32
Q8NFH5	Nucleoporin NUP35 OS=Homo sapiens	NUP35	34.773	82.054	22.4	5	7	5	0.893	0.876	1.111	1.028	1.046	1.13	0.978	1.007	1.21	1.23	1.12
Q8WUA2	Peptidyl-prolyl cis-trans isomerase-like 4	PPIL4	57.224	37.934	12.4	4	6	4	0.947	0.801	1.007	1.106	1.087	0.918	1.092	1.028	1.21	1.15	1.21
Q13867	Bleomycin hydrolase OS=Homo sapiens	BLMH	52.562	23.688	7.9	3	3	3	0.966	0.941	1.242	1.063	0.993	0.833	1.159	0.823	1.21	0.96	1.04
Q96ME1	F-box/LRR-repeat protein 18 OS=Homo	FBXL18	88.34	54.154	11.2	6	9	6	0.873	0.918	1.087	1.076	1.02	1.031	1.081	0.948	1.21	1.15	1.13
Q9BUQ8	Probable ATP-dependent RNA helicase I	DDX23	95.581	120.25	20.9	15	19	15	0.893	0.855	1.065	1.046	1.132	1.034	1.089	1.007	1.21	1.24	1.20
O00590	Atypical chemokine receptor 2 OS=Hom	ACKR2	43.442	13.918	3.6	1	1	1	0.9	0.815	0.994	1.077	1.036	1.071	1.068	1.116	1.21	1.23	1.27
Q96CV9	Optineurin OS=Homo sapiens OX=9606	OPTN	65.921	12.748	3.1	2	2	2	1.005	0.763	1.125	1.009	1.25	0.697	1.286	0.887	1.21	1.10	1.23
Q9UKT5	F-box only protein 4 OS=Homo sapiens	FBXO4	44.136	14.86	4.7	2	3	2	0.945	0.907	1.178	1.056	1.136	1.129	0.872	0.822	1.21	1.22	0.91
Q14554	Protein disulfide-isomerase A5 OS=Hom	PDIA5	59.594	63.993	19.7	9	11	9	0.904	0.9	1.089	1.087	1.094	1.19	0.802	0.972	1.21	1.27	0.98
Q6ZRS2	Helicase SRCAP OS=Homo sapiens OX	SRCAP	343.55	56.055	3	9	12	9	1	0.695	1	1.043	1.216	0.93	1.109	1.023	1.21	1.27	1.26
Q9NVQ4	Fas apoptotic inhibitory molecule 1 OS=	FAIM	20.215	29.761	26.3	4	5	4	0.891	0.856	1.075	1.03	1.04	1.102	1.012	1.018	1.20	1.23	1.16
Q9H204	Mediator of RNA polymerase II transcrip	MED28	19.52	7.0815	5.1	1	1	1	1.02	0.83	1.147	1.082	1.155	0.833	1.02	0.877	1.20	1.07	1.03
Q9UGL1	Lysine-specific demethylase 5B OS=Hon	KDM5B	175.66	192.48	16.6	21	25	19	0.933	0.881	1.113	1.072	1.049	1.006	0.946	0.925	1.20	1.13	1.03
Q9H5V9	UPF0428 protein CXorf56 OS=Homo sa	CXorf56	25.624	34.92	23.4	5	6	5	0.938	0.951	1.123	1.152	0.974	1.143	0.743	0.967	1.20	1.12	0.91
Q12899	Tripartite motif-containing protein 26 OS	TRIM26	62.165	8.3022	2	1	1	1	0.87	0.854	0.929	1.147	1.086	1.065	1.102	0.986	1.20	1.25	1.21
Q14746	Conserved oligomeric Golgi complex sub	COG2	83.207	59.49	13.4	7	8	7	0.916	0.967	1.178	1.088	1.05	1.111	0.894	0.884	1.20	1.15	0.94
Q96DU7	Inositol-trisphosphate 3-kinase C OS=H	ITPKC	75.207	7.0606	1.5	1	1	1	1.017	0.898	1.172	1.131	0.945	0.774	1.008	0.996	1.20	0.90	1.05
Q14108	Lysosome membrane protein 2 OS=Hom	SCARB2	54.29	37.355	13.2	5	4	4	0.999	0.724	1.055	1.017	1.038	1.337	0.975	1.031	1.20	1.38	1.16
Q9UPU7	TBC1 domain family member 2B OS=H	TBC1D2B	109.88	7.0933	1.7	1	1	1	0.872	0.926	1.068	1.094	1.076	1.337	0.769	0.942	1.20	1.34	0.95
Q9H6H4	Receptor expression-enhancing protein 4	REEP4	29.395	6.4629	3.9	1	2	1	0.907	0.963	1.068	1.18	0.98	0.968	0.993	0.925	1.20	1.04	1.03
O00193	Small acidic protein OS=Homo sapiens	SMAP	20.332	36.154	13.1	2	5	2	0.918	0.893	1.147	1.03	0.984	0.964	1.05	0.99	1.20	1.08	1.13
Q6P2H3	Centrosomal protein of 85 kDa OS=Hom	CEP85	85.638	31.621	5.6	4	4	4	0.929	0.858	1.059	1.089	1.138	0.903	1.151	1.023	1.20	1.14	1.22
P07858	Cathepsin B OS=Homo sapiens OX=960	CTSB	37.821	53.017	13.6	4	5	4	0.908	0.911	1.092	1.094	0.945	0.934	1.137	1.031	1.20	1.03	1.19
Q96EK7	Constitutive coactivator of peroxisome p	FAM120B	103.78	20.499	4.1	3	3	3	0.903	0.925	1.148	1.048	1.144	1.111	0.916	0.892	1.20	1.23	0.99
Q96EW2	HSPB1-associated protein 1 OS=Homo s	HSPBAP1	55.166	14.998	6.6	2	2	2	0.873	0.858	1.008	1.071	1.224	0.962	1.079	1.015	1.20	1.26	1.21
Q6WCQ1	Myosin phosphatase Rho-interacting pro	MPRIP	116.53	130	16.3	12	13	11	0.915	0.843	1.05	1.061	1.106	0.956	1.05	1.06	1.20	1.17	1.20
Q9NZ08	Endoplasmic reticulum aminopeptidase 1	ERAP1	107.23	50.667	9.1	8	10	8	0.902	0.91	1.074	1.101	0.971	1.066	0.968	1.016	1.20	1.12	1.09
Q8N2E2	von Willebrand factor D and EGF domai	VWDE	176.78	42.145	4.3	6	7	6	0.975	0.912	1.218	1.047	0.966	1.005	0.93	1.041	1.20	1.04	1.04
Q9HDC9	Adipocyte plasma membrane-associated	APMAP	46.48	74.213	20	7	9	7	0.914	0.849	1.053	1.063	1.067	1.096	0.997	1.046	1.20	1.23	1.16
Q6P6C2	RNA demethylase ALKBH5 OS=Homo s	ALKBH5	44.255	14.453	6.6	2	2	2	0.942	0.851	1.091	1.061	1.115	0.984	1.049	0.952	1.20	1.17	1.12
P36507	Dual specificity mitogen-activated protei	MAP2K2	44.424	84.002	30.5	10	15	6	0.901	0.892	1.096	1.056	0.968	1.034	0.962	0.931	1.20	1.12	1.06
O15047	Histone-lysine N-methyltransferase SETI	SETD1A	186.03	29.828	2.9	3	3	3	0.932	1	1.216	1.102	0.993	1.236	0.814	0.728	1.20	1.15	0.80
Q9H4L5	Oxysterol-binding protein-related protein	OSBPL3	101.22	57.062	11.4	8	10	8	0.866	0.846	0.98	1.074	1.074	0.997	1.069	1.155	1.20	1.21	1.30
O00479	High mobility group nucleosome-binding	HMGN4	9.5388	6.9791	18.9	2	2	1	0.8	0.761	0.805	1.067	1.177	0.752	1.342	1.462	1.20	1.24	1.80
O15118	NPC intracellular cholesterol transporter	NPC1	142.17	37.651	3.8	4	5	4	0.851	0.944	1.045	1.107	0.983	0.929	1.2	1.078	1.20	1.07	1.27
Q14186	Transcription factor Dp-1 OS=Homo sap	TFDP1	45.07	14.428	7.1	2	2	2	0.901	0.916	1.082	1.096	1.078	1.221	0.821	0.942	1.20	1.27	0.97
Q96Q15	Serine/threonine-protein kinase SMG1 O	SMG1	410.5	60.051	2.8	8	9	8	0.916	0.899	1.134	1.041	1.016	0.945	1.186	0.957	1.20	1.08	1.18
O94776	Metastasis-associated protein MTA2 OS	MTA2	75.022	114.66	18.7	11	21	10	0.825	0.901	1.043	1.025	1.17	1.268	0.967	1.093	1.20	1.41	1.19
O75223	Gamma-glutamylcyclotransferase OS=Ho	GGCT	21.007	60.943	37.2	6	12	6	0.912	0.81	0.985	1.078	1.082	1.02	1.058	1.109	1.20	1.22	1.26
O95983	Methyl-CpG-binding domain protein 3 O	MBD3	32.844	31.324	22.3	5	4	4	0.892	0.891	1.099	1.036	1.089	1.169	1.038	0.959	1.20	1.27	1.12
Q9UJA5	tRNA (adenine(58)-N(1))-methyltransfer	TRMT6	55.799	46.588	13.3	6	8	6	0.887	0.874	1.07	1.037	1.049	1.2	0.888	1.067	1.20	1.28	1.11
Q9UJY5	ADP-ribosylation factor-binding protein	GGA1	70.383	30.456	7.2	4	4	4	0.92	0.87	1.069	1.072	1.059	0.872	1.142	1.051	1.20	1.08	1.23
P84085	ADP-ribosylation factor 5 OS=Homo sap	ARF5	20.529	28.747	41.1	7	15	3	0.88	0.946	1.065	1.118	0.938	0.91	1.167	1.034	1.20	1.01	1.21
O95822	Malonyl-CoA decarboxylase, mitochond	MLYCD	55.003	29.286	9.3	4	4	4	0.905	0.845	1.077	1.015	1.118	0.97	1.208	0.969	1.20	1.19	1.24
O15357	Phosphatidylinositol 3,4,5-trisphosphate	INPPL1	138.6	77.597	9.7	10	11	10	0.891	0.901	1.118	1.024	1.042	0.946	1.141	0.994	1.20	1.11	1.19
P41250	Glycine--tRNA ligase OS=Homo sapiens	GARS	83.165	122.06	24.5	16	19	16	0.914	0.865	1.067	1.059	1.005	0.927	1.16	1.05	1.20	1.09	1.24
P61081	NEDD8-conjugating enzyme Ubc12 OS=	UBE2M	20.9	51.046	36.1	6	9	6	0.922	0.867	1.094	1.043	1.062	0.987	1.065	0.979	1.19	1.15	1.14
P19838	Nuclear factor NF-kappa-B p105 subunit	NFKB1	105.35	31.186	6.6	4	4	4	0.985	0.872	1.156	1.062	1.1	0.892	1.082	0.853	1.19	1.07	1.04
P00488	Coagulation factor XIII A chain OS=Hon	F13A1	83.266	15.546	3.6	2	4	2	1.328	1.288	1.409	1.715	1.097	0.536	1.144	1.498	1.19	0.62	1.01
Q14451	Growth factor receptor-bound protein 7 C	GRB7	59.68	24.602	10	4	4	4	0.913	0.895	1.084	1.075	1.061	1.082	1.056	0.955	1.19	1.19	1.11
O00499	Myc box-dependent-interacting protein 1	BIN1	64.699	42.13	13.5	6	8	5	0.965	0.942	1.257	1.02	1.203	1.415	0.638	0.744	1.19	1.37	0.72
Q9H3R5	Centromere protein H OS=Homo sapiens	CENPH	28.481	19.868	11.7	3	4	3	0.888	0.821	1.039	1.001	1.243	0.941	1.142	1.058	1.19	1.28	1.29
Q53H12	Acylglycerol kinase, mitochondrial OS=I	AGK	47.137	21.228	6.6	3	4	3	0.878	0.831	0.992	1.048	1.071	0.969	1.126	1.139	1.19	1.19	1.33
Q9BQD7	Protein N-lysine methyltransferase FAM	FAM173A	25.13	6.9492	7.7	1	1	1	0.843	1.017	1.002	1.218	1.101	1.19	0.778	0.859	1.19	1.23	0.88
Q08378	Golgin subfamily A member 3 OS=Homo	GOLGA3	167.35	116.26	13	15	15	15	0.932	0.851	1.109	1.019	1.079	1.015	1.104	0.965	1.19	1.17	1.16
Q9HAB8	Phosphopantothenate--cysteine ligase OS	PPCS	34.005	15.695	6.8	2	3	2	0.951	0.84	1.121	1.016	1.121	0.884	1.137	1.001	1.19	1.12	1.19
P29992	Guanine nucleotide-binding protein subu	GNA11	42.123	12.699	14.2	5	6	2	0.842	0.997	1.116	1.078	1.015	0.995	1.141	0.93	1.19	1.09	1.13
Q15800	Methylsterol monooxygenase 1 OS=Hom	MSMO1	35.215	24.526	8.5	2	3	2	0.895	0.851	1.038	1.045	1.141	1.441	0.744	0.945	1.19	1.48	0.97
Q5T6V5	Queuosine salvage protein OS=Homo sa	C9orf64	39.028	46.197	17.3	6	10	6	0.94	0.906	1.102	1.1	0.981	1.11	0.861	0.918	1.19	1.13	0.96
P09104	Gamma-enolase OS=Homo sapiens OX=	ENO2	47.268	12.875	10.4	3	4	2	0.812	0.973	0.967	1.162	0.924	0.989	1.131	1.125	1.19	1.07	1.26
Q8WZA0	Protein LZIC OS=Homo sapiens OX=96	LZIC	21.494	37.506	27.9	5	6	5	0.946	0.865	1.127	1.033	1.124	1.002	1.168	0.911	1.19	1.17	1.15
Q8N488	RING1 and YY1-binding protein OS=H	RYBP	24.821	20.537	22.4	3	3	3	0.91	0.896	1.073	1.081	1.019	1.212	0.819	1.058	1.19	1.24	1.04
Q06210	Glutamine--fructose-6-phosphate aminot	GFPT1	78.806	313.19	41.1	24	33	24	0.906	0.9	1.087	1.067	0.983	0.987	1.116	1.034	1.19	1.09	1.19
Q9Y4C1	Lysine-specific demethylase 3A OS=Hon	KDM3A	147.34	61.741	7.7	9	10	9	0.938	0.832	1.057	1.054	1.064	0.928	1.046	1.14	1.19	1.13	1.24
Q15287	RNA-binding protein with serine-rich do	RNPS1	34.208	68.138	20.7	4	6	3	0.999	0.912	1.315	0.964	1.149	1.028	0.865	0.842	1.19	1.14	0.89
Q86W42	THO complex subunit 6 homolog OS=H	THOC6	37.535	132.48	26.7	5	7	5	0.91	0.925	1.195	0.993	1.056	0.985	1.048	0.945	1.19	1.11	1.09
Q9NZT2	Opioid growth factor receptor OS=Homo	OGFR	73.324	68.756	14.2	7	9	7	0.911	0.863	1.033	1.082	1.051	0.961	0.985	1.008	1.19	1.13	1.12
Q9BZE9	Tether containing UBX domain for GLU	ASPSCR1	60.182	84.438	21.7	7	8	7	0.896	0.842	1.052	1.02	1.175	0.99	1.134	1.057	1.19	1.25	1.26
Q7Z6R9	Transcription factor AP-2-delta OS=Hom	TFAP2D	49.577	6.8651	4	2	3	1	0.791	0.979	0.962	1.148	0.927	1.232	0.964	1.111	1.19	1.22	1.17
Q92610	Zinc finger protein 592 OS=Homo sapie	ZNF592	137.53	45.22	4.3	4	5	4	0.949	0.824	1.113	0.999	1.092	1.042	1.017	0.946	1.19	1.20	1.11
P56270	Myc-associated zinc finger protein OS=I	MAZ	48.607	29.412	8.8	3	4	3	0.858	0.823	0.95	1.052	1.272	1.247	0.853	1.07	1.19	1.50	1.14
Q9UBW8	COP9 signalosome complex subunit 7a C	COPS7A	30.276	7.0285	5.1	1	1	1	0.921	0.904	1.092	1.081	1.037	1.165	0.868	0.987	1.19	1.21	1.02
P10696	Alkaline phosphatase, placental-like OS=	ALPPL2	57.376	79.202	24.4	11	14	2	0.851	0.876	0.983	1.073	0.986	1.102	1.159	1.086	1.19	1.21	1.30
P16083	Ribosyldihydronicotinamide dehydrogen	NQO2	25.918	16.171	12.6	2	2	2	0.876	0.995	1.113	1.114	1.015	0.992	0.967	0.992	1.19	1.07	1.05
Q15276	Rab GTPase-binding effector protein 1 O	RABEP1	99.289	31.463	7.2	5	5	5	0.876	0.891	1.028	1.075	1.082	0.728	1.214	1.06	1.19	1.02	1.29
Q96NY8	Nectin-4 OS=Homo sapiens OX=9606 G	NECTIN4	55.454	8.4489	3.1	1	1	1	0.966	0.78	1.096	0.982	1.184	0.901	1.128	1.051	1.19	1.19	1.25
Q86XL3	Ankyrin repeat and LEM domain-contain	ANKLE2	104.11	62.574	8.3	6	7	6	0.872	0.887	0.983	1.11	1.139	1.086	1.021	0.965	1.19	1.26	1.13
P49840	Glycogen synthase kinase-3 alpha OS=H	GSK3A	50.98	38.924	23.2	7	8	4	0.929	0.913	1.128	1.063	1.036	0.928	1.044	0.912	1.19	1.07	1.06
P00558	Phosphoglycerate kinase 1 OS=Homo sa	PGK1	44.614	323.31	73.9	28	88	27	0.936	0.922	1.119	1.091	0.936	0.871	1.137	0.987	1.19	0.97	1.14
Q9NS91	E3 ubiquitin-protein ligase RAD18 OS=I	RAD18	56.222	29.916	10.7	4	4	4	0.894	0.876	1.087	1.018	1.047	0.847	1.262	1.088	1.19	1.07	1.33
Q00587	Cdc42 effector protein 1 OS=Homo sapi	CDC42EP1	40.294	15.4	6.1	2	2	2	0.899	0.916	1.079	1.079	1.033	0.973	1.103	0.988	1.19	1.11	1.15
Q9UK59	Lariat debranching enzyme OS=Homo sa	DBR1	61.554	19.376	5.9	3	3	3	0.881	0.86	1.084	0.986	1.15	0.909	1.291	0.985	1.19	1.18	1.31
Q6ZMZ3	Nesprin-3 OS=Homo sapiens OX=9606	SYNE3	112.22	18.112	3	3	4	3	0.547	0.973	0.886	0.921	1.948	1.111	0.787	1.296	1.19	2.01	1.37
P25205	DNA replication licensing factor MCM3	MCM3	90.98	308.11	37.9	25	36	25	0.874	0.859	1.041	1.019	1.125	1.031	1.104	1.066	1.19	1.24	1.25
Q9NXH9	tRNA (guanine(26)-N(2))-dimethyltransf	TRMT1	72.233	18.916	6.8	3	3	3	0.806	0.882	1.007	0.999	1.142	1.038	1.071	1.27	1.19	1.29	1.39
O94919	Endonuclease domain-containing 1 prote	ENDOD1	55.016	39.876	11	4	4	4	0.854	0.82	1.001	0.988	1.155	1.103	1.1	0.989	1.19	1.35	1.25
P56962	Syntaxin-17 OS=Homo sapiens OX=960	STX17	33.403	19.753	10.9	3	4	3	0.901	0.913	1	1.155	1.002	1.011	0.987	1.047	1.19	1.11	1.12
Q13620	Cullin-4B OS=Homo sapiens OX=9606	CUL4B	103.98	155.83	27.1	21	28	14	0.943	0.893	1.083	1.098	0.992	0.997	1.036	0.962	1.19	1.08	1.09
Q8WXA9	Splicing regulatory glutamine/lysine-rich	SREK1	59.38	52.823	11.6	4	5	3	0.944	0.906	1.137	1.06	1.097	0.99	1.032	0.966	1.19	1.13	1.08
Q9UBV8	Peflin OS=Homo sapiens OX=9606 GN=	PEF1	30.381	18.655	8.5	2	3	2	0.919	0.815	1.048	1.011	1.144	0.858	1.218	1.02	1.19	1.15	1.29
Q9P206	Uncharacterized protein KIAA1522 OS=I	KIAA1522	107.09	10.433	2.8	2	1	1	0.946	0.894	1.074	1.11	0.962	0.853	1.189	0.978	1.19	0.99	1.18
Q9NXJ5	Pyroglutamyl-peptidase 1 OS=Homo sapi	PGPEP1	23.138	6.3988	3.8	1	1	1	0.884	0.85	1.033	1.025	1.136	0.938	1.169	1.088	1.19	1.20	1.30
Q8N129	Protein canopy homolog 4 OS=Homo sa	CNPY4	28.309	14.356	9.7	2	2	2	0.85	0.879	0.937	1.115	1.059	1.032	1.115	1.096	1.19	1.21	1.28
O75071	EF-hand calcium-binding domain-contai	EFCAB14	55.031	10.892	3.4	1	2	1	0.972	0.832	1.081	1.06	1.039	0.986	1.116	0.938	1.19	1.12	1.14
Q9BV57	1,2-dihydroxy-3-keto-5-methylthiopente	ADI1	21.498	48.01	49.2	7	10	7	0.845	0.839	1.01	0.988	1.117	1.172	1.032	1.121	1.19	1.36	1.28
Q8IZE3	Protein-associating with the carboxyl-ter	SCYL3	82.857	17.502	6.6	3	3	3	1.029	0.855	1.257	0.978	1.102	1.145	0.809	0.868	1.19	1.19	0.89
Q9UMX0	Ubiquilin-1 OS=Homo sapiens OX=960	UBQLN1	62.518	87.147	17	6	7	3	1.133	0.716	1.181	1.012	0.926	1.061	0.706	1.223	1.19	1.07	1.04
Q9ULV3	Cip1-interacting zinc finger protein OS=I	CIZ1	100.04	20.333	4	2	2	2	0.959	0.855	1.155	0.996	1.001	1.282	0.851	0.992	1.19	1.26	1.02
Q86VR2	Reticulophagy regulator 3 OS=Homo sap	RETREG3	51.396	29.768	12	4	5	4	0.921	0.879	1.039	1.095	1.12	0.862	1.212	1.031	1.19	1.10	1.25

O95747	Serine/threonine-protein kinase OSR1 O:	OXSR1	58.022	108.6	24.5	11	16	11	0.921	0.924	1.107	1.08	1.013	0.914	1.026	1.018	1.19	1.04	1.11
Q9NQT8	Kinesin-like protein KIF13B OS=Homo :	KIF13B	202.79	12.94	1.7	3	3	2	0.956	0.882	1.048	1.13	0.976	0.92	1.114	0.959	1.18	1.03	1.13
Q9BUJ2	Heterogeneous nuclear ribonucleoprotein	HNRNPUL	95.737	80.125	15.4	10	13	10	0.952	0.846	1.093	1.037	1.095	0.953	1.07	1.017	1.18	1.14	1.16
Q13188	Serine/threonine-protein kinase 3 OS=Hc	STK3	56.3	35.251	17.1	8	13	5	0.979	0.82	1.038	1.093	1.036	0.83	1.158	1.05	1.18	1.04	1.23
Q8TEQ8	GPI ethanolamine phosphate transferase :	PIGO	118.7	7.3187	1	1	1	1	0.916	0.915	1.072	1.096	1.061	0.955	1.033	0.996	1.18	1.10	1.11
Q99959	Plakophilin-2 OS=Homo sapiens OX=96	PKP2	97.414	165.51	27.2	19	22	18	0.898	1.011	1.175	1.085	0.805	0.818	1.178	0.964	1.18	0.85	1.12
Q9NZC9	SWI/SNF-related matrix-associated actin	SMARCAL	105.94	26.683	5.7	4	4	4	0.875	1.003	1.191	1.032	0.776	1.143	1.073	1.04	1.18	1.02	1.13
P31150	Rab GDP dissociation inhibitor alpha OS	GDI1	50.582	64.048	30.9	12	26	7	0.943	0.903	1.109	1.076	0.982	0.791	1.17	0.995	1.18	0.96	1.17
O43715	TP53-regulated inhibitor of apoptosis 1 (	TRIAP1	8.7858	29.85	48.7	4	4	4	0.878	0.936	1.096	1.051	1.092	0.949	1.104	1	1.18	1.13	1.16
Q8IWR1	Tripartite motif-containing protein 59 OS	TRIM59	47.113	15.563	8.7	2	2	2	0.837	0.856	1.046	0.957	1.052	1.009	1.366	1.088	1.18	1.22	1.45
Q8N668	COMM domain-containing protein 1 OS	COMMD1	21.178	7.637	7.4	1	1	1	0.906	0.955	1.12	1.081	1.067	1.035	1.044	0.848	1.18	1.13	1.02
Q8TBX8	Phosphatidylinositol 5-phosphate 4-kinas	PIP4K2C	47.299	32.329	5.7	2	2	2	0.904	0.953	1.105	1.091	1.132	0.734	1.201	0.926	1.18	1.00	1.15
O75676	Ribosomal protein S6 kinase alpha-4 OS	RPS6KA4	85.605	155.56	23.6	17	19	17	0.906	0.908	1.081	1.062	1.029	1.095	0.959	1.002	1.18	1.17	1.08
O75879	Glutamyl-tRNA(Gln) amidotransferase su	GATB	61.863	12.13	3.1	2	2	2	0.914	0.947	1.179	1.019	1.083	1.181	0.926	0.851	1.18	1.22	0.95
P63027	Vesicle-associated membrane protein 2 C	VAMP2	12.663	7.8561	34.5	3	7	1	1.035	0.796	1.086	1.076	0.932	0.964	1.123	0.953	1.18	1.04	1.13
O15533	Tapasin OS=Homo sapiens OX=9606 GN	TAPBP	47.625	19.146	7.4	3	5	3	0.823	1.006	1.111	1.048	1	1.351	0.818	1.007	1.18	1.29	1.00
Q9BYD2	39S ribosomal protein L9, mitochondrial	MRPL9	30.243	61.421	34.5	8	8	8	1.004	0.671	1.004	0.972	1.114	1.254	0.798	1.017	1.18	1.41	1.08
P35573	Glycogen debranching enzyme OS=Homo	AGL	174.76	164.73	15.9	21	25	20	0.947	0.841	1.064	1.045	1.143	1.094	0.994	0.961	1.18	1.25	1.09
P49715	CCAAT/enhancer-binding protein alpha (	CEBPA	37.561	19.992	8.1	3	2	2	0.926	0.932	1.056	1.135	0.913	0.907	1.126	1.013	1.18	0.98	1.15
Q9NRR5	Ubiquilin-4 OS=Homo sapiens OX=960(	UBQLN4	63.852	33.119	11.1	5	7	4	0.979	0.886	1.185	1.014	1.157	0.993	1.054	0.883	1.18	1.15	1.04
O14763	Tumor necrosis factor receptor superfami	TNFRSF1C	47.878	6.551	2	1	1	1	0.856	0.885	0.968	1.084	1.135	1.031	0.974	1.174	1.18	1.24	1.23
P60174	Triosephosphate isomerase OS=Homo sa	TPI1	30.791	172.72	53.8	11	29	11	0.907	0.919	1.085	1.067	1.051	0.979	1.056	0.976	1.18	1.11	1.11
Q9HB90	Ras-related GTP-binding protein C OS=I	RRAGC	44.223	69.004	27.1	7	9	5	0.91	0.851	1.07	1.005	1.039	0.96	1.125	1.112	1.18	1.14	1.27
Q8N5F7	NF-kappa-B-activating protein OS=Homo	NKAP	47.138	14.648	6.3	2	3	2	0.934	0.844	1.012	1.083	1.153	0.861	1.162	0.982	1.18	1.13	1.21
Q96NB3	Zinc finger protein 830 OS=Homo sapien	ZNF830	41.998	14.235	8.6	2	2	2	0.924	0.861	1.111	0.992	1.097	0.905	1.18	1.044	1.18	1.12	1.25
Q8WYQ5	Microprocessor complex subunit DGCR8	DGCR8	86.044	17.224	3.9	2	2	2	0.979	0.964	1.251	1.038	1.149	1.017	0.924	0.705	1.18	1.11	0.84
P62304	Small nuclear ribonucleoprotein E OS=H	SNRPE	10.803	18.315	25	2	1	1	0.903	0.993	1.19	1.042	1.081	1.111	0.89	0.884	1.18	1.16	0.94
O15084	Serine/threonine-protein phosphatase 6 re	ANKRD28	112.96	55.412	5.9	4	5	4	0.9	0.902	1.05	1.071	1.053	0.978	1.186	0.931	1.18	1.13	1.17
P55209	Nucleosome assembly protein 1-like 1 O:	NAP1L1	45.374	52.827	14.6	4	8	4	0.973	0.798	1.016	1.068	1.082	1.152	0.98	1.037	1.18	1.26	1.14
Q8N9N5	Protein BANP OS=Homo sapiens OX=9	BANP	56.494	13.156	4.8	2	2	2	0.795	1.024	1.024	1.116	1.068	0.914	1.072	1.118	1.18	1.09	1.20
P10398	Serine/threonine-protein kinase A-Raf O:	ARAF	67.585	14.282	6.1	3	3	2	0.941	0.89	1.118	1.035	1.073	0.902	1.17	0.935	1.18	1.08	1.15
Q96HW7	Integrator complex subunit 4 OS=Homo :	INTS4	108.17	33.368	6.7	6	6	6	0.946	0.903	1.036	1.138	1.116	1.108	0.866	0.957	1.18	1.20	0.99
Q13126	S-methyl-5'-thioadenosine phosphorylase	MTAP	31.236	81.709	50.5	9	13	9	0.899	0.91	1.098	1.028	0.969	0.958	1.123	1.058	1.18	1.07	1.21
P98196	Probable phospholipid-transporting ATP	ATP11A	129.75	12.615	2.5	3	3	2	0.99	1.026	1.377	0.992	0.977	1.267	0.832	0.594	1.18	1.11	0.71
P56937	3-keto-steroid reductase OS=Homo sapie	HSD17B7	38.206	12.393	6.5	2	2	2	0.861	1.075	1.166	1.108	0.875	1.333	0.782	0.886	1.17	1.14	0.86
Q14566	DNA replication licensing factor MCM6	MCM6	92.888	159.83	28.1	20	30	20	0.912	0.866	1.058	1.03	1.065	0.945	1.147	1.051	1.17	1.13	1.24
C4AMC7	Putative WAS protein family homolog 3 (	WASH3P	49.994	26.423	8.9	4	8	4	0.917	0.908	1.115	1.028	1.021	1.102	0.939	1.038	1.17	1.16	1.08
B5MCY1	Tudor domain-containing protein 15 OS=	TDRD15	221.72	11.146	1.1	2	2	2	0.911	0.915	1.066	1.078	1.055	1.005	1.097	0.93	1.17	1.13	1.11
Q9Y617	Phosphoserine aminotransferase OS=Hor	PSAT1	40.422	98.615	38.6	12	16	11	0.893	0.882	1.025	1.059	1.001	0.986	1.133	1.136	1.17	1.12	1.28
Q13546	Receptor-interacting serine/threonine-pro	RIPK1	75.93	52.776	14.5	7	8	7	0.869	0.895	1.014	1.057	1.058	1.277	0.948	1.107	1.17	1.32	1.16
Q8WW01	tRNA-splicing endonuclease subunit Sen	TSEN15	18.641	8.1258	13.5	1	2	1	0.957	0.905	1.115	1.071	0.996	1.004	1.057	0.924	1.17	1.07	1.06
O00469	Procollagen-lysine,2-oxoglutarate 5-diox	PLOD2	84.685	103.83	19.4	11	18	11	0.925	0.916	1.07	1.091	0.978	0.923	1.052	1.069	1.17	1.03	1.15
Q8NBJ5	Procollagen galactosyltransferase 1 OS=I	COLGALT	71.635	99.749	27.5	15	16	14	0.932	0.819	1.037	1.018	1.079	0.912	1.201	1.094	1.17	1.14	1.31
A4D1P6	WD repeat-containing protein 91 OS=Ho	WDR91	83.343	7.5618	3.3	1	1	1	0.821	1.013	1.093	1.059	1.049	1.234	0.892	0.989	1.17	1.24	1.03
Q8N3F8	MICAL-like protein 1 OS=Homo sapiens	MICALL1	93.44	161.99	27.6	16	18	16	0.906	0.895	1.031	1.082	1.02	0.933	1.11	1.093	1.17	1.08	1.22
Q8WUF5	RelA-associated inhibitor OS=Homo sap	PPP1R13L	89.09	124.93	27.9	16	17	16	0.98	0.816	1.07	1.037	1.108	0.918	1.046	0.997	1.17	1.13	1.14
P04406	Glyceraldehyde-3-phosphate dehydrogena	GAPDH	36.053	298.9	60.6	17	112	16	0.928	0.956	1.129	1.081	0.979	1.054	0.951	0.947	1.17	1.08	1.01
Q99543	DnaJ homolog subfamily C member 2 OS	DNAJC2	71.996	69.574	15.3	8	11	8	0.932	0.885	1.107	1.023	1.109	1.159	0.916	0.974	1.17	1.25	1.04
Q9BU64	Centromere protein O OS=Homo sapiens	CENPO	33.785	16.921	8	2	2	2	0.826	0.841	0.984	0.97	0.986	1.032	1.371	1.202	1.17	1.21	1.54
Q15773	Myeloid leukemia factor 2 OS=Homo sap	MLF2	28.147	33.465	15.3	3	4	3	0.899	0.845	1.026	1.018	1.076	1.148	1.001	1.111	1.17	1.28	1.21
Q86VE9	Serine incorporator 5 OS=Homo sapiens	SERINC5	47.009	12.978	4.5	2	2	2	0.864	0.825	1.002	0.977	1.25	1.176	0.959	1.124	1.17	1.44	1.23
P27361	Mitogen-activated protein kinase 3 OS=I	MAPK3	43.135	66.804	22.2	7	10	6	0.913	0.841	1.004	1.051	1.07	0.83	1.19	1.013	1.17	1.08	1.26
Q8N4C8	Misshapen-like kinase 1 OS=Homo sapie	MINK1	149.82	57.123	6.3	8	8	6	0.925	0.931	1.133	1.041	1.086	1.007	1.114	1.021	1.17	1.13	1.15
Q9BYG5	Partitioning defective 6 homolog beta OS	PARD6B	41.182	36.279	18.3	5	8	5	0.921	0.807	1.112	0.912	1.244	1.006	1.127	0.991	1.17	1.30	1.23
O94766	Galactosylgalactosylxylosylprotein 3-beta	B3GAT3	37.121	10.955	5.7	2	2	2	0.9	0.84	0.987	1.051	1.163	1.047	1.042	1.06	1.17	1.27	1.21
P04075	Fructose-bisphosphate aldolase A OS=H	ALDOA	39.42	323.31	64.6	21	70	18	0.913	0.964	1.122	1.076	0.994	1.036	0.98	0.932	1.17	1.08	1.02
O14802	DNA-directed RNA polymerase III subun	POLR3A	155.64	24.963	3	4	4	4	0.872	0.947	1.026	1.104	0.96	1.244	0.992	0.929	1.17	1.21	1.06
O95630	STAM-binding protein OS=Homo sapien	STAMBP	48.076	28.858	8.7	3	3	3	1.033	0.729	0.965	1.098	1.113	0.824	1.103	1.083	1.17	1.10	1.24
O60664	Perilipin-3 OS=Homo sapiens OX=9606	PLIN3	47.074	104	31.6	10	15	10	0.939	0.984	1.185	1.066	0.998	0.929	1.063	0.952	1.17	1.00	1.05
Q14241	Elongin-A OS=Homo sapiens OX=9606	ELOA	89.908	68.891	8.5	7	8	7	0.92	0.845	1.044	1.022	1.138	0.992	1.079	1.142	1.17	1.21	1.26
Q9H3U5	Major facilitator superfamily domain-con	MFSD1	51.208	10.188	2.6	1	1	1	0.898	0.973	1.12	1.07	1.03	1.224	0.871	0.894	1.17	1.20	0.94
Q99571	P2X purinoceptor 4 OS=Homo sapiens C	P2RX4	43.369	13.577	3.4	1	1	1	0.919	0.984	1.106	1.121	0.967	0.953	1.015	0.959	1.17	1.01	1.04
Q9H8Y5	Ankyrin repeat and zinc finger domain-cc	ANKZF1	80.926	114.5	20.8	11	13	11	0.94	0.952	1.168	1.045	0.968	0.954	1.107	0.961	1.17	1.02	1.09
Q9BVV7	Mitochondrial import inner membrane tra	TIMM21	28.202	10.978	7.3	2	2	2	0.955	0.779	0.932	1.096	1.151	1.018	0.982	1.102	1.17	1.25	1.20
Q99856	AT-rich interactive domain-containing pr	ARID3A	62.888	106.36	28	14	24	13	0.926	0.885	1.071	1.047	1.027	0.953	1.084	1.059	1.17	1.09	1.18
Q9HBR0	Putative sodium-coupled neutral amino a	SLC38A10	119.76	99.052	14.3	14	17	14	0.932	0.921	1.086	1.081	1.039	0.993	1.075	1.047	1.17	1.10	1.15
Q96R06	Sperm-associated antigen 5 OS=Homo sa	SPAG5	134.42	20.765	2.9	3	3	3	0.927	0.924	1.114	1.05	0.989	0.938	1.139	0.988	1.17	1.04	1.15
P13489	Ribonuclease inhibitor OS=Homo sapien	RNH1	49.973	214.44	43.2	15	22	15	0.897	0.861	1.014	1.041	0.996	0.931	1.205	0.999	1.17	1.10	1.25
P62070	Ras-related protein R-Ras2 OS=Homo sa	RRAS2	23.399	32.485	16.7	3	5	3	0.911	0.93	1.058	1.094	1.039	0.953	1.046	1.042	1.17	1.08	1.13
O00148	ATP-dependent RNA helicase DDX39A	DDX39A	49.129	43.447	30.7	11	21	4	0.966	0.888	1.089	1.078	1.034	0.928	1.047	0.99	1.17	1.06	1.10
Q7Z417	Nuclear fragile X mental retardation-inter	NUFIP2	76.12	160.39	32.4	16	24	16	0.955	0.894	1.101	1.06	0.998	1.006	1.009	0.966	1.17	1.08	1.07
Q14344	Guanine nucleotide-binding protein subu	GNA13	44.049	77.816	24.4	9	11	8	0.891	0.804	0.958	1.023	1.182	1.095	1.042	1.079	1.17	1.34	1.25
Q9UHD2	Serine/threonine-protein kinase TBK1 O:	TBK1	83.641	61.664	13.2	8	9	8	0.957	0.887	1.117	1.038	1.175	0.932	1.119	0.863	1.17	1.14	1.07
Q8N4S9	MARVEL domain-containing protein 2 (	MARVELI	64.167	18.189	4.1	2	3	2	0.934	0.843	1.042	1.034	1.166	1.011	1.043	1.002	1.17	1.23	1.15
Q8N6R0	Methyltransferase-like protein 13 OS=Hc	METTL13	78.767	62.957	10.9	5	7	5	0.956	0.845	1.104	1	1.149	0.986	1.059	0.885	1.17	1.19	1.08
Q8IXJ6	NAD-dependent protein deacetylase sirtu	SIRT2	43.182	19.617	6.4	2	2	2	0.932	0.834	0.99	1.073	1.125	0.897	1.19	1	1.17	1.14	1.24
O75410	Transforming acidic coiled-coil-containir	TACC1	87.793	78.27	15.8	9	10	8	0.948	0.872	1.1	1.026	0.961	1.021	1.174	0.995	1.17	1.09	1.19
P17480	Nucleolar transcription factor 1 OS=Hom	UBTF	89.405	125.79	16.9	12	17	12	0.91	0.91	1.083	1.043	1.039	0.997	1.104	1.013	1.17	1.12	1.16
Q9GZT6	Coiled-coil domain-containing protein 9(	CCDC90B	29.505	12.523	7.9	2	2	2	0.905	0.985	1.191	1.016	0.995	1.201	0.906	0.919	1.17	1.16	0.97
A6NCS6	Uncharacterized protein C2orf72 OS=Ho	C2orf72	30.48	10.481	8.1	1	1	1	0.949	0.804	1.043	1.004	1.212	0.884	1.249	0.928	1.17	1.20	1.24
Q12893	Transmembrane protein 115 OS=Homo s	TMEM115	38.197	10.961	7.7	2	2	2	1.028	1.074	1.61	0.844	1.179	0.595	1.258	0.53	1.17	0.84	0.85
Q9Y3D0	Mitotic spindle-associated MMXD comp	FAM96B	17.663	7.5165	12.9	1	1	1	0.853	0.958	0.956	1.158	1.035	0.841	1.09	1.161	1.17	1.04	1.24
Q9NVJ2	ADP-ribosylation factor-like protein 8B (	ARL8B	21.539	37.423	18.8	4	7	3	0.923	0.906	1.14	0.995	1.083	1.017	1.125	0.969	1.17	1.15	1.14
Q6P2C8	Mediator of RNA polymerase II transcrip	MED27	35.431	21.835	9.6	3	4	3	0.958	0.798	1.104	0.945	1.176	1.129	1.006	1.008	1.17	1.31	1.15
Q96S59	Ran-binding protein 9 OS=Homo sapiens	RANBP9	77.846	40.169	8.1	5	6	5	0.916	0.907	1.08	1.047	1.129	0.96	1.071	0.978	1.17	1.15	1.12
Q9UN37	Vacuolar protein sorting-associated prote	VPS4A	48.897	49.814	19.7	7	10	5	0.922	0.873	1.088	1.006	1.02	0.989	1.104	1.042	1.17	1.12	1.20
Q9NSV4	Protein diaphanous homolog 3 OS=Homo	DIAPH3	136.92	101.8	14.1	13	14	13	0.835	0.807	0.883	1.032	1.05	1.046	1.257	1.186	1.17	1.28	1.49
Q9NP58	ATP-binding cassette sub-family B memb	ABCB6	93.884	109.15	14.5	9	12	9	0.947	0.861	1.083	1.025	1.119	1.013	1.142	0.954	1.17	1.18	1.16
Q9BQA1	Methylosome protein 50 OS=Homo sapie	WDR77	36.724	107.51	22.8	6	9	6	0.94	1.032	1.231	1.068	0.952	0.997	0.884	0.895	1.17	0.99	0.90
Q8WZ82	Esterase OVCA2 OS=Homo sapiens OX	OVCA2	24.418	23.615	16.3	2	3	2	0.947	0.936	1.131	1.064	0.994	1.038	1.004	0.931	1.17	1.08	1.03
Q15942	Zyxin OS=Homo sapiens OX=9606 GN=	ZYX	61.277	70.447	19.8	7	9	7	0.914	0.939	1.103	1.057	1.003	0.934	1.136	1.024	1.17	1.05	1.17
Q9H330	Transmembrane protein 245 OS=Homo s	TMEM245	97.356	41.616	9.7	6	7	6	0.898	1	1.112	1.1	0.993	0.939	1.132	0.981	1.17	1.02	1.11
A4UGR9	Xin actin-binding repeat-containing prote	XIRP2	382.3	11.087	0.5	2	3	2	0.867	0.983	1.059	1.097	1.029	0.788	1.155	1.108	1.17	0.98	1.22
Q8NEU8	DCC-interacting protein 13-beta OS=Ho	APPL2	74.493	15.813	4.2	2	2	2	0.856	0.995	1.08	1.077	0.973	1.085	1.049	0.994	1.17	1.11	1.10
O15027	Protein transport protein Sec16A OS=Ho	SEC16A	233.51	127.32	10.2	17	18	17	0.939	0.876	1.072	1.043	1.052	1.022	0.988	0.974	1.17	1.14	1.08
P42331	Rho GTPase-activating protein 25 OS=H	ARHGAP2	73.434	5.9911	2.2	1	1	1	0.975	0.822	0.976	1.118	1.136	0.933	0.948	1.076	1.17	1.15	1.13
P0DMV9	Heat shock 70 kDa protein 1B OS=Homo	HSPA1B	70.051	165.93	34.6	17	43	9	0.917	0.798	0.958	1.04	1.156	0.922	1.202	1.094	1.17	1.21	1.34
Q9UIJ7	GTP:AMP phosphotransferase AK3, mitc	AK3	25.565	105.25	43.2	9	11	9	0.897	0.873	0.989	1.073	1.023	1.065	1.036	1.096	1.16	1.18	1.20
P13674	Prolyl 4-hydroxylase subunit alpha-1 OS	P4HA1	61.049	86.779	25.3	12	15	12	0.933	0.905	1.057	1.084	0.9	0.847	1.184	1.116	1.16	0.95	1.25
P23786	Carnitine O-palmitoyltransferase 2, mitoc	CPT2	73.776	100.57	27.2	14	16	14	0.937	0.932	1.115	1.062	1.017	1.05	0.859	0.933	1.16	1.11	0.96
Q9Y281	Cofilin-2 OS=Homo sapiens OX=9606 (	CFL2	18.736	35.084	47.6	6	9	5	0.926	0.841	0.985	1.073	1.101	1.008	1.075	1.017	1.16	1.19	1.18
O95248	Myotubularin-related protein 5 OS=Hom	SBF1	208.44	55.179	4.8	7	8	7	0.927	0.817	0.989	1.042	1.044	0.886	1.197	1.169	1.16	1.11	1.36
Q9UQN3	Charged multivesicular body protein 2b (	CHMP2B	23.906	14.1	8.9	2	4	2	0.955	0.863	1.069	1.047	1.022	1.222	0.907	0.969	1.16	1.23	1.03
Q5VT52	Regulation of nuclear pre-mRNA domain	RPRD2	156.02	73.354	8.2	9	11	9	0.904	0.953	1.107	1.054	1.069	1.099	0.891	0.988	1.16	1.17	1.01
Q15293	Reticulocalbin-1 OS=Homo sapiens OX=	RCN1	38.89	101.04	40.8	11	30	11	0.943	0.872	1.087	1.025	1.072	1.022	1.003	1.068	1.16	1.15	1.14
Q9NZN8	CCR4-NOT transcription complex subun	CNOT2	59.737	21.031	4.8	2	3	2	0.939	0.957	1.16	1.046	1.134	1.052	0.861	0.915	1.16	1.15	0.94
O75886	Signal transducing adapter molecule 2 O:	STAM2	58.164	31.845	9	4	5	4	0.918	0.951	1.071	1.103	0.962	1.165	0.927	0.907	1.16	1.14	0.98
P07686	Beta-hexosaminidase subunit beta OS=H	HEXB	63.111	106.93	22.7	12	17	12	0.901	0.896	1.019	1.071	1.042	1.255	0.934	1.038	1.16	1.28	1.10
P62314	Small nuclear ribonucleoprotein Sm D1 (	SNRPD1	13.281	14.461	10.9	2	4	2	0.999	0.78	1.082	0.987	1.256	0.885	1.039	1.028	1.16	1.20	1.16
Q6PCB5	Round spermatid basic protein 1-like pro	RSBN1L	94.868	34.533	6.4	4	4	4	0.967	0.967	1.175	1.074	1.082	1.012	0.958	0.776	1.16	1.08	0.90
Q13616	Cullin-1 OS=Homo sapiens OX=9606 G	CUL1	89.677	168.43	22.7	15	18	15	0.941	0.848	1.062	1.018	1.182	0.915	1.127	1.016	1.16	1.17	1.20
Q92734	Protein TFG OS=Homo sapiens OX=960	TFG	43.447	142.28	31.2	9	16	9	0.919	0.87	1.038	1.042	1.073	0.97	1.067	1.121	1.16	1.14	1.22
Q15361	Transcription termination factor 1 OS=H	TTF1	103.05	16.401	4.9	2	2	2	0.899	0.929	1.097	1.028	1.094	1.072	1.046	0.943	1.16	1.18	1.09
P50748	Kinetochore-associated protein 1 OS=Ho	KNTC1	250.75	53.439	3.3	7	9	7	0.937	0.863	1.073	1.019	1.155	0.845	1.204	0.998	1.16	1.11	1.22
Q13409	Cytoplasmic dynein 1 intermediate chain	DYNC1I2	71.456	67.187	10.8	6	7	6	0.929	0.948	1.096	1.085	0.962	0.989	0.972	1.017	1.16	1.04	1.06
O60231	Pre-mRNA-splicing factor ATP-depender	DHX16	119.26	66.197	9.6	9	13	8	0.988	0.848	1.065	1.068	1.019	1.001	1.103	1.046	1.16	1.10	1.17
P31943	Heterogeneous nuclear ribonucleoprotein	HNRNPH1	49.229	308.23	50.8	16	44	8	0.923	0.926	1.092	1.056	1.001	1.182	0.906	0.928	1.16	1.18	0.99
Q9Y282	Endoplasmic reticulum-Golgi intermediat	ERGIC3	43.222	28.932	10.2	4	5	4	0.977	0.799	0.976	1.087	1.1	0.807	1.126	1.026	1.16	1.07	1.21
Q9Y2L1	Exosome complex exonuclease RRP44 C	DIS3	109	178.9	25.6	21	29	21	0.879	0.917	1.053	1.033	1.062	0.999	1.081	1.049	1.16	1.15	1.19
P46976	Glycogenin-1 OS=Homo sapiens OX=96	GYG1	39.383	20.693	12.3	3	6	3	0.925	0.865	1.039	1.04	1.022	1.04	1.024	0.982	1.16	1.15	1.12
O95197	Reticulon-3 OS=Homo sapiens OX=960(	RTN3	112.61	9.7399	1.1	1	2	1	1.035	0.786	1.084	1.03	1.165	0.629	1.204	1.061	1.16	0.99	1.24
Q9NZL9	Methionine adenosyltransferase 2 subuni	MAT2B	37.551	63.47	24	7	11	7	0.927	0.947	1.079	1.096	1.017	1.026	1.063	0.968	1.16	1.09	1.08
Q9BRX9	WD repeat domain-containing protein 83	WDR83	34.342	14.971	8.3	2	2	2	0.916	0.859	1.034	1.026	1.098	1.226	0.885	1.058	1.16	1.31	1.09
O95926	Pre-mRNA-splicing factor SYF2 OS=Ho	SYF2	28.722	20.434	12.3	3	3	3	0.91	0.911	1.039	1.074	0.997	0.901	1.202	1.031	1.16	1.04	1.23
O15427	Monocarboxylate transporter 4 OS=Hom	SLC16A3	49.469	20.206	7.3	3	6	3	0.91	0.874	1.022	1.048	0.918	1.243	0.881	1.206	1.16	1.21	1.17
Q9Y3P9	Rab GTPase-activating protein 1 OS=Ho	RABGAP1	121.74	116.97	14.6	13	16	11	0.941	0.893	1.088	1.04	1.003	0.965	1.105	1.014	1.16	1.07	1.16
P14209	CD99 antigen OS=Homo sapiens OX=9(	CD99	18.848	16.544	15.1	2	3	2	0.908	0.939	1.005	1.138	0.881	0.853	1.387	0.897	1.16	0.94	1.24
P36639	7,8-dihydro-8-oxoguanine triphosphatase	NUDT1	22.519	7.7735	6.6	1	1	1	0.861	0.919	0.988	1.077	1.052	1.271	0.778	1.171	1.16	1.31	1.09
Q9NZJ7	Mitochondrial carrier homolog 1 OS=Ho	MTCH1	41.544	31.371	10.8	4	5	4	0.979	0.931	1.186	1.029	0.953	0.923	1.154	0.946	1.16	0.98	1.10
Q9GZS1	DNA-directed RNA polymerase I subunit	POLR1E	53.961	83.874	24.5	11	15	11	0.936	0.85	1.078	0.993	1.172	1.045	1.024	1.024	1.16	1.24	1.15
Q13444	Disintegrin and metalloproteinase domair	ADAM15	92.958	62.979	11.9	8	8	8	0.909	0.895	1.056	1.035	1.084	1.215	0.91	0.993	1.16	1.27	1.05
Q8WY22	BRI3-binding protein OS=Homo sapiens	BRI3BP	27.835	21.484	7.6	1	2	1	0.871	0.919	1.03	1.044	1.081	1.168	0.973	1.036	1.16	1.26	1.12
O43823	A-kinase anchor protein 8 OS=Homo sap	AKAP8	76.107	57.913	10.7	7	10	7	0.91	0.899	1.074	1.022	1.099	1.032	1.019	0.978	1.16	1.18	1.10
Q9H2C2	Protein ARV1 OS=Homo sapiens OX=9(	ARV1	31.052	9.5946	7.4	1	1	1	0.741	1.125	1.169	0.993	1.031	1.311	0.919	0.978	1.16	1.26	1.02
Q9NX08	COMM domain-containing protein 8 OS	COMMD8	21.09	14.464	14.8	2	2	2	0.953	0.84	1.089	0.988	1.256	1.187	0.74	1.049	1.16	1.36	1.00
Q9UBB6	Neurochondrin OS=Homo sapiens OX=9	NCDN	78.863	13.035	3.2	2	2	2	1.055	0.789	1.139	0.997	1.298	1.141	0.517	1.083	1.16	1.32	0.87
Q9NPJ6	Mediator of RNA polymerase II transcrip	MED4	29.745	9.9684	6.3	1	1	1	0.88	1.028	1.248	0.962	1.169	0.865	0.828	1.208	1.16	1.07	1.07
Q04837	Single-stranded DNA-binding protein, m	SSBP1	17.259	42.443	39.2	5	11	5	0.881	0.919	1.039	1.045	1.052	1.083	1.023	1.123	1.16	1.19	1.19
O75438	NADH dehydrogenase [ubiquinone] 1 be	NDUFB1	6.9611	11.113	19	1	2	1	0.912	0.939	1.138	1.005	1.065	1.069	1.032	0.954	1.16	1.15	1.07
Q8WVV9	Heterogeneous nuclear ribonucleoprotein	HNRNPLL	60.082	85.375	21.6	8	8	8	0.939	0.901	1.054	1.076	1.018	1.006	1.089	0.954	1.16	1.10	1.11
Q13363	C-terminal-binding protein 1 OS=Homo :	CTBP1	47.535	8.3579	8.6	3	5	1	0.889	1.063	1.151	1.108	0.98	1.316	0.724	0.828	1.16	1.18	0.80
Q96G46	tRNA-dihydrouridine(47) synthase [NAD	DUS3L	72.593	29.838	6.3	4	4	4	0.945	0.9	1.077	1.058	1.076	1.044	0.93	1.023	1.16	1.15	1.06
Q9NX40	OCIA domain-containing protein 1 OS=I	OCIAD1	27.626	53.195	24.5	5	6	5	0.963	0.93	1.144	1.046	1.127	0.833	1.151	0.963	1.16	1.04	1.12
Q9NVE5	Ubiquitin carboxyl-terminal hydrolase 40	USP40	140.13	7.3048	1.3	1	1	1	0.956	0.928	1.098	1.081	1.011	0.871	1.175	0.897	1.16	1.00	1.10
Q8NBW4	Sodium-coupled neutral amino acid trans	SLC38A9	63.775	23.721	4.6	1	2	1	0.921	0.912	1.102	1.018	1.066	1.346	0.76	0.983	1.16	1.32	0.95
Q9UGP4	LIM domain-containing protein 1 OS=Hc	LIMD1	72.189	60.803	13.9	6	7	6	0.866	0.929	1.008	1.068	1.04	1.03	1.031	1.074	1.16	1.15	1.17
Q5VT66	Mitochondrial amidoxime-reducing comp	MARC1	37.499	15.655	8	2	2	2	0.913	0.882	1.059	1.017	1.057	1.076	1.113	0.986	1.16	1.19	1.17
Q9NP92	39S ribosomal protein S30, mitochondria	MRPS30	50.364	32.861	11.4	3	3	3	0.952	0.856	1.036	1.055	0.979	1.172	0.83	0.927	1.16	1.19	0.97
O75347	Tubulin-specific chaperone A OS=Homo	TBCA	12.855	59.142	25.9	3	4	3	0.958	0.946	1.073	1.129	0.933	0.913	1.072	0.987	1.16	0.97	1.08
P55795	Heterogeneous nuclear ribonucleoprotein	HNRNPH2	49.263	69.918	37.2	12	20	5	0.929	0.899	1.091	1.023	1.07	0.968	1.095	1.016	1.16	1.11	1.15
Q9NX46	Poly(ADP-ribose) glycohydrolase ARH3	ADPRHL2	38.946	28.264	8.3	2	3	2	0.947	0.882	1.087	1.028	1.159	0.814	1.196	0.95	1.16	1.08	1.17
Q15628	Tumor necrosis factor receptor type 1-ass	TRADD	34.247	9.6482	6.4	1	1	1	0.901	0.992	1.15	1.039	1.092	1.305	0.723	0.901	1.16	1.27	0.86
Q6NW34	Nucleolus and neural progenitor protein (	NEPRO	64.551	21.345	6	3	3	3	0.96	0.799	1	1.034	1.076	0.99	1.093	1.108	1.16	1.17	1.25
Q92820	Gamma-glutamyl hydrolase OS=Homo sa	GGH	35.964	67.57	24.2	7	15	7	0.998	0.858	1.079	1.067	1.012	1.072	0.912	0.928	1.16	1.12	0.99
Q15274	Nicotinate-nucleotide pyrophosphorylase	QPRT	30.845	69.102	27.6	6	10	6	0.944	0.881	1.074	1.036	1.141	1.176	0.89	0.933	1.16	1.27	1.00
Q9NWS8	Required for meiotic nuclear division pro	RMND1	51.603	22.403	8.5	3	4	3	0.946	0.785	0.939	1.062	1.16	0.961	1.152	1.036	1.16	1.23	1.26

P07902	Galactose-1-phosphate uridylyltransferas	GALT	43.363	31.218	15	4	4	4	0.914	1.037	1.197	1.058	0.888	0.899	1.136	0.947	1.16	0.92	1.07
Q9UP95	Solute carrier family 12 member 4 OS=H	SLC12A4	120.65	74.346	7.3	7	11	7	0.941	0.895	1.07	1.052	1.029	1.19	0.896	0.96	1.16	1.21	1.01
P49589	Cysteine--tRNA ligase, cytoplasmic OS=	CARS	85.472	107.29	20.7	15	19	15	0.917	0.894	1.027	1.066	1.008	0.936	1.174	1.079	1.16	1.07	1.24
Q93034	Cullin-5 OS=Homo sapiens OX=9606 G	CUL5	90.954	39.151	7.8	6	6	6	0.917	0.939	1.007	1.138	1.006	1.209	0.829	1.075	1.16	1.19	1.03
Q9BWU0	Kanadaptin OS=Homo sapiens OX=9606	SLC4A1AP	88.813	120.48	19.8	14	17	14	0.918	0.923	1.046	1.081	1.045	1.032	0.976	0.995	1.16	1.13	1.07
P19634	Sodium/hydrogen exchanger 1 OS=Homo	SLC9A1	90.762	56.213	7.6	4	4	4	0.956	0.925	1.147	1.026	1.048	1	1.087	0.869	1.16	1.09	1.04
P20618	Proteasome subunit beta type-1 OS=Hom	PSMB1	26.489	59.195	27.8	5	10	5	0.936	0.903	1.093	1.031	1.078	1.017	1.027	0.936	1.15	1.14	1.07
Q9UL15	BAG family molecular chaperone regulat	BAG5	51.199	57.068	18.3	6	8	6	0.878	0.903	0.986	1.071	1.122	0.976	1.096	1.082	1.15	1.18	1.22
Q12904	Aminoacyl tRNA synthase complex-inter	AIMP1	34.352	146.88	50.6	12	19	12	0.972	0.932	1.133	1.066	1.013	1.186	0.851	0.898	1.15	1.15	0.92
P49815	Tuberin OS=Homo sapiens OX=9606 GN	TSC2	200.61	77.466	7.1	10	10	10	0.912	0.911	1.084	1.021	1.068	1.209	0.943	0.945	1.15	1.25	1.04
Q12888	TP53-binding protein 1 OS=Homo sapien	TP53BP1	213.57	323.31	22.6	30	37	30	0.938	0.913	1.065	1.072	1.001	0.976	1.092	1.025	1.15	1.07	1.14
Q5T1M5	FK506-binding protein 15 OS=Homo sap	FKBP15	133.63	186.57	12.5	10	15	10	0.913	0.905	1.019	1.079	1.053	1.008	1.083	1.049	1.15	1.13	1.17
P49736	DNA replication licensing factor MCM2	MCM2	101.89	187.01	27.3	21	33	21	0.939	0.84	1.036	1.016	1.043	0.944	1.164	1.014	1.15	1.12	1.22
Q96C57	Protein CUSTOS OS=Homo sapiens OX	CUSTOS	28.17	21.278	14.9	3	3	3	0.935	0.867	1.035	1.043	1.132	1.163	0.937	0.956	1.15	1.27	1.05
Q9NZA1	Chloride intracellular channel protein 5 (	CLIC5	46.502	8.2509	4.4	1	1	1	0.989	0.872	1.105	1.041	0.945	0.924	1.105	1.052	1.15	1.00	1.16
Q9UKN8	General transcription factor 3C polypepti	GTF3C4	91.981	82.527	17.6	11	13	11	0.952	0.878	1.022	1.088	0.993	0.949	1.068	1.085	1.15	1.06	1.18
Q86TV6	Tetratricopeptide repeat protein 7B OS=I	TTC7B	94.178	14.82	2.8	2	2	2	0.849	0.87	0.94	1.042	1.216	1.266	0.744	1.226	1.15	1.44	1.15
Q96BN8	Ubiquitin thioesterase otulin OS=Homo s	OTULIN	40.262	6.8284	2.6	1	2	1	0.924	0.928	1.134	1.001	1.012	0.924	1.192	0.993	1.15	1.05	1.18
Q9NUM4	Transmembrane protein 106B OS=Homo	TMEM106	31.127	6.444	3.6	1	1	1	0.858	0.955	1.096	0.994	1.13	1.059	1.105	0.968	1.15	1.21	1.14
Q96T23	Remodeling and spacing factor 1 OS=Ho	RSF1	163.82	35.51	4.1	5	5	5	1.033	0.957	1.211	1.083	0.988	0.897	0.885	0.911	1.15	0.95	0.90
Q9NY27	Serine/threonine-protein phosphatase 4 r	PPP4R2	46.898	137.04	33.6	8	12	8	0.911	0.957	1.072	1.081	0.961	1.002	1.039	1.004	1.15	1.05	1.09
P33993	DNA replication licensing factor MCM7	MCM7	81.307	197.48	34.6	19	28	19	0.897	0.914	1.06	1.027	1.056	1.002	1.127	1.045	1.15	1.14	1.20
Q9BRR6	ADP-dependent glucokinase OS=Homo s	ADPGK	54.088	26.822	10.3	4	3	3	1.047	0.661	0.965	1.003	1.323	0.758	1.124	1.127	1.15	1.22	1.32
Q9Y508	E3 ubiquitin-protein ligase RNF114 OS=	RNF114	25.694	42.72	26.8	4	9	4	0.964	0.869	1.117	0.995	1.063	1.289	0.795	1.103	1.15	1.28	1.04
P31949	Protein S100-A11 OS=Homo sapiens OX	S100A11	11.74	37.469	34.3	3	9	3	0.967	0.866	1.045	1.067	0.955	0.753	1.255	1.064	1.15	0.93	1.27
Q9Y512	Sorting and assembly machinery compon	SAMM50	51.976	46.9	14.9	5	6	5	0.968	0.806	1.057	0.987	1.081	1.047	1.031	1.031	1.15	1.20	1.16
Q16186	Proteasomal ubiquitin receptor ADRM1 (	ADRM1	42.153	49.49	13.3	5	8	5	0.964	0.916	1.079	1.086	1.042	1.054	0.901	0.977	1.15	1.11	1.00
P22670	MHC class II regulatory factor RFX1 OS	RFX1	104.76	7.8483	1.1	1	2	1	0.959	0.849	1.085	0.997	1.225	0.765	1.223	0.972	1.15	1.10	1.21
P11908	Ribose-phosphate pyrophosphokinase 2 (	PRPS2	34.769	17.764	17.9	3	3	2	0.961	0.902	1.161	0.984	1.093	1.055	0.995	0.942	1.15	1.15	1.04
P58546	Myotrophin OS=Homo sapiens OX=960	MTPN	12.895	60.695	33.1	4	6	4	1.011	0.835	1.034	1.091	1.071	0.82	1.119	0.998	1.15	1.02	1.15
O60942	mRNA-capping enzyme OS=Homo sapie	RNGTT	68.556	66.39	17.4	9	10	9	0.963	0.923	1.128	1.043	1.051	1.053	0.957	0.935	1.15	1.12	1.00
P29590	Protein PML OS=Homo sapiens OX=96	PML	97.55	55.495	9.9	8	8	8	0.963	0.804	1.042	0.992	1.156	1.01	1.026	0.986	1.15	1.23	1.14
Q9Y316	Protein MEMO1 OS=Homo sapiens OX=	MEMO1	33.733	24.417	14.1	3	5	3	0.959	0.899	1.068	1.07	1.077	1.081	0.938	0.938	1.15	1.16	1.01
Q96D05	Uncharacterized protein FAM241B OS=I	FAM241B	13.238	7.1444	9.9	1	1	1	0.84	0.942	1.081	0.969	0.971	1.035	1.274	1.091	1.15	1.13	1.33
O76095	Protein JTB OS=Homo sapiens OX=960	JTB	16.358	18.376	8.9	1	2	1	1.012	0.824	1.112	1	1.253	0.779	1.138	0.912	1.15	1.11	1.12
Q9ULX6	A-kinase anchor protein 8-like OS=Hom	AKAP8L	71.648	86.291	16.3	7	10	7	0.85	0.949	1.02	1.049	1.031	1.05	1.113	1.126	1.15	1.16	1.24
Q7Z6E9	E3 ubiquitin-protein ligase RBBP6 OS=I	RBBP6	201.56	76.881	5.2	9	9	9	0.977	0.832	1.046	1.034	1.093	0.945	1.222	1.02	1.15	1.13	1.24
P02786	Transferrin receptor protein 1 OS=Homo	TFRC	84.87	232.19	32.4	21	49	21	0.914	0.895	1.053	1.026	1.062	1.045	1.065	1.034	1.15	1.16	1.16
O75874	Isocitrate dehydrogenase [NADP] cytopla	IDH1	46.659	108.25	30	12	22	12	0.942	0.902	1.068	1.051	1.067	1.184	0.84	0.971	1.15	1.22	0.98
P53365	Arfaptin-2 OS=Homo sapiens OX=9606	ARFIP2	37.855	54.834	14.4	4	5	4	0.942	0.936	1.066	1.092	1.039	1.034	0.928	0.993	1.15	1.10	1.02
Q04206	Transcription factor p65 OS=Homo sapie	RELA	60.218	48.015	14	6	8	6	0.932	0.874	0.964	1.111	1.102	0.91	1.042	1.088	1.15	1.11	1.18
Q96S55	ATPase WRNIP1 OS=Homo sapiens OX	WRNIP1	72.132	12.719	3.6	2	2	2	0.849	0.87	1.018	0.957	1.31	1.11	1.057	1.028	1.15	1.41	1.21
Q9NPA8	Transcription and mRNA export factor E	ENY2	11.528	32.829	16.8	1	2	1	0.825	1.339	1.369	1.117	0.655	1.24	0.895	0.664	1.15	0.88	0.72
P14061	Estradiol 17-beta-dehydrogenase 1 OS=H	HSD17B1	34.95	40.849	24.4	6	8	6	0.891	0.817	0.972	0.99	1.197	0.87	1.342	1.192	1.15	1.21	1.48
Q12996	Cleavage stimulation factor subunit 3 OS	CSTF3	82.921	77.846	15.1	8	13	8	0.985	0.878	1.052	1.088	1.04	0.994	0.934	1.008	1.15	1.09	1.04
Q96EY5	Multivesicular body subunit 12A OS=Ho	MVB12A	28.783	21.372	18.3	3	3	3	1.004	0.799	1.081	0.99	1.176	0.872	1.235	0.885	1.15	1.14	1.18
Q14696	LRP chaperone MESD OS=Homo sapien	MESD	26.076	55.766	35.5	7	9	7	0.927	0.919	1.062	1.058	1.04	1.06	1.008	1.046	1.15	1.14	1.11
P04083	Annexin A1 OS=Homo sapiens OX=960	ANXA1	38.714	165.25	43.1	12	20	12	0.92	0.88	1.02	1.047	0.929	0.823	1.279	1.141	1.15	0.97	1.34
Q03135	Caveolin-1 OS=Homo sapiens OX=9606	CAV1	20.471	35.201	27.5	5	8	5	0.93	0.796	0.981	1.001	1.208	1.06	1.128	1.135	1.15	1.31	1.31
P28799	Granulins OS=Homo sapiens OX=9606 (	GRN	63.544	84.715	26.5	11	18	11	0.89	0.979	1.05	1.096	1	0.995	1.065	0.932	1.15	1.07	1.07
Q96K76	Ubiquitin carboxyl-terminal hydrolase 47	USP47	157.31	99.654	12.1	12	12	12	0.923	0.914	1.066	1.043	1.054	1.148	0.898	0.993	1.15	1.20	1.03
O00214	Galectin-8 OS=Homo sapiens OX=9606	LGALS8	35.808	29.512	14.8	4	4	4	0.864	0.787	0.926	0.969	1.199	1.045	1.215	1.081	1.15	1.36	1.39
Q5TAQ9	DDB1- and CUL4-associated factor 8 OS	DCAF8	66.851	17.713	4.7	3	4	3	0.911	1.019	1.079	1.136	1.049	1.101	0.785	0.927	1.15	1.11	0.89
P11169	Solute carrier family 2, facilitated glucos	SLC2A3	53.924	160.03	12.5	8	23	8	0.949	0.934	1.088	1.072	0.89	0.892	1.14	1.014	1.15	0.95	1.14
P06132	Uroporphyrinogen decarboxylase OS=Ho	UROD	40.786	95.631	23.7	5	7	5	0.928	0.99	1.134	1.066	0.876	0.982	1.053	0.987	1.15	0.97	1.06
Q9NX58	Cell growth-regulating nucleolar protein	LYAR	43.614	78.3	28.5	8	9	8	0.981	0.864	1.098	1.018	1.054	1.042	1.01	1.034	1.15	1.14	1.11
Q9H2P0	Activity-dependent neuroprotector homeo	ADNP	123.56	78.319	11.2	10	11	10	0.977	0.848	1.033	1.06	1.081	1.107	0.923	1.003	1.15	1.20	1.06
Q9HD15	Steroid receptor RNA activator 1 OS=Ho	SRA1	25.673	33.714	14.8	3	6	3	0.925	0.846	0.916	1.115	1.072	0.778	1.172	1.247	1.15	1.04	1.37
Q8NHP6	Motile sperm domain-containing protein	MOSPD2	59.745	19.534	5.8	3	4	3	1.004	0.883	1.155	1.009	0.989	1.033	0.98	0.992	1.15	1.07	1.05
Q9NVM9	Integrator complex subunit 13 OS=Homo	INTS13	80.224	34.315	7.1	5	5	5	0.89	0.911	1.043	1.022	1.016	1.023	1.064	0.927	1.15	1.13	1.11
Q13029	PR domain zinc finger protein 2 OS=Hor	PRDM2	188.91	7.2828	1.1	1	1	1	1.038	0.73	1.11	0.917	1.332	0.828	1.155	0.963	1.15	1.22	1.20
Q9Y678	Coatomer subunit gamma-1 OS=Homo sa	COPG1	97.717	183.18	29.9	21	29	19	0.949	0.958	1.12	1.066	1.098	0.978	1.052	0.961	1.15	1.09	1.06
Q3SY56	Transcription factor Sp6 OS=Homo sapie	SP6	39.839	33.8	14.1	4	5	4	0.998	0.868	1.109	1.03	1.078	1.129	0.918	0.943	1.15	1.18	1.00
Q9UDW1	Cytochrome b-c1 complex subunit 9 OS=	UQCR10	7.3084	13.733	27	1	1	1	0.937	0.923	1.108	1.024	0.967	0.917	1.112	1.102	1.15	1.01	1.19
Q6DT37	Serine/threonine-protein kinase MRCK g	CDC42BP(	172.46	107.07	11.3	14	15	14	0.966	0.833	1.015	1.047	1.107	1.216	0.9	0.965	1.15	1.29	1.04
Q9H7N4	Splicing factor, arginine/serine-rich 19 O	SCAF1	139.27	45.267	5.8	6	7	5	0.867	0.926	1.075	0.98	0.989	1.007	1.206	0.975	1.15	1.11	1.22
P36915	Guanine nucleotide-binding protein-like	GNL1	68.66	68.93	16.3	8	10	8	0.96	0.89	1.055	1.064	1.062	0.969	1.075	0.975	1.15	1.10	1.11
Q9NUS5	AP-5 complex subunit sigma-1 OS=Hom	AP5S1	22.522	30.366	31.5	4	5	4	0.93	0.9	1.046	1.05	1.039	1.007	1.043	1.196	1.15	1.12	1.22
Q6IQ19	Centriole, cilia and spindle-associated pr	CCSAP	30.216	6.4456	4.4	1	1	1	0.832	0.985	0.986	1.095	1.06	1.221	0.863	1.079	1.15	1.26	1.07
O95302	Peptidyl-prolyl cis-trans isomerase FKBP	FKBP9	63.083	52.169	13.3	7	8	7	0.945	0.874	1.019	1.064	1.132	0.952	1.029	1.125	1.15	1.15	1.18
P25685	DnaJ homolog subfamily B member 1 OS	DNAJB1	38.044	92.717	38.2	11	16	10	0.915	0.891	1.087	0.981	1.042	1.155	1.111	1.058	1.15	1.22	1.20
Q9HCD5	Nuclear receptor coactivator 5 OS=Homo	NCOA5	65.536	65.557	20	8	9	8	0.913	0.859	1.011	1.018	1.113	0.973	1.005	0.976	1.15	1.18	1.12
Q99961	Endophilin-A2 OS=Homo sapiens OX=9	SH3GL1	41.489	86.703	38	11	18	10	0.988	0.998	1.182	1.092	0.932	0.911	0.981	0.801	1.15	0.93	0.90
P46109	Crk-like protein OS=Homo sapiens OX=	CRKL	33.777	157.43	52.8	12	17	12	0.924	0.975	1.079	1.095	0.952	1.032	0.963	0.979	1.14	1.04	1.02
O43660	Pleiotropic regulator 1 OS=Homo sapien	PLRG1	57.193	112.05	32.7	10	15	9	0.948	0.924	1.093	1.05	1.076	1.128	0.905	0.969	1.14	1.18	1.00
Q15654	Thyroid receptor-interacting protein 6 OS	TRIP6	50.287	83.728	34.2	9	10	9	0.88	0.944	1.03	1.058	0.986	1.06	1.103	1.045	1.14	1.12	1.18
O15460	Prolyl 4-hydroxylase subunit alpha-2 OS	P4HA2	60.901	106.05	20.7	8	12	8	0.906	0.946	1.031	1.089	0.944	0.964	1.113	1.005	1.14	1.03	1.14
Q16850	Lanosterol 14-alpha demethylase OS=Ho	CYP51A1	56.805	23.207	8	3	3	3	0.886	0.897	0.963	1.078	1.024	1.226	0.893	1.126	1.14	1.26	1.13
Q15833	Syntaxin-binding protein 2 OS=Homo sa	STXBP2	66.452	70.324	18.5	7	8	7	0.959	0.921	1.056	1.096	1.011	0.936	1.003	1.027	1.14	1.04	1.08
Q93062	RNA-binding protein with multiple splici	RBPMS	21.802	18.59	14.8	2	3	2	0.901	0.997	1.066	1.106	0.941	0.94	1.094	1.005	1.14	0.99	1.11
Q96E09	Protein FAM122A OS=Homo sapiens O	FAM122A	30.529	18.209	16.7	3	3	3	0.965	0.968	1.124	1.088	1.03	1.077	0.912	0.845	1.14	1.09	0.91
O75448	Mediator of RNA polymerase II transcrip	MED24	110.3	36.524	5.2	4	4	4	0.978	0.81	1.048	0.998	1.16	1.088	0.998	0.987	1.14	1.26	1.11
Q5H8A4	GPI ethanolamine phosphate transferase 2	PIGG	108.17	32.996	5.5	4	6	4	0.998	0.925	1.19	1.01	1.114	1.004	1.045	0.771	1.14	1.10	0.94
Q5T3J3	Ligand-dependent nuclear receptor-intera	LRIF1	84.567	18.098	3.9	2	2	2	1.004	0.886	1.043	1.119	1.031	1.014	0.953	0.906	1.14	1.08	0.98
P29144	Tripeptidyl-peptidase 2 OS=Homo sapien	TPP2	138.35	277.99	29.1	29	39	28	0.926	0.886	1.032	1.04	1.038	1.041	0.968	0.971	1.14	1.15	1.07
P33992	DNA replication licensing factor MCM5	MCM5	82.285	122.45	27	16	19	13	0.881	0.887	1.014	1.007	0.991	1.052	1.092	1.109	1.14	1.16	1.24
P10599	Thioredoxin OS=Homo sapiens OX=960	TXN	11.737	44.018	48.6	6	9	5	0.983	0.822	1.03	1.033	0.942	1.021	0.908	1.072	1.14	1.09	1.10
Q6VN20	Ran-binding protein 10 OS=Homo sapien	RANBP10	67.256	27.897	7.3	3	3	3	0.934	0.901	1.012	1.085	0.959	1.135	0.966	1.054	1.14	1.14	1.10
P46459	Vesicle-fusing ATPase OS=Homo sapien	NSF	82.593	131.04	22.7	16	26	16	0.923	0.901	1.058	1.026	1.072	1.048	0.984	1.02	1.14	1.16	1.10
Q96SZ5	2-aminoethanethiol dioxygenase OS=Hor	ADO	29.751	8.7695	4.1	1	2	1	0.962	0.914	1.094	1.048	1.039	0.909	1.146	0.928	1.14	1.04	1.11
P52701	DNA mismatch repair protein Msh6 OS=	MSH6	152.78	323.31	30.7	32	40	31	0.893	0.832	0.966	1.003	1.125	1.018	1.154	1.144	1.14	1.24	1.33
Q9BWH2	FUN14 domain-containing protein 2 OS=	FUNDC2	20.675	19.686	16.9	3	3	3	0.963	0.848	0.99	1.077	1.039	0.964	1.047	1.098	1.14	1.11	1.18
Q9H9Q4	Non-homologous end-joining factor 1 OS	NHEJ1	33.337	6.542	5.4	1	1	1	0.846	0.924	0.978	1.042	1.076	0.983	1.196	1.097	1.14	1.16	1.30
P48556	26S proteasome non-ATPase regulatory s	PSMD8	39.611	65.852	21.1	5	8	5	0.921	0.984	1.095	1.079	0.925	1.047	0.945	0.931	1.14	1.04	0.98
Q9Y6A9	Signal peptidase complex subunit 1 OS=	SPCS1	11.805	55.847	16.7	2	2	2	0.971	0.914	1.109	1.042	1.099	0.938	1.016	0.951	1.14	1.08	1.04
Q9H4A5	Golgi phosphoprotein 3-like OS=Homo s	GOLPH3L	32.767	7.3911	10.9	2	3	1	1.049	0.789	1.071	1.026	1.15	0.939	0.934	1.036	1.14	1.14	1.07
Q9UM54	Unconventional myosin-VI OS=Homo sa	MYO6	149.69	67.726	8	9	9	9	0.877	0.947	1.051	1.03	1.074	1.101	1.038	0.975	1.14	1.19	1.10
Q96K37	Solute carrier family 35 member E1 OS=	SLC35E1	44.772	7.8449	2.9	1	1	1	0.967	0.747	0.913	1.042	1.163	0.937	1.13	1.146	1.14	1.23	1.33
O43670	BUB3-interacting and GLEBS motif-con	ZNF207	50.75	16.687	5.9	2	2	2	1.008	0.965	1.136	1.114	0.948	1.297	0.64	0.858	1.14	1.14	0.76
P56945	Breast cancer anti-estrogen resistance pro	BCAR1	93.371	92.704	19.2	10	11	10	0.935	0.9	1.058	1.034	1.034	1.085	1.001	0.968	1.14	1.15	1.07
Q53T94	TATA box-binding protein-associated fac	TAF1B	68.831	17.128	6.1	3	3	3	1.017	0.883	1.221	0.945	1.072	1.291	0.868	0.778	1.14	1.24	0.87
Q9H8M7	Ubiquitin carboxyl-terminal hydrolase M	MINDY3	49.724	7.4515	2.2	1	1	1	0.922	1.001	1.19	1.002	0.996	0.987	1.13	0.878	1.14	1.03	1.04
Q9H3H3	UPF0696 protein C11orf68 OS=Homo sa	C11orf68	27.354	21.178	14.7	3	3	3	0.922	0.973	1.081	1.079	0.966	1.197	0.781	1.066	1.14	1.14	0.97
Q8WZA9	Immunity-related GTPase family Q protei	IRGQ	62.717	65.45	16.9	7	7	7	0.917	0.879	0.947	1.1	1.113	0.974	1.099	1.037	1.14	1.16	1.19
Q56P03	E2F-associated phosphoprotein OS=Hon	EAPP	32.762	33.535	9.5	2	2	2	0.926	0.906	1.012	1.076	1.076	0.982	1.054	1.019	1.14	1.12	1.13
P08397	Porphobilinogen deaminase OS=Homo sa	HMBS	39.33	48.685	23.5	7	8	7	0.955	0.952	1.134	1.039	1.012	0.989	1.049	0.96	1.14	1.05	1.05
P22061	Protein-L-isoaspartate(D-aspartate) O-me	PCMT1	24.636	78.106	43.6	6	8	5	0.966	0.892	1.053	1.064	1.045	0.946	1.111	1	1.14	1.07	1.14
Q96HE7	ERO1-like protein alpha OS=Homo sapi	ERO1A	54.392	83.485	25	9	12	9	0.939	0.943	1.088	1.056	0.98	0.927	1.127	0.993	1.14	1.01	1.13
Q9BRX5	DNA replication complex GINS protein F	GINS3	24.534	72.192	30.6	6	8	6	0.906	0.855	0.973	1.033	1.18	1.244	0.983	1.094	1.14	1.38	1.18
P49593	Protein phosphatase 1F OS=Homo sapien	PPM1F	49.83	25.257	10.6	3	4	3	0.978	0.913	1.098	1.056	1.074	0.98	1.014	0.93	1.14	1.09	1.03
Q96TA1	Niban-like protein 1 OS=Homo sapiens (	FAM129B	84.137	140.25	17.8	12	17	12	0.916	0.933	1.056	1.05	1.045	1.008	1.05	1.04	1.14	1.11	1.13
Q9UBK9	Protein UXT OS=Homo sapiens OX=96	UXT	18.246	12.206	14	2	2	2	1.001	0.828	0.962	1.121	1.062	1.093	0.901	0.993	1.14	1.18	1.04
O15234	Protein CASC3 OS=Homo sapiens OX=	CASC3	76.277	20.419	6	3	3	3	0.928	0.901	1.115	0.968	1.074	1.191	0.955	1.003	1.14	1.24	1.07
Q14232	Translation initiation factor eIF-2B subur	EIF2B1	33.712	54.633	22.3	6	8	6	0.926	0.918	1.062	1.038	1.014	0.972	1.069	0.955	1.14	1.08	1.10
Q9H875	PRKR-interacting protein 1 OS=Homo sa	PRKRIP1	20.997	13.679	14.7	2	2	2	0.933	0.897	1.122	0.962	1.098	1.231	0.911	0.98	1.14	1.27	1.03
Q969Q5	Ras-related protein Rab-24 OS=Homo sa	RAB24	23.124	28.857	24.1	4	5	4	0.957	0.874	0.976	1.109	1.091	0.87	1.103	1.021	1.14	1.07	1.16
Q9UHR5	SAP30-binding protein OS=Homo sapier	SAP30BP	33.87	48.522	18.2	5	6	5	0.935	0.997	1.165	1.035	0.9	0.927	1.078	1.11	1.14	0.95	1.13
Q96HH9	GRAM domain-containing protein 2B OS	GRAMD2B	47.869	7.9835	2.8	1	1	1	0.769	0.817	0.779	1.027	1.074	1.04	1.189	1.539	1.14	1.33	1.72
O95834	Echinoderm microtubule-associated prote	EML2	70.678	82.668	17.4	9	11	9	0.93	0.876	1.015	1.041	1.101	0.975	1.077	1.116	1.14	1.15	1.21
Q9BTU6	Phosphatidylinositol 4-kinase type 2-alph	PI4K2A	54.022	57.178	15.4	5	7	5	0.886	0.907	1.039	1.002	0.946	1.01	1.121	1.02	1.14	1.09	1.19
Q5TDH0	Protein DDI1 homolog 2 OS=Homo sapi	DDI2	44.522	166.82	45.9	12	18	12	0.976	0.922	1.107	1.053	1.054	0.963	1.076	0.943	1.14	1.06	1.06
Q9NP81	Serine--tRNA ligase, mitochondrial OS=	SARS2	58.282	111.96	31.5	13	18	13	0.934	0.914	1.047	1.056	1.072	1.254	0.908	0.994	1.14	1.26	1.03
Q969U6	F-box/WD repeat-containing protein 5 O	FBXW5	63.922	14.802	6.4	2	2	2	0.884	0.979	1.024	1.096	0.933	1.049	0.998	1.117	1.14	1.06	1.14
Q8WUD4	Coiled-coil domain-containing protein 12	CCDC12	19.181	73.31	19.9	2	3	2	0.959	0.876	1.038	1.05	1.032	1.034	1.129	0.924	1.14	1.13	1.12
O15504	Nucleoporin-like protein 2 OS=Homo sa	NUPL2	44.871	20.095	9	3	3	3	0.923	0.916	1.024	1.068	1.017	1.139	0.993	0.982	1.14	1.17	1.07
Q06546	GA-binding protein alpha chain OS=Hon	GABPA	51.295	69.143	21.1	8	9	8	0.97	0.973	1.18	1.03	1.011	1.025	0.873	0.844	1.14	1.05	0.88
Q5T7N3	KN motif and ankyrin repeat domain-con	KANK4	107.34	20.62	5.9	3	3	3	0.959	0.927	1.131	1.014	0.972	1.145	0.889	1.046	1.14	1.12	1.03
P78332	RNA-binding protein 6 OS=Homo sapier	RBM6	128.64	45.147	6	7	10	7	0.892	0.938	1.053	1.028	1.038	1.018	0.985	1.009	1.14	1.12	1.09
O60825	6-phosphofructo-2-kinase/fructose-2,6-bi	PFKFB2	58.476	16.705	8.5	4	5	2	0.991	0.883	1.112	1.019	1.094	1.074	0.874	1	1.14	1.16	1.00
O00244	Copper transport protein ATOX1 OS=Ho	ATOX1	7.4016	98.847	79.4	5	11	5	0.895	1.091	1.204	1.054	0.916	1.223	0.75	0.894	1.14	1.08	0.83
Q8IWS0	PHD finger protein 6 OS=Homo sapiens	PHF6	41.29	42.943	12.3	4	8	4	1.007	0.893	1.166	0.994	1.109	0.914	1.19	0.943	1.14	1.06	1.12
Q96KG9	N-terminal kinase-like protein OS=Homo	SCYL1	89.63	76.059	15.7	9	10	9	0.974	1.015	1.157	1.104	0.967	1.093	0.797	1.014	1.14	1.04	0.91
O94761	ATP-dependent DNA helicase Q4 OS=H	RECQL4	133.07	7.8971	1.5	1	1	1	0.956	0.824	0.915	1.108	1.077	0.987	1.12	1.014	1.14	1.16	1.20
Q5TFE4	5'-nucleotidase domain-containing protei	NT5DC1	51.844	100.07	24.2	9	15	9	0.936	0.932	1.055	1.068	1.025	1.202	0.93	0.944	1.14	1.19	1.00
Q9NYF8	Bcl-2-associated transcription factor 1 OS	BCLAF1	106.12	187.16	20	15	18	15	0.825	0.934	0.968	1.031	0.987	1.033	1.137	1.024	1.14	1.15	1.23
Q96G25	Mediator of RNA polymerase II transcrip	MED8	29.08	25.469	12.3	2	2	2	0.941	0.96	1.127	1.033	0.995	1.193	0.874	0.955	1.14	1.15	0.96
Q9NW68	BSD domain-containing protein 1 OS=H	BSDC1	47.163	37.331	13.5	5	5	5	0.924	0.919	1.058	1.036	1.159	1.034	0.988	0.963	1.14	1.19	1.06
Q96GX9	Methylthioribulose-1-phosphate dehydra	APIP	27.125	26.912	23.1	4	5	4	0.875	0.932	1.138	0.915	1.092	0.982	1.136	1.151	1.14	1.15	1.27
O94915	Protein furry homolog-like OS=Homo sa	FRYL	339.59	66.608	3.2	9	12	9	0.935	0.883	1.064	1.001	1.199	1.091	1.026	0.864	1.14	1.26	1.04
Q6IA69	Glutamine-dependent NAD(+) synthetase	NADSYN1	79.284	38.56	7.8	4	3	3	0.9	1.022	1.102	1.081	1.03	1.068	0.959	0.908	1.14	1.09	0.97
Q9BT78	COP9 signalosome complex subunit 4 OS	COPS4	46.268	80.872	31.5	10	14	10	0.984	0.975	1.175	1.05	0.982	1.21	0.897	0.869	1.14	1.12	0.90
Q8TF68	Zinc finger protein 384 OS=Homo sapier	ZNF384	63.218	24.047	11.6	4	6	4	0.761	1.007	0.996	1.012	1.103	1.484	0.775	1.107	1.14	1.46	1.06
Q9NR46	Endophilin-B2 OS=Homo sapiens OX=9	SH3GLB2	43.973	77.501	24.6	10	14	9	0.901	0.937	1.055	1.032	1.053	1.025	1.103	1.019	1.14	1.13	1.15
Q96RF0	Sorting nexin-18 OS=Homo sapiens OX=	SNX18	68.894	57.997	16.1	8	11	8	0.873	0.965	1.043	1.044	1.005	0.996	1.151	1.004	1.14	1.09	1.17
Q5VZE5	N-alpha-acetyltransferase 35, NatC auxil	NAA35	83.638	34.764	9.1	5	6	5	0.987	0.874	1.071	1.042	1.071	0.952	1.05	1.042	1.14	1.09	1.12
Q8ND24	RING finger protein 214 OS=Homo sapie	RNF214	77.667	22.321	5	3	3	3	0.934	1.026	1.206	1.019	0.961	0.914	1.108	0.919	1.14	0.96	1.03
P07900	Heat shock protein HSP 90-alpha OS=Ho	HSP90AA1	84.659	323.31	45.4	36	119	23	0.938	0.89	1.033	1.042	1.039	1.028	1.03	1.01	1.14	1.13	1.12
Q96T58	Msx2-interacting protein OS=Homo sapi	SPEN	402.24	80.813	3.4	10	10	10	0.951	0.88	1.06	1.018	1.118	1.025	1.034	1	1.13	1.17	1.11
O14907	Tax1-binding protein 3 OS=Homo sapier	TAX1BP3	13.735	32.726	28.2	2	4	2	0.902	0.976	1.073	1.058	0.938	0.934	1.203	1.012	1.13	1.00	1.18
P29558	RNA-binding motif, single-stranded-inter	RBMS1	44.505	31.361	9.9	3	5	2	0.953	0.858	0.991	1.063	1.05	1.237	0.967	1.097	1.13	1.26	1.14
Q92793	CREB-binding protein OS=Homo sapien	CREBBP	265.35	40.876	4.5	9	10	5	0.945	0.993	1.136	1.062	1.04	1.194	0.877	0.937	1.13	1.15	0.94
O75955	Flotillin-1 OS=Homo sapiens OX=9606	FLOT1	47.355	101.84	27.9	9	12	9	0.937	0.936	1.064	1.06	0.993	1.046	0.906	1.096	1.13	1.09	1.07
O14545	TRAF-type zinc finger domain-containin	TRAFD1	64.84	27.371	7.9	4	4	4	0.934	0.954	1.087	1.054	1.084	1.094	0.85	0.969	1.13	1.15	0.96
Q8WY36	HMG box transcription factor BBX OS=	BBX	105.13	6.0967	1.5	1	1	1	0.798	0.994	0.883	1.149	0.955	1.104	1.109	1.113	1.13	1.15	1.24
Q9UBN7	Histone deacetylase 6 OS=Homo sapiens	HDAC6	131.42	27.326	3.9	4	4	4	0.933	0.972	1.161	0.999	0.855	1	1.069	1.128	1.13	0.97	1.15
Q96B23	Uncharacterized protein C18orf25 OS=H	C18orf25	43.395	7.7079	5.4	1	1	1	0.948	0.743	0.92	0.997	1.257	0.97	1.092	1.168	1.13	1.32	1.34

Q15008	26S proteasome non-ATPase regulatory s	PSMD6	45.531	120.35	36.5	13	19	13	0.978	0.88	1.083	1.023	1.016	0.92	1.106	0.988	1.13	1.04	1.13
O75663	TIP41-like protein OS=Homo sapiens OX	TIPRL	31.444	32.473	14.3	4	6	4	0.92	1.007	1.082	1.102	0.925	1.099	0.96	0.992	1.13	1.05	1.01
P06733	Alpha-enolase OS=Homo sapiens OX=96	ENO1	47.168	323.31	60.4	21	104	20	0.945	0.953	1.099	1.052	0.992	0.96	1.028	0.969	1.13	1.03	1.05
Q6UX04	Spliceosome-associated protein CWC27	CWC27	53.846	40.688	17.4	5	9	5	0.953	0.887	1.054	1.031	1.053	1.038	1.033	1.031	1.13	1.14	1.12
Q3KQV9	UDP-N-acetylhexosamine pyrophosphory	UAP1L1	57.03	43.703	14	6	8	5	0.943	0.943	1.049	1.088	1.041	0.951	1	0.991	1.13	1.06	1.06
Q14562	ATP-dependent RNA helicase DHX8 OS	DHX8	139.31	53.998	7.7	8	11	7	0.93	0.92	1.062	1.034	1.073	0.997	1.052	0.958	1.13	1.12	1.09
Q9NR12	PDZ and LIM domain protein 7 OS=Hon	PDLIM7	49.844	76.341	26.5	8	13	8	0.891	0.962	0.993	1.106	1.023	0.976	1.06	1.037	1.13	1.08	1.13
O95639	Cleavage and polyadenylation specificity	CPSF4	30.255	52.77	32.3	6	7	6	0.949	0.928	1.045	1.081	1.029	0.997	1.133	1.014	1.13	1.08	1.14
P42226	Signal transducer and activator of transcr	STAT6	94.134	6.1893	0.9	1	1	1	0.991	0.826	1.184	0.874	1.097	1.074	1.123	0.983	1.13	1.19	1.16
O00267	Transcription elongation factor SPT5 OS	SUPT5H	121	182.16	24.2	19	25	18	0.966	0.88	1.044	1.046	1.083	0.991	1.039	1.003	1.13	1.12	1.11
Q03426	Mevalonate kinase OS=Homo sapiens OX	MVK	42.45	20.112	9.1	3	3	3	0.955	0.907	1.083	1.025	1.118	1.312	0.743	0.929	1.13	1.31	0.90
Q92581	Sodium/hydrogen exchanger 6 OS=Homo	SLC9A6	74.161	8.5386	2.8	1	1	1	1.048	0.816	0.994	1.116	0.992	0.966	1.106	0.883	1.13	1.05	1.07
O95139	NADH dehydrogenase [ubiquinone] 1 be	NDUFB6	15.489	42.398	32	4	9	4	0.934	0.923	1.078	1.024	1.055	0.973	1.14	1.033	1.13	1.09	1.17
Q9C0C2	182 kDa tankyrase-1-binding protein OS	TNKS1BP1	181.79	140.49	14.7	17	22	17	0.939	0.979	1.103	1.068	0.973	0.944	1.038	0.994	1.13	1.00	1.06
Q13586	Stromal interaction molecule 1 OS=Homo	STIM1	77.422	8.1508	2	1	2	1	0.912	0.875	0.993	1.029	1.121	1.043	1.105	1.018	1.13	1.21	1.19
Q99614	Tetratricopeptide repeat protein 1 OS=Ho	TTC1	33.526	40.981	24	5	10	5	0.97	0.962	1.064	1.122	0.999	1.085	0.913	0.997	1.13	1.08	0.99
Q9NYV4	Cyclin-dependent kinase 12 OS=Homo s	CDK12	164.15	52.466	7.7	8	8	7	0.992	0.834	1.002	1.064	1.128	1.051	1.161	0.944	1.13	1.19	1.15
Q04726	Transducin-like enhancer protein 3 OS=I	TLE3	83.416	153.96	25.3	14	17	12	0.915	1.006	1.094	1.079	1.004	1.119	0.802	0.937	1.13	1.11	0.91
Q63HN8	E3 ubiquitin-protein ligase RNF213 OS=	RNF213	591.4	109.57	2.8	14	14	14	0.902	0.882	0.947	1.071	1.025	0.96	1.113	1.093	1.13	1.11	1.24
A0MZ66	Shootin-1 OS=Homo sapiens OX=9606 (	SHTN1	71.639	153.67	24.7	14	20	14	0.897	0.926	1.02	1.041	0.966	0.946	1.178	1.055	1.13	1.05	1.22
Q5VW32	BRO1 domain-containing protein BROX	BROX	46.476	25.164	11.9	4	4	3	0.942	0.935	0.981	1.141	1.025	0.944	1.144	0.902	1.13	1.05	1.09
Q5VV41	Rho guanine nucleotide exchange factor	ARHGEF1(	80.104	36.712	10.7	6	6	6	0.879	1.021	1.079	1.068	0.943	1.114	0.971	1.03	1.13	1.08	1.05
O15031	Plexin-B2 OS=Homo sapiens OX=9606	PLXNB2	205.12	147.81	13.1	19	22	19	0.951	0.926	1.081	1.04	1.042	1.001	0.988	1.011	1.13	1.09	1.06
Q9NSI2	Protein FAM207A OS=Homo sapiens OX	FAM207A	25.456	28.201	25.2	4	5	4	0.893	1.025	1.023	1.144	1.002	0.635	1.275	1.027	1.13	0.85	1.20
Q92540	Protein SMG7 OS=Homo sapiens OX=9	SMG7	127.28	15.264	1.9	2	2	2	0.958	0.868	1.036	1.027	1.079	1.143	0.957	0.998	1.13	1.22	1.07
Q9Y3X0	Coiled-coil domain-containing protein 9	CCDC9	59.702	14.5	4.1	2	2	2	0.914	0.889	0.959	1.078	1.097	1.003	1.041	1.082	1.13	1.16	1.18
Q53EP0	Fibronectin type III domain-containing pr	FNDC3B	132.89	46.129	7.8	6	6	6	0.942	0.871	1.001	1.047	1.01	0.968	1.129	1.098	1.13	1.09	1.23
Q13889	General transcription factor IIH subunit 3	GTF2H3	34.378	26.132	17.5	4	4	4	0.965	0.921	1.028	1.102	1.031	0.91	1.045	1.003	1.13	1.03	1.09
Q9BY43	Charged multivesicular body protein 4a C	CHMP4A	25.098	36.574	20.3	4	5	4	0.938	0.959	1.085	1.057	0.967	0.929	1.082	1.042	1.13	1.00	1.12
Q13557	Calcium/calmodulin-dependent protein k	CAMK2D	56.369	59.269	19	9	12	8	0.889	0.892	1.018	0.993	0.992	1.081	1.006	1.057	1.13	1.16	1.16
P20839	Inosine-5'-monophosphate dehydrogenase	IMPDH1	55.405	18.73	8.8	3	3	2	0.91	0.949	0.921	1.178	0.932	0.977	0.969	1.163	1.13	1.03	1.15
Q8NFG4	Folliculin OS=Homo sapiens OX=9606 (	FLCN	64.473	7.6225	2.6	1	1	1	0.885	0.929	1.024	1.024	1.068	1.23	1.02	0.942	1.13	1.27	1.08
Q6ZNB6	NF-X1-type zinc finger protein NFXL1 (	NFXL1	101.34	87.826	19.2	11	11	11	0.919	0.923	1.084	0.995	1.002	0.966	1.045	1.056	1.13	1.07	1.14
P48506	Glutamate--cysteine ligase catalytic subu	GCLC	72.765	92.304	21	12	16	12	0.94	0.941	1.042	1.081	1.11	1.018	0.986	0.972	1.13	1.13	1.04
P22681	E3 ubiquitin-protein ligase CBL OS=Ho	CBL	99.632	78.133	15.5	9	10	8	0.95	0.893	1.061	1.019	1.071	0.934	1.09	1.048	1.13	1.09	1.16
Q96CX2	BTB/POZ domain-containing protein KC	KCTD12	35.7	74.491	31.7	8	11	8	0.968	0.922	1.077	1.056	1.037	0.92	1.027	1.038	1.13	1.04	1.09
O95391	Pre-mRNA-splicing factor SLU7 OS=Ho	SLU7	68.386	53.731	17.6	9	7	7	1.027	0.794	0.971	1.084	1.098	1.011	0.972	1.009	1.13	1.16	1.09
Q8IY95	Transmembrane protein 192 OS=Homo s	TMEM192	30.922	35.048	13.3	3	4	3	1.012	0.794	1.032	1.006	1.161	1.006	1.003	1.023	1.13	1.20	1.12
P50502	Hsc70-interacting protein OS=Homo sapi	ST13	41.331	122.64	27.4	9	18	8	0.97	0.969	1.122	1.066	0.945	0.914	1.072	0.892	1.13	0.96	1.01
Q04864	Proto-oncogene c-Rel OS=Homo sapiens	REL	68.519	7.6371	1.8	1	2	1	0.888	0.912	0.998	1.033	1.13	0.994	1.077	1.083	1.13	1.18	1.20
Q8NFW8	N-acylneuraminate cytidylyltransferase O	CMAS	48.379	31.469	12.7	5	7	5	0.914	0.888	1.005	1.028	1.059	1.228	0.897	1.045	1.13	1.27	1.08
Q9NPJ8	NTF2-related export protein 2 OS=Homo	NXT2	16.228	18.518	11.3	1	1	1	0.949	0.939	1.133	0.997	1.102	1.038	0.955	0.982	1.13	1.13	1.03
Q9H0E2	Toll-interacting protein OS=Homo sapien	TOLLIP	30.281	23.809	8.4	2	5	2	0.939	0.949	1.088	1.042	1.063	0.872	1.166	0.952	1.13	1.02	1.12
P61764	Syntaxin-binding protein 1 OS=Homo sa	STXBP1	67.568	43.383	10.9	5	8	5	0.949	0.863	1.017	1.027	1.113	1.14	0.981	1.032	1.13	1.24	1.11
Q9NPI6	mRNA-decapping enzyme 1A OS=Homo	DCP1A	63.309	51.665	15.5	7	6	6	0.932	0.92	1.053	1.036	1.009	0.96	0.899	1.044	1.13	1.06	1.05
Q9NUQ6	SPATS2-like protein OS=Homo sapiens	SPATS2L	61.728	89.842	17.2	8	10	8	0.899	0.845	0.947	1.02	1.092	1.04	1.07	1.137	1.13	1.22	1.27
Q68DK7	Male-specific lethal 1 homolog OS=Hom	MSL1	67.128	22.028	5.5	2	2	2	0.87	0.915	0.988	1.025	1.077	1.215	0.886	1.17	1.13	1.28	1.15
Q86V48	Leucine zipper protein 1 OS=Homo sapie	LUZP1	120.27	66.664	8.9	8	9	8	0.96	0.976	1.103	1.08	0.967	1.193	0.783	1.063	1.13	1.12	0.95
Q86TP1	Exopolyphosphatase PRUNE1 OS=Hom	PRUNE1	50.199	26.335	12.1	4	5	4	0.958	0.861	1.014	1.037	1.016	0.932	1.134	0.978	1.13	1.07	1.16
P05108	Cholesterol side-chain cleavage enzyme,	CYP11A1	60.102	42.24	11.9	5	6	5	0.873	0.913	1.007	1.006	1.132	0.993	0.911	1.014	1.13	1.19	1.08
P32019	Type II inositol 1,4,5-trisphosphate 5-pho	INPP5B	112.85	28.316	3.9	4	4	4	0.992	0.873	1.046	1.056	1.079	0.862	1.117	0.986	1.13	1.04	1.13
Q09028	Histone-binding protein RBBP4 OS=Ho	RBBP4	47.655	38.357	21.9	7	11	4	0.898	0.928	1.031	1.027	1.058	1.131	0.962	1.115	1.13	1.20	1.14
Q12972	Nuclear inhibitor of protein phosphatase	PPP1R8	38.478	61.32	28.2	7	7	7	0.966	0.941	1.078	1.071	1.032	1.143	0.82	0.977	1.13	1.14	0.94
Q14149	MORC family CW-type zinc finger prote	MORC3	107.11	60.835	8.8	6	7	6	0.9	1.04	1.14	1.046	1.086	1.098	0.918	1.012	1.13	1.13	0.99
Q12830	Nucleosome-remodeling factor subunit B	BPTF	338.26	80.053	3.9	11	13	11	0.945	0.909	1.025	1.064	1.029	0.955	1.079	0.968	1.13	1.07	1.10
Q9Y6D6	Brefeldin A-inhibited guanine nucleotide	ARFGEF1	208.76	75.214	11.7	16	18	10	0.93	0.912	1.055	1.02	1.047	0.992	1.052	1.038	1.13	1.11	1.13
Q9NP79	Vacuolar protein sorting-associated prote	VTA1	33.879	73.303	34.5	9	12	9	0.956	0.958	1.078	1.078	1.009	1.016	0.969	0.967	1.13	1.06	1.01
Q9UBF2	Coatomer subunit gamma-2 OS=Homo sa	COPG2	97.621	28.027	9.1	6	9	4	0.883	0.994	1.125	0.989	0.995	1.192	0.745	0.925	1.13	1.17	0.89
Q9Y6B7	AP-4 complex subunit beta-1 OS=Homo	AP4B1	83.259	14.339	4.3	2	2	2	0.997	0.896	1.125	1.007	1.132	0.928	1.023	0.934	1.13	1.09	1.03
Q9BYI3	Hyccin OS=Homo sapiens OX=9606 GN	FAM126A	57.625	19.816	6.7	3	3	3	0.841	1.005	1.031	1.048	1.087	1.439	0.684	1.018	1.13	1.37	0.92
P56537	Eukaryotic translation initiation factor 6	EIF6	26.599	91.591	50.6	8	19	8	0.941	0.909	1.026	1.057	1.057	1.067	1.004	1.006	1.13	1.15	1.09
Q9BZJ0	Crooked neck-like protein 1 OS=Homo s	CRNKL1	100.45	74.837	9.7	8	10	8	0.971	0.816	1.001	1.011	1.12	0.876	1.14	0.972	1.13	1.12	1.18
O75116	Rho-associated protein kinase 2 OS=Hor	ROCK2	160.9	150.72	16.2	21	25	17	0.954	0.929	1.088	1.032	1.072	0.944	1.036	0.987	1.13	1.07	1.07
Q9H2P9	Diphthine methyl ester synthase OS=Hor	DPH5	31.651	22.647	14.4	3	3	3	0.989	0.815	0.954	1.077	1.041	1.157	0.891	1.085	1.13	1.22	1.10
O95785	Protein Wiz OS=Homo sapiens OX=960	WIZ	178.67	26.651	3.3	4	4	4	0.916	0.936	1.022	1.063	1.065	1.07	0.983	1.018	1.13	1.15	1.08
Q13033	Striatin-3 OS=Homo sapiens OX=9606 (	STRN3	87.208	20.799	3	2	3	2	0.978	0.906	1.067	1.054	0.994	1.266	0.865	0.901	1.13	1.20	0.94
O95163	Elongator complex protein 1 OS=Homo s	ELP1	150.25	101.51	13.1	13	15	13	0.969	0.931	1.046	1.093	0.999	0.979	0.891	0.993	1.13	1.04	0.99
P11216	Glycogen phosphorylase, brain form OS=	PYGB	96.695	62.941	15.8	12	13	8	0.963	0.911	1.063	1.046	0.962	0.967	0.958	1.014	1.13	1.03	1.05
Q9HC35	Echinoderm microtubule-associated prote	EML4	108.91	224.55	21.5	18	26	18	0.952	0.908	1.048	1.045	1.012	0.967	1.065	1.018	1.13	1.06	1.12
P51991	Heterogeneous nuclear ribonucleoprotein	HNRNPA3	39.594	210.77	43.4	14	26	14	0.919	0.966	1.102	1.019	1	1.127	0.934	0.991	1.13	1.13	1.02
P30041	Peroxiredoxin-6 OS=Homo sapiens OX=	PRDX6	25.035	177.26	55.8	13	38	13	0.958	1.007	1.118	1.093	0.92	0.913	1.039	0.976	1.13	0.93	1.03
Q70UQ0	Inhibitor of nuclear factor kappa-B kinas	IKBIP	39.309	56.468	18.9	7	8	7	0.958	0.875	0.973	1.089	1.095	0.897	1.162	1.018	1.12	1.09	1.19
Q9NUE0	Palmitoyltransferase ZDHHC18 OS=Hor	ZDHHC18	42.03	6.7588	3.6	1	1	1	0.986	0.881	1.089	1.011	1.101	0.921	1.07	0.994	1.12	1.08	1.11
P31749	RAC-alpha serine/threonine-protein kina	AKT1	55.686	34.458	16.9	5	5	4	0.95	0.893	1.099	0.974	1.074	1.03	1.143	0.943	1.12	1.14	1.13
Q9NWU2	Glucose-induced degradation protein 8 h	GID8	26.748	23.605	15.8	2	2	2	1.136	0.997	1.046	1.353	0.744	1.058	0.599	0.75	1.12	0.84	0.63
Q15911	Zinc finger homeobox protein 3 OS=Hon	ZFHX3	404.41	30.382	1.4	5	5	5	0.97	0.936	1.08	1.063	1.041	0.954	0.999	0.986	1.12	1.05	1.04
Q7LDG7	RAS guanyl-releasing protein 2 OS=Hon	RASGRP2	69.248	12.893	3	2	2	2	0.864	0.802	0.799	1.074	0.974	0.838	1.222	1.552	1.12	1.09	1.67
Q01105	Protein SET OS=Homo sapiens OX=960	SET	33.488	99.3	28.3	7	18	7	0.941	0.953	1.077	1.052	1.025	1.028	0.953	0.942	1.12	1.08	1.00
Q6BDS2	UHRF1-binding protein 1 OS=Homo sap	UHRF1BP	159.48	8.0838	0.9	1	1	1	0.96	0.797	0.989	0.986	1.153	0.808	1.227	1.172	1.12	1.12	1.37
P54578	Ubiquitin carboxyl-terminal hydrolase 14	USP14	56.068	140.33	28.3	14	20	14	0.97	0.917	1.054	1.067	0.975	0.981	1.023	0.953	1.12	1.04	1.05
Q13952	Nuclear transcription factor Y subunit ga	NFYC	50.302	22.342	6.6	3	5	3	0.997	0.909	1.162	0.98	1.181	1.276	0.78	0.778	1.12	1.29	0.82
Q96HA1	Nuclear envelope pore membrane protein	POM121	127.72	52.837	6.2	6	7	2	0.897	0.856	0.898	1.072	1.097	1.011	1.134	1.114	1.12	1.20	1.28
P42695	Condensin-2 complex subunit D3 OS=H	NCAPD3	168.89	15.609	1.6	2	2	2	0.926	0.916	1.05	1.02	1.074	1.343	0.858	0.908	1.12	1.31	0.96
O60547	GDP-mannose 4,6 dehydratase OS=Hom	GMDS	41.949	52.384	16.7	5	6	5	0.942	1.014	1.108	1.09	1.073	1.087	0.855	0.922	1.12	1.10	0.91
Q15814	Tubulin-specific chaperone C OS=Homo	TBCC	39.248	26.009	16.5	4	6	4	1.038	0.913	1.065	1.127	0.91	0.907	1.057	0.909	1.12	0.93	1.01
Q99497	Protein/nucleic acid deglycase DJ-1 OS=	PARK7	19.891	105.93	70.4	11	17	11	0.95	0.989	1.109	1.069	0.947	1.189	0.868	0.905	1.12	1.10	0.91
Q5SWX8	Protein odr-4 homolog OS=Homo sapien	ODR4	51.103	74.596	19.6	7	8	7	0.972	0.962	1.161	1.011	1.053	0.946	1.072	0.884	1.12	1.03	1.01
Q9H0J9	Poly [ADP-ribose] polymerase 12 OS=H	PARP12	79.063	6.2021	1.4	1	1	1	0.957	1.051	1.193	1.062	1.163	0.975	0.841	0.792	1.12	1.06	0.81
P51798	H(+)/Cl(-) exchange transporter 7 OS=H	CLCN7	88.678	23.279	3.6	3	4	3	0.944	0.91	1.054	1.028	1.074	0.923	1.166	0.972	1.12	1.08	1.15
Q16637	Survival motor neuron protein OS=Homo	SMN1	31.848	41.616	14.3	4	4	4	0.965	0.883	1.036	1.039	1.051	0.957	1.016	0.985	1.12	1.09	1.08
Q16514	Transcription initiation factor TFIID subu	TAF12	17.924	6.1211	5	1	1	1	1.011	0.889	1.163	0.97	0.932	1.412	0.749	0.952	1.12	1.23	0.90
Q9P032	NADH dehydrogenase [ubiquinone] 1 alp	NDUFAF4	20.266	36.88	25.7	5	8	5	0.934	0.926	1.073	1.015	1.037	0.975	1.119	1.015	1.12	1.08	1.15
Q99584	Protein S100-A13 OS=Homo sapiens OX	S100A13	11.471	104.67	41.8	5	12	5	0.991	0.918	1.109	1.034	0.957	0.963	1.084	0.981	1.12	1.01	1.08
P43307	Translocon-associated protein subunit alp	SSR1	32.235	13.256	9.1	2	4	2	0.961	0.91	1.071	1.029	1.063	1.177	0.879	1.008	1.12	1.20	1.01
Q9NXV6	CDKN2A-interacting protein OS=Homo	CDKN2AII	61.124	78.986	17.1	7	8	7	0.912	0.968	1.04	1.07	1.058	1.057	0.966	1.004	1.12	1.13	1.05
P62714	Serine/threonine-protein phosphatase 2A	PPP2CB	35.575	13.639	44.7	10	18	1	0.901	1.012	1.083	1.064	0.996	1.021	1.003	1.004	1.12	1.05	1.05
Q99653	Calcineurin B homologous protein 1 OS=	CHP1	22.456	63.942	47.2	7	10	7	0.922	0.992	1.088	1.06	1.046	0.958	0.955	0.865	1.12	1.05	0.95
Q8NCW5	NAD(P)H-hydrate epimerase OS=Homo	NAXE	31.674	31.842	26.4	5	6	4	0.957	0.917	1.084	1.019	1.072	0.914	1.119	1.028	1.12	1.06	1.15
O15446	DNA-directed RNA polymerase I subunit	CD3EAP	54.985	100.33	25.9	7	7	7	0.937	0.858	1.037	0.977	1.156	1.188	0.972	1.077	1.12	1.31	1.14
Q96N21	AP-4 complex accessory subunit Tepsin (	TEPSIN	55.136	17.356	6.9	2	2	2	0.937	0.908	1.035	1.035	0.995	0.971	1.113	1.083	1.12	1.07	1.19
O75157	TSC22 domain family protein 2 OS=Hon	TSC22D2	79.227	27.002	5.3	4	4	3	0.985	0.885	1.074	1.024	1.07	0.967	1.027	1.013	1.12	1.09	1.09
O95319	CUGBP Elav-like family member 2 OS=I	CELF2	54.284	22.337	7.1	3	4	2	0.959	0.871	1.047	1.006	1.097	1.076	0.978	1.049	1.12	1.19	1.11
Q96T37	RNA-binding protein 15 OS=Homo sapie	RBM15	107.19	70.114	12.2	10	11	10	0.936	0.903	1.032	1.031	1.062	1	1.028	1.055	1.12	1.12	1.13
Q8IW45	ATP-dependent (S)-NAD(P)H-hydrate de	NAXD	36.576	19.547	9.5	2	2	2	0.907	0.845	0.959	1.006	1.178	0.947	1.203	1.073	1.12	1.21	1.30
P49023	Paxillin OS=Homo sapiens OX=9606 GI	PXN	64.505	136.68	34.3	13	16	13	0.971	0.941	1.066	1.078	0.887	0.923	1.083	1.042	1.12	0.95	1.11
Q9NX62	Inositol monophosphatase 3 OS=Homo s	IMPAD1	38.681	48.735	9.5	4	5	4	0.914	0.908	1.063	0.98	1.089	1.363	0.814	1.012	1.12	1.35	1.00
O43852	Calumenin OS=Homo sapiens OX=9606	CALU	37.106	224.81	48.6	14	30	14	0.909	1.014	1.1	1.056	0.883	1.179	1.014	0.928	1.12	1.07	1.01
O75391	Sperm-associated antigen 7 OS=Homo sa	SPAG7	26.034	26.177	22.9	4	4	4	0.932	0.661	0.768	1.018	1.147	0.952	1.079	1.553	1.12	1.32	1.65
P08648	Integrin alpha-5 OS=Homo sapiens OX=	ITGA5	114.54	129.47	13.3	11	14	11	0.986	0.939	1.086	1.072	0.946	0.92	1.127	0.987	1.12	0.97	1.10
P10253	Lysosomal alpha-glucosidase OS=Homo	GAA	105.32	82.641	14.4	11	16	11	0.935	0.953	1.092	1.024	1.034	1.051	0.875	1.017	1.12	1.10	1.00
Q92574	Hamartin OS=Homo sapiens OX=9606 C	TSC1	129.77	35.431	4.1	5	5	5	0.99	0.957	1.07	1.112	0.889	0.963	0.915	0.939	1.12	0.95	0.95
Q9NQY0	Bridging integrator 3 OS=Homo sapiens	BIN3	29.665	5.9652	4.3	1	2	1	0.938	0.504	0.603	1.013	1.612	0.865	1.117	1.442	1.12	1.72	1.77
Q96SB3	Neurabin-2 OS=Homo sapiens OX=9606	PPP1R9B	89.191	82.412	16.8	10	10	10	0.932	1.028	1.169	1.027	1.041	1.084	0.999	0.908	1.12	1.08	0.97
Q13621	Solute carrier family 12 member 1 OS=H	SLC12A1	121.45	6.0188	0.9	1	1	1	0.837	1.065	1.107	1.024	1.088	0.688	1.372	0.971	1.12	0.93	1.23
P83436	Conserved oligomeric Golgi complex sub	COG7	86.343	23.66	4.4	3	4	3	0.988	0.939	1.15	1.009	0.999	1.041	1.03	0.898	1.12	1.06	1.00
Q9H0E3	Histone deacetylase complex subunit SA	SAP130	110.32	20.289	3	3	4	3	0.933	0.913	1.033	1.035	1.055	1.078	0.961	1.075	1.12	1.16	1.10
Q12929	Epidermal growth factor receptor kinase s	EPS8	91.88	20.964	4.1	3	3	3	0.953	0.886	1.091	0.969	1.232	1.083	0.914	0.982	1.12	1.26	1.03
P48637	Glutathione synthetase OS=Homo sapien	GSS	52.384	108.53	31.4	12	17	12	0.918	0.938	1.026	1.053	1.037	0.978	1.07	1.049	1.12	1.09	1.14
P15121	Aldose reductase OS=Homo sapiens OX=	AKR1B1	35.853	127.97	35.8	9	24	9	0.982	0.884	1.018	1.072	1.013	0.959	0.958	1.102	1.12	1.06	1.10
Q9BRT9	DNA replication complex GINS protein S	GINS4	26.047	33.67	12.1	2	2	2	0.939	0.92	1.083	0.999	1.06	1.096	0.994	1.01	1.12	1.16	1.08
O43813	LanC-like protein 1 OS=Homo sapiens C	LANCL1	45.283	31.663	12.5	4	5	4	0.923	0.98	1.059	1.072	0.98	0.916	1.129	0.972	1.12	1.00	1.10
Q9P270	SLAIN motif-containing protein 2 OS=H	SLAIN2	62.543	19.081	6.2	3	4	3	0.893	0.881	0.988	0.998	1.181	1.279	0.94	0.976	1.12	1.39	1.08
P35659	Protein DEK OS=Homo sapiens OX=96(	DEK	42.674	113.43	23.2	10	16	10	0.957	0.832	0.972	1.03	1.053	1.021	1.139	1.084	1.12	1.16	1.24
Q8N6T3	ADP-ribosylation factor GTPase-activatin	ARFGAP1	44.667	112.42	26.6	7	12	7	0.971	0.93	1.075	1.052	1.043	1.022	0.958	0.989	1.12	1.09	1.02
Q8N271	Prominin-2 OS=Homo sapiens OX=9606	PROM2	91.882	21.729	4.1	3	4	3	0.97	0.875	1.08	0.984	1.126	0.988	1.056	1.009	1.12	1.15	1.12
Q9NV35	Nucleotide triphosphate diphosphatase N	NUDT15	18.609	7.2739	11	1	1	1	0.918	0.962	1.015	1.088	0.871	0.98	1.15	1.074	1.12	0.98	1.18
Q15906	Vacuolar protein sorting-associated prote	VPS72	40.594	13.678	7.1	2	3	2	1.003	0.827	1.019	1.028	1.133	0.901	1.155	0.955	1.12	1.11	1.15
Q99627	COP9 signalosome complex subunit 8 OS	COPS8	23.225	22.785	14.8	2	4	2	0.892	0.922	1.082	0.947	1.125	0.878	1.21	1.025	1.12	1.10	1.23
Q02218	2-oxoglutarate dehydrogenase, mitochon	OGDH	115.93	269.06	29.4	26	33	26	0.893	0.888	0.978	1.014	1.061	1.157	1.021	1.084	1.12	1.25	1.18
P52747	Zinc finger protein 143 OS=Homo sapier	ZNF143	68.895	13.117	5.3	2	2	2	0.936	0.948	1.111	0.996	1.002	1.064	1.024	1.029	1.12	1.10	1.09
P49768	Presenilin-1 OS=Homo sapiens OX=960	PSEN1	52.667	22.85	10.1	3	3	3	0.887	1.016	1.154	0.974	1.008	0.998	1.196	0.926	1.12	1.05	1.12
Q9H9B1	Histone-lysine N-methyltransferase EHM	EHMT1	141.46	55.008	7.7	8	8	8	0.865	1.157	1.212	1.049	0.886	1.336	0.828	0.791	1.12	1.10	0.80
Q14202	Zinc finger MYM-type protein 3 OS=Ho	ZMYM3	152.38	97.965	11.9	14	18	13	0.969	0.884	1.039	1.033	1.065	1.201	0.885	1.055	1.12	1.22	1.05
O15085	Rho guanine nucleotide exchange factor	ARHGEF1	167.7	28.915	3.8	4	4	4	0.986	0.826	1.069	0.957	1.031	1.052	1.085	1.093	1.12	1.15	1.20
Q13485	Mothers against decapentaplegic homolo	SMAD4	60.438	27.912	8	4	4	4	0.959	0.991	1.147	1.033	0.953	1.089	0.94	0.951	1.12	1.05	0.97
O75351	Vacuolar protein sorting-associated prote	VPS4B	49.301	25.662	13.3	5	6	3	0.829	0.986	1.057	0.972	1.054	1.286	0.957	1.076	1.12	1.29	1.12
Q9BWF3	RNA-binding protein 4 OS=Homo sapier	RBM4	40.313	122.08	39.3	12	16	3	0.937	0.98	1.069	1.073	0.969	0.95	1.024	1.049	1.12	1.00	1.08
O14773	Tripeptidyl-peptidase 1 OS=Homo sapier	TPP1	61.247	41.368	7.5	3	4	3	0.931	0.987	1.063	1.08	1.073	0.884	1.133	1.013	1.12	1.02	1.12
Q9Y4R8	Telomere length regulation protein TEL2	TELO2	91.746	38.19	6.6	5	5	5	0.939	0.904	0.962	1.097	1.021	1.199	0.896	1.013	1.12	1.20	1.04
P61326	Protein mago nashi homolog OS=Homo s	MAGOH	17.163	59.064	32.2	4	7	4	0.976	0.904	1.132	0.968	1.097	1.149	0.895	0.963	1.12	1.19	0.99
P42785	Lysosomal Pro-X carboxypeptidase OS=	PRCP	55.799	41.5	12.1	5	5	5	1.01	0.913	1.054	1.094	0.982	0.92	1.039	0.962	1.12	0.99	1.04
Q8NBN3	Transmembrane protein 87A OS=Homo s	TMEM87A	63.429	19.526	7.7	3	4	3	0.94	0.881	1.038	0.996	1.093	1.052	1.058	0.989	1.12	1.18	1.12
Q12792	Twinfilin-1 OS=Homo sapiens OX=9606	TWF1	40.282	87.939	26.6	7	8	7	0.987	0.976	1.142	1.05	1.035	0.923	1.059	0.894	1.12	1.00	0.99
Q8IWT6	Volume-regulated anion channel subunit	LRRC8A	94.198	31.392	5.4	4	5	4	0.912	0.914	0.985	1.054	0.989	1.105	1.12	0.943	1.12	1.15	1.13
Q9Y383	Putative RNA-binding protein Luc7-like	LUC7L2	46.513	73.615	23.7	8	21	3	0.965	0.883	1.071	0.992	1.056	1.152	0.962	1.015	1.12	1.19	1.07
P51659	Peroxisomal multifunctional enzyme type	HSD17B4	79.685	171.55	31.5	18	23	17	0.963	0.954	1.118	1.022	0.958	1.038	1.029	0.984	1.12	1.04	1.05
Q08945	FACT complex subunit SSRP1 OS=Hom	SSRP1	81.074	173.02	21.4	13	18	13	0.942	0.962	1.063	1.062	1.03	1.248	0.856	0.968	1.12	1.20	0.96
Q9NP64	Nucleolar protein of 40 kDa OS=Homo s	ZCCHC17	27.569	14.41	10.8	2	3	2	0.965	0.899	1.021	1.059	1.04	1.261	0.77	1.028	1.12	1.23	0.96
P53618	Coatomer subunit beta OS=Homo sapien	COPB1	107.14	227.36	28.9	24	34	24	0.981	0.946	1.094	1.056	0.98	0.984	1.03	0.976	1.12	1.02	1.04
Q96A46	Mitoferrin-2 OS=Homo sapiens OX=960	SLC25A28	39.271	6.1142	4.1	1	1	1	0.937	0.845	0.909	1.079	0.92	1.325	0.978	1.056	1.12	1.26	1.14
Q8TE68	Epidermal growth factor receptor kinase s	EPS8L1	80.25	41.234	8.9	6	7	6	0.809	0.699	0.715	0.967	1.227	1.265	1.108	1.405	1.12	1.65	1.67
P18887	DNA repair protein XRCC1 OS=Homo s	XRCC1	69.476	44.812	10.9	6	8	6	0.917	0.964	1.06	1.038	1.006	1.011	1.093	1	1.12	1.07	1.11
Q9Y6J9	TAF6-like RNA polymerase II p300/CBF	TAF6L	67.814	23.572	8.7	4	4	4	0.955	0.945	1.091	1.028	1.018	1.281	0.812	1.015	1.12	1.21	0.96
Q6IN85	Serine/threonine-protein phosphatase 4 r	PPP4R3A	95.367	85.045	12.5	9	15	7	0.942	0.951	1.012	1.099	1.025	1.081	0.938	0.992	1.12	1.11	1.02
Q9UQ35	Serine/arginine repetitive matrix protein 2	SRRM2	299.61	323.31	15.6	31	44	31	0.942	0.927	1.054	1.03	1.092	0.969	1.051	0.998	1.12	1.10	1.10
Q7L8W6	Diphthine--ammonia ligase OS=Homo sa	DPH6	30.306	18.577	12	3	3	3	0.95	1.007	1.105	1.077	1.022	0.909	1.037	0.924	1.11	0.99	1.00

Q15075	Early endosome antigen 1 OS=Homo sap	EEA1	162.46	323.31	40.5	54	79	54	0.957	0.922	1.052	1.043	1.021	0.972	1.017	1.023	1.11	1.06	1.09
Q9P0T7	Transmembrane protein 9 OS=Homo sapi	TMEM9	20.574	18.319	22.4	2	4	2	0.99	0.907	1.083	1.032	0.93	0.999	0.857	0.915	1.11	1.02	0.93
O43148	mRNA cap guanine-N7 methyltransferase	RNMT	54.843	61.008	18.5	8	10	8	0.919	0.865	0.994	0.995	1.007	0.982	1.115	1.043	1.11	1.11	1.21
P55786	Puromycin-sensitive aminopeptidase OS=	NPEPPS	103.28	320.66	33.9	28	50	28	0.948	0.935	1.069	1.03	0.987	0.944	1.09	1.007	1.11	1.03	1.11
Q8NCF5	NFATC2-interacting protein OS=Homo s	NFATC2IP	45.816	32.349	14.3	3	3	3	0.892	0.948	1.077	0.974	1.035	1.361	0.829	1.008	1.11	1.30	1.00
Q8NBT2	Kinetochore protein Spc24 OS=Homo sa	SPC24	22.478	18.664	12.2	2	3	2	0.958	0.909	1.096	0.985	1.116	1.022	1.089	1.033	1.11	1.15	1.14
P62879	Guanine nucleotide-binding protein G(I)/	GNB2	37.331	45.583	21.8	7	11	3	0.938	0.917	1.04	1.027	1.062	1.204	0.92	1.101	1.11	1.22	1.09
Q8N0X7	Spartin OS=Homo sapiens OX=9606 GN	SPART	72.832	22.405	5.6	3	4	3	0.943	0.984	1.005	1.142	0.908	1.199	0.764	1.059	1.11	1.09	0.95
Q562E7	WD repeat-containing protein 81 OS=Ho	WDR81	211.69	7.0399	0.6	1	1	1	0.983	0.859	1.02	1.032	1.123	0.987	1.042	0.993	1.11	1.15	1.10
O75030	Microphthalmia-associated transcription	MITF	58.795	11.758	4	2	2	2	0.907	0.971	1.095	0.997	1.014	0.9	1.154	1.093	1.11	1.02	1.20
Q9UFW8	CGG triplet repeat-binding protein 1 OS=	CGGBP1	18.82	31.377	31.1	5	5	5	0.924	0.896	1.044	0.983	1.116	1.019	1.13	1.01	1.11	1.17	1.18
Q92974	Rho guanine nucleotide exchange factor 2	ARHGEF2	111.54	93.171	12.3	9	11	9	0.91	0.989	1.046	1.068	1.041	0.999	1.009	1.013	1.11	1.07	1.06
Q06124	Tyrosine-protein phosphatase non-recept	PTPN11	68.436	82.125	20.4	10	11	10	0.929	0.998	1.081	1.064	1.009	1.114	0.879	1.013	1.11	1.10	0.98
Q86VU5	Catechol O-methyltransferase domain-co	COMTD1	28.808	23.286	5.7	1	2	1	0.939	0.838	0.943	1.035	1.076	0.822	1.214	1.209	1.11	1.07	1.36
Q92547	DNA topoisomerase 2-binding protein 1	TOPBP1	170.68	50.314	5.7	7	7	7	0.926	0.843	1.001	0.968	1.119	1.034	1.181	1.063	1.11	1.22	1.27
Q9UIC8	Leucine carboxyl methyltransferase 1 OS	LCMT1	38.379	6.1722	3	1	2	1	0.973	0.973	1.085	1.081	0.929	0.913	1.063	0.999	1.11	0.95	1.06
P09960	Leukotriene A-4 hydrolase OS=Homo sa	LTA4H	69.284	159.69	33.7	16	22	16	1.001	0.937	1.083	1.074	0.954	0.961	0.967	0.932	1.11	0.99	0.98
Q8NBF2	NHL repeat-containing protein 2 OS=Ho	NHLRC2	79.443	38.519	9.9	5	5	5	0.912	0.968	1.009	1.083	0.961	1.039	1.127	1.025	1.11	1.06	1.14
Q8TCG1	Protein CIP2A OS=Homo sapiens OX=9	CIP2A	102.18	70.622	11.3	9	9	9	0.939	0.988	1.047	1.097	1.002	1.047	1.005	0.918	1.11	1.06	1.00
P00492	Hypoxanthine-guanine phosphoribosyltra	HPRT1	24.579	68.39	29.8	5	8	5	0.927	0.942	1.033	1.046	0.99	0.98	1.224	1.021	1.11	1.05	1.20
Q2M2I8	AP2-associated protein kinase 1 OS=Ho	AAK1	103.88	182.32	21	15	21	15	0.895	0.92	0.998	1.02	1.03	0.976	1.13	1.12	1.11	1.11	1.24
P63096	Guanine nucleotide-binding protein G(i)	GNAI1	40.361	71.769	30.5	9	14	5	0.909	0.996	1.055	1.063	1.002	1.215	0.866	0.997	1.11	1.16	0.98
P31939	Bifunctional purine biosynthesis protein	ATIC	64.615	316.31	54.7	24	52	24	0.963	0.979	1.118	1.041	0.921	0.908	1.164	0.97	1.11	0.94	1.10
Q9Y2J2	Band 4.1-like protein 3 OS=Homo sapier	EPB41L3	120.68	53.359	9.7	9	9	7	0.95	0.868	1.019	1.002	1.088	1.042	1.01	1.057	1.11	1.17	1.14
P52756	RNA-binding protein 5 OS=Homo sapier	RBM5	92.153	45.128	10.2	8	8	6	0.874	0.99	0.974	1.098	1.031	1.006	1.084	1.061	1.11	1.09	1.15
Q14181	DNA polymerase alpha subunit B OS=H	POLA2	65.947	36.541	9.2	4	4	4	0.931	0.899	1.03	1.004	1.119	1.053	1.08	1.006	1.11	1.19	1.14
O14929	Histone acetyltransferase type B catalytic	HAT1	49.512	123.03	29.4	8	15	8	0.875	0.911	0.983	1.002	1.101	0.999	1.152	1.102	1.11	1.18	1.26
P00338	L-lactate dehydrogenase A chain OS=Ho	LDHA	36.688	61.694	27.7	9	23	8	0.968	0.965	1.097	1.051	0.989	0.859	1.135	0.981	1.11	0.96	1.09
P04424	Argininosuccinate lyase OS=Homo sapie	ASL	51.657	37.044	13.1	5	6	5	0.954	0.901	1.048	1.013	1.124	1.084	1.046	0.904	1.11	1.19	1.05
P15586	N-acetylglucosamine-6-sulfatase OS=Ho	GNS	62.081	66.695	16.1	9	17	9	0.985	0.925	1.097	1.025	0.98	0.931	1.145	0.922	1.11	1.00	1.08
Q7Z2Z2	Elongation factor-like GTPase 1 OS=Ho	EFL1	125.43	35.138	6.2	5	5	5	0.955	1.029	0.996	1.208	0.884	1.016	1.03	0.81	1.11	0.96	0.93
O43707	Alpha-actinin-4 OS=Homo sapiens OX=9	ACTN4	104.85	323.31	60.2	47	115	32	0.938	0.952	1.036	1.063	0.964	0.95	1.087	1.053	1.11	1.01	1.13
P61964	WD repeat-containing protein 5 OS=Hon	WDR5	36.588	101.64	31.4	9	14	9	0.925	0.893	1.01	1.009	1.144	1.002	1.076	1.06	1.11	1.18	1.17
P60903	Protein S100-A10 OS=Homo sapiens OX	S100A10	11.203	67.155	45.4	4	13	4	0.932	0.964	1.042	1.063	0.931	0.921	1.066	1.028	1.11	0.98	1.10
Q9HA64	Ketosamine-3-kinase OS=Homo sapiens	FN3KRP	34.412	28.944	14.6	4	5	4	0.748	1.277	1.246	1.002	0.769	1.625	0.792	0.802	1.11	1.18	0.79
P20290	Transcription factor BTF3 OS=Homo sa	BTF3	22.168	118.97	35	5	6	4	0.996	1.158	1.37	1.021	0.946	0.992	0.917	0.617	1.11	0.90	0.71
Q06830	Peroxiredoxin-1 OS=Homo sapiens OX=	PRDX1	22.11	107.72	63.3	12	35	9	0.959	0.96	1.076	1.054	0.991	0.982	1.009	0.966	1.11	1.03	1.03
P60900	Proteasome subunit alpha type-6 OS=Ho	PSMA6	27.399	73.244	37.8	8	11	7	1.02	0.909	1.135	1.006	1.011	0.927	0.991	0.939	1.11	1.00	1.00
Q15434	RNA-binding motif, single-stranded-inte	RBMS2	43.958	7.2232	5.7	2	3	1	0.919	0.901	1.013	1.007	0.997	1.245	0.794	1.258	1.11	1.23	1.13
Q86UU0	B-cell CLL/lymphoma 9-like protein OS=	BCL9L	157.13	71.591	6.7	9	11	9	0.964	0.885	1.022	1.03	1.076	0.988	1.101	1.009	1.11	1.12	1.14
Q92888	Rho guanine nucleotide exchange factor	ARHGEF1	102.43	103.86	16.6	12	15	11	0.978	0.936	1.057	1.067	0.978	1.009	0.958	0.992	1.11	1.04	1.02
Q8TAT6	Nuclear protein localization protein 4 ho	NPLOC4	68.119	51.535	9	6	9	6	0.949	0.94	1.013	1.083	1.008	0.971	1.106	1.022	1.11	1.05	1.13
A6NDG6	Glycerol-3-phosphate phosphatase OS=H	PGP	34.006	75.195	27.1	7	11	7	0.986	0.886	1.066	1.011	1.109	0.979	1.15	0.956	1.11	1.12	1.13
Q7Z3U7	Protein MON2 homolog OS=Homo sapie	MON2	190.36	56.66	4	6	8	6	0.994	0.924	1.061	1.067	1.058	1.033	1.029	1.021	1.11	1.09	1.07
Q16877	6-phosphofructo-2-kinase/fructose-2,6-bi	PFKFB4	54.039	33.995	9.8	5	6	3	0.89	1.02	1.102	1.017	1.133	1.132	0.888	0.945	1.11	1.19	0.96
O00487	26S proteasome non-ATPase regulatory s	PSMD14	34.577	87.605	19	5	9	5	0.948	1.008	1.114	1.056	0.962	0.95	1.05	1.004	1.11	0.98	1.05
O75586	Mediator of RNA polymerase II transcrip	MED6	28.424	13.375	6.9	2	2	2	0.996	0.815	0.997	1.012	1.259	0.745	1.173	1.043	1.11	1.11	1.22
Q05397	Focal adhesion kinase 1 OS=Homo sapie	PTK2	119.23	37.22	5.6	5	5	5	0.894	1.009	1.072	1.039	0.976	0.949	1.14	1.031	1.11	1.01	1.14
Q8WXI9	Transcriptional repressor p66-beta OS=H	GATAD2B	65.26	53.024	14.7	6	6	6	0.945	0.868	1.018	0.993	1.114	1.032	1.128	0.992	1.11	1.18	1.17
Q13042	Cell division cycle protein 16 homolog O	CDC16	71.655	69.115	16.6	9	9	9	0.964	0.896	1.034	1.029	1.038	0.947	1.121	1.046	1.11	1.07	1.17
Q8WV99	AN1-type zinc finger protein 2B OS=Ho	ZFAND2B	28.022	7.8756	7	1	1	1	0.922	0.957	1.008	1.076	1.008	0.871	1.063	1.159	1.11	1.00	1.18
Q9BTT6	Leucine-rich repeat-containing protein 1	LRRC1	59.241	96.122	23.7	10	13	7	0.956	0.951	1.068	1.047	1.079	1.074	1.036	0.978	1.11	1.13	1.06
P40818	Ubiquitin carboxyl-terminal hydrolase 8	USP8	127.52	74.917	10.6	9	10	9	0.953	0.936	1.034	1.061	0.997	0.892	1.149	1.02	1.11	1.00	1.15
Q15853	Upstream stimulatory factor 2 OS=Homo	USF2	36.954	10.295	3.2	1	1	1	1.016	0.763	0.905	1.068	1.234	0.883	1.098	1.015	1.11	1.19	1.19
Q9Y5Y2	Cytosolic Fe-S cluster assembly factor N	NUBP2	28.825	25.22	22.5	4	4	4	0.868	0.884	0.803	1.14	1.099	0.953	1.036	1.279	1.11	1.17	1.32
O43493	Trans-Golgi network integral membrane p	TGOLN2	51.018	55.367	12.1	6	7	6	1.005	0.896	1.107	1.001	1.111	0.83	1.157	0.937	1.11	1.02	1.10
Q92625	Ankyrin repeat and SAM domain-contain	ANKS1A	123.11	27.924	6.1	4	4	4	1.004	0.934	1.048	1.101	0.941	0.766	1.086	1.099	1.11	0.88	1.13
Q99590	Protein SCAF11 OS=Homo sapiens OX=	SCAF11	164.65	97.261	10.9	11	12	11	0.953	0.94	1.057	1.042	1.021	1.267	0.837	0.938	1.11	1.21	0.94
Q9H7Z6	Histone acetyltransferase KAT8 OS=Hon	KAT8	52.402	38.185	8.1	3	3	3	0.956	0.993	1.175	0.986	1.134	1.022	0.952	0.875	1.11	1.11	0.94
O95870	Protein ABHD16A OS=Homo sapiens O	ABHD16A	63.243	15.026	5	2	2	2	0.959	0.927	1.071	1.02	0.979	1.018	1.156	0.936	1.11	1.06	1.11
Q9NVZ3	Adaptin ear-binding coat-associated prot	NECAP2	28.338	51.713	28.9	5	6	5	0.94	1.021	1.062	1.112	0.95	1.022	0.947	0.983	1.11	1.01	0.98
Q9H8H0	Nucleolar protein 11 OS=Homo sapiens	NOL11	81.123	95.505	20.6	10	17	9	0.968	0.966	1.062	1.082	0.967	1.103	0.908	0.931	1.11	1.07	0.95
Q00535	Cyclin-dependent-like kinase 5 OS=Hom	CDK5	33.304	20.87	14.4	4	4	3	0.98	0.946	1.053	1.082	1.02	0.957	0.959	1.013	1.11	1.03	1.02
Q9BW92	Threonine--tRNA ligase, mitochondrial C	TARS2	81.035	45.116	9.3	6	6	6	0.948	0.831	1.012	0.96	1.126	1.157	0.906	0.99	1.11	1.28	1.07
O60610	Protein diaphanous homolog 1 OS=Hom	DIAPH1	141.35	271.38	28.1	33	53	33	0.962	0.947	1.078	1.038	1.009	0.999	1.048	0.984	1.11	1.05	1.06
Q32P28	Prolyl 3-hydroxylase 1 OS=Homo sapien	P3H1	83.393	34.88	7.2	5	6	5	1.015	1.081	1.264	1.059	0.827	1.174	0.826	0.753	1.11	0.95	0.75
P52209	6-phosphogluconate dehydrogenase, dec	PGD	53.139	164.43	33.3	13	18	13	0.905	0.879	0.994	0.983	1.166	1.109	0.967	1.097	1.11	1.28	1.16
Q96GD0	Pyridoxal phosphate phosphatase OS=H	PDXP	31.698	15.012	8.4	2	2	2	0.91	0.912	1.016	1.003	1.061	0.973	1.171	1.076	1.11	1.12	1.23
O00400	Acetyl-coenzyme A transporter 1 OS=Ho	SLC33A1	60.908	18.197	3.6	2	3	2	0.942	0.909	1.01	1.041	1.09	0.94	1.092	1.042	1.11	1.10	1.15
O14646	Chromodomain-helicase-DNA-binding p	CHD1	196.69	111.07	10.1	14	15	11	0.989	0.921	1.068	1.048	1.01	1.242	0.811	0.964	1.11	1.18	0.93
O15144	Actin-related protein 2/3 complex subuni	ARPC2	34.333	68.72	30.7	8	13	7	1.008	0.93	1.133	1.014	0.927	0.964	1.025	0.987	1.11	0.98	1.04
Q8NE86	Calcium uniporter protein, mitochondrial	MCU	39.866	24.861	10.8	3	3	3	0.976	0.945	1.199	0.929	1	1.134	0.986	0.959	1.11	1.11	1.01
Q8IWJ2	GRIP and coiled-coil domain-containing	GCC2	195.91	235.93	20.8	32	36	30	0.975	0.937	1.065	1.053	1.051	1.019	0.963	0.99	1.11	1.08	1.02
Q96MX6	WD repeat-containing protein 92 OS=Ho	WDR92	39.74	25.596	7.3	2	3	2	0.996	0.928	1.053	1.078	1.031	1.165	0.756	0.997	1.11	1.14	0.91
Q9H993	Protein-glutamate O-methyltransferase O	ARMT1	51.172	30.2	12.2	4	5	4	1.048	0.895	1.076	1.076	0.979	0.941	0.971	0.963	1.11	0.99	1.00
P30419	Glycylpeptide N-tetradecanoyltransferase	NMT1	56.806	113.41	26.8	11	13	9	0.932	0.853	0.933	1.044	1.117	0.962	1.095	1.099	1.11	1.16	1.23
O60784	Target of Myb protein 1 OS=Homo sapie	TOM1	53.818	81.406	21.7	8	10	8	0.944	0.955	1.036	1.067	1.025	0.939	1.058	1.029	1.11	1.03	1.10
O94916	Nuclear factor of activated T-cells 5 OS=	NFAT5	165.76	28.814	3.4	4	5	4	0.934	0.891	0.946	1.075	1.138	0.962	1.032	1.071	1.11	1.15	1.15
Q9UKV3	Apoptotic chromatin condensation induc	ACIN1	151.86	184.67	15.5	17	23	17	0.953	0.92	1.041	1.033	1.012	1.059	0.939	0.965	1.11	1.11	1.02
O75882	Attractin OS=Homo sapiens OX=9606 G	ATRN	158.54	7.8765	1	1	1	1	0.98	0.95	0.979	1.158	0.947	1.229	0.725	0.991	1.11	1.13	0.89
Q6IAA8	Ragulator complex protein LAMTOR1 O	LAMTOR1	17.745	60.095	29.8	3	6	3	0.956	0.891	1.057	0.988	1.067	1.023	1.077	0.966	1.11	1.13	1.11
Q9NXE4	Sphingomyelin phosphodiesterase 4 OS=	SMPD4	97.809	84.371	16.3	11	11	11	0.921	0.899	0.987	1.028	1.111	1.058	0.997	1.049	1.11	1.19	1.12
Q9HB96	Fanconi anemia group E protein OS=Ho	FANCE	58.71	7.0015	2.6	1	1	1	0.891	0.864	0.973	0.97	1.137	0.917	1.446	0.947	1.11	1.17	1.36
Q9UIQ6	Leucyl-cystinyl aminopeptidase OS=Hon	LNPEP	117.35	23.881	3	3	3	3	0.974	0.913	1.022	1.067	1.011	1.066	0.935	1.037	1.11	1.10	1.05
Q92621	Nuclear pore complex protein Nup205 O	NUP205	227.92	204.8	12.8	22	26	22	0.933	0.946	1.054	1.026	1.031	1.025	1.017	0.996	1.11	1.09	1.07
Q9H1I8	Activating signal cointegrator 1 complex	ASCC2	86.359	26.689	6.6	4	4	3	0.885	1.004	1.013	1.078	1.072	0.877	1.067	1.093	1.11	1.03	1.14
Q8IX90	Spindle and kinetochore-associated prote	SKA3	46.359	13.566	7	2	2	2	0.826	1.008	1.153	0.877	1.221	1.331	0.94	0.926	1.11	1.39	1.02
Q7L5L3	Lysophospholipase D GDPD3 OS=Hom	GDPD3	36.596	27.518	15.4	4	6	4	0.895	0.846	0.938	0.989	1.314	1.368	0.764	1.202	1.11	1.54	1.13
Q9NNW7	Thioredoxin reductase 2, mitochondrial C	TXNRD2	56.506	14.286	7.8	3	5	2	1.033	0.849	1.023	1.06	1.116	0.985	0.978	0.934	1.11	1.12	1.02
P62937	Peptidyl-prolyl cis-trans isomerase A OS	PPIA	18.012	135.97	55.8	10	38	10	0.941	0.972	1.086	1.031	1.043	0.989	1.016	0.966	1.11	1.06	1.04
Q9BTM9	Ubiquitin-related modifier 1 OS=Homo s	URM1	11.38	15.534	12.9	1	2	1	0.966	0.948	1.05	1.068	0.974	1.089	0.937	1.002	1.11	1.08	1.01
Q96CM8	Acyl-CoA synthetase family member 2, m	ACSF2	68.124	144.15	22.6	10	11	10	0.927	0.903	1.01	1.015	1.059	1.05	1.041	1.051	1.11	1.15	1.14
Q8WVM7	Cohesin subunit SA-1 OS=Homo sapiens	STAG1	144.43	26.056	6.8	7	7	4	0.996	0.874	0.878	1.191	1.144	0.841	1.01	0.972	1.11	1.06	1.06
Q12981	Vesicle transport protein SEC20 OS=Ho	BNIP1	26.132	71.815	39.9	8	8	8	0.895	0.929	0.999	1.019	1.129	0.869	1.25	1.069	1.11	1.10	1.27
Q9H425	Uncharacterized protein C1orf198 OS=H	C1orf198	36.346	92.094	18	4	4	4	0.941	0.91	1.04	1.007	1.052	0.977	1.1	1.069	1.11	1.10	1.17
Q14676	Mediator of DNA damage checkpoint pro	MDC1	226.66	121.43	14.4	20	20	20	0.946	1.002	1.074	1.08	1.056	0.908	1.162	0.898	1.11	1.01	1.06
Q96FK6	WD repeat-containing protein 89 OS=Ho	WDR89	43.214	48.746	19.4	5	6	5	0.951	0.97	1.071	1.053	1.046	1.216	0.798	0.9	1.11	1.18	0.88
Q01581	Hydroxymethylglutaryl-CoA synthase, cy	HMGCS1	57.293	122.35	34.8	13	21	13	0.929	0.803	0.967	0.948	1.303	1.174	0.928	1.01	1.11	1.43	1.12
P46199	Translation initiation factor IF-2, mitoch	MTIF2	81.316	21.128	4.1	3	4	3	0.937	0.975	1.156	0.958	1.125	0.821	1.196	0.962	1.11	1.02	1.13
Q9ULI0	ATPase family AAA domain-containing p	ATAD2B	164.91	25.008	3.7	4	5	4	0.931	0.877	1.06	0.939	1.109	1.159	0.918	1.047	1.11	1.25	1.09
Q68E01	Integrator complex subunit 3 OS=Homo s	INTS3	118.07	80.856	11.4	8	9	8	0.946	0.885	1.015	1.009	1.063	1.075	0.941	1.067	1.11	1.17	1.10
Q9NV56	MRG/MORF4L-binding protein OS=Ho	MRGBP	22.417	20.432	13.7	2	2	2	0.929	0.922	0.99	1.056	1.108	1.108	0.971	0.982	1.11	1.20	1.06
Q9H446	RWD domain-containing protein 1 OS=H	RWDD1	27.939	13.116	7.4	2	3	2	1.027	0.976	1.196	1.018	0.997	1.119	0.799	0.89	1.11	1.06	0.84
P49770	Translation initiation factor eIF-2B subu	EIF2B2	38.989	43.738	18.2	6	7	6	0.936	0.955	1.117	0.973	1.044	1.268	0.88	0.949	1.11	1.22	0.97
Q8NC56	LEM domain-containing protein 2 OS=H	LEMD2	56.974	35.779	12.1	5	8	5	1.001	0.949	1.109	1.046	1.02	1.167	0.814	1.028	1.11	1.12	0.94
O43776	Asparagine--tRNA ligase, cytoplasmic O	NARS	62.942	121.58	22.3	10	16	10	0.964	0.929	1.043	1.049	1.01	1.012	1.046	1.013	1.11	1.07	1.09
P52888	Thimet oligopeptidase OS=Homo sapiens	THOP1	78.839	60.261	13.8	8	11	8	0.941	0.915	1.013	1.038	1.056	1.277	0.765	1.05	1.11	1.26	0.98
Q96I51	RCC1-like G exchanging factor-like prot	RCC1L	49.996	31.396	11.2	3	3	3	0.968	0.918	1.11	0.974	1.12	1.17	0.906	0.932	1.10	1.21	0.97
Q7Z422	SUZ domain-containing protein 1 OS=H	SZRD1	16.997	6.5779	5.3	1	1	1	0.995	0.959	1.044	1.115	0.905	0.966	0.973	1.018	1.10	0.96	1.02
Q9NWS0	PIH1 domain-containing protein 1 OS=H	PIH1D1	32.363	50.662	27.2	5	5	5	0.972	0.993	1.11	1.061	1	1.1	0.834	0.966	1.10	1.07	0.92
O43572	A-kinase anchor protein 10, mitochondri	AKAP10	73.817	27.627	8.6	4	5	4	0.947	0.984	1.044	1.089	0.944	1.078	0.845	1.021	1.10	1.05	0.97
Q8N3X1	Formin-binding protein 4 OS=Homo sapi	FNBP4	110.26	42.589	5	4	5	4	0.98	0.913	1.096	0.995	1.114	1.182	0.93	0.893	1.10	1.21	0.96
Q6IA86	Elongator complex protein 2 OS=Homo s	ELP2	92.499	48.273	8.8	5	5	5	1.081	0.879	1.122	1.043	1.047	1.051	0.918	0.807	1.10	1.07	0.88
Q9H9E3	Conserved oligomeric Golgi complex sub	COG4	89.082	101.11	13.2	8	10	8	0.954	0.892	1.049	0.99	1.097	0.955	1.12	0.958	1.10	1.11	1.13
Q9H4L4	Sentrin-specific protease 3 OS=Homo sa	SENP3	65.009	25.937	8.5	4	5	4	0.901	0.841	0.873	1.051	1.129	1.253	0.834	1.231	1.10	1.37	1.19
Q86VM9	Zinc finger CCCH domain-containing pro	ZC3H18	106.38	71.993	10	9	12	9	0.971	0.935	1.064	1.041	1.022	1.029	0.962	1.045	1.10	1.08	1.05
Q5JS54	Proteasome assembly chaperone 4 OS=H	PSMG4	13.775	27.264	20.3	1	1	1	0.884	1.004	1.168	0.917	0.994	0.948	1.367	0.917	1.10	1.03	1.21
P62253	Ubiquitin-conjugating enzyme E2 G1 OS	UBE2G1	19.509	36.982	14.7	2	3	2	0.936	1.034	1.096	1.079	0.849	0.976	1.086	0.994	1.10	0.93	1.06
Q68DH5	LMBR1 domain-containing protein 2 OS	LMBRD2	81.171	9.3002	1.6	1	1	1	0.937	0.967	1.068	1.034	1.155	1.186	0.88	0.845	1.10	1.23	0.91
Q8N1B4	Vacuolar protein sorting-associated prote	VPS52	82.22	44.677	7.9	4	5	4	1	0.866	0.976	1.084	0.959	0.931	1.052	1.127	1.10	1.01	1.17
P13716	Delta-aminolevulinic acid dehydratase O	ALAD	36.294	61.853	26.7	8	12	8	0.964	0.922	1.066	1.016	1.002	1.005	1.051	0.978	1.10	1.06	1.08
Q9UKJ3	G patch domain-containing protein 8 OS=	GPATCH8	164.2	64.944	4.3	6	7	6	0.923	0.954	1.033	1.039	1.16	1.147	0.887	0.939	1.10	1.23	0.97
Q9BW91	ADP-ribose pyrophosphatase, mitochond	NUDT9	39.125	17.437	10.6	3	3	2	0.903	0.996	1.031	1.065	1.068	1.055	0.911	1.056	1.10	1.12	1.04
Q8TB24	Ras and Rab interactor 3 OS=Homo sapi	RIN3	107.85	6.7254	1.5	1	1	1	0.962	0.948	1.073	1.035	0.963	0.899	1.162	1.015	1.10	0.97	1.14
P54725	UV excision repair protein RAD23 homo	RAD23A	39.609	18.45	20.1	6	7	3	0.953	0.998	1.133	1.02	0.943	0.945	1.124	0.905	1.10	0.97	1.04
Q96LW7	Caspase recruitment domain-containing p	CARD19	25.589	6.9106	5.7	1	1	1	0.997	0.846	1.02	1.013	1.112	1.273	0.746	1.045	1.10	1.29	0.97
P20929	Nebulin OS=Homo sapiens OX=9606 GI	NEB	772.91	7.2639	0.1	1	2	1	0.885	0.932	0.791	1.213	1.022	0.963	1.046	1.134	1.10	1.09	1.20
Q15257	Serine/threonine-protein phosphatase 2A	PTPA	40.667	30.742	12.3	3	4	3	0.928	0.959	1.06	1.021	1.01	0.972	1.044	0.913	1.10	1.05	1.04
P48200	Iron-responsive element-binding protein 2	IREB2	105.04	31.821	4.8	5	6	5	0.964	0.877	1.072	0.958	1.161	1.337	0.786	0.96	1.10	1.36	0.95
Q13428	Treacle protein OS=Homo sapiens OX=9	TCOF1	152.1	323.31	33.9	44	71	43	0.93	0.895	1.005	1.007	1.101	1.051	1.09	1.016	1.10	1.18	1.15
P63208	S-phase kinase-associated protein 1 OS=	SKP1	18.658	49.816	33.1	6	10	6	0.96	0.963	1.063	1.057	1.081	1.045	0.978	0.981	1.10	1.11	1.02
P24666	Low molecular weight phosphotyrosine p	ACP1	18.042	35.498	32.3	4	6	4	0.989	0.944	1.074	1.057	0.98	0.956	1.051	0.95	1.10	1.00	1.04
Q9UNM6	26S proteasome non-ATPase regulatory s	PSMD13	42.945	122.09	31.4	11	16	11	0.969	0.919	1.049	1.032	1.078	1.04	1.044	1	1.10	1.12	1.08
Q96IJ6	Mannose-1-phosphate guanyltransferase	GMPPA	46.291	27.651	11.7	4	5	3	0.95	0.92	0.978	1.083	0.998	1.181	0.963	0.959	1.10	1.17	1.03
P54920	Alpha-soluble NSF attachment protein O	NAPA	33.232	196.54	54.6	14	19	13	0.932	0.949	1.041	1.032	0.995	1.029	1.064	1.042	1.10	1.08	1.12
Q16543	Hsp90 co-chaperone Cdc37 OS=Homo s	CDC37	44.468	88.219	28.6	9	18	9	0.994	0.95	1.098	1.044	0.947	0.971	1.013	1.008	1.10	0.99	1.04
P13010	X-ray repair cross-complementing protein	XRCC5	82.704	323.31	30.7	20	38	20	0.943	0.954	1.05	1.04	1.027	1.037	0.968	1.005	1.10	1.09	1.04
Q9H1B7	Interferon regulatory factor 2-binding pro	IRF2BPL	82.658	61.389	19.2	11	14	8	0.952	0.945	1.035	1.055	1.062	0.974	0.988	0.939	1.10	1.07	1.02
Q9GZT8	NIF3-like protein 1 OS=Homo sapiens O	NIF3L1	41.968	45.243	13.3	5	6	5	0.929	0.959	1.039	1.041	1.11	1.013	0.97	1.017	1.10	1.12	1.05
P54577	Tyrosine--tRNA ligase, cytoplasmic OS=	YARS	59.143	139.37	34.1	18	29	17	0.958	0.94	1.032	1.059	0.985	0.985	1.026	1.04	1.10	1.04	1.09
Q9Y5K5	Ubiquitin carboxyl-terminal hydrolase is	UCHL5	37.606	40.778	23.1	6	8	6	0.983	0.957	1.078	1.059	0.988	0.958	1.03	0.954	1.10	1.00	1.02
Q9H6D7	HAUS augmin-like complex subunit 4 O	HAUS4	42.399	13.303	5.5	2	2	2	0.882	1.026	1.103	0.998	1.072	1.282	0.74	1.055	1.10	1.23	0.94
P78406	mRNA export factor OS=Homo sapiens (	RAE1	40.968	90.909	30.4	8	15	7	0.948	0.98	1.076	1.047	1.018	1.231	0.817	0.984	1.10	1.17	0.93
Q15527	Surfeit locus protein 2 OS=Homo sapien	SURF2	29.647	9.7615	7.4	1	1	1	0.823	1.384	1.362	1.068	0.702	1.445	0.745	0.615	1.10	0.97	0.62
O43264	Centromere/kinetochore protein zw10 ho	ZW10	88.828	72.047	13.5	8	9	8	0.949	0.953	1.061	1.033	1.023	1.082	0.907	0.994	1.10	1.11	1.00
Q9C005	Protein dpy-30 homolog OS=Homo sapie	DPY30	11.25	8.3203	16.2	1	2	1	0.982	0.861	0.946	1.083	1.18	1.099	0.867	0.986	1.10	1.24	1.01
Q9UPT9	Ubiquitin carboxyl-terminal hydrolase 22	USP22	59.96	13.317	5	2	2	2	0.824	1.13	1.178	0.973	1.066	1.254	0.84	0.951	1.10	1.19	0.92
Q9BUL5	PHD finger protein 23 OS=Homo sapien	PHF23	43.818	28.678	8.9	3	3	3	1.033	0.933	1.108	1.056	1.101	0.92	1	0.823	1.10	1.03	0.93
P31946	14-3-3 protein beta/alpha OS=Homo sapi	YWHAB	28.082	120.17	45.5	10	36	5	0.948	0.979	1.075	1.046	0.934	0.956	1.063	0.98	1.10	0.98	1.06
Q69YN4	Protein virilizer homolog OS=Homo sapi	VIRMA	202.02	46.042	4.9	7	8	7	0.922	1.028	1.062	1.084	0.924	1.001	1.076	0.954	1.10	0.99	1.04
Q86UA1	Pre-mRNA-processing factor 39 OS=Ho	PRPF39	78.429	11.865	2.8	2	2	2	1.002	0.859	0.952	1.096	1.045	1.226	0.763	1.044	1.10	1.22	0.97
Q5JTV8	Torsin-1A-interacting protein 1 OS=Hom	TOR1AIP1	66.248	103.64	20.6	10	14	9	0.988	0.917	1.074	1.022	1.043	1.005	0.955	0.939	1.10	1.08	0.99
Q96MW1	Coiled-coil domain-containing protein 43	CCDC43	25.248	17.074	10.7	2	2	2	0.979	0.837	1.018	0.98	1.119	1.009	1.109	1.03	1.10	1.17	1.18
Q8N1G4	Leucine-rich repeat-containing protein 47	LRRC47	63.472	151.22	31.6	15	23	15	0.936	0.951	1.04	1.036	1.091	0.996	1.056	0.99	1.10	1.11	1.08
Q14847	LIM and SH3 domain protein 1 OS=Hom	LASP1	29.717	119.2	49.4	10	17	10	0.949	0.959	1.068	1.031	1.029	0.97	1.124	1.022	1.10	1.05	1.12
Q7Z7H5	Transmembrane emp24 domain-containin	TMED4	25.943	15.985	12.3	2	5	1	1.008	0.775	0.914	1.047	1.18	0.719	1.304	1.054	1.10	1.07	1.32

P41236	Protein phosphatase inhibitor 2 OS=Hom	PPP1R2	23.015	30.196	21.5	4	6	4	0.963	0.963	1.079	1.039	1.028	1.197	0.902	1.004	1.10	1.16	0.99
P62195	26S proteasome regulatory subunit 8 OS=	PSMC5	45.626	172.4	52.2	19	34	17	0.957	0.972	1.052	1.069	0.947	1.046	1.022	0.96	1.10	1.03	1.03
Q15526	Surfeit locus protein 1 OS=Homo sapiens	SURF1	33.331	14.77	8.7	2	2	2	1.009	0.841	1.06	0.974	1.08	1.206	0.935	0.961	1.10	1.24	1.02
Q53GS7	Nucleoporin GLE1 OS=Homo sapiens O	GLE1	79.835	21.94	5	3	3	3	0.995	0.828	1.009	0.995	1.08	1.117	0.973	1.067	1.10	1.21	1.12
Q8IXH7	Negative elongation factor C/D OS=Hom	NELFCD	66.246	54.184	11.2	5	6	5	0.984	0.942	1.061	1.056	1.047	1.176	0.923	0.913	1.10	1.15	0.95
P35670	Copper-transporting ATPase 2 OS=Homo	ATP7B	157.26	12.872	1.4	2	2	2	0.932	0.98	1.052	1.049	0.908	1.233	0.827	1.102	1.10	1.12	1.01
Q9BZH6	WD repeat-containing protein 11 OS=Ho	WDR11	136.68	43.823	5.2	6	7	6	0.953	0.914	0.955	1.096	1.072	0.88	1.186	1.032	1.10	1.05	1.19
Q96SL1	Disrupted in renal carcinoma protein 2 O	DIRC2	52.087	7.3008	2.1	1	2	1	1.037	0.73	0.937	1.004	1.238	1.007	1.04	1.023	1.10	1.27	1.17
Q8IV38	Ankyrin repeat and MYND domain-conta	ANKMY2	49.298	20.778	8.4	3	3	3	1.039	0.841	1.047	1.018	1.246	0.769	1.196	0.835	1.10	1.07	1.08
Q6S8J3	POTE ankyrin domain family member E (	POTEE	121.36	-2	10.2	7	39	1	0.941	0.91	1.083	0.95	1.166	0.896	1.12	1.07	1.10	1.11	1.18
Q9Y3L3	SH3 domain-binding protein 1 OS=Homo	SH3BP1	75.712	96.158	15	9	11	9	0.923	0.908	0.97	1.041	1.025	1.001	0.974	1.091	1.10	1.11	1.13
Q96RS6	NudC domain-containing protein 1 OS=H	NUDCD1	66.755	33.008	9.9	5	5	4	0.997	0.95	1.051	1.087	1.011	1.24	0.849	0.976	1.10	1.16	0.94
P63241	Eukaryotic translation initiation factor 5A	EIF5A	16.832	72.955	56.5	9	16	9	0.961	0.998	1.069	1.082	0.982	0.984	1.013	1.023	1.10	1.00	1.04
Q15493	Regucalcin OS=Homo sapiens OX=9606	RGN	33.252	7.6811	3	1	1	1	0.837	0.694	0.714	0.967	1.165	0.81	1.47	1.559	1.10	1.29	1.98
Q9BTC8	Metastasis-associated protein MTA3 OS=	MTA3	67.503	36.535	14	7	11	4	0.953	0.946	1.065	1.02	0.986	1.11	0.951	1.067	1.10	1.10	1.06
Q13641	Trophoblast glycoprotein OS=Homo sapi	TPBG	46.031	6.243	1.9	1	1	1	0.83	0.827	0.739	1.08	1.031	1.023	1.19	1.421	1.10	1.24	1.58
Q9Y4G6	Talin-2 OS=Homo sapiens OX=9606 GN	TLN2	271.61	-2	5.1	11	16	1	0.906	1.058	1.023	1.133	0.956	0.965	1.005	0.984	1.10	0.98	1.01
O00232	26S proteasome non-ATPase regulatory s	PSMD12	52.904	120.83	31.8	13	14	11	0.959	0.996	1.047	1.099	0.983	1.152	0.916	1.019	1.10	1.09	0.99
Q7L5N1	COP9 signalosome complex subunit 6 OS	COPS6	36.163	49.356	24.2	7	10	7	1.025	0.93	1.09	1.056	0.918	0.955	1.016	1.002	1.10	0.96	1.03
Q9Y5S9	RNA-binding protein 8A OS=Homo sapi	RBM8A	19.889	34.038	29.3	4	8	4	0.953	0.922	1.02	1.038	1.076	0.973	1.127	0.928	1.10	1.09	1.10
Q86TU7	Histone-lysine N-methyltransferase setd3	SETD3	67.256	165.19	19.2	11	22	11	0.997	0.94	1.084	1.042	0.998	0.981	1.071	0.964	1.10	1.02	1.05
Q99417	c-Myc-binding protein OS=Homo sapien	MYCBP	11.967	40.737	30.1	2	3	2	0.998	0.962	1.094	1.057	0.988	1.07	0.889	0.959	1.10	1.05	0.94
Q9ULK4	Mediator of RNA polymerase II transcrip	MED23	156.47	53.364	6.9	8	9	8	0.964	0.905	1.021	1.03	1.065	1.219	0.906	1.055	1.10	1.22	1.05
P52594	Arf-GAP domain and FG repeat-containi	AGFG1	58.259	75.076	17.1	8	9	7	0.964	1.009	1.12	1.045	0.967	0.93	1.082	0.973	1.10	0.96	1.04
Q6NUM9	All-trans-retinol 13,14-reductase OS=Ho	RETSAT	66.819	18.812	6.4	3	3	3	0.966	0.946	1.002	1.096	0.922	1.299	0.675	1.119	1.10	1.16	0.94
Q8IYI6	Exocyst complex component 8 OS=Homo	EXOC8	81.798	55.849	12.7	8	8	8	1.018	0.928	1.113	1.022	1.042	0.817	1.138	0.94	1.10	0.96	1.07
Q76FK4	Nucleolar protein 8 OS=Homo sapiens O	NOL8	131.61	57.371	8.7	9	9	9	0.928	0.916	1	1.023	1.179	0.993	1.007	1.02	1.10	1.18	1.10
O94830	Phospholipase DDHD2 OS=Homo sapie	DDHD2	81.031	6.55	1.5	1	1	1	0.965	1.027	1.164	1.021	1.023	0.888	1.134	0.834	1.10	0.96	0.99
Q9Y4B6	DDB1- and CUL4-associated factor 1 OS	DCAF1	169.01	78.423	9.2	12	14	12	0.977	0.944	1.071	1.036	0.938	1.032	0.95	0.989	1.10	1.03	1.01
Q96G23	Ceramide synthase 2 OS=Homo sapiens (	CERS2	44.876	6.7136	4.2	1	2	1	0.918	1.127	1.191	1.052	0.847	1.449	0.706	0.795	1.10	1.12	0.73
O14744	Protein arginine N-methyltransferase 5 O	PRMT5	72.683	96.412	21.8	12	17	12	0.948	0.984	1.056	1.063	1.027	0.975	1.016	0.975	1.10	1.04	1.03
Q00796	Sorbitol dehydrogenase OS=Homo sapie	SORD	38.324	24.503	11.2	4	5	4	0.962	1.007	1.171	0.988	1.035	0.937	1.171	0.92	1.10	1.00	1.06
Q641Q2	WASH complex subunit 2A OS=Homo s	WASHC2A	147.18	75.274	11.4	8	8	8	0.975	0.901	1.011	1.046	0.982	0.907	1.137	1.117	1.10	1.01	1.20
Q6PJG6	BRCA1-associated ATM activator 1 OS=	BRAT1	88.118	18.669	6.7	3	3	3	1.034	0.824	0.955	1.082	0.969	1.067	0.982	1.055	1.10	1.10	1.10
Q16555	Dihydropyrimidinase-related protein 2 O	DPYSL2	62.293	89.876	25.2	10	12	10	0.903	0.872	0.948	0.998	1.086	1.073	1.096	1.084	1.10	1.22	1.23
Q9NVH2	Integrator complex subunit 7 OS=Homo s	INTS7	106.83	42.31	7.6	6	7	6	1.046	0.918	1.282	0.871	1.058	0.847	1.008	1.208	1.10	0.97	1.13
O43592	Exportin-T OS=Homo sapiens OX=9606	XPOT	109.96	112.42	13.3	11	18	11	0.966	0.863	0.986	1.019	1.046	1.011	1.06	1.028	1.10	1.12	1.14
Q9H6U8	Alpha-1,2-mannosyltransferase ALG9 OS	ALG9	69.863	12.777	5.1	2	2	2	0.95	0.986	1.103	1.019	1.072	0.905	1.143	0.894	1.10	1.02	1.05
Q9UM00	Calcium load-activated calcium channel (	TMCO1	21.175	15.143	12.2	2	3	2	1.011	0.833	1.008	1.013	1.081	1.01	0.965	1.076	1.10	1.13	1.11
Q9Y6E0	Serine/threonine-protein kinase 24 OS=H	STK24	49.307	56.628	25.3	10	12	4	0.985	0.943	1.073	1.04	1.087	1.159	0.881	0.996	1.10	1.16	0.97
Q9NPE3	H/ACA ribonucleoprotein complex subu	NOP10	7.7059	12.773	32.8	2	2	2	0.922	0.903	1	1	1.012	1.042	1.11	1.13	1.10	1.13	1.23
Q9NXD2	Myotubularin-related protein 10 OS=Ho	MTMR10	88.272	20.88	4.6	3	3	3	0.944	0.937	1.052	1.009	1.043	0.999	1.125	0.98	1.10	1.09	1.12
Q92620	Pre-mRNA-splicing factor ATP-depende	DHX38	140.5	108.62	15.2	15	16	15	0.929	0.932	0.999	1.04	1.053	1.005	1.022	1.055	1.10	1.11	1.12
Q14C86	GTPase-activating protein and VPS9 don	GAPVD1	164.98	203.18	16.9	19	23	18	0.926	0.978	1.048	1.038	0.962	1.049	0.979	0.991	1.10	1.06	1.03
P45974	Ubiquitin carboxyl-terminal hydrolase 5 (	USP5	95.785	158.8	28.6	18	24	18	0.944	0.982	1.107	1.003	0.976	0.995	1.039	0.92	1.10	1.02	1.02
Q96GK7	Fumarylacetoacetate hydrolase domain-c	FAHD2A	34.596	39.762	21.7	5	8	5	0.962	1.027	1.123	1.056	0.915	0.937	1.124	0.936	1.10	0.93	1.04
P49354	Protein farnesyltransferase/geranylgeran	FNTA	44.408	13.977	5.3	2	3	2	0.891	1.025	1.049	1.05	1.042	1.134	0.951	0.959	1.10	1.14	1.00
Q9NR50	Translation initiation factor eIF-2B subu	EIF2B3	50.24	80.214	24.6	8	9	8	0.977	0.929	1.088	1	1.106	0.896	1.164	0.918	1.10	1.05	1.09
Q9BT73	Proteasome assembly chaperone 3 OS=H	PSMG3	13.104	9.632	10.7	1	1	1	0.735	1.204	1.087	1.037	1.148	1.335	0.787	0.903	1.10	1.28	0.87
P08962	CD63 antigen OS=Homo sapiens OX=96	CD63	25.636	15.836	7.6	2	6	2	1.011	0.929	1.059	1.066	0.959	0.883	1.149	1.002	1.10	0.95	1.11
Q5MIZ7	Serine/threonine-protein phosphatase 4 r	PPP4R3B	97.457	10.519	5.1	4	6	2	0.954	0.83	0.955	0.999	1.151	1.216	0.853	1.142	1.10	1.33	1.12
Q9NPF5	DNA methyltransferase 1-associated prot	DMAP1	52.992	51.011	14.3	5	5	5	0.937	0.933	1.013	1.035	1.075	1.188	0.893	1.071	1.10	1.21	1.05
Q15056	Eukaryotic translation initiation factor 4H	EIF4H	27.385	88.57	26.2	5	10	5	0.965	0.938	1.031	1.053	0.968	0.953	1.109	1.024	1.10	1.01	1.12
Q96L91	E1A-binding protein p400 OS=Homo sa	EP400	343.48	66.233	3.4	9	11	9	0.975	0.878	1.044	0.985	1.092	1.107	1.016	1.005	1.09	1.19	1.09
Q9BXP2	Solute carrier family 12 member 9 OS=H	SLC12A9	96.109	53.669	11.9	7	10	7	0.961	0.935	1.045	1.031	1.012	1.086	0.92	1.022	1.09	1.11	1.02
Q8N9N7	Leucine-rich repeat-containing protein 57	LRRC57	26.754	17.488	9.6	2	3	2	0.943	0.987	0.952	1.161	0.976	0.953	0.991	1.016	1.09	1.00	1.04
Q92945	Far upstream element-binding protein 2 (	KHSRP	73.114	323.31	46.3	24	45	21	0.966	1.008	1.132	1.029	0.983	1.031	0.938	0.91	1.09	1.02	0.94
Q5ZPR3	CD276 antigen OS=Homo sapiens OX=9	CD276	57.235	16.508	8.6	2	2	2	0.945	0.987	1.138	0.977	0.985	1.135	1.013	0.936	1.09	1.10	1.01
P21283	V-type proton ATPase subunit C 1 OS=H	ATP6V1C1	43.941	88.669	30.6	12	16	11	0.962	0.907	1.009	1.037	1.082	0.975	1.048	1.075	1.09	1.10	1.14
Q9H2J4	Phosducin-like protein 3 OS=Homo sapi	PDCL3	27.614	46.795	24.7	5	6	5	0.981	0.911	0.999	1.072	1.071	0.949	1.075	1.021	1.09	1.07	1.11
P51116	Fragile X mental retardation syndrome-re	FXR2	74.222	46.221	17.5	9	9	7	0.937	1.008	1.066	1.063	0.963	1.001	0.919	0.946	1.09	1.01	0.96
Q12986	Transcriptional repressor NF-X1 OS=Ho	NFX1	124.39	15.995	2.1	2	2	2	0.913	1.001	1.105	0.99	1.012	1.115	1.059	0.933	1.09	1.11	1.04
A1L0T0	Acetolactate synthase-like protein OS=H	ILVBL	67.867	75.173	19	8	10	7	0.95	1.008	1.105	1.038	0.912	1.025	1.025	0.912	1.09	0.99	0.99
Q14157	Ubiquitin-associated protein 2-like OS=H	UBAP2L	114.53	123.73	19.9	13	19	13	0.976	0.994	1.123	1.033	1.008	1.236	0.767	0.959	1.09	1.14	0.88
O43242	26S proteasome non-ATPase regulatory s	PSMD3	60.977	142.56	31.1	16	23	15	0.962	0.945	1.027	1.06	0.97	0.999	0.995	1.043	1.09	1.03	1.07
P33240	Cleavage stimulation factor subunit 2 OS	CSTF2	60.959	113.86	27.4	12	19	8	0.945	0.941	1.057	1.007	1.074	1.029	1.024	0.971	1.09	1.12	1.06
Q7Z589	BRCA2-interacting transcriptional repres	EMSY	141.47	12.336	1.7	2	2	2	0.953	0.977	1.165	0.947	1.218	0.92	1.056	0.886	1.09	1.11	1.01
O00273	DNA fragmentation factor subunit alpha	DFFA	36.521	77.063	26.3	7	9	7	0.941	0.968	1.015	1.074	1.02	1.162	0.885	1.004	1.09	1.14	0.99
O43739	Cytohesin-3 OS=Homo sapiens OX=960	CYTH3	46.348	47.552	18.8	5	5	2	0.941	0.922	1.05	0.988	1.015	1.03	1.119	1.046	1.09	1.10	1.16
Q5BJH7	Protein YIF1B OS=Homo sapiens OX=9	YIF1B	34.435	14.533	12.1	2	3	2	0.936	1.088	1.15	1.064	0.969	0.872	0.979	1.001	1.09	0.91	0.98
O43172	U4/U6 small nuclear ribonucleoprotein P	PRPF4	58.449	118.9	25.5	11	15	11	0.921	0.912	1	1.005	1.055	1.138	0.953	1.019	1.09	1.20	1.08
O75475	PC4 and SFRS1-interacting protein OS=	PSIP1	60.103	122.82	24.5	12	18	10	0.959	0.909	0.986	1.057	1.052	1.024	1.026	1.027	1.09	1.11	1.10
P20962	Parathymosin OS=Homo sapiens OX=96	PTMS	11.53	15.234	10.8	1	2	1	1.044	0.967	1.12	1.079	1.013	0.794	1.066	0.868	1.09	0.90	0.96
Q8WUY1	Protein THEM6 OS=Homo sapiens OX=	THEM6	23.865	24.319	15.4	3	3	3	0.926	0.949	1.044	1.006	1.146	1.001	0.963	1.075	1.09	1.15	1.09
Q8WUM4	Programmed cell death 6-interacting prot	PDCD6IP	96.022	232.51	32.7	25	43	24	0.971	0.969	1.069	1.052	0.978	0.989	1.044	0.968	1.09	1.01	1.04
Q92572	AP-3 complex subunit sigma-1 OS=Hom	AP3S1	21.732	45.919	34.7	5	6	5	0.968	0.969	1.069	1.048	0.994	0.863	1.135	0.879	1.09	0.96	1.04
Q15269	Periodic tryptophan protein 2 homolog O	PWP2	102.45	68.029	11.2	9	11	9	1.013	0.795	0.993	0.983	1.2	0.912	1.177	0.982	1.09	1.17	1.19
Q15554	Telomeric repeat-binding factor 2 OS=H	TERF2	59.593	20.488	8.1	3	3	3	0.967	0.885	1.024	1	1.109	1.047	1.019	1.026	1.09	1.16	1.10
O43488	Aflatoxin B1 aldehyde reductase member	AKR7A2	39.589	36.937	23.1	5	6	5	0.91	0.967	1.01	1.041	1.061	1.113	1.052	0.962	1.09	1.16	1.07
Q9UBQ7	Glyoxylate reductase/hydroxypyruvate re	GRHPR	35.668	72.223	22.6	5	11	5	0.981	0.94	1.079	1.02	0.982	0.977	1.064	0.929	1.09	1.02	1.04
Q9Y3I0	tRNA-splicing ligase RtcB homolog OS=	RTCB	55.21	148.92	38.2	16	31	16	0.938	0.985	1.053	1.048	1.004	1.068	0.885	0.931	1.09	1.08	0.94
P09486	SPARC OS=Homo sapiens OX=9606 GI	SPARC	34.632	39.782	13.2	4	5	4	0.98	0.846	1.079	0.916	1.048	0.914	1.316	1.173	1.09	1.07	1.36
P36406	E3 ubiquitin-protein ligase TRIM23 OS=	TRIM23	64.066	7.1525	1.7	1	2	1	0.988	0.958	1.126	1	0.947	1.06	0.984	1.006	1.09	1.03	1.02
Q9NQX6	Zinc finger protein 331 OS=Homo sapier	ZNF331	53.738	70.463	19.9	8	8	8	0.925	0.859	0.888	1.061	1.135	1.05	0.982	1.143	1.09	1.22	1.19
Q9BW71	HIRA-interacting protein 3 OS=Homo sa	HIRIP3	61.956	6.5198	1.4	1	1	1	0.884	0.924	1.005	0.97	1.162	1.226	0.955	1.043	1.09	1.32	1.11
Q9BXS5	AP-1 complex subunit mu-1 OS=Homo s	AP1M1	48.586	56.358	23.4	7	8	4	0.988	0.953	1.118	1.002	1.056	0.933	1.097	0.93	1.09	1.02	1.04
O95394	Phosphoacetylglucosamine mutase OS=H	PGM3	59.851	113.4	25.8	11	15	10	0.89	0.928	0.972	1.013	1.037	1.029	1.129	1.086	1.09	1.14	1.22
Q8TBZ6	tRNA methyltransferase 10 homolog A O	TRMT10A	39.718	22.127	12.1	3	4	3	0.996	0.9	1.12	0.95	1.123	0.891	1.132	0.968	1.09	1.06	1.11
Q53F19	Nuclear cap-binding protein subunit 3 OS	NCBP3	70.592	35.293	7.4	4	6	4	0.934	0.963	1.069	1.002	1.046	1.206	0.9	0.903	1.09	1.19	0.95
P35754	Glutaredoxin-1 OS=Homo sapiens OX=9	GLRX	11.776	21.06	30.2	2	3	2	0.99	1.115	1.174	1.124	0.746	0.793	1.039	0.996	1.09	0.73	0.97
Q05932	Folylpolyglutamate synthase, mitochondr	FPGS	64.608	13.758	4.6	2	2	2	0.957	1.075	1.198	1.02	0.918	0.974	1.031	0.899	1.09	0.93	0.95
Q9UHB6	LIM domain and actin-binding protein 1	LIMA1	85.225	270.48	39.1	25	45	24	0.907	0.951	1.002	1.026	1.008	0.98	1.14	1.07	1.09	1.07	1.19
O94842	TOX high mobility group box family mer	TOX4	66.194	57.823	12.2	6	7	6	1.038	0.909	1.11	1.015	1.046	0.987	0.897	0.925	1.09	1.04	0.94
P12004	Proliferating cell nuclear antigen OS=Ho	PCNA	28.768	111.69	44.4	9	28	9	0.923	0.853	0.972	0.966	1.098	1.078	1.063	1.135	1.09	1.23	1.24
Q8N2Z9	Centromere protein S OS=Homo sapiens	CENPS	15.893	6.1549	6.5	1	1	1	0.852	0.738	0.739	0.996	1.382	1.167	0.959	1.349	1.09	1.60	1.45
Q9BZL4	Protein phosphatase 1 regulatory subunit	PPP1R12C	84.88	31.061	7.4	4	4	4	0.99	1.007	1.062	1.117	0.906	0.937	1.015	0.941	1.09	0.92	0.98
Q9H4I3	TraB domain-containing protein OS=Ho	TRABD	42.321	20.512	5.6	2	3	2	0.991	0.888	1.075	0.975	1.184	0.851	1.191	0.912	1.09	1.08	1.12
Q9GZT9	Egl nine homolog 1 OS=Homo sapiens C	EGLN1	46.02	6.7949	2.1	1	1	1	0.991	0.878	0.988	1.051	1.029	0.944	1.149	0.989	1.09	1.06	1.14
P61981	14-3-3 protein gamma OS=Homo sapiens	YWHAG	28.302	29.801	26.7	7	23	4	0.997	0.939	1.063	1.049	0.99	1.021	0.978	0.972	1.09	1.04	1.01
P52948	Nuclear pore complex protein Nup98-Nu	NUP98	197.58	251.37	16	24	31	24	0.942	0.956	1.019	1.051	1.034	1.024	1.014	1.015	1.09	1.08	1.07
P20936	Ras GTPase-activating protein 1 OS=Ho	RASA1	116.4	150.5	19.1	16	22	16	0.916	0.928	0.99	1.021	1.074	0.946	1.122	1.113	1.09	1.10	1.21
Q96BW5	Phosphotriesterase-related protein OS=H	PTER	39.017	38.496	16	5	6	5	0.937	1.029	1.102	1.042	1.005	0.959	0.922	1.083	1.09	1.00	1.02
P29374	AT-rich interactive domain-containing pr	ARID4A	142.75	26.977	3.6	4	4	4	1.005	0.961	1.141	1.003	1.002	0.921	1.155	0.851	1.09	0.98	1.02
P55081	Microfibrillar-associated protein 1 OS=H	MFAP1	51.958	38.398	11.2	3	5	3	0.917	0.973	1.039	1.022	1.017	1.227	0.899	1.042	1.09	1.19	1.03
Q16563	Synaptophysin-like protein 1 OS=Homo	SYPL1	28.565	24.414	10	2	4	2	0.881	0.877	0.862	1.055	1.051	0.97	1.134	1.285	1.09	1.15	1.38
Q96JB2	Conserved oligomeric Golgi complex sub	COG3	94.095	42.455	8	6	6	6	0.921	0.959	0.995	1.055	1.031	1.025	1.068	1.019	1.09	1.09	1.11
Q8N684	Cleavage and polyadenylation specificity	CPSF7	52.049	90.663	24.2	11	15	11	0.955	0.914	1.036	1.002	1.134	1.174	0.9	1.001	1.09	1.23	1.02
P06307	Cholecystokinin OS=Homo sapiens OX=	CCK	12.669	6.678	7.8	1	1	1	0.772	0.945	1.103	0.769	1.136	1.099	1.313	1.277	1.09	1.30	1.51
O75094	Slit homolog 3 protein OS=Homo sapien	SLIT3	167.71	6.4394	0.7	1	1	1	0.942	0.956	1.051	1.018	1	0.998	1.142	0.977	1.09	1.05	1.12
Q9BUP3	Oxidoreductase HTATIP2 OS=Homo sap	HTATIP2	27.049	62.18	31	6	12	6	0.96	0.995	1.128	1.003	1.007	0.986	0.901	0.956	1.09	1.02	0.95
Q8IUF8	Ribosomal oxygenase 2 OS=Homo sapie	RIOX2	52.8	25.519	9.2	4	6	4	0.975	0.881	0.989	1.034	1.132	1.276	0.759	0.977	1.09	1.30	0.94
Q8IZ69	tRNA (uracil-5-)-methyltransferase homo	TRMT2A	68.725	110.5	25	11	11	11	0.923	0.912	0.986	1.014	1.02	1.22	0.919	1.054	1.09	1.22	1.08
Q9H0G5	Nuclear speckle splicing regulatory prote	NSRP1	66.389	20.761	6.3	3	3	3	0.983	0.966	1.04	1.084	1.027	1.184	0.816	0.897	1.09	1.13	0.88
Q9H0Q0	Protein FAM49A OS=Homo sapiens OX	FAM49A	37.312	55.176	17.3	4	4	2	0.949	0.833	0.85	1.092	1.023	0.884	1.139	1.264	1.09	1.07	1.35
Q9NYP9	Protein Mis18-alpha OS=Homo sapiens (	MIS18A	25.863	36.528	16.3	2	2	2	0.947	0.902	1.059	0.956	1.207	1.066	1.01	0.978	1.09	1.23	1.08
P42336	Phosphatidylinositol 4,5-bisphosphate 3-	PIK3CA	124.28	6.6042	0.8	1	2	1	0.9	1.027	1.013	1.087	1.049	0.984	0.965	1.047	1.09	1.06	1.04
Q9NVP2	Histone chaperone ASF1B OS=Homo sa	ASF1B	22.433	11.683	14.4	2	2	2	0.734	0.525	0.589	0.783	1.622	1.327	1.22	1.637	1.09	2.34	2.27
P04899	Guanine nucleotide-binding protein G(i)	GNAI2	40.45	28.129	24.2	7	13	3	0.914	0.891	0.966	1.001	1.113	1.001	1.048	1.067	1.09	1.17	1.17
P31483	Nucleolysin TIA-1 isoform p40 OS=Hom	TIA1	42.963	78.425	22.5	8	13	6	1.011	0.888	1.046	1.023	1.111	1.073	0.96	0.95	1.09	1.15	1.01
Q14677	Clathrin interactor 1 OS=Homo sapiens (	CLINT1	68.259	91.463	22.6	11	13	11	0.958	1.058	1.165	1.031	0.933	1.178	0.881	0.958	1.09	1.05	0.91
Q14139	Ubiquitin conjugation factor E4 A OS=H	UBE4A	122.56	81.844	13.1	11	13	11	0.927	0.967	1	1.063	0.944	1.164	0.981	1.058	1.09	1.11	1.08
P63151	Serine/threonine-protein phosphatase 2A	PPP2R2A	51.691	52.914	14.3	5	10	5	0.985	0.946	1.051	1.052	1.048	0.899	1.079	0.955	1.09	1.01	1.05
P14373	Zinc finger protein RFP OS=Homo sapie	TRIM27	58.489	8.0586	2.7	1	1	1	0.848	0.984	0.919	1.076	0.977	1.36	0.827	1.138	1.09	1.28	1.07
Q9NXG2	THUMP domain-containing protein 1 OS	THUMPD1	39.315	136.75	43.9	14	23	14	0.981	0.945	1.08	1.017	1.135	1.038	0.954	0.89	1.09	1.13	0.96
Q8N1G0	Zinc finger protein 687 OS=Homo sapier	ZNF687	129.53	60.119	6.9	7	7	7	0.999	0.793	0.847	1.104	1.094	0.793	1.051	1.304	1.09	1.05	1.31
Q9H7D7	WD repeat-containing protein 26 OS=Ho	WDR26	72.123	41.675	12.6	6	7	6	0.969	0.96	1.039	1.061	1.02	1.01	0.99	0.953	1.09	1.05	1.01
O75934	Pre-mRNA-splicing factor SPF27 OS=H	BCAS2	26.131	60.851	36.9	7	10	7	0.989	0.877	1.026	1.005	1.029	0.966	1.109	1.012	1.09	1.07	1.14
P49585	Choline-phosphate cytidylyltransferase A	PCYT1A	41.731	60.147	21.3	7	8	7	0.923	1.068	1.139	1.028	0.853	1.095	0.958	0.994	1.09	0.98	0.98
Q6UWL2	Sushi domain-containing protein 1 OS=H	SUSD1	82.709	7.9595	2	1	1	1	0.912	0.98	1.073	0.986	0.956	0.937	1.282	1.006	1.09	1.00	1.21
Q9NRW7	Vacuolar protein sorting-associated prote	VPS45	65.076	38.047	10.4	6	8	6	0.988	0.884	0.999	1.038	1.109	1.199	0.915	0.9	1.09	1.23	0.97
Q6P9B9	Integrator complex subunit 5 OS=Homo s	INTS5	107.99	33.578	5.1	5	5	5	0.964	0.887	1.006	1.008	1.232	1.226	0.869	0.878	1.09	1.33	0.94
Q8N9T8	Protein KRI1 homolog OS=Homo sapien	KRI1	82.597	33.183	7.3	4	4	4	0.918	0.96	1.03	1.013	1.03	0.974	1.173	0.992	1.09	1.07	1.15
Q9Y679	Ancient ubiquitous protein 1 OS=Homo s	AUP1	53.028	28.609	10.7	4	6	4	1.047	0.911	1.125	1.005	0.962	0.977	1.062	0.84	1.09	0.99	0.97
Q8TAF3	WD repeat-containing protein 48 OS=Ho	WDR48	76.21	46.127	11.2	6	7	5	0.954	0.913	0.988	1.043	1.065	1.002	0.964	1.073	1.09	1.11	1.09
Q9H1X3	DnaJ homolog subfamily C member 25 O	DNAJC25	42.403	6.894	2.8	1	1	1	1.004	0.894	1.02	1.044	1.111	0.953	1.008	0.978	1.09	1.09	1.05
P35606	Coatomer subunit beta' OS=Homo sapien	COPB2	102.49	244.77	28.9	20	26	20	0.964	0.972	1.072	1.033	0.984	0.999	1.023	0.959	1.09	1.02	1.02
P07355	Annexin A2 OS=Homo sapiens OX=960	ANXA2	38.604	217.29	49.3	19	46	19	0.93	0.928	0.993	1.027	0.988	0.917	1.105	1.069	1.09	1.03	1.17
Q9Y312	Protein AAR2 homolog OS=Homo sapie	AAR2	43.472	27.563	15.1	4	4	4	0.937	1.003	1.043	1.066	0.954	1.073	0.988	0.992	1.09	1.04	1.02
Q9BTW9	Tubulin-specific chaperone D OS=Homo	TBCD	132.6	57.68	9	7	6	6	0.932	1.01	1.073	1.038	0.943	1.05	0.961	0.941	1.09	1.03	0.98
Q7L4I2	Arginine/serine-rich coiled-coil protein 2	RSRC2	50.559	23.511	5.1	2	2	2	1.071	0.806	1.007	1.033	1.146	0.873	1.067	0.963	1.09	1.08	1.08
P17676	CCAAT/enhancer-binding protein beta O	CEBPB	36.105	31.484	14.5	4	7	4	0.974	0.904	1.052	0.989	1.141	1.169	0.863	1.024	1.09	1.23	1.00
P25787	Proteasome subunit alpha type-2 OS=Ho	PSMA2	25.898	41.371	25.6	4	5	4	0.971	1.012	1.133	1.022	1.05	1.046	0.958	0.901	1.09	1.06	0.94
Q5T4S7	E3 ubiquitin-protein ligase UBR4 OS=H	UBR4	573.83	323.31	11.5	42	50	42	0.96	0.931	1.021	1.034	1.055	0.989	1.013	1.019	1.09	1.08	1.07
Q9NWV4	UPF0587 protein C1orf123 OS=Homo s	C1orf123	18.048	57.604	50	6	6	6	0.953	0.916	0.97	1.061	1.023	0.981	0.988	1.058	1.09	1.07	1.09
P28340	DNA polymerase delta catalytic subunit (	POLD1	123.63	166.38	17.3	16	16	15	0.923	0.856	0.953	0.98	1.102	1.033	1.094	1.101	1.09	1.20	1.23
P61086	Ubiquitin-conjugating enzyme E2 K OS=	UBE2K	22.406	53.453	38	6	10	6	0.985	0.864	0.984	1.025	1.035	0.905	1.125	1.033	1.09	1.05	1.17
P63279	SUMO-conjugating enzyme UBC9 OS=H	UBE2I	18.007	51.322	28.5	6	14	6	0.974	0.922	1.028	1.032	0.961	1.044	0.994	1.018	1.09	1.06	1.06
P06280	Alpha-galactosidase A OS=Homo sapiens	GLA	48.766	31.372	15.2	5	5	5	0.995	0.914	1.02	1.054	1.079	1.094	0.839	0.988	1.09	1.14	0.96
Q01813	ATP-dependent 6-phosphofructokinase,	PFKP	85.595	322.35	43.2	28	65	23	0.942	0.913	0.998	1.017	0.985	0.908	1.227	1.109	1.09	1.02	1.26
Q6ZMI0	Protein phosphatase 1 regulatory subunit	PPP1R21	88.313	66.422	16.4	9	12	9	0.954	0.913	1.001	1.027	0.977	0.951	1.133	1.035	1.09	1.03	1.16
Q99471	Prefoldin subunit 5 OS=Homo sapiens O	PFDN5	17.328	51.158	55.2	7	9	7	1.004	0.98	1.106	1.049	0.994	0.98	0.942	0.907	1.09	0.99	0.93
Q9H6W3	Ribosomal oxygenase 1 OS=Homo sapie	RIOX1	71.085	36.42	5.6	3	3	3	0.942	0.904	0.967	1.038	1.058	0.999	1.084	1.079	1.09	1.11	1.17
O95825	Quinone oxidoreductase-like protein 1 O	CRYZL1	38.696	41.261	17.5	5	7	5	0.957	0.982	1.03	1.076	0.971	1.212	0.872	0.984	1.09	1.13	0.96
Q9UJU6	Drebrin-like protein OS=Homo sapiens (	DBNL	48.207	105.35	34.7	12	15	12	0.956	0.925	0.982	1.061	1.064	0.969	1.025	0.981	1.09	1.08	1.07
Q9Y237	Peptidyl-prolyl cis-trans isomerase NIM	PIN4	13.81	25.185	27.5	3	4	3	0.961	0.897	0.957	1.061	0.983	0.879	1.058	0.966	1.09	1.00	1.09
P38919	Eukaryotic initiation factor 4A-III OS=H	EIF4A3	46.871	130.65	29.7	16	27	12	0.952	0.908	0.997	1.023	1.041	1.02	1.029	1.018	1.09	1.11	1.10

Q6P1M0	Long-chain fatty acid transport protein 4	SLC27A4	72.063	95.82	17.6	8	8	8	0.959	0.936	1.021	1.037	1.044	0.828	1.133	0.967	1.09	0.99	1.11
P22314	Ubiquitin-like modifier-activating enzym	UBA1	117.85	271.22	28.1	24	40	23	0.966	0.969	1.057	1.044	1.018	0.972	1.029	1.032	1.09	1.03	1.07
Q9NTI5	Sister chromatid cohesion protein PDS5	PDS5B	164.67	172.9	13.9	17	21	17	0.952	0.925	1.004	1.034	1.037	1.045	1.108	1.056	1.09	1.11	1.15
P35613	Basigin OS=Homo sapiens OX=9606 GN	BSG	42.2	114.02	21.8	6	17	6	0.904	0.997	1.038	1.026	0.915	1.056	0.943	0.973	1.09	1.04	1.01
Q14997	Proteasome activator complex subunit 4 (	PSME4	211.33	35.911	3.5	5	6	5	0.999	0.928	1.027	1.065	1.024	1.027	0.987	0.937	1.09	1.06	1.00
Q96BI3	Gamma-secretase subunit APH-1A OS=H	APH1A	28.996	6.0167	5.3	1	1	1	0.983	0.888	1	1.031	0.967	1.298	0.873	1.01	1.09	1.21	1.01
A3KN83	Protein strawberry notch homolog 1 OS=	SBNO1	154.31	197.84	21	20	26	20	0.989	0.953	1.132	0.976	0.969	1.2	0.85	0.947	1.09	1.12	0.93
Q6PGP7	Tetratricopeptide repeat protein 37 OS=H	TTC37	175.48	138.12	12.5	15	20	15	0.958	0.974	1.064	1.033	0.97	0.965	1.115	0.946	1.09	1.00	1.07
Q15393	Splicing factor 3B subunit 3 OS=Homo s	SF3B3	135.58	221.63	22	18	30	18	0.977	0.91	1.015	1.033	1.073	0.971	0.996	1.022	1.09	1.08	1.07
Q9H8G2	Caspase activity and apoptosis inhibitor 1	CAAP1	38.367	22.819	12.5	3	3	3	0.958	1.012	1.057	1.081	1.028	0.995	0.883	1.016	1.09	1.03	0.96
Q8IZ52	Chondroitin sulfate synthase 2 OS=Homo	CHPF	85.466	33.678	6.8	5	6	5	0.967	0.889	0.956	1.058	1.302	0.79	1.21	0.844	1.09	1.13	1.11
Q4G148	Glucoside xylosyltransferase 1 OS=Homo	GXYLT1	50.566	10.169	3.6	1	2	1	0.86	1.059	1.101	0.981	1.083	1.381	0.726	0.995	1.08	1.28	0.90
Q96GW9	Methionine--tRNA ligase, mitochondrial	MARS2	66.59	47.995	15.7	7	8	7	0.885	1.072	1.102	1.021	0.953	0.982	1.024	0.905	1.08	0.99	0.99
Q0VDF9	Heat shock 70 kDa protein 14 OS=Homo	HSPA14	54.794	35.241	13	5	7	4	0.964	0.982	1.085	1.026	1.011	1.085	0.962	0.968	1.08	1.08	0.99
Q587I9	Vesicle transport protein SFT2C OS=Ho	SFT2D3	21.789	15.721	15.3	2	3	2	1.048	0.828	1.03	1.005	1.144	0.99	1.012	0.949	1.08	1.14	1.05
Q14161	ARF GTPase-activating protein GIT2 OS	GIT2	84.542	13.248	2.6	2	2	2	0.964	0.999	1.12	1.009	0.96	0.977	1.026	1.024	1.08	0.99	1.04
Q96SK2	Transmembrane protein 209 OS=Homo s	TMEM209	62.921	56.017	15.5	5	5	5	0.967	0.924	1.004	1.046	1.024	1.132	0.921	1.041	1.08	1.14	1.04
Q9BZZ5	Apoptosis inhibitor 5 OS=Homo sapiens	API5	59.004	125.83	27.9	15	21	15	1.012	0.799	0.954	1.009	1.091	0.922	1.091	1.085	1.08	1.11	1.20
Q86X76	Deaminated glutathione amidase OS=Ho	NIT1	35.896	8.794	3.1	1	1	1	0.921	0.893	1.009	0.957	1.141	1.067	1.073	1.09	1.08	1.22	1.19
Q9NZ63	Telomere length and silencing protein 1 h	C9orf78	33.688	59.008	30.8	7	10	7	0.992	0.93	1.031	1.052	1.071	1.021	1.023	0.972	1.08	1.09	1.04
Q96I23	Protein preY, mitochondrial OS=Homo s	PYURF	12.654	5.9705	7	1	1	1	0.99	0.98	1.138	0.997	1.066	1.091	0.8	1.009	1.08	1.09	0.92
Q9UJY1	Heat shock protein beta-8 OS=Homo sap	HSPB8	21.604	45.736	24	5	5	5	0.866	0.843	0.88	0.972	1.087	0.91	1.229	1.293	1.08	1.17	1.48
P21127	Cyclin-dependent kinase 11B OS=Homo	CDK11B	92.619	103.32	15.3	11	16	11	0.923	0.909	0.973	1.012	1.07	0.991	1.07	1.084	1.08	1.13	1.18
Q8N5M4	Tetratricopeptide repeat protein 9C OS=H	TTC9C	20.012	7.4617	8.2	1	1	1	0.981	0.961	1.047	1.057	0.979	1.164	0.8	1.047	1.08	1.10	0.95
Q92754	Transcription factor AP-2 gamma OS=Ho	TFAP2C	49.176	121.3	31.6	11	18	9	0.956	0.941	1.016	1.039	1.106	1.031	1.074	0.969	1.08	1.13	1.08
P61960	Ubiquitin-fold modifier 1 OS=Homo sapi	UFM1	9.1175	14.158	27.1	2	5	2	1.009	0.997	1.118	1.055	0.878	0.979	0.974	1.026	1.08	0.93	1.00
O75362	Zinc finger protein 217 OS=Homo sapier	ZNF217	115.27	72.448	12.7	10	10	9	0.976	0.938	1.021	1.052	1.039	1.024	1.044	0.967	1.08	1.08	1.05
Q06203	Amidophosphoribosyltransferase OS=Ho	PPAT	57.398	110.25	28	14	19	14	0.969	0.982	1.084	1.029	1.035	1.167	0.955	0.919	1.08	1.13	0.96
Q96JJ7	Protein disulfide-isomerase TMX3 OS=H	TMX3	51.871	24.919	5.5	2	2	2	0.954	0.951	1.044	1.019	1.08	1.094	1.001	0.931	1.08	1.14	1.01
Q8NCA5	Protein FAM98A OS=Homo sapiens OX	FAM98A	55.4	73.983	14.3	4	10	4	0.91	1.009	1.066	1.012	0.993	1.082	1.016	0.995	1.08	1.08	1.05
Q9H9T3	Elongator complex protein 3 OS=Homo s	ELP3	62.258	42.323	11.3	5	5	5	0.985	0.864	1.058	0.944	1.098	0.861	1.322	0.966	1.08	1.06	1.24
Q86UU1	Pleckstrin homology-like domain family	PHLDB1	151.16	11.254	1.2	2	2	2	0.958	0.952	1.092	0.976	1.105	1.266	0.825	0.934	1.08	1.24	0.92
Q14166	Tubulin--tyrosine ligase-like protein 12 (	TTLL12	74.403	172.08	28.9	15	21	15	0.931	0.932	1.037	0.98	1.079	1.092	0.905	1.01	1.08	1.17	1.03
Q9H814	Phosphorylated adapter RNA export prot	PHAX	44.402	14.397	6.9	2	3	2	0.96	0.857	0.986	0.981	1.156	1.172	0.983	1.005	1.08	1.28	1.09
O43395	U4/U6 small nuclear ribonucleoprotein P	PRPF3	77.528	141.22	26.1	14	20	14	0.933	0.933	1.003	1.017	1.073	1.19	0.864	1.021	1.08	1.21	1.01
O00299	Chloride intracellular channel protein 1 (	CLIC1	26.922	164.93	61.8	10	29	10	0.981	0.982	1.059	1.066	0.922	0.941	1.091	1.042	1.08	0.95	1.09
P52907	F-actin-capping protein subunit alpha-1 (	CAPZA1	32.922	112.88	52.1	9	18	7	0.99	0.998	1.142	1.01	0.958	0.974	0.993	0.914	1.08	0.97	0.96
Q9HB40	Retinoid-inducible serine carboxypeptida	SCPEP1	50.83	22.274	8	3	3	3	0.994	0.995	1.108	1.045	0.989	1.067	0.918	0.909	1.08	1.03	0.92
Q66K14	TBC1 domain family member 9B OS=Ho	TBC1D9B	140.52	74.584	8.3	10	13	10	0.971	0.901	1.003	1.023	1.061	0.927	1.099	1.024	1.08	1.06	1.13
P11310	Medium-chain specific acyl-CoA dehydro	ACADM	46.588	68.323	16.9	5	8	5	1.012	0.812	0.953	1.021	1.003	0.957	1.167	1.134	1.08	1.07	1.26
Q16401	26S proteasome non-ATPase regulatory s	PSMD5	56.195	95.385	23	8	9	8	0.983	0.904	1.033	1.009	1.025	1.015	1.076	0.996	1.08	1.08	1.10
Q8TBE0	Bromo adjacent homology domain-contai	BAHD1	84.651	9.7623	1.7	1	2	1	1.001	0.887	1.041	1.002	1.108	1.186	0.872	0.954	1.08	1.22	0.97
P32780	General transcription factor IIH subunit 1	GTF2H1	62.031	22.773	6	3	4	3	0.92	0.968	1.011	1.032	1.057	0.99	1.141	1.011	1.08	1.08	1.14
P46379	Large proline-rich protein BAG6 OS=Ho	BAG6	119.41	130.33	14.8	14	18	14	0.893	0.948	0.998	0.994	1.086	1.01	1.066	0.992	1.08	1.14	1.12
Q1ED39	Lysine-rich nucleolar protein 1 OS=Hom	KNOP1	51.588	92.476	27.9	9	9	9	0.847	0.982	1.021	0.958	1.066	1.311	0.909	0.989	1.08	1.30	1.04
Q9UNH7	Sorting nexin-6 OS=Homo sapiens OX=9	SNX6	46.648	128.62	36.5	15	26	14	0.955	0.888	0.979	1.015	1.046	0.904	1.161	1.082	1.08	1.06	1.22
Q9NVM4	Protein arginine N-methyltransferase 7 O	PRMT7	78.458	75.4	9.8	5	5	5	0.942	0.926	0.99	1.031	1.02	1.105	1.013	1.006	1.08	1.14	1.08
P21953	2-oxoisovalerate dehydrogenase subunit b	BCKDHB	43.122	17.477	12.2	3	2	2	0.994	0.888	1.131	0.905	1.147	1.052	1.032	0.981	1.08	1.17	1.07
P08237	ATP-dependent 6-phosphofructokinase, r	PFKM	85.182	173.08	27.7	16	20	13	0.955	1.013	1.068	1.061	0.931	0.943	1.083	0.965	1.08	0.95	1.04
Q9Y224	RNA transcription, translation and transp	RTRAF	28.068	86.338	31.6	11	17	11	0.968	0.976	1.091	1.012	0.985	1.047	0.944	0.955	1.08	1.05	0.98
O43633	Charged multivesicular body protein 2a (	CHMP2A	25.104	59.608	27.9	6	9	6	0.992	0.94	1.035	1.055	0.963	0.898	1.039	1.034	1.08	0.96	1.07
Q15057	Arf-GAP with coiled-coil, ANK repeat ar	ACAP2	88.028	58.528	13.5	7	7	7	0.983	0.95	1.024	1.067	1.083	0.977	1.029	0.991	1.08	1.07	1.05
Q9UHB4	NADPH-dependent diflavin oxidoreducta	NDOR1	66.762	21.569	6.5	2	2	2	1.039	0.872	1.008	1.059	0.933	0.98	1.16	0.923	1.08	1.00	1.09
P41252	Isoleucine--tRNA ligase, cytoplasmic OS	IARS	144.5	209.88	27.9	27	32	26	0.96	0.891	1.004	0.998	1.103	0.991	1.072	1.047	1.08	1.13	1.14
O14730	Serine/threonine-protein kinase RIO3 OS	RIOK3	59.092	28.549	8.9	4	4	4	1.055	0.993	1.19	1.025	0.886	0.937	1.076	0.825	1.08	0.89	0.93
O15066	Kinesin-like protein KIF3B OS=Homo sa	KIF3B	85.124	19.598	4.8	3	3	3	0.948	0.99	1.023	1.073	1.037	0.88	1.093	0.992	1.08	0.99	1.08
P29084	Transcription initiation factor IIE subunit	GTF2E2	33.043	12.584	5.8	2	2	2	0.975	0.939	1.086	0.984	1.042	1.246	0.916	0.897	1.08	1.20	0.95
P35711	Transcription factor SOX-5 OS=Homo sa	SOX5	84.025	12.143	2.6	2	2	1	0.929	0.973	0.981	1.076	0.965	0.931	1.017	1.192	1.08	1.00	1.16
Q96KR1	Zinc finger RNA-binding protein OS=Ho	ZFR	117.01	154.29	20.4	13	16	13	0.917	0.936	1.027	0.977	1.042	1.083	0.966	1.035	1.08	1.15	1.08
P17980	26S proteasome regulatory subunit 6A O	PSMC3	49.203	200.73	56.7	19	35	19	0.96	0.992	1.091	1.02	0.993	1.011	0.976	0.967	1.08	1.03	1.00
P23921	Ribonucleoside-diphosphate reductase la	RRM1	90.069	83.386	14.6	9	14	9	0.921	0.823	0.926	0.96	1.21	1.085	1.058	1.071	1.08	1.32	1.22
O60271	C-Jun-amino-terminal kinase-interacting	SPAG9	146.2	217.15	15.4	14	21	14	0.935	0.973	1.002	1.061	1.009	1.019	1.016	1.059	1.08	1.06	1.09
Q9UQ90	Paraplegin OS=Homo sapiens OX=9606	SPG7	88.234	11.624	2.9	2	2	2	0.953	1.005	1.042	1.075	1.015	0.935	1.016	0.995	1.08	1.00	1.03
Q9BU23	Lipase maturation factor 2 OS=Homo sap	LMF2	79.697	41.713	7.9	4	4	4	0.908	0.978	1.032	1.007	1.103	1.051	1.014	1.041	1.08	1.14	1.09
P15374	Ubiquitin carboxyl-terminal hydrolase iso	UCHL3	26.182	142.66	55.2	9	16	9	0.912	1.085	1.134	1.025	0.942	1.148	0.937	0.965	1.08	1.05	0.95
Q92609	TBC1 domain family member 5 OS=Hom	TBC1D5	89.003	82.33	15.8	10	12	10	0.973	0.963	1.044	1.049	1.017	1.004	0.975	0.949	1.08	1.04	0.99
P06737	Glycogen phosphorylase, liver form OS=I	PYGL	97.147	323.31	42.3	33	60	29	0.962	0.962	1.024	1.056	0.956	0.942	1.08	1.06	1.08	0.99	1.11
Q92759	General transcription factor IIH subunit 4	GTF2H4	52.186	27.875	11	4	4	4	1.012	0.939	1.065	1.044	0.978	1.084	0.916	0.976	1.08	1.06	0.97
P49841	Glycogen synthase kinase-3 beta OS=Ho	GSK3B	46.744	159.94	43.1	11	14	8	0.975	0.94	1.02	1.05	1.03	0.9	1.119	1.023	1.08	1.01	1.12
O95721	Synaptosomal-associated protein 29 OS=	SNAP29	28.97	47.891	28.7	6	6	6	0.924	1.041	1.111	1.013	0.953	1.061	1.002	1	1.08	1.02	1.02
A5PLL7	Transmembrane protein 189 OS=Homo s	TMEM189	31.135	23.094	17	4	4	4	0.906	0.985	1.027	1.017	0.92	1.375	0.768	1.134	1.08	1.21	1.01
Q9UNZ5	Leydig cell tumor 10 kDa protein homolo	C19orf53	10.576	18.539	17.2	3	3	3	0.763	0.832	0.756	0.968	1.126	1.08	1.254	1.503	1.08	1.38	1.73
Q96EK6	Glucosamine 6-phosphate N-acetyltransfe	GNPNAT1	20.749	24.893	16.8	2	2	2	1.006	0.931	1.091	1.002	0.96	1.347	0.771	0.948	1.08	1.19	0.89
Q8NI22	Multiple coagulation factor deficiency pr	MCFD2	16.39	11.113	11.6	1	1	1	1.066	0.835	0.959	1.095	0.979	0.913	1.048	1.037	1.08	1.00	1.10
Q96CS3	FAS-associated factor 2 OS=Homo sapie	FAF2	52.623	128.84	35.3	10	18	10	0.984	0.992	1.105	1.03	0.992	1.011	0.987	0.976	1.08	1.01	0.99
Q9HA65	TBC1 domain family member 17 OS=Ho	TBC1D17	72.727	15.437	2.9	2	3	2	1.012	0.878	0.956	1.086	1.01	1.022	0.962	1.058	1.08	1.08	1.07
Q8N5A5	Zinc finger CCCH-type with G patch dor	ZGPAT	57.358	22.154	7.3	3	3	3	0.959	0.874	1.013	0.967	1.112	1.025	1.056	0.944	1.08	1.17	1.09
Q9NQT5	Exosome complex component RRP40 OS	EXOSC3	29.572	27.984	21.8	4	5	4	0.885	1.011	0.998	1.05	1.006	1.079	1.059	1.057	1.08	1.10	1.12
Q8IUH4	Palmitoyltransferase ZDHHC13 OS=Hor	ZDHHC13	70.86	7.8808	1.9	1	2	1	0.914	0.982	1.12	0.928	0.98	0.992	1.212	1.048	1.08	1.04	1.19
Q96E39	RNA binding motif protein, X-linked-like	RBMXL1	42.141	25.55	36.2	15	35	4	0.984	0.864	0.957	1.039	1.022	0.973	1.114	1.079	1.08	1.08	1.19
Q9UPT8	Zinc finger CCCH domain-containing pro	ZC3H4	140.26	91.525	12.7	10	12	10	0.93	1.036	1.093	1.03	0.994	1.046	0.917	0.921	1.08	1.04	0.93
P61225	Ras-related protein Rap-2b OS=Homo sa	RAP2B	20.504	48.994	36.1	6	9	4	0.972	0.973	1.059	1.041	0.948	0.917	1.147	1.01	1.08	0.96	1.11
Q8NE71	ATP-binding cassette sub-family F memb	ABCF1	95.925	235.85	25.9	18	25	18	0.928	0.955	1.011	1.022	1.04	0.959	1.092	1.027	1.08	1.06	1.13
Q7LBC6	Lysine-specific demethylase 3B OS=Hom	KDM3B	191.58	159.93	14.8	18	20	18	0.98	1.044	1.15	1.035	1.019	1.013	0.914	0.923	1.08	1.00	0.91
Q9Y6X9	MORC family CW-type zinc finger prote	MORC2	117.82	58.872	10.3	9	10	9	0.943	0.86	0.934	1.012	1.084	0.985	1.128	1.096	1.08	1.15	1.23
Q96ST3	Paired amphipathic helix protein Sin3a C	SIN3A	145.17	116.5	11.6	13	14	13	0.967	0.95	1.026	1.043	1.012	0.99	1.079	1.021	1.08	1.04	1.10
Q15459	Splicing factor 3A subunit 1 OS=Homo s	SF3A1	88.885	215.08	28.2	21	31	20	0.988	0.907	1.034	1.011	1.034	1.02	0.984	1.001	1.08	1.08	1.05
O75643	U5 small nuclear ribonucleoprotein 200 k	SNRNP200	244.5	323.31	26.6	47	71	47	0.949	0.921	1.011	1.007	1.075	1.048	0.989	1.013	1.08	1.14	1.07
Q9UNS2	COP9 signalosome complex subunit 3 OS	COPS3	47.873	83.919	24.8	8	12	8	0.971	0.965	1.079	1.01	1.048	1.155	0.932	0.977	1.08	1.14	0.99
Q9C0I1	Myotubularin-related protein 12 OS=Hor	MTMR12	86.147	20.558	6.8	3	3	3	0.903	0.971	0.948	1.074	0.891	1.293	0.932	1.072	1.08	1.17	1.07
Q99426	Tubulin-folding cofactor B OS=Homo sa	TBCB	27.325	78.361	22.1	5	6	5	0.971	0.992	1.102	1.016	1.037	1.105	0.979	0.945	1.08	1.09	0.98
Q7Z434	Mitochondrial antiviral-signaling protein	MAVS	56.527	95.277	27.8	8	10	8	0.986	0.902	1.016	1.021	1.034	1.095	0.965	1.095	1.08	1.13	1.09
Q13162	Peroxiredoxin-4 OS=Homo sapiens OX=	PRDX4	30.54	104.77	43.9	10	20	8	0.947	1.006	1.08	1.027	1	0.977	1.01	1.027	1.08	1.01	1.04
Q16851	UTP--glucose-1-phosphate uridylyltransf	UGP2	56.94	251.53	39.6	19	36	18	0.989	0.991	1.086	1.05	0.94	0.935	1.097	0.974	1.08	0.95	1.05
P00387	NADH-cytochrome b5 reductase 3 OS=H	CYB5R3	34.234	127.53	26.9	6	9	6	0.967	1.003	1.105	1.02	1.031	0.912	1.109	0.969	1.08	0.99	1.05
Q86U86	Protein polybromo-1 OS=Homo sapiens	PBRM1	192.95	73.063	8.4	11	14	11	0.963	0.908	1.032	0.985	1.085	1.201	0.863	0.997	1.08	1.22	0.99
P35658	Nuclear pore complex protein Nup214 O	NUP214	213.62	259.66	16.9	26	32	26	0.952	0.939	1.002	1.036	1.045	0.982	0.986	1.035	1.08	1.07	1.07
Q5T0F9	Coiled-coil and C2 domain-containing pr	CC2D1B	94.223	23.583	3.3	3	4	3	0.956	0.858	0.946	1.009	1.134	0.978	1.171	1.024	1.08	1.16	1.21
P78381	UDP-galactose translocator OS=Homo sa	SLC35A2	41.307	7.3984	2.8	1	2	1	0.934	0.997	0.996	1.085	0.991	1.009	0.969	1.067	1.08	1.04	1.05
Q9H944	Mediator of RNA polymerase II transcrip	MED20	23.222	23.169	14.6	3	4	3	0.966	0.914	0.989	1.037	1.087	1.107	0.973	0.943	1.08	1.17	1.02
P05783	Keratin, type I cytoskeletal 18 OS=Homo	KRT18	48.057	323.31	58.6	25	82	17	0.943	0.951	1.033	1.008	1.004	0.999	1.084	1.048	1.08	1.06	1.13
Q00765	Receptor expression-enhancing protein 5	REEP5	21.493	43.271	15.9	5	6	5	0.981	0.967	1.12	0.979	0.961	0.881	1.178	0.991	1.08	0.95	1.11
P86791	Vacuolar fusion protein CCZ1 homolog (	CCZ1	55.866	56.519	19.7	8	10	8	1.059	0.802	0.979	1.026	1.075	1.002	0.971	1.007	1.08	1.12	1.06
Q9ULU4	Protein kinase C-binding protein 1 OS=H	ZMYND8	131.69	152.05	13.4	13	16	13	0.963	0.994	1.064	1.044	1.044	1.237	0.794	0.961	1.08	1.17	0.90
Q66K89	Transcription factor E4F1 OS=Homo sap	E4F1	83.495	18.407	1.9	1	1	1	0.916	1.031	1.093	1.004	0.961	1.079	1.035	1.003	1.08	1.05	1.05
Q9BY32	Inosine triphosphate pyrophosphatase OS	ITPA	21.445	35.957	32	3	6	3	0.975	0.989	1.081	1.034	0.965	1.041	1.037	0.87	1.08	1.02	0.97
O94903	Pyridoxal phosphate homeostasis protein	PLPBP	30.344	21.004	11.3	3	5	3	0.981	0.944	1.059	1.014	1.179	0.83	1.131	0.981	1.08	1.04	1.10
P06865	Beta-hexosaminidase subunit alpha OS=I	HEXA	60.702	53.19	16.4	7	11	7	1.004	0.921	1.028	1.045	1.043	1.157	0.817	0.966	1.08	1.14	0.93
Q9UIF9	Bromodomain adjacent to zinc finger dor	BAZ2A	211.2	110.31	9.6	15	15	15	0.969	0.906	0.982	1.037	1.12	0.977	0.999	1.029	1.08	1.12	1.08
O14818	Proteasome subunit alpha type-7 OS=Ho	PSMA7	27.887	116.46	46.8	9	18	9	0.958	0.987	1.043	1.051	0.97	1	0.995	0.947	1.08	1.01	1.00
Q8IV08	Phospholipase D3 OS=Homo sapiens OX	PLD3	54.705	28.402	7.6	4	4	4	0.949	0.957	1.082	0.97	1.094	1.303	0.77	0.999	1.08	1.26	0.93
P33316	Deoxyuridine 5'-triphosphate nucleotidoh	DUT	26.563	115.6	43.7	9	12	9	0.938	0.891	0.973	0.996	1.091	0.951	1.142	1.126	1.08	1.12	1.24
Q13526	Peptidyl-prolyl cis-trans isomerase NIMA	PIN1	18.243	26.254	30.7	3	5	3	0.982	1.043	1.147	1.033	1.022	0.936	0.98	0.899	1.08	0.97	0.93
P35813	Protein phosphatase 1A OS=Homo sapie	PPM1A	42.447	36.555	19.6	6	6	5	0.941	1.006	1.079	1.017	0.974	0.976	1.084	0.836	1.08	1.00	0.99
Q96RQ1	Endoplasmic reticulum-Golgi intermediat	ERGIC2	42.548	18.797	5.8	2	2	2	0.948	0.947	1.014	1.026	0.964	0.959	1.164	1.055	1.08	1.01	1.17
Q15029	116 kDa U5 small nuclear ribonucleoprot	EFTUD2	109.43	314.59	35.9	29	42	27	0.968	0.928	1.026	1.015	1.044	1.039	1.037	1.004	1.08	1.10	1.08
Q9Y696	Chloride intracellular channel protein 4 (	CLIC4	28.772	41.462	21.3	5	11	5	0.974	1.04	1.087	1.081	0.865	0.9	1.035	0.974	1.08	0.88	1.00
O14647	Chromodomain-helicase-DNA-binding pr	CHD2	211.34	36.121	5	8	8	5	1.002	0.909	1.004	1.053	0.987	0.97	1.029	1.06	1.08	1.02	1.09
P29966	Myristoylated alanine-rich C-kinase subs	MARCKS	31.554	75.106	23.5	5	5	5	0.982	1.008	1.036	1.106	0.811	0.77	1.151	1.066	1.08	0.79	1.11
P68400	Casein kinase II subunit alpha OS=Homo	CSNK2A1	45.143	103.02	30.4	10	14	10	0.974	0.951	1.019	1.053	1.038	1.074	0.944	0.981	1.08	1.10	1.00
P55010	Eukaryotic translation initiation factor 5 (	EIF5	49.222	69.414	27.4	11	9	9	0.971	1.033	1.049	1.108	0.943	1.029	1.092	0.944	1.08	0.98	1.02
O14579	Coatomer subunit epsilon OS=Homo sap	COPE	34.482	96.604	34.1	7	12	7	0.993	0.921	1.022	1.038	0.964	1.217	0.85	0.997	1.08	1.14	0.96
P48634	Protein PRRC2A OS=Homo sapiens OX	PRRC2A	228.86	125.63	9.7	15	17	15	0.981	0.951	1.053	1.026	1.032	1.049	1.018	0.975	1.08	1.08	1.03
Q9BYJ9	YTH domain-containing family protein 1	YTHDF1	60.873	35.94	15	7	9	4	0.969	0.977	1.096	0.998	1.042	1.172	0.875	0.851	1.08	1.14	0.89
Q9UJV9	Probable ATP-dependent RNA helicase I	DDX41	69.837	60.628	14.3	8	11	8	0.997	0.884	0.989	1.035	1.049	0.953	1.092	1.006	1.08	1.06	1.12
P14550	Alcohol dehydrogenase [NADP(+)] OS=I	AKR1A1	36.573	100.39	42.8	10	12	10	0.976	0.985	1.059	1.051	0.972	0.958	1.048	0.898	1.08	0.98	0.99
P31689	DnaJ homolog subfamily A member 1 OS	DNAJA1	44.868	102.08	30.2	9	13	9	0.948	0.935	1.004	1.022	1.069	1.098	0.939	0.973	1.08	1.15	1.02
Q3KQU3	MAP7 domain-containing protein 1 OS=	MAP7D1	92.819	41.329	8.6	5	5	5	0.888	0.969	0.967	1.031	1.115	1.188	0.928	1.133	1.08	1.24	1.11
P63104	14-3-3 protein zeta/delta OS=Homo sapie	YWHAZ	27.745	115.67	37.1	11	37	7	0.991	0.998	1.082	1.058	0.904	0.955	1.069	0.95	1.08	0.93	1.02
O43290	U4/U6.U5 tri-snRNP-associated protein 1	SART1	90.254	257.86	31.5	19	26	19	0.935	0.951	0.991	1.038	1.047	1.15	0.879	0.988	1.08	1.16	0.99
Q16531	DNA damage-binding protein 1 OS=Hom	DDB1	126.97	323.31	36	34	60	34	0.979	0.952	1.055	1.022	1.007	1.036	0.96	0.941	1.08	1.06	0.98
Q96AX1	Vacuolar protein sorting-associated prote	VPS33A	67.61	29.438	10.4	5	5	5	0.964	0.929	1.033	1.003	1.102	1.251	0.95	0.96	1.08	1.24	1.01
P21291	Cysteine and glycine-rich protein 1 OS=I	CSRP1	20.567	209.17	64.8	10	24	10	0.946	1	1.06	1.033	0.952	0.932	1.11	1.01	1.08	0.97	1.09
P53634	Dipeptidyl peptidase 1 OS=Homo sapien	CTSC	51.853	76.618	20.5	6	9	6	0.986	0.909	1.054	0.984	1.098	0.942	0.989	0.962	1.08	1.08	1.03
Q8N163	Cell cycle and apoptosis regulator protein	CCAR2	102.9	129.65	20.4	14	20	14	0.968	0.914	1.009	1.015	1.054	1.045	0.992	0.996	1.08	1.12	1.06
P80303	Nucleobindin-2 OS=Homo sapiens OX=9	NUCB2	50.222	63.21	23.8	8	11	8	0.935	0.947	1.043	0.981	1.032	1.034	1.038	1.033	1.08	1.10	1.10
O75122	CLIP-associating protein 2 OS=Homo sa	CLASP2	141.13	97.646	13.4	14	14	14	0.937	0.985	1.016	1.051	0.978	1.159	0.91	0.984	1.08	1.11	0.99
Q9BXP5	Serrate RNA effector molecule homolog (	SRRT	100.67	200.02	22	18	26	18	0.95	0.946	1.017	1.022	1.032	1.08	1.009	1.001	1.08	1.11	1.06
P35556	Fibrillin-2 OS=Homo sapiens OX=9606	FBN2	314.77	182.24	7.3	18	21	18	0.933	0.857	0.912	1.013	1.127	1.056	1.044	1.137	1.08	1.22	1.22
Q99436	Proteasome subunit beta type-7 OS=Hom	PSMB7	29.965	45.818	19.5	6	11	6	0.991	0.982	1.082	1.039	0.986	0.935	1.052	0.965	1.08	0.97	1.02
P10155	60 kDa SS-A/Ro ribonucleoprotein OS=I	TROVE2	60.67	53.92	15.1	8	9	8	0.976	0.893	1.013	0.996	1.036	0.965	1.055	1.088	1.07	1.07	1.15
P49750	YLP motif-containing protein 1 OS=Hom	YLPM1	241.64	179.19	10.2	15	18	15	0.968	0.995	1.067	1.043	1.04	1.158	0.916	0.959	1.07	1.12	0.96
O75083	WD repeat-containing protein 1 OS=Hom	WDR1	66.193	218.85	37.8	15	32	14	0.931	1.073	1.093	1.061	0.916	1.21	0.895	0.911	1.07	1.06	0.90
O95400	CD2 antigen cytoplasmic tail-binding pro	CD2BP2	37.646	50.167	19.9	5	8	5	0.98	0.907	1.017	1.011	1.147	1.024	0.955	0.965	1.07	1.15	1.02
Q9H939	Proline-serine-threonine phosphatase-inte	PSTPIP2	38.858	42.108	17.1	5	6	5	0.904	0.943	1.019	0.966	1.086	1.136	1.003	1.064	1.07	1.20	1.12
Q96Q11	CCA tRNA nucleotidyltransferase 1, mito	TRNT1	50.127	52.841	25.3	8	7	7	0.969	0.92	0.961	1.069	1.01	0.93	0.993	1.086	1.07	1.03	1.10
Q9P0L0	Vesicle-associated membrane protein-ass	VAPA	27.893	77.586	36.9	7	11	6	0.872	1.178	1.187	1.016	0.812	1.337	0.816	0.938	1.07	1.05	0.86
P55854	Small ubiquitin-related modifier 3 OS=H	SUMO3	11.637	19.304	20.4	2	4	1	1.013	0.986	1.072	1.076	0.954	1.27	0.76	0.856	1.07	1.11	0.81
Q52LJ0	Protein FAM98B OS=Homo sapiens OX	FAM98B	37.19	54.004	23	5	5	4	0.972	0.988	1.073	1.033	0.961	0.968	1.091	0.957	1.07	0.98	1.04
Q14739	Lamin-B receptor OS=Homo sapiens OX	LBR	70.702	69.656	9.6	5	6	5	0.975	1.014	1.134	1.003	0.872	0.968	0.926	0.789	1.07	0.93	0.86
Q86V21	Acetoacetyl-CoA synthetase OS=Homo s	AACS	75.143	16.511	4.2	3	3	3	0.918	0.984	1.101	0.942	1.133	0.965	0.993	1.133	1.07	1.10	1.12
P19474	E3 ubiquitin-protein ligase TRIM21 OS=	TRIM21	54.169	80.773	16.2	6	7	6	0.927	1.002	1.018	1.054	1.011	0.93	1.103	0.984	1.07	1.01	1.08
Q6UUV7	CREB-regulated transcription coactivator	CRTC3	66.958	28.01	5.3	3	3	3	0.904	0.946	0.91	1.077	1.05	1.132	0.924	1.136	1.07	1.18	1.11
Q147X3	N-alpha-acetyltransferase 30 OS=Homo s	NAA30	39.319	17.036	13.3	3	3	3	0.975	0.976	0.995	1.1	1.019	0.923	1.095	0.91	1.07	1.00	1.03
P49848	Transcription initiation factor TFIID subu	TAF6	72.668	79.969	13.9	7	9	7	0.964	0.987	1.065	1.03	1.009	1.017	1.032	1.023	1.07	1.04	1.05
Q969X6	U3 small nucleolar RNA-associated prote	UTP4	76.889	81.349	13.1	7	9	7	0.986	0.938	1.046	1.02	1.181	1.109	0.898	0.898	1.07	1.19	0.93

Q08257	Quinone oxidoreductase OS=Homo sapie	CRYZ	35.206	132.14	42.6	10	19	10	0.971	0.994	1.053	1.057	0.967	0.989	1.013	0.953	1.07	1.00	1.00
Q14156	Protein EFR3 homolog A OS=Homo sap	EFR3A	92.923	37.212	7.8	6	6	6	0.929	0.971	1.013	1.027	1.037	1.028	1.027	0.994	1.07	1.09	1.06
Q93008	Probable ubiquitin carboxyl-terminal hyd	USP9X	292.28	140.03	9.9	18	21	18	0.947	0.955	1.007	1.035	1.019	0.997	1.02	0.998	1.07	1.06	1.06
P49257	Protein ERGIC-53 OS=Homo sapiens O	LMAN1	57.548	112.62	16.9	7	9	7	0.997	0.905	1.027	1.015	1.051	1.04	0.986	0.975	1.07	1.10	1.03
P07711	Cathepsin L1 OS=Homo sapiens OX=96	CTSL	37.564	48.029	20.7	6	10	6	0.976	0.929	0.953	1.092	1.047	1.052	1.074	1.065	1.07	1.10	1.12
Q00169	Phosphatidylinositol transfer protein alpl	PITPNA	31.806	44.969	28.5	7	7	6	0.968	0.91	0.987	1.029	1.052	1.037	0.974	1.007	1.07	1.11	1.05
Q8TF74	WAS/WASL-interacting protein family n	WIPF2	46.288	69.834	21.8	7	10	7	0.983	0.938	1.01	1.052	1.023	1.009	1.015	1.012	1.07	1.06	1.06
Q9BV19	Uncharacterized protein C1orf50 OS=Ho	C1orf50	21.876	7.537	8	1	1	1	1.028	1.061	1.096	1.146	0.936	0.954	0.883	0.814	1.07	0.90	0.81
P26640	Valine--tRNA ligase OS=Homo sapiens (	VARS	140.47	236.55	24.8	26	37	24	0.988	0.967	1.077	1.021	1.013	1.036	0.931	0.936	1.07	1.05	0.95
O94888	UBX domain-containing protein 7 OS=H	UBXN7	54.862	46.319	16.2	6	7	6	0.912	1.086	1.084	1.06	0.811	1.073	1.011	0.93	1.07	0.94	0.97
Q96I24	Far upstream element-binding protein 3 (	FUBP3	61.64	168.36	34.6	16	24	14	0.989	0.983	1.062	1.054	1.008	0.929	1.038	0.954	1.07	0.98	1.01
Q9HBH1	Peptide deformylase, mitochondrial OS=	PDF	27.013	31.248	24.3	3	3	3	0.962	0.95	1.068	0.983	1.021	1.07	1.052	0.993	1.07	1.09	1.07
Q9NUJ1	Mycophenolic acid acyl-glucuronide este	ABHD10	33.932	50.795	26.1	6	7	5	0.946	0.967	1.048	1.004	1.018	1.051	1.073	1.002	1.07	1.08	1.08
Q70J99	Protein unc-13 homolog D OS=Homo sa	UNC13D	123.28	40.027	7	6	7	6	0.986	0.862	1.026	0.956	0.987	0.94	1.264	1.077	1.07	1.04	1.27
Q08623	Pseudouridine-5'-phosphatase OS=Homo	PUDP	25.249	16.709	5.3	1	2	1	0.964	0.971	1.059	1.016	1.051	1.098	0.924	0.99	1.07	1.11	0.99
O43447	Peptidyl-prolyl cis-trans isomerase H OS	PPIH	19.208	37.53	27.1	6	5	5	0.984	0.958	1.05	1.032	1.004	0.935	0.907	0.975	1.07	1.00	0.97
Q9Y6H1	Coiled-coil-helix-coiled-coil-helix domai	CHCHD2	15.512	25.176	24.5	2	3	2	0.824	1.189	1.085	1.073	0.866	1.073	1.023	1.013	1.07	0.96	1.01
P61923	Coatomer subunit zeta-1 OS=Homo sapie	COPZ1	20.198	35.075	22.6	3	6	3	0.985	0.976	1.086	1.016	0.992	1.045	0.981	0.96	1.07	1.04	0.99
Q9UHJ6	Sedoheptulokinase OS=Homo sapiens O	SHPK	51.49	30.453	10.9	3	3	3	0.992	0.886	0.998	1.015	1.144	0.952	1.1	1.007	1.07	1.12	1.12
Q5FBB7	Shugoshin 1 OS=Homo sapiens OX=960	SGO1	64.189	17.508	4.8	2	2	2	0.791	0.924	0.885	0.953	1.069	1.059	1.172	1.416	1.07	1.24	1.51
Q9HCG8	Pre-mRNA-splicing factor CWC22 homo	CWC22	105.47	74.086	10.8	9	9	9	1	0.94	1.054	1.025	1.04	1.062	0.888	0.932	1.07	1.08	0.94
Q8TEA8	D-aminoacyl-tRNA deacylase 1 OS=Hon	DTD1	23.423	17.982	14.4	3	3	3	0.998	1.014	1.09	1.066	0.922	0.901	1.113	0.898	1.07	0.91	1.00
P55196	Afadin OS=Homo sapiens OX=9606 GN	AFDN	206.8	297.31	17.7	25	35	24	0.96	0.955	1	1.052	1.001	1.007	1.011	1.02	1.07	1.05	1.06
Q05519	Serine/arginine-rich splicing factor 11 O	SRSF11	53.542	31.003	11	4	8	4	0.945	1.026	1.018	1.094	1.042	1.07	1.018	1.021	1.07	1.07	1.03
Q9UJ14	Glutathione hydrolase 7 OS=Homo sapie	GGT7	70.466	16	3.5	2	2	2	0.903	0.933	1.001	0.966	0.993	1.195	1.256	0.902	1.07	1.19	1.18
Q9NPJ3	Acyl-coenzyme A thioesterase 13 OS=Hc	ACOT13	14.96	29.113	25	3	6	3	1.001	0.872	0.942	1.064	1.021	1.177	0.937	0.993	1.07	1.17	1.03
Q6GYQ0	Ral GTPase-activating protein subunit al	RALGAPA	229.83	10.807	0.9	2	2	2	0.909	0.995	1.015	1.024	1.104	1.137	0.905	1.022	1.07	1.18	1.01
P46531	Neurogenic locus notch homolog protein	NOTCH1	272.5	27.408	2	4	5	4	0.938	1.009	1.148	0.937	1.144	1.088	1.036	0.842	1.07	1.15	0.96
Q05086	Ubiquitin-protein ligase E3A OS=Homo	UBE3A	100.69	48.724	9.1	6	9	6	1.027	0.908	1.034	1.038	1.031	0.988	0.913	0.996	1.07	1.04	0.99
Q86WR7	Proline and serine-rich protein 2 OS=Ho	PROSER2	45.801	6.894	2.5	1	1	1	0.954	0.925	0.929	1.083	1.019	0.935	1.118	1.064	1.07	1.04	1.16
Q13439	Golgin subfamily A member 4 OS=Homo	GOLGA4	261.14	168.47	10.6	22	26	21	1.019	0.993	1.132	1.022	0.981	0.956	0.979	0.922	1.07	0.96	0.94
Q15363	Transmembrane emp24 domain-containin	TMED2	22.761	8.0438	6	1	1	1	0.945	1.046	1.085	1.046	0.945	1.245	0.776	0.985	1.07	1.10	0.88
Q15126	Phosphomevalonate kinase OS=Homo sa	PMVK	21.995	29.478	20.3	3	4	3	0.988	0.98	1.065	1.041	1.078	0.952	0.996	1.015	1.07	1.03	1.02
Q96BP3	Peptidylprolyl isomerase domain and WI	PPWD1	73.574	79.342	16.6	9	11	9	0.945	0.956	1.011	1.023	1.167	1.226	0.926	0.961	1.07	1.26	0.99
Q08J23	tRNA (cytosine(34)-C(5))-methyltransfer	NSUN2	86.47	243.78	39.5	26	40	26	0.985	0.947	1.037	1.03	1.022	0.972	1.012	0.975	1.07	1.03	1.03
P29401	Transketolase OS=Homo sapiens OX=96	TKT	67.877	323.31	38.2	22	65	22	0.957	0.908	0.961	1.034	1.045	0.996	1.074	1.064	1.07	1.09	1.15
P80723	Brain acid soluble protein 1 OS=Homo s	BASP1	22.693	242.5	74.4	15	36	15	0.94	0.954	1.012	1.014	1.021	1.01	1.064	1.067	1.07	1.07	1.13
Q7Z3K3	Pogo transposable element with ZNF dor	POGZ	155.34	68.76	5.9	6	7	6	0.98	0.931	1.073	0.971	1.117	0.843	1.105	1.072	1.07	1.03	1.14
P25786	Proteasome subunit alpha type-1 OS=Ho	PSMA1	29.555	101.92	43	10	22	10	0.991	0.95	1.052	1.024	1.026	0.985	1.028	0.965	1.07	1.04	1.03
O95685	Protein phosphatase 1 regulatory subunit	PPP1R3D	32.559	50.161	20.7	6	7	6	0.961	0.981	1.052	1.025	1.003	1.007	1.029	1.004	1.07	1.04	1.05
P67870	Casein kinase II subunit beta OS=Homo	CSNK2B	24.942	70.414	45.1	7	10	6	0.948	0.966	1.016	1.031	1.026	1.085	1.006	0.965	1.07	1.10	1.03
Q15154	Pericentriolar material 1 protein OS=Hor	PCM1	228.54	101.49	8.3	15	16	15	0.984	0.959	1.063	1.015	1.024	1.031	0.942	0.961	1.07	1.06	0.98
P50570	Dynamin-2 OS=Homo sapiens OX=9606	DNM2	98.063	115.74	22.9	15	20	12	0.95	0.966	1.052	0.997	0.992	0.906	1.122	0.984	1.07	0.99	1.10
P28288	ATP-binding cassette sub-family D meml	ABCD3	75.475	60.208	9.6	5	6	5	0.951	0.923	1.026	0.978	1.019	1.23	0.812	1.041	1.07	1.20	0.99
A6NIH7	Protein unc-119 homolog B OS=Homo s	UNC119B	28.136	15.318	11.2	2	2	2	0.999	0.877	0.991	1.015	1.158	0.84	1.087	1.076	1.07	1.07	1.15
O00231	26S proteasome non-ATPase regulatory s	PSMD11	47.463	153.8	35.1	12	22	12	0.926	1.055	1.079	1.039	0.926	1.165	0.893	0.947	1.07	1.06	0.93
Q8N8A6	ATP-dependent RNA helicase DDX51 O	DDX51	72.456	43.264	14	6	7	6	0.986	0.985	1.108	0.999	1.029	0.994	0.962	0.937	1.07	1.03	0.96
P51397	Death-associated protein 1 OS=Homo sa	DAP	11.164	12.702	15.7	2	4	2	1	0.788	0.996	0.915	1.093	1.031	1.143	0.959	1.07	1.19	1.18
Q9GZS3	WD repeat-containing protein 61 OS=Ho	WDR61	33.58	93.625	27.2	7	10	7	0.968	0.937	1.026	1.01	1.041	0.985	1.097	0.984	1.07	1.06	1.09
Q96BW9	Phosphatidate cytidylyltransferase, mitoc	TAMM41	51.066	16.567	8	2	2	2	1.007	1.015	1.109	1.052	0.877	0.954	1.072	0.923	1.07	0.91	0.99
P08842	Steryl-sulfatase OS=Homo sapiens OX=	STS	65.492	65.689	16.3	9	15	8	0.928	0.949	0.995	1.011	1.037	1.022	1.162	1.094	1.07	1.10	1.20
O75818	Ribonuclease P protein subunit p40 OS=	RPP40	41.833	26.55	14	3	3	3	0.938	0.867	0.911	1.018	1.212	1.071	0.979	1.094	1.07	1.26	1.15
Q13144	Translation initiation factor eIF-2B subu	EIF2B5	80.379	18.435	6	3	3	3	0.957	1.052	1.115	1.032	1.03	1.281	0.729	0.871	1.07	1.15	0.80
Q70CQ2	Ubiquitin carboxyl-terminal hydrolase 34	USP34	404.23	30.178	1.5	5	5	5	0.984	0.912	0.974	1.052	1.136	0.998	0.927	0.961	1.07	1.13	1.00
Q13459	Unconventional myosin-IXb OS=Homo s	MYO9B	243.4	122.29	11.5	18	21	18	0.95	0.92	0.987	1.011	0.986	1.043	0.983	1.053	1.07	1.09	1.09
P25490	Transcriptional repressor protein YY1 O	YY1	44.712	53.827	19.8	6	8	6	0.955	0.93	0.953	1.061	0.989	0.994	0.995	1.043	1.07	1.05	1.08
Q06787	Synaptic functional regulator FMR1 OS=	FMR1	71.174	75.904	17.2	10	11	9	0.926	0.962	0.984	1.033	1.04	1.076	1.017	1.046	1.07	1.12	1.09
O75821	Eukaryotic translation initiation factor 3	EIF3G	35.611	100.14	33.1	10	13	10	1.02	0.971	1.032	1.095	0.972	1.01	0.943	1.011	1.07	1.00	0.98
P98175	RNA-binding protein 10 OS=Homo sapie	RBM10	103.53	108.21	12.8	10	10	8	0.95	0.955	1.041	0.994	1.132	1.134	0.919	0.952	1.07	1.19	0.98
O00488	Zinc finger protein 593 OS=Homo sapier	ZNF593	15.199	33.267	28.4	2	2	2	0.964	1.366	1.352	1.137	0.719	0.891	1.017	0.824	1.07	0.69	0.79
Q09161	Nuclear cap-binding protein subunit 1 O	NCBP1	91.838	150.8	25.2	14	22	14	0.979	0.928	0.985	1.052	1.04	1.053	1.033	1.047	1.07	1.10	1.09
P19784	Casein kinase II subunit alpha' OS=Hom	CSNK2A2	41.213	140.49	51.1	14	17	13	0.933	0.99	1.044	1.01	0.994	1.222	0.935	0.996	1.07	1.15	1.00
Q9ULR0	Pre-mRNA-splicing factor ISY1 homolog	ISY1	32.992	84.131	15.4	4	5	4	0.933	0.946	0.964	1.043	0.977	1.083	0.981	1.05	1.07	1.10	1.08
P08047	Transcription factor Sp1 OS=Homo sapie	SP1	80.692	44.268	9	5	6	4	0.984	0.94	1.03	1.025	1.013	0.964	1.18	1.056	1.07	1.03	1.16
Q9H0X4	Protein FAM234A OS=Homo sapiens O	FAM234A	59.659	6.7231	2.4	1	1	1	1.019	0.88	1.009	1.019	1.139	1.115	0.888	0.954	1.07	1.19	0.97
P27348	14-3-3 protein theta OS=Homo sapiens C	YWHAQ	27.764	158.99	41.2	13	42	9	0.975	1.015	1.07	1.055	0.966	0.979	0.924	0.941	1.07	0.98	0.94
P56211	cAMP-regulated phosphoprotein 19 OS=	ARPP19	12.323	57.686	42.9	5	8	4	0.968	1.067	1.116	1.057	0.844	1.01	1.046	0.894	1.07	0.91	0.95
Q9H0E9	Bromodomain-containing protein 8 OS=I	BRD8	135.33	19.801	3.2	3	3	3	0.984	0.889	0.984	1.016	1.087	1.203	0.905	0.988	1.07	1.22	1.01
O75554	WW domain-binding protein 4 OS=Hom	WBP4	42.506	13.892	7.4	2	3	2	0.913	0.991	1.007	1.026	1.087	0.946	1.054	1.084	1.07	1.07	1.12
Q13596	Sorting nexin-1 OS=Homo sapiens OX=	SNX1	59.069	88.304	19	9	15	7	0.96	0.963	1.03	1.023	1.012	0.983	1.093	0.991	1.07	1.04	1.08
Q9Y3D8	Adenylate kinase isoenzyme 6 OS=Homo	AK6	20.061	16.762	15.1	2	3	2	1.006	0.92	0.978	1.078	1.047	1.191	0.803	0.955	1.07	1.16	0.91
Q14790	Caspase-8 OS=Homo sapiens OX=9606	CASP8	55.391	21.833	6.9	3	3	3	0.981	0.945	1.009	1.047	1.059	0.908	1.045	1.038	1.07	1.02	1.08
O94979	Protein transport protein Sec31A OS=Ho	SEC31A	133.01	110.8	14.1	12	19	12	0.966	1.037	1.096	1.042	1.006	1.073	0.889	0.933	1.07	1.04	0.91
Q10713	Mitochondrial-processing peptidase subu	PMPCA	58.252	98.91	22.9	11	16	11	0.984	0.945	1.034	1.025	0.995	0.992	0.979	1.009	1.07	1.03	1.03
Q8IVW6	AT-rich interactive domain-containing pr	ARID3B	60.636	46.104	11.1	6	6	6	1.019	0.944	1.094	1.001	1.105	0.938	1.098	0.926	1.07	1.04	1.03
Q9UH62	Armadillo repeat-containing X-linked pro	ARMCX3	42.5	33.113	14	4	5	4	0.953	0.847	1	0.921	1.173	1.131	0.983	1.133	1.07	1.28	1.18
Q15007	Pre-mRNA-splicing regulator WTAP OS	WTAP	44.243	79.89	33.3	10	13	10	0.984	0.965	1.059	1.021	1.024	1.01	0.937	0.974	1.07	1.04	0.98
Q9Y3E5	Peptidyl-tRNA hydrolase 2, mitochondri	PTRH2	19.193	8.3325	10.6	1	1	1	0.739	1.344	1.118	1.105	0.64	1.563	0.794	0.894	1.07	1.06	0.81
Q9NX55	Huntingtin-interacting protein K OS=Ho	HYPK	14.665	46.597	40.3	5	8	5	1.024	0.911	1.098	0.967	1.006	0.961	1.092	1.016	1.07	1.02	1.09
Q96AC1	Fermitin family homolog 2 OS=Homo sa	FERMT2	77.86	137.59	20.4	12	16	11	0.999	0.983	1.104	1.011	0.997	0.946	1.088	0.894	1.07	0.98	1.00
O94874	E3 UFM1-protein ligase 1 OS=Homo sa	UFL1	89.594	75.728	14.5	11	14	11	0.968	0.926	1.03	0.991	1.036	0.946	1.026	1.05	1.07	1.05	1.10
Q9BVQ7	Spermatogenesis-associated protein 5-lik	SPATA5L1	80.709	52.256	14.1	7	8	7	0.958	0.922	0.975	1.031	1.094	0.966	1.039	1.104	1.07	1.10	1.14
Q9NTK5	Obg-like ATPase 1 OS=Homo sapiens O	OLA1	44.743	133.49	30.3	11	22	11	1.012	0.959	1.048	1.055	0.982	1.009	0.946	0.923	1.07	1.01	0.95
O76024	Wolframin OS=Homo sapiens OX=9606	WFS1	100.29	96.419	14.8	10	12	10	0.981	1.039	1.118	1.037	0.954	0.911	1.083	0.897	1.07	0.92	0.98
P25098	Beta-adrenergic receptor kinase 1 OS=H	GRK2	79.573	19.301	6	3	4	3	0.993	0.986	1.089	1.022	1.033	1.048	0.92	0.955	1.07	1.05	0.95
Q8IWZ8	SURP and G-patch domain-containing pr	SUGP1	72.47	78.567	29.8	13	13	13	0.97	0.906	1.024	0.977	1.064	0.968	1.056	1.038	1.07	1.08	1.12
P09471	Guanine nucleotide-binding protein G(o)	GNAO1	40.05	15.098	10.2	3	8	1	0.949	0.842	0.978	0.932	1.21	1.267	0.959	1.009	1.07	1.38	1.10
P09884	DNA polymerase alpha catalytic subunit	POLA1	165.91	34.261	3.9	5	6	5	0.977	0.86	1.019	0.94	1.234	1.078	1.047	0.957	1.07	1.26	1.09
Q9BRP8	Partner of Y14 and mago OS=Homo sapi	PYM1	22.655	67.7	32.8	4	7	4	0.982	0.916	1.02	1.004	1.062	0.96	1.087	0.984	1.07	1.07	1.09
Q14147	Probable ATP-dependent RNA helicase I	DHX34	128.12	27.331	3.6	3	4	3	0.948	0.878	0.978	0.969	1.206	0.882	1.179	1.075	1.07	1.14	1.23
P35250	Replication factor C subunit 2 OS=Homo	RFC2	39.157	110.51	29.7	8	12	8	0.932	0.897	0.967	0.983	1.136	0.961	1.132	1.062	1.07	1.15	1.20
Q7Z7E8	Ubiquitin-conjugating enzyme E2 Q1 OS	UBE2Q1	46.126	43.669	14	6	6	6	1.004	1.19	1.292	1.047	0.804	1.202	0.901	0.614	1.07	0.91	0.69
O75150	E3 ubiquitin-protein ligase BRE1B OS=	RNF40	113.65	187.71	22.9	17	22	16	0.994	0.928	1.03	1.019	1.043	0.948	1.056	0.956	1.07	1.04	1.05
Q8NEY8	Periphilin-1 OS=Homo sapiens OX=960	PPHLN1	52.736	12.972	4.1	2	3	2	1.025	0.962	1.037	1.081	0.966	1.159	0.759	0.991	1.07	1.07	0.88
O15397	Importin-8 OS=Homo sapiens OX=9606	IPO8	119.94	55.057	8	7	9	6	1.044	0.989	1.143	1.024	1.024	1.225	0.721	1.081	1.07	1.11	0.89
O75396	Vesicle-trafficking protein SEC22b OS=	SEC22B	24.593	104.17	43.3	9	18	9	0.954	0.935	0.993	1.02	1.034	0.955	1.134	1.069	1.07	1.05	1.17
Q9UKS6	Protein kinase C and casein kinase subst	PACSIN3	48.486	111.62	36.1	12	15	12	0.924	0.95	0.987	1.01	1.01	1.032	1.08	1.119	1.07	1.09	1.17
Q6ZVM7	TOM1-like protein 2 OS=Homo sapiens	TOM1L2	55.556	10.712	3.6	2	2	2	0.909	0.874	0.989	0.911	1.201	0.94	1.265	1.099	1.07	1.20	1.33
Q96B49	Mitochondrial import receptor subunit T(	TOMM6	8.0019	8.5115	18.9	1	4	1	0.957	1.048	1.119	1.017	0.973	0.926	1.042	0.997	1.07	0.95	1.02
Q13263	Transcription intermediary factor 1-beta (	TRIM28	88.549	323.31	49.1	23	46	23	0.965	0.952	1.047	0.995	1.01	1.02	0.986	1.022	1.07	1.06	1.05
Q9BV20	Methylthioribose-1-phosphate isomerase	MRI1	39.149	93.839	20.6	5	10	5	0.923	0.995	0.998	1.045	0.978	1.006	1.009	1.018	1.07	1.03	1.06
P04066	Tissue alpha-L-fucosidase OS=Homo sap	FUCA1	53.688	15.439	6	2	2	2	0.983	1.013	1.028	1.098	1.039	0.95	0.941	0.942	1.07	1.00	0.94
O43617	Trafficking protein particle complex subu	TRAPPC3	20.274	37.666	23.3	4	5	4	1.04	0.961	1.072	1.059	1.012	0.957	0.932	0.854	1.06	0.98	0.89
Q9Y265	RuvB-like 1 OS=Homo sapiens OX=960	RUVBL1	50.227	187.93	48.9	15	28	15	1.007	0.982	1.087	1.031	0.965	0.941	1.043	0.933	1.06	0.96	0.99
Q9Y266	Nuclear migration protein nudC OS=Hor	NUDC	38.242	117.7	34.1	11	25	11	0.962	0.985	1.032	1.041	0.957	0.969	1.034	0.99	1.06	0.99	1.04
Q06323	Proteasome activator complex subunit 1 (	PSME1	28.723	91.987	51.8	11	20	11	0.993	0.986	1.059	1.048	0.937	0.872	1.169	0.943	1.06	0.91	1.07
Q9Y5B9	FACT complex subunit SPT16 OS=Hom	SUPT16H	119.91	186.89	19.7	20	30	18	0.97	0.919	0.963	1.048	1.085	1.004	1.069	1.043	1.06	1.11	1.12
P43490	Nicotinamide phosphoribosyltransferase	NAMPT	55.52	71.701	18.5	8	13	8	0.934	0.975	1.041	0.991	1.014	0.965	1.16	1.054	1.06	1.04	1.16
Q9ULM6	CCR4-NOT transcription complex subun	CNOT6	63.306	44.869	3.4	1	1	1	0.971	1.016	1.083	1.032	1.031	0.961	1.109	0.842	1.06	1.00	0.98
Q9NQP4	Prefoldin subunit 4 OS=Homo sapiens O	PFDN4	15.314	27.983	29.1	3	6	3	0.961	1.058	1.064	1.085	0.973	1.007	1.013	1.002	1.06	0.98	1.00
Q92878	DNA repair protein RAD50 OS=Homo s	RAD50	153.89	206.23	18.8	23	28	23	0.972	0.955	1.027	1.024	1.025	0.965	1.087	1.018	1.06	1.03	1.09
Q4VCS5	Angiomotin OS=Homo sapiens OX=960	AMOT	118.08	10.76	1.8	2	2	2	1.029	0.869	0.968	1.052	0.959	0.831	1.156	1.13	1.06	0.94	1.20
Q9HCS7	Pre-mRNA-splicing factor SYF1 OS=Ho	XAB2	100.01	51.054	11.8	6	7	6	0.949	0.999	1.069	1.004	0.944	1.108	1.027	1.055	1.06	1.05	1.07
Q9H3S7	Tyrosine-protein phosphatase non-recept	PTPN23	178.97	54.491	6.2	7	7	7	1.02	0.928	1.034	1.039	1.045	0.81	1.175	0.956	1.06	0.95	1.09
Q07955	Serine/arginine-rich splicing factor 1 OS	SRSF1	27.744	105.92	45.2	11	26	11	0.967	0.953	1.024	1.019	1.095	0.96	1.107	0.998	1.06	1.07	1.10
Q9NZ52	ADP-ribosylation factor-binding protein	GGA3	78.314	45.027	9.3	5	5	5	1.045	0.897	1.106	0.96	1.069	1.033	0.987	0.909	1.06	1.08	0.98
P28066	Proteasome subunit alpha type-5 OS=Ho	PSMA5	26.411	199.01	43.6	8	17	8	0.957	1.032	1.087	1.029	0.933	1.177	0.844	0.979	1.06	1.06	0.92
Q96DC8	Enoyl-CoA hydratase domain-containing	ECHDC3	32.633	9.0321	4	1	1	1	1.01	0.936	1.133	0.937	1.124	1.005	0.942	1.011	1.06	1.09	1.00
Q8WWM7	Ataxin-2-like protein OS=Homo sapiens	ATXN2L	113.37	201.44	26.1	23	28	21	0.98	0.968	1.056	1.016	1.03	0.965	0.963	0.971	1.06	1.02	0.99
P12956	X-ray repair cross-complementing proteir	XRCC6	69.842	207.87	40.9	24	60	24	0.97	0.964	1.037	1.02	0.985	0.986	1.009	1.037	1.06	1.02	1.06
Q5M775	Cytospin-B OS=Homo sapiens OX=960	SPECC1	118.58	25.002	4	4	4	4	1.015	0.938	1.08	0.997	0.992	0.935	1.027	1.01	1.06	0.99	1.04
Q13905	Rap guanine nucleotide exchange factor	RAPGEF1	120.55	38.333	3.3	3	5	3	0.942	1.059	1.09	1.038	0.972	1.075	0.986	0.91	1.06	1.02	0.95
Q9P1F3	Costars family protein ABRACL OS=Ho	ABRACL	9.0564	12.399	16	1	1	1	1.142	0.72	0.921	1.059	1.182	0.593	1.16	1.115	1.06	0.95	1.22
O43399	Tumor protein D54 OS=Homo sapiens O	TPD52L2	22.237	68.819	45.1	8	15	8	0.994	0.933	1.044	1.005	1.028	0.924	1.159	0.954	1.06	1.01	1.10
P49721	Proteasome subunit beta type-2 OS=Hom	PSMB2	22.836	55.739	27.4	6	13	6	0.993	0.998	1.06	1.057	1.036	0.981	0.909	0.979	1.06	1.01	0.95
Q9H8Y8	Golgi reassembly-stacking protein 2 OS=	GORASP2	47.145	56.635	21.9	7	9	7	0.989	0.987	1.061	1.04	0.985	1.072	0.953	0.924	1.06	1.04	0.95
Q9NZM3	Intersectin-2 OS=Homo sapiens OX=960	ITSN2	193.46	101.62	8.6	12	11	9	0.949	0.902	0.951	1.017	0.988	0.995	1.162	1.068	1.06	1.07	1.20
O95487	Protein transport protein Sec24B OS=Ho	SEC24B	137.42	58.067	7.9	9	11	9	0.972	0.994	1.085	1.005	1.009	0.962	1.094	0.979	1.06	1.00	1.05
P15927	Replication protein A 32 kDa subunit O	RPA2	29.247	43.854	28.9	5	7	5	0.982	0.939	1.024	1.018	1.048	0.978	1.014	1.045	1.06	1.05	1.07
P84095	Rho-related GTP-binding protein RhoG (	RHOG	21.308	62.055	36.6	5	7	5	0.97	0.984	1.072	1.005	1.013	1.121	0.947	0.964	1.06	1.09	0.98
Q8NE01	Metal transporter CNNM3 OS=Homo sa	CNNM3	76.118	43.52	12.6	6	6	5	1.015	0.893	1.021	1.007	1.104	0.939	1.078	0.976	1.06	1.07	1.08
Q6P3W7	SCY1-like protein 2 OS=Homo sapiens (	SCYL2	103.71	64.351	11.3	7	7	7	1.014	0.974	1.112	1.001	0.989	1.113	0.861	0.942	1.06	1.06	0.91
A0FGR8	Extended synaptotagmin-2 OS=Homo sa	ESYT2	102.36	78.867	13.4	9	12	9	0.957	0.968	1.021	1.025	1.049	1.083	1.064	0.963	1.06	1.11	1.05
Q13190	Syntaxin-5 OS=Homo sapiens OX=9606	STX5	39.672	86.392	17.7	6	8	6	0.978	0.979	1.07	1.01	1.077	0.964	1.1	0.915	1.06	1.04	1.03
Q9HBH5	Retinol dehydrogenase 14 OS=Homo sap	RDH14	36.864	57.814	28.6	7	7	7	0.952	0.944	0.995	1.02	1.012	1.046	1.076	1.032	1.06	1.09	1.11
Q9Y3C4	EKC/KEOPS complex subunit TPRKB (	TPRKB	19.661	25.042	25.7	3	4	3	1.018	0.881	0.946	1.072	1.05	0.83	1.156	1.028	1.06	0.99	1.15
Q9BTL3	RNMT-activating mRNA cap methyltrans	RAMMET	14.381	14.817	23.7	2	3	2	0.973	0.818	0.905	0.998	1.11	0.88	1.269	1.121	1.06	1.11	1.33
Q9BV81	ER membrane protein complex subunit 6	EMC6	12.017	6.9473	10.9	1	1	1	0.99	0.85	0.982	0.973	1.234	0.95	1.105	0.99	1.06	1.19	1.14
P29692	Elongation factor 1-delta OS=Homo sapi	EEF1D	31.121	191.4	40.9	8	20	7	0.97	1.031	1.079	1.047	0.943	1.105	0.953	0.907	1.06	1.02	0.93
Q16854	Deoxyguanosine kinase, mitochondrial O	DGUOK	32.055	18.939	12.3	2	2	2	0.984	1.001	1.016	1.093	0.959	0.954	1.006	0.985	1.06	0.96	1.00
Q9NYZ1	Golgi apparatus membrane protein TVP2	TVP23B	23.576	7.2762	4.9	1	1	1	0.93	0.927	0.912	1.061	0.993	1.208	0.942	1.1	1.06	1.19	1.10
Q5JTW2	Centrosomal protein of 78 kDa OS=Hom	CEP78	76.396	18.074	4.6	3	3	3	0.79	0.78	0.681	0.987	1.295	1.425	0.882	1.407	1.06	1.73	1.46
P49591	Serine--tRNA ligase, cytoplasmic OS=H	SARS	58.777	61.744	20.8	7	11	7	0.967	0.991	1.049	1.031	0.985	1.021	0.988	0.998	1.06	1.02	1.01
Q92616	eIF-2-alpha kinase activator GCN1 OS=I	GCN1	292.75	323.31	24.7	56	79	56	0.989	0.971	1.062	1.02	0.983	0.949	1.018	0.984	1.06	0.99	1.02
Q07954	Prolow-density lipoprotein receptor-relat	LRP1	504.6	323.31	15.6	58	75	54	1.002	1.007	1.082	1.052	0.976	1.046	0.97	0.934	1.06	1.01	0.95
Q96DZ1	Endoplasmic reticulum lectin 1 OS=Hom	ERLEC1	54.858	24.795	6.6	3	3	3	1.006	0.973	1.075	1.027	1.073	1.024	0.935	0.912	1.06	1.06	0.93
P49368	T-complex protein 1 subunit gamma OS=	CCT3	60.533	260.09	51.4	27	56	27	0.982	0.981	1.068	1.017	1.014	1.004	0.95	1.005	1.06	1.03	1.00
Q92900	Regulator of nonsense transcripts 1 OS=l	UPF1	124.34	323.31	38.2	34	48	34	0.987	0.993	1.073	1.03	0.978	1.057	0.897	0.949	1.06	1.03	0.93
P07237	Protein disulfide-isomerase OS=Homo sa	P4HB	57.116	225.41	41.3	21	54	21	0.982	0.919	0.99	1.029	1.021	0.99	1.039	1.01	1.06	1.06	1.08
P52306	Rap1 GTPase-GDP dissociation stimulat	RAP1GDS	66.316	50.814	12.9	7	10	7	1.005	0.977	1.054	1.051	0.981	1.313	0.927	0.888	1.06	1.16	0.92
P30837	Aldehyde dehydrogenase X, mitochondri	ALDH1B1	57.206	73.562	18.6	8	9	8	0.997	0.969	1.017	1.071	1.004	1.013	1.016	1.016	1.06	1.03	1.03
Q9UBT2	SUMO-activating enzyme subunit 2 OS=	UBA2	71.223	147.43	31.4	15	17	15	0.953	0.982	1.043	1.012	1.023	1.149	0.899	0.987	1.06	1.12	0.97
Q8IX01	SURP and G-patch domain-containing pr	SUGP2	120.21	50.847	8	7	8	7	0.995	0.924	0.97	1.068	1.064	0.91	1.103	1.03	1.06	1.03	1.11
P31751	RAC-beta serine/threonine-protein kinas	AKT2	55.768	53.072	20.4	6	6	5	0.937	1.031	1.078	1.012	1.056	0.994	1.011	1.079	1.06	1.04	1.06
Q9BQ70	Transcription factor 25 OS=Homo sapien	TCF25	76.666	6.0363	1.3	1	1	1	0.941	1.049	1.113	1	1.211	1.272	0.783	0.723	1.06	1.25	0.76
O60524	Nuclear export mediator factor NEMF O	NEMF	122.95	56.244	8.2	8	9	8	0.936	0.976	1.035	0.995	1.116	1.063	1.058	1.092	1.06	1.14	1.12

O43768	Alpha-endosulfine OS=Homo sapiens O)	ENSA	13.389	38.778	43.8	5	6	4	0.984	0.961	0.978	1.087	1.019	1.117	0.884	1.034	1.06	1.10	0.99
Q7Z4H7	HAUS augmin-like complex subunit 6 O!	HAUS6	108.62	19.07	3.8	3	3	3	0.961	0.889	1.011	0.953	1.069	0.912	1.267	1.052	1.06	1.07	1.25
Q9NVU0	DNA-directed RNA polymerase III subun	POLR3E	79.897	21.72	5.6	3	4	3	0.878	0.973	0.926	1.039	1.169	0.94	1.112	1.084	1.06	1.14	1.19
Q15545	Transcription initiation factor TFIID subu	TAF7	40.259	22.56	8.6	3	4	3	1.043	0.971	1.11	1.028	0.932	0.991	1.1	0.954	1.06	0.95	1.02
O43390	Heterogeneous nuclear ribonucleoprotein	HNRNPR	70.942	180.03	32.4	19	39	13	0.976	0.976	1.062	1.01	1.041	1.062	0.902	0.959	1.06	1.08	0.95
P48444	Coatomer subunit delta OS=Homo sapier	ARCN1	57.21	162.22	35	20	31	20	0.988	0.981	1.065	1.025	1.018	1.068	0.957	0.977	1.06	1.06	0.98
Q9UJX2	Cell division cycle protein 23 homolog O	CDC23	68.833	43.618	15.7	7	8	7	0.978	0.861	0.976	0.976	1.121	0.973	1.107	1.037	1.06	1.14	1.17
O43752	Syntaxin-6 OS=Homo sapiens OX=9606	STX6	29.176	33.394	15.3	3	3	3	0.964	0.974	1.055	1.002	0.955	1.066	0.964	0.962	1.06	1.04	0.99
Q5T6F2	Ubiquitin-associated protein 2 OS=Homo	UBAP2	117.11	157.47	15.3	13	15	11	0.974	0.885	0.988	0.985	1.082	1.042	1.072	1.025	1.06	1.14	1.13
P42765	3-ketoacyl-CoA thiolase, mitochondrial (	ACAA2	41.924	205.61	34.8	9	11	9	0.986	0.972	1.012	1.066	0.987	0.974	0.924	1.005	1.06	1.00	0.99
Q9H307	Pinin OS=Homo sapiens OX=9606 GN=	PNN	81.627	144.7	20.2	14	20	14	0.932	1.027	1.058	1.021	1.023	1.168	0.92	0.99	1.06	1.12	0.97
Q9NRR4	Ribonuclease 3 OS=Homo sapiens OX=9	DROSHA	159.31	11.002	2.3	2	2	2	1.035	0.81	0.93	1.028	1.192	0.975	0.981	1.053	1.06	1.17	1.10
Q12769	Nuclear pore complex protein Nup160 O	NUP160	162.12	133.27	12.8	14	18	13	0.974	0.969	1.074	0.988	1.085	1.08	0.898	0.935	1.06	1.11	0.94
Q15386	Ubiquitin-protein ligase E3C OS=Homo	UBE3C	123.92	26.476	4	4	5	4	0.97	1.006	1.011	1.086	0.98	1.074	0.971	1.022	1.06	1.04	1.01
Q69YQ0	Cytospin-A OS=Homo sapiens OX=9606	SPECC1L	124.6	44.533	6.8	7	8	7	0.846	0.853	0.736	1.067	0.963	0.866	1.135	1.325	1.06	1.08	1.45
O95571	Persulfide dioxygenase ETHE1, mitochor	ETHE1	27.873	25.184	9.1	2	4	2	0.953	0.894	0.949	1.011	1.042	0.93	1.115	1.145	1.06	1.07	1.22
Q3YEC7	Rab-like protein 6 OS=Homo sapiens O)	RABL6	79.548	37.432	9.2	5	6	5	0.954	0.863	0.849	1.079	1.07	1.21	0.887	1.126	1.06	1.25	1.11
Q9P0J0	NADH dehydrogenase [ubiquinone] 1 alp	NDUFA13	16.698	31.364	26.4	3	4	3	0.969	0.98	1.126	0.942	1.157	0.995	0.976	0.941	1.06	1.10	0.98
P63218	Guanine nucleotide-binding protein G(I)/	GNG5	7.3184	11.391	52.9	2	2	2	0.637	1.781	1.599	0.966	0.491	1.906	0.566	0.433	1.06	0.99	0.41
P51648	Fatty aldehyde dehydrogenase OS=Homo	ALDH3A2	54.847	92.121	18.4	9	13	9	0.999	0.959	1.047	1.03	0.98	0.97	1.065	1.037	1.06	1.00	1.07
Q9UFC0	Leucine-rich repeat and WD repeat-conta	LRWD1	70.86	34.027	5.9	3	4	3	0.98	1.012	1.027	1.086	0.939	1.018	0.954	0.93	1.06	0.98	0.95
Q96B36	Proline-rich AKT1 substrate 1 OS=Homo	AKT1S1	27.383	21.089	14.5	3	3	3	0.961	0.982	1.09	0.971	1.09	1.44	0.549	1.039	1.06	1.30	0.82
Q9Y2G8	DnaJ homolog subfamily C member 16 O	DNAJC16	90.59	19.12	4.5	2	2	2	1.019	0.974	1.075	1.039	1.002	0.995	0.963	0.943	1.06	1.00	0.96
Q6YN16	Hydroxysteroid dehydrogenase-like prote	HSDL2	45.394	118.87	21.5	8	9	8	1.008	0.969	1.088	1.009	1.083	1.255	0.789	0.896	1.06	1.18	0.85
Q6YHU6	Thyroid adenoma-associated protein OS=	THADA	219.6	60.264	4	8	8	8	1.042	0.839	1.043	0.952	1.237	0.969	1.048	0.955	1.06	1.17	1.06
P07814	Bifunctional glutamate/proline--tRNA lig	EPRS	170.59	323.31	35.4	44	69	42	0.979	0.94	1.008	1.027	1.026	0.928	1.094	1.015	1.06	1.02	1.10
Q13951	Core-binding factor subunit beta OS=Ho	CBFB	21.508	25.726	18.1	3	5	3	0.966	1.036	1.087	1.036	1	1.153	0.798	1.002	1.06	1.08	0.90
Q9Y5X1	Sorting nexin-9 OS=Homo sapiens OX=9	SNX9	66.591	33.085	8.6	4	6	4	0.992	0.944	1.026	1.027	1.092	0.945	1.077	1.042	1.06	1.05	1.09
P53621	Coatomer subunit alpha OS=Homo sapie	COPA	138.34	234.21	27.6	30	40	30	0.985	0.952	1.052	1.002	1.038	1.096	0.984	1.022	1.06	1.10	1.04
Q8WUA8	Tsukushin OS=Homo sapiens OX=9606	TSKU	37.807	12.231	6.5	2	2	2	0.985	0.87	1.025	0.942	1.048	1.033	1.237	0.965	1.06	1.12	1.19
Q10570	Cleavage and polyadenylation specificity	CPSF1	160.88	100.27	11.2	14	15	14	0.986	0.938	1.009	1.031	1.033	0.955	1.028	0.993	1.06	1.03	1.05
P26368	Splicing factor U2AF 65 kDa subunit OS	U2AF2	53.5	101.36	22.5	9	23	9	0.935	1.007	1.047	1.012	1.001	1.046	1.001	0.957	1.06	1.05	1.01
Q86UV5	Ubiquitin carboxyl-terminal hydrolase 48	USP48	119.03	100.7	14.1	12	12	12	0.996	0.966	1.036	1.044	0.96	1.058	0.826	0.986	1.06	1.03	0.92
P23528	Cofilin-1 OS=Homo sapiens OX=9606 C	CFL1	18.502	116.21	54.8	10	23	9	1.01	0.952	1.032	1.048	0.961	0.856	1.081	0.991	1.06	0.93	1.06
P40227	T-complex protein 1 subunit zeta OS=Ho	CCT6A	58.024	175.83	39.4	18	32	18	0.962	1.017	1.065	1.033	0.967	1.095	0.908	0.946	1.06	1.04	0.94
Q8TEQ6	Gem-associated protein 5 OS=Homo sapi	GEMIN5	168.59	142.06	11.8	15	21	15	0.994	0.954	1.041	1.024	0.936	0.958	1.058	1.024	1.06	0.97	1.07
P21281	V-type proton ATPase subunit B, brain is	ATP6V1B2	56.5	91.381	37.6	15	23	10	1.001	0.981	1.104	0.997	0.955	0.92	1.104	0.993	1.06	0.95	1.06
O00330	Pyruvate dehydrogenase protein X compo	PDHX	54.122	69.356	24.4	9	13	9	0.932	0.955	1.03	0.97	0.992	1.059	1.006	1.042	1.06	1.09	1.09
Q9NPL8	Complex I assembly factor TIMMDC1, n	TIMMDC1	32.177	14.284	8.4	2	2	2	1.007	0.951	1.093	0.982	1.015	1.072	0.946	1	1.06	1.07	0.99
Q13155	Aminoacyl tRNA synthase complex-inter	AIMP2	35.348	112.33	22.2	5	8	5	0.975	1.001	1.075	1.019	1.008	0.974	1.024	0.891	1.06	1.00	0.97
Q9UHB9	Signal recognition particle subunit SRP6	SRP68	70.729	255.97	40	21	34	21	0.989	0.954	1.015	1.044	0.989	0.987	0.894	0.984	1.06	1.02	0.97
P17050	Alpha-N-acetylgalactosaminidase OS=Ho	NAGA	46.564	33.121	9.5	3	3	3	0.988	0.973	1.01	1.068	0.907	1.038	1.119	1.145	1.06	0.99	1.15
O60763	General vesicular transport factor p115 O	USO1	107.89	199.82	26.9	20	32	20	1.009	0.986	1.062	1.052	1.011	0.93	1.04	0.936	1.06	0.97	0.99
P48739	Phosphatidylinositol transfer protein beta	PITPNB	31.54	130.41	58.7	11	16	10	1.023	0.989	1.095	1.037	0.972	0.908	1.069	0.938	1.06	0.93	1.00
P25788	Proteasome subunit alpha type-3 OS=Ho	PSMA3	28.433	63.906	28.2	7	11	6	0.98	0.953	1.053	0.995	1.059	1.09	1.03	0.957	1.06	1.11	1.03
Q9UHI6	Probable ATP-dependent RNA helicase I	DDX20	92.239	77.246	15.7	10	13	10	0.956	1.013	1.065	1.021	0.975	1.081	1.016	0.996	1.06	1.04	1.02
O43719	HIV Tat-specific factor 1 OS=Homo sapi	HTATSF1	85.852	71.254	16.7	9	12	9	0.952	0.967	1.014	1.019	1.017	0.961	1.022	0.977	1.06	1.03	1.04
Q2KHT3	Protein CLEC16A OS=Homo sapiens O)	CLEC16A	117.71	10.937	2.2	2	2	2	1.139	0.833	1.134	0.955	1.141	0.925	0.759	1.1	1.06	1.05	0.94
P58107	Epiplakin OS=Homo sapiens OX=9606 (	EPPK1	555.65	323.31	28.6	44	55	38	0.949	0.925	0.975	1.01	1.032	0.921	1.209	1.089	1.06	1.04	1.23
Q8TBA6	Golgin subfamily A member 5 OS=Homo	GOLGA5	83.023	231.42	33.7	19	23	19	0.974	0.951	1.01	1.029	1.026	0.949	1.068	0.974	1.06	1.03	1.06
P08240	Signal recognition particle receptor subu	SRPRA	69.81	108.67	22.6	12	16	12	0.989	0.938	1.059	0.982	1.018	1.006	1.004	0.945	1.06	1.05	1.01
Q14738	Serine/threonine-protein phosphatase 2A	PPP2R5D	69.991	91.173	24.1	13	16	11	1.018	0.948	1.072	1.01	1.008	0.899	1.055	0.929	1.06	0.97	1.01
Q9BRP1	Programmed cell death protein 2-like OS	PDCD2L	39.416	19.357	8.9	3	4	3	1.015	1.003	1.11	1.027	1.002	1.035	0.95	0.873	1.06	1.01	0.90
Q9HB07	UPF0160 protein MYG1, mitochondrial (	C12orf10	42.449	94.769	18.6	6	10	6	0.966	0.952	1.012	1.019	1.024	1.078	0.953	1.035	1.06	1.10	1.04
Q86V40	Metalloprotease TIKI1 OS=Homo sapien	TRABD2A	57.676	12.743	5.1	2	2	2	0.922	1.049	1.18	0.907	1.171	0.626	1.29	1.037	1.06	0.91	1.18
P42166	Lamina-associated polypeptide 2, isoforn	TMPO	75.491	233.6	38.5	17	28	10	1.003	0.938	0.995	1.06	1.036	1.035	0.989	1.013	1.06	1.07	1.03
O14980	Exportin-1 OS=Homo sapiens OX=9606	XPO1	123.38	233.28	23.2	21	37	20	0.999	1.011	1.084	1.044	0.94	0.992	0.936	0.964	1.06	0.96	0.95
Q96DT7	Zinc finger and BTB domain-containing	ZBTB10	94.893	80.562	16.9	11	14	11	1.011	0.965	1.058	1.034	0.961	1.185	0.859	1.008	1.06	1.09	0.94
Q8IWA5	Choline transporter-like protein 2 OS=H	SLC44A2	80.123	18.224	3.7	3	4	3	0.99	0.935	1.057	0.981	0.98	0.976	1.11	1.05	1.06	1.02	1.12
Q99615	DnaJ homolog subfamily C member 7 OS	DNAJC7	56.44	139.91	27.7	15	23	15	0.976	0.954	1.037	1.006	1.036	0.993	0.975	0.962	1.06	1.05	1.00
Q9UHG3	Prenylcysteine oxidase 1 OS=Homo sapi	PCYOX1	56.639	85.01	19.8	8	11	8	1.051	0.966	1.118	1.017	0.958	0.91	1.071	0.969	1.06	0.93	1.01
Q8IVH8	Mitogen-activated protein kinase kinase	MAP4K3	101.31	12.076	2.2	2	2	2	0.896	1.123	1.075	1.062	0.932	1.041	1.003	0.96	1.06	0.98	0.97
Q13901	Nuclear nucleic acid-binding protein C1D	C1D	16.019	7.1375	7.1	1	2	1	1.004	0.879	0.972	1.021	1	0.982	1.048	1.134	1.06	1.05	1.16
P55060	Exportin-2 OS=Homo sapiens OX=9606	CSE1L	110.42	247.3	26.2	25	44	25	0.942	0.981	1.023	1.012	1.031	1.03	0.988	0.997	1.06	1.07	1.03
P05423	DNA-directed RNA polymerase III subun	POLR3D	44.395	8.4553	4.5	1	1	1	0.982	0.891	0.923	1.059	1.111	1.223	0.866	0.968	1.06	1.25	0.98
Q5T3I0	G patch domain-containing protein 4 OS	GPATCH4	50.381	43.103	10.3	4	5	4	0.942	0.933	1.011	0.973	1.252	1.082	0.908	0.978	1.06	1.24	1.01
Q9Y285	Phenylalanine--tRNA ligase alpha subuni	FARSA	57.563	80.595	14.8	6	12	6	0.923	0.987	1.033	0.988	1.082	0.992	1.006	1.093	1.06	1.09	1.10
O43237	Cytoplasmic dynein 1 light intermediate (	DYNC1LI2	54.099	134.66	27.4	10	16	9	1.039	0.891	0.989	1.053	1.002	0.958	1.081	1.034	1.06	1.02	1.10
Q9H0R6	Glutamyl-tRNA(Gln) amidotransferase su	QRSL1	57.46	60.696	19.3	8	9	8	0.967	0.998	1.095	0.984	1.077	1.061	0.957	0.976	1.06	1.09	0.98
Q53H96	Pyrroline-5-carboxylate reductase 3 OS=	PYCR3	28.663	26.762	21.5	4	4	4	0.941	1.011	1.071	0.994	0.998	0.968	1.104	1.021	1.06	1.01	1.09
P30740	Leukocyte elastase inhibitor OS=Homo s	SERPINB1	42.741	47.547	19.5	7	11	6	0.96	1.01	1.062	1.022	0.958	0.908	1.085	0.993	1.06	0.95	1.05
Q9BQC6	Ribosomal protein 63, mitochondrial OS	MRPL57	12.266	6.4158	12.7	1	1	1	0.974	0.969	1.099	0.956	1.083	1.002	1.146	0.869	1.06	1.07	1.04
Q9NYY8	FAST kinase domain-containing protein	FASTKD2	81.462	51.014	12	6	6	6	0.961	1.088	1.165	1.002	1.018	1.01	0.95	0.891	1.06	0.99	0.90
P17858	ATP-dependent 6-phosphofructokinase, l	PFKL	85.018	157.26	30.5	21	35	17	0.99	1.008	1.059	1.054	0.933	0.918	1.026	0.965	1.06	0.93	1.00
O43583	Density-regulated protein OS=Homo sapi	DENR	22.092	35.875	26.8	5	5	5	1.011	0.883	0.991	1.012	1.051	0.956	1.066	1.012	1.06	1.06	1.10
O60568	Procollagen-lysine,2-oxoglutarate 5-diox	PLOD3	84.784	152.26	22.1	13	16	13	0.962	0.951	1.002	1.021	0.984	0.998	0.971	1.001	1.06	1.04	1.03
Q13011	Delta(3,5)-Delta(2,4)-dienoyl-CoA isome	ECH1	35.816	55.9	21.3	7	15	7	0.969	0.91	0.968	1.019	1.018	0.906	1.104	1.108	1.06	1.02	1.18
P12270	Nucleoprotein TPR OS=Homo sapiens O	TPR	267.29	323.31	28.4	59	89	59	0.98	0.969	1.024	1.037	1.029	1.067	0.932	0.959	1.06	1.08	0.97
P08758	Annexin A5 OS=Homo sapiens OX=960	ANXA5	35.936	123.51	54.7	18	31	17	1.001	1.003	1.086	1.033	0.996	0.895	1.087	0.99	1.06	0.94	1.04
O15372	Eukaryotic translation initiation factor 3	EIF3H	39.93	142.46	29.8	10	15	10	0.988	0.999	1.074	1.027	0.964	0.991	1.077	0.992	1.06	0.98	1.04
Q9NQR4	Omega-amidase NIT2 OS=Homo sapiens	NIT2	30.608	97.952	39.9	9	13	9	0.984	0.934	1.034	0.994	1.004	0.974	1.108	0.965	1.06	1.03	1.08
O95232	Luc7-like protein 3 OS=Homo sapiens O	LUC7L3	51.466	75.511	23.6	9	14	9	1.01	0.979	1.082	1.021	1.02	1.008	0.994	0.837	1.06	1.02	0.92
Q9ULV0	Unconventional myosin-Vb OS=Homo sa	MYO5B	213.67	12.732	1.2	2	2	2	0.942	0.927	0.966	1.01	1.078	0.871	1.144	1.158	1.06	1.04	1.23
Q1KMD3	Heterogeneous nuclear ribonucleoprotein	HNRNPUL	85.104	155.66	24.5	14	24	14	0.992	0.93	1.019	1.013	1.068	0.969	1.005	1.024	1.06	1.06	1.06
Q9Y5T5	Ubiquitin carboxyl-terminal hydrolase 16	USP16	93.569	12.404	2.7	2	2	2	0.943	0.962	0.938	1.076	1.054	1.042	0.962	1.069	1.06	1.10	1.07
Q7Z4S6	Kinesin-like protein KIF21A OS=Homo	KIF21A	187.18	108.73	8.5	10	10	8	0.976	0.97	1.025	1.032	0.973	1.074	0.959	0.967	1.06	1.05	0.99
P53602	Diphosphomevalonate decarboxylase OS	MVD	43.404	43.845	15.5	6	6	6	0.999	0.948	1.015	1.043	1.128	1.005	0.901	0.976	1.06	1.10	0.96
Q14197	Peptidyl-tRNA hydrolase ICT1, mitochor	MRPL58	23.63	31.338	15	3	3	3	1.033	0.935	1.063	1.017	1.06	0.935	0.978	0.996	1.06	1.01	1.00
Q9NRX5	Serine incorporator 1 OS=Homo sapiens	SERINC1	50.494	11.51	5.7	2	2	2	1.048	0.815	0.984	0.985	1.378	0.962	0.99	0.85	1.06	1.26	0.99
Q9NRL2	Bromodomain adjacent to zinc finger dor	BAZ1A	178.7	122.06	10.9	16	19	16	0.961	0.92	1	0.988	1.143	1.213	0.901	0.978	1.06	1.25	1.00
O60293	Zinc finger C3H1 domain-containing pro	ZFC3H1	226.35	53.746	5.6	8	9	8	1.008	0.961	1.084	0.997	0.985	1.134	0.869	1.013	1.06	1.08	0.96
Q86UK7	E3 ubiquitin-protein ligase ZNF598 OS=	ZNF598	98.636	101.26	13.9	10	10	9	0.96	0.994	1.048	1.017	1.031	0.984	1.048	0.922	1.06	1.03	1.01
P09661	U2 small nuclear ribonucleoprotein A' OS	SNRPA1	28.415	53.394	28.2	8	9	8	1.024	0.985	1.114	1.009	1.05	1.228	0.792	0.902	1.06	1.13	0.84
Q9Y446	Plakophilin-3 OS=Homo sapiens OX=96	PKP3	87.081	134.03	19.3	12	15	12	1.003	0.905	1.03	0.986	1.133	1.043	1.021	0.955	1.06	1.14	1.04
Q6L8Q7	2',5'-phosphodiesterase 12 OS=Homo sap	PDE12	67.351	48.906	16.9	5	6	5	0.947	0.963	1.045	0.973	1.016	1.015	1.062	1.078	1.06	1.06	1.12
Q9UM13	Anaphase-promoting complex subunit 10	ANAPC10	21.252	6.303	4.9	1	2	1	1.004	0.924	1.013	1.024	1.058	0.958	1.064	0.986	1.06	1.05	1.06
Q96C36	Pyrroline-5-carboxylate reductase 2 OS=	PYCR2	33.637	25.214	14.4	3	4	2	0.952	0.961	0.987	1.034	1.04	0.941	1.088	1.065	1.06	1.04	1.13
Q9NPQ8	Synembryn-A OS=Homo sapiens OX=96	RIC8A	59.709	58.264	13.4	7	8	6	0.997	0.881	1.008	0.976	1.004	1.098	0.999	0.976	1.06	1.12	1.05
Q15020	Squamous cell carcinoma antigen recogni	SART3	109.93	154	23.2	16	22	15	0.996	1.008	1.096	1.021	0.955	1.168	0.914	0.957	1.06	1.06	0.93
O43292	Glycosylphosphatidylinositol anchor atta	GPAA1	67.622	22.603	5.2	3	3	3	1.025	0.963	1.053	1.047	0.987	1.034	0.927	0.966	1.06	1.02	0.95
P42167	Lamina-associated polypeptide 2, isoforn	TMPO	50.67	115.28	47.6	14	27	7	0.942	1.002	1.094	0.959	1.026	1.126	0.929	1.001	1.06	1.11	0.99
Q96LJ7	Dehydrogenase/reductase SDR family me	DHRS1	33.909	29.403	10.9	3	3	3	1.034	0.912	1.107	0.948	1.045	0.998	0.972	1.057	1.06	1.05	1.04
Q99549	M-phase phosphoprotein 8 OS=Homo sa	MPHOSPH	97.181	53.644	8.4	5	6	5	1.006	0.979	1.07	1.026	1.034	1.141	0.846	0.946	1.06	1.10	0.90
Q6NXT4	Zinc transporter 6 OS=Homo sapiens OX	SLC30A6	51.115	7.9309	2.6	1	2	1	0.943	0.975	0.931	1.094	1.019	1.217	0.801	1.059	1.06	1.17	0.97
O95359	Transforming acidic coiled-coil-containin	TACC2	309.42	95.787	6.1	11	12	10	0.943	1.011	1.006	1.057	0.967	0.866	1.153	1.068	1.06	0.94	1.14
P61020	Ras-related protein Rab-5B OS=Homo sa	RAB5B	23.707	39.189	41.9	7	10	4	0.98	0.924	0.989	1.021	1.065	1.08	0.908	1.164	1.06	1.13	1.09
Q92804	TATA-binding protein-associated factor 2	TAF15	61.829	72.119	18.1	8	12	6	1.016	0.944	0.994	1.075	1.018	0.947	0.98	0.958	1.06	1.00	0.99
Q8WW12	PEST proteolytic signal-containing nucle	PCNP	18.925	61.565	36	5	10	5	0.978	1.036	1.056	1.07	0.987	0.968	0.94	0.921	1.06	0.97	0.92
Q13043	Serine/threonine-protein kinase 4 OS=Ho	STK4	55.63	86.084	25.1	10	12	6	1.01	1.007	1.098	1.031	0.892	0.909	1.138	0.947	1.06	0.89	1.03
Q96J02	E3 ubiquitin-protein ligase Itchy homolo	ITCH	102.8	33.371	6.5	5	5	5	0.974	0.938	0.978	1.04	0.973	0.923	1.124	1.022	1.06	0.99	1.12
Q86X10	Ral GTPase-activating protein subunit be	RALGAPB	166.8	27.533	3.7	4	4	4	0.952	1.016	1.084	0.993	0.981	1.004	1.129	0.936	1.06	1.01	1.05
Q9C0C9	(E3-independent) E2 ubiquitin-conjugati	UBE2O	141.29	80.048	10.4	11	13	11	0.992	1.031	1.096	1.039	0.991	1.003	0.959	0.923	1.06	0.99	0.93
Q9Y6C9	Mitochondrial carrier homolog 2 OS=Ho	MTCH2	33.331	35.568	19.1	4	6	4	0.788	1.47	1.348	1.035	0.632	1.595	0.681	0.656	1.06	0.99	0.59
Q02241	Kinesin-like protein KIF23 OS=Homo sa	KIF23	110.06	181.68	22.9	17	20	17	0.986	0.931	0.989	1.034	1.054	1.05	0.943	1.059	1.06	1.10	1.04
Q96D53	Atypical kinase COQ8B, mitochondrial (	COQ8B	60.069	8.1185	2.8	1	1	1	1.048	0.96	1.046	1.073	1.019	0.904	1.037	0.87	1.06	0.96	0.95
O60841	Eukaryotic translation initiation factor 5I	EIF5B	138.83	222.64	26.1	26	40	25	0.99	0.93	0.979	1.047	1.008	0.971	1.018	1.066	1.06	1.03	1.09
L0R819	ASNSD1 upstream open reading frame pi	ASDURF	11.25	28.818	56.2	4	5	4	0.884	1.182	1.123	1.057	0.824	1.222	0.941	0.876	1.06	0.99	0.88
Q08170	Serine/arginine-rich splicing factor 4 OS	SRSF4	56.678	19.901	10.3	5	6	2	1.02	0.869	1.02	0.973	1.137	0.852	1.114	1.071	1.06	1.05	1.16
Q15437	Protein transport protein Sec23B OS=Ho	SEC23B	86.478	128.7	28.3	15	20	13	0.998	0.983	1.096	0.994	1.069	1.138	0.895	0.906	1.06	1.11	0.91
P46063	ATP-dependent DNA helicase Q1 OS=H	RECQL	73.457	142.9	22.2	10	13	10	0.945	0.964	1.023	0.991	0.983	0.996	1.104	1.041	1.06	1.04	1.12
P13807	Glycogen [starch] synthase, muscle OS=I	GYS1	83.785	53.474	11.3	6	7	6	0.962	0.989	1.029	1.029	0.946	0.961	1.058	0.991	1.05	0.98	1.05
O43913	Origin recognition complex subunit 5 OS	ORC5	50.282	51.295	16.6	7	9	7	0.976	0.942	0.995	1.028	1.026	1.176	0.824	0.943	1.05	1.15	0.92
Q99460	26S proteasome non-ATPase regulatory s	PSMD1	105.84	221.12	36.3	25	35	23	0.958	0.981	1.018	1.027	0.992	1.014	0.928	1.032	1.05	1.03	1.01
Q9HC98	Serine/threonine-protein kinase Nek6 OS	NEK6	35.714	13.204	5.8	2	2	2	0.995	0.872	0.99	0.979	1.072	0.943	1.242	0.973	1.05	1.08	1.19
P0DN76	Splicing factor U2AF 35 kDa subunit-lik	U2AF1L5	27.872	57.85	24.2	5	8	4	0.899	0.99	0.964	1.028	0.961	1.088	1.012	1.111	1.05	1.08	1.12
Q5VIR6	Vacuolar protein sorting-associated prote	VPS53	79.652	6.6967	1.7	1	1	1	0.912	0.999	1.059	0.956	1.12	0.938	1.151	1.018	1.05	1.08	1.14
P02792	Ferritin light chain OS=Homo sapiens O)	FTL	20.019	91.474	37.1	6	11	6	1.03	0.918	0.99	1.064	0.999	0.939	1.045	0.962	1.05	0.99	1.03
Q00613	Heat shock factor protein 1 OS=Homo sa	HSF1	57.26	33.32	4.5	2	2	2	0.992	0.96	0.999	1.059	0.993	1.019	0.951	1.047	1.05	1.03	1.02
Q9UH17	DNA dC->dU-editing enzyme APOBEC-	APOBEC3	45.924	12.355	5	2	2	2	0.902	0.857	0.894	0.96	1.041	1.112	1.13	1.275	1.05	1.22	1.37
P51610	Host cell factor 1 OS=Homo sapiens OX	HCFC1	208.73	276.77	17.4	27	38	27	0.975	0.951	0.998	1.032	1.022	0.956	1.029	0.983	1.05	1.03	1.04
P28074	Proteasome subunit beta type-5 OS=Hor	PSMB5	28.48	104.7	36.9	9	14	9	0.938	1.106	1.125	1.029	0.874	1.258	0.765	0.876	1.05	1.04	0.80
O96000	NADH dehydrogenase [ubiquinone] 1 be	NDUFB10	20.776	77.918	43	7	10	7	0.97	0.965	1.027	1.012	1.094	1.02	0.992	0.985	1.05	1.09	1.02
P43487	Ran-specific GTPase-activating protein C	RANBP1	23.31	91.023	31.8	4	13	4	0.99	0.985	1.047	1.034	0.975	0.997	0.96	0.948	1.05	1.00	0.97
Q96A49	Synapse-associated protein 1 OS=Homo	SYAP1	39.933	47.915	26.4	7	7	7	0.957	0.926	1.001	0.983	1.035	1.056	1.076	1.027	1.05	1.11	1.12
Q9UHY1	Nuclear receptor-binding protein OS=Ho	NRBP1	59.844	66.146	13.8	5	10	5	0.969	0.997	1.019	1.052	0.969	1.004	0.977	0.981	1.05	1.00	1.00
Q13435	Splicing factor 3B subunit 2 OS=Homo s	SF3B2	100.23	261.76	27.7	21	37	21	0.984	0.983	1.044	1.028	1.044	1.003	1.013	0.99	1.05	1.04	1.02
O60645	Exocyst complex component 3 OS=Hom	EXOC3	86.844	57.357	12.3	7	8	7	0.945	1.062	1.052	1.062	1.086	1.038	0.881	0.925	1.05	1.06	0.90
Q8WUQ7	Cactin OS=Homo sapiens OX=9606 GN	CACTIN	88.701	26.684	8.2	4	4	4	1.015	0.918	1.021	1.015	0.98	1.194	0.774	0.91	1.05	1.12	0.87
P14868	Aspartate--tRNA ligase, cytoplasmic OS	DARS	57.136	276.4	48.5	23	43	23	0.98	0.972	1.014	1.042	1.006	0.968	1.067	0.973	1.05	1.01	1.05
Q96KP1	Exocyst complex component 2 OS=Hom	EXOC2	104.07	122.37	12	10	14	10	1.002	0.989	1.057	1.04	1.009	0.972	1.087	0.941	1.05	0.99	1.02
P61758	Prefoldin subunit 3 OS=Homo sapiens O	VBP1	22.658	54.847	33.5	6	9	6	0.983	0.99	1.042	1.036	1.058	1.027	0.96	0.959	1.05	1.06	0.97
Q9BU61	NADH dehydrogenase [ubiquinone] 1 alp	NDUFAF3	20.35	32.066	31.5	4	6	4	0.988	0.934	1.007	1.017	1.044	0.962	1.182	1.003	1.05	1.04	1.14
Q9UKG1	DCC-interacting protein 13-alpha OS=H	APPL1	79.663	29.696	9.3	4	4	4	0.923	1.038	1.01	1.055	0.943	1.238	0.895	0.976	1.05	1.11	0.95
Q5J8M3	ER membrane protein complex subunit 4	EMC4	20.086	19.012	15.8	2	3	2	0.957	0.987	1.025	1.022	0.938	1.16	0.853	1.067	1.05	1.08	0.99
O96033	Molybdopterin synthase sulfur carrier sul	MOCS2	9.7552	18.998	37.5	3	3	3	1.04	1.003	1.198	0.953	0.915	1.053	1.068	0.823	1.05	0.96	0.93
Q8NFH3	Nucleoporin Nup43 OS=Homo sapiens C	NUP43	42.15	25.68	11.6	4	8	4	0.968	0.93	1.052	0.946	1.163	1.094	1.003	0.959	1.05	1.19	1.03
Q8ND25	E3 ubiquitin-protein ligase ZNRF1 OS=l	ZNRF1	23.782	7.8729	6.2	1	1	1	1.04	0.934	1.072	1.006	1.041	1.271	0.697	0.965	1.05	1.17	0.84
Q71UI9	Histone H2A.V OS=Homo sapiens OX=9	H2AFV	13.509	31.928	25.8	3	18	2	0.951	1.042	1.12	0.978	0.873	1.056	0.957	1.008	1.05	0.97	0.99
Q8WTW3	Conserved oligomeric Golgi complex sub	COG1	108.98	56.719	8.8	6	6	6	1.042	0.899	1.058	0.985	1.02	1.113	0.988	0.928	1.05	1.10	0.99
Q969Z0	FAST kinase domain-containing protein	TBRG4	70.737	33.204	11.9	5	5	5	0.985	0.996	1.06	1.025	0.961	1.136	0.882	1.008	1.05	1.06	0.95
O95782	AP-2 complex subunit alpha-1 OS=Hom	AP2A1	107.54	254.9	33.6	27	45	20	0.992	0.952	1.047	0.999	1.037	1.084	0.925	0.961	1.05	1.09	0.97
Q6P1N9	Putative deoxyribonuclease TATDN1 OS	TATDN1	33.601	33.224	19.2	5	6	5	1.068	0.897	0.99	1.078	1.01	1.135	0.859	0.905	1.05	1.09	0.90
P41223	Protein BUD31 homolog OS=Homo sapi	BUD31	17	14.463	13.9	2	4	2	0.941	0.95	0.983	1.007	0.99	1.091	1.073	1.076	1.05	1.10	1.14
Q8N5I9	Uncharacterized protein C12orf45 OS=H	C12orf45	20.123	20.398	19.5	3	4	3	1.025	0.906	1.053	0.979	0.981	0.915	1.108	1.013	1.05	0.98	1.10
Q9P2T1	GMP reductase 2 OS=Homo sapiens OX	GMPR2	37.874	54.226	27	7	10	7	0.935	1.036	1.077	0.997	0.976	0.966	1.057	1.005	1.05	0.99	1.05
O75792	Ribonuclease H2 subunit A OS=Homo sa	RNASEH2	33.395	30.761	22.4	5	6	5	0.953	0.908	0.952	1.006	1.166	1.076	0.99	1.037	1.05	1.20	1.09

Q9Y5Q0	Fatty acid desaturase 3 OS=Homo sapien	FADS3	51.144	18.303	7.6	2	2	2	0.898	1.06	1.015	1.045	1.101	0.879	1.216	0.876	1.05	1.01	1.07
P15531	Nucleoside diphosphate kinase A OS=Hc	NME1	17.149	108.64	70.4	11	23	4	0.96	1.018	1.049	1.032	0.954	1.001	1.005	0.963	1.05	0.99	0.99
O95433	Activator of 90 kDa heat shock protein A	AHSA1	38.274	75.875	29.6	8	15	8	0.993	1.006	1.061	1.042	1.012	0.988	1.044	0.962	1.05	1.00	1.00
Q9H0U4	Ras-related protein Rab-1B OS=Homo sa	RAB1B	22.171	107.57	66.7	10	23	5	1.086	0.895	1.056	1.028	0.977	0.825	1.136	1.02	1.05	0.91	1.09
Q6PKG0	La-related protein 1 OS=Homo sapiens C	LARP1	123.51	164.66	25.3	23	27	21	1.02	0.965	1.047	1.041	0.972	1.042	0.931	0.956	1.05	1.01	0.95
Q13200	26S proteasome non-ATPase regulatory s	PSMD2	100.2	201.59	32.8	23	33	22	0.966	0.982	1.013	1.036	1.007	1.159	0.939	1.007	1.05	1.11	1.00
P62333	26S proteasome regulatory subunit 10B (	PSMC6	44.172	181.48	48.3	15	23	15	0.99	0.998	1.075	1.016	0.994	1.123	0.94	0.961	1.05	1.06	0.96
P51151	Ras-related protein Rab-9A OS=Homo sa	RAB9A	22.837	15.623	12.4	2	2	2	1	0.911	1.009	1.001	1.066	0.91	1.18	0.97	1.05	1.03	1.13
O75208	Ubiquinone biosynthesis protein COQ9,	COQ9	35.509	26.938	16	4	4	4	1.024	0.875	0.98	1.017	1.09	1.102	1.082	0.999	1.05	1.15	1.10
Q09666	Neuroblast differentiation-associated pro	AHNAK	629.09	323.31	55.2	196	368	195	0.991	1.025	1.065	1.055	0.898	0.908	1.059	0.988	1.05	0.90	1.02
Q96C90	Protein phosphatase 1 regulatory subunit	PPP1R14B	15.911	18.448	17	2	3	2	0.992	1.01	1.102	1.003	0.991	1.215	0.85	0.898	1.05	1.10	0.87
O95169	NADH dehydrogenase [ubiquinone] 1 be	NDUFB8	21.766	28.102	28	4	5	4	0.969	0.956	1.026	0.998	1.044	0.95	1.102	1.038	1.05	1.04	1.11
Q9UHV9	Prefoldin subunit 2 OS=Homo sapiens O	PFDN2	16.648	42.038	24	3	8	3	0.996	0.97	1.037	1.03	1.017	1.03	0.931	0.969	1.05	1.04	0.97
Q16539	Mitogen-activated protein kinase 14 OS=	MAPK14	41.293	45.093	20.3	6	7	6	1.06	0.907	1.02	1.048	1.043	0.898	1.066	0.956	1.05	0.99	1.03
Q6P2Q9	Pre-mRNA-processing-splicing factor 8 (	PRPF8	273.6	323.31	27	52	84	52	0.958	0.972	1.016	1.013	1.051	1.226	0.895	1.008	1.05	1.18	0.99
Q9H773	dCTP pyrophosphatase 1 OS=Homo sapi	DCTPP1	18.681	29.973	26.5	4	6	4	1.008	0.926	1.02	1.013	1.066	0.892	1.078	0.976	1.05	1.01	1.06
P26374	Rab proteins geranylgeranyltransferase cc	CHML	74.071	44.453	10.2	6	7	6	0.993	1.039	1.113	1.023	0.964	0.784	1.174	0.915	1.05	0.86	1.03
O95801	Tetratricopeptide repeat protein 4 OS=Hc	TTC4	44.678	27.478	9.3	4	4	4	1.082	0.777	0.899	1.055	1.138	0.899	1.036	1.06	1.05	1.10	1.13
Q5SXM2	snRNA-activating protein complex subun	SNAPC4	159.43	17.945	2.2	3	3	3	0.996	0.964	1.082	0.978	1.184	1.007	0.914	0.945	1.05	1.12	0.95
P41567	Eukaryotic translation initiation factor 1 (	EIF1	12.732	165.54	65.5	7	10	7	1.002	0.983	1.038	1.048	1.007	0.945	1.109	0.977	1.05	0.98	1.05
Q13277	Syntaxin-3 OS=Homo sapiens OX=9606	STX3	33.155	26.163	13.5	4	4	4	0.983	0.984	1.019	1.048	0.941	1.01	0.986	1.058	1.05	0.99	1.04
Q96F86	Enhancer of mRNA-decapping protein 3	EDC3	56.077	50.437	17.7	7	8	7	0.981	0.978	1.032	1.026	1.051	1.023	0.913	1.018	1.05	1.06	0.99
Q9H269	Vacuolar protein sorting-associated prote	VPS16	94.693	38.501	6.8	4	5	4	0.97	0.97	1.08	0.958	1.07	1.13	1.018	0.946	1.05	1.13	1.01
P11233	Ras-related protein Ral-A OS=Homo sap	RALA	23.567	28.91	19.4	4	6	2	0.999	0.922	0.994	1.024	1.053	0.979	1.03	1.032	1.05	1.06	1.07
Q5VW38	Protein GPR107 OS=Homo sapiens OX=	GPR107	66.99	12.717	3.7	2	2	2	0.954	1.007	1.081	0.979	0.936	1.193	0.867	1.102	1.05	1.09	1.00
Q9UBB9	Tuftelin-interacting protein 11 OS=Homo	TFIP11	96.819	64.97	15.8	9	10	9	0.943	0.981	1.022	0.999	1.049	1.231	0.808	1.044	1.05	1.19	0.96
P23246	Splicing factor, proline- and glutamine-ri	SFPQ	76.149	219.9	31.5	21	43	19	0.985	1.004	1.058	1.031	1.014	1.096	0.868	0.939	1.05	1.06	0.91
Q13177	Serine/threonine-protein kinase PAK 2 O	PAK2	58.042	115.83	36.3	13	27	9	0.984	1.01	1.06	1.034	0.973	0.946	1.084	0.98	1.05	0.96	1.04
Q9Y314	Nitric oxide synthase-interacting protein	NOSIP	33.172	102.62	49.2	10	11	10	0.972	1.062	1.092	1.044	0.922	1.138	0.962	0.935	1.05	1.01	0.93
P60842	Eukaryotic initiation factor 4A-I OS=Hor	EIF4A1	46.153	195.63	43.3	16	48	8	0.997	1.003	1.072	1.028	0.93	1.202	0.839	0.907	1.05	1.07	0.87
Q96BD0	Solute carrier organic anion transporter fa	SLCO4A1	77.193	14.458	3.2	2	2	2	0.915	0.967	1.011	0.965	1.217	1.27	0.87	0.931	1.05	1.32	0.96
Q9NYK5	39S ribosomal protein L39, mitochondria	MRPL39	38.711	55.672	14.5	6	6	5	1.049	0.984	1.139	0.995	1.065	0.971	0.876	0.892	1.05	1.00	0.87
O43809	Cleavage and polyadenylation specificity	NUDT21	26.227	94.928	30.8	7	17	7	0.963	1.003	1.087	0.976	1	0.968	1.017	1.021	1.05	1.00	1.04
Q68CZ2	Tensin-3 OS=Homo sapiens OX=9606 G	TNS3	155.26	83.177	9.2	9	10	9	0.921	0.944	0.959	0.998	1.02	1.023	0.986	1.053	1.05	1.10	1.09
O60826	Coiled-coil domain-containing protein 22	CCDC22	70.755	86.317	25	11	13	11	0.973	0.939	0.978	1.028	1.067	1.04	1.016	0.975	1.05	1.10	1.04
P12236	ADP/ATP translocase 3 OS=Homo sapie	SLC25A6	32.866	14.952	31.5	10	17	1	1.031	0.965	1.121	0.973	1.032	1.155	0.852	0.925	1.05	1.10	0.89
P16333	Cytoplasmic protein NCK1 OS=Homo sa	NCK1	42.864	25.652	13	5	7	4	0.997	0.942	1.028	1.006	0.982	0.928	1.165	0.969	1.05	0.99	1.10
Q15477	Helicase SKI2W OS=Homo sapiens OX=	SKIV2L	137.75	124.71	15.7	15	19	15	0.977	0.969	1.024	1.017	0.98	1.124	0.926	1.03	1.05	1.08	1.01
Q9H6T3	RNA polymerase II-associated protein 3 (	RPAP3	75.718	107.25	18.6	10	15	10	0.994	0.995	1.02	1.066	0.97	1.049	0.941	0.98	1.05	1.02	0.97
P37198	Nuclear pore glycoprotein p62 OS=Homo	NUP62	53.254	88.717	18.8	9	11	9	0.956	0.954	1.022	0.981	1.052	0.966	1.112	1.013	1.05	1.06	1.11
Q14247	Src substrate cortactin OS=Homo sapiens	CTTN	61.585	214.08	39.1	18	32	18	0.963	0.989	1.041	1.006	0.921	0.94	1.019	1.045	1.05	0.95	1.06
P28070	Proteasome subunit beta type-4 OS=Hom	PSMB4	29.204	39.995	25	4	10	4	0.962	1.059	1.066	1.053	0.91	1.16	0.969	1.02	1.05	1.02	0.98
Q7KZ85	Transcription elongation factor SPT6 OS	SUPT6H	199.07	161.74	13.6	20	22	20	0.965	0.955	1.015	0.998	1.027	1.18	0.916	1.048	1.05	1.15	1.02
Q86UP2	Kinectin OS=Homo sapiens OX=9606 G	KTN1	156.27	323.31	32.2	43	55	43	1.017	0.993	1.081	1.026	0.974	1.01	0.963	0.888	1.05	0.99	0.92
Q75N03	E3 ubiquitin-protein ligase Hakai OS=Hc	CBLL1	54.519	12.462	4.1	1	1	1	0.985	0.865	0.971	0.968	1.083	0.965	1.162	1.091	1.05	1.11	1.22
Q99459	Cell division cycle 5-like protein OS=Ho	CDC5L	92.25	170.04	22.4	15	24	15	0.971	0.944	1.023	0.984	1.056	1.164	0.978	1.028	1.05	1.16	1.05
Q9BQP7	Mitochondrial genome maintenance exon	MGME1	39.42	51.822	26.2	7	9	7	0.983	0.932	1.035	0.972	1.073	1.076	1.014	1.089	1.05	1.12	1.10
Q92890	Ubiquitin recognition factor in ER-associ	UFD1	34.5	51.331	20.5	5	6	5	0.927	0.949	0.99	0.976	1.081	1.106	1.059	0.999	1.05	1.17	1.10
Q9C0B5	Palmitoyltransferase ZDHHC5 OS=Homo	ZDHHC5	77.544	38.491	7.7	5	6	5	1	0.94	1.013	1.02	1.048	1.031	0.992	0.95	1.05	1.07	1.00
Q9BTC0	Death-inducer obliterator 1 OS=Homo sa	DIDO1	243.87	114.37	10.1	16	16	16	0.961	0.942	1.014	0.98	1.072	1.019	1.025	1.031	1.05	1.10	1.08
Q9NUQ9	Protein FAM49B OS=Homo sapiens OX	FAM49B	36.748	154.6	45.1	11	15	9	0.991	0.934	0.999	1.018	0.983	0.972	1.12	1.041	1.05	1.02	1.12
Q15024	Exosome complex component RRP42 OS	EXOSC7	31.821	71.527	31.3	7	13	7	0.933	1	1.064	0.961	1.039	1.003	1.089	1.028	1.05	1.06	1.10
P05455	Lupus La protein OS=Homo sapiens OX=	SSB	46.836	164.9	35.5	17	27	17	1.009	0.988	1.053	1.039	0.945	0.937	1.08	0.984	1.05	0.94	1.03
Q92990	Glomulin OS=Homo sapiens OX=9606 (	GLMN	68.207	39.681	9.9	6	8	6	0.914	0.92	0.947	0.974	1.091	1.116	0.992	1.118	1.05	1.20	1.15
O60506	Heterogeneous nuclear ribonucleoprotein	SYNCRIP	69.602	242.88	35.6	20	38	14	0.971	1.013	1.035	1.043	0.998	1.02	0.932	0.953	1.05	1.02	0.95
Q15172	Serine/threonine-protein phosphatase 2A	PPP2R5A	56.193	41.418	9.5	4	5	4	1.049	0.893	1.061	0.973	1.109	0.946	1.056	1.015	1.05	1.06	1.07
Q5BKZ1	DBIRD complex subunit ZNF326 OS=H	ZNF326	65.653	233.38	25.6	12	17	12	0.979	0.964	1.006	1.029	1.039	1.182	0.861	1	1.05	1.14	0.96
A6NHR9	Structural maintenance of chromosomes f	SMCHD1	226.37	111.05	9.3	17	19	17	1.004	0.984	1.098	0.984	1.036	0.999	1.082	0.938	1.05	1.02	1.02
Q9Y4X5	E3 ubiquitin-protein ligase ARIH1 OS=H	ARIH1	64.117	51.066	11.5	6	6	6	1.006	0.94	1.011	1.027	1.008	0.999	1.041	0.959	1.05	1.03	1.03
P62820	Ras-related protein Rab-1A OS=Homo sa	RAB1A	22.677	70.509	59	9	32	4	1.028	0.9	1.026	0.993	1.005	0.912	1.118	1.046	1.05	0.99	1.12
Q9Y4K4	Mitogen-activated protein kinase kinase k	MAP4K5	95.023	61.731	14.2	9	9	9	0.944	1.051	1.065	1.024	1.037	1.184	0.83	0.987	1.05	1.11	0.91
P78527	DNA-dependent protein kinase catalytic s	PRKDC	469.08	323.31	22.6	82	111	78	0.955	0.934	0.968	1.01	1.048	1.04	0.99	1.048	1.05	1.11	1.08
Q9BPY3	Protein FAM118B OS=Homo sapiens O.	FAM118B	39.499	35.395	16.8	5	6	5	1.043	1.016	1.065	1.091	0.971	0.896	0.967	0.902	1.05	0.91	0.91
P82979	SAP domain-containing ribonucleoprotei	SARNP	23.671	81.049	29.5	5	7	5	1.009	0.948	1.055	0.994	1.042	1.144	0.915	0.976	1.05	1.12	0.97
Q07960	Rho GTPase-activating protein 1 OS=Ho	ARHGAP1	50.435	110.32	31.2	11	13	11	0.917	0.978	0.958	1.026	0.962	1.023	1.067	1.026	1.05	1.05	1.10
Q04323	UBX domain-containing protein 1 OS=H	UBXN1	33.325	55.78	23.6	4	4	4	0.963	1.06	1.104	1.014	0.989	0.976	1.072	0.93	1.05	0.97	0.99
Q6FI81	Anamorsin OS=Homo sapiens OX=9606	CIAPIN1	33.582	107	39.7	12	15	12	1.019	0.947	0.996	1.062	1.041	0.909	1.076	0.999	1.05	0.99	1.06
Q9GZN1	Actin-related protein 6 OS=Homo sapien	ACTR6	45.81	22.169	9.3	3	4	3	0.945	0.939	0.952	1.02	0.995	0.958	1.181	1.094	1.05	1.04	1.21
P22059	Oxysterol-binding protein 1 OS=Homo s	OSBP	89.42	104.25	19.2	13	16	13	1.029	0.922	1.017	1.025	1.038	0.987	1.029	0.97	1.05	1.04	1.02
Q16222	UDP-N-acetylhexosamine pyrophosphory	UAP1	58.768	24.001	11.5	4	5	3	0.956	0.781	0.837	0.981	1.228	1.008	1.163	1.147	1.05	1.29	1.33
O00458	Interferon-related developmental regulato	IFRD1	50.268	57.418	13.1	4	4	4	0.788	1.691	1.4	1.194	0.616	0.713	1.048	0.626	1.05	0.54	0.68
Q15382	GTP-binding protein Rheb OS=Homo sa	RHEB	20.497	30.404	20.1	3	6	3	1.009	1.039	1.104	1.039	0.946	1.119	0.767	0.854	1.05	1.01	0.79
Q01780	Exosome component 10 OS=Homo sapie	EXOSC10	100.83	149.72	27.3	16	24	16	0.943	0.978	0.992	1.018	1	1.143	0.93	1.016	1.05	1.12	1.01
Q14457	Beclin-1 OS=Homo sapiens OX=9606 G	BECN1	51.896	6.046	1.8	1	1	1	0.976	0.99	0.981	1.076	0.956	1.149	0.87	1.027	1.05	1.07	0.96
Q92797	Symplekin OS=Homo sapiens OX=9606	SYMPK	141.15	189.79	19.7	18	26	18	0.968	0.955	0.981	1.031	1.047	0.992	0.973	1.018	1.05	1.06	1.04
O75179	Ankyrin repeat domain-containing protei	ANKRD17	274.25	114.46	7.5	14	15	7	1.029	0.986	1.118	0.99	0.951	1.07	0.814	1.011	1.05	1.00	0.91
P49756	RNA-binding protein 25 OS=Homo sapie	RBM25	100.18	119.95	15.2	13	17	13	0.976	0.974	1.002	1.038	1.001	0.997	1.069	0.984	1.05	1.02	1.05
P62258	14-3-3 protein epsilon OS=Homo sapiens	YWHAE	29.174	123.49	54.1	15	54	13	0.987	1.007	1.05	1.036	0.95	0.993	0.995	0.983	1.05	0.97	0.99
Q96AG4	Leucine-rich repeat-containing protein 59	LRRC59	34.93	163.08	30.6	8	9	8	0.963	0.924	0.956	1.018	1.159	0.937	1.092	1.026	1.05	1.11	1.12
Q8NBK3	Formylglycine-generating enzyme OS=Hc	SUMF1	40.556	13.003	7.2	2	2	2	0.985	0.947	1.014	1.007	1.144	0.866	1.152	0.938	1.05	1.04	1.08
Q9HB71	Calcyclin-binding protein OS=Homo sap	CACYBP	26.21	110.23	28.1	7	12	7	0.966	0.967	0.996	1.026	1.001	1.09	0.968	0.996	1.05	1.08	1.02
P54136	Arginine--tRNA ligase, cytoplasmic OS=	RARS	75.378	238.18	42.6	25	49	25	0.964	1.014	1.055	1.014	0.976	0.984	1.01	0.979	1.05	0.99	1.01
P30291	Wee1-like protein kinase OS=Homo sapi	WEE1	71.597	50.771	12.7	7	9	7	0.927	0.921	0.94	0.993	1.072	1.226	0.893	1.035	1.05	1.24	1.04
Q92905	COP9 signalosome complex subunit 5 OS	COPS5	37.578	83.764	28.4	7	11	7	1.005	0.996	1.021	1.072	0.93	1.205	0.804	1.003	1.05	1.07	0.90
P19387	DNA-directed RNA polymerase II subuni	POLR2C	31.441	16.568	14.2	3	3	3	0.914	1.026	1.075	0.954	1.045	0.982	1.111	0.858	1.05	1.04	1.01
Q9UHQ9	NADH-cytochrome b5 reductase 1 OS=H	CYB5R1	34.094	105.95	42.6	10	17	10	0.971	0.955	1.042	0.972	0.983	1.04	1.142	1.035	1.05	1.05	1.13
Q9P265	Disco-interacting protein 2 homolog B O	DIP2B	171.49	88.01	10.2	11	13	10	0.976	0.951	0.996	1.019	1.024	0.955	1.035	1.024	1.05	1.03	1.07
P55735	Protein SEC13 homolog OS=Homo sapie	SEC13	35.54	85.879	41.9	9	12	8	0.964	0.965	0.991	1.026	1.001	1.089	0.923	1.001	1.05	1.08	1.00
O95347	Structural maintenance of chromosomes p	SMC2	135.65	235.53	23	26	36	26	0.974	0.956	1.003	1.015	1.057	1.039	0.957	0.979	1.05	1.09	1.00
O75400	Pre-mRNA-processing factor 40 homolog	PRPF40A	108.8	187.25	20.5	17	23	17	0.985	0.946	0.993	1.026	1.028	0.997	1.017	1.004	1.05	1.05	1.05
Q00610	Clathrin heavy chain 1 OS=Homo sapiens	CLTC	191.61	323.31	34.9	48	91	47	1	0.981	1.046	1.025	1.018	0.987	0.984	1.004	1.05	1.01	1.00
P57740	Nuclear pore complex protein Nup107 O	NUP107	106.37	101.74	12.4	9	14	9	0.941	0.978	0.999	1.007	1.038	1.116	0.919	1.073	1.05	1.12	1.04
Q9NQ92	Coordinator of PRMT5 and differentiatic	COPRS	20.066	7.7273	6.5	1	1	1	0.964	1.026	1.1	0.98	0.993	0.932	1.13	0.974	1.05	0.97	1.06
P50402	Emerin OS=Homo sapiens OX=9606 GN	EMD	28.994	146.45	42.5	8	13	8	0.976	0.948	1.046	0.965	1.065	1.112	0.951	0.971	1.05	1.13	1.00
Q9NW64	Pre-mRNA-splicing factor RBM22 OS=I	RBM22	46.895	71.457	23.8	10	12	10	0.99	0.89	1.001	0.964	1.106	1.131	0.977	1.01	1.05	1.19	1.06
Q9UBQ5	Eukaryotic translation initiation factor 3	EIF3K	25.059	109.28	41.7	6	7	6	0.956	1.049	1.071	1.024	0.981	0.954	1.026	0.885	1.04	0.97	0.95
O14662	Syntaxin-16 OS=Homo sapiens OX=960	STX16	37.031	29.192	11.4	3	4	3	0.961	1.003	1.066	0.986	1.089	0.96	1.033	0.997	1.04	1.04	1.03
Q9Y673	Dolichyl-phosphate beta-glucosyltransfer	ALG5	36.946	29.153	14.2	3	4	3	0.982	0.872	0.947	0.99	1.223	1.136	0.953	1.07	1.04	1.27	1.09
P31948	Stress-induced-phosphoprotein 1 OS=Hc	STIP1	62.639	254.94	46.2	26	46	26	1.005	0.984	1.039	1.039	0.986	0.98	0.985	0.977	1.04	0.99	0.99
O95218	Zinc finger Ran-binding domain-containi	ZRANB2	37.404	53.133	24.5	7	12	7	0.985	0.982	1.011	1.044	1.001	1.229	0.73	0.984	1.04	1.13	0.87
Q7L273	BTB/POZ domain-containing protein KC	KCTD9	42.566	14.922	6.7	2	2	2	0.818	0.814	0.724	0.981	1.24	0.953	1.294	1.395	1.04	1.34	1.65
Q9H7Z7	Prostaglandin E synthase 2 OS=Homo sa	PTGES2	41.943	23.928	10.9	4	4	4	0.928	0.973	0.924	1.062	1.007	1.044	1.056	1.075	1.04	1.08	1.12
P43246	DNA mismatch repair protein Msh2 OS=	MSH2	104.74	144.96	20.8	17	26	17	0.94	0.878	0.926	0.973	1.107	0.973	1.184	1.09	1.04	1.14	1.25
Q15286	Ras-related protein Rab-35 OS=Homo sa	RAB35	23.025	50.002	21.4	5	9	5	0.959	0.928	0.961	1.01	1.102	1.017	1.012	1.014	1.04	1.12	1.07
Q13247	Serine/arginine-rich splicing factor 6 OS=	SRSF6	39.586	56.281	19.2	6	11	4	1.003	0.974	1.036	1.029	0.978	1.088	0.915	0.964	1.04	1.05	0.95
Q8IXT5	RNA-binding protein 12B OS=Homo sap	RBM12B	118.1	41.115	13.4	7	7	7	0.919	0.879	0.783	1.095	1.05	0.993	0.968	1.173	1.04	1.14	1.19
P51114	Fragile X mental retardation syndrome-re	FXR1	69.72	175.79	24.3	12	13	11	1.047	1.05	1.142	1.048	0.941	0.917	0.942	0.86	1.04	0.89	0.86
P49005	DNA polymerase delta subunit 2 OS=Ho	POLD2	51.289	36.304	8.3	3	4	3	0.954	0.92	0.972	0.985	1.171	1.076	1.006	1.017	1.04	1.20	1.08
A8MV65	Transcription cofactor vestigial-like prote	VGLL3	36.009	6.8512	2.8	1	1	1	0.932	0.947	0.969	0.993	1.01	1.205	0.912	1.157	1.04	1.18	1.10
Q14254	Flotillin-2 OS=Homo sapiens OX=9606	FLOT2	47.064	49.305	17.3	7	9	7	0.923	1.072	1.051	1.032	1	0.97	1.127	1.097	1.04	0.99	1.11
Q13724	Mannosyl-oligosaccharide glucosidase O	MOGS	91.916	123.84	18.4	12	19	12	0.968	0.938	0.966	1.024	1.049	1.005	1.017	0.981	1.04	1.08	1.05
Q12955	Ankyrin-3 OS=Homo sapiens OX=9606	ANK3	480.4	47.889	2	7	8	6	0.974	0.917	1.008	0.966	1.051	0.976	1.145	1.066	1.04	1.07	1.17
Q9BRR8	G patch domain-containing protein 1 OS=	GPATCH1	103.34	6.2904	1	1	1	1	0.969	0.904	0.904	1.051	1.052	0.936	1.161	1.064	1.04	1.06	1.19
Q8WX92	Negative elongation factor B OS=Homo s	NELFB	65.697	55.641	16.9	8	10	8	1.005	0.917	1.013	0.993	1.064	0.944	1.08	1.024	1.04	1.04	1.09
Q9H3P2	Negative elongation factor A OS=Homo s	NELFA	57.276	26.106	8.7	3	4	3	0.962	1.006	1.043	1.011	0.996	1.349	0.813	0.98	1.04	1.19	0.91
Q9H4A6	Golgi phosphoprotein 3 OS=Homo sapie	GOLPH3	33.81	30.176	19.8	4	6	3	1.001	0.99	1.037	1.041	0.973	0.883	1.109	1.066	1.04	0.93	1.09
P37235	Hippocalcin-like protein 1 OS=Homo sap	HPCAL1	22.313	52.65	26.4	4	5	4	1.041	0.883	1.053	0.955	0.941	1.009	1.202	1.01	1.04	1.01	1.15
Q86WC4	Osteopetrosis-associated transmembrane	OSTM1	37.256	22.263	7.8	2	3	2	1.024	0.992	1.044	1.06	0.972	1.24	0.735	0.93	1.04	1.10	0.83
O95167	NADH dehydrogenase [ubiquinone] 1 alp	NDUFA3	9.2787	27.223	41.7	3	3	3	0.98	1.018	1.094	0.991	0.982	1.081	1.001	0.931	1.04	1.03	0.97
Q8N122	Regulatory-associated protein of mTOR (	RPTOR	149.04	33.469	4.2	5	6	5	0.997	0.917	0.991	1.006	1.027	1.044	0.991	0.967	1.04	1.08	1.02
Q7Z3J2	UPF0505 protein C16orf62 OS=Homo sa	C16orf62	109.56	45.385	7.3	6	7	6	0.974	0.919	0.987	0.988	1.133	1.013	1.069	0.997	1.04	1.13	1.09
P42345	Serine/threonine-protein kinase mTOR O	MTOR	288.89	131.13	8.5	17	18	17	0.946	0.976	1.017	0.988	1.129	1.013	0.977	1.032	1.04	1.11	1.05
Q9UBU9	Nuclear RNA export factor 1 OS=Homo	NXF1	70.182	67.195	18.9	10	14	10	0.978	0.923	0.979	1.004	1.033	0.948	1.078	1.007	1.04	1.04	1.10
Q9Y2G3	Probable phospholipid-transporting ATP	ATP11B	134.19	11.102	3.1	3	3	2	0.987	1.01	1.002	1.081	0.989	0.937	1.021	0.978	1.04	0.96	1.00
Q15102	Platelet-activating factor acetylhydrolase	PAFAH1B3	25.734	20.817	14.7	3	4	3	1.035	0.939	1.032	1.027	1.023	1.071	0.878	0.97	1.04	1.06	0.94
Q4G0J3	La-related protein 7 OS=Homo sapiens C	LARP7	66.898	47.192	11.3	6	7	6	0.895	0.969	0.951	0.993	1.055	0.97	1.191	1.188	1.04	1.09	1.28
Q53GS9	U4/U6.U5 tri-snRNP-associated protein 2	USP39	65.38	90.506	23.2	10	14	10	0.976	0.958	0.984	1.033	1.066	0.983	0.95	0.98	1.04	1.06	1.00
Q93074	Mediator of RNA polymerase II transcrip	MED12	243.08	63.19	5	9	10	9	1.048	0.934	1.07	0.997	0.998	1.138	0.86	0.999	1.04	1.08	0.94
Q92575	UBX domain-containing protein 4 OS=H	UBXN4	56.777	19.799	8.7	3	4	3	1.009	0.951	1.043	1.001	1.019	0.989	0.948	0.955	1.04	1.02	0.97
Q6P2E9	Enhancer of mRNA-decapping protein 4	EDC4	151.66	184.65	20.4	18	22	18	1.004	0.981	1.031	1.039	0.982	1.185	0.971	0.939	1.04	1.09	0.96
Q9Y6G9	Cytoplasmic dynein 1 light intermediate (	DYNC1LI1	56.578	116.26	37.3	15	21	14	0.983	0.91	0.957	1.017	1.075	0.974	1.035	1.091	1.04	1.08	1.12
Q9NUW8	Tyrosyl-DNA phosphodiesterase 1 OS=H	TDP1	68.42	29.981	9.2	5	5	5	0.988	0.952	1.003	1.02	1.044	1.037	1.087	0.914	1.04	1.07	1.03
O75165	DnaJ homolog subfamily C member 13 O	DNAJC13	254.41	212.52	12.5	23	27	23	0.977	0.95	0.961	1.048	1.067	1.004	1.02	1.043	1.04	1.07	1.07
O14737	Programmed cell death protein 5 OS=Ho	PDCD5	14.285	35.9	28	3	7	3	0.917	1.037	1.08	0.957	0.993	1.212	0.82	0.973	1.04	1.13	0.92
Q9NT62	Ubiquitin-like-conjugating enzyme ATG3	ATG3	35.864	43.131	21.3	5	7	5	0.998	0.963	1.011	1.033	0.971	0.974	1.134	0.91	1.04	0.99	1.04
Q9H9H4	Vacuolar protein sorting-associated prote	VPS37B	31.307	17.233	13	2	2	2	0.944	0.855	0.816	1.059	1.084	0.857	1.206	1.238	1.04	1.08	1.36
Q86VP6	Cullin-associated NEDD8-dissociated pr	CAND1	136.37	272.29	27.8	30	52	30	1.005	0.985	1.065	1.009	0.984	0.978	0.984	0.963	1.04	0.99	0.98
P22735	Protein-glutamine gamma-glutamyltransfe	TGM1	89.786	12.639	3.1	2	2	2	0.851	0.811	0.694	1.038	1.03	1.028	1.278	1.425	1.04	1.24	1.63
P32321	Deoxycytidylate deaminase OS=Homo sa	DCTD	20.016	42.876	31.5	5	9	5	1	1.067	1.107	1.047	0.979	0.971	0.97	0.971	1.04	0.94	0.94
P78344	Eukaryotic translation initiation factor 4	EIF4G2	102.36	176.22	25.4	19	26	19	0.977	1.001	1.044	1.017	0.991	0.983	1.057	1.007	1.04	1.00	1.04
Q8IUW5	RELT-like protein 1 OS=Homo sapiens (	RELL1	29.34	6.4861	3.3	1	1	1	1.061	0.728	0.907	0.957	1.243	0.92	1.03	1.196	1.04	1.21	1.24
Q13153	Serine/threonine-protein kinase PAK 1 O	PAK1	60.646	117.44	33.6	14	17	9	0.988	0.926	0.951	1.043	0.994	1.003	1.121	1.049	1.04	1.04	1.13
Q5VU43	Myomegalin OS=Homo sapiens OX=960	PDE4DIP	265.1	7.8058	0.5	1	1	1	0.999	1.014	1.083	1.014	1	1.01	1.014	0.912	1.04	1.00	0.96
Q8WVJ2	NudC domain-containing protein 2 OS=I	NUDCD2	17.676	22.409	19.1	3	4	3	1.041	1	1.105	1.021	0.938	0.836	1.154	0.906	1.04	0.87	1.01
O00410	Importin-5 OS=Homo sapiens OX=9606	IPO5	123.63	318.71	28.5	23	44	22	0.979	0.996	1.032	1.025	0.993	1.094	0.931	0.914	1.04	1.06	0.93
Q8TBC4	NEDD8-activating enzyme E1 catalytic s	UBA3	51.852	66.254	24.6	9	12	9	0.98	0.954	1.005	1.009	1.027	1.077	0.994	1.048	1.04	1.09	1.06
P00491	Purine nucleoside phosphorylase OS=Ho	PNP	32.118	245.15	58.1	14	29	14	0.967	0.946	1.017	0.975	1.056	1.241	0.933	0.998	1.04	1.20	1.01
P18583	Protein SON OS=Homo sapiens OX=96(	SON	263.83	139.3	10.3	20	25	19	0.984	0.992	1.014	1.043	0.949	0.966	1.021	0.956	1.04	0.97	1.00
P46100	Transcriptional regulator ATRX OS=Hor	ATRX	282.58	70.939	5.5	9	9	9	0.993	0.987	1.051	1.01	1.041	1.009	1.013	0.895	1.04	1.04	0.96
Q06330	Recombining binding protein suppressor	RBPJ	55.637	32.038	10	4	5	4	0.963	1.017	1.026	1.035	0.991	0.941	1.06	0.976	1.04	0.98	1.03
Q9P258	Protein RCC2 OS=Homo sapiens OX=9(	RCC2	56.084	198.12	41	15	26	15	0.981	1.002	1.03	1.034	0.984	1.017	0.953	0.976	1.04	1.01	0.97
O95486	Protein transport protein Sec24A OS=Ho	SEC24A	119.75	97.203	9	7	12	7	1.014	0.97	1.04	1.025	1.032	1.061	0.922	0.993	1.04	1.05	0.97
Q15061	WD repeat-containing protein 43 OS=Ho	WDR43	74.89	53.881	13.6	8	10	7	0.99	1.048	1.055	1.066	0.945	0.982	0.902	0.959	1.04	0.95	0.91
Q9UI10	Translation initiation factor eIF-2B subu	EIF2B4	57.557	42.551	9.2	3	4	3	0.97	0.975	1.058	0.966	1.044	1.062	1.17	1.161	1.04	1.08	1.20
Q9H4P4	E3 ubiquitin-protein ligase NRDP1 OS=	RNF41	35.905	12.934	9.8	2	2	2	1.026	1.044	1.272	0.882	1.029	1.178	0.86	0.832	1.04	1.07	0.82
Q8N680	Zinc finger and BTB domain-containing	ZBTB2	57.336	11.738	7.2	2	2	2	0.873	0.977	0.936	0.989	1.048	1.408	0.846	1.097	1.04	1.33	1.05

P13667	Protein disulfide-isomerase A4 OS=Hom	PDIA4	72.932	323.31	46.4	34	58	34	0.991	0.983	1.037	1.017	0.99	1.003	0.99	0.997	1.04	1.01	1.01
P49760	Dual specificity protein kinase CLK2 OS	CLK2	60.089	15.037	5.6	2	2	2	0.946	0.956	1.021	0.958	1.289	1.179	0.825	0.952	1.04	1.30	0.93
P51531	Probable global transcription activator SI	SMARCA2	181.28	112.45	11.3	16	19	9	0.95	0.929	0.933	1.022	1.104	1.207	0.893	0.987	1.04	1.23	1.00
Q7Z6K3	Protein prenyltransferase alpha subunit re	PTAR1	46.405	17.739	6.7	3	3	3	0.98	0.951	1.022	0.987	1.145	1.017	0.97	1.008	1.04	1.12	1.02
Q9GZP4	PITH domain-containing protein 1 OS=H	PITHD1	24.178	12.929	15.6	2	2	2	1.023	0.909	0.985	1.025	1.086	0.885	1.069	1.034	1.04	1.02	1.09
P35998	26S proteasome regulatory subunit 7 OS=	PSMC2	48.633	165.72	47.3	19	29	18	0.98	0.986	1.004	1.041	0.994	1.048	0.93	1.017	1.04	1.04	0.99
Q9P253	Vacuolar protein sorting-associated prote	VPS18	110.18	113.94	16.3	12	14	12	1.016	0.976	1.037	1.035	1.058	0.96	1.017	1.032	1.04	1.01	1.03
Q8IY18	Structural maintenance of chromosomes p	SMC5	128.81	70.622	7.6	8	10	8	0.965	0.903	0.97	0.973	1.145	0.925	1.205	1.041	1.04	1.11	1.20
Q9UNF1	Melanoma-associated antigen D2 OS=Hc	MAGED2	64.953	120.04	19.6	12	17	12	0.944	0.95	0.977	0.993	1.104	1.084	1.094	1.057	1.04	1.16	1.14
Q99575	Ribonucleases P/MRP protein subunit P(	POP1	114.71	61.929	8.8	6	6	6	0.947	0.972	1.073	0.923	1.089	1.15	0.982	0.972	1.04	1.17	1.02
O43681	ATPase ASNA1 OS=Homo sapiens OX=	ASNA1	38.792	49.271	19.3	5	8	5	0.99	1.005	1.061	1.014	0.959	1.008	1.028	1.022	1.04	0.99	1.03
Q15650	Activating signal cointegrator 1 OS=Hon	TRIP4	66.145	58.379	18.1	7	7	7	0.985	1.01	1.07	1.005	0.979	1.13	0.901	1.021	1.04	1.06	0.96
Q5T8P6	RNA-binding protein 26 OS=Homo sapie	RBM26	113.6	112.58	16.4	15	19	15	1.002	0.971	1.045	1.007	1.064	1.035	0.994	1.027	1.04	1.06	1.02
O95479	GDH/6PGL endoplasmic bifunctional pro	H6PD	88.892	6.482	1.9	1	1	1	0.999	0.999	1.115	0.963	0.96	0.907	1.146	0.995	1.04	0.93	1.07
Q9HC36	rRNA methyltransferase 3, mitochondrial	MRM3	47.019	51.557	15	6	7	6	0.994	0.934	1.004	1.001	1.091	1.307	0.717	1.041	1.04	1.24	0.91
P56192	Methionine--tRNA ligase, cytoplasmic O	MARS	101.11	208.23	31.7	22	31	22	0.953	0.98	0.984	1.026	0.972	0.964	1.063	1.052	1.04	1.00	1.09
Q9NVE7	Pantothenate kinase 4 OS=Homo sapiens	PANK4	85.99	45.386	9.2	6	7	6	0.954	0.981	0.989	1.023	1.078	0.947	1.1	0.994	1.04	1.05	1.08
O00268	Transcription initiation factor TFIID subu	TAF4	110.11	50.476	7.2	6	6	6	1.002	1.036	1.077	1.042	1.014	0.97	0.986	0.892	1.04	0.97	0.92
O14753	Putative transcription factor Ovo-like 1 C	OVOL1	30.259	35.783	9.4	2	3	2	1.049	1.016	1.174	0.973	1.151	1.011	0.835	0.823	1.04	1.05	0.80
Q96GX2	Ataxin-7-like protein 3B OS=Homo sapie	ATXN7L3B	10.771	9.895	30.9	2	1	1	0.951	1.041	0.951	1.12	0.893	0.873	1.139	1.037	1.04	0.89	1.09
O00139	Kinesin-like protein KIF2A OS=Homo sa	KIF2A	79.954	159.44	29.5	16	21	14	0.963	0.955	1.022	0.972	1.057	1.254	0.899	0.974	1.04	1.20	0.98
P40222	Alpha-taxilin OS=Homo sapiens OX=96	TXLNA	61.89	116.9	23.3	12	18	12	0.991	0.978	1.032	1.015	1.029	1.081	0.962	0.947	1.04	1.07	0.97
P20585	DNA mismatch repair protein Msh3 OS=	MSH3	127.41	69.172	9	9	11	9	0.922	1.025	1.022	1.002	0.973	1.065	1.102	1.006	1.04	1.05	1.08
O95071	E3 ubiquitin-protein ligase UBR5 OS=H	UBR5	309.35	146.97	7.3	15	18	15	0.971	1.029	1.055	1.024	0.97	0.953	0.994	0.916	1.04	0.96	0.96
Q7Z739	YTH domain-containing family protein 3	YTHDF3	63.86	19.198	9.4	5	5	2	1.05	1.03	1.237	0.925	0.984	1.13	0.878	0.836	1.04	1.02	0.82
P30876	DNA-directed RNA polymerase II subuni	POLR2B	133.9	105.83	11.8	11	14	11	1.02	0.889	0.991	0.993	1.012	0.934	0.976	1.052	1.04	1.02	1.06
Q13136	Liprin-alpha-1 OS=Homo sapiens OX=9	PPFIA1	135.78	89.381	13	12	13	12	0.959	1.055	1.038	1.055	0.984	1.165	0.859	0.964	1.04	1.07	0.91
Q8WUM0	Nuclear pore complex protein Nup133 O	NUP133	128.98	195.15	17.5	15	20	15	0.939	0.959	0.988	0.984	1.047	1.161	0.912	1.029	1.04	1.16	1.02
Q96PV6	Leukocyte receptor cluster member 8 OS=	LENG8	88.156	27.884	7.1	4	4	4	0.928	0.714	0.764	0.942	1.32	0.966	1.171	1.353	1.04	1.39	1.54
O00422	Histone deacetylase complex subunit SAI	SAP18	17.561	34.167	20.3	4	8	4	0.973	0.953	1.03	0.971	1.057	0.993	1.137	0.995	1.04	1.06	1.11
Q7Z4R8	UPF0669 protein C6orf120 OS=Homo sa	C6orf120	20.772	20.829	14.1	1	2	1	0.969	1.06	1.061	1.047	0.991	0.949	1.001	0.968	1.04	0.96	0.97
Q9NTJ3	Structural maintenance of chromosomes p	SMC4	147.18	230.06	23.9	27	37	26	1.008	1.024	1.044	1.067	0.962	1.111	0.853	0.945	1.04	1.02	0.88
Q9Y6W5	Wiskott-Aldrich syndrome protein family	WASF2	54.283	53.986	20.9	8	7	7	0.982	1.05	1.055	1.056	0.949	0.891	1.119	0.95	1.04	0.91	1.02
Q9Y6D5	Brefeldin A-inhibited guanine nucleotide	ARFGEF2	202.04	140.61	13.4	19	19	13	0.999	0.957	1.023	1.009	1.041	0.985	1.039	0.944	1.04	1.04	1.01
Q8NFC6	Biorientation of chromosomes in cell divi	BOD1L1	330.46	107.01	4.6	11	11	9	1.011	1.027	1.05	1.067	0.943	1.134	0.847	0.904	1.04	1.02	0.86
Q5TBB1	Ribonuclease H2 subunit B OS=Homo sa	RNASEH2	35.138	74.361	29.5	9	11	9	0.946	0.938	0.99	0.967	1.171	1.002	1.09	0.99	1.04	1.15	1.10
Q3ZCM7	Tubulin beta-8 chain OS=Homo sapiens	TUBB8	49.775	12.232	21.4	9	26	1	0.666	1.951	1.712	1.006	0.301	1.78	0.547	0.365	1.04	0.80	0.35
O14672	Disintegrin and metalloproteinase domain	ADAM10	84.141	78.439	18.2	9	8	8	0.948	0.92	0.914	1.026	1.047	0.891	1.197	1.12	1.04	1.04	1.24
P34932	Heat shock 70 kDa protein 4 OS=Homo s	HSPA4	94.33	323.31	52.4	38	78	33	1.023	0.95	1.034	1.015	1.005	0.968	1.035	0.986	1.04	1.00	1.02
Q9H2D6	TRIO and F-actin-binding protein OS=H	TRIOBP	261.37	35.878	3.2	6	7	5	1.01	0.942	1.021	1.006	1.075	1.221	0.732	1.079	1.04	1.18	0.93
Q9UI12	V-type proton ATPase subunit H OS=Ho	ATP6V1H	55.882	109.36	33.3	11	16	11	0.993	0.96	0.996	1.032	1.024	1.014	0.921	1.041	1.04	1.04	1.00
O15160	DNA-directed RNA polymerases I and III	POLR1C	39.249	51.9	19.7	5	5	5	1.016	0.896	0.934	1.051	0.958	0.985	1.095	1.054	1.04	1.02	1.12
Q9Y4A5	Transformation/transcription domain-ass	TRRAP	437.6	116	4.2	13	14	13	0.922	0.996	0.978	1.013	1.04	1.221	0.984	0.913	1.04	1.18	0.99
Q7L5D6	Golgi to ER traffic protein 4 homolog OS	GET4	36.504	44.211	25.7	6	8	6	1.016	0.959	1.032	1.018	0.986	0.972	1.11	1	1.04	0.99	1.07
Q02818	Nucleobindin-1 OS=Homo sapiens OX=9	NUCB1	53.879	118.68	38.4	14	22	14	0.977	0.925	0.965	1.009	1.066	1.072	1.022	1.047	1.04	1.12	1.09
P26358	DNA (cytosine-5)-methyltransferase 1 OS	DNMT1	183.16	306.93	23.9	32	41	32	0.932	0.944	0.969	0.978	1.073	1.138	1.018	1.058	1.04	1.18	1.11
P14866	Heterogeneous nuclear ribonucleoprotein	HNRNPL	64.132	323.31	42.8	19	54	18	0.994	0.989	1.028	1.03	0.969	0.999	0.977	1.028	1.04	0.99	1.01
Q13488	V-type proton ATPase 116 kDa subunit a	TCIRG1	92.967	66.101	11.8	8	8	8	0.969	1.016	1.009	1.051	0.993	1.003	1.065	1.09	1.04	1.01	1.09
P07919	Cytochrome b-c1 complex subunit 6, mit	UQCRH	10.739	40.345	19.8	1	2	1	0.842	1.093	0.963	1.045	0.961	0.856	1.267	1.123	1.04	0.94	1.24
Q92499	ATP-dependent RNA helicase DDX1 OS	DDX1	82.431	297.09	44.9	29	54	28	0.993	1	1.058	1.01	0.991	0.979	1.02	0.984	1.04	0.99	1.01
P23381	Tryptophan--tRNA ligase, cytoplasmic O	WARS	53.165	153.95	29.1	12	17	12	1.018	1.004	1.065	1.033	0.962	0.94	1.109	0.933	1.04	0.94	1.01
P05114	Non-histone chromosomal protein HMG-	HMGN1	10.659	36.797	43	5	6	5	1.002	0.998	1.073	1.002	1.045	0.994	1.126	0.939	1.04	1.02	1.03
Q96HY6	DDRGK domain-containing protein 1 OS	DDRGK1	35.61	22.487	14.6	3	4	3	0.971	1.06	1.163	0.944	0.987	1.255	0.913	0.794	1.04	1.10	0.84
P19623	Spermidine synthase OS=Homo sapiens (	SRM	33.824	76.59	39.4	8	14	8	1.019	0.934	1.006	1.02	1.087	0.981	0.963	0.945	1.04	1.06	0.98
Q6PIU2	Neutral cholesterol ester hydrolase 1 OS=	NCEH1	45.807	38.65	11	4	4	4	1.002	0.874	0.979	0.967	1.104	0.904	1.156	1.092	1.04	1.07	1.20
Q8IY67	Ribonucleoprotein PTB-binding 1 OS=H	RAVER1	63.876	41.317	9.6	4	5	4	0.867	1.013	0.997	0.953	0.948	1.096	1.077	1.121	1.04	1.09	1.17
P36551	Oxygen-dependent coproporphyrinogen-I	CPOX	50.151	93.885	26	11	14	11	0.987	0.98	1.027	1.013	1.014	0.934	1.109	1.026	1.04	0.99	1.09
Q14697	Neutral alpha-glucosidase AB OS=Homo	GANAB	106.87	247.61	22.6	19	38	19	0.996	0.972	1.041	1	1.014	0.987	0.994	0.976	1.04	1.02	1.00
Q15435	Protein phosphatase 1 regulatory subunit	PPP1R7	41.564	84.537	26.9	9	12	9	0.991	1.004	1.036	1.033	0.979	0.997	0.956	0.937	1.04	0.99	0.95
Q96IZ7	Serine/Arginine-related protein 53 OS=H	RSRC1	38.677	14.219	8.4	2	3	2	0.973	1.023	1.066	1.004	0.995	1	0.992	1.026	1.04	1.00	1.01
Q9P0K7	Ankycorbin OS=Homo sapiens OX=960(	RAI14	110.04	146.75	20.5	17	19	17	0.984	0.934	1.011	0.978	0.986	0.957	1.095	1.09	1.04	1.01	1.14
P13747	HLA class I histocompatibility antigen, al	HLA-E	40.156	7.8899	7.5	2	3	1	0.966	0.986	0.973	1.051	1.028	1.235	0.864	0.942	1.04	1.16	0.93
P46937	Transcriptional coactivator YAP1 OS=Hc	YAP1	54.461	116.03	28.4	8	9	8	0.949	1.009	1.006	1.024	1.098	1.156	0.965	1	1.04	1.15	1.00
Q9Y6X3	MAU2 chromatid cohesion factor homolo	MAU2	69.081	57.058	9.5	4	5	4	0.961	0.946	0.927	1.05	1.146	0.956	0.96	1.105	1.04	1.10	1.08
O00233	26S proteasome non-ATPase regulatory s	PSMD9	24.682	42.238	27.4	6	7	6	0.961	1.032	1.04	1.026	1.038	1.154	0.825	0.969	1.04	1.10	0.90
Q8TCJ2	Dolichyl-diphosphooligosaccharide--prot	STT3B	93.673	26.493	5	4	4	4	1.003	0.992	1.051	1.017	0.981	1.039	0.922	1.041	1.04	1.01	0.98
Q7Z7H8	39S ribosomal protein L10, mitochondria	MRPL10	29.282	14.589	10.3	2	2	2	0.785	1.414	1.311	0.968	0.927	0.993	0.952	0.907	1.04	0.87	0.85
P50552	Vasodilator-stimulated phosphoprotein C	VASP	39.829	169.13	34.7	10	19	10	1.005	1.033	1.077	1.035	0.893	0.969	0.973	0.916	1.04	0.91	0.93
Q14789	Golgin subfamily B member 1 OS=Homo	GOLGB1	376.01	323.31	20.3	52	57	52	0.974	0.962	1.011	0.995	1.035	1.037	1.057	0.997	1.04	1.07	1.06
Q00839	Heterogeneous nuclear ribonucleoprotein	HNRNPU	90.583	323.31	36	23	69	22	0.973	0.97	0.996	1.017	1.04	0.989	1.026	1.021	1.04	1.04	1.05
P31937	3-hydroxyisobutyrate dehydrogenase, mit	HIBADH	35.329	47.114	23.8	6	9	6	1.006	0.943	0.978	1.041	1.053	0.989	1.037	1.031	1.04	1.05	1.06
Q8WVM8	Sec1 family domain-containing protein 1	SCFD1	72.379	217.06	31.3	15	21	14	1.002	1.003	1.1	0.977	1.099	0.981	0.95	0.933	1.04	1.04	0.94
P57076	Cilia- and flagella-associated protein 298	CFAP298	33.224	13.134	9.7	2	2	2	0.92	1.034	0.992	1.032	1.008	1.14	0.942	1.031	1.04	1.10	1.01
P62191	26S proteasome regulatory subunit 4 OS=	PSMC1	49.184	173.98	41.6	16	24	16	0.948	1.011	1.03	0.999	0.965	1.053	0.972	0.964	1.04	1.03	0.99
Q96DI7	U5 small nuclear ribonucleoprotein 40 kI	SNRNP40	39.31	169.03	38.7	10	12	9	0.991	1.024	1.09	0.997	1.032	1.091	0.932	0.99	1.04	1.05	0.95
O00505	Importin subunit alpha-4 OS=Homo sapi	KPNA3	57.81	18.929	9.8	5	10	2	1.006	0.954	1.059	0.971	1.121	0.853	1.06	1.098	1.04	1.01	1.10
Q6BCY4	NADH-cytochrome b5 reductase 2 OS=H	CYB5R2	31.458	42.502	21	5	7	5	0.979	0.981	0.979	1.051	0.945	0.893	1.179	1.048	1.04	0.94	1.14
Q9UBR2	Cathepsin Z OS=Homo sapiens OX=960	CTSZ	33.868	29.688	12.9	3	4	3	1.042	0.892	1.007	0.996	1.147	1.011	0.9	0.984	1.04	1.12	0.97
P49792	E3 SUMO-protein ligase RanBP2 OS=H	RANBP2	358.2	323.31	23.8	58	82	35	0.991	0.972	1.035	0.998	1.011	1.038	0.952	0.96	1.04	1.04	0.97
O94804	Serine/threonine-protein kinase 10 OS=H	STK10	112.13	130.62	19.7	17	19	17	0.971	0.909	0.929	1.018	1.016	0.971	1.131	1.129	1.04	1.06	1.20
Q09472	Histone acetyltransferase p300 OS=Homo	EP300	264.16	94.933	5.6	11	13	8	0.93	1.035	1.022	1.013	0.981	1.131	0.962	0.983	1.04	1.07	0.99
Q9UKM7	Endoplasmic reticulum mannosyl-oligosa	MAN1B1	79.579	20.456	5	3	4	3	1.032	0.942	1.002	1.042	0.983	1.121	0.948	0.928	1.04	1.07	0.95
Q9Y388	RNA-binding motif protein, X-linked 2 (	RBMX2	37.335	19.608	10.2	3	5	2	0.964	0.983	1.052	0.964	1.04	1.034	0.999	1.126	1.04	1.07	1.09
Q9HD20	Manganese-transporting ATPase 13A1 O	ATP13A1	132.95	120.88	18.1	16	18	15	1.012	0.999	1.059	1.023	0.964	1.141	0.873	0.912	1.04	1.05	0.89
Q9H553	Alpha-1,3/1,6-mannosyltransferase ALG2	ALG2	47.091	26.778	11.5	4	6	4	1.023	0.997	1.041	1.05	0.982	0.962	0.983	0.968	1.04	0.96	0.97
Q14839	Chromodomain-helicase-DNA-binding p	CHD4	218	323.31	18.5	30	35	25	1.031	0.966	1.038	1.029	1.011	0.987	0.964	0.947	1.04	1.00	0.96
P06730	Eukaryotic translation initiation factor 4E	EIF4E	25.097	26.584	15.2	3	5	3	1.007	1.025	1.039	1.064	0.921	1.144	0.753	0.926	1.03	1.02	0.83
Q6RW13	Type-1 angiotensin II receptor-associated	AGTRAP	17.419	14.864	13.8	1	2	1	1.03	1.06	1.138	1.025	0.966	0.871	0.981	0.943	1.03	0.88	0.92
Q9UNN5	FAS-associated factor 1 OS=Homo sapie	FAF1	73.953	51.71	12.6	6	6	6	1.024	0.988	1.081	1.001	1.098	0.962	0.918	0.936	1.03	1.02	0.92
O95140	Mitofusin-2 OS=Homo sapiens OX=960	MFN2	86.401	115.51	17	12	14	12	0.936	0.904	0.913	0.991	1.081	0.978	1.207	1.173	1.03	1.12	1.29
Q9Y235	C->U-editing enzyme APOBEC-2 OS=H	APOBEC2	25.703	6.5308	3.6	1	1	1	0.915	0.725	0.726	0.971	1.257	0.932	1.161	1.468	1.03	1.33	1.60
Q9BST9	Rhotekin OS=Homo sapiens OX=9606 (	RTKN	62.667	99.765	25.8	11	12	11	0.957	1.001	1.011	1.015	1.015	1.01	1.04	1.05	1.03	1.03	1.07
Q53GQ0	Very-long-chain 3-oxoacyl-CoA reductas	HSD17B12	34.324	37.24	19.9	5	6	5	0.993	0.969	1.018	1.012	1.021	1.116	0.965	0.951	1.03	1.09	0.98
Q9Y6W3	Calpain-7 OS=Homo sapiens OX=9606 (	CAPN7	92.651	6.1435	1.2	1	1	1	0.985	1.011	1.011	1.054	1.005	0.995	1.074	0.886	1.03	1.00	0.98
Q9BX68	Histidine triad nucleotide-binding protei	HINT2	17.162	80.862	47.2	5	8	5	0.951	0.903	0.953	0.965	1.066	1.182	0.918	0.965	1.03	1.21	1.02
Q96CU9	FAD-dependent oxidoreductase domain-(	FOXRED1	53.811	46.146	14.2	5	6	5	0.97	0.971	1.05	0.958	1.155	1.062	0.918	0.88	1.03	1.14	0.93
Q96F07	Cytoplasmic FMR1-interacting protein 2	CYFIP2	148.4	94.597	11.3	14	16	5	0.983	0.996	1.053	0.994	1	0.971	0.967	0.981	1.03	1.00	0.98
O14562	Ubiquitin domain-containing protein UBI	UBFD1	33.382	41.333	13.6	3	3	3	0.99	1.05	1.038	1.072	0.943	1.109	0.843	0.971	1.03	1.01	0.89
Q8NAV1	Pre-mRNA-splicing factor 38A OS=Hom	PRPF38A	37.476	10.177	3.8	1	2	1	0.964	0.874	0.987	0.914	0.982	1.018	1.229	1.187	1.03	1.09	1.31
O00443	Phosphatidylinositol 4-phosphate 3-kinas	PIK3C2A	190.68	62.587	6.3	9	12	9	0.925	1.003	1.045	0.949	1.129	1.087	0.908	0.993	1.03	1.15	0.99
Q9UL18	Protein argonaute-1 OS=Homo sapiens C	AGO1	97.213	56.618	12.6	8	8	5	1.006	0.926	0.954	1.044	1.058	1.074	0.886	1.078	1.03	1.10	1.02
Q52LW3	Rho GTPase-activating protein 29 OS=H	ARHGAP2	142.06	13.29	2.4	2	2	2	1.158	0.865	1.118	0.974	1.012	0.634	1.272	0.906	1.03	0.81	1.08
Q92973	Transportin-1 OS=Homo sapiens OX=96	TNPO1	102.35	172.81	22.3	16	37	11	0.984	0.959	1.02	0.989	1.005	1.085	0.879	0.977	1.03	1.08	0.96
Q15717	ELAV-like protein 1 OS=Homo sapiens (	ELAVL1	36.091	132.08	35.3	8	16	8	0.969	1.008	1.042	1.002	1.034	0.987	1.021	0.994	1.03	1.02	1.02
Q9Y2V2	Calcium-regulated heat-stable protein 1 (	CARHSP1	15.892	6.6302	5.4	1	1	1	1.018	0.961	1.045	1.001	0.93	0.92	1.096	1.074	1.03	0.93	1.10
O15305	Phosphomannomutase 2 OS=Homo sapie	PMM2	28.082	58.579	33.7	8	12	8	0.987	0.969	1.027	0.995	1.01	0.938	1.032	0.985	1.03	1.00	1.03
Q9H3H1	tRNA dimethylallyltransferase OS=Homo	TRIT1	52.725	13.663	5.4	2	2	2	0.981	1.006	1.067	0.987	1.138	0.828	1.138	0.928	1.03	0.99	1.04
Q9UKY7	Protein CDV3 homolog OS=Homo sapie	CDV3	27.335	127.22	54.3	10	17	10	1.005	0.923	0.974	1.019	0.984	0.95	1.133	1.1	1.03	1.00	1.16
Q14204	Cytoplasmic dynein 1 heavy chain 1 OS=	DYNC1H1	532.4	323.31	30.7	122	169	120	0.985	0.98	1.014	1.017	0.981	0.966	1.078	1.026	1.03	0.99	1.07
Q9HCE5	N6-adenosine-methyltransferase non-cata	METTL14	52.15	12.607	4.6	2	2	2	0.915	0.966	0.894	1.05	1.018	0.839	1.264	1.139	1.03	0.99	1.28
Q7L576	Cytoplasmic FMR1-interacting protein 1	CYFIP1	145.18	50.878	10.2	12	16	3	1.039	0.902	0.988	1.018	1.034	0.832	1.128	0.972	1.03	0.96	1.08
P11172	Uridine 5'-monophosphate synthase OS=	UMPS	52.221	58.683	18.5	8	10	8	0.965	0.921	0.922	1.027	1.018	1.015	1.007	1.078	1.03	1.08	1.11
Q5JRX3	Presequence protease, mitochondrial OS=	PITRM1	117.41	230.57	27.1	23	28	23	0.954	0.935	0.941	1.011	1.073	1.047	1.027	1.091	1.03	1.12	1.12
P51965	Ubiquitin-conjugating enzyme E2 E1 OS	UBE2E1	21.404	11.895	15	2	3	1	0.924	1.055	0.979	1.066	0.953	1.271	0.813	1.012	1.03	1.12	0.92
P15924	Desmoplakin OS=Homo sapiens OX=96	DSP	331.77	323.31	35.6	91	142	90	1.03	1.011	1.057	1.052	0.964	0.936	0.989	0.896	1.03	0.93	0.92
P51580	Thiopurine S-methyltransferase OS=Hon	TPMT	28.18	19.68	13.5	3	4	3	1.002	1.009	1.073	1.005	1.003	1.14	0.816	1.008	1.03	1.07	0.91
P57678	Gem-associated protein 4 OS=Homo sapi	GEMIN4	120.04	84.052	12.3	12	13	12	0.984	0.942	0.99	1	1.061	1.023	1.019	1.071	1.03	1.08	1.09
P67936	Tropomyosin alpha-4 chain OS=Homo sa	TPM4	28.521	197.22	45.6	15	17	10	0.999	0.904	0.941	1.025	0.974	0.859	1.165	1.102	1.03	0.96	1.19
P78364	Polyhomeotic-like protein 1 OS=Homo s	PHC1	105.53	24.633	5.9	4	3	3	0.922	0.891	0.842	1.031	1.009	1.422	0.734	1.264	1.03	1.34	1.10
Q99570	Phosphoinositide 3-kinase regulatory sub	PIK3R4	153.1	45.576	4.5	6	7	6	1.078	0.924	1.116	0.952	1.098	0.949	1.09	0.968	1.03	1.02	1.03
Q9Y3A3	MOB-like protein phocein OS=Homo sap	MOB4	26.032	45.254	26.7	5	9	5	1.012	0.995	1.061	1.012	0.993	1.03	0.977	0.912	1.03	1.01	0.94
Q9Y639	Neuroplastin OS=Homo sapiens OX=96(	NPTN	44.387	38.502	11.1	4	5	4	1.012	0.995	1.03	1.043	0.943	0.916	1.005	0.967	1.03	0.93	0.98
Q6PJG2	ELM2 and SANT domain-containing pro	ELMSAN1	114.99	12.707	3.2	2	2	2	1.067	0.889	1.105	0.915	1.011	0.861	1.151	1.07	1.03	0.96	1.14
Q5F1R6	DnaJ homolog subfamily C member 21 O	DNAJC21	62.027	14.989	4.9	2	2	2	1.054	0.999	1.051	1.069	0.869	0.974	1.067	0.876	1.03	0.90	0.95
O14893	Gem-associated protein 2 OS=Homo sapi	GEMIN2	31.585	12.987	7.9	2	2	2	0.988	1.005	1.048	1.01	0.941	0.913	1.208	0.941	1.03	0.93	1.08
Q9H2W6	39S ribosomal protein L46, mitochondria	MRPL46	31.705	80.236	25.1	5	8	5	1.006	0.961	1.058	0.973	1.129	1.035	0.99	0.967	1.03	1.10	0.99
Q9NXK8	F-box/LRR-repeat protein 12 OS=Homo	FBXL12	37.026	10.995	4.9	1	1	1	0.947	0.934	0.929	1.013	1.091	1.173	0.935	1.069	1.03	1.20	1.07
P27695	DNA-(apurinic or apyrimidinic site) lyase	APEX1	35.554	209.44	48.7	12	18	12	0.991	1	1.052	1.003	0.973	1.046	1.087	0.958	1.03	1.01	1.03
Q99700	Ataxin-2 OS=Homo sapiens OX=9606 G	ATXN2	140.28	86.425	12	14	15	12	0.97	1.023	1.048	1.009	0.986	0.89	1.121	1.077	1.03	0.94	1.10
Q9NXF7	DDB1- and CUL4-associated factor 16 C	DCAF16	24.193	15.417	12.5	2	2	2	0.958	0.978	0.94	1.058	0.972	0.916	1.169	1.054	1.03	0.98	1.15
Q9H845	Acyl-CoA dehydrogenase family member	ACAD9	68.76	61.558	18.8	9	10	8	0.981	0.93	0.944	1.028	1.064	1.231	0.895	1.054	1.03	1.20	1.02
P20339	Ras-related protein Rab-5A OS=Homo sa	RAB5A	23.658	74.99	54	9	12	5	1.015	0.93	0.988	1.019	1.03	0.849	1.071	1.018	1.03	0.97	1.07
Q9H8E8	Cysteine-rich protein 2-binding protein C	KAT14	88.843	11.469	2.3	2	2	2	0.98	0.934	0.966	1.009	0.99	1.265	0.905	1.021	1.03	1.18	1.01
Q6IBS0	Twinfilin-2 OS=Homo sapiens OX=9606	TWF2	39.548	98.89	22.6	5	6	5	0.934	0.994	1.019	0.97	1.005	1.088	1.084	0.941	1.03	1.09	1.05
Q9H0L4	Cleavage stimulation factor subunit 2 tau	CSTF2T	64.436	47.829	28.6	11	13	7	1.023	1.075	1.133	1.031	0.85	1.119	0.76	0.933	1.03	0.94	0.81
O00425	Insulin-like growth factor 2 mRNA-bindi	IGF2BP3	63.704	274.47	45.3	25	52	22	0.958	0.984	0.997	1.006	0.982	0.961	1.136	1.041	1.03	1.00	1.12
Q7KZI7	Serine/threonine-protein kinase MARK2	MARK2	87.91	13.322	7	5	5	2	1.013	0.901	0.978	0.996	1.112	0.952	1.038	1.058	1.03	1.08	1.10
P25208	Nuclear transcription factor Y subunit be	NFYB	22.831	7.5447	6.3	1	3	1	0.999	0.958	1.009	1.009	1.096	0.901	1.073	1.002	1.03	1.02	1.06
Q9H0H5	Rac GTPase-activating protein 1 OS=Ho	RACGAP1	71.026	38.212	12.2	5	6	5	0.966	0.931	1.004	0.952	1.156	1.204	0.986	0.982	1.03	1.24	1.04
Q9BUH6	Protein PAXX OS=Homo sapiens OX=9	PAXX	21.639	29.118	21.1	3	6	3	1.032	1.026	1.016	1.106	1.03	0.931	0.926	1.082	1.03	0.95	0.98
Q6ZTN6	Ankyrin repeat domain-containing protei	ANKRD13	58.475	13.706	2.3	1	1	1	0.962	0.936	1.005	0.952	1.143	0.9	1.153	1.071	1.03	1.08	1.17
P12081	Histidine--tRNA ligase, cytoplasmic OS=	HARS	57.41	163.92	38.1	17	30	13	0.996	0.969	0.998	1.028	1.021	1.006	1.01	0.969	1.03	1.03	1.01
Q92997	Segment polarity protein dishevelled hon	DVL3	78.054	27.934	5.4	3	3	2	1.006	1.056	1.065	1.061	0.968	1.215	0.747	0.892	1.03	1.06	0.79
P27816	Microtubule-associated protein 4 OS=Hc	MAP4	121	301.16	32.9	33	52	33	0.988	0.946	0.993	1.001	0.983	0.926	1.117	1.071	1.03	0.99	1.13
Q16629	Serine/arginine-rich splicing factor 7 OS=	SRSF7	27.366	47.896	28.6	7	11	6	0.95	0.989	1.02	0.979	1.018	1.029	0.991	1.111	1.03	1.06	1.08
Q53H82	Endoribonuclease LACTB2 OS=Homo sa	LACTB2	32.805	48.282	23.3	7	8	7	0.984	0.957	0.972	1.029	1.08	1.212	0.793	1.007	1.03	1.18	0.93
Q8TF01	Arginine/serine-rich protein PNISR OS=	PNISR	92.576	20.992	3.9	3	4	3	1.041	1.006	1.065	1.045	0.919	0.972	1.012	0.93	1.03	0.92	0.95
P78371	T-complex protein 1 subunit beta OS=Hc	CCT2	57.488	240.34	53.3	23	44	22	0.974	1.05	1.086	1	0.949	1.145	0.895	0.952	1.03	1.03	0.91
P51665	26S proteasome non-ATPase regulatory s	PSMD7	37.025	64.702	27.5	6	7	6	1.064	0.838	0.935	1.025	1.068	0.78	1.103	1.106	1.03	0.97	1.16
Q8IZP0	Abl interactor 1 OS=Homo sapiens OX=9	ABI1	55.08	87.391	14	8	11	5	0.985	1.016	1.05	1.012	0.965	0.923	1.127	1.04	1.03	0.94	1.08
Q7Z4Q2	HEAT repeat-containing protein 3 OS=H	HEATR3	74.582	59.533	10.6	6	8	6	0.941	0.964	0.983	0.98	1.023	1.197	0.901	0.989	1.03	1.17	0.99
O60343	TBC1 domain family member 4 OS=Hon	TBC1D4	146.56	79.963	9.8	11	14	11	0.973	1.011	1.033	1.011	1.075	1.016	1.013	1.021	1.03	1.05	1.03
Q9NZJ5	Eukaryotic translation initiation factor 2-	EIF2AK3	125.21	11.981	2.2	2	2	2	0.969	0.982	0.997	1.013	1.112	1.311	0.76	0.927	1.03	1.24	0.86
Q13685	Angio-associated migratory cell protein C	AAMP	46.75	57.36	17.5	6	7	6	0.913	0.974	0.951	0.993	1.083	0.932	1.205	1.035	1.03	1.07	1.19
Q6DKK2	Tetratricopeptide repeat protein 19, mitoc	TTC19	42.456	14.441	5	2	3	2	0.994	0.96	1.02	0.993	1.141	1.073	0.929	0.954	1.03	1.13	0.96
Q15022	Polycomb protein SUZ12 OS=Homo sap	SUZ12	83.054	62.079	9.2	7	7	7	0.974	0.947	0.971	1.008	1.072	1.271	0.813	1.034	1.03	1.22	0.96

O96019	Actin-like protein 6A OS=Homo sapiens	ACTL6A	47.46	102.59	31.5	11	18	11	1.015	1.007	1.081	1.002	1.003	1.166	0.866	0.919	1.03	1.07	0.88
P19105	Myosin regulatory light chain 12A OS=F	MYL12A	19.794	77.786	44.4	6	16	1	0.986	1.038	1.035	1.05	0.889	0.891	0.966	1.05	1.03	0.88	1.00
Q96PZ0	Pseudouridylate synthase 7 homolog OS=	PUS7	75.035	86.768	18.3	10	12	10	0.966	0.927	0.957	0.993	0.996	1.023	1.093	1.004	1.03	1.07	1.11
Q7Z569	BRCA1-associated protein OS=Homo sa	BRAP	67.304	12.339	3.4	2	2	2	1.026	0.967	1.084	0.969	1.096	0.786	1.19	0.933	1.03	0.94	1.07
P53396	ATP-citrate synthase OS=Homo sapiens (	ACLY	120.84	323.31	37.1	33	64	32	0.998	0.968	1.014	1.011	1.049	1.2	0.874	0.973	1.03	1.14	0.94
Q92871	Phosphomannomutase 1 OS=Homo sapie	PMM1	29.746	27.552	19.1	4	4	4	0.996	0.974	1.011	1.018	1.095	0.938	1.071	0.907	1.03	1.03	1.00
Q8WU90	Zinc finger CCCH domain-containing pro	ZC3H15	48.602	69.427	20.7	8	13	8	1.038	0.969	1.029	1.038	0.948	1.003	0.912	0.997	1.03	0.97	0.95
Q8N3C0	Activating signal cointegrator 1 complex	ASCC3	251.46	170.79	11.3	22	27	22	0.991	0.984	1.029	1.005	1.039	1.106	0.959	0.967	1.03	1.09	0.98
Q9Y5Q9	General transcription factor 3C polypepti	GTF3C3	101.27	75.65	13.4	10	13	10	0.972	1.006	1.031	1.006	0.914	1.102	0.965	0.919	1.03	1.02	0.95
O95295	SNARE-associated protein Snapin OS=F	SNAPIN	14.874	19.851	26.5	3	3	3	1.02	0.96	0.987	1.052	1.08	0.939	0.965	0.997	1.03	1.02	0.99
O96013	Serine/threonine-protein kinase PAK 4 O	PAK4	64.071	34.449	8.5	5	6	5	1.097	0.75	0.826	1.076	1.085	0.725	0.818	1.572	1.03	0.98	1.29
Q9Y375	Complex I intermediate-associated protei	NDUFAF1	37.763	6.6567	2.8	1	1	1	0.975	0.981	0.955	1.059	1.02	1.254	0.782	1.013	1.03	1.16	0.92
P60981	Destrin OS=Homo sapiens OX=9606 GN	DSTN	18.506	45	23	7	8	7	1.073	0.966	1.072	1.027	0.986	0.851	1.043	0.964	1.03	0.90	0.98
P43034	Platelet-activating factor acetylhydrolase	PAFAH1B	46.637	123.61	38.3	13	22	13	1.009	1	1.056	1.012	0.948	0.942	1.06	0.949	1.03	0.94	1.00
Q5T0W9	Protein FAM83B OS=Homo sapiens OX	FAM83B	114.8	60.744	9.4	9	10	9	0.989	0.952	0.971	1.027	0.944	0.839	1.215	1.082	1.03	0.92	1.18
Q96BH1	E3 ubiquitin-protein ligase RNF25 OS=F	RNF25	51.218	32.746	11.1	4	5	4	1.028	0.914	0.971	1.028	0.994	0.979	1.033	1.014	1.03	1.02	1.05
Q13191	E3 ubiquitin-protein ligase CBL-B OS=F	CBLB	109.45	14.341	4.9	3	4	2	0.906	1.038	1.075	0.926	1.241	0.72	1.223	1.05	1.03	1.01	1.17
P09234	U1 small nuclear ribonucleoprotein C OS	SNRPC	17.394	27.625	30.2	4	5	4	1.052	0.933	1.027	1.016	1.042	1.058	0.936	0.979	1.03	1.06	0.96
Q9H5K3	Protein O-mannose kinase OS=Homo sap	POMK	40.05	15.277	7.7	2	2	2	1.173	0.579	0.955	0.848	1.736	1.168	0.691	0.866	1.03	1.66	0.89
Q12933	TNF receptor-associated factor 2 OS=Ho	TRAF2	55.859	64.394	17	8	9	8	1.027	1	1.05	1.036	0.989	1.04	0.952	1.015	1.03	1.00	0.97
Q9H082	Ras-related protein Rab-33B OS=Homo s	RAB33B	25.717	9.6834	6.6	1	1	1	1.028	0.969	1.004	1.051	1.061	1.005	0.944	0.932	1.03	1.03	0.94
O14681	Etoposide-induced protein 2.4 homolog (	EI24	38.964	7.5595	3.2	1	1	1	0.95	1.049	1.021	1.036	0.933	0.956	1.16	0.961	1.03	0.94	1.06
Q9NUQ8	ATP-binding cassette sub-family F memb	ABCF3	79.744	69.236	17.5	9	8	8	1.007	0.97	1.021	1.013	1.046	1.027	1.016	0.982	1.03	1.05	1.01
Q99567	Nuclear pore complex protein Nup88 OS	NUP88	83.541	121.78	14.4	8	12	8	0.98	0.964	1.03	0.97	1.075	1.205	0.876	1.015	1.03	1.17	0.97
Q96KB5	Lymphokine-activated killer T-cell-origin	PBK	36.085	19.016	8.4	2	3	2	0.924	0.885	0.869	0.992	1.217	1.145	0.884	1.215	1.03	1.31	1.16
P17544	Cyclic AMP-dependent transcription fact	ATF7	52.967	20.274	7.7	3	4	3	1.001	1.019	1.017	1.061	0.929	0.867	1.098	1.019	1.03	0.89	1.05
O14976	Cyclin-G-associated kinase OS=Homo sa	GAK	143.19	91.08	12.1	13	17	13	0.995	1.028	1.047	1.034	0.947	0.975	1.047	0.965	1.03	0.95	0.99
Q96P70	Importin-9 OS=Homo sapiens OX=9606	IPO9	115.96	242.42	25.4	23	42	22	0.976	0.978	1.001	1.009	1.009	0.996	1.04	1.007	1.03	1.03	1.05
P49189	4-trimethylaminobutyraldehyde dehydrog	ALDH9A1	53.801	100.32	31.2	12	17	12	0.969	1.056	1.021	1.062	0.953	0.933	1.026	0.997	1.03	0.93	1.00
Q8WWC4	m-AAA protease-interacting protein 1, mi	MAIP1	32.544	27.648	14.1	4	5	4	0.975	0.948	0.956	1.022	1.116	0.985	1.114	1.049	1.03	1.09	1.12
Q5GLZ8	Probable E3 ubiquitin-protein ligase HEF	HERC4	118.56	11.369	2.6	2	2	2	0.952	1.078	0.991	1.097	0.976	1.08	0.853	0.999	1.03	1.01	0.91
O95551	Tyrosyl-DNA phosphodiesterase 2 OS=F	TDP2	40.929	25.419	12.4	3	3	3	1.026	0.869	0.924	1.025	1.082	1.115	0.849	1.134	1.03	1.16	1.05
P08579	U2 small nuclear ribonucleoprotein B'' O:	SNRPB2	25.486	40.122	28	6	12	5	1.033	0.901	0.988	1.001	1.084	0.965	1.103	1.061	1.03	1.06	1.12
Q93050	V-type proton ATPase 116 kDa subunit a	ATP6V0A1	96.412	77.794	12.9	8	10	8	0.954	0.983	0.968	1.024	1.002	1.031	0.973	1.074	1.03	1.05	1.06
O15498	Synaptobrevin homolog YKT6 OS=Homo	YKT6	22.417	59.945	38.9	7	9	7	1.012	0.962	1.023	1.007	1.058	1.219	0.832	0.909	1.03	1.15	0.88
P62834	Ras-related protein Rap-1A OS=Homo sa	RAP1A	20.987	33.537	38	7	12	2	0.983	1.062	1.043	1.06	0.942	1.045	1.012	0.954	1.03	0.97	0.96
P09651	Heterogeneous nuclear ribonucleoprotein	HNRNPA1	38.746	287.96	48.1	17	58	17	0.986	1.027	1.061	1.009	1.014	1.148	0.905	0.902	1.03	1.07	0.90
Q96F63	Coiled-coil domain-containing protein 97	CCDC97	38.946	25.578	16.9	4	4	4	1.015	0.917	1.002	0.984	1.101	1.08	0.839	0.995	1.03	1.13	0.95
Q9BTY2	Plasma alpha-L-fucosidase OS=Homo sap	FUCA2	54.066	12.683	4.1	2	2	2	0.997	0.938	0.953	1.036	0.975	0.964	1.118	1.053	1.03	1.00	1.12
Q6P1N0	Coiled-coil and C2 domain-containing pr	CC2D1A	104.06	82.858	15	12	14	12	0.965	0.975	1.015	0.979	0.977	0.994	1.141	0.999	1.03	1.02	1.10
Q9Y3Y2	Chromatin target of PRMT1 protein OS=	CHTOP	26.396	27.576	20.2	4	8	4	0.914	0.853	0.787	1.029	1.104	0.849	1.12	1.238	1.03	1.11	1.33
P08238	Heat shock protein HSP 90-beta OS=Hor	HSP90AB1	83.263	323.31	48.2	35	132	19	0.986	0.998	1.028	1.011	0.978	0.968	1.064	0.983	1.03	0.98	1.03
Q9Y2I1	Nischarin OS=Homo sapiens OX=9606 (	NISCH	166.63	11.938	1.3	2	2	2	1.048	1.086	1.129	1.064	0.928	1.15	0.689	0.883	1.03	0.97	0.74
Q8TBF4	Zinc finger CCHC-type and RNA-bindin	ZCRB1	24.591	12.304	11.1	2	2	2	1.025	1.009	1.083	1.007	1.036	1.276	0.654	0.949	1.03	1.14	0.79
Q07021	Complement component 1 Q subcompone	C1QBP	31.362	88.124	31.6	6	15	6	0.944	0.983	0.993	0.987	1.049	1.139	0.98	1.02	1.03	1.14	1.04
Q9BQE9	B-cell CLL/lymphoma 7 protein family m	BCL7B	22.195	7.765	12.4	2	2	1	0.899	1.174	1.125	1.005	1.03	0.957	1.002	0.94	1.03	0.96	0.94
P46939	Utrophin OS=Homo sapiens OX=9606 G	UTRN	394.46	133.01	5.7	17	18	17	0.973	0.955	0.977	1.004	1.015	0.971	1.103	1.012	1.03	1.03	1.10
Q8NHU6	Tudor domain-containing protein 7 OS=F	TDRD7	123.58	10.293	1.1	1	1	1	1.019	1.094	1.094	1.077	0.831	0.883	0.985	1.007	1.03	0.81	0.94
P28072	Proteasome subunit beta type-6 OS=Hom	PSMB6	25.357	46.496	29.7	6	10	6	0.994	0.937	0.991	0.993	1.08	0.958	1.019	1.029	1.03	1.06	1.06
Q6UW68	Transmembrane protein 205 OS=Homo s	TMEM205	21.198	20.3	20.6	3	3	3	1.003	0.975	0.936	1.096	0.939	0.868	1.176	0.985	1.03	0.91	1.09
Q9UHY7	Enolase-phosphatase E1 OS=Homo sapie	ENOPH1	28.932	96.637	24.5	4	7	4	1.006	1.054	1.084	1.032	0.943	0.964	1.041	0.944	1.03	0.93	0.96
P49419	Alpha-aminoadipic semialdehyde dehydro	ALDH7A1	58.486	285.17	43.8	19	39	19	0.971	0.982	0.993	1.013	1.023	0.998	1.019	1.015	1.03	1.03	1.04
Q15370	Elongin-B OS=Homo sapiens OX=9606	ELOB	13.133	57.598	28.8	4	6	4	1.02	1.119	1.159	1.038	0.887	0.854	1.108	0.929	1.03	0.81	0.95
Q969V3	Nicalin OS=Homo sapiens OX=9606 GN	NCLN	62.974	64.752	16.5	7	10	7	1.012	1.101	1.153	1.017	0.962	0.893	1.087	0.889	1.03	0.88	0.94
O75688	Protein phosphatase 1B OS=Homo sapie	PPM1B	52.642	104.73	28.4	10	10	7	0.938	1.032	1.037	0.986	1.077	0.989	1.016	0.968	1.03	1.05	1.01
Q9H910	Jupiter microtubule associated homolog 2	JPT2	20.063	73.585	52.1	8	13	8	0.986	0.947	1.007	0.978	1.054	0.923	1.156	1.108	1.03	1.02	1.17
Q05D32	CTD small phosphatase-like protein 2 OS	CTDSPL2	52.998	69.25	21.5	9	9	8	0.968	0.929	0.951	0.997	1.203	1.23	0.921	0.996	1.03	1.28	1.01
Q9UEU0	Vesicle transport through interaction witl	VTI1B	26.688	32.432	15.1	3	3	3	1.03	0.951	1.025	1.009	1.076	0.97	0.977	0.988	1.03	1.03	0.99
Q9NUU7	ATP-dependent RNA helicase DDX19A	DDX19A	53.974	114.5	33.5	11	18	11	1.005	0.986	1.01	1.034	1.024	0.982	0.963	0.961	1.03	1.01	0.97
O15226	NF-kappa-B-repressing factor OS=Homo	NKRF	77.672	83.026	18.1	11	12	11	0.958	0.923	0.909	1.022	1.082	1.046	0.998	1.073	1.03	1.13	1.10
Q99832	T-complex protein 1 subunit eta OS=Hor	CCT7	59.366	181.08	41.3	17	33	17	1.036	0.962	1.048	1.003	1.05	1.025	1.004	0.944	1.03	1.04	0.97
Q8IWA0	WD repeat-containing protein 75 OS=Ho	WDR75	94.498	109.26	20.2	13	19	13	0.967	1.001	0.986	1.034	0.978	1.052	0.942	0.921	1.03	1.03	0.95
O95372	Acyl-protein thioesterase 2 OS=Homo sa	LYPLA2	24.737	82.666	34.2	7	13	7	1.014	0.994	1.035	1.026	1.037	0.927	1.007	1	1.03	0.98	1.00
O75368	SH3 domain-binding glutamic acid-rich-l	SH3BGRL	12.774	50.664	56.1	6	7	6	1.018	1.046	1.094	1.024	0.93	0.934	1.131	0.907	1.03	0.90	0.99
P42858	Huntingtin OS=Homo sapiens OX=9606	HTT	347.6	61.083	3.4	9	11	9	1.028	0.964	1.068	0.976	0.982	0.963	1.073	0.956	1.03	0.98	1.02
Q3ZCQ8	Mitochondrial import inner membrane tra	TIMM50	39.646	72.565	29.2	7	11	7	0.989	0.927	0.958	1.008	1.083	1.091	0.909	1.01	1.03	1.13	1.00
P25789	Proteasome subunit alpha type-4 OS=Ho	PSMA4	29.483	101.33	40.6	9	14	9	1.01	1.024	1.064	1.023	0.979	1.008	0.967	0.947	1.03	0.98	0.94
Q4J6C6	Prolyl endopeptidase-like OS=Homo sap	PREPL	83.926	20.078	5.1	3	3	3	1.043	0.957	1.071	0.981	1.024	1.01	1	0.952	1.03	1.02	0.98
Q16537	Serine/threonine-protein phosphatase 2A	PPP2R5E	54.699	43.268	9.9	4	6	4	0.908	1.133	1.044	1.05	0.784	0.995	1.065	1.011	1.03	0.87	1.02
Q14980	Nuclear mitotic apparatus protein 1 OS=I	NUMA1	238.26	323.31	27.2	47	62	47	0.994	0.973	1.039	0.979	1.051	1.004	1.068	1.001	1.03	1.04	1.05
Q96DG6	Carboxymethylenebutenolidase homolog	CMBL	28.048	34.456	20.4	5	7	5	0.988	0.941	0.937	1.042	1.073	1.148	0.862	1.068	1.03	1.15	1.00
Q07157	Tight junction protein ZO-1 OS=Homo s	TJP1	195.46	252.1	19.2	24	31	24	1.008	1.039	1.064	1.036	0.944	0.939	1.023	0.958	1.03	0.92	0.97
Q14155	Rho guanine nucleotide exchange factor '	ARHGEF7	90.011	74.468	17.3	10	10	10	0.986	0.947	0.929	1.054	0.997	0.963	1.053	1.097	1.03	1.01	1.11
Q9UGU0	Transcription factor 20 OS=Homo sapien	TCF20	211.77	12.083	0.9	2	2	2	0.985	1.067	1.098	1.007	0.96	1.305	0.747	0.901	1.03	1.10	0.80
O60281	Zinc finger protein 292 OS=Homo sapier	ZNF292	304.81	11.25	0.8	2	2	2	0.968	1.011	1.041	0.989	0.989	1.028	0.972	1.099	1.03	1.02	1.05
Q8N2F6	Armadillo repeat-containing protein 10 C	ARMC10	37.54	16.279	8.2	2	2	2	1.042	1.019	1.151	0.963	0.969	0.962	1.043	0.898	1.03	0.94	0.94
Q15758	Neutral amino acid transporter B(0) OS=	SLC1A5	56.598	36.366	9.8	4	5	4	0.92	0.989	1.015	0.943	1.107	1.13	0.986	1.076	1.03	1.17	1.08
Q9Y2L5	Trafficking protein particle complex subu	TRAPPC8	161	29.146	3.7	4	4	4	0.98	0.971	0.964	1.037	0.944	0.885	1.198	1.065	1.03	0.94	1.16
O60678	Protein arginine N-methyltransferase 3 O	PRMT3	59.902	65.274	21.3	9	9	9	1.012	1.022	1.083	1.003	0.95	1.072	0.987	0.811	1.03	0.99	0.88
Q8N9M1	Uncharacterized protein C19orf47 OS=H	C19orf47	44.746	20.078	8.3	3	3	3	0.966	0.846	0.87	0.988	1.155	0.881	1.218	1.164	1.03	1.12	1.31
P55884	Eukaryotic translation initiation factor 3 :	EIF3B	92.48	191.51	28.7	20	29	18	0.982	0.993	1.001	1.024	0.987	0.99	1.05	1.029	1.03	1.00	1.05
Q13618	Cullin-3 OS=Homo sapiens OX=9606 G	CUL3	88.929	165.99	22.5	15	20	15	1.016	0.976	1.035	1.007	1.061	0.969	1.033	0.927	1.03	1.02	0.98
Q96IZ6	Methyltransferase-like protein 2A OS=H	METTL2A	43.537	7.3794	4	1	1	1	0.927	0.807	0.699	1.078	1.226	0.765	1.236	1.32	1.02	1.15	1.47
Q86TB9	Protein PAT1 homolog 1 OS=Homo sapi	PATL1	86.849	20.779	6.8	4	3	3	0.983	0.956	0.999	0.988	1.002	0.964	1.18	1.002	1.02	1.01	1.13
Q9Y6A5	Transforming acidic coiled-coil-containin	TACC3	90.359	112.86	16.1	9	9	9	1.007	0.812	0.953	0.911	1.121	1.024	1.161	1.09	1.02	1.18	1.24
Q8IWV8	E3 ubiquitin-protein ligase UBR2 OS=H	UBR2	200.54	46.753	3.2	5	5	5	1.018	0.967	1.043	0.991	1.052	1.073	1.004	0.897	1.02	1.07	0.96
P50579	Methionine aminopeptidase 2 OS=Homo	METAP2	52.891	50.634	21.3	6	9	5	1.023	0.925	1.072	0.924	1.093	1.027	1.041	1.118	1.02	1.09	1.11
P40123	Adenylyl cyclase-associated protein 2 OS	CAP2	52.823	75.608	18.7	7	9	7	0.948	1.043	1.033	1.007	1.023	1.282	0.815	0.985	1.02	1.16	0.90
Q9Y263	Phospholipase A-2-activating protein OS	PLAA	87.156	187.61	33.2	19	23	19	1.016	1.023	1.052	1.037	0.999	1.041	0.974	0.992	1.02	1.00	0.96
P39748	Flap endonuclease 1 OS=Homo sapiens (	FEN1	42.592	130	32.6	9	12	8	0.916	0.88	0.872	0.968	1.113	1.059	1.164	1.221	1.02	1.21	1.33
Q99717	Mothers against decapentaplegic homolo;	SMAD5	52.258	19.248	7.3	3	3	2	0.985	1.141	1.078	1.1	0.823	1.1	0.926	0.841	1.02	0.90	0.83
P26196	Probable ATP-dependent RNA helicase I	DDX6	54.416	139.42	33.1	12	16	12	1.01	0.994	1.026	1.027	1.001	1.128	0.829	0.979	1.02	1.06	0.90
Q9NPD3	Exosome complex component RRP41 OS	EXOSC4	26.383	19.519	13.5	3	4	3	0.958	0.967	1.027	0.945	0.984	1.295	0.728	1.086	1.02	1.18	0.94
Q96JB5	CDK5 regulatory subunit-associated prot	CDK5RAP	56.92	63.749	14.8	8	11	8	0.988	0.989	1.041	0.984	1.06	0.975	0.936	1.046	1.02	1.03	1.00
Q8IX03	Protein KIBRA OS=Homo sapiens OX=9	WWC1	125.3	50.731	7.5	7	7	6	0.99	0.984	0.987	1.034	1.035	0.912	1.06	1.096	1.02	0.99	1.09
Q6ZP29	Lysosomal amino acid transporter 1 homo	PQLC2	31.947	9.6303	4.5	1	1	1	0.931	1.015	0.972	1.02	0.962	0.916	1.096	1.192	1.02	0.97	1.18
P50991	T-complex protein 1 subunit delta OS=H	CCT4	57.924	239.71	37.5	18	29	18	1.023	1.01	1.068	1.013	0.969	0.958	1.041	0.981	1.02	0.95	0.99
Q15046	Lysine--tRNA ligase OS=Homo sapiens (	KARS	68.047	185.8	33	20	38	20	0.994	1.042	1.062	1.022	0.954	1.085	0.915	0.907	1.02	1.00	0.89
Q96ER9	Coiled-coil domain-containing protein 51	CCDC51	45.811	48.443	15.3	5	5	5	0.967	1.031	1.049	0.996	0.959	0.99	1.174	0.872	1.02	0.98	1.02
Q9H583	HEAT repeat-containing protein 1 OS=H	HEATR1	242.37	202.3	14.8	28	32	28	0.974	0.991	0.999	1.012	1.05	1.141	0.947	0.991	1.02	1.12	0.99
P15289	Arylsulfatase A OS=Homo sapiens OX=9	ARSA	53.588	23.232	9.9	3	3	3	1	0.966	1.022	0.99	0.941	0.96	1.14	1.046	1.02	0.97	1.11
Q9Y2Z0	Protein SGT1 homolog OS=Homo sapier	SUGT1	41.024	190.33	57	20	33	20	0.989	0.986	0.996	1.025	1.043	0.965	1.015	0.997	1.02	1.02	1.02
Q96BJ3	Axin interactor, dorsalization-associated	AIDA	35.023	29.843	14.7	4	5	4	1.039	0.937	0.974	1.048	1.11	0.902	1.068	1.023	1.02	1.02	1.06
Q53T59	HCLS1-binding protein 3 OS=Homo sap	HS1BP3	42.78	59.174	26.3	7	9	7	0.975	1.003	1.029	0.995	0.988	0.928	1.106	1.096	1.02	0.97	1.11
Q9UBP0	Spastin OS=Homo sapiens OX=9606 GN	SPAST	67.196	42.832	9.1	4	4	4	1.002	0.978	1.048	0.978	1.271	0.912	1.127	0.853	1.02	1.10	1.00
Q8N7H5	RNA polymerase II-associated factor 1 ho	PAF1	59.975	61.722	17.1	8	12	8	0.972	1.013	0.97	1.061	0.972	1.002	0.969	1.049	1.02	0.99	1.02
Q14669	E3 ubiquitin-protein ligase TRIP12 OS=I	TRIP12	220.43	75.963	6.6	10	13	10	1.075	1.045	1.13	1.039	0.961	1.12	0.928	0.832	1.02	0.98	0.83
Q9Y223	Bifunctional UDP-N-acetylglucosamine 2	GNE	79.274	85.597	17.7	11	15	11	1.004	0.997	1.049	0.998	1.101	1.017	1.045	0.958	1.02	1.06	1.00
Q9BXW7	Haloacid dehalogenase-like hydrolase do	HDHD5	46.321	63.26	24.6	7	9	6	1.03	0.902	1.051	0.925	1.052	1.042	1.02	0.785	1.02	1.08	0.93
Q96EV2	RNA-binding protein 33 OS=Homo sapie	RBM33	129.98	26.792	4.3	4	4	4	0.957	1.024	1.056	0.97	1.018	0.981	1.17	0.931	1.02	1.01	1.06
P27708	CAD protein OS=Homo sapiens OX=960	CAD	242.98	323.31	19.5	31	54	31	0.988	0.956	0.98	1.008	1.033	1.038	0.99	1.001	1.02	1.07	1.02
P46926	Glucosamine-6-phosphate isomerase 1 O:	GNPDA1	32.668	141.37	46.7	10	16	9	1.016	0.974	1.029	1.006	1.027	0.877	0.983	1.021	1.02	0.96	1.01
Q92934	Bcl2-associated agonist of cell death OS=	BAD	18.392	14.66	14.9	2	4	2	0.963	0.9	0.954	0.951	1.159	0.913	1.186	1.141	1.02	1.11	1.25
P52735	Guanine nucleotide exchange factor VAV	VAV2	101.29	191.5	21	17	21	17	1.001	1.046	1.035	1.058	0.921	1.151	0.884	0.911	1.02	1.01	0.88
Q5VYK3	Proteasome adapter and scaffold protein I	ECPAS	204.29	293.93	22.9	33	48	33	0.969	0.989	1.005	0.997	1.024	1.022	1.059	1.037	1.02	1.04	1.07
P49720	Proteasome subunit beta type-3 OS=Hom	PSMB3	22.949	33.393	22	3	4	3	0.948	1.065	1.007	1.051	0.922	1.222	0.818	0.857	1.02	1.07	0.83
Q6YP21	Kynurenine--oxoglutarate transaminase 3	KYAT3	51.4	97.448	23.3	8	9	8	0.966	0.958	0.957	1.01	0.968	1.224	0.787	1.006	1.02	1.14	0.93
P41240	Tyrosine-protein kinase CSK OS=Homo	CSK	50.704	12.509	5.3	2	3	2	0.96	1.016	1.029	0.991	1.074	1.07	0.985	0.971	1.02	1.09	0.99
Q9Y3C6	Peptidyl-prolyl cis-trans isomerase-like 1	PPIL1	18.237	57.457	33.1	5	9	5	1.015	1.007	1.019	1.048	0.952	1.097	0.906	1.033	1.02	1.01	0.96
Q01518	Adenylyl cyclase-associated protein 1 OS	CAP1	51.901	171.54	36.2	14	30	14	1.021	1.004	1.029	1.041	0.966	0.921	1.104	0.968	1.02	0.93	1.02
Q9BXV9	EKC/KEOPS complex subunit GON7 OS	GON7	10.859	53.78	41	2	4	2	0.99	1.007	1.046	0.995	0.958	1.234	0.885	1.041	1.02	1.10	0.96
P55036	26S proteasome non-ATPase regulatory s	PSMD4	40.736	121.24	31.8	8	11	5	1.016	0.937	1.007	0.989	1.047	1.054	0.95	1.05	1.02	1.08	1.02
Q9NY33	Dipeptidyl peptidase 3 OS=Homo sapien	DPP3	82.588	72.661	17.4	9	11	9	0.986	1.014	1.003	1.041	0.979	0.965	1.004	0.997	1.02	0.97	1.00
P08754	Guanine nucleotide-binding protein G(k)	GNAI3	40.532	25.972	24	7	12	3	1.004	0.952	1.012	0.987	0.901	1.042	1.117	1.098	1.02	0.99	1.13
Q9HC38	Glyoxalase domain-containing protein 4 (	GLOD4	34.793	101.26	42.5	11	20	11	0.991	1.012	1.036	1.011	0.943	0.952	0.999	1.004	1.02	0.95	1.00
P46736	Lys-63-specific deubiquitinase BRCC36	BRCC3	36.072	22.164	9.5	2	2	2	0.996	0.967	0.992	1.014	0.985	1.06	1.001	1.038	1.02	1.04	1.04
Q86YL5	Testis development-related protein OS=F	TDRP	20.402	66.354	27.6	4	5	4	0.988	1.024	1.023	1.033	0.94	1.138	0.946	0.952	1.02	1.03	0.94
P68133	Actin, alpha skeletal muscle OS=Homo s	ACTA1	42.051	13.207	29.7	11	109	1	0.958	1.012	0.989	1.024	0.919	0.903	1.139	1.09	1.02	0.92	1.13
O60684	Importin subunit alpha-7 OS=Homo sapie	KPNA6	60.029	118.83	25.6	11	13	7	0.98	0.945	0.964	1.003	1.105	0.863	1.151	1.015	1.02	1.02	1.13
Q6VY07	Phosphofurin acidic cluster sorting protei	PACS1	104.9	13.419	2.6	2	2	2	0.963	1.049	0.984	1.071	0.894	0.744	1.268	1.059	1.02	0.81	1.16
Q6UN15	Pre-mRNA 3'-end-processing factor FIP1	FIP1L1	66.526	58.341	13.6	6	7	6	0.991	1.022	1.075	0.981	1.022	1.067	0.926	0.967	1.02	1.04	0.94
O76094	Signal recognition particle subunit SRP7	SRP72	74.605	170.53	32.6	17	34	17	1	1.017	1.048	1.012	0.97	0.971	0.987	0.967	1.02	0.96	0.97
Q9HAS0	Protein Njmu-R1 OS=Homo sapiens OX	C17orf75	44.621	16.55	9.3	3	2	2	0.979	1.086	1.105	1.004	0.963	0.934	0.991	1.012	1.02	0.92	0.97
Q9NUQ7	Ufm1-specific protease 2 OS=Homo sapi	UFSP2	53.261	22.083	9.8	3	3	3	1.026	1.042	1.052	1.06	0.989	1.225	0.725	0.874	1.02	1.07	0.77
Q9ULC4	Malignant T-cell-amplified sequence 1 O	MCTS1	20.555	67.818	37.6	5	9	5	0.997	1.039	1.04	1.039	0.981	1.123	0.866	0.92	1.02	1.03	0.88
O43426	Synaptojanin-1 OS=Homo sapiens OX=9	SYNJ1	173.1	83.662	9.8	12	13	12	1.002	0.899	0.885	1.056	1.08	1.077	0.866	1.157	1.02	1.13	1.06
Q8WUA4	General transcription factor 3C polypepti	GTF3C2	100.68	42.93	7	5	7	5	0.977	1.021	1.014	1.026	1.023	1.169	0.887	0.938	1.02	1.10	0.91
Q9BRX2	Protein pelota homolog OS=Homo sapier	PELO	43.359	63.375	24.4	9	14	9	1.02	0.981	1.012	1.031	0.938	1.038	0.95	0.986	1.02	0.99	0.97
Q9NPI1	Bromodomain-containing protein 7 OS=I	BRD7	74.138	47.111	12.7	6	6	6	0.983	0.975	0.98	1.019	1.009	1.186	0.911	0.945	1.02	1.12	0.95
Q9BTE7	DCN1-like protein 5 OS=Homo sapiens (	DCUN1D5	27.508	27.601	17.7	4	6	4	1.009	1.102	1.083	1.072	0.981	1.21	0.735	0.802	1.02	1.04	0.73
Q9NR28	Diablo homolog, mitochondrial OS=Hom	DIABLO	27.131	34.2	24.7	5	7	5	0.981	1.013	1.046	0.989	1.03	1.058	0.986	0.971	1.02	1.05	0.98
O75439	Mitochondrial-processing peptidase subu	PMPCB	54.366	108.3	19.2	8	13	8	1.031	0.967	1.054	0.985	1.071	1.142	0.87	0.929	1.02	1.11	0.90
Q5SYE7	NHS-like protein 1 OS=Homo sapiens O	NHSL1	170.67	8.0541	0.9	1	1	1	1.019	0.934	0.936	1.057	1.044	1.005	1.016	0.988	1.02	1.05	1.03
Q9P2J5	Leucine--tRNA ligase, cytoplasmic OS=I	LARS	134.46	317.74	27.6	29	44	28	0.987	0.968	0.985	1.01	0.997	0.972	1.059	1.024	1.02	1.01	1.07
P11766	Alcohol dehydrogenase class-3 OS=Hom	ADH5	39.724	119.46	36.4	12	22	12	1.013	1.09	1.103	1.043	0.835	1.134	0.868	0.949	1.02	0.94	0.86
O75388	Probable G-protein coupled receptor 32 (	GPR32	40.087	17.805	3.7	1	2	1	0.988	0.929	0.93	1.026	1.046	0.98	1.084	1.064	1.02	1.06	1.12
P61978	Heterogeneous nuclear ribonucleoprotein	HNRNPK	50.976	119.47	31.5	14	26	12	0.978	0.992	1.029	0.981	1.019	0.958	1.07	1.02	1.02	1.00	1.06
Q9Y6M5	Zinc transporter 1 OS=Homo sapiens OX	SLC30A1	55.299	36.947	13.4	5	6	5	1.058	0.962	1.083	0.978	1.034	1.035	0.942	0.936	1.02	1.02	0.93
Q13619	Cullin-4A OS=Homo sapiens OX=9606 (	CUL4A	87.679	33.928	14.5	11	15	4	0.979	1.004	1.034	0.989	1.019	0.929	1.063	1.032	1.02	0.98	1.06
Q13330	Metastasis-associated protein MTA1 OS=	MTA1	80.785	65.907	16.5	9	10	5	1.05	1.043	1.059	1.076	0.981	1.171	0.865	0.937	1.02	1.03	0.86
Q15155	Nodal modulator 1 OS=Homo sapiens O:	NOMO1	134.32	119.34	17.6	16	21	1	1.029	1.022	1.036	1.056	0.95	1.007	0.916	0.979	1.02	0.95	0.92
Q6KC79	Nipped-B-like protein OS=Homo sapiens	NIPBL	316.05	119.04	6.9	17	21	17	1.035	0.92	0.974	1.02	1.069	1.171	0.851	0.991	1.02	1.15	0.94
Q99622	Protein C10 OS=Homo sapiens OX=960	C12orf57	13.178	21.625	29.4	3	4	3	1.033	1.023	1.081	1.016	1.012	1.108	0.846	0.847	1.02	1.03	0.82
P27694	Replication protein A 70 kDa DNA-bind	RPA1	68.137	214.62	35.4	18	29	18	0.983	0.931	0.951	1.001	1.065	1.089	1.001	1.047	1.02	1.13	1.07
Q9HBM6	Transcription initiation factor TFIID subu	TAF9B	27.622	24.137	15.1	4	4	4	1.036	0.934	0.997	1.012	0.916	1.139	1.022	0.965	1.02	1.04	1.01
O15381	Nuclear valosin-containing protein-like C	NVL	95.05	64.355	11.8	9	10	9	1.028	0.995	1.036	1.027	1.08	0.98	0.932	0.963	1.02	1.02	0.94

Q15813	Tubulin-specific chaperone E OS=Homo	TBCE	59.345	26.661	8	4	4	4	1.006	1.02	1.067	0.999	1.008	0.913	1.064	0.974	1.02	0.95	1.01
Q96K17	Transcription factor BTF3 homolog 4 OS	BTF3L4	17.27	45.953	36.7	4	6	4	0.917	1.07	0.907	1.119	0.927	1.144	0.821	1.061	1.02	1.04	0.95
O14617	AP-3 complex subunit delta-1 OS=Homo	AP3D1	130.16	145.21	18.7	16	24	16	1.009	1.032	1.018	1.063	0.946	0.955	0.966	0.916	1.02	0.93	0.92
O75925	E3 SUMO-protein ligase PIAS1 OS=Hor	PIAS1	71.835	15.792	3.8	2	2	2	0.994	1.057	1.118	0.973	1.116	0.915	1.046	0.855	1.02	0.99	0.93
P07737	Profilin-1 OS=Homo sapiens OX=9606 (	PFN1	15.054	95.866	70	10	41	9	1.022	1.029	1.059	1.032	0.936	0.935	1.034	0.946	1.02	0.91	0.97
Q96CW5	Gamma-tubulin complex component 3 O	TUBGCP3	103.57	85.963	10.7	8	8	8	1.002	1.013	1.043	1.011	0.997	1.082	0.892	1.012	1.02	1.03	0.94
Q9UHB7	AF4/FMR2 family member 4 OS=Homo	AFF4	127.46	33.088	4.8	5	6	5	0.943	1.024	1.001	1.004	1.075	1.098	0.909	0.903	1.02	1.10	0.92
Q9H832	Ubiquitin-conjugating enzyme E2 Z OS=	UBE2Z	38.21	39.577	17.8	5	6	5	0.976	1.097	1.121	0.992	0.917	1.17	0.99	0.926	1.02	1.01	0.92
Q96AT9	Ribulose-phosphate 3-epimerase OS=Ho	RPE	24.927	67.264	15.8	3	6	3	1.028	1.01	1.046	1.031	0.952	1.109	0.881	0.996	1.02	1.01	0.92
Q8N0W3	L-fucose kinase OS=Homo sapiens OX=	FUK	117.62	13.582	2.5	2	2	2	0.835	1.152	1.04	0.985	1.067	1.415	0.668	1.05	1.02	1.25	0.86
Q09327	Beta-1,4-mannosyl-glycoprotein 4-beta-N	MGAT3	61.312	62.653	8.3	3	4	3	0.968	0.77	0.809	0.962	1.173	0.985	1.212	1.232	1.02	1.24	1.41
Q8NC44	Reticulophagy regulator 2 OS=Homo sap	RETREG2	57.829	13.968	6.3	2	3	2	1.01	0.943	0.94	1.05	0.936	0.987	1.076	1.073	1.02	0.98	1.10
Q99594	Transcriptional enhancer factor TEF-5 O	TEAD3	48.676	37.132	12.2	5	6	2	1.119	0.994	1.116	1.037	0.933	0.894	1.039	0.795	1.02	0.86	0.87
Q9BRZ2	E3 ubiquitin-protein ligase TRIM56 OS=	TRIM56	81.487	23.569	5.6	3	3	3	0.865	1.206	1.115	0.995	0.942	1.015	1.073	0.959	1.02	0.94	0.98
P50895	Basal cell adhesion molecule OS=Homo	BCAM	67.404	99.287	22	9	10	9	0.95	1.015	1.012	0.99	1.033	1.394	0.834	0.915	1.02	1.24	0.89
Q2TAA5	GDP-Man:Man(3)GlcNAc(2)-PP-Dol alp	ALG11	55.65	11.966	4.7	2	2	2	1.021	0.945	0.937	1.066	1.053	0.968	0.999	1.001	1.02	1.03	1.02
P35269	General transcription factor IIF subunit 1	GTF2F1	58.24	62.868	17.2	8	9	8	1.015	1.015	1.087	0.981	1.043	1.035	1.031	0.949	1.02	1.02	0.98
Q9Y6I4	Ubiquitin carboxyl-terminal hydrolase 3 (	USP3	58.896	12.565	5.2	2	2	2	1.008	1.094	1.222	0.919	0.882	0.943	1.032	1.019	1.02	0.87	0.98
Q13564	NEDD8-activating enzyme E1 regulatory	NAE1	60.246	53.74	14.8	8	11	7	1.016	0.979	1.003	1.029	0.967	0.979	1.05	1.014	1.02	0.98	1.03
Q7Z4H8	KDEL motif-containing protein 2 OS=Ho	KDELC2	58.572	81.235	25	9	13	9	0.921	1.023	0.971	1.009	1.083	1.289	0.781	1.07	1.02	1.22	0.95
Q9H3Q1	Cdc42 effector protein 4 OS=Homo sapie	CDC42EP4	37.979	13.709	8.4	2	2	2	1.006	1.003	1.075	0.971	1.064	0.896	1.05	1.008	1.02	0.98	1.02
P53680	AP-2 complex subunit sigma OS=Homo s	AP2S1	17.018	33.843	28.9	4	4	4	1.081	1.047	1.077	1.09	0.938	1.105	0.868	0.709	1.02	0.96	0.74
O00159	Unconventional myosin-Ic OS=Homo sap	MYO1C	121.68	113.94	15.3	15	18	15	0.972	0.903	0.983	0.926	1.148	0.964	1.178	1.137	1.02	1.13	1.23
Q9ULA0	Aspartyl aminopeptidase OS=Homo sapi	DNPEP	52.428	66.321	22.5	9	13	9	1.002	1.04	1.089	0.99	0.913	1.007	0.951	0.888	1.02	0.94	0.90
P15291	Beta-1,4-galactosyltransferase 1 OS=Hor	B4GALT1	43.92	26.844	10.1	2	3	2	1.088	0.734	0.914	0.941	1.109	0.881	1.323	1.034	1.02	1.09	1.29
Q9P2R3	Rabankyrin-5 OS=Homo sapiens OX=96	ANKFY1	128.4	58.007	8.6	7	8	7	1.035	0.901	0.952	1.019	0.97	1.144	1.005	1.021	1.02	1.09	1.05
Q5BVD1	TPA-induced transmembrane protein OS=	TTMP	24.294	19.111	8.3	1	1	1	0.917	0.854	0.723	1.08	1.032	0.93	1.202	1.332	1.02	1.11	1.43
P14923	Junction plakoglobin OS=Homo sapiens	JUP	81.744	282.99	47.5	25	37	19	0.99	1.008	1.033	1.001	1.009	0.934	1.058	1.01	1.02	0.97	1.04
Q9BQ67	Glutamate-rich WD repeat-containing prc	GRWD1	49.419	49.269	10.5	4	5	4	1.023	1.045	1.072	1.033	1	1.146	0.785	0.844	1.02	1.04	0.79
P23193	Transcription elongation factor A protein	TCEA1	33.969	140.52	42.5	13	20	13	1.046	0.866	0.959	0.987	1.096	0.954	1.011	1.039	1.02	1.07	1.07
O43716	Glutamyl-tRNA(Gln) amidotransferase su	GATC	15.086	18.023	20.6	2	3	2	1.027	1.003	1.059	1.007	1.016	1.03	0.978	0.908	1.02	1.01	0.93
Q7LGA3	Heparan sulfate 2-O-sulfotransferase 1 O	HS2ST1	41.881	8.7051	4.5	1	1	1	1.009	1.029	1.058	1.016	0.967	0.869	1.085	1.008	1.02	0.90	1.03
Q9NZW5	MAGUK p55 subfamily member 6 OS=H	MPP6	61.116	31.113	9.3	4	4	4	0.918	1.066	0.901	1.118	0.902	1.051	1.065	1.014	1.02	0.98	1.05
P82094	TATA element modulatory factor OS=Ho	TMF1	122.84	72.089	11.2	9	9	9	1.024	0.962	0.999	1.022	1.06	0.997	0.96	0.97	1.02	1.04	0.97
Q9NVA1	Ubiquinol-cytochrome-c reductase compl	UQCC1	34.6	20.292	10.4	3	4	3	1.066	0.935	1.051	0.985	1.049	1.234	0.778	0.918	1.02	1.14	0.85
O00255	Menin OS=Homo sapiens OX=9606 GN	MEN1	68.023	18.731	4.1	2	2	2	1.031	0.913	1.023	0.955	1.16	0.906	1.088	0.985	1.02	1.06	1.07
Q5VTR2	E3 ubiquitin-protein ligase BRE1A OS=l	RNF20	113.66	195.34	22.7	18	27	17	0.967	0.977	0.997	0.981	1.032	0.999	1.043	1.015	1.02	1.04	1.06
P53041	Serine/threonine-protein phosphatase 5 C	PPP5C	56.878	129.39	36.7	15	18	14	1.009	0.948	0.977	1.014	1.066	0.944	0.929	1.038	1.02	1.03	1.01
Q96AE4	Far upstream element-binding protein 1 C	FUBP1	67.56	238.6	43.8	22	46	20	1.021	1.002	1.052	1.006	0.98	0.982	0.984	0.934	1.02	0.97	0.95
Q96S44	EKC/KEOPS complex subunit TP53RK (	TP53RK	28.16	22.736	11.5	3	5	3	1.053	0.981	1.08	0.989	1.025	1.041	0.933	0.924	1.02	1.02	0.91
P09110	3-ketoacyl-CoA thiolase, peroxisomal OS	ACAA1	44.292	124.84	42.5	10	12	10	1.026	1.011	1.06	1.012	1.128	1.197	0.757	0.946	1.02	1.14	0.84
Q15406	Nuclear receptor subfamily 6 group A me	NR6A1	54.382	30.818	7.9	3	5	3	0.841	1.202	1.093	0.985	0.928	0.729	1.151	0.869	1.02	0.81	0.99
O43432	Eukaryotic translation initiation factor 4	EIF4G3	176.65	21.354	2.5	3	3	2	1.018	0.911	0.911	1.051	1.079	0.947	1.118	0.962	1.02	1.05	1.08
Q99598	Translin-associated protein X OS=Homo	TSNAX	33.112	60.904	28.3	6	7	6	0.995	1.008	0.989	1.048	1.07	0.991	1.014	0.903	1.02	1.03	0.96
O75153	Clustered mitochondria protein homolog	CLUH	146.67	126.58	16.3	16	19	16	1.001	0.95	1.004	0.98	1.037	0.994	1.053	1.04	1.02	1.04	1.07
Q96QG7	Myotubularin-related protein 9 OS=Hom	MTMR9	63.461	15.203	4.4	2	2	2	0.999	1.012	1.113	0.932	0.978	1.269	0.909	0.9	1.02	1.12	0.90
O14949	Cytochrome b-c1 complex subunit 8 OS=	UQCRQ	9.9062	14.145	12.2	2	4	2	1.042	0.915	0.971	1.019	1.014	0.889	1.118	1.001	1.02	0.97	1.08
Q8WUW1	Protein BRICK1 OS=Homo sapiens OX=	BRK1	8.7448	43.023	54.7	4	6	4	1.043	1.101	1.188	0.992	0.952	0.975	0.868	0.903	1.02	0.90	0.83
Q9UJ70	N-acetyl-D-glucosamine kinase OS=Hom	NAGK	37.375	87.081	31.7	8	10	8	0.997	1.006	1.041	0.995	1.086	0.953	0.951	0.916	1.02	1.02	0.93
P49903	Selenide, water dikinase 1 OS=Homo sap	SEPHS1	42.91	39.91	18.1	5	6	5	1.014	1.053	1.087	1.014	0.945	1.133	0.943	0.976	1.02	1.01	0.93
O60476	Mannosyl-oligosaccharide 1,2-alpha-man	MAN1A2	73.003	23.962	6.1	3	3	3	1.039	0.912	0.994	0.989	1.009	1.119	0.934	1.042	1.02	1.09	1.01
O15127	Secretory carrier-associated membrane pr	SCAMP2	36.648	87.278	13.7	3	5	3	1.015	0.938	0.98	1.005	1.078	0.854	1.183	1.061	1.02	0.99	1.15
Q9BYX2	TBC1 domain family member 2A OS=Ho	TBC1D2	105.41	30.531	5	4	4	4	0.974	0.934	0.952	0.987	1.021	0.813	1.255	1.152	1.02	0.96	1.26
Q68CQ4	Digestive organ expansion factor homolo	DIEXF	87.054	51.98	11.8	7	8	7	0.976	0.875	0.891	0.99	1.101	1.016	1.123	1.045	1.02	1.14	1.17
Q9Y315	Deoxyribose-phosphate aldolase OS=Ho	DERA	35.23	64.397	34.3	8	10	8	1.005	0.921	0.976	0.981	1.181	1.035	0.967	1.048	1.02	1.15	1.05
Q9Y2H5	Pleckstrin homology domain-containing f	PLEKHA6	117.13	147.02	18.5	16	18	16	0.947	0.947	0.896	1.028	0.956	0.77	1.334	1.204	1.02	0.91	1.34
Q8IX12	Cell division cycle and apoptosis regulato	CCAR1	132.82	123.21	15.2	15	20	15	0.993	0.975	0.975	1.024	1.026	0.998	0.984	1.014	1.02	1.03	1.02
Q9UPN6	Protein SCAF8 OS=Homo sapiens OX=9	SCAF8	140.52	60.973	8.7	9	10	8	0.963	1.095	1.045	1.045	1.009	0.98	0.941	0.979	1.02	0.97	0.93
P35637	RNA-binding protein FUS OS=Homo sap	FUS	53.425	109.53	21.7	10	18	7	1.028	0.98	1.047	0.992	0.962	1.065	0.945	0.978	1.02	1.01	0.96
Q15643	Thyroid receptor-interacting protein 11 C	TRIP11	227.58	204.7	17.5	28	31	28	1.03	0.981	1.028	1.014	1.03	0.975	0.974	0.95	1.02	1.00	0.96
Q15910	Histone-lysine N-methyltransferase EZH2	EZH2	85.362	41.885	10.6	5	6	5	1.01	0.966	0.992	1.014	1.004	1.087	0.938	1.026	1.02	1.06	0.99
O14867	Transcription regulator protein BACH1 (	BACH1	81.957	13.613	3	2	2	2	0.977	1.093	0.996	1.105	0.927	1.198	0.661	1.052	1.01	1.03	0.83
Q9UK61	Protein TASOR OS=Homo sapiens OX=	FAM208A	189.03	43.306	3.8	6	7	6	0.993	0.945	0.965	1.002	1.046	0.897	1.116	0.923	1.01	1.00	1.05
Q9Y6Q5	AP-1 complex subunit mu-2 OS=Homo s	AP1M2	48.108	12.963	9.5	4	4	2	0.982	0.957	0.987	0.981	1.094	1.17	0.91	1.006	1.01	1.17	0.99
Q9H9C1	Spermatogenesis-defective protein 39 hor	VIPAS39	57.005	36.74	12.2	5	5	5	0.944	0.995	0.952	1.016	0.98	0.991	1.119	1.066	1.01	1.02	1.13
P18858	DNA ligase 1 OS=Homo sapiens OX=96	LIG1	101.73	80.401	14.8	11	14	11	0.95	0.867	0.89	0.954	1.084	0.999	1.134	1.137	1.01	1.15	1.25
Q96JB3	Hypermethylated in cancer 2 protein OS=	HIC2	66.155	81.908	17.2	8	8	8	1.042	0.988	1.05	1.01	0.98	0.96	0.943	0.902	1.01	0.96	0.91
Q8N9B5	Junction-mediating and -regulatory protei	JMY	111.44	7.7648	1.4	1	1	1	0.982	0.933	0.961	0.982	1	0.91	1.198	1.12	1.01	1.00	1.21
P60709	Actin, cytoplasmic 1 OS=Homo sapiens (	ACTB	41.736	323.31	61.1	18	184	6	0.939	1.062	1.007	1.023	0.898	0.957	1.096	1.038	1.01	0.93	1.07
P53701	Cytochrome c-type heme lyase OS=Homo	HCCS	30.601	53.073	23.1	5	5	5	1.009	1	1.033	1.005	1.005	1.225	0.802	0.974	1.01	1.11	0.88
P10644	cAMP-dependent protein kinase type I-al	PRKAR1A	42.981	57.95	14.4	5	6	5	1.014	1.002	1.056	0.989	0.987	0.99	1.13	0.943	1.01	0.98	1.03
Q9P2C4	Transmembrane protein 181 OS=Homo s	TMEM181	69.324	15.84	3.9	2	3	2	1.003	1.087	1.096	1.024	0.913	1.361	0.732	0.824	1.01	1.09	0.74
O15321	Transmembrane 9 superfamily member 1	TM9SF1	68.86	96.128	20	8	9	8	0.971	0.986	0.967	1.018	1.01	1.173	0.915	1.031	1.01	1.12	0.99
P39656	Dolichyl-diphosphooligosaccharide--prot	DDOST	50.8	86.83	28.1	10	17	10	0.981	1.046	1.082	0.974	0.939	1.24	0.936	0.973	1.01	1.07	0.94
Q9BSH5	Haloacid dehalogenase-like hydrolase do	HDHD3	28	24.42	21.5	3	3	3	1.021	0.939	0.996	0.992	1.014	0.908	1.168	0.961	1.01	0.98	1.09
Q15165	Serum paraoxonase/arylesterase 2 OS=Ho	PON2	39.38	33.157	17.2	4	6	4	0.968	1.004	1.017	0.983	1.017	1.029	1.122	0.951	1.01	1.04	1.05
Q13151	Heterogeneous nuclear ribonucleoprotein	HNRNPA0	30.84	137.59	39	8	18	8	0.959	1.016	1.012	0.991	1.008	1.009	0.842	1	1.01	1.02	0.93
Q16610	Extracellular matrix protein 1 OS=Homo	ECM1	60.673	26.541	9.8	4	4	4	0.977	0.929	0.935	0.998	1.041	1.039	1.126	1.029	1.01	1.09	1.13
P61201	COP9 signalosome complex subunit 2 O	COPS2	51.596	62.951	21.9	9	9	8	0.995	1.053	1.009	1.068	0.984	1.129	0.814	0.937	1.01	1.03	0.85
O15541	RING finger protein 113A OS=Homo sap	RNF113A	38.787	37.789	14.9	5	6	5	0.974	0.947	0.959	0.989	1.004	0.964	1.139	1.054	1.01	1.02	1.14
Q8NFQ8	Torsin-1A-interacting protein 2 OS=Hom	TOR1AIP2	51.263	19.621	9.6	4	4	3	1.013	0.985	1.06	0.966	1.066	1.175	0.724	0.943	1.01	1.12	0.83
P49757	Protein numb homolog OS=Homo sapien	NUMB	70.803	58.12	13.8	8	9	8	0.973	1.1	1.056	1.046	0.949	1.113	0.907	0.955	1.01	0.99	0.90
Q13442	28 kDa heat- and acid-stable phosphopro	PDAP1	20.63	72.688	32.6	8	11	8	0.976	1.034	1.003	1.035	1.008	1.03	1.017	1.032	1.01	1.01	1.02
P53999	Activated RNA polymerase II transcriptic	SUB1	14.395	47.879	29.1	4	10	4	1.025	0.993	1.024	1.022	0.942	1.139	0.9	0.947	1.01	1.03	0.92
Q06587	E3 ubiquitin-protein ligase RING1 OS=H	RING1	42.429	17.131	6.7	2	2	2	0.954	1.069	1.05	1.001	1.009	1.259	0.759	0.999	1.01	1.12	0.87
Q58FF8	Putative heat shock protein HSP 90-beta	HSP90AB2	44.348	9.4221	20.7	9	36	1	1.046	0.99	1.128	0.936	1.015	0.954	1.061	0.913	1.01	0.97	0.97
Q9BUL8	Programmed cell death protein 10 OS=Ho	PDCD10	24.701	51.35	23.6	4	4	4	1.059	0.978	1.055	1.01	1.023	1.065	1.032	0.973	1.01	1.03	0.98
P60510	Serine/threonine-protein phosphatase 4 c	PPP4C	35.08	52.261	30.3	6	10	5	1.012	1.028	1.065	1.003	1.006	0.902	1.115	0.978	1.01	0.94	1.03
Q96N46	Tetratricopeptide repeat protein 14 OS=H	TTC14	88.318	5.9889	1.3	1	1	1	0.974	1.003	1.018	0.986	0.954	1.068	1.091	0.994	1.01	1.02	1.05
O94906	Pre-mRNA-processing factor 6 OS=Hom	PRPF6	106.92	110.71	19.2	16	19	16	0.969	0.941	0.972	0.964	1.06	1.031	1.066	1.029	1.01	1.09	1.10
P68371	Tubulin beta-4B chain OS=Homo sapien	TUBB4B	49.83	315.18	60	20	93	1	1.009	1.059	1.062	1.034	0.983	0.989	0.935	0.903	1.01	0.95	0.89
Q13057	Bifunctional coenzyme A synthase OS=H	COASY	62.328	27.183	7.8	3	4	3	0.98	1.016	0.995	1.028	0.914	1.056	0.845	1.238	1.01	0.99	1.04
Q15369	Elongin-C OS=Homo sapiens OX=9606	ELOC	12.473	41.973	54.5	5	11	5	0.954	1.123	1.125	0.98	0.796	1.128	0.746	0.888	1.01	0.93	0.79
Q14966	Zinc finger protein 638 OS=Homo sapier	ZNF638	220.62	200.65	13.8	24	32	24	1.034	0.974	0.993	1.042	1.023	0.986	0.977	1.016	1.01	1.00	0.99
P23769	Endothelial transcription factor GATA-2	GATA2	50.499	55.771	16.9	5	6	3	1.023	1.075	1.059	1.067	0.888	1.028	0.882	0.852	1.01	0.91	0.83
O43402	ER membrane protein complex subunit 8	EMC8	23.773	89.378	48.1	8	8	7	0.998	0.984	0.961	1.047	1.03	0.911	0.962	1.005	1.01	0.98	0.99
Q8N4V1	Membrane magnesium transporter 1 OS=	MMGT1	14.686	64.841	18.3	1	2	1	0.982	1.079	1.056	1.032	0.901	1.084	0.936	0.983	1.01	0.96	0.93
O60244	Mediator of RNA polymerase II transcrip	MED14	160.6	31.639	3.5	5	7	5	0.931	0.978	0.896	1.038	0.988	0.952	1.12	1.122	1.01	1.02	1.17
Q9HD26	Golgi-associated PDZ and coiled-coil mo	GOPC	50.519	83.12	21.2	9	11	9	1.033	1.038	1.084	1.014	1.007	1.049	0.872	0.905	1.01	0.99	0.86
P49959	Double-strand break repair protein MRE	MRE11	80.592	100.75	23	13	15	13	0.979	0.895	0.904	0.994	1.108	1.054	0.974	1.154	1.01	1.15	1.14
Q9NP77	RNA polymerase II subunit A C-terminal	SSU72	22.574	36.728	22.7	4	5	4	0.983	1.048	1.072	0.985	0.93	0.964	1.046	1.018	1.01	0.93	1.02
P98170	E3 ubiquitin-protein ligase XIAP OS=Ho	XIAP	56.684	14.841	6.6	2	2	2	1.015	0.948	0.937	1.051	0.999	0.987	1.017	1.057	1.01	1.01	1.06
C9J7I0	UBAP1-MVB12-associated (UMA)-dom	UMAD1	15.2	39.828	16.8	1	1	1	0.971	1.085	1.077	1.005	0.943	0.885	1.11	1.002	1.01	0.89	1.03
Q6ZN17	Protein lin-28 homolog B OS=Homo sap	LIN28B	27.083	134.87	52.8	11	24	10	0.994	0.906	0.955	0.969	1.037	0.97	1.033	1.126	1.01	1.06	1.14
Q15631	Translin OS=Homo sapiens OX=9606 GI	TSN	26.183	28.581	14.9	4	5	4	0.976	1.005	0.948	1.058	1.076	0.984	1.008	1.031	1.01	1.04	1.03
Q96CT7	Coiled-coil domain-containing protein 12	CCDC124	25.835	105.57	22.9	4	6	4	0.953	1.029	1.082	0.925	1.086	1.009	1.017	0.992	1.01	1.06	1.01
Q9BU76	Multiple myeloma tumor-associated prote	MMTAG2	29.411	57.746	13.7	3	5	3	0.953	0.965	0.954	0.988	1.101	1.213	0.964	0.971	1.01	1.21	1.01
Q16762	Thiosulfate sulfurtransferase OS=Homo s	TST	33.429	18.145	8.4	2	2	2	0.877	1.289	1.11	1.083	0.661	1.454	0.711	0.923	1.01	0.98	0.75
P10588	Nuclear receptor subfamily 2 group F me	NR2F6	42.979	78.904	22	6	7	6	1.052	0.959	1.047	0.989	1.049	1.047	0.896	0.966	1.01	1.04	0.93
Q96DX5	Ankyrin repeat and SOCS box protein 9 (	ASB9	31.857	6.1638	6.5	1	1	1	1.124	0.971	1.169	0.952	1.136	0.763	1.13	0.734	1.01	0.91	0.89
P43243	Matrin-3 OS=Homo sapiens OX=9606 G	MATR3	94.622	190.76	23.7	17	26	17	0.987	1.04	1.035	1.017	1.021	1.024	0.996	0.953	1.01	1.01	0.96
Q13362	Serine/threonine-protein phosphatase 2A	PPP2R5C	61.06	28.608	8.6	4	4	2	0.969	0.978	0.919	1.052	1.012	1.022	1.01	1.085	1.01	1.04	1.08
P30085	UMP-CMP kinase OS=Homo sapiens OX	CMPK1	22.222	31.367	17.9	3	8	3	1.014	0.944	0.988	0.994	1.041	1.062	0.978	1.08	1.01	1.07	1.05
Q5VWZ2	Lysophospholipase-like protein 1 OS=Ho	LYPLAL1	26.316	27.832	19.4	4	5	4	0.988	1.052	1.025	1.04	1.03	0.993	0.849	0.935	1.01	0.99	0.87
Q9Y376	Calcium-binding protein 39 OS=Homo sa	CAB39	39.869	52.293	17.6	6	9	6	1.045	0.922	1.034	0.957	0.996	0.843	1.202	0.927	1.01	0.93	1.08
Q9H3Z4	DnaJ homolog subfamily C member 5 OS	DNAJC5	22.149	25.932	8.6	2	3	2	0.969	1.001	0.902	1.092	0.926	1.047	0.926	1.161	1.01	1.00	1.06
Q92917	G-patch domain and KOW motifs-contair	GPKOW	52.228	102.2	41.4	12	14	11	1.02	0.952	0.969	1.027	0.985	0.931	1.034	1.082	1.01	0.97	1.07
Q99873	Protein arginine N-methyltransferase 1 O	PRMT1	42.461	121.09	37.5	13	25	13	1.033	1.029	1.118	0.969	1.009	0.963	0.98	0.915	1.01	0.96	0.92
Q7L014	Probable ATP-dependent RNA helicase I	DDX46	117.36	264.57	29.5	27	45	27	1.005	0.986	0.992	1.023	1.033	1.009	0.985	0.981	1.01	1.03	0.99
Q9UMZ2	Synergin gamma OS=Homo sapiens OX=	SYNRG	140.65	12.821	1.8	2	2	2	0.931	0.996	0.875	1.075	0.981	1.034	1.046	1.122	1.01	1.05	1.13
P23368	NAD-dependent malic enzyme, mitochon	ME2	65.443	72.659	12.2	6	8	6	1.002	0.928	1.01	0.943	1.145	0.985	1.021	1.011	1.01	1.10	1.05
Q7Z3T8	Zinc finger FYVE domain-containing prc	ZFYVE16	168.9	60.287	7.1	9	10	9	0.973	0.981	0.979	0.998	1.054	1.003	0.964	1.043	1.01	1.05	1.03
O60216	Double-strand-break repair protein rad21	RAD21	71.689	109.66	20.8	10	13	10	0.943	1.037	0.995	1.008	1.06	1.059	1.043	0.986	1.01	1.07	1.02
Q9UGY1	Nucleolar protein 12 OS=Homo sapiens (	NOL12	24.663	13.226	16.9	2	3	2	0.953	0.882	0.831	1.025	1.121	1.31	0.776	1.189	1.01	1.32	1.07
Q9NZI5	Grainyhead-like protein 1 homolog OS=H	GRHL1	70.112	152.03	33	14	22	14	1.025	0.986	1.007	1.027	0.977	1.1	0.848	0.997	1.01	1.03	0.92
Q9GZZ9	Ubiquitin-like modifier-activating enzym	UBA5	44.863	22.499	8.4	3	3	3	1.006	1.094	1.135	0.989	0.918	1.282	0.732	0.826	1.01	1.05	0.74
P41208	Centrin-2 OS=Homo sapiens OX=9606 (	CETN2	19.738	28.82	26.7	4	5	4	1.023	0.994	1.053	0.987	0.96	0.961	0.983	1.022	1.01	0.95	0.99
Q9H0W9	Ester hydrolase C11orf54 OS=Homo sap	C11orf54	35.117	51.302	23.5	6	5	5	1.053	1.055	1.18	0.952	1.019	0.991	0.949	0.856	1.01	0.95	0.86
Q9Y3A5	Ribosome maturation protein SBDS OS=	SBDS	28.763	108.14	39.2	9	13	9	0.974	0.97	1.015	0.951	1.062	1.008	1.023	1.068	1.01	1.06	1.08
Q5T447	E3 ubiquitin-protein ligase HECTD3 OS	HECTD3	97.112	29.818	4.5	3	5	3	1.023	1.021	1.071	0.996	0.996	1.102	0.803	0.999	1.01	1.03	0.88
Q8N6T7	NAD-dependent protein deacetylase sirtu	SIRT6	39.118	6.6203	3.7	1	1	1	0.978	0.98	0.919	1.061	1.086	1.225	0.758	1.027	1.01	1.18	0.91
Q9NWY4	Histone PARylation factor 1 OS=Homo s	HPF1	39.436	46.435	22.3	7	7	7	0.981	0.983	1.014	0.972	0.975	1.018	1.02	1.042	1.01	1.01	1.05
O15550	Lysine-specific demethylase 6A OS=Hon	KDM6A	154.17	12.602	1.4	2	2	2	1.044	1.037	1.088	1.016	0.987	0.949	0.997	0.888	1.01	0.93	0.91
Q8IWD4	Coiled-coil domain-containing protein 11	CCDC117	30.541	12.192	6.1	2	2	2	0.967	0.665	0.662	0.988	1.37	0.915	1.118	1.412	1.01	1.40	1.55
Q9H2U2	Inorganic pyrophosphatase 2, mitochondr	PPA2	37.92	123.77	37.1	10	17	10	1.018	1.073	1.114	1	0.994	1.037	0.931	0.864	1.01	0.97	0.86
O60318	Germinal-center associated nuclear protei	MCM3AP	218.4	43.894	4.5	6	7	6	0.955	1.051	0.984	1.044	0.948	1.158	0.964	0.959	1.01	1.05	0.96
P22102	Trifunctional purine biosynthetic protein	GART	107.77	191.82	26.4	20	36	20	0.994	0.934	0.95	0.999	1.057	0.952	1.095	1.046	1.01	1.04	1.11
Q9NYQ8	Protocadherin Fat 2 OS=Homo sapiens C	FAT2	479.31	62.372	2.4	9	9	9	0.969	0.963	0.88	1.073	1.084	0.903	1.072	1.081	1.01	1.03	1.11
Q9BXB4	Oxysterol-binding protein-related protein	OSBPL11	83.642	35.137	8.6	5	6	5	0.995	1.041	1.021	1.037	0.88	1.052	1.01	1	1.01	0.95	0.99
Q6GMV3	Putative peptidyl-tRNA hydrolase PTRH	PTRHD1	15.805	55.118	47.1	5	9	5	0.989	1.054	1.028	1.037	0.955	1.016	0.903	0.95	1.01	0.96	0.91
Q6NXG1	Epithelial splicing regulatory protein 1 O	ESRP1	75.585	61.597	11.5	7	9	6	1.036	1.023	1.07	1.011	0.991	1.18	0.844	0.886	1.01	1.05	0.84
Q13148	TAR DNA-binding protein 43 OS=Homo	TARDBP	44.739	26.194	8	3	5	3	1.069	0.921	1.05	0.961	1.005	0.935	0.997	0.953	1.01	0.97	0.98
P55039	Developmentally-regulated GTP-binding	DRG2	40.746	39.741	20.1	6	10	6	0.996	0.997	1.039	0.975	1.043	0.975	1.035	1.006	1.01	1.01	1.02
Q9P0I2	ER membrane protein complex subunit 3	EMC3	29.952	69.758	27.6	6	8	6	1.014	0.905	0.944	0.995	0.999	1.006	0.98	1.036	1.01	1.04	1.05
O75354	Ectonucleoside triphosphate diphosphohy	ENTPD6	53.245	12.337	3.1	1	1	1	1.008	1.009	1.042	0.996	1.021	1	1.015	0.961	1.01	1.00	0.98
P13693	Translationally-controlled tumor protein (	TPT1	19.595	68.998	36	6	15	6	1.001	1.325	1.267	1.083	0.704	1.181	0.688	0.692	1.01	0.81	0.59
O75427	Leucine-rich repeat and calponin homolo	LRCH4	73.449	26.38	8.5	4	4	4	0.997	0.857	0.895	0.978	1.241	1.112	1.002	0.988	1.01	1.27	1.07
Q9Y3S2	Zinc finger protein 330 OS=Homo sapier	ZNF330	36.201	37.336	17.2	5	6	5	0.975	1.08	1.073	1.003	0.977	1.231	0.745	0.932	1.01	1.07	0.82
P54098	DNA polymerase subunit gamma-1 OS=I	POLG	139.56	22.586	4.2	4	4	4	0.99	0.976	0.982	1.004	1.17	1.093	0.865	0.981	1.01	1.15	0.94
P54760	Ephrin type-B receptor 4 OS=Homo sapi	EPHB4	108.27	78.967	10	8	10	5	1.042	1.03	1.069	1.024	1.032	1.294	0.699	0.817	1.01	1.12	0.73
Q9HCY8	Protein S100-A14 OS=Homo sapiens OX	S100A14	11.662	7.6413	10.6	1	1	1	1.01	0.965	1.003	0.992	0.965	0.884	1.148	1.091	1.01	0.94	1.13
Q6UXV4	MICOS complex subunit MIC27 OS=Ho	APOOL	29.159	15.595	10.8	2	2	2	1.023	0.96	1.015	0.988	1.007	0.893	1.256	0.896	1.01	0.96	1.09
Q9NVC6	Mediator of RNA polymerase II transcrip	MED17	72.889	39.749	9.2	6	8	6	1.004	0.989	1.041	0.972	1.116	0.987	0.958	0.928	1.01	1.06	0.95
P16615	Sarcoplasmic/endoplasmic reticulum calc	ATP2A2	114.76	245.78	25.3	25	43	25	1.03	0.989	1.031	1.008	1.036	0.992	0.93	0.957	1.01	1.00	0.93
Q12789	General transcription factor 3C polypepti	GTF3C1	238.87	165.73	12.1	22	24	22	1.056	0.965	1.032	1.009	1.079	0.908	1.082	1.003	1.01	0.98	1.03
Q5VZ89	DENN domain-containing protein 4C OS	DENND4C	212.71	111.66	7	11	14	11	0.992	1.059	1.039	1.032	0.946	0.97	1.188	0.919	1.01	0.93	1.03

Q03252	Lamin-B2 OS=Homo sapiens OX=9606 (	LMNB2	69.948	167.95	33.2	19	32	18	1.019	0.966	0.996	1.008	1.032	1.104	0.914	1.017	1.01	1.08	0.97
Q96ER3	Protein SAAL1 OS=Homo sapiens OX=9	SAAL1	53.557	71.097	20.9	8	11	8	0.959	1.038	0.965	1.051	0.95	1.172	0.843	1.03	1.01	1.06	0.94
Q9NYU2	UDP-glucose:glycoprotein glucosyltransf	UGGT1	177.19	282.83	26.1	31	47	30	0.986	1.025	1.01	1.02	1.004	1.027	0.958	0.948	1.01	1.01	0.95
Q9UG63	ATP-binding cassette sub-family F memb	ABCF2	71.289	121.49	19.6	11	16	11	0.992	1.02	1.078	0.953	1.003	1.201	0.882	0.968	1.01	1.10	0.92
P61221	ATP-binding cassette sub-family E memb	ABCE1	67.314	151.67	30.9	15	19	15	1.053	0.971	1.025	1.018	0.994	0.921	0.973	0.99	1.01	0.95	0.97
O95168	NADH dehydrogenase [ubiquinone] 1 be	NDUFB4	15.208	26.361	23.3	2	4	2	0.883	1.055	0.99	0.966	1.069	1.176	0.868	1.182	1.01	1.16	1.06
Q9UKX7	Nuclear pore complex protein Nup50 OS	NUP50	50.144	162.34	45.7	17	17	16	1.004	0.946	0.963	1.005	1.063	0.956	1.107	0.919	1.01	1.04	1.04
Q9UKV8	Protein argonaute-2 OS=Homo sapiens C	AGO2	97.207	28.226	10.9	7	7	4	0.994	0.956	0.949	1.019	1.019	0.85	1.1	1.118	1.01	0.96	1.14
Q96IR2	Zinc finger protein 845 OS=Homo sapier	ZNF845	113.13	49.825	12.6	5	6	2	0.91	0.973	0.998	0.902	1.084	1.428	0.878	1.035	1.01	1.33	1.02
Q9H061	Transmembrane protein 126A OS=Homo	TMEM126	21.527	20.608	15.4	2	2	2	1.011	0.993	1.038	0.984	1.072	1.244	0.828	0.892	1.01	1.16	0.86
Q16795	NADH dehydrogenase [ubiquinone] 1 alp	NDUFA9	42.509	80.347	22.8	7	10	7	1.051	0.954	1.024	0.999	1.01	0.981	0.964	0.951	1.01	0.99	0.96
Q9BUE0	Mediator of RNA polymerase II transcrip	MED18	23.662	11.964	7.7	2	2	2	0.791	1.226	1.043	0.992	0.937	0.967	1.333	0.932	1.01	0.94	1.12
Q15042	Rab3 GTPase-activating protein catalytic	RAB3GAP	110.52	136.3	19.4	14	15	14	1.009	1.012	1.01	1.029	0.96	1.023	0.943	0.914	1.01	0.98	0.92
Q7RTV0	PHD finger-like domain-containing prote	PHF5A	12.405	48.868	55.5	5	6	5	1.01	0.925	0.97	0.982	1.053	0.98	0.996	0.992	1.01	1.05	1.03
Q8N9V3	WD repeat, SAM and U-box domain-con	WDSUB1	52.817	16.916	8.8	3	3	3	0.939	1.232	1.103	1.087	0.673	1.185	0.868	0.965	1.01	0.86	0.84
Q68EM7	Rho GTPase-activating protein 17 OS=H	ARHGAP1	95.436	78.77	14.8	10	14	10	0.962	0.987	0.999	0.967	1.029	1.288	0.855	1.028	1.01	1.19	0.97
Q9Y4P3	Transducin beta-like protein 2 OS=Homo	TBL2	49.797	62.58	22.4	9	11	9	0.98	0.983	1	0.98	0.996	1.025	1.027	1.064	1.01	1.03	1.07
P69849	Nodal modulator 3 OS=Homo sapiens O	NOMO3	134.13	6.8641	18	16	20	1	1.007	1.094	1.07	1.049	0.931	1.005	0.9	0.963	1.01	0.92	0.89
Q96JI7	Spatacsin OS=Homo sapiens OX=9606 (	SPG11	278.86	11.905	0.9	2	2	2	0.99	0.996	1.003	1	0.95	1.006	1.013	1.115	1.01	0.98	1.07
Q9NSD9	Phenylalanine--tRNA ligase beta subunit	FARSB	66.115	159.31	32.4	18	27	18	1.001	0.987	1.006	0.999	0.987	0.955	1.001	0.979	1.01	0.98	1.00
Q8WXF0	Serine/arginine-rich splicing factor 12 OS	SRSF12	30.512	20.875	8.4	2	2	2	1.004	0.986	0.993	1.014	1.124	0.874	1.129	0.909	1.01	1.00	1.02
P30038	Delta-1-pyrroline-5-carboxylate dehydrog	ALDH4A1	61.719	53.048	17.6	8	9	8	0.968	0.909	0.933	0.96	1.104	1.308	0.853	1.005	1.01	1.29	0.99
P53367	Arfaptin-1 OS=Homo sapiens OX=9606	ARFIP1	41.738	74.885	26.8	8	10	8	1.016	0.987	1.006	1.014	0.988	0.918	1.04	0.957	1.01	0.95	1.00
P43686	26S proteasome regulatory subunit 6B O	PSMC4	47.366	156.8	40	14	28	13	1.032	1.02	1.05	1.019	1.003	0.966	0.962	0.911	1.01	0.96	0.91
Q02040	A-kinase anchor protein 17A OS=Homo s	AKAP17A	80.735	36.233	9.4	5	6	5	1.01	0.937	0.939	1.024	1.069	1.202	0.886	0.913	1.01	1.17	0.92
P31942	Heterogeneous nuclear ribonucleoprotein	HNRNPH3	36.926	135.98	40.5	11	22	10	0.98	0.989	0.989	0.996	1.013	1.03	0.995	1.027	1.01	1.04	1.03
Q2TAL8	Glutamine-rich protein 1 OS=Homo sapie	QRICH1	86.435	42.838	7.7	6	6	6	1.054	0.927	0.996	1.001	1.17	0.855	1.101	0.99	1.01	1.02	1.06
Q14686	Nuclear receptor coactivator 6 OS=Homo	NCOA6	219.14	11.908	0.8	2	2	2	1.009	0.976	1.005	0.996	0.987	0.901	1.141	1.036	1.01	0.95	1.10
Q96DF8	Splicing factor ESS-2 homolog OS=Hom	ESS2	52.567	60.362	14.9	4	4	4	0.996	1.032	1.052	0.992	1.015	1.139	0.94	0.898	1.01	1.06	0.91
P61970	Nuclear transport factor 2 OS=Homo sap	NUTF2	14.478	131.44	51.2	4	18	4	0.992	1.039	1.075	0.972	0.956	0.929	1.087	0.984	1.01	0.93	1.02
Q8N1P7	Beta/gamma crystallin domain-containing	CRYBG2	68.645	49.417	11.4	5	7	5	0.932	0.995	0.916	1.026	1.003	1.031	1.061	1.113	1.01	1.06	1.13
Q9Y2K7	Lysine-specific demethylase 2A OS=Hon	KDM2A	132.79	18.791	3	3	3	3	0.991	0.952	0.944	1.014	1.031	1.251	0.821	1.056	1.01	1.17	0.97
Q96EP5	DAZ-associated protein 1 OS=Homo sap	DAZAP1	43.383	81.121	23.8	6	11	6	1.021	1.059	1.097	0.999	0.915	0.985	1.033	0.905	1.01	0.91	0.93
Q8IWZ3	Ankyrin repeat and KH domain-containin	ANKHD1	269.45	179.38	10.8	18	21	11	0.971	0.987	0.98	0.993	1.061	1.131	0.921	1.032	1.01	1.12	1.00
O15371	Eukaryotic translation initiation factor 3	EIF3D	63.972	207.33	42.7	16	25	16	0.962	1	0.996	0.981	0.97	1.044	1.024	1.049	1.01	1.03	1.06
Q96ED9	Protein Hook homolog 2 OS=Homo sapie	HOOK2	83.206	17.971	3.3	3	3	3	0.981	1.007	1.048	0.955	0.993	0.995	1.106	1.02	1.01	1.00	1.07
O43143	Pre-mRNA-splicing factor ATP-depender	DHX15	90.932	191.97	32.6	23	37	21	1.019	0.973	1.009	0.998	1.008	1.01	1	0.954	1.01	1.01	0.98
O75044	SLIT-ROBO Rho GTPase-activating prot	SRGAP2	120.87	30.882	5.2	5	6	5	0.893	1.103	0.937	1.074	0.927	1.186	0.883	1.093	1.01	1.06	0.99
P14678	Small nuclear ribonucleoprotein-associat	SNRPB	24.61	71.689	23.8	5	10	5	0.996	1.007	1.012	1.006	0.988	1.096	1.019	1.001	1.01	1.04	1.01
Q8TAQ2	SWI/SNF complex subunit SMARCC2 (	SMARCC2	132.88	62.526	15.5	15	22	8	1.021	0.998	1.007	1.027	1.067	1.219	0.798	0.997	1.01	1.13	0.89
Q9H089	Large subunit GTPase 1 homolog OS=Ho	LSG1	75.225	41.291	9.9	6	7	6	1.001	0.887	0.985	0.917	1.068	1.217	1.028	1.015	1.01	1.21	1.08
Q9H0V9	VIP36-like protein OS=Homo sapiens O	LMAN2L	39.71	28.848	12.9	4	5	4	1.063	0.993	0.989	1.082	0.939	0.962	0.996	0.919	1.01	0.92	0.93
Q9UK58	Cyclin-L1 OS=Homo sapiens OX=9606 (	CCNL1	59.633	7.6017	1.9	1	1	1	0.962	1.115	1.038	1.054	0.89	1.136	0.772	1.095	1.01	0.98	0.90
Q99766	ATP synthase subunit s, mitochondrial O	ATP5S	24.866	21.741	14.9	3	4	3	0.96	0.872	0.864	0.981	1.187	1.233	0.846	1.168	1.01	1.32	1.10
Q96M27	Protein PRRC1 OS=Homo sapiens OX=9	PRRC1	46.701	28.164	10.1	4	6	4	0.984	1.001	0.968	1.031	0.951	1.068	0.964	1.074	1.01	1.02	1.03
Q13480	GRB2-associated-binding protein 1 OS=	GAB1	76.615	58.675	14.8	8	10	8	0.977	1.015	0.979	1.027	0.967	0.964	1.086	1.046	1.01	0.97	1.07
Q29RF7	Sister chromatid cohesion protein PDS5	PDS5A	150.83	222.05	19.8	22	26	21	0.984	1.046	1.048	0.996	0.97	1.13	0.945	0.981	1.01	1.03	0.95
Q9Y4B4	Helicase ARIP4 OS=Homo sapiens OX=	RAD54L2	162.77	21.307	3.3	3	3	3	1.123	0.916	0.995	1.058	1.081	0.792	1.054	0.886	1.01	0.92	0.95
P26639	Threonine--tRNA ligase, cytoplasmic OS	TARS	83.434	210.19	36.2	22	35	21	0.962	0.957	0.955	0.977	1.041	0.961	1.072	1.065	1.01	1.04	1.11
Q8IXI1	Mitochondrial Rho GTPase 2 OS=Homo	RHOT2	68.117	76.099	14.7	6	8	6	0.986	1.105	1.08	1.025	0.936	1.257	0.729	0.919	1.01	1.05	0.79
Q9H6V9	Lipid droplet-associated hydrolase OS=H	LDAH	37.318	47.469	19.7	7	8	7	1.034	1.057	1.139	0.966	0.993	1.037	0.929	0.936	1.01	0.97	0.89
Q9BW27	Nuclear pore complex protein Nup85 OS	NUP85	75.019	91.457	18.1	12	13	12	0.978	0.977	0.995	0.973	1.007	1.009	1.02	0.975	1.01	1.03	1.02
O75822	Eukaryotic translation initiation factor 3	EIF3J	29.062	58.536	28.3	7	11	7	1.002	0.991	0.994	1.012	0.992	0.937	1.086	0.998	1.01	0.97	1.05
Q4G0F5	Vacuolar protein sorting-associated prote	VPS26B	39.154	38.387	11.9	3	3	3	1.007	1.01	1.017	1.013	1.053	1.241	0.79	0.913	1.01	1.14	0.84
O14686	Histone-lysine N-methyltransferase 2D O	KMT2D	593.38	30.082	1.1	5	5	5	0.934	1.098	1.112	0.933	1.023	1.033	1.168	0.991	1.01	1.01	1.06
P52565	Rho GDP-dissociation inhibitor 1 OS=H	ARHGDIA	23.207	99.289	22.5	5	13	5	1.024	1.025	1.009	1.053	0.907	0.904	1.056	0.991	1.01	0.88	1.00
Q9Y5S2	Serine/threonine-protein kinase MRCK b	CDC42BPB	194.31	72.833	6.5	10	11	9	0.953	0.951	0.95	0.966	1.051	0.948	1.233	1.052	1.01	1.05	1.20
P18031	Tyrosine-protein phosphatase non-recept	PTPN1	49.966	111.26	37.2	13	20	13	0.967	0.976	0.967	0.988	1.002	0.991	1.097	1.021	1.01	1.03	1.09
P49790	Nuclear pore complex protein Nup153 O	NUP153	153.94	323.31	27.4	30	41	30	1.018	0.939	0.975	0.994	1.079	1.001	0.954	0.984	1.01	1.06	0.99
Q86V81	THO complex subunit 4 OS=Homo sapie	ALYREF	26.888	211.58	49	12	19	10	0.974	0.989	1.03	0.945	1.027	1.284	0.891	1.002	1.01	1.18	0.96
Q9NVI7	ATPase family AAA domain-containing p	ATAD3A	71.368	102.42	26.3	13	22	6	1.046	0.918	0.986	0.99	1.083	0.927	1.046	1.06	1.01	1.02	1.07
Q8NC96	Adaptin ear-binding coat-associated prote	NECAP1	29.737	19.693	12	3	4	3	1.072	1.056	1.154	0.987	0.898	1.283	0.801	0.76	1.01	1.02	0.73
Q86Y91	Kinesin-like protein KIF18B OS=Homo s	KIF18B	94.222	27.316	5.9	4	4	4	0.766	1.21	1.014	0.974	0.893	1.594	0.835	0.987	1.01	1.26	0.92
O00750	Phosphatidylinositol 4-phosphate 3-kinas	PIK3C2B	184.77	14.138	1.3	2	2	2	0.934	1.042	1.002	0.986	0.958	0.969	1.085	1.153	1.01	0.98	1.13
Q13426	DNA repair protein XRCC4 OS=Homo s	XRCC4	38.286	47.423	15.8	5	8	5	1.017	0.963	0.995	0.997	0.995	0.955	1.063	0.991	1.01	0.98	1.04
P46020	Phosphorylase b kinase regulatory subun	PHKA1	137.31	16.304	3.3	3	3	3	0.967	1.022	0.984	1.017	0.989	1.061	1.023	1.012	1.01	1.03	1.02
Q9UBK8	Methionine synthase reductase OS=Hom	MTRR	80.409	32.023	7.6	4	4	4	0.985	1.013	1.015	0.995	0.986	1.088	0.883	0.961	1.01	1.04	0.92
A5YKK6	CCR4-NOT transcription complex subun	CNOT1	266.94	190.92	13.9	28	35	28	1.017	0.982	0.998	1.013	1.021	0.976	1.058	0.966	1.01	1.00	1.01
P49321	Nuclear autoantigenic sperm protein OS=	NASP	85.237	323.31	40.5	23	47	23	1.006	0.994	0.999	1.013	0.984	1	0.999	1.009	1.01	0.99	1.00
O75533	Splicing factor 3B subunit 1 OS=Homo s	SF3B1	145.83	323.31	35.4	38	62	37	1.001	1.027	1.023	1.017	0.973	1.071	0.925	0.972	1.01	1.01	0.94
Q96RL1	BRCA1-A complex subunit RAP80 OS=	UIMC1	79.727	16.367	4	2	2	2	1.019	1.031	1.006	1.056	0.942	0.885	1.151	0.903	1.01	0.89	1.00
Q96SB4	SRSF protein kinase 1 OS=Homo sapien	SRPK1	74.324	45.951	11.9	6	8	5	0.973	0.946	0.932	0.998	1.044	1.226	0.963	1.006	1.01	1.18	1.03
Q7L1W4	Volume-regulated anion channel subunit	LRRC8D	98.2	12.145	2.2	2	2	2	0.987	0.986	0.931	1.053	0.939	0.891	1.101	1.147	1.01	0.93	1.14
Q99714	3-hydroxyacyl-CoA dehydrogenase type-2	HSD17B10	26.923	96.079	53.3	10	14	10	1.005	1.005	1.026	0.995	0.986	1.023	1.025	0.983	1.01	1.00	1.00
Q9Y305	Acyl-coenzyme A thioesterase 9, mitocho	ACOT9	49.901	15.218	4.3	2	2	2	1.026	0.838	0.914	0.96	1.024	1.043	1.172	1.089	1.01	1.11	1.21
Q13547	Histone deacetylase 1 OS=Homo sapiens	HDAC1	55.102	60.172	29	10	13	7	0.996	1.058	1.022	1.043	1.011	0.913	0.969	0.939	1.01	0.94	0.93
Q9H488	GDP-fucose protein O-fucosyltransferase	POFUT1	43.955	58.633	21.1	6	8	6	0.977	1.097	1.052	1.033	0.953	1.195	0.904	0.922	1.01	1.04	0.88
P06400	Retinoblastoma-associated protein OS=H	RB1	106.16	27.203	4.4	4	4	4	1.019	0.873	0.969	0.933	1.196	1.02	1.047	1.031	1.01	1.17	1.10
P30622	CAP-Gly domain-containing linker prote	CLIP1	162.24	249.94	23.2	32	41	32	0.961	0.972	0.93	1.013	1.017	1.004	1.033	1.075	1.01	1.05	1.09
Q53HC9	EARP-interacting protein OS=Homo sap	EIPR1	43.603	30.308	11.6	3	3	3	0.99	0.97	0.977	0.993	1.044	0.954	1.063	1.08	1.01	1.02	1.09
P27540	Aryl hydrocarbon receptor nuclear transl	ARNT	86.636	22.342	4.6	3	3	3	1.039	0.936	0.946	1.039	1.017	0.926	1.015	1.083	1.01	0.98	1.06
Q12959	Disks large homolog 1 OS=Homo sapien	DLG1	100.45	94.056	11.5	9	12	8	1.005	0.998	1.009	1.004	1.062	1.058	0.933	0.991	1.00	1.06	0.96
Q9UI30	Multifunctional methyltransferase subuni	TRMT112	14.199	24.177	21.6	2	6	2	0.986	1.025	0.945	1.076	1.016	1.098	0.924	0.923	1.00	1.05	0.92
Q15005	Signal peptidase complex subunit 2 OS=	SPCS2	25.003	121.22	46.5	8	14	8	1.014	1.021	1.013	1.032	1.037	0.946	1.009	1.001	1.00	0.97	0.99
Q13045	Protein flightless-1 homolog OS=Homo s	FLII	144.75	249.02	22.4	24	37	24	1.034	1.004	1.03	1.018	0.952	0.899	1.04	0.972	1.00	0.91	0.99
Q15843	NEDD8 OS=Homo sapiens OX=9606 Gl	NEDD8	9.0714	17.959	24.7	2	3	2	1.068	1.041	1.057	1.062	0.931	0.853	1.039	0.904	1.00	0.85	0.92
P10768	S-formylglutathione hydrolase OS=Homo	ESD	31.462	37.879	19.1	5	10	5	0.973	1.152	1.141	0.994	0.946	1.106	0.979	1.019	1.00	0.97	0.94
Q5THK1	Protein PRR14L OS=Homo sapiens OX=	PRR14L	237.3	32.891	2.9	5	6	5	1.007	1.121	1.083	1.055	0.863	0.977	0.96	0.947	1.00	0.86	0.90
Q9Y2W1	Thyroid hormone receptor-associated pro	THRAP3	108.66	199.64	20.6	18	26	17	0.971	0.986	0.977	0.989	1.004	0.999	1.136	1.026	1.00	1.02	1.10
Q9UJY4	ADP-ribosylation factor-binding protein	GGA2	67.15	91.543	15	7	7	7	0.973	1.03	0.993	1.019	1.05	0.914	1.085	1.043	1.00	0.98	1.06
Q9Y3D6	Mitochondrial fission 1 protein OS=Hom	FIS1	16.937	35.393	22.4	3	4	3	1.027	0.986	1.013	1.009	1.033	0.966	1.003	0.991	1.00	0.99	0.99
Q9NS87	Kinesin-like protein KIF15 OS=Homo sa	KIF15	160.16	20.812	2.4	3	3	3	0.96	1.057	1.067	0.959	0.984	1.264	0.824	1.012	1.00	1.11	0.91
P62633	Cellular nucleic acid-binding protein OS=	CNBP	19.463	41.256	29.4	5	8	5	0.904	1.114	0.969	1.058	1.01	1.142	0.943	1.055	1.00	1.07	0.99
P61962	DDB1- and CUL4-associated factor 7 OS	DCAF7	38.926	73.13	22.5	6	7	6	1.031	0.989	1.051	0.978	1.009	1.12	0.94	0.979	1.00	1.05	0.95
Q96PU8	Protein quaking OS=Homo sapiens OX=	QKI	37.67	100.65	29.3	9	15	9	1.013	1.015	1.013	1.024	1.056	0.964	1.035	0.95	1.00	1.00	0.98
Q9UKF6	Cleavage and polyadenylation specificity	CPSF3	77.485	50.002	12.3	7	7	7	1.011	1.02	1.043	0.997	0.999	1.118	0.938	0.927	1.00	1.04	0.92
P56181	NADH dehydrogenase [ubiquinone] flavo	NDUFV3	11.94	6.0221	7.4	1	2	1	0.993	1.06	1.063	0.999	0.99	1.221	0.85	0.89	1.00	1.08	0.85
P09012	U1 small nuclear ribonucleoprotein A OS	SNRPA	31.279	117.87	33.3	7	9	6	1.033	1.036	1.044	1.034	0.919	1.009	1.121	1.029	1.00	0.93	1.04
O14548	Cytochrome c oxidase subunit 7A-related	COX7A2L	12.615	29.644	19.3	2	3	2	1.02	1.103	1.119	1.013	1.023	1.136	0.757	0.863	1.00	1.02	0.76
Q9BVG9	Phosphatidylserine synthase 2 OS=Homo	PTDSS2	56.252	16.342	5.7	2	2	2	0.99	0.909	0.901	1.006	1.127	0.891	1.169	1.062	1.00	1.06	1.17
P50613	Cyclin-dependent kinase 7 OS=Homo sap	CDK7	39.038	42.944	15.9	6	7	6	1.013	0.942	0.997	0.966	1.15	1.193	0.929	1.009	1.00	1.20	0.99
P22626	Heterogeneous nuclear ribonucleoprotein	HNRNPA2	37.429	208.49	58.6	17	51	17	0.969	0.988	0.96	1.005	1.002	1.028	0.981	0.989	1.00	1.04	1.01
Q92598	Heat shock protein 105 kDa OS=Homo s	HSPH1	96.864	299.16	41	29	52	26	0.994	1.023	1.018	1.007	1.058	1.225	0.819	0.926	1.00	1.13	0.87
O60427	Fatty acid desaturase 1 OS=Homo sapien	FADS1	51.964	23.712	9.5	3	4	3	0.915	0.85	0.803	0.969	1.204	1.194	1.023	1.193	1.00	1.36	1.26
Q96AQ6	Pre-B-cell leukemia transcription factor-i	PBXIP1	80.642	98.02	17.1	10	14	10	0.994	1.035	1.005	1.032	0.996	0.982	0.958	1.025	1.00	0.97	0.98
P31040	Succinate dehydrogenase [ubiquinone] fl	SDHA	72.691	148.03	23.6	11	18	10	1.042	0.991	1.082	0.959	1.048	0.981	0.94	0.929	1.00	1.00	0.92
Q9Y5K6	CD2-associated protein OS=Homo sapien	CD2AP	71.45	134.4	27.2	15	21	15	1.02	1.075	1.063	1.04	0.941	0.961	0.96	0.947	1.00	0.91	0.91
O14495	Phospholipid phosphatase 3 OS=Homo s	PLPP3	35.116	16.428	7.1	2	2	2	0.878	0.978	0.877	0.986	1.228	1.312	0.851	1.055	1.00	1.37	1.03
Q99816	Tumor susceptibility gene 101 protein OS	TSG101	43.944	95.015	26.7	8	9	8	0.959	0.987	0.952	1.001	1.011	1.031	0.968	1.033	1.00	1.05	1.03
Q9BVL2	Nucleoporin p58/p45 OS=Homo sapiens	NUP58	60.896	21.739	5	2	3	2	1.005	0.96	0.975	0.997	1.114	1.116	0.987	0.995	1.00	1.13	1.01
P12814	Alpha-actinin-1 OS=Homo sapiens OX=9	ACTN1	103.06	323.31	60.8	45	120	31	0.982	1.031	1.01	1.01	0.912	0.997	1.076	1.048	1.00	0.95	1.06
P30153	Serine/threonine-protein phosphatase 2A	PPP2R1A	65.308	201.3	41.8	21	38	17	1.02	1.002	1.011	1.018	1.001	1.089	0.93	0.976	1.00	1.03	0.94
Q14126	Desmoglein-2 OS=Homo sapiens OX=96	DSG2	122.29	185.47	22.9	17	22	17	1.012	1.027	1.061	0.985	0.932	0.958	1.019	0.993	1.00	0.93	0.99
Q96KP4	Cytosolic non-specific dipeptidase OS=H	CNDP2	52.878	184.22	40	14	26	13	0.992	1.071	1.041	1.029	0.944	0.979	0.983	0.953	1.00	0.93	0.94
Q9NQG5	Regulation of nuclear pre-mRNA domain	RPRD1B	36.899	110.61	26.7	7	8	6	1.014	0.969	1.015	0.974	1.084	0.994	0.981	0.979	1.00	1.05	0.99
Q86X55	Histone-arginine methyltransferase CAR	CARM1	65.853	55.892	15.6	8	16	8	1.076	1.017	1.055	1.044	0.948	1.102	0.746	0.988	1.00	0.98	0.83
O95453	Poly(A)-specific ribonuclease PARN OS	PARN	73.45	67.692	15.6	9	15	9	0.996	0.945	0.964	0.982	1.049	1.015	1.065	0.956	1.00	1.06	1.04
O43353	Receptor-interacting serine/threonine-pro	RIPK2	61.194	27.591	9.4	4	4	4	0.985	1.009	0.949	1.05	0.958	0.855	1.147	1.08	1.00	0.91	1.12
Q9NU22	Midasin OS=Homo sapiens OX=9606 Gl	MDN1	632.81	323.31	9.6	42	53	42	0.996	1.003	0.997	1.007	1.059	1.166	0.915	0.963	1.00	1.11	0.94
Q9Y217	Myotubularin-related protein 6 OS=Hom	MTMR6	71.967	12.607	3.7	2	2	2	0.964	1.079	0.937	1.111	0.889	0.871	1.079	1.079	1.00	0.86	1.06
Q5T200	Zinc finger CCCH domain-containing pro	ZC3H13	196.63	23.389	2.9	4	5	4	0.927	1.005	0.878	1.058	0.987	1.015	0.9	1.058	1.00	1.04	1.01
Q5T8D3	Acyl-CoA-binding domain-containing pro	ACBD5	60.091	15.422	4.1	2	2	2	1.038	0.929	0.997	0.974	1.023	1.37	0.796	0.921	1.00	1.22	0.87
Q9BVK6	Transmembrane emp24 domain-containin	TMED9	27.277	68.356	22.1	4	10	3	0.969	1.017	0.966	1.024	1.005	0.936	1.1	1.063	1.00	0.98	1.09
Q9BTV4	Transmembrane protein 43 OS=Homo sap	TMEM43	44.875	32.787	13.2	5	5	5	1.019	0.969	0.95	1.042	0.999	1.033	0.937	1.017	1.00	1.02	0.98
Q9UNF0	Protein kinase C and casein kinase substr	PACSIN2	55.738	118.22	30	11	15	10	0.966	1.038	1.001	1.007	0.964	0.945	1.137	1.038	1.00	0.95	1.09
O14745	Na(+)/H(+) exchange regulatory cofactor	SLC9A3R1	38.868	74.989	29.3	10	12	9	0.973	1.038	1.003	1.012	0.984	1.055	0.971	1.028	1.00	1.01	0.99
P18074	General transcription and DNA repair fac	ERCC2	86.908	83.27	13.3	8	10	8	0.982	1.065	0.996	1.055	0.938	1.052	0.969	1.001	1.00	0.97	0.96
Q9H2U1	ATP-dependent RNA helicase DHX36 O	DHX36	114.76	100.09	13.3	11	12	11	1.044	1.04	1.089	0.999	0.971	0.996	0.972	0.911	1.00	0.94	0.90
P42356	Phosphatidylinositol 4-kinase alpha OS=	PI4KA	236.83	141.74	9.7	17	17	17	1.011	0.896	0.916	0.994	1.16	1.039	0.991	1	1.00	1.15	1.04
Q96I25	Splicing factor 45 OS=Homo sapiens OX	RBM17	44.961	56.573	22.2	8	12	8	1.037	0.961	1.03	0.971	1.032	1.025	1.019	0.926	1.00	1.03	0.97
P14314	Glucosidase 2 subunit beta OS=Homo sa	PRKCSH	59.425	145.53	36.4	12	19	12	0.976	1.022	1.036	0.965	1.012	1.05	0.916	1.01	1.00	1.03	0.96
Q969G3	SWI/SNF-related matrix-associated actin	SMARCE1	46.649	97.538	19	5	8	5	1.036	1.005	1.037	1.007	0.981	0.978	0.998	0.928	1.00	0.96	0.94
P40616	ADP-ribosylation factor-like protein 1 O	ARL1	20.417	70.657	32	5	9	5	1.014	0.971	0.99	0.997	1.056	0.909	1.023	0.995	1.00	0.99	1.02
Q05682	Caldesmon OS=Homo sapiens OX=9606	CALD1	93.23	169.37	24.1	19	33	18	1.023	0.968	0.988	1.005	0.941	0.927	1.123	1.074	1.00	0.94	1.10
Q9H7H0	Methyltransferase-like protein 17, mitoch	METTL17	50.733	6.7489	1.8	1	1	1	1.033	0.978	1.003	1.01	1.122	1.333	0.68	0.865	1.00	1.22	0.77
P57737	Coronin-7 OS=Homo sapiens OX=9606	CORO7	100.6	103.71	14.8	10	13	10	0.983	1.109	1.076	1.018	0.871	0.984	1.058	0.962	1.00	0.89	0.97
P49427	Ubiquitin-conjugating enzyme E2 R1 OS	CDC34	26.736	7.9712	4.7	1	1	1	1.03	0.887	0.894	1.024	0.994	1.055	0.957	1.184	1.00	1.07	1.12
Q96FV9	THO complex subunit 1 OS=Homo sapie	THOC1	75.665	83.597	20.7	12	14	12	0.983	0.971	0.997	0.958	1.03	1.021	0.982	1.003	1.00	1.05	1.02
Q9UDY2	Tight junction protein ZO-2 OS=Homo s	TJP2	133.96	323.31	28.9	28	57	28	0.998	0.985	0.974	1.01	0.965	0.965	1.081	1.03	1.00	0.97	1.06
P62875	DNA-directed RNA polymerases I, II, and	POLR2L	7.645	7.7387	16.4	1	2	1	1.035	0.965	0.93	1.071	0.902	0.947	1.125	1.001	1.00	0.92	1.06
Q9NQ50	39S ribosomal protein L40, mitochondria	MRPL40	24.49	32.214	30.1	4	6	4	1.004	1.039	1.012	1.032	1.076	1.087	0.945	0.876	1.00	1.06	0.89
Q7Z4H3	HD domain-containing protein 2 OS=Ho	HDDC2	23.39	51.16	45.6	6	7	6	0.941	1.12	1.064	0.998	0.905	1.28	0.767	0.95	1.00	1.06	0.83
P52435	DNA-directed RNA polymerase II subuni	POLR2J	13.293	64.898	37.6	5	6	5	1.009	1.067	1.078	0.999	1.022	0.922	1.12	0.927	1.00	0.94	0.99
Q92896	Golgi apparatus protein 1 OS=Homo sapi	GLG1	134.55	66.458	10	9	9	9	1.001	1.027	1.026	1.002	1	1.007	1.007	0.925	1.00	0.99	0.95
O75151	Lysine-specific demethylase PHF2 OS=H	PHF2	120.77	17.727	4.3	4	4	3	0.981	0.885	0.968	0.898	0.945	1.403	0.929	1.152	1.00	1.26	1.12
O00308	NEDD4-like E3 ubiquitin-protein ligase	WWP2	98.911	7.1699	1.5	1	1	1	0.955	0.799	0.67	1.084	1.295	0.826	1.132	1.267	1.00	1.21	1.37
Q96MG7	Non-structural maintenance of chromoso	NSMCE3	34.308	51.975	21.1	5	6	5	0.941	0.985	0.975	0.951	1.041	0.959	1.16	1.013	1.00	1.04	1.13
Q13123	Protein Red OS=Homo sapiens OX=960	IK	65.601	139.19	32.5	13	18	12	1.034	0.96	0.99	1.004	1.022	0.984	0.995	1.003	1.00	1.01	1.00
Q96KQ7	Histone-lysine N-methyltransferase EHM	EHMT2	132.37	34.305	5.3	5	6	5	0.938	1.115	1.047	1.006	0.944	0.921	1.123	1.026	1.00	0.91	1.05
Q9UIG0	Tyrosine-protein kinase BAZ1B OS=Hon	BAZ1B	170.9	222.72	18.6	23	28	23	0.899	0.894	0.836	0.957	1.162	1.121	1.056	1.244	1.00	1.27	1.28
Q9BRT8	COBW domain-containing protein 1 OS=	CBWD1	44.068	14.12	6.1	2	3	2	0.971	1.133	1.074	1.029	0.94	1.187	0.845	0.883	1.00	1.01	0.82
Q9NRH3	Tubulin gamma-2 chain OS=Homo sapien	TUBG2	51.091	78.427	22.2	7	8	7	0.953	1.123	1.074	1.001	0.984	1.023	0.954	0.937	1.00	0.97	0.91
P09001	39S ribosomal protein L3, mitochondrial	MRPL3	38.632	33.074	14.7	5	5	5	1.105	0.938	1.034	1.008	1.046	1.168	0.724	0.922	1.00	1.08	0.81
Q2TAY7	WD40 repeat-containing protein SMU1 (	SMU1	57.543	136.96	25.1	10	15	10	1.017	1.012	1.023	1.005	1.01	1.11	0.878	0.986	1.00	1.04	0.92
Q15233	Non-POU domain-containing octamer-bin	NONO	54.231	182.39	48.4	20	54	18	1	1.012	1.018	0.993	1.014	1.014	1.003	0.983	1.00	1.01	0.99
Q8N543	Prolyl 3-hydroxylase OGFOD1 OS=Hom	OGFOD1	63.245	110.17	22.1	10	10	9	1.033	0.944	0.957	1.019	1.011	1.165	0.877	0.998	1.00	1.10	0.95

Q9UPP1	Histone lysine demethylase PHF8 OS=H	PHF8	117.86	38.301	5.8	4	5	3	0.936	0.999	0.949	0.985	1.052	1.183	0.993	1.133	1.00	1.16	1.10
P35241	Radixin OS=Homo sapiens OX=9606 GI	RDX	68.563	102.48	33.1	22	32	13	1.09	0.949	1.021	1.016	0.967	0.949	1.001	0.957	1.00	0.94	0.96
Q13795	ADP-ribosylation factor-related protein 1	ARFRP1	22.614	12.472	13.4	2	2	2	1.093	0.928	1.086	0.933	0.948	1.063	1.024	0.96	1.00	1.00	0.98
P06744	Glucose-6-phosphate isomerase OS=Hon	GPI	63.146	166.32	33	16	40	15	0.982	1.022	1.02	0.982	0.982	1.154	0.926	0.959	1.00	1.07	0.94
Q3LXA3	Triokinase/FMN cyclase OS=Homo sapi	TKFC	58.946	55.017	15.7	7	8	7	1.041	0.954	0.967	1.026	0.99	0.944	1.032	1.007	1.00	0.97	1.02
P12268	Inosine-5'-monophosphate dehydrogenase	IMPDH2	55.804	218.38	33.9	13	20	12	0.979	0.95	0.906	1.021	0.977	0.88	1.132	1.217	1.00	0.96	1.22
Q6NUK1	Calcium-binding mitochondrial carrier pr	SLC25A24	53.354	99.92	22.6	11	16	11	1.08	0.976	1.082	0.971	0.965	0.89	1.124	1.037	1.00	0.90	1.05
Q13405	39S ribosomal protein L49, mitochondria	MRPL49	19.198	43.669	23.5	3	5	3	1.053	0.998	1.061	0.987	1.044	1.028	0.908	0.869	1.00	1.01	0.87
O43252	Bifunctional 3'-phosphoadenosine 5'-pho	PAPSS1	70.832	131.68	22.3	10	17	10	1.002	0.986	0.955	1.03	1.069	0.968	0.971	1.06	1.00	1.02	1.02
Q02880	DNA topoisomerase 2-beta OS=Homo sa	TOP2B	183.26	167.2	19.5	27	36	20	1.018	0.955	0.965	1.005	1.055	1.183	0.91	1.045	1.00	1.13	0.99
Q86XA9	HEAT repeat-containing protein 5A OS=	HEATR5A	222	37.941	3.5	6	6	6	0.959	0.97	0.909	1.017	1.124	0.91	0.973	0.845	1.00	1.05	0.94
P24941	Cyclin-dependent kinase 2 OS=Homo saj	CDK2	33.929	124.48	45.3	11	19	9	0.948	0.911	0.893	0.963	1.132	1.064	1.061	1.154	1.00	1.18	1.19
O00764	Pyridoxal kinase OS=Homo sapiens OX=	PDXK	35.102	38.438	12.5	3	4	3	1.062	1.107	1.164	1.001	0.915	0.906	0.992	0.859	1.00	0.84	0.85
P61088	Ubiquitin-conjugating enzyme E2 N OS=	UBE2N	17.138	58.453	48.7	6	13	6	1.019	1.036	1.018	1.033	0.92	0.991	0.997	0.973	1.00	0.93	0.96
O43818	U3 small nucleolar RNA-interacting prot	RRP9	51.84	33.811	11.6	5	5	5	1.016	1.002	1.022	0.992	0.995	1.072	0.962	0.95	1.00	1.02	0.95
O60232	Sjoegren syndrome/scleroderma autoanti	SSSCA1	21.474	97.909	55.8	6	10	6	0.967	1.051	0.999	1.015	0.964	0.979	1.049	1.009	1.00	0.96	1.02
Q8ND56	Protein LSM14 homolog A OS=Homo sa	LSM14A	50.529	114.46	16.4	7	10	7	1.001	1.01	1.001	1.006	1.017	1.021	0.979	0.993	1.00	1.01	0.98
Q6NZY4	Zinc finger CCHC domain-containing pr	ZCCHC8	78.576	149.02	27.3	13	14	12	1.001	0.995	1.001	0.991	1.029	1.119	0.958	0.993	1.00	1.08	0.98
Q9UQ80	Proliferation-associated protein 2G4 OS=	PA2G4	43.786	245.65	47.2	17	51	17	0.981	1.011	0.974	1.014	0.983	1.077	0.923	1.008	1.00	1.03	0.97
Q12962	Transcription initiation factor TFIID subu	TAF10	21.711	16.54	15.6	2	2	2	1.047	0.878	0.966	0.955	1.086	0.988	0.972	1.168	1.00	1.08	1.11
P78346	Ribonuclease P protein subunit p30 OS=	RPP30	29.321	21.16	13.1	3	4	3	0.917	0.933	0.894	0.952	1.363	1.115	0.957	1.023	1.00	1.34	1.07
Q9H8X2	Inositol-pentakisphosphate 2-kinase OS=	IPPK	56.016	6.4423	1.6	1	1	1	1.064	0.661	0.66	1.061	1.171	0.778	1.155	1.417	1.00	1.13	1.49
Q4KMQ2	Anoctamin-6 OS=Homo sapiens OX=96(	ANO6	106.16	97.246	13.8	11	10	10	0.986	1.066	0.997	1.05	1.02	0.891	1.106	0.985	1.00	0.93	1.02
Q9Y3A6	Transmembrane emp24 domain-containin	TMED5	26.005	20.704	9.6	2	7	2	1.036	1.012	1.054	0.989	0.968	1.095	0.933	0.979	1.00	1.01	0.93
Q14152	Eukaryotic translation initiation factor 3	EIF3A	166.57	225.08	20.6	28	40	27	0.985	1.038	1.01	1.008	0.996	0.987	0.985	1.005	1.00	0.98	0.98
Q00534	Cyclin-dependent kinase 6 OS=Homo saj	CDK6	36.938	13.417	8.6	3	4	2	1.015	1.003	0.971	1.042	0.978	1.017	0.967	1.024	1.00	0.99	0.99
O15164	Transcription intermediary factor 1-alpha	TRIM24	116.83	23.57	4.2	4	4	3	0.98	1.019	0.978	1.016	1.068	1.21	0.801	0.995	1.00	1.14	0.90
P61106	Ras-related protein Rab-14 OS=Homo sa	RAB14	23.897	57.703	33	7	10	7	1.028	0.954	0.986	0.991	1.059	0.93	1.038	0.976	1.00	1.00	1.02
Q86U44	N6-adenosine-methyltransferase catalytic	METTL3	64.473	79.91	25.2	10	12	10	1.019	0.956	1.017	0.953	1.011	0.943	1.164	1.061	1.00	0.99	1.13
Q9Y6Q9	Nuclear receptor coactivator 3 OS=Homo	NCOA3	155.29	32.171	4.9	5	4	4	0.958	1.01	1.009	0.954	1.02	1.007	1.158	1.008	1.00	1.03	1.10
Q07864	DNA polymerase epsilon catalytic subuni	POLE	261.51	41.215	2.1	4	5	4	1.005	0.92	1.021	0.899	1.154	1.159	0.911	1.063	1.00	1.20	1.03
Q86XI2	Condensin-2 complex subunit G2 OS=H	NCAPG2	130.96	32.414	3.9	5	5	5	0.976	0.919	0.967	0.923	1.168	1.091	0.991	1.067	1.00	1.19	1.09
Q7L099	Protein RUFY3 OS=Homo sapiens OX=	RUFY3	52.964	26.791	10	4	4	4	0.975	1.058	1.031	0.996	0.978	0.935	1.094	1.014	1.00	0.94	1.04
P53992	Protein transport protein Sec24C OS=Ho	SEC24C	118.32	145.09	19.2	14	22	14	1.011	0.997	1.007	0.995	1.018	1.053	0.965	1.009	1.00	1.03	0.98
Q14974	Importin subunit beta-1 OS=Homo sapie	KPNB1	97.169	309.76	29.2	22	51	22	0.981	1.005	1.001	0.979	1.034	0.993	1.026	0.999	1.00	1.02	1.02
Q14146	Unhealthy ribosome biogenesis protein 2	URB2	170.54	75.475	8.3	11	13	11	1.006	0.98	0.992	0.988	1.043	1.08	0.957	0.981	1.00	1.07	0.98
P26641	Elongation factor 1-gamma OS=Homo sa	EEF1G	50.118	134.55	35.5	16	29	16	1.032	1.073	1.087	1.011	0.931	1.047	0.829	0.873	1.00	0.94	0.81
P51946	Cyclin-H OS=Homo sapiens OX=9606 C	CCNH	37.643	25.788	13.3	4	4	4	1.022	0.986	0.957	1.044	0.998	0.973	0.925	0.996	1.00	0.98	0.96
P85037	Forkhead box protein K1 OS=Homo sapi	FOXK1	75.456	40.187	10	6	7	6	1.015	0.968	0.949	1.027	1.04	1.121	0.907	0.956	1.00	1.09	0.94
Q9UK41	Vacuolar protein sorting-associated prote	VPS28	25.425	50.445	30.3	6	8	6	0.983	0.991	0.945	1.022	1.018	0.978	1.162	0.978	1.00	1.01	1.08
Q8WW59	SPRY domain-containing protein 4 OS=	SPRYD4	23.128	15.053	10.1	2	3	2	0.986	0.933	0.865	1.047	1.032	1.031	1.011	1.132	1.00	1.08	1.12
Q5T160	Probable arginine--tRNA ligase, mitocho	RARS2	65.505	20.255	6.2	3	3	3	0.842	0.797	0.649	0.984	1.202	1.347	0.884	1.511	1.00	1.56	1.46
Q9NVI1	Fanconi anemia group I protein OS=Hom	FANCI	149.32	138.04	13	16	21	16	0.946	0.898	0.926	0.911	1.181	1.289	0.998	1.043	1.00	1.34	1.11
Q9Y230	RuvB-like 2 OS=Homo sapiens OX=960	RUVBL2	51.156	163.63	49.9	20	33	19	1.037	1.02	1.015	1.034	0.972	0.931	1.025	0.938	1.00	0.93	0.95
P05198	Eukaryotic translation initiation factor 2	EIF2S1	36.112	183.97	41.9	11	16	11	1.018	1.005	1.002	1.013	1.004	1.007	0.931	1.002	1.00	0.99	0.96
Q8N201	Integrator complex subunit 1 OS=Homo	INTS1	244.29	35.325	2.4	5	6	5	1.009	0.983	0.951	1.033	1.067	1.15	0.842	1.006	1.00	1.11	0.93
Q6ZUM4	Rho GTPase-activating protein 27 OS=H	ARHGAP2	98.395	28.113	5.2	4	5	4	0.929	1.023	0.941	1.003	1.102	0.935	0.863	1.176	1.00	1.04	1.04
Q5RKV6	Exosome complex component MTR3 OS	EXOSC6	28.235	71.7	27.9	6	8	6	0.957	0.981	0.946	0.984	1.089	1.008	1.017	0.965	1.00	1.08	1.02
O43847	Nardilysin OS=Homo sapiens OX=9606	NRDC	131.57	84.677	11.6	12	16	12	1.036	1.041	1.022	1.046	0.988	1.015	0.972	0.913	1.00	0.96	0.91
P07108	Acyl-CoA-binding protein OS=Homo saj	DBI	10.044	99.253	62.1	5	12	5	1.042	1.023	1.07	0.986	1.029	1.169	0.823	0.942	1.00	1.06	0.85
Q8NDF8	Non-canonical poly(A) RNA polymerase	TENT4B	63.266	7.0627	1.6	1	1	1	1.049	1.01	1.018	1.032	0.972	1.161	0.886	0.864	1.00	1.04	0.85
Q8TED0	U3 small nucleolar RNA-associated prot	UTP15	58.414	56.221	16.4	8	11	8	0.978	1.063	1.043	0.989	1.011	1.058	1.008	0.932	1.00	1.01	0.95
A0AVT1	Ubiquitin-like modifier-activating enzym	UBA6	117.97	161.61	18.6	19	25	19	1.05	0.988	1.02	1.009	1.065	0.917	1.014	1.017	1.00	0.97	1.00
Q08379	Golgin subfamily A member 2 OS=Homo	GOLGA2	113.08	187.97	25	18	23	18	1.024	1.011	1.02	1.006	0.951	0.973	0.909	0.994	1.00	0.95	0.94
P49915	GMP synthase [glutamine-hydrolyzing] C	GMPS	76.715	203.72	39.4	22	37	22	0.986	0.974	0.953	0.998	1.031	1.186	0.903	1.019	1.00	1.13	0.98
P18754	Regulator of chromosome condensation C	RCC1	44.969	122.78	24.7	10	16	10	0.941	1.019	0.977	0.974	1.056	1.056	1.013	1.095	1.00	1.08	1.08
Q96T51	RUN and FYVE domain-containing prot	RUFY1	79.817	63.712	13.8	9	12	9	0.954	0.997	0.949	0.993	1.042	0.95	1.085	1.069	1.00	1.02	1.10
Q9UMS4	Pre-mRNA-processing factor 19 OS=Hor	PRPF19	55.18	105.99	32.3	10	19	10	0.949	0.992	0.975	0.957	1.002	1.171	0.928	1.003	1.00	1.12	0.99
P35249	Replication factor C subunit 4 OS=Homo	RFC4	39.681	82.86	31.4	10	13	10	0.929	0.923	0.878	0.965	1.078	1.036	1.084	1.115	1.00	1.14	1.19
Q15648	Mediator of RNA polymerase II transcrip	MED1	168.48	60.363	6.6	8	8	8	0.971	1.04	0.995	1.006	1.014	1.038	1.064	0.961	1.00	1.02	1.01
P08621	U1 small nuclear ribonucleoprotein 70 kI	SNRNP70	51.556	100.85	22.7	11	23	11	0.984	1.022	0.992	1.004	1	1.062	0.935	1.031	1.00	1.03	0.98
P61244	Protein max OS=Homo sapiens OX=960	MAX	18.275	32.698	38.8	5	6	5	0.956	1.045	1.014	0.977	1.076	1.052	1.007	0.992	1.00	1.06	1.00
Q96HQ2	CDKN2AIP N-terminal-like protein OS=	CDKN2AII	13.196	23.941	35.3	3	5	3	1.056	0.942	1.101	0.887	1.074	1.243	0.753	1.052	0.99	1.16	0.90
P82673	28S ribosomal protein S35, mitochondria	MRPS35	36.844	125.44	31.6	7	12	7	1.06	0.938	1	0.988	1.085	1.018	0.916	0.987	0.99	1.05	0.95
Q10567	AP-1 complex subunit beta-1 OS=Homo	AP1B1	104.64	58.119	25	24	39	7	1.011	0.986	0.997	0.99	0.985	1.029	1.001	1.021	0.99	1.01	1.01
P19388	DNA-directed RNA polymerases I, II, and	POLR2E	24.551	19.02	19	3	3	3	1.063	0.929	0.968	1.014	1.094	1.048	0.978	0.897	0.99	1.08	0.94
Q5UIP0	Telomere-associated protein RIF1 OS=H	RIF1	274.46	282.29	15.3	28	36	28	1.019	0.97	0.988	0.991	1.012	0.991	0.983	1.019	0.99	1.01	1.01
Q8WVC0	RNA polymerase-associated protein LEO	LEO1	75.403	21.486	5.3	3	4	3	0.975	0.993	0.984	0.974	1.09	1.101	0.907	1.014	0.99	1.11	0.98
Q13561	Dynactin subunit 2 OS=Homo sapiens O	DCTN2	44.23	147.96	42.4	12	21	12	1.032	1.028	1.045	1.004	0.96	0.989	1.006	0.971	0.99	0.95	0.96
Q9UKL0	REST corepressor 1 OS=Homo sapiens C	RCOR1	53.327	115.66	26.6	9	16	7	1.015	1.002	1.006	1	0.99	0.969	0.937	1.058	0.99	0.97	0.99
Q6P1X6	UPF0598 protein C8orf82 OS=Homo saj	C8orf82	23.889	13.901	10.2	2	3	2	0.93	1.072	0.962	1.029	1.034	1.209	0.828	1.034	0.99	1.12	0.93
Q6PL18	ATPase family AAA domain-containing p	ATAD2	158.55	47.516	5.8	7	7	7	0.894	0.909	0.869	0.924	1.077	1.096	0.838	1.328	0.99	1.21	1.20
Q96GG9	DCN1-like protein 1 OS=Homo sapiens	DCUN1D1	30.124	50.574	26.3	6	7	6	1.018	0.958	1.001	0.964	0.97	0.975	0.953	0.957	0.99	0.98	0.97
Q15036	Sorting nexin-17 OS=Homo sapiens OX=	SNX17	52.901	16.074	4.9	2	2	2	0.982	0.965	0.934	1.002	1.096	0.885	1.162	1.04	0.99	1.02	1.13
O75695	Protein XRP2 OS=Homo sapiens OX=9(	RP2	39.641	32.481	10	5	5	5	0.995	1.07	1.04	1.013	0.994	1.174	0.814	1.012	0.99	1.05	0.88
Q9Y530	O-acetyl-ADP-ribose deacetylase 1 OS=I	OARD1	17.025	29.866	19.7	3	5	3	1.059	0.982	1.02	1.009	1.066	0.903	1.057	0.946	0.99	0.96	0.98
Q6NXE6	Armadillo repeat-containing protein 6 OS	ARMC6	54.141	47.824	14.8	5	6	5	0.919	1.1	0.993	1.014	1.012	1.244	0.929	0.876	0.99	1.12	0.89
Q15633	RISC-loading complex subunit TARBP2	TARBP2	39.039	24.753	12	4	4	4	1.052	0.915	0.978	0.977	0.915	1.044	1.152	1.001	0.99	1.00	1.09
Q969G6	Riboflavin kinase OS=Homo sapiens OX	RFK	17.623	11.505	5.8	2	2	2	0.975	0.992	0.86	1.095	0.872	0.925	1.197	1.092	0.99	0.91	1.16
O75694	Nuclear pore complex protein Nup155 O	NUP155	155.2	199.85	21.8	20	30	20	0.996	0.964	0.977	0.971	1.047	1.015	1.027	0.997	0.99	1.05	1.03
Q9BVC6	Transmembrane protein 109 OS=Homo s	TMEM109	26.21	30.542	9.1	3	5	3	0.904	1.023	0.921	0.994	1.023	1.063	1.114	1.069	0.99	1.08	1.13
P17612	cAMP-dependent protein kinase catalytic	PRKACA	40.589	42.017	19.9	6	7	6	0.934	1.153	1.063	1.011	1	0.938	1.147	1.079	0.99	0.93	1.07
P07195	L-lactate dehydrogenase B chain OS=Hor	LDHB	36.638	191.17	36.8	12	49	11	1.02	1.05	1.026	1.031	0.96	1.062	0.922	0.923	0.99	0.98	0.89
Q12802	A-kinase anchor protein 13 OS=Homo sa	AKAP13	307.55	45.077	3.2	6	7	6	1.017	1.042	1.107	0.939	0.935	0.947	1.118	0.985	0.99	0.91	1.02
Q15291	Retinoblastoma-binding protein 5 OS=H	RBBP5	59.152	41.78	14.1	6	8	6	1.041	1.004	1.042	0.99	1.008	1.021	0.942	0.996	0.99	0.99	0.95
Q96LR5	Ubiquitin-conjugating enzyme E2 E2 OS	UBE2E2	22.255	27.181	21.9	3	6	2	1.013	1.012	1.027	0.985	1.012	0.996	0.938	0.923	0.99	0.99	0.92
Q13416	Origin recognition complex subunit 2 OS	ORC2	65.971	19.173	5.9	3	3	3	1.112	1.061	1.409	0.75	0.725	0.978	1.364	0.751	0.99	0.78	0.97
Q6IAN0	Dehydrogenase/reductase SDR family me	DHRS7B	35.119	20.468	8	2	2	2	1.032	0.967	1.01	0.976	1.1	0.856	1.135	0.968	0.99	0.98	1.05
Q5JPH6	Probable glutamate--tRNA ligase, mitoch	EARS2	58.688	31.824	8.6	4	4	4	1.006	0.992	0.997	0.988	1.036	0.997	0.919	0.936	0.99	1.02	0.93
P07954	Fumarate hydratase, mitochondrial OS=I	FH	54.636	133.36	25.7	9	16	9	1.001	0.988	0.986	0.99	1.021	0.992	0.941	1.07	0.99	1.01	1.01
Q9BTX1	Nucleoporin NDC1 OS=Homo sapiens C	NDC1	76.304	70.281	14.7	6	6	6	0.939	1.039	0.965	1	1.014	0.898	1.072	1.079	0.99	0.97	1.09
Q8TC07	TBC1 domain family member 15 OS=Ho	TBC1D15	79.49	48.779	12.3	8	8	8	1.02	0.954	0.941	1.02	1	0.932	1.11	1.057	0.99	0.98	1.10
P09132	Signal recognition particle 19 kDa protei	SRP19	16.156	46.49	37.5	4	5	3	1.039	0.934	0.984	0.976	1.143	0.992	0.996	1.058	0.99	1.08	1.04
Q13017	Rho GTPase-activating protein 5 OS=Ho	ARHGAP5	172.46	12.915	1.1	2	2	2	1.013	1.094	1.126	0.967	1.087	1.144	0.861	0.776	0.99	1.06	0.78
O75449	Katanin p60 ATPase-containing subunit	KATNA1	55.964	46.659	15.5	7	7	7	1.018	0.932	0.958	0.979	1.027	1.057	0.918	1.05	0.99	1.07	1.01
P57721	Poly(rC)-binding protein 3 OS=Homo sa	PCBP3	39.465	15.994	17.5	5	18	1	1.056	1.006	1.046	1.002	0.972	1.095	0.947	0.885	0.99	1.00	0.89
Q9HD67	Unconventional myosin-X OS=Homo saj	MYO10	237.34	11.638	1.1	2	2	2	0.959	0.943	0.887	1.002	1.12	1.07	0.91	1.207	0.99	1.15	1.11
Q9Y5Z4	Heme-binding protein 2 OS=Homo sapie	HEBP2	22.875	67.831	36.6	7	12	7	1.045	1.003	1.029	1.005	1.017	1.183	0.85	0.917	0.99	1.07	0.86
O95292	Vesicle-associated membrane protein-ass	VAPB	27.228	46.201	26.3	7	13	6	1.052	0.982	0.98	1.04	0.94	1.025	0.948	0.973	0.99	0.97	0.94
P13797	Plastin-3 OS=Homo sapiens OX=9606 C	PLS3	70.81	314.77	50.3	29	56	19	1.009	1.013	1.037	0.971	0.951	0.956	1.074	1.034	0.99	0.94	1.04
Q9H444	Charged multivesicular body protein 4b C	CHMP4B	24.95	47.375	27.2	7	10	7	1.04	0.975	1	1.001	0.991	1.026	0.953	1.024	0.99	1.00	0.98
Q9BUB7	Transmembrane protein 70, mitochondria	TMEM70	28.969	9.2268	4.2	1	1	1	0.888	0.982	0.886	0.971	1.103	1.353	0.928	1.062	0.99	1.31	1.06
Q9NY26	Zinc transporter ZIP1 OS=Homo sapiens	SLC39A1	34.249	37.067	10.2	1	1	1	0.902	1.097	0.962	1.023	1.054	1.122	1.042	0.912	0.99	1.09	0.98
Q9P2R7	Succinate--CoA ligase [ADP-forming] su	SUCLA2	50.317	50.441	17.9	7	8	7	1.012	0.975	0.955	1.018	1.014	1.057	0.891	0.975	0.99	1.04	0.94
Q9Y4C2	TRPM8 channel-associated factor 1 OS=	TCAF1	102.12	27.977	4.7	4	4	4	0.931	0.908	0.866	0.96	1.096	1.039	1.212	1.233	0.99	1.16	1.33
Q9NXF1	Testis-expressed protein 10 OS=Homo sa	TEX10	105.67	105.12	15.5	12	13	12	0.985	0.99	0.981	0.98	1.045	1.208	0.927	1.009	0.99	1.14	0.98
Q8IUD2	ELKS/Rab6-interacting/CAST family me	ERC1	128.08	81.344	11.1	11	13	11	1.014	1.034	1.053	0.98	0.954	0.854	1.13	1.026	0.99	0.88	1.05
Q86VZ5	Phosphatidylcholine:ceramide cholineph	SGMS1	48.616	6.2066	2.2	1	2	1	0.994	1.173	1.076	1.075	0.779	0.735	1.248	0.924	0.99	0.70	1.00
Q9BVG4	Protein PBDC1 OS=Homo sapiens OX=	PBDC1	26.056	78.086	32.6	8	14	8	1.014	1.006	1.068	0.937	0.932	1.127	0.912	0.95	0.99	1.02	0.92
Q9Y3F4	Serine-threonine kinase receptor-associat	STRAP	38.438	144.15	45.1	12	21	12	1.018	1.106	1.091	1.017	0.899	1.109	0.865	0.885	0.99	0.95	0.82
Q9UBL3	Set1/Ash2 histone methyltransferase com	ASH2L	68.722	101.82	21.3	10	13	10	1.02	0.959	0.95	1.014	0.99	1.004	1.042	1.022	0.99	1.01	1.04
Q02447	Transcription factor Sp3 OS=Homo sapie	SP3	81.924	8.249	3.6	1	1	1	0.797	1.274	1.044	1.011	0.92	1.407	0.763	1.005	0.99	1.12	0.85
Q9UL46	Proteasome activator complex subunit 2 (	PSME2	27.401	33.34	24.3	5	6	5	1.055	1.012	1.026	1.025	0.942	0.934	0.97	0.934	0.99	0.91	0.92
Q8TD16	Protein bicaudal D homolog 2 OS=Homo	BICD2	93.532	111.68	20.3	16	20	16	1	1.053	1.03	1.007	1.006	1.017	0.895	0.939	0.99	0.99	0.89
Q96RU3	Formin-binding protein 1 OS=Homo sapi	FNBP1	71.306	12.315	2.8	2	3	2	1.016	1.008	0.993	1.015	0.988	1.07	0.951	0.995	0.99	1.02	0.96
Q5TF39	Sodium-dependent glucose transporter 1	MFSD4B	56.217	6.7604	1.5	1	1	1	1.023	0.994	1.082	0.919	1.007	1.361	0.691	1.036	0.99	1.17	0.86
P63000	Ras-related C3 botulinum toxin substrate	RAC1	21.45	43.258	32.3	6	14	6	1.103	0.91	0.978	1.019	1.037	0.97	0.936	1.196	0.99	1.00	1.06
Q5T5X7	BEN domain-containing protein 3 OS=H	BEND3	94.474	41.01	7	5	6	5	1.025	0.862	1.011	0.861	1.157	1.21	0.976	1.038	0.99	1.25	1.07
Q9Y2W2	WW domain-binding protein 11 OS=Hor	WBP11	69.997	60.284	14	8	11	8	0.985	1.012	0.99	0.991	1.03	1.045	0.952	1.07	0.99	1.04	1.01
O60479	Homeobox protein DLX-3 OS=Homo saj	DLX3	31.738	26.938	10.8	2	2	2	1.08	1.144	1.217	0.989	0.997	1.116	0.779	0.675	0.99	0.95	0.65
P07437	Tubulin beta chain OS=Homo sapiens O	TUBB	49.67	132.38	56.3	19	91	4	1.003	0.972	0.97	0.989	0.96	0.996	1.012	1.077	0.99	0.99	1.06
Q9UBP6	tRNA (guanine-N(7)-)-methyltransferase	METTL1	31.471	15.605	10.5	2	2	2	1.053	0.892	0.932	0.997	1.008	1.216	0.848	1.079	0.99	1.14	0.99
O60671	Cell cycle checkpoint protein RAD1 OS=	RAD1	31.827	14.333	6	1	1	1	0.923	1.02	0.958	0.969	0.963	1.465	0.75	1.11	0.99	1.25	0.96
Q9BYC9	39S ribosomal protein L20, mitochondria	MRPL20	17.442	19.715	20.1	2	3	2	0.992	0.936	0.92	0.992	1.075	0.944	1.125	1.086	0.99	1.05	1.15
Q92696	Geranylgeranyl transferase type-2 subuni	RABGGTA	65.071	100.12	23.6	13	16	13	1.015	1.026	1.009	1.015	0.96	1.028	0.983	0.983	0.99	0.97	0.96
Q16890	Tumor protein D53 OS=Homo sapiens O	TPD52L1	22.449	40.069	18.1	3	4	3	1.087	1.051	1.16	0.96	0.91	0.779	1.232	0.831	0.99	0.79	0.96
P40937	Replication factor C subunit 5 OS=Homo	RFC5	38.496	26.56	8.8	3	4	3	1.026	0.866	0.944	0.932	1.127	0.955	1.273	1.038	0.99	1.10	1.22
P82909	28S ribosomal protein S36, mitochondria	MRPS36	11.466	24.923	41.7	4	4	2	0.986	1.022	1.069	0.922	1.105	1.013	1.117	0.884	0.99	1.05	1.00
Q96EX3	WD repeat-containing protein 34 OS=Ho	WDR34	57.8	36.909	9.7	5	5	5	0.886	0.872	0.8	0.943	1.136	1.171	1.158	1.228	0.99	1.31	1.36
Q8IXU6	Solute carrier family 35 member F2 OS=	SLC35F2	41.211	30.184	9.6	2	2	2	0.995	0.985	0.999	0.964	0.987	0.971	1.124	1.064	0.99	0.99	1.11
Q96KA5	Cleft lip and palate transmembrane protei	CLPTM1L	62.228	50.155	15.4	6	7	6	0.977	0.994	0.966	0.988	1.009	1.005	1.105	1.032	0.99	1.02	1.08
Q5SY16	Polynucleotide 5'-hydroxyl-kinase NOL9	NOL9	79.322	36.446	8.8	5	4	4	1.032	1.013	0.984	1.043	1.033	0.873	1.17	1.054	0.99	0.93	1.09
Q96RL7	Vacuolar protein sorting-associated prote	VPS13A	360.27	32.135	1.6	5	5	5	1.012	1.002	0.995	1.001	1.026	0.957	1.081	0.974	0.99	0.98	1.02
Q9UPY3	Endoribonuclease Dicer OS=Homo sapie	DICER1	218.68	103.38	9.3	14	14	14	1.006	1.003	0.983	1.008	0.988	0.976	1.012	0.934	0.99	0.98	0.97
Q04637	Eukaryotic translation initiation factor 4	EIF4G1	175.49	305.51	21	32	52	31	0.982	1.026	0.986	1.004	0.997	0.979	1.061	0.977	0.99	0.98	1.01
Q14331	Protein FRG1 OS=Homo sapiens OX=9(	FRG1	29.172	41.752	24.4	5	5	4	1.043	1	0.986	1.038	1.005	1.204	0.727	1.001	0.99	1.08	0.85
Q9NPF4	Probable tRNA N6-adenosine threonylca	OSGEP	36.426	48.149	23	6	9	6	0.999	1.043	1.029	0.994	0.959	1.03	1.027	0.918	0.99	0.97	0.95
A1L390	Pleckstrin homology domain-containing f	PLEKHG3	134.41	50.365	7.9	7	8	7	1.055	0.972	1.033	0.975	1.13	0.909	1.096	0.934	0.99	1.01	1.00
Q92947	Glutaryl-CoA dehydrogenase, mitochond	GCDH	48.127	18.873	6.2	2	3	2	0.995	0.977	0.99	0.963	0.95	0.916	1.274	1.022	0.99	0.95	1.16
Q9NQZ2	Something about silencing protein 10 OS	UTP3	54.557	28.049	10	4	6	4	0.951	0.999	0.943	0.988	1.058	1.126	0.974	1.072	0.99	1.12	1.05
Q9BVS5	tRNA (adenine(58)-N(1))-methyltransfera	TRMT61B	52.965	39.688	13	5	5	5	0.98	1.069	1.035	0.994	1.013	1.047	0.973	0.95	0.99	1.01	0.94
P18085	ADP-ribosylation factor 4 OS=Homo sap	ARF4	20.511	95.156	48.3	8	11	4	1.059	0.974	0.991	1.022	0.96	0.919	1.049	1.053	0.99	0.92	1.03
O43684	Mitotic checkpoint protein BUB3 OS=H	BUB3	37.154	97.237	27.4	7	16	7	1.017	0.999	0.979	1.017	1.055	1.048	0.896	1.003	0.99	1.04	0.94
Q14151	Scaffold attachment factor B2 OS=Homo	SAFB2	107.47	238.68	19.8	18	29	12	0.957	0.957	0.91	0.985	1.067	1.152	0.973	1.117	0.99	1.16	1.09
Q6P1K8	General transcription factor IIH subunit 2	GTF2H2C	44.452	41.759	11.1	4	5	4	0.994	1.005	0.972	1.007	1.04	1.047	0.983	1.02	0.99	1.04	1.00
P83111	Serine beta-lactamase-like protein LACT	LACTB	60.693	43.635	15	6	7	6	1.035	1.059	1.069	1.004	0.954	0.979	1.043	0.862	0.99	0.92	0.91
Q9BWJ5	Splicing factor 3B subunit 5 OS=Homo s	SF3B5	10.135	19.998	43	3	3	3	1.093	0.877	0.927	1.023	0.929	0.875	1.063	1.19	0.99	0.92	1.14
O14657	Torsin-1B OS=Homo sapiens OX=9606	TOR1B	37.978	19.963	9.8	3	3	3	0.991	0.976	0.957	0.99	1.108	0.942	1.125	0.978	0.99	1.04	1.07
P49327	Fatty acid synthase OS=Homo sapiens O	FASN	273.42	323.31	38	70	141	70	1.005	1.058	1.046	0.996	1.007	1.238	0.777	0.885	0.99	1.09	0.81
P34059	N-acetylgalactosamine-6-sulfatase OS=H	GALNS	58.025	25.609	7.9	3	5	3	0.959	0.998	0.957	0.98	0.995	0.981	1.169	1.02	0.99	1.01	1.12
Q5VWN6	Protein FAM208B OS=Homo sapiens O	FAM208B	268.84	45.268	3.3	7	7	7	1.045	0.99	1.06	0.954	0.976	1.24	0.843	0.956	0.99	1.09	0.88
Q8N1G2	Cap-specific mRNA (nucleoside-2'-O-)-n	CMTR1	95.32	70.901	9.2	8	9	8	0.982	1.052	0.998	1.015	0.95	0.883	1.149	0.977	0.99	0.90	1.05
Q7L2J0	7SK snRNA methylphosphate capping er	MEPCE	74.354	38.325	9.6	6	7	6	0.919	0.999	0.927	0.971	1.027	1.375	0.793	1.064	0.99	1.25	0.97
Q9BZG8	2-(3-amino-3-carboxypropyl)histidine sy	DPH1	48.805	13.162	7	2	2	2	0.969	1.126	0.995	1.078	0.961	1.114	0.837	0.947	0.99	0.99	0.85
Q9UIA9	Exportin-7 OS=Homo sapiens OX=9606	XPO7	123.91	97.963	16.9	14	17	13	0.966	1.025	0.992	0.978	1.003	1.271	0.861	1.035	0.99	1.14	0.95
Q969F1	General transcription factor 3C polypepti	GTF3C6	24.048	13.562	7.5	2	2	2	1.02	0.966	0.924	1.041	1.027	0.991	0.934	1.115	0.99	1.02	1.03

O15523	ATP-dependent RNA helicase DDX3Y C	DDX3Y	73.153	36.578	40.6	21	36	5	1.036	1.041	1.035	1.02	0.969	1.13	0.922	0.916	0.99	1.01	0.88
Q96GM5	SWI/SNF-related matrix-associated actin	SMARCD1	58.232	50.523	16.5	7	9	6	1.035	0.926	0.973	0.967	1.068	0.979	1.039	1.02	0.99	1.04	1.05
O94913	Pre-mRNA cleavage complex 2 protein P	PCF11	173.05	130.08	8.9	11	11	11	1.007	1.027	0.995	1.017	1.025	0.992	0.852	0.943	0.99	0.99	0.88
P61224	Ras-related protein Rap-1b OS=Homo sa	RAP1B	20.825	83.239	38	8	16	3	1.005	1.02	0.997	1.006	1.031	0.992	1.034	0.995	0.99	1.00	1.00
O75530	Polycomb protein EED OS=Homo sapier	EED	50.197	57.062	19.5	7	9	7	1.009	0.978	0.934	1.031	1.052	1.106	1.022	0.989	0.99	1.09	1.01
Q14203	Dynactin subunit 1 OS=Homo sapiens O	DCTN1	141.69	235.92	20	20	32	20	1.013	1.064	1.014	1.04	0.944	0.939	1.082	0.954	0.99	0.91	0.98
Q9Y5B6	PAX3- and PAX7-binding protein 1 OS=	PAXBP1	104.8	66.845	11.7	9	10	9	1.047	1.013	1.055	0.982	0.999	0.984	0.984	0.948	0.99	0.96	0.94
Q7Z6Z7	E3 ubiquitin-protein ligase HUWE1 OS=	HUWE1	481.89	323.31	10.4	35	48	35	1.036	1.014	0.996	1.031	0.972	0.957	0.967	0.983	0.99	0.94	0.95
Q7Z392	Trafficking protein particle complex subu	TRAPPC11	128.88	38.017	5.2	5	5	5	0.985	1.059	1.059	0.962	0.997	1.237	0.791	1.019	0.99	1.09	0.89
Q14CX7	N-alpha-acetyltransferase 25, NatB auxili	NAA25	112.29	85.644	14.8	12	12	12	1.009	1.012	0.971	1.027	0.993	0.946	1.019	0.993	0.99	0.96	1.00
Q92530	Proteasome inhibitor PI31 subunit OS=H	PSMF1	29.816	41.602	14.4	3	4	3	0.922	1.362	1.275	0.983	0.76	1.38	0.725	0.732	0.99	0.94	0.64
Q9NX63	MICOS complex subunit MIC19 OS=Ho	CHCHD3	26.152	47.062	28.2	6	8	6	1.017	0.989	0.982	1.001	1.011	0.987	1	1.042	0.99	1.00	1.02
P62995	Transformer-2 protein homolog beta OS=	TRA2B	33.665	48.544	24.7	5	8	5	0.98	1.008	1.014	0.951	1.033	1.006	1.091	1.02	0.99	1.03	1.06
P62942	Peptidyl-prolyl cis-trans isomerase FKBF	FKBP1A	11.951	31.568	25	2	7	2	1.054	1.095	1.107	1.017	0.814	0.994	0.954	0.908	0.99	0.84	0.87
Q9HBL8	NmrA-like family domain-containing pro	NMRAL1	33.344	34.178	18.1	5	5	5	1.029	0.94	0.931	1.015	1.097	0.881	1.032	0.948	0.99	1.00	1.01
Q9UEY8	Gamma-adducin OS=Homo sapiens OX=	ADD3	79.154	23.546	6.2	4	4	4	1.275	1.016	1.081	1.183	0.831	0.779	0.627	0.919	0.99	0.70	0.67
Q99640	Membrane-associated tyrosine- and threo	PKMYT1	54.521	36.087	14.4	5	7	5	1.029	0.922	0.954	0.974	1.046	1.168	0.977	0.984	0.99	1.13	1.01
O43747	AP-1 complex subunit gamma-1 OS=Hor	AP1G1	91.35	105.33	19.1	12	19	12	1.021	1.012	1.005	1.004	1.022	0.994	1.029	0.963	0.99	0.99	0.98
P47755	F-actin-capping protein subunit alpha-2 (	CAPZA2	32.949	119.07	51	10	14	8	0.997	1.035	0.97	1.038	0.981	0.936	0.995	1.005	0.99	0.94	0.98
Q14165	Malectin OS=Homo sapiens OX=9606 G	MLEC	32.233	48.457	21.6	5	8	5	1.061	0.968	1.024	0.981	1.025	0.933	1.089	0.987	0.99	0.97	1.02
Q16698	2,4-dienoyl-CoA reductase, mitochondria	DECR1	36.067	44.482	26.6	7	8	7	0.972	1.053	1.033	0.968	0.921	1.041	1.022	1.022	0.99	0.97	1.01
Q9BZI7	Regulator of nonsense transcripts 3B OS	UPF3B	57.761	68.506	19.7	8	9	8	0.983	1.036	0.971	1.024	0.961	1.021	1.009	1.015	0.99	0.98	1.00
Q9P107	GEM-interacting protein OS=Homo sapi	GMIP	106.68	7.8576	1.3	1	1	1	1.047	0.988	0.931	1.079	1.006	0.927	0.819	1.177	0.99	0.95	0.98
P52298	Nuclear cap-binding protein subunit 2 OS	NCBP2	18.001	25	15.4	2	5	2	1.092	0.936	0.975	1.028	1.08	0.963	0.99	0.99	0.99	1.01	0.98
Q9UEE5	Serine/threonine-protein kinase 17A OS=	STK17A	46.558	14.669	6.3	2	2	2	1.054	1.049	1.087	0.99	1.031	0.86	1.018	0.931	0.99	0.90	0.93
Q92783	Signal transducing adapter molecule 1 OS	STAM	59.179	37.779	10.6	5	6	5	1.02	0.996	0.972	1.019	0.995	1.123	0.935	1.045	0.99	1.05	0.98
P49914	5-formyltetrahydrofolate cyclo-ligase OS	MTHFS	23.255	27.006	21.2	4	4	4	0.981	1.033	1.012	0.977	1.11	1.143	0.915	0.918	0.99	1.12	0.91
Q9NZ32	Actin-related protein 10 OS=Homo sapie	ACTR10	46.306	33.991	17.3	5	6	5	1.054	1.08	1.036	1.071	1.013	0.972	0.886	1	0.99	0.93	0.88
P61006	Ras-related protein Rab-8A OS=Homo sa	RAB8A	23.668	61.465	36.2	7	13	5	0.997	0.975	0.977	0.97	1.051	1.235	0.897	0.986	0.99	1.16	0.95
Q9H078	Caseinolytic peptidase B protein homolo	CLPB	78.728	136.92	17.3	9	10	9	0.997	0.973	0.955	0.99	1.114	0.981	0.959	1.008	0.99	1.06	1.00
O95299	NADH dehydrogenase [ubiquinone] 1 alp	NDUFA10	40.75	80.207	24.2	11	15	11	1.003	1.045	1.031	0.991	1.005	1.155	0.869	0.953	0.99	1.05	0.89
Q96EE3	Nucleoporin SEH1 OS=Homo sapiens O	SEH1L	39.648	148.28	38.6	10	13	9	1.014	1.033	1.046	0.975	1.002	1.188	0.965	0.921	0.99	1.07	0.92
O15212	Prefoldin subunit 6 OS=Homo sapiens O	PFDN6	14.582	31.816	23.3	4	8	4	0.995	1.034	1.032	0.971	1.089	0.909	1.012	0.991	0.99	0.98	0.99
Q9BSL1	Ubiquitin-associated domain-containing	UBAC1	45.338	13.929	5.2	2	2	2	0.966	1.293	1.147	1.083	0.727	1.097	0.836	0.883	0.99	0.81	0.76
P49916	DNA ligase 3 OS=Homo sapiens OX=96	LIG3	112.91	140.67	17.7	15	18	15	1.012	0.969	0.968	0.987	1.043	0.979	1.078	1.048	0.99	1.02	1.07
Q15424	Scaffold attachment factor B1 OS=Homo	SAFB	102.64	134.96	20	17	26	11	0.994	0.982	0.971	0.979	0.998	1.005	1.035	1	0.99	1.01	1.03
Q8WYA6	Beta-catenin-like protein 1 OS=Homo sa	CTNNBL1	65.173	103.84	23.4	11	15	11	1.009	0.966	0.953	0.996	0.988	1.124	0.884	1.054	0.99	1.07	0.98
Q9Y295	Developmentally-regulated GTP-binding	DRG1	40.542	122.38	44.7	14	22	14	1.016	1.029	0.998	1.02	0.97	1.157	0.807	0.932	0.99	1.04	0.85
Q9Y624	Junctional adhesion molecule A OS=Hor	F11R	32.583	81.461	22.7	5	11	5	0.998	1.041	0.991	1.021	0.942	0.897	1.144	1.022	0.99	0.90	1.06
P11498	Pyruvate carboxylase, mitochondrial OS=	PC	129.63	119.09	14.8	13	15	13	1.006	0.943	0.967	0.956	1.089	1.102	0.916	0.955	0.99	1.12	0.96
Q8N3P4	Vacuolar protein sorting-associated prote	VPS8	161.75	33.755	3.9	5	5	5	1.111	0.985	1.071	0.997	1.017	0.747	1.253	0.777	0.99	0.84	0.97
Q9Y6D9	Mitotic spindle assembly checkpoint prot	MAD1L1	83.066	99.28	22.1	13	15	13	0.978	0.985	0.968	0.968	1.046	1.186	0.899	1.05	0.99	1.14	0.99
Q00688	Peptidyl-prolyl cis-trans isomerase FKBF	FKBP3	25.177	83.335	45.1	9	13	9	1.024	1.083	1.062	1.016	0.917	1.157	0.818	0.963	0.99	0.98	0.85
Q5VZ03	Nucleoredoxin-like protein 2 OS=Homo	NXNL2	17.614	10.402	14.7	2	2	2	1.113	0.842	0.9	1.028	1.035	0.874	1.191	0.956	0.99	0.98	1.10
Q9Y3B8	Oligoribonuclease, mitochondrial OS=Ho	REXO2	26.832	36.557	20.7	5	8	5	1.018	1.069	1.049	1.009	0.945	0.996	0.959	0.952	0.99	0.93	0.92
Q9P2I0	Cleavage and polyadenylation specificity	CPSF2	88.486	80.527	14.7	9	12	9	0.984	1.015	1.037	0.934	0.992	1.071	0.883	0.965	0.99	1.03	0.92
Q6PJT7	Zinc finger CCCH domain-containing pro	ZC3H14	82.875	72.275	18.2	11	11	10	1.012	0.979	1.028	0.935	1.09	0.958	1.028	1.041	0.99	1.03	1.04
Q9UHN6	Cell surface hyaluronidase OS=Homo sap	TMEM2	154.37	120.09	11.8	14	15	14	0.985	1.004	0.974	0.987	1.029	0.966	1.04	1.067	0.99	1.00	1.06
Q8WXD5	Gem-associated protein 6 OS=Homo sapi	GEMIN6	18.824	8.1899	7.8	1	1	1	1.01	1.016	0.954	1.043	0.982	1.038	1.061	0.91	0.99	1.00	0.97
O15355	Protein phosphatase 1G OS=Homo sapie	PPM1G	59.271	150.04	30.8	14	23	13	1.041	0.983	1.014	0.981	0.986	0.951	0.979	0.977	0.99	0.96	0.97
Q8IVT5	Kinase suppressor of Ras 1 OS=Homo sa	KSR1	102.16	68.128	9.8	7	8	6	0.99	1.033	1.055	0.939	1.035	0.85	1.14	0.986	0.99	0.93	1.05
Q92688	Acidic leucine-rich nuclear phosphoprote	ANP32B	28.787	30.875	24.3	5	24	3	1	0.99	0.973	0.988	0.958	1.096	0.923	0.982	0.99	1.03	0.96
Q9Y5Q8	General transcription factor 3C polypepti	GTF3C5	59.57	57.335	20	9	10	9	1.052	1.001	1.047	0.976	1.037	1.236	0.768	0.922	0.99	1.11	0.82
Q96SB8	Structural maintenance of chromosomes	SMC6	126.32	20.608	3.9	3	3	3	1.005	0.977	0.96	0.993	1.028	1.076	1.019	1.002	0.99	1.06	1.02
Q15428	Splicing factor 3A subunit 2 OS=Homo s	SF3A2	49.255	74.744	14.2	6	8	6	1.079	0.963	0.981	1.031	1.055	1.095	0.834	1.014	0.99	1.05	0.90
P61158	Actin-related protein 3 OS=Homo sapien	ACTR3	47.371	160.17	42.8	15	20	15	1.059	1.049	1.062	1.015	0.954	0.94	0.975	0.946	0.99	0.90	0.91
Q6P1J9	Parafibromin OS=Homo sapiens OX=960	CDC73	60.576	94.856	22.6	13	15	13	1.017	0.982	0.989	0.98	1.066	0.975	1.004	1	0.98	1.02	1.00
O00399	Dynactin subunit 6 OS=Homo sapiens O	DCTN6	20.747	11.598	8.9	2	2	2	1.011	0.854	0.936	0.901	1.389	1.06	0.989	0.974	0.98	1.31	1.05
O14530	Thioredoxin domain-containing protein 9	TXNDC9	26.534	46.601	24.8	3	4	3	0.948	1.087	1.021	0.983	1.009	0.99	1.041	1.034	0.98	0.98	1.02
Q01970	1-phosphatidylinositol 4,5-bisphosphate	PLCB3	138.8	45.012	6.2	6	9	6	1.01	1.024	0.974	1.029	1.023	0.926	1.097	0.934	0.98	0.96	1.00
Q969H8	Myeloid-derived growth factor OS=Homo	MYDGF	18.795	57.603	27.2	4	8	4	0.996	1.101	1.041	1.024	0.943	1.15	0.863	0.884	0.98	1.00	0.83
Q03154	Aminoacylase-1 OS=Homo sapiens OX=	ACY1	45.884	27.856	13.5	4	7	4	1.105	0.851	0.856	1.07	0.973	0.827	1.084	0.954	0.98	0.92	1.04
Q86WR0	Coiled-coil domain-containing protein 25	CCDC25	24.479	27.761	21.2	5	4	4	1.069	1.077	1.043	1.07	0.955	0.839	1.037	0.92	0.98	0.84	0.91
Q8NC42	E3 ubiquitin-protein ligase RNF149 OS=	RNF149	43.164	34.527	15.2	4	4	4	0.947	0.972	0.91	0.979	0.939	0.962	1.223	0.998	0.98	0.99	1.16
Q8NCL4	Polypeptide N-acetylgalactosaminyltrans	GALNT6	71.158	43.192	12.9	6	6	5	1.052	0.855	0.858	1.019	1.069	0.924	1.02	1.212	0.98	1.05	1.17
Q99733	Nucleosome assembly protein 1-like 4 OS	NAP1L4	42.823	79.11	23.7	7	11	7	1.024	1.005	0.991	1.006	0.981	0.981	1.013	0.994	0.98	0.97	0.99
Q16718	NADH dehydrogenase [ubiquinone] 1 alp	NDUFA5	13.459	22.323	29.3	3	6	3	1	0.95	0.937	0.982	1.078	1.039	1.041	1.193	0.98	1.09	1.15
O60888	Protein CutA OS=Homo sapiens OX=96	CUTA	19.116	24.179	15.6	2	5	2	1.015	0.99	0.947	1.026	1.067	0.986	0.944	1.042	0.98	1.02	0.99
Q6PD62	RNA polymerase-associated protein CTR	CTR9	133.5	95.144	11.1	11	11	10	1.028	0.974	1.007	0.963	0.964	1.018	0.959	1.003	0.98	0.99	0.98
Q9Y676	28S ribosomal protein S18b, mitochondri	MRPS18B	29.395	26.351	19	3	6	3	0.973	1.025	1.004	0.962	0.961	1.259	0.903	0.89	0.98	1.11	0.90
Q96E11	Ribosome-recycling factor, mitochondria	MRRF	29.277	43.969	24.8	5	5	5	1.055	0.994	1.032	0.984	1.091	1.132	0.843	0.926	0.98	1.08	0.86
P48735	Isocitrate dehydrogenase [NADP], mitoch	IDH2	50.909	181.98	39.2	15	30	15	0.968	0.938	0.919	0.956	1.127	1.24	0.917	1.108	0.98	1.24	1.06
P60660	Myosin light polypeptide 6 OS=Homo sa	MYL6	16.93	111	47.7	7	22	5	1.048	0.96	0.998	0.977	1.014	0.901	1.141	1.055	0.98	0.95	1.09
P33151	Cadherin-5 OS=Homo sapiens OX=9606	CDH5	87.527	6.7221	1.4	1	1	1	0.973	1.077	1.054	0.962	1.07	0.918	1.006	1.05	0.98	0.97	1.00
Q92925	SWI/SNF-related matrix-associated actin	SMARCD2	58.92	99.086	21.7	9	12	8	1.019	1.028	1.022	0.991	0.981	1.105	0.913	0.992	0.98	1.02	0.93
Q96EY4	Translation machinery-associated protein	TMA16	23.864	31.139	25.6	5	6	5	1.115	0.989	1.082	0.987	1.068	1.041	0.824	0.981	0.98	1.00	0.86
Q15021	Condensin complex subunit 1 OS=Homo	NCAPD2	157.18	234.83	21.3	24	32	22	1.061	1.085	1.073	1.037	0.96	1.077	0.857	0.877	0.98	0.95	0.81
Q9H0D6	5'-3' exoribonuclease 2 OS=Homo sapien	XRN2	108.58	127.25	13.7	12	18	12	1.056	0.91	0.956	0.977	1.103	0.972	0.98	1.028	0.98	1.06	1.02
O14492	SH2B adapter protein 2 OS=Homo sapie	SH2B2	67.737	9.4013	3.2	1	1	1	0.993	0.958	0.94	0.978	1.055	0.881	1.144	1.132	0.98	0.99	1.17
O95793	Double-stranded RNA-binding protein St	STAU1	63.182	154.75	30	14	20	12	1.011	0.996	0.987	0.986	1.066	0.986	0.991	1.005	0.98	1.02	0.99
Q13217	DnaJ homolog subfamily C member 3 OS	DNAJC3	57.579	100.98	20.6	10	14	10	0.979	1.052	1.001	0.995	1.024	1.208	0.908	0.926	0.98	1.10	0.90
P06454	Prothymosin alpha OS=Homo sapiens OX	PTMA	12.203	13.848	22.5	2	5	2	1.069	1.072	1.139	0.965	0.901	0.958	0.931	0.784	0.98	0.87	0.80
P55072	Transitional endoplasmic reticulum ATPa	VCP	89.321	311.64	51.9	30	65	29	1.006	1.005	0.986	0.99	1.001	0.98	0.991	0.989	0.98	0.99	0.98
P50990	T-complex protein 1 subunit theta OS=H	CCT8	59.62	314.72	50.5	28	58	28	1.024	1.044	1.032	1	0.977	1.018	0.953	0.95	0.98	0.96	0.92
P11441	Ubiquitin-like protein 4A OS=Homo sap	UBL4A	17.776	20.218	26.1	3	3	3	1.088	0.98	1.046	0.986	0.954	0.933	0.956	0.907	0.98	0.91	0.90
P36405	ADP-ribosylation factor-like protein 3 OS	ARL3	20.455	35.827	37.4	5	7	5	1.081	1.09	1.037	1.096	0.963	0.926	0.998	0.954	0.98	0.87	0.90
P33176	Kinesin-1 heavy chain OS=Homo sapiens	KIF5B	109.68	323.31	43.1	34	50	34	1.036	1.069	1.031	1.037	0.936	0.938	0.982	0.948	0.98	0.89	0.92
Q96FF9	Sororin OS=Homo sapiens OX=9606 GN	CDCA5	27.6	12.587	9.5	2	2	2	0.883	0.589	0.603	0.843	1.434	1.181	1.242	1.496	0.98	1.78	1.86
Q92993	Histone acetyltransferase KAT5 OS=Hom	KAT5	58.581	12.281	3.3	2	2	2	0.997	1.039	0.988	1.012	0.947	0.948	1.059	1.068	0.98	0.93	1.04
Q13404	Ubiquitin-conjugating enzyme E2 variant	UBE2V1	16.495	23.131	32.7	4	5	1	1.036	1.054	1.007	1.046	1.008	0.865	1.008	0.969	0.98	0.90	0.95
Q9BSF8	BTB/POZ domain-containing protein 10	BTBD10	53.779	8.2878	3.2	1	1	1	1.005	0.969	0.912	1.027	1.089	0.904	1.036	1.093	0.98	1.01	1.08
Q8TDX7	Serine/threonine-protein kinase Nek7 OS	NEK7	34.551	39.81	19.5	6	8	6	0.962	1.066	1.031	0.961	0.957	1.244	0.766	0.894	0.98	1.09	0.82
Q9Y6K1	DNA (cytosine-5)-methyltransferase 3A (	DNMT3A	101.86	14.288	3.5	3	4	2	1.031	0.88	0.902	0.975	1.157	1.121	0.949	1.039	0.98	1.19	1.04
Q13427	Peptidyl-prolyl cis-trans isomerase G OS	PPIG	88.616	66.398	12.1	8	9	7	0.974	1.045	0.933	1.05	0.964	1.135	0.847	1.077	0.98	1.04	0.95
Q12874	Splicing factor 3A subunit 3 OS=Homo s	SF3A3	58.848	82.627	20.6	8	13	8	0.998	1.002	0.951	1.013	1.051	1.083	0.948	0.967	0.98	1.07	0.96
Q9Y296	Trafficking protein particle complex subu	TRAPPC4	24.34	38.689	30.6	5	7	5	1.015	0.982	1.009	0.952	0.973	0.967	1.181	0.997	0.98	0.97	1.09
Q9H270	Vacuolar protein sorting-associated prote	VPS11	107.84	21.161	4	3	3	3	1.002	0.88	0.882	0.966	1.102	0.894	1.173	1.183	0.98	1.06	1.25
Q8NHH9	Atlastin-2 OS=Homo sapiens OX=9606 (	ATL2	66.228	49.276	13.2	5	5	5	0.999	1.032	1.019	0.975	0.982	1.031	0.944	1	0.98	0.99	0.96
Q06265	Exosome complex component RRP45 OS	EXOSC9	48.948	51.958	18	6	9	6	0.993	0.981	0.93	1.008	1.026	1.192	0.929	1.046	0.98	1.12	1.00
Q99487	Platelet-activating factor acetylhydrolase	PAFAH2	44.035	6.8284	2.3	1	1	1	1.014	1.062	1.073	0.965	1.012	1.152	0.9	0.895	0.98	1.04	0.86
Q9NVG8	TBC1 domain family member 13 OS=Ho	TBC1D13	46.553	77.088	24.8	9	11	9	0.996	1.018	0.935	1.042	0.978	0.995	0.991	0.977	0.98	0.98	0.98
P09874	Poly [ADP-ribose] polymerase 1 OS=Hor	PARP1	113.08	323.31	32.1	33	47	33	0.963	0.941	0.895	0.974	1.09	1.079	1.01	1.136	0.98	1.14	1.13
Q92560	Ubiquitin carboxyl-terminal hydrolase BA	BAP1	80.361	7.0399	1.5	1	1	1	1.06	0.929	0.944	1.008	1.044	1.209	0.689	1.135	0.98	1.13	0.92
Q9C0B1	Alpha-ketoglutarate-dependent dioxygena	FTO	58.281	96.746	29.7	14	14	14	1.003	0.985	0.951	1	1.048	0.943	1.122	1.009	0.98	1.00	1.07
Q8TB61	Adenosine 3'-phospho 5'-phosphosulfate	SLC35B2	47.514	48.538	14.8	7	7	7	0.969	1.018	0.953	0.997	1.038	1.183	0.82	1.04	0.98	1.12	0.94
P04179	Superoxide dismutase [Mn], mitochondri	SOD2	24.75	19.396	13.1	2	2	2	1.146	0.838	0.977	0.97	1.256	1.069	0.766	0.939	0.98	1.17	0.86
Q9BY42	Protein RTF2 homolog OS=Homo sapien	RTFDC1	33.886	31.664	19.3	4	5	4	1.049	0.934	0.92	1.026	1.071	1.03	0.939	1.032	0.98	1.06	0.99
Q8WVT3	Trafficking protein particle complex subu	TRAPPC12	79.374	49.077	15.4	7	7	7	1.02	0.951	0.95	0.984	1.087	0.97	1.007	0.99	0.98	1.04	1.01
Q6ZSR9	Uncharacterized protein FLJ45252 OS=H	---	37.976	65.179	32.1	7	8	7	0.961	1.008	1.02	0.912	0.876	0.948	1.054	1.124	0.98	0.93	1.11
Q14534	Squalene monooxygenase OS=Homo sap	SQLE	63.922	12.731	5.4	2	2	2	0.947	0.912	0.85	0.974	1.057	1.184	1.098	1.1	0.98	1.21	1.18
Q9UJX4	Anaphase-promoting complex subunit 5 (	ANAPC5	85.076	27.164	5.4	3	4	3	0.999	1.069	0.977	1.052	0.944	0.941	1.006	1.035	0.98	0.91	0.99
P49281	Natural resistance-associated macrophage	SLC11A2	62.265	8.408	1.9	1	2	1	0.98	1.133	1.078	0.995	0.823	1.188	0.898	0.995	0.98	0.95	0.90
Q9Y276	Mitochondrial chaperone BCS1 OS=Hor	BCS1L	47.534	86.099	16.7	6	7	6	1.004	0.999	0.977	0.988	1.033	1.015	0.982	1.039	0.98	1.02	1.01
O76071	Probable cytosolic iron-sulfur protein ass	CIAO1	37.84	64.538	28	8	12	8	0.981	1.126	1.026	1.041	0.839	1.017	1.091	0.975	0.98	0.88	0.98
P54278	Mismatch repair endonuclease PMS2 OS	PMS2	95.796	15.914	2.9	2	2	2	0.998	1.046	0.957	1.048	0.968	0.986	1.017	1.007	0.98	0.96	0.99
Q13129	Zinc finger protein Rlf OS=Homo sapien	RLF	217.95	14.974	1.3	2	2	2	1.014	0.964	1.061	0.879	1.144	1.006	1.063	1.005	0.98	1.09	1.05
Q96EY8	Cob(I)yrinic acid a,c-diamide adenosyltra	MMAB	27.388	35.324	22.4	4	4	4	1.002	0.915	0.94	0.94	1.256	1.312	0.79	0.928	0.98	1.34	0.90
O60256	Phosphoribosyl pyrophosphate synthase-	PRPSAP2	40.925	35.205	10	3	3	3	1.05	1.022	1.064	0.968	0.995	0.931	1.034	0.932	0.98	0.93	0.95
Q99805	Transmembrane 9 superfamily member 2	TM9SF2	75.775	73.752	11.6	7	10	7	1.039	1.016	1.004	1.011	1.005	1.013	0.972	0.916	0.98	0.98	0.92
Q9UNZ2	NSFL1 cofactor p47 OS=Homo sapiens (	NSFL1C	40.572	135.74	45.7	15	22	15	1.036	1.019	1.013	1.002	0.932	0.928	0.979	1.033	0.98	0.91	0.98
Q96N67	Dedicator of cytokinesis protein 7 OS=H	DOCK7	242.56	114.59	7.5	15	17	14	0.999	1.053	1.002	1.01	0.966	1.016	1.067	0.931	0.98	0.97	0.97
P14649	Myosin light chain 6B OS=Homo sapiens	MYL6B	22.764	19.027	30.3	5	10	3	1.007	1.079	1.008	1.037	0.959	0.978	1.002	0.956	0.98	0.93	0.94
Q9Y2S7	Polymerase delta-interacting protein 2 OS	POLDIP2	42.033	62.342	23.1	8	9	8	1.034	0.944	0.953	0.986	1.047	1.15	0.871	1.045	0.98	1.11	0.97
Q96FW1	Ubiquitin thioesterase OTUB1 OS=Homo	OTUB1	31.284	130.81	47.2	11	19	11	0.991	1.074	1	1.024	0.937	1.077	0.961	0.993	0.98	0.98	0.95
P40938	Replication factor C subunit 3 OS=Homo	RFC3	40.556	98.316	30.3	9	12	9	1.022	0.941	0.971	0.953	1.104	1.076	0.989	1.01	0.98	1.11	1.02
O75312	Zinc finger protein ZPR1 OS=Homo sapi	ZPR1	50.925	47.61	15.7	6	8	6	1.054	1	1.015	0.998	1.037	0.976	1.079	0.941	0.98	0.98	0.98
Q9Y4E8	Ubiquitin carboxyl-terminal hydrolase 15	USP15	112.42	142.84	17.6	17	20	15	1.031	1.071	1.044	1.016	0.949	1.078	0.895	0.93	0.98	0.96	0.87
P68036	Ubiquitin-conjugating enzyme E2 L3 OS	UBE2L3	17.861	39.764	36.4	5	7	5	1.047	1.053	0.971	1.087	0.899	1.034	0.811	0.97	0.98	0.92	0.85
P11234	Ras-related protein Ral-B OS=Homo sap	RALB	23.408	15.15	16.5	4	5	2	0.958	0.789	0.688	1.024	1.118	0.963	1.134	1.412	0.98	1.19	1.46
Q6UB99	Ankyrin repeat domain-containing protein	ANKRD11	297.91	12.043	1.1	2	2	2	1.004	0.887	0.807	1.046	1.062	1.054	0.92	1.251	0.98	1.12	1.15
O75489	NADH dehydrogenase [ubiquinone] iron-	NDUFS3	30.241	102.6	45.5	10	19	10	0.994	0.985	0.939	1	1.006	1.01	0.966	1.021	0.98	1.02	1.00
P09429	High mobility group protein B1 OS=Hom	HMGB1	24.893	106.54	40	10	19	7	0.985	1.041	0.976	1.009	0.964	1.108	0.921	1.053	0.98	1.02	0.97
Q9Y5M8	Signal recognition particle receptor subu	SRPRB	29.702	137.63	40.2	9	16	9	1.012	0.953	0.951	0.974	1.1	1.107	0.908	1.018	0.98	1.12	0.98
P48643	T-complex protein 1 subunit epsilon OS=	CCT5	59.67	225.13	37	22	42	22	1.011	1.052	1.013	1.008	0.997	1.107	0.909	0.961	0.98	1.02	0.91
Q9Y490	Talin-1 OS=Homo sapiens OX=9606 GN	TLN1	269.76	323.31	42.8	80	125	68	1.002	1.074	1.023	1.01	0.918	0.964	1.037	0.988	0.98	0.91	0.98
Q01844	RNA-binding protein EWS OS=Homo sa	EWSR1	68.477	81.747	14	7	12	7	0.975	1.1	1.026	1.006	0.979	1.195	0.771	0.953	0.98	1.05	0.83
P15848	Arylsulfatase B OS=Homo sapiens OX=9	ARSB	59.687	29.77	7.3	3	3	3	0.978	0.948	0.83	1.056	1.097	0.879	1.108	1.139	0.98	1.03	1.17
P15313	V-type proton ATPase subunit B, kidney	ATP6V1B1	56.832	227.17	46	20	31	15	0.999	1.06	1.016	1	0.984	1.014	1.072	1.027	0.98	0.97	1.02
Q6IQ49	Replication stress response regulator SDI	SDE2	49.741	6.8277	2.2	1	1	1	1.015	1.029	1.029	0.972	0.924	0.994	1.166	0.936	0.98	0.94	1.03
P12074	Cytochrome c oxidase subunit 6A1, mito	COX6A1	12.155	51.995	26.6	1	2	1	0.957	1.213	1.141	0.983	0.822	1.354	0.833	0.806	0.98	1.00	0.76
Q9Y6B6	GTP-binding protein SAR1b OS=Homo s	SAR1B	22.41	25.081	34.8	7	13	3	1.003	1.061	1.034	0.986	0.984	0.989	1.029	0.916	0.98	0.96	0.94
O75182	Paired amphipathic helix protein Sin3b C	SIN3B	133.06	57.276	9.6	9	10	8	1.01	1.005	1.005	0.967	1.002	0.926	1.061	1.014	0.98	0.96	1.03
O14776	Transcription elongation regulator 1 OS=	TCERG1	123.9	159.9	15.3	19	31	19	0.991	1.024	0.968	1.004	1.016	0.982	1.046	1.055	0.98	0.99	1.04
Q9BYE9	Cadherin-related family member 2 OS=H	CDHR2	141.54	6.8462	1.1	1	1	1	1.036	1.114	1.022	1.082	0.939	1.124	0.86	0.789	0.98	0.96	0.77
Q8TD19	Serine/threonine-protein kinase Nek9 OS	NEK9	107.17	86.559	15.5	12	13	12	1.041	1.014	0.969	1.042	0.972	0.932	1.001	0.93	0.98	0.93	0.94
Q9Y613	FH1/FH2 domain-containing protein 1 O	FHOD1	126.55	144.19	16.4	14	21	14	1.022	1.025	1.002	1.001	1.041	0.961	0.975	0.988	0.98	0.98	0.96
O43169	Cytochrome b5 type B OS=Homo sapiens	CYB5B	16.332	24.549	31.5	2	3	2	0.999	1.047	1.019	0.983	0.98	1.14	0.806	1.051	0.98	1.04	0.91
Q9UHR4	Brain-specific angiogenesis inhibitor 1-as	BAIAP2L1	56.882	93.901	21.1	9	12	9	1.023	1.067	0.972	1.073	0.955	0.963	1.011	0.974	0.98	0.92	0.95
Q9BRQ6	MICOS complex subunit MIC25 OS=Ho	CHCHD6	26.457	19.11	15.7	3	3	3	0.944	1.083	0.991	0.992	0.933	1.147	0.974	1.053	0.98	1.03	1.00
Q96QU8	Exportin-6 OS=Homo sapiens OX=9606	XPO6	128.88	16.176	2.5	2	2	2	1.047	1.019	0.988	1.033	0.97	0.924	1.154	0.852	0.98	0.92	0.97
P13861	cAMP-dependent protein kinase type II-a	PRKAR2A	45.518	69.869	26	9	12	9	1.038	0.927	0.933	0.989	1.014	0.934	1.099	1.01	0.98	0.99	1.07
O60884	DnaJ homolog subfamily A member 2 OS	DNAJA2	45.745	87.356	34.7	11	20	10	1.036	0.973	0.985	0.98	1.019	0.979	1.113	0.999	0.98	0.99	1.05
Q9Y2A7	Nck-associated protein 1 OS=Homo sapi	NCKAP1	128.79	171.13	21.5	22	35	22	1.032	0.973	0.977	0.984	0.982	1.005	1.033	1.004	0.98	0.99	1.02
Q969T9	WW domain-binding protein 2 OS=Homo	WBP2	28.087	23.065	5.4	1	1	1	1.008	1.039	0.971	1.031	1.117	1.017	0.947	0.893	0.98	1.04	0.90
O14880	Microsomal glutathione S-transferase 3 C	MGST3	16.516	84.935	30.9	5	18	5	0.858	1.598	1.42	0.982	0.562	1.547	0.647	0.544	0.98	0.86	0.48
O00151	PDZ and LIM domain protein 1 OS=Hon	PDLIM1	36.071	166.04	58.1	14	28	14	0.964	0.976	0.928	0.969	1.044	0.924	1.172	1.14	0.98	1.01	1.19

Q9NTZ6	RNA-binding protein 12 OS=Homo sapi	RBM12	97.394	119.23	19.1	15	23	15	1.031	1.043	1.025	1.003	0.96	1.159	0.91	0.928	0.98	1.02	0.89
O00459	Phosphatidylinositol 3-kinase regulatory	PIK3R2	81.544	11.277	3.8	2	2	2	1	1.026	0.985	0.996	1.138	0.771	1.1	1.042	0.98	0.94	1.06
Q9P2K8	eIF-2-alpha kinase GCN2 OS=Homo sap	EIF2AK4	186.91	33.568	2.7	4	4	4	0.976	0.913	0.822	1.025	1.015	0.922	1.089	1.308	0.98	1.03	1.27
Q6NYC1	Bifunctional arginine demethylase and ly	JMJD6	46.461	22.119	10.2	3	4	3	1.016	0.779	0.668	1.087	1.049	0.91	1.123	1.358	0.98	1.09	1.38
P42566	Epidermal growth factor receptor substrat	EPS15	98.655	141.5	21.5	16	17	16	1.058	0.997	1.009	1	0.941	0.922	0.999	0.958	0.98	0.91	0.95
P62136	Serine/threonine-protein phosphatase PP	PPP1CA	37.512	24.084	33	11	16	3	1.116	0.89	0.974	0.987	1.08	0.821	1.113	0.989	0.98	0.95	1.05
A6NMB9	Putative fidgetin-like protein 2 OS=Hom	FIGNL2	66.603	29.502	6.9	4	5	4	1.031	1.019	1.005	0.999	1.003	0.912	1.146	0.977	0.98	0.93	1.04
P57081	tRNA (guanine-N(7)-)-methyltransferase	WDR4	45.489	41.626	11.2	3	3	3	1.012	1.034	0.998	1.002	1.001	1.043	0.979	0.976	0.98	1.00	0.96
Q68CP9	AT-rich interactive domain-containing pr	ARID2	197.39	19.996	1.7	3	3	3	1.011	1.021	0.986	1	1.038	1.307	0.783	0.905	0.98	1.15	0.83
Q99941	Cyclic AMP-dependent transcription fact	ATF6B	76.708	27.649	4.8	2	2	2	1.037	1.116	1.122	0.982	0.964	1.098	0.839	0.886	0.98	0.96	0.80
P26583	High mobility group protein B2 OS=Hon	HMGB2	24.033	47.869	29.7	7	15	6	1.033	1.03	0.992	1.024	0.967	1.173	0.764	0.969	0.98	1.04	0.84
O00116	Alkyldihydroxyacetonephosphate synthas	AGPS	72.911	192.47	35.9	15	24	15	1.031	1.074	1.04	1.017	0.96	0.973	0.931	0.93	0.98	0.92	0.88
Q8TDH9	Biogenesis of lysosome-related organelle	BLOC1S5	21.609	15.313	11.2	2	2	2	1.002	0.993	0.933	1.016	0.832	0.959	1.166	1.152	0.98	0.90	1.16
A2RRP1	Neuroblastoma-amplified sequence OS=I	NBAS	268.57	42.727	2.8	6	6	6	1.022	1.059	1.022	1.011	0.958	1.107	0.943	0.964	0.98	0.99	0.92
Q86Y37	CDK2-associated and cullin domain-cont	CACUL1	41.063	18.693	8.4	3	3	3	1.009	0.985	0.952	0.996	1.075	1.003	1.015	1.02	0.98	1.04	1.02
Q8NBZ7	UDP-glucuronic acid decarboxylase 1 OS	UXS1	47.576	84.173	14.5	5	7	5	0.933	0.97	0.898	0.961	1.08	1.072	1.116	1.04	0.98	1.13	1.13
P49674	Casein kinase I isoform epsilon OS=Hon	CSNK1E	47.315	26.443	8.7	3	3	3	1.034	0.867	0.844	1.013	1.084	1.039	0.989	1.157	0.98	1.12	1.13
P30086	Phosphatidylethanolamine-binding protei	PEBP1	21.057	148.54	69.5	10	22	10	1.033	1.077	1.035	1.026	0.952	1.008	0.974	0.981	0.98	0.93	0.93
Q96SU4	Oxysterol-binding protein-related protein	OSBPL9	83.184	79.541	16	9	11	9	0.972	1.05	0.951	1.024	0.983	0.908	1.161	1.016	0.98	0.94	1.08
Q92576	PHD finger protein 3 OS=Homo sapiens	PHF3	229.48	100.14	7.7	12	12	12	1.023	0.951	0.904	1.024	1.048	0.986	1.005	1.025	0.98	1.03	1.03
Q6PL24	Protein TMED8 OS=Homo sapiens OX=	TMED8	35.74	38.748	9.2	2	2	2	0.938	0.991	0.848	1.036	1.049	1.1	0.963	1.162	0.98	1.11	1.10
Q5H9R7	Serine/threonine-protein phosphatase 6 r	PPP6R3	97.668	143.31	12.5	8	10	8	1.011	1.045	0.98	1.028	0.976	0.915	1.05	1.016	0.98	0.92	1.00
Q9H1K1	Iron-sulfur cluster assembly enzyme ISCU	ISCU	17.999	54.445	26.9	4	6	4	1.054	0.997	1.037	0.966	1.071	0.993	0.882	0.929	0.98	1.01	0.88
O60264	SWI/SNF-related matrix-associated actin	SMARCA5	121.9	247.75	25.4	24	35	16	1.058	0.95	0.962	0.999	1.048	1.104	0.945	1.006	0.98	1.07	0.97
Q9UBS3	DnaJ homolog subfamily B member 9 OS	DNAJB9	25.518	14.217	9.9	2	2	2	0.989	0.967	0.885	1.025	1.03	1.097	0.977	1.083	0.98	1.09	1.05
Q9UBD5	Origin recognition complex subunit 3 OS	ORC3	82.253	51.636	11.3	7	7	7	0.971	1.104	0.95	1.076	0.916	1.139	0.854	1.023	0.98	0.99	0.90
P0DP25	Calmodulin-3 OS=Homo sapiens OX=96	CALM3	16.837	135.2	47.7	7	30	7	1.014	1.018	0.986	0.998	0.94	0.939	1.024	0.956	0.98	0.92	0.97
P18615	Negative elongation factor E OS=Homo s	NELFE	43.239	65.496	25.5	7	7	6	1.028	1.001	1.034	0.947	0.98	1.192	0.866	0.998	0.98	1.07	0.92
O75976	Carboxypeptidase D OS=Homo sapiens (	CPD	152.93	31.987	3	4	6	4	1.052	0.85	0.882	0.975	1.141	0.84	1.212	1.086	0.98	1.04	1.21
P67809	Nuclease-sensitive element-binding prote	YBX1	35.924	237.82	31.8	7	25	4	1.013	1.014	0.958	1.021	1.017	0.979	0.883	1.039	0.98	0.98	0.95
Q9H6R3	Acyl-CoA synthetase short-chain family r	ACSS3	74.777	26.628	7.7	4	4	4	1.05	0.973	0.963	1.012	0.972	1.23	0.757	1.064	0.98	1.09	0.90
Q9P2X3	Protein IMPACT OS=Homo sapiens OX:	IMPACT	36.476	8.3811	3.4	1	1	1	1.102	0.912	0.916	1.05	0.967	0.795	1.129	1.067	0.98	0.87	1.09
O43505	Beta-1,4-glucuronyltransferase 1 OS=Ho	B4GAT1	47.119	6.3959	1.9	1	1	1	0.947	0.976	0.919	0.958	1.05	1.258	0.96	1.069	0.98	1.20	1.06
O75569	Interferon-inducible double-stranded RN	PRKRA	34.404	51.814	27.2	7	9	7	0.968	1.076	1.031	0.964	0.981	1.269	0.746	1.033	0.98	1.10	0.87
Q8N5M9	Protein jagunal homolog 1 OS=Homo sa	JAGN1	21.125	13.176	7.1	2	3	2	1.031	0.97	0.983	0.97	1.017	1.167	0.907	1.017	0.98	1.09	0.96
Q9UP83	Conserved oligomeric Golgi complex sub	COG5	92.742	58.207	11.9	9	9	9	0.988	1.01	0.937	1.013	1.032	1.197	0.906	1.023	0.98	1.12	0.97
O75376	Nuclear receptor corepressor 1 OS=Hom	NCOR1	270.21	25.844	2.1	4	4	4	1.032	1.083	1.015	1.049	0.854	1.153	0.83	0.991	0.98	0.95	0.86
Q13613	Myotubularin-related protein 1 OS=Hom	MTMR1	74.677	49.875	13.2	6	8	6	0.979	1.075	1.013	0.991	0.968	1.173	0.871	0.983	0.98	1.04	0.90
Q7L8L6	FAST kinase domain-containing protein !	FASTKD5	86.573	21.523	5.2	3	3	3	1.073	0.977	0.987	1.013	1.059	1.127	0.868	0.938	0.98	1.07	0.88
Q9NX74	tRNA-dihydrouridine(20) synthase [NAD	DUS2	55.05	21.468	6.9	3	4	3	1.001	1.123	1.039	1.033	1.068	0.902	0.846	1.025	0.98	0.93	0.88
Q9UKD1	Glucocorticoid modulatory element-bindi	GMEB2	56.421	7.3973	2.3	1	1	1	0.956	1.165	1.045	1.024	0.933	1.169	0.724	1.075	0.98	0.99	0.85
O75348	V-type proton ATPase subunit G 1 OS=H	ATP6V1G1	13.757	31.291	23.7	3	5	3	1.082	1.035	1.069	0.996	1.002	0.996	0.94	0.938	0.98	0.94	0.89
O43159	Ribosomal RNA-processing protein 8 OS	RRP8	50.714	49.363	22.1	7	8	7	0.925	0.977	0.925	0.93	1.072	1.387	0.848	1.131	0.98	1.29	1.04
P61019	Ras-related protein Rab-2A OS=Homo sa	RAB2A	23.545	72.354	46.7	9	18	3	0.988	0.988	0.956	0.971	1.06	0.949	1.103	0.975	0.98	1.02	1.05
Q13112	Chromatin assembly factor 1 subunit B C	CHAF1B	61.492	44.206	17.9	5	6	5	0.88	0.733	0.711	0.862	1.369	1.326	1.047	1.363	0.98	1.67	1.49
Q9UBS4	DnaJ homolog subfamily B member 11 O	DNAJB11	40.513	141.52	25.1	11	15	11	1.032	1.061	1.061	0.98	0.974	0.983	0.952	0.973	0.98	0.94	0.92
Q92769	Histone deacetylase 2 OS=Homo sapiens	HDAC2	55.364	81.791	26.8	10	15	7	1.076	0.975	0.985	1.015	0.988	0.987	0.994	0.952	0.98	0.96	0.95
Q5TC12	ATP synthase mitochondrial F1 complex	ATPAF1	36.436	37.039	18.6	4	5	4	0.941	1.029	0.962	0.959	1.099	1.092	1.045	1.009	0.98	1.11	1.04
P43897	Elongation factor Ts, mitochondrial OS=	TSFM	35.39	53.15	22.8	6	11	6	1.025	0.983	0.952	1.006	1.042	1.205	0.873	1.017	0.98	1.12	0.94
Q9BU89	Deoxyhypusine hydroxylase OS=Homo s	DOHH	32.904	26.903	12.6	3	3	3	0.9	0.982	0.694	1.141	0.897	1.373	0.761	1.306	0.98	1.21	1.10
P08134	Rho-related GTP-binding protein RhoC (	RHOC	22.006	15.927	28	4	7	1	1.028	1.046	0.978	1.044	0.92	0.977	0.992	1.022	0.97	0.91	0.97
Q9BSJ2	Gamma-tubulin complex component 2 OS	TUBGCP2	102.53	64.292	10	9	9	8	1.066	1.001	1.009	1.006	1.031	0.899	0.999	0.926	0.97	0.93	0.93
Q5T9A4	ATPase family AAA domain-containing p	ATAD3B	72.572	93.179	24.5	12	17	5	1.016	0.969	0.968	0.967	1.016	1.18	0.956	0.974	0.97	1.11	0.97
Q96H20	Vacuolar-sorting protein SNF8 OS=Hom	SNF8	28.864	30.731	17.8	4	5	4	1.055	1.041	1.085	0.958	1.052	1.215	0.702	0.945	0.97	1.08	0.79
Q13769	THO complex subunit 5 homolog OS=Ho	THOC5	78.507	48.541	9.2	6	9	6	1.012	1.041	0.995	1.006	1.018	0.926	1.064	1.021	0.97	0.95	1.02
Q9BQ04	RNA-binding protein 4B OS=Homo sapi	RBM4B	40.149	52.196	31.8	10	13	1	0.909	0.903	0.734	1.032	0.975	0.966	1.224	1.375	0.97	1.07	1.43
P36543	V-type proton ATPase subunit E 1 OS=H	ATP6V1E1	26.145	62.432	29.6	7	12	6	1.009	1.037	1.045	0.949	0.901	0.988	0.981	0.971	0.97	0.92	0.95
Q9P2E9	Ribosome-binding protein 1 OS=Homo s	RRBP1	152.45	323.31	39.9	44	70	44	1.022	1.022	1.012	0.98	0.984	0.979	0.969	0.963	0.97	0.96	0.95
P78536	Disintegrin and metalloproteinase domai	ADAM17	93.02	56.193	12.5	7	7	7	1.032	1.126	1.058	1.045	0.908	1.209	0.74	0.831	0.97	0.98	0.73
Q9BRG1	Vacuolar protein-sorting-associated prote	VPS25	20.747	11.77	10.2	2	3	2	0.927	1.11	0.947	1.038	0.832	1.063	1.038	1.143	0.97	0.93	1.07
Q92643	GPI-anchor transamidase OS=Homo sapi	PIGK	45.251	27.908	10.9	3	3	3	0.968	0.784	0.78	0.927	1.587	0.74	1.219	1.118	0.97	1.33	1.33
Q6Y7W6	GRB10-interacting GYF protein 2 OS=H	GIGYF2	150.07	104.67	12.9	12	16	12	1.054	0.967	0.93	1.039	1.005	0.914	1.107	1.002	0.97	0.95	1.04
Q8N3U4	Cohesin subunit SA-2 OS=Homo sapiens	STAG2	141.32	108.15	12.7	13	15	10	0.992	0.986	0.92	1.007	1.021	1.255	0.762	0.993	0.97	1.15	0.89
Q08211	ATP-dependent RNA helicase A OS=Ho	DHX9	140.96	323.31	34.9	37	71	36	1.006	1	0.985	0.969	1.018	1.069	0.921	1.021	0.97	1.04	0.97
Q9C0E8	Endoplasmic reticulum junction formatio	LNPK	47.739	37.072	16.6	6	7	6	1.022	0.945	0.917	0.999	1.003	1.052	0.882	1.05	0.97	1.04	0.98
Q13492	Phosphatidylinositol-binding clathrin ass	PICALM	70.754	86.759	20.1	10	14	10	1.102	0.939	0.989	0.999	1.024	1.054	0.894	0.911	0.97	1.02	0.88
Q9BZQ6	ER degradation-enhancing alpha-mannos	EDEM3	104.66	49.324	9.9	7	7	7	1.005	1.013	0.925	1.04	0.995	1.277	0.81	0.963	0.97	1.13	0.88
P06748	Nucleophosmin OS=Homo sapiens OX=9	NPM1	32.575	153.58	39.5	12	30	10	1.02	1.046	1.023	0.988	0.998	1.015	1	0.969	0.97	0.97	0.95
Q9UJX3	Anaphase-promoting complex subunit 7 (	ANAPC7	66.855	40.067	14	6	7	6	1	1.018	0.988	0.976	1.007	1.289	0.845	1.02	0.97	1.14	0.92
Q13472	DNA topoisomerase 3-alpha OS=Homo s	TOP3A	112.37	12.341	2.3	2	2	2	0.997	0.907	0.877	0.976	1.027	0.912	1.167	1.221	0.97	1.02	1.25
O75843	AP-1 complex subunit gamma-like 2 OS=	AP1G2	87.116	17.132	5.2	3	4	3	1.002	1.011	0.991	0.968	1.038	1.312	0.778	0.987	0.97	1.17	0.88
P20340	Ras-related protein Rab-6A OS=Homo sa	RAB6A	23.593	41.318	23.6	4	12	2	1.022	0.953	0.945	0.977	0.956	0.93	1.089	1.09	0.97	0.95	1.10
O95373	Importin-7 OS=Homo sapiens OX=9606	IPO7	119.52	187.12	22.4	18	24	17	1.02	1.025	0.983	1.007	1.022	1.135	0.887	0.972	0.97	1.05	0.91
Q27J81	Inverted formin-2 OS=Homo sapiens OX	INF2	135.62	323.31	27.9	23	37	21	1.011	1.058	1.026	0.987	0.934	1.071	0.956	0.952	0.97	0.97	0.92
Q9Y450	HBS1-like protein OS=Homo sapiens OX	HBS1L	75.472	58.415	14.6	8	10	8	1	1.066	1.002	1.008	0.921	0.908	1.131	0.945	0.97	0.89	1.00
Q9NV88	Integrator complex subunit 9 OS=Homo s	INTS9	73.814	20.992	7.6	3	4	3	0.941	1.006	0.908	0.986	1.116	1.12	0.99	1.05	0.97	1.15	1.05
Q13625	Apoptosis-stimulating of p53 protein 2 O	TP53BP2	125.61	23.299	3.6	3	3	3	1.065	0.991	1.017	0.983	1.088	0.82	1.111	0.936	0.97	0.93	1.00
Q49AR2	UPF0489 protein C5orf22 OS=Homo sap	C5orf22	49.967	19.724	9.5	3	3	3	0.994	1.025	0.953	1.011	1.016	1.254	0.755	1.056	0.97	1.12	0.90
Q9NZ09	Ubiquitin-associated protein 1 OS=Hom	UBAP1	55.083	12.231	6	2	2	2	0.882	1.134	1.14	0.821	0.933	1.092	1.202	1.088	0.97	1.00	1.14
P11586	C-1-tetrahydrofolate synthase, cytoplasm	MTHFD1	101.56	252.14	33.5	30	46	30	0.988	0.988	0.936	0.986	0.989	1.031	1.078	1.049	0.97	1.02	1.08
Q8N5C6	S1 RNA-binding domain-containing prot	SRBD1	111.77	94.757	13.4	14	15	14	1.022	1.055	1.063	0.957	1.08	0.984	1.012	0.986	0.97	0.99	0.96
Q9UKZ1	CCR4-NOT transcription complex subun	CNOT11	55.215	28.292	12.5	4	4	4	1.076	0.999	1.069	0.949	1.02	1.047	1.009	0.853	0.97	1.00	0.90
Q53LP3	Ankyrin repeat domain-containing protei	SOWAHC	55.671	24.017	10.9	3	3	3	0.981	1.02	0.938	1.008	0.998	1.01	1.004	1.029	0.97	1.00	1.02
O95104	Splicing factor, arginine/serine-rich 15 O	SCAF4	125.87	35.461	8.3	5	6	4	1.009	0.99	0.97	0.974	1.087	1.195	0.854	0.963	0.97	1.14	0.91
P47756	F-actin-capping protein subunit beta OS=	CAPZB	31.35	80.151	42.2	11	20	11	1.016	1.018	0.968	1.01	0.983	0.969	1.029	1.004	0.97	0.96	1.00
Q92541	RNA polymerase-associated protein RTF	RTF1	80.313	71.541	14.2	10	12	10	0.989	1.007	0.947	0.994	0.974	1.025	1.003	1.011	0.97	1.00	1.01
P35548	Homeobox protein MSX-2 OS=Homo sa	MSX2	28.897	19.686	13.5	3	4	3	0.997	1.034	1.016	0.959	0.987	1.334	0.736	0.94	0.97	1.14	0.83
Q5T5Y3	Calmodulin-regulated spectrin-associated	CAMSAP1	177.97	18.557	1.8	3	3	3	1.007	1.059	0.954	1.055	0.947	1.126	0.876	0.993	0.97	1.00	0.90
P84077	ADP-ribosylation factor 1 OS=Homo sap	ARF1	20.697	20.214	38.7	6	16	2	1.054	1.009	0.961	1.045	1.009	0.985	0.969	0.97	0.97	0.97	0.94
Q86Y82	Syntaxin-12 OS=Homo sapiens OX=960	STX12	31.642	27.856	18.1	4	4	3	1.012	0.827	0.847	0.941	1.26	0.751	1.225	1.227	0.97	1.09	1.33
Q86U42	Polyadenylate-binding protein 2 OS=Hor	PABPN1	32.749	22.486	13.1	3	3	3	1.055	0.928	0.955	0.973	0.966	1.007	1.104	1.017	0.97	0.99	1.07
Q9BQA9	Cytochrome b-245 chaperone 1 OS=Hom	CYBC1	20.774	8.0472	7.5	1	1	1	0.986	1.283	1.198	1.008	0.627	1.362	0.693	0.924	0.97	0.88	0.71
O95861	3'(2'),5'-bisphosphate nucleotidase 1 OS=	BPNT1	33.392	73.364	35.1	8	11	8	1.036	1.039	1.001	1.016	0.984	1.023	0.944	0.967	0.97	0.97	0.92
Q9BZF1	Oxysterol-binding protein-related protein	OSBPL8	101.19	88.794	9.8	6	9	6	1.024	1.041	1.052	0.955	0.977	0.961	1.031	1.006	0.97	0.94	0.99
Q9BT40	Inositol polyphosphate 5-phosphatase K (	INPP5K	51.09	33.667	12.5	5	5	5	0.995	1.031	0.982	0.987	1.016	1.054	0.992	1.075	0.97	1.02	1.02
Q9BZK7	F-box-like/WD repeat-containing protein	TBL1XR1	55.594	60.029	21	7	11	5	1.055	1.003	0.995	1.005	0.996	0.961	0.98	0.992	0.97	0.95	0.96
P00374	Dihydrofolate reductase OS=Homo sapie	DHFR	21.452	73.632	55.6	9	14	9	0.552	0.508	0.302	0.728	0.874	0.512	2.067	2.3	0.97	1.31	4.12
Q8NFZ0	F-box DNA helicase 1 OS=Homo sapien:	FBH1	117.68	19.847	3	3	4	3	1.019	0.995	0.932	1.025	1.008	1.048	0.906	1.098	0.97	1.02	1.00
Q5SRE5	Nucleoporin NUP188 homolog OS=Hom	NUP188	196.04	140.83	12.6	18	21	18	1.008	0.961	0.963	0.95	1.051	1.177	0.912	1.03	0.97	1.13	0.99
O75970	Multiple PDZ domain protein OS=Homo	MPDZ	221.62	77.45	7.1	10	10	10	1.004	1	0.94	1.007	0.995	1.102	1.021	1.017	0.97	1.05	1.02
Q9BY77	Polymerase delta-interacting protein 3 OS	POLDIP3	46.089	158.73	38.5	12	16	12	0.996	0.959	0.943	0.956	1.134	1.188	0.858	1.048	0.97	1.19	0.97
P51571	Translocon-associated protein subunit de	SSR4	18.998	32.343	19.7	3	7	3	1.043	1.038	0.992	1.029	0.93	1.053	0.862	0.876	0.97	0.95	0.84
P25325	3-mercaptopyruvate sulfurtransferase OS:	MPST	33.178	51.822	31	6	8	6	1.068	1.043	0.983	1.067	0.973	0.995	0.987	0.975	0.97	0.93	0.93
Q9BXK5	Bcl-2-like protein 13 OS=Homo sapiens	BCL2L13	52.723	71.639	16.1	5	6	4	0.986	1.055	1.033	0.949	0.958	1.082	1.021	1.023	0.97	1.00	1.00
Q9H4A3	Serine/threonine-protein kinase WNK1 C	WNK1	250.79	79.101	5.5	10	10	10	1.059	1.113	1.119	0.99	0.817	1.143	0.904	0.976	0.97	0.90	0.87
Q96II8	Leucine-rich repeat and calponin homolo	LRCH3	86.082	10.255	2.8	2	2	1	1.005	1.098	1.154	0.888	1.054	0.983	1.03	0.927	0.97	0.97	0.93
Q16763	Ubiquitin-conjugating enzyme E2 S OS=	UBE2S	23.845	48.383	34.2	6	8	6	1.068	1.034	1.044	0.997	0.929	1.178	0.788	0.916	0.97	1.00	0.81
P24534	Elongation factor 1-beta OS=Homo sapie	EEF1B2	24.763	64.066	33.8	5	15	4	1.032	1.085	1.008	1.047	0.98	1.031	0.89	0.911	0.97	0.95	0.85
P30520	Adenylosuccinate synthetase isozyme 2 C	ADSS	50.097	162.44	34.2	11	24	11	0.993	1.089	1.037	0.984	0.966	1.117	0.955	0.92	0.97	1.00	0.90
O43815	Striatin OS=Homo sapiens OX=9606 GN	STRN	86.131	94.282	20	9	11	9	1.015	1.056	0.979	1.031	0.93	0.968	1.074	1.026	0.97	0.92	1.01
Q9NRY5	Protein FAM114A2 OS=Homo sapiens (	FAM114A2	55.468	40.543	14.9	6	9	6	1.038	0.996	0.93	1.044	0.98	0.938	1.037	0.997	0.97	0.94	1.00
Q9BYD6	39S ribosomal protein L1, mitochondrial	MRPL1	36.908	27.995	11.7	3	5	3	1.047	0.98	0.982	0.985	1.02	1.247	0.731	1.062	0.97	1.12	0.88
Q9BRJ6	Uncharacterized protein C7orf50 OS=Ho	C7orf50	22.083	28.847	28.4	3	4	3	0.978	1.015	0.934	1	0.99	1.191	0.888	1.089	0.97	1.09	0.99
P22695	Cytochrome b-c1 complex subunit 2, mit	UQCRC2	48.442	175.05	36.4	12	22	12	1.007	0.98	0.946	0.982	1.062	1.154	0.939	0.984	0.97	1.12	0.97
Q9H9L4	KAT8 regulatory NSL complex subunit 2	KANSL2	55.042	14.608	4.3	2	3	2	0.966	1.046	0.896	1.056	1.037	0.831	1.202	1.005	0.97	0.93	1.10
P09669	Cytochrome c oxidase subunit 6C OS=H	COX6C	8.7813	17.986	24	3	4	3	1.04	1.039	1.039	0.978	1.082	1.105	0.85	0.908	0.97	1.05	0.85
Q8IZ83	Aldehyde dehydrogenase family 16 memb	ALDH16A1	85.126	51.019	10.1	7	7	7	1.001	1.072	1.019	0.992	0.955	1.116	0.904	0.996	0.97	1.00	0.92
Q9NUN5	Probable lysosomal cobalamin transporte	LMBRD1	61.388	14.126	3.3	1	4	1	0.974	1.098	0.942	1.068	0.894	1.11	0.886	1.066	0.97	0.97	0.94
O15514	DNA-directed RNA polymerase II subuni	POLR2D	16.311	31.259	32.4	3	3	3	1.012	0.991	0.965	0.978	1.122	0.939	1.14	1.003	0.97	1.03	1.07
P23588	Eukaryotic translation initiation factor 4E	EIF4B	69.15	189.12	31.6	20	27	20	1.04	1.024	1.026	0.976	0.985	0.99	0.983	0.956	0.97	0.96	0.94
Q8N3V7	Synaptopodin OS=Homo sapiens OX=96	SYNPO	99.462	81.711	10.5	7	11	7	1.023	1.005	0.974	0.993	0.956	1.135	0.995	1.021	0.97	1.03	0.99
Q13242	Serine/arginine-rich splicing factor 9 OS:	SRSF9	25.542	71.432	40.7	8	13	8	1.02	1.07	1.013	1.014	0.997	1.198	0.768	0.971	0.97	1.05	0.83
P49588	Alanine--tRNA ligase, cytoplasmic OS=H	AARS	106.81	323.31	39.7	32	48	32	0.941	0.946	0.866	0.964	1.027	0.98	1.169	1.326	0.97	1.06	1.32
Q9H6E4	Coiled-coil domain-containing protein 13	CCDC134	26.56	34.331	24.5	5	6	5	1.042	0.974	0.957	0.998	1.022	0.897	1.073	1.03	0.97	0.95	1.04
Q07820	Induced myeloid leukemia cell differentia	MCL1	37.337	9.3697	5.7	1	1	1	1.023	0.991	0.929	1.024	1.071	1.009	0.915	1.065	0.97	1.03	0.98
Q92844	TRAF family member-associated NF-kap	TANK	47.815	19.142	7.5	3	3	3	0.991	1.069	1.035	0.962	1.082	1.263	0.732	1.005	0.97	1.14	0.84
Q9NNW5	WD repeat-containing protein 6 OS=Hon	WDR6	121.72	20.494	3.4	3	3	3	0.993	1.032	0.963	1	1.058	0.929	1.141	1.059	0.97	0.98	1.09
Q7KZF4	Staphylococcal nuclease domain-containi	SND1	102	323.31	31.1	26	46	25	1.024	1.03	0.994	0.997	0.978	0.933	1.056	1.053	0.97	0.93	1.03
P52272	Heterogeneous nuclear ribonucleoprotein	HNRNPM	77.515	317.26	52.6	29	58	28	1.014	1.031	1.003	0.979	0.982	0.99	1.005	0.983	0.97	0.96	0.97
P04843	Dolichyl-diphosphooligosaccharide--prot	RPN1	68.569	239.28	46.1	22	44	22	1.033	1.007	0.997	0.98	0.984	1.026	0.984	0.977	0.97	0.99	0.96
Q9BRT6	Protein LLP homolog OS=Homo sapiens	LLPH	15.225	50.703	38	4	5	4	1.036	1.035	1.005	1.002	0.971	1.175	0.792	0.904	0.97	1.04	0.82
P46934	E3 ubiquitin-protein ligase NEDD4 OS=	NEDD4	149.11	39.78	5.8	5	5	4	0.913	0.863	0.747	0.974	1.097	0.977	1.15	1.383	0.97	1.17	1.43
Q7Z460	CLIP-associating protein 1 OS=Homo sa	CLASP1	169.45	92.42	10.8	14	15	14	0.975	1.056	0.971	0.997	0.958	1.032	0.923	1.063	0.97	0.98	0.98
O15530	3-phosphoinositide-dependent protein ki	PDPK1	63.151	52.824	14.6	8	8	8	0.994	1	0.909	1.023	1.013	0.986	1.04	1	0.97	1.00	1.02
P13804	Electron transfer flavoprotein subunit alp	ETFA	35.079	136.86	38.1	9	22	9	1.004	1.012	0.948	1.005	1.04	1.187	0.895	1	0.97	1.10	0.94
Q9BQE3	Tubulin alpha-1C chain OS=Homo sapie	TUBA1C	49.895	47.427	51.9	17	81	2	0.995	1.181	1.053	1.055	0.85	0.902	1.024	0.963	0.97	0.81	0.91
P41214	Eukaryotic translation initiation factor 2D	EIF2D	64.706	69.403	19.5	9	9	9	1.01	0.971	0.881	1.038	1.001	0.875	1.109	1.061	0.97	0.95	1.10
Q96A33	Coiled-coil domain-containing protein 47	CCDC47	55.873	132.41	24.2	9	11	9	1.026	1.016	1	0.978	0.979	1.184	0.849	0.939	0.97	1.06	0.88
O15182	Centrin-3 OS=Homo sapiens OX=9606 (	CETN3	19.55	18.976	17.4	2	2	2	1.112	0.93	0.947	1.031	0.981	0.925	1.039	0.981	0.97	0.93	0.99
Q9HCU5	Prolactin regulatory element-binding prot	PREB	45.468	39.374	16.3	5	6	5	1.022	0.978	0.987	0.95	0.989	1.126	1.053	1.091	0.97	1.06	1.07
Q9Y618	Nuclear receptor corepressor 2 OS=Hom	NCOR2	274.8	74.547	5.4	11	11	11	1.04	1.018	0.984	1.009	1.066	0.996	1.01	0.965	0.97	1.00	0.96
Q9Y6A4	Cilia- and flagella-associated protein 20 (	CFAP20	22.774	31.759	16.1	3	4	3	1.021	0.971	0.932	0.997	1.049	1.143	0.891	1.046	0.97	1.10	0.97
O14964	Hepatocyte growth factor-regulated tyrosi	HGS	86.191	44.879	8.8	7	8	6	0.996	1.025	0.961	0.996	0.955	1.024	1.003	1.059	0.97	0.98	1.02
P20700	Lamin-B1 OS=Homo sapiens OX=9606 (	LMNB1	66.408	302.11	45.2	29	60	28	1.006	0.95	0.94	0.954	1.102	0.946	1.081	1.05	0.97	1.05	1.09
P07384	Calpain-1 catalytic subunit OS=Homo sa	CAPN1	81.889	110.28	21	12	20	12	1.009	1.032	0.961	1.015	0.987	1.001	0.934	1.054	0.97	0.97	0.97
P41091	Eukaryotic translation initiation factor 2 :	EIF2S3	51.109	119.21	35.4	13	16	13	1.036	1.001	0.993	0.979	0.975	1.018	0.971	0.981	0.97	0.98	0.96
Q01804	OTU domain-containing protein 4 OS=H	OTUD4	124.04	20.732	3.3	3	3	3	1.035	1.032	0.962	1.039	0.953	0.947	1	1.038	0.97	0.92	0.99
Q9NX14	NADH dehydrogenase [ubiquinone] 1 be	NDUFB11	17.316	45.827	33.3	3	6	3	1.019	0.984	0.98	0.959	1.087	0.979	0.988	1.019	0.97	1.03	1.00
P36873	Serine/threonine-protein phosphatase PP	PPP1CC	36.983	11.148	26.6	9	14	1	1.09	0.911	0.92	1.017	1.193	0.768	1.096	0.972	0.97	0.98	1.03
Q4G0N4	NAD kinase 2, mitochondrial OS=Homo	NADK2	49.432	42.848	16.1	6	7	6	1.005	0.989	0.948	0.982	1.13	1.128	0.934	0.994	0.97	1.13	0.97
P20042	Eukaryotic translation initiation factor 2 :	EIF2S2	38.388	182.09	41.7	14	21	14	1.014	1.005	0.961	0.993	0.973	0.973	1	1.036	0.97	0.96	1.01
O43427	Acidic fibroblast growth factor intracellu	FIBP	41.878	8.0753	2.5	1	2	1	1.014	1.036	1.037	0.947	1.122	1.136	0.883	0.91	0.97	1.10	0.87
Q9H2M9	Rab3 GTPase-activating protein non-cata	RAB3GAP	155.98	148.79	18.7	21	26	21	1.011	1.006	0.976	0.976	1.05	0.992	0.938	1.075	0.97	1.01	1.00
Q9H0A0	RNA cytidine acetyltransferase OS=Hom	NAT10	115.73	154.92	17.7	16	24	16	0.976	0.946	0.921	0.939	1.065	1.04	1.074	1.104	0.97	1.10	1.13
Q6ZN18	Zinc finger protein AEBP2 OS=Homo sa	AEBP2	54.466	15.861	5	2	3	2	1.039	0.911	0.92	0.967	1.157	1.194	0.912	1.034	0.97	1.21	1.00
Q93009	Ubiquitin carboxyl-terminal hydrolase 7 (	USP7	128.3	161.93	24.7	22	28	21	1.045	1.024	0.974	1.028	0.977	1.012	0.904	0.961	0.97	0.96	0.90

O95219	Sorting nexin-4 OS=Homo sapiens OX=	SNX4	51.908	40.444	17.1	6	6	6	1.024	0.997	0.953	1.002	0.969	1.093	0.927	1.041	0.97	1.02	0.97
Q16774	Guanylate kinase OS=Homo sapiens OX	GUK1	21.725	21.892	15.2	3	4	3	1.032	1.018	1.033	0.95	0.984	1.172	0.884	1.004	0.97	1.05	0.92
Q5JVF3	PCI domain-containing protein 2 OS=Ho	PCID2	46.029	55.623	19	8	11	8	1.015	1.059	1.006	1	0.985	1.132	0.877	0.997	0.97	1.02	0.90
Q9Y657	Spindlin-1 OS=Homo sapiens OX=9606	SPIN1	29.6	15.755	14.5	2	2	2	0.964	0.919	0.894	0.927	1.106	1.069	1.135	1.13	0.97	1.16	1.20
O00186	Syntaxin-binding protein 3 OS=Homo sa	STXBP3	67.764	44.54	11.1	6	8	6	1.005	1.06	0.974	1.023	1	1.082	0.949	1.037	0.97	1.01	0.96
Q9H2V7	Protein spinster homolog 1 OS=Homo sa	SPNS1	56.629	26.632	8.1	3	5	3	0.967	0.943	0.91	0.937	1.21	1.056	1.016	1.042	0.97	1.19	1.08
Q13838	Spliceosome RNA helicase DDX39B OS	DDX39B	48.991	119.19	35	12	26	5	1.117	0.971	1.021	0.998	0.979	0.973	0.984	0.922	0.97	0.93	0.91
O14974	Protein phosphatase 1 regulatory subunit	PPP1R12A	115.28	170.03	20.3	17	22	16	1.041	1.074	1.019	1.026	0.942	0.94	1	0.958	0.97	0.89	0.93
O14497	AT-rich interactive domain-containing pr	ARID1A	242.04	181.46	12.4	20	23	19	1.043	1.035	1.007	1.002	0.947	1.041	0.914	0.933	0.97	0.96	0.89
Q96HP4	Oxidoreductase NAD-binding domain-co	OXNAD1	34.854	24.829	11.2	3	4	3	1.003	0.951	0.912	0.977	1.102	1.312	0.754	1.069	0.97	1.24	0.93
Q15003	Condensin complex subunit 2 OS=Homo	NCAPH	82.562	213.31	26.3	15	20	15	1.021	1.075	1.035	0.991	0.997	1.127	0.833	0.893	0.97	1.01	0.82
Q96B26	Exosome complex component RRP43 OS	EXOSC8	30.039	25.754	15.2	4	4	4	0.932	1.157	0.931	1.088	1.011	1.21	0.712	1.014	0.97	1.06	0.83
O60934	Nibrin OS=Homo sapiens OX=9606 GN	NBN	84.958	48.797	9	6	6	6	1.018	0.98	0.975	0.956	1.086	0.973	0.98	1.003	0.97	1.03	0.99
Q2NL82	Pre-rRNA-processing protein TSR1 hom	TSR1	91.809	137.35	19.8	12	15	12	0.98	1.017	0.972	0.958	1.076	0.98	1.101	0.981	0.97	1.03	1.04
Q15436	Protein transport protein Sec23A OS=Ho	SEC23A	86.16	97.858	21.8	12	17	11	1.011	1.043	0.971	1.014	0.964	0.99	1.005	0.983	0.97	0.95	0.97
O60885	Bromodomain-containing protein 4 OS=I	BRD4	152.22	108.13	11.2	13	17	8	1.064	0.99	0.983	1.002	0.984	0.964	0.964	0.953	0.97	0.95	0.93
Q96AQ8	Mitochondrial calcium uniporter regulato	MCUR1	39.694	12.769	5.6	2	2	2	1.046	0.948	0.956	0.971	1.057	1.155	0.863	1.053	0.97	1.11	0.96
Q8IWL3	Iron-sulfur cluster co-chaperone protein I	HSCB	27.422	6.4274	4.3	1	1	1	1.019	1.004	0.904	1.051	1.028	1.227	0.739	1.042	0.97	1.11	0.88
P23634	Plasma membrane calcium-transporting A	ATP2B4	137.92	281.31	25.1	23	30	15	1.045	1.026	0.971	1.03	0.943	0.896	1.049	1.053	0.97	0.89	1.01
Q9UQB8	Brain-specific angiogenesis inhibitor 1-a	BAIAP2	60.867	61.801	20.3	9	11	9	0.978	0.945	0.891	0.967	1.072	1.004	1.21	1.137	0.97	1.08	1.22
P35611	Alpha-adducin OS=Homo sapiens OX=9	ADD1	80.954	79.411	20.1	10	11	10	1.046	1.05	1.037	0.988	0.94	0.935	1.05	0.942	0.97	0.89	0.95
P35270	Sepiapterin reductase OS=Homo sapiens	SPR	28.048	32.631	19.9	4	4	4	0.986	0.987	0.932	0.974	0.974	1.066	1.219	0.945	0.97	1.03	1.10
Q14498	RNA-binding protein 39 OS=Homo sapi	RBM39	59.379	83.31	17.7	9	21	8	1.035	1.022	1.02	0.967	1.028	1.036	0.956	0.988	0.97	1.00	0.95
Q9H9A5	CCR4-NOT transcription complex subun	CNOT10	82.309	13.052	3.6	2	2	2	1.088	0.851	0.923	0.95	1.041	0.851	1.208	1.115	0.97	0.98	1.20
Q8IUR7	Armadillo repeat-containing protein 8 OS	ARMC8	75.509	43.443	10.1	6	7	6	1.053	1.017	0.984	1.015	0.912	0.881	1.046	1.087	0.97	0.87	1.03
P0CG08	Golgi pH regulator B OS=Homo sapiens	GPR89B	52.916	14.324	5.1	2	3	2	1.095	0.932	1.014	0.943	0.958	1.194	0.899	0.995	0.97	1.06	0.93
O43709	Probable 18S rRNA (guanine-N(7))-meth	BUD23	31.88	22.033	14.2	2	2	2	1.023	1.088	1.002	1.036	0.977	1.249	0.817	0.82	0.97	1.05	0.78
Q9UBQ6	Exostosin-like 2 OS=Homo sapiens OX=	EXTL2	37.465	6.7987	3.3	1	2	1	1.051	0.998	1.059	0.919	0.951	0.946	1.169	0.987	0.97	0.93	1.05
P48509	CD151 antigen OS=Homo sapiens OX=	CD151	28.295	27.226	14.2	4	5	4	0.997	0.931	0.863	0.998	1.06	0.843	1.177	1.196	0.97	0.99	1.23
Q9HA77	Probable cysteine--tRNA ligase, mitocho	CARS2	62.223	50.547	13.8	7	9	7	1.014	1.025	0.967	1.001	0.989	0.839	1.17	1.018	0.97	0.90	1.07
Q9NYL9	Tropomodulin-3 OS=Homo sapiens OX=	TMOD3	39.594	151.63	42.9	13	18	13	1.024	0.981	0.954	0.981	1.031	0.906	1.146	1.056	0.97	0.97	1.10
P56134	ATP synthase subunit f, mitochondrial O	ATP5J2	10.918	19.071	25.5	2	4	2	0.922	0.966	0.936	0.886	1.167	1.125	0.981	0.98	0.97	1.21	1.04
Q12872	Splicing factor, suppressor of white-apric	SFSWAP	104.82	71.614	7.3	5	5	5	1.047	1.01	1.009	0.976	0.936	1.074	0.939	1.057	0.96	0.98	0.97
Q6ZRP7	Sulfhydryl oxidase 2 OS=Homo sapiens	QSOX2	77.528	32.219	8.6	4	4	4	1.02	1.024	1.036	0.936	1.042	1.222	0.883	0.955	0.96	1.11	0.90
O43765	Small glutamine-rich tetratricopeptide re	SGTA	34.063	52.852	25.6	8	11	8	1.084	1.016	1.005	1.021	1.012	1.028	0.936	0.957	0.96	0.97	0.90
Q9Y4W2	Ribosomal biogenesis protein LAS1L OS	LAS1L	83.064	98.555	21.5	12	12	12	1.042	0.915	0.881	1.007	1.101	0.9	1.109	1.074	0.96	1.02	1.12
P32119	Peroxiredoxin-2 OS=Homo sapiens OX=	PRDX2	21.892	38.802	36.4	7	14	6	1.039	1.054	1.024	0.995	0.988	1.189	0.8	1.018	0.96	1.04	0.87
P08574	Cytochrome c1, heme protein, mitochond	CYC1	35.422	28.05	4.9	1	1	1	0.941	0.952	0.736	1.09	0.957	0.992	0.977	1.412	0.96	1.03	1.26
Q9UBE0	SUMO-activating enzyme subunit 1 OS=	SAE1	38.449	78.575	38.7	10	15	9	0.994	1.04	0.964	0.998	0.999	1.006	0.861	1.011	0.96	0.99	0.92
Q9BPX3	Condensin complex subunit 3 OS=Homo	NCAPG	114.33	207.08	21.4	18	24	18	1.019	1.043	0.994	0.995	0.964	1.027	0.961	0.99	0.96	0.97	0.95
P67812	Signal peptidase complex catalytic subun	SEC11A	20.625	31.79	20.7	4	6	4	1.048	1.04	1.035	0.979	1.053	0.993	0.928	0.91	0.96	0.98	0.88
Q9BYN8	28S ribosomal protein S26, mitochondria	MRPS26	24.211	30.435	27.3	5	5	5	1.085	0.916	0.891	1.039	1.003	1.324	0.719	1.089	0.96	1.16	0.90
P08572	Collagen alpha-2(IV) chain OS=Homo sa	COL4A2	167.55	94.648	9.4	10	12	10	0.997	1.002	0.917	1.011	1.003	0.922	1.113	1.111	0.96	0.96	1.11
P55212	Caspase-6 OS=Homo sapiens OX=9606	CASP6	33.31	20.322	10.9	3	3	3	1.038	0.989	0.956	0.999	0.917	0.821	1.155	1.163	0.96	0.86	1.14
Q8NI60	Atypical kinase COQ8A, mitochondrial	COQ8A	71.949	51.949	7.9	5	4	4	0.922	1.035	0.866	1.021	1.108	1.054	1.004	1.097	0.96	1.10	1.07
O00429	Dynamin-1-like protein OS=Homo sapie	DNM1L	81.876	170.92	34.6	20	26	20	1.02	1.018	0.977	0.988	0.999	1.018	0.983	1.029	0.96	0.99	0.99
Q9H3U1	Protein unc-45 homolog A OS=Homo sa	UNC45A	103.08	114.87	18.5	15	17	15	1.045	0.99	0.954	1.008	1.01	0.969	1.052	0.971	0.96	0.97	0.99
Q9BSJ8	Extended synaptotagmin-1 OS=Homo sa	ESYT1	122.85	127.25	14.7	13	18	13	0.989	1.046	0.967	0.995	0.963	0.948	1.135	1.058	0.96	0.94	1.08
Q12772	Sterol regulatory element-binding protein	SREBF2	123.69	18.733	1.8	2	2	2	0.976	1.029	0.892	1.041	0.983	1.261	0.917	0.948	0.96	1.12	0.93
Q01415	N-acetylgalactosamine kinase OS=Homo	GALK2	50.378	31.449	10.9	5	6	5	0.987	0.934	0.936	0.916	1.053	0.934	1.135	1.024	0.96	1.03	1.12
P62140	Serine/threonine-protein phosphatase PP	PPP1CB	37.186	104.93	33.6	11	18	3	0.979	0.94	0.874	0.976	1.023	1.019	1.166	1.065	0.96	1.06	1.16
Q3MHD2	Protein LSM12 homolog OS=Homo sapi	LSM12	21.701	13.793	11.8	2	3	2	1.09	0.965	0.939	1.042	1.007	0.917	1.002	1.022	0.96	0.94	0.98
Q9UBI1	COMM domain-containing protein 3 OS	COMMD3	22.151	35.516	24.6	3	4	3	1.049	1.031	1.063	0.942	0.971	0.995	1.047	1.037	0.96	0.95	1.00
P14324	Farnesyl pyrophosphate synthase OS=Ho	FDPS	48.275	52.188	19.1	7	10	7	1.007	1.039	0.942	1.03	0.999	1.269	0.857	1.021	0.96	1.11	0.92
O60437	Periplakin OS=Homo sapiens OX=9606	PPL	204.74	133.1	10.3	14	15	14	0.99	0.916	0.881	0.956	1.087	0.976	1.154	1.121	0.96	1.08	1.19
Q9BQ61	Telomerase RNA component interacting	TRIR	18.419	22.855	30.1	3	4	3	1.03	0.985	0.957	0.985	0.95	1.172	0.875	1.072	0.96	1.05	0.97
P49755	Transmembrane emp24 domain-containin	TMED10	24.976	114.32	33.8	6	10	6	1.004	1.035	0.991	0.974	0.908	0.97	1.144	0.959	0.96	0.92	1.03
Q9UBS0	Ribosomal protein S6 kinase beta-2 OS=	RPS6KB2	53.455	19.123	6.8	3	4	3	1.015	0.941	0.919	0.966	1	0.92	1.13	1.188	0.96	0.98	1.19
Q96TA2	ATP-dependent zinc metalloprotease YM	YME1L1	86.454	85.07	14.9	9	15	9	1.009	1.024	1.004	0.955	1.057	1.003	0.941	1.006	0.96	1.01	0.96
Q8IZ73	RNA pseudouridylate synthase domain-c	RPUSD2	61.311	53.323	16.9	7	8	7	0.955	1.016	0.919	0.98	1.032	1.117	0.948	1.152	0.96	1.09	1.07
O43504	Ragulator complex protein LAMTOR5 O	LAMTOR5	9.6138	67.483	54.9	3	4	3	0.997	1.107	1.059	0.968	0.927	1.004	1.004	0.937	0.96	0.92	0.92
P31930	Cytochrome b-c1 complex subunit 1, mit	UQCRC1	52.645	134.53	26.2	7	10	7	1.036	0.957	0.947	0.973	1.089	1.008	0.919	0.995	0.96	1.05	0.96
Q9NW08	DNA-directed RNA polymerase III subun	POLR3B	127.78	16.668	2.3	3	3	3	1.007	1.063	0.983	1.011	1.126	1.087	0.802	0.97	0.96	1.07	0.86
Q9NSE4	Isoleucine--tRNA ligase, mitochondrial	IARS2	113.79	103.04	17.6	14	17	13	1.003	1.036	0.968	0.996	0.939	1.103	0.88	0.963	0.96	1.00	0.90
P20020	Plasma membrane calcium-transporting A	ATP2B1	138.75	27.121	11.4	11	16	3	1.041	0.889	0.922	0.937	1.153	1.131	0.914	1.089	0.96	1.18	1.04
Q12979	Active breakpoint cluster region-related	ABR	97.597	50.826	8.7	7	9	6	1.03	1.003	0.968	0.99	0.979	0.949	1.104	1.066	0.96	0.95	1.07
P46108	Adapter molecule crk OS=Homo sapiens	CRK	33.83	47.025	23.4	5	6	4	1.042	0.991	0.949	1.009	0.948	0.943	1.112	1.055	0.96	0.93	1.07
Q16254	Transcription factor E2F4 OS=Homo sap	E2F4	43.96	28.599	10.2	4	5	4	1.019	1.062	0.996	1.008	1.077	0.883	1.018	0.972	0.96	0.94	0.96
Q8N4J0	Carnosine N-methyltransferase OS=Hom	CARNMT1	47.185	17.658	6.8	3	4	3	1.07	1.062	1.048	1.005	1.033	0.917	0.94	0.924	0.96	0.91	0.87
Q13283	Ras GTPase-activating protein-binding p	G3BP1	52.164	172.54	40.1	14	23	12	1.026	1.052	1.003	0.998	0.919	1.051	0.914	1.031	0.96	0.95	0.94
P53611	Geranylgeranyl transferase type-2 subuni	RABGGTB	36.924	30.617	13	4	5	4	1.05	1.128	1.06	1.037	0.923	1.101	0.836	0.917	0.96	0.93	0.80
P53990	IST1 homolog OS=Homo sapiens OX=9	IST1	39.75	38.507	14.8	6	8	6	1.052	1.018	0.987	1.006	1.014	0.882	1.076	0.972	0.96	0.92	0.99
Q7Z4G4	tRNA (guanine(10)-N2)-methyltransferas	TRMT11	53.42	15.404	4.5	2	3	2	1.004	0.931	0.966	0.897	1.075	1.018	1.086	1.063	0.96	1.08	1.11
P0DN79	Cystathionine beta-synthase-like protein	CBSL	60.586	120.8	23.6	11	14	11	0.982	1.166	1.027	1.041	0.839	1.128	0.888	0.956	0.96	0.92	0.86
O75306	NADH dehydrogenase [ubiquinone] iron-	NDUFS2	52.545	82.154	24	10	13	10	1.073	0.965	1.005	0.957	1.017	1.033	1.101	1.001	0.96	1.01	1.03
Q9BYC8	39S ribosomal protein L32, mitochondria	MRPL32	21.405	49.69	24.5	4	5	4	1.051	0.986	0.966	0.995	1.016	0.999	1.082	1.007	0.96	0.99	1.03
O75940	Survival of motor neuron-related-splicing	SMNDC1	26.711	103.47	42.4	6	9	6	0.994	1.069	1.03	0.956	0.941	1.236	0.91	1.104	0.96	1.06	0.98
P26885	Peptidyl-prolyl cis-trans isomerase FKB	FKBP2	15.649	19.565	18.3	2	4	2	1.013	0.94	0.921	0.959	1.035	1.01	1.072	1.111	0.96	1.05	1.12
Q86XK2	F-box only protein 11 OS=Homo sapiens	FBXO11	103.58	11.948	2.2	2	2	2	1.02	1.039	0.977	1.005	1.04	1.344	0.707	0.912	0.96	1.16	0.79
O14733	Dual specificity mitogen-activated protei	MAP2K7	47.484	19.234	8.1	3	3	3	1.02	0.957	0.923	0.98	1.153	1.274	0.671	1.089	0.96	1.23	0.89
Q8IZL8	Proline-, glutamic acid- and leucine-rich	PELP1	119.7	93.625	13.5	10	17	10	1.03	0.998	0.976	0.976	1.036	0.967	1.09	1.032	0.96	0.99	1.05
O94905	Erlin-2 OS=Homo sapiens OX=9606 GN	ERLIN2	37.839	130.65	38.3	11	19	9	1.006	1.072	1.047	0.953	0.968	0.906	1.109	0.968	0.96	0.90	1.00
Q9Y5L0	Transportin-3 OS=Homo sapiens OX=96	TNPO3	104.2	88.328	15.1	11	12	11	1.019	1.005	0.93	1.018	1.013	0.992	0.943	1.079	0.96	0.99	1.00
Q9Y259	Choline/ethanolamine kinase OS=Homo	CHKB	45.271	6.6101	2.8	1	1	1	1.065	1.091	1.01	1.065	0.847	0.956	1.061	0.862	0.96	0.84	0.89
Q92623	Tetratricopeptide repeat protein 9A OS=I	TTC9	24.379	7.3459	5	1	1	1	1.065	0.931	0.94	0.981	1.106	0.77	1.166	1.059	0.96	0.94	1.11
Q5T5P2	Sickle tail protein homolog OS=Homo sa	KIAA1217	214.11	264.48	21.2	33	46	32	1.032	1.041	0.985	1.01	0.964	0.901	1.072	0.97	0.96	0.90	0.99
Q32P41	tRNA (guanine(37)-N1)-methyltransferas	TRMT5	58.246	47.615	14.5	7	9	7	1.016	1.083	1.019	1.001	1	0.931	0.962	0.969	0.96	0.92	0.92
O75175	CCR4-NOT transcription complex subun	CNOT3	81.871	37.144	8.6	5	6	5	0.963	1.077	0.985	0.978	0.947	0.906	1.097	1.084	0.96	0.91	1.07
Q96SY0	Integrator complex subunit 14 OS=Homo	INTS14	57.47	38.222	8.5	3	3	3	1.054	0.985	1.032	0.93	1.068	1.286	0.781	0.936	0.96	1.15	0.84
P63172	Dynein light chain Tctex-type 1 OS=Hon	DYNLT1	12.452	19.774	44.2	3	2	2	0.954	1.217	1.047	1.042	0.779	1.013	1.056	0.961	0.96	0.83	0.93
Q7Z2K6	Endoplasmic reticulum metallopeptidase	ERMP1	100.23	69.103	11.7	9	11	9	1.001	1.117	1.016	1.022	0.914	0.928	1.037	1.047	0.96	0.87	0.98
P61024	Cyclin-dependent kinases regulatory subu	CKS1B	9.6601	8.3692	20.3	1	1	1	1.033	0.974	0.9	1.031	1.018	1.198	0.799	1.064	0.96	1.10	0.93
P17987	T-complex protein 1 subunit alpha OS=H	TCP1	60.343	158.93	35.8	18	31	17	0.975	1.03	0.963	0.966	1.029	1.18	0.933	0.951	0.96	1.10	0.94
Q9ULV4	Coronin-1C OS=Homo sapiens OX=960	CORO1C	53.248	115.86	24.7	14	28	14	1.051	1.023	1.016	0.979	1.012	0.939	1.082	0.949	0.96	0.94	0.98
Q9NVH1	DnaJ homolog subfamily C member 11 O	DNAJC11	63.277	36.662	9.5	5	7	5	0.936	1.057	0.938	0.979	1.002	1.24	0.787	1.115	0.96	1.12	0.95
O43615	Mitochondrial import inner membrane tra	TIMM44	51.355	168.77	37.2	15	19	15	0.993	1	0.955	0.962	1.072	1.124	0.906	1.002	0.96	1.10	0.96
Q12983	BCL2/adenovirus E1B 19 kDa protein-in	BNIP3	27.832	22.095	11.6	3	4	3	0.836	1.384	1.081	1.054	0.701	1.136	0.873	1.028	0.96	0.83	0.86
Q5KU26	Collectin-12 OS=Homo sapiens OX=960	COLEC12	81.514	13.551	2.7	2	2	2	0.977	1.134	1.002	1.028	0.818	0.994	1.019	1.095	0.96	0.86	1.00
Q8WYP5	Protein ELYS OS=Homo sapiens OX=9	AHCTF1	252.5	194.29	10.9	20	27	20	1.028	0.926	0.914	0.965	1.135	0.999	1.073	0.982	0.96	1.09	1.05
Q9BTE3	Mini-chromosome maintenance complex-	MCMBP	72.979	58.241	14.5	7	9	7	0.978	1.025	0.947	0.979	0.98	1.063	1.062	1.057	0.96	1.02	1.06
Q6P4A8	Phospholipase B-like 1 OS=Homo sapie	PLBD1	63.254	32.753	10.1	4	4	4	1.039	1.092	1.069	0.98	0.883	1.002	0.994	0.989	0.96	0.88	0.93
P15170	Eukaryotic peptide chain release factor G	GSPT1	55.755	124.83	25.9	10	21	10	1.044	1.061	0.997	1.027	0.968	0.943	0.956	0.912	0.96	0.91	0.89
Q9Y3I1	F-box only protein 7 OS=Homo sapiens	FBXO7	58.502	28.498	10.2	4	5	4	1.025	1.028	1.027	0.947	0.864	1.082	0.909	1.152	0.96	0.95	1.00
P68363	Tubulin alpha-1B chain OS=Homo sapie	TUBA1B	50.151	8.9424	51.7	17	92	1	1.021	1.023	0.971	0.994	0.975	0.976	1.108	0.995	0.96	0.95	1.03
Q96QC0	Serine/threonine-protein phosphatase 1 r	PPP1R10	99.057	64.612	12.4	10	12	10	1.048	1.125	1.062	1.027	0.988	1.097	0.829	0.93	0.96	0.96	0.81
O75027	ATP-binding cassette sub-family B mem	ABCB7	82.64	37.782	7	5	6	5	0.988	1.074	1.037	0.945	1.023	1.016	1.029	1.079	0.96	0.99	1.02
Q9UII4	E3 ISG15--protein ligase HERC5 OS=H	HERC5	116.85	54.04	8.3	8	9	8	1.034	1	0.952	1.003	0.985	0.985	1.061	1.101	0.96	0.97	1.06
Q8N1F7	Nuclear pore complex protein Nup93 OS	NUP93	93.487	236.1	30.5	24	36	24	1.043	1.004	0.972	0.995	0.975	0.984	1.007	1.011	0.96	0.96	0.99
P82675	28S ribosomal protein S5, mitochondrial	MRPS5	48.006	60.609	21.2	8	9	8	1.032	1.036	0.987	1	1.05	1.049	0.902	0.888	0.96	1.01	0.87
Q96TC7	Regulator of microtubule dynamics prote	RMDN3	52.118	31.016	11.7	4	5	4	0.919	1.016	0.863	0.996	1.028	1.214	0.763	1.349	0.96	1.16	1.09
Q9UL40	Zinc finger protein 346 OS=Homo sapie	ZNF346	32.932	35.471	16.7	4	6	4	1.026	0.779	0.787	0.947	1.263	0.947	1.097	1.232	0.96	1.22	1.29
Q2M389	WASH complex subunit 4 OS=Homo sa	WASHC4	136.4	19.513	2.5	3	3	3	1.016	1.015	1.052	0.899	1.107	0.97	1.124	0.933	0.96	1.02	1.01
Q4V328	GRIP1-associated protein 1 OS=Homo s	GRIPAP1	95.988	69.314	15.6	9	9	9	1.017	1.036	0.978	0.994	0.998	0.891	1.04	1.061	0.96	0.92	1.02
P52655	Transcription initiation factor IIA subuni	GTF2A1	41.513	9.9765	2.9	1	2	1	1.049	1.053	0.952	1.067	0.907	1.105	0.899	0.939	0.96	0.96	0.87
Q9Y697	Cysteine desulfurase, mitochondrial OS=	NFS1	50.195	101.85	31.5	10	11	9	1.063	0.96	0.954	0.989	1.085	1.147	0.859	1.071	0.96	1.10	0.95
Q9P2K3	REST corepressor 3 OS=Homo sapiens	RCOR3	55.581	14.812	7.9	4	7	2	1.055	0.99	1.049	0.915	1.105	0.914	1.079	0.969	0.96	0.99	1.00
P62316	Small nuclear ribonucleoprotein Sm D2	SNRPD2	13.527	90.787	55.9	7	24	7	1.022	0.972	0.94	0.975	1.047	1.001	0.973	1.018	0.96	1.03	1.00
Q969H6	Ribonuclease P/MRP protein subunit PC	POP5	18.819	7.0657	7.4	1	2	1	1.007	1.004	0.962	0.969	0.95	1.314	0.814	1.069	0.96	1.13	0.94
Q9H0A8	COMM domain-containing protein 4 OS	COMMD4	21.764	33.108	29.1	5	7	5	0.943	1.04	0.937	0.967	1.009	0.917	1.158	1.047	0.96	0.97	1.11
Q9NRR8	CDC42 small effector protein 1 OS=Hon	CDC42SE1	8.9252	6.7528	15.2	1	2	1	0.986	1.019	0.866	1.059	0.979	1.394	0.691	1.043	0.96	1.18	0.86
O76031	ATP-dependent Clp protease ATP-bindin	CLPX	69.223	119.34	26.2	15	25	15	1.005	1.023	0.967	0.98	1.041	1.202	0.862	1.017	0.96	1.11	0.93
Q8TCE6	Protein FAM45A OS=Homo sapiens OX	FAM45A	40.513	38.535	7.6	3	4	3	0.987	1.066	0.959	1.012	0.958	0.951	1.188	0.996	0.96	0.93	1.06
Q5EBL8	PDZ domain-containing protein 11 OS=I	PDZD11	16.131	13.683	13.6	2	3	2	1.096	0.98	1.085	0.908	1.008	1.269	0.758	0.956	0.96	1.10	0.83
A4D1E9	GTP-binding protein 10 OS=Homo sapie	GTPBP10	42.932	12.459	4.9	2	2	2	1.048	0.998	0.995	0.969	1.048	1.001	0.961	1.029	0.96	1.00	0.97
Q13107	Ubiquitin carboxyl-terminal hydrolase 4	USP4	108.56	20.333	4.4	3	4	2	0.77	1.358	1.048	0.994	0.73	0.938	1.102	1.329	0.96	0.78	1.14
Q9NRP4	Succinate dehydrogenase assembly factor	SDHAF3	14.651	8.5961	8.8	1	2	1	1.032	1.046	0.988	1.006	1.006	1.148	0.846	0.962	0.96	1.04	0.87
Q9NS00	Glycoprotein-N-acetylgalactosamine 3-be	C1GALT1	42.202	27.749	15.7	4	6	4	0.989	1.013	0.99	0.93	0.967	1	1.104	1.092	0.96	0.98	1.10
P68104	Elongation factor 1-alpha 1 OS=Homo s	EEF1A1	50.14	310.89	43.5	18	69	17	1.044	1.101	1.053	1.004	0.938	1.051	0.855	0.894	0.96	0.93	0.82
O75937	DnaJ homolog subfamily C member 8 OS	DNAJC8	29.841	44.788	19.8	4	11	4	1.008	1.086	0.998	1.01	1.004	1.248	0.863	0.942	0.96	1.08	0.86
P14174	Macrophage migration inhibitory factor	MIF	12.476	18.418	17.4	2	10	2	0.994	1.268	1.105	1.064	0.845	1.099	0.962	0.892	0.96	0.86	0.82
P29218	Inositol monophosphatase 1 OS=Homo s	IMPA1	30.188	7.1869	4	1	2	1	1.105	0.984	1.02	0.983	0.847	0.938	1.156	0.956	0.96	0.85	1.01
Q9GZU8	Protein FAM192A OS=Homo sapiens O	FAM192A	28.912	11.872	11	2	2	2	1.005	1.034	0.926	1.029	0.955	0.991	1.04	1.058	0.96	0.95	1.03
Q5T0Z8	Uncharacterized protein C6orf132 OS=H	C6orf132	124.03	8.0074	0.9	1	1	1	0.975	0.964	0.92	0.939	1.057	0.868	1.286	1.113	0.96	0.99	1.24
Q96C23	Aldose 1-epimerase OS=Homo sapiens C	GALM	37.765	50.27	24	6	7	6	1.035	1.097	1.07	0.974	0.915	0.931	1.072	0.993	0.96	0.87	0.97
A1X283	SH3 and PX domain-containing protein 2	SH3PXD2B	101.58	84.306	14.9	12	15	12	1.01	1.025	0.987	0.964	0.99	0.948	1.107	1.016	0.96	0.95	1.04
Q9Y2Z4	Tyrosine--tRNA ligase, mitochondrial OS	YARS2	53.198	60.014	13.2	6	9	6	1.047	0.987	0.981	0.969	1.015	0.969	0.997	0.98	0.96	0.98	0.97
P67775	Serine/threonine-protein phosphatase 2A	PPP2CA	35.594	138.96	44.7	10	19	1	1.031	1.073	0.991	1.026	0.889	0.964	0.917	1.012	0.96	0.88	0.92
Q9Y244	Proteasome maturation protein OS=Hom	POMP	15.789	10.802	12.8	2	2	2	1.139	0.648	0.716	0.997	1.153	0.876	1.167	1.256	0.96	1.14	1.36
O00203	AP-3 complex subunit beta-1 OS=Homo	AP3B1	121.32	159.93	18.1	16	22	16	1.046	1.027	0.99	0.997	1.002	0.913	0.987	0.987	0.96	0.92	0.95
O15400	Syntaxin-7 OS=Homo sapiens OX=9606	STX7	29.815	65.265	27.6	6	6	6	1.08	1.04	1.036	0.996	0.9	0.984	1.012	0.959	0.96	0.89	0.93
Q8N3Y3	LARGE xylosyl- and glucuronyltransfera	LARGE2	81.786	13.209	3.1	2	2	2	0.832	1.173	1.021	0.9	1.008	1.051	1.243	1.039	0.96	1.03	1.14
P22087	rRNA 2'-O-methyltransferase fibrillarin	FBL	33.784	116.63	36.4	11	21	10	1.036	0.966	0.955	0.963	1.046	0.978	1.033	1.058	0.96	1.01	1.04
Q8TDY4	Arf-GAP with SH3 domain, ANK repeat	ASAP3	99.154	9.4845	2.3	1	2	1	0.991	1.056	0.968	0.993	1.048	0.945	1.045	1.025	0.96	0.97	1.01
P15104	Glutamine synthetase OS=Homo sapiens	GLUL	42.064	33.051	13.1	5	5	5	1.022	1.084	1.002	1.015	0.994	0.909	1.088	0.906	0.96	0.90	0.95
Q15750	TGF-beta-activated kinase 1 and MAP3K	TAB1	54.643	53.607	15.3	6	8	6	1.031	0.979	0.902	1.023	1.016	1.194	0.885	1.009	0.96	1.10	0.94
Q12768	WASH complex subunit 5 OS=Homo sa	WASHC5	134.28	46.847	6.6	7	7	7	1.022	1.057	1.012	0.979	0.983	0.953	1.033	1.022	0.96	0.93	0.99
P46977	Dolichyl-diphosphooligosaccharide--prot	STT3A	80.529	59.751	10.8	8	10	8	1.04	1.034	0.993	0.993	1.045	1.078	0.947	0.985	0.96	1.02	0.93
Q16864	V-type proton ATPase subunit F OS=Ho	ATP6V1F	13.37	41.544	47.9	5	7	5	1.016	1.054	0.961	1.021	0.964	1.218	0.874	0.993	0.96	1.05	0.90
Q5T0D9	Tumor protein p63-regulated gene 1-like	TPRG1L	30.211	8.1976	4	1	1	1	1.049	1.02	0.976	1.005	0.934	1.044	1.018	0.97	0.96	0.96	0.96
Q53SF7	Cordon-bleu protein-like 1 OS=Homo sa	COBLL1	131.79	61.982	8.7	7	7	7	1.059	0.962	0.984	0.951	1.044	0.895	1.012	1.065	0.96	0.96	1.03
P61011	Signal recognition particle 54 kDa protei	SRP54	55.704	129.51	33.5	13	19	12	1.036	1.008	0.989	0.968	0.98	0.973	0.983	0.957	0.96	0.96	0.95
Q8NCN5	Pyruvate dehydrogenase phosphatase reg	PDPR	99.363	28.492	5.2	4	4	4	1.003	1.04	0.96	0.996	0.964	1.229	0.841	1.036	0.96	1.07	0.92
Q9H8S9	MOB kinase activator 1A OS=Homo sap	MOB1A	25.079	36.217	22.2	4	5	4	1.105	1.017	1.1	0.931	0.931	1.202	0.779	0.883	0.96	1.01	0.78
Q9Y5A9	YTH domain-containing family protein 2	YTHDF2	62.333	92.213	20.7	10	13	7	1.059	1.013	0.981	1.002	1.06	0.96	0.951	0.976	0.96	0.97	0.93
Q9Y2R5	28S ribosomal protein S17, mitochondria	MRPS17	14.502	33.238	30	4	8	4	1.085	0.917	0.981	0.935	1.083	1.188	0.829	1.017	0.96	1.13	0.92
Q9Y333	U6 snRNA-associated Sm-like protein L	LSM2	10.834	42.298	40	3	4	3	0.902	1.169	0.945	1.037	0.964	1.256	0.823	1.174	0.96	1.07	0.96

P21796	Voltage-dependent anion-selective chann	VDAC1	30.772	323.31	64.7	15	41	15	1.029	1.065	0.999	1.005	0.933	0.979	0.975	1.009	0.96	0.91	0.95
Q9NVA2	Septin-11 OS=Homo sapiens OX=9606 (	SEPT11	49.398	164.82	31.5	11	24	4	1.038	1.047	1	0.995	0.958	0.927	1.011	0.986	0.96	0.90	0.96
A8CG34	Nuclear envelope pore membrane protein	POM121C	125.09	6.1139	5.6	5	7	1	1.036	0.947	0.916	0.981	1.045	1.034	0.993	1.096	0.96	1.05	1.05
P61160	Actin-related protein 2 OS=Homo sapien	ACTR2	44.76	87.161	16	6	17	6	1.036	1.077	1.014	1.007	0.955	1.017	0.878	0.935	0.96	0.93	0.86
P80297	Metallothionein-1X OS=Homo sapiens (	MT1X	6.0683	6.4826	34.4	3	1	1	1.194	0.94	0.922	1.119	0.868	0.719	1.063	0.991	0.96	0.74	0.96
Q9BTE1	Dynactin subunit 5 OS=Homo sapiens O.	DCTN5	20.126	22.892	25.3	4	4	3	1.044	1.019	0.969	1.004	0.978	0.999	0.993	1.081	0.96	0.96	1.01
P34897	Serine hydroxymethyltransferase, mitoch	SHMT2	55.992	133.03	37.5	16	26	15	1.001	1.016	0.947	0.982	1.015	1.011	1.061	1.056	0.96	1.00	1.05
O60287	Nucleolar pre-ribosomal-associated prote	URB1	254.39	81.298	6.8	12	13	12	0.977	1.012	0.934	0.968	1.06	1.219	0.913	1.032	0.96	1.15	0.98
P03891	NADH-ubiquinone oxidoreductase chain	MT-ND2	38.96	6.551	2.6	1	1	1	0.957	0.986	1.007	0.851	1.145	0.978	1.23	1.047	0.96	1.09	1.17
Q96AN5	Transmembrane protein 143 OS=Homo s	TMEM143	51.714	7.6038	3.1	1	1	1	0.791	1.555	1.188	1.055	0.547	1.67	0.63	0.764	0.96	0.95	0.59
Q8WUA7	TBC1 domain family member 22A OS=H	TBC1D22A	59.12	18.276	5.2	3	3	2	1.068	1.002	0.989	0.99	0.986	0.937	1.009	1.036	0.96	0.93	0.99
Q16630	Cleavage and polyadenylation specificity	CPSF6	59.209	113.29	22	11	20	11	1.029	1.061	0.988	1.01	0.976	0.968	0.897	0.982	0.96	0.93	0.90
Q53RE8	Ankyrin repeat domain-containing protei	ANKRD39	19.651	9.059	10.9	1	1	1	0.914	1.072	1.012	0.886	1.029	1.372	0.8	1.141	0.96	1.21	0.98
Q9BVT8	Transmembrane and ubiquitin-like domai	TMUB1	26.261	24.244	20.7	3	3	3	1.042	1.009	0.977	0.983	0.986	1.287	0.82	0.945	0.96	1.11	0.86
Q96PK6	RNA-binding protein 14 OS=Homo sapie	RBM14	69.491	216.16	34.5	22	33	21	0.966	1.059	0.997	0.938	1.005	1.089	0.963	0.993	0.96	1.03	0.97
Q86U38	Nucleolar protein 9 OS=Homo sapiens O	NOP9	69.437	80.731	18.6	11	13	11	1.009	0.97	0.894	0.997	0.985	1.027	0.879	1.066	0.96	1.02	0.98
P04080	Cystatin-B OS=Homo sapiens OX=9606	CSTB	11.139	34.246	58.2	4	10	3	0.99	1.076	0.937	1.037	0.928	0.979	1.022	1.029	0.96	0.92	0.99
O75164	Lysine-specific demethylase 4A OS=Hon	KDM4A	120.66	7.8767	1.1	1	1	1	0.973	1.137	0.918	1.098	0.886	0.883	0.991	1.13	0.96	0.84	1.01
Q8IVD9	NudC domain-containing protein 3 OS=I	NUDCD3	40.822	23.771	11.4	4	4	4	1.061	1.091	1.069	0.987	0.916	0.972	0.963	0.966	0.96	0.88	0.90
Q9NYR9	NF-kappa-B inhibitor-interacting Ras-lik	NKIRAS2	21.508	11.484	6.3	1	2	1	1.106	1.189	1.226	0.966	0.794	1.084	0.807	0.837	0.96	0.82	0.72
Q8WWY3	U4/U6 small nuclear ribonucleoprotein P	PRPF31	55.455	78.724	18.2	7	13	7	1.064	0.985	0.994	0.963	1.02	1.066	0.949	0.951	0.96	1.02	0.93
Q99808	Equilibrative nucleoside transporter 1 OS	SLC29A1	50.219	52.943	14.9	6	8	6	1.001	0.975	0.957	0.93	1.073	1.08	1.036	0.998	0.95	1.09	1.03
Q04760	Lactoylglutathione lyase OS=Homo sapie	GLO1	20.777	31.514	23.9	4	8	4	1.029	1.033	1.024	0.945	0.937	1.191	0.94	0.957	0.95	1.03	0.92
O00154	Cytosolic acyl coenzyme A thioester hydı	ACOT7	41.796	63.39	22.6	6	11	6	0.956	1.125	1.012	0.975	0.932	0.997	0.972	1.05	0.95	0.93	0.97
P11717	Cation-independent mannose-6-phosphat	IGF2R	274.37	323.31	21.9	44	57	43	1.026	1.053	1.001	0.984	0.966	1.066	0.939	0.951	0.95	0.98	0.91
O94901	SUN domain-containing protein 1 OS=H	SUN1	90.063	48.254	9.4	6	6	6	0.999	0.968	0.896	0.982	1.05	1.036	1.046	1.073	0.95	1.06	1.08
P63010	AP-2 complex subunit beta OS=Homo sa	AP2B1	104.55	264.71	29.9	27	49	11	1.043	1.033	0.973	1.009	0.958	0.99	0.979	0.981	0.95	0.94	0.94
O95379	Tumor necrosis factor alpha-induced prot	TNFAIP8	23.003	14.302	11.1	2	2	2	1.098	0.82	0.802	1.029	1.139	0.848	1.061	1.164	0.95	1.04	1.16
O00571	ATP-dependent RNA helicase DDX3X (	DDX3X	73.243	233.56	44.4	23	46	7	0.999	1.094	0.991	1.007	0.96	1.048	0.91	0.946	0.95	0.96	0.89
Q7Z5G4	Golgin subfamily A member 7 OS=Homo	GOLGA7	15.824	19.475	24.8	3	5	3	1.037	0.964	0.971	0.939	1.062	1.136	0.91	1.102	0.95	1.10	1.01
P57088	Transmembrane protein 33 OS=Homo sa	TMEM33	27.978	23.423	16.2	4	5	3	1.046	1.038	0.995	0.994	1	0.99	0.979	0.939	0.95	0.95	0.92
Q9BQL6	Fermitin family homolog 1 OS=Homo sa	FERMT1	77.436	25.359	8.4	4	5	3	1.024	1.035	0.989	0.976	0.968	0.951	1.039	1.083	0.95	0.93	1.03
Q02790	Peptidyl-prolyl cis-trans isomerase FKBI	FKBP4	51.804	222.29	50.8	21	35	20	1.031	1.137	1.069	1	0.863	1.04	0.862	0.893	0.95	0.88	0.81
Q9H7L9	Sin3 histone deacetylase corepressor con	SUDS3	38.136	14.418	5.8	2	3	2	1.087	0.967	0.996	0.964	0.96	0.97	1.08	0.992	0.95	0.94	1.01
Q16181	Septin-7 OS=Homo sapiens OX=9606 G	SEPT7	50.679	232.22	46.7	19	33	19	1.035	1.097	1.022	1.012	0.943	1.03	0.936	0.946	0.95	0.93	0.88
P13051	Uracil-DNA glycosylase OS=Homo sapie	UNG	34.645	36.515	7.7	1	2	1	0.975	0.828	0.822	0.898	1.129	1.009	1.242	1.255	0.95	1.19	1.38
Q9NQW7	Xaa-Pro aminopeptidase 1 OS=Homo sa	XPNPEP1	69.917	86.133	18.6	10	12	9	1.012	1.008	0.99	0.937	0.984	0.964	1.188	1.039	0.95	0.96	1.10
Q86YP4	Transcriptional repressor p66-alpha OS=	GATAD2A	68.062	76.343	20.1	11	13	10	1.003	1.082	1.007	0.982	0.991	1.209	0.83	0.934	0.95	1.06	0.85
P15260	Interferon gamma receptor 1 OS=Homo s	IFNGR1	54.404	7.0399	2.2	1	2	1	1.057	0.894	0.909	0.952	1.089	1.091	1.046	1.01	0.95	1.12	1.05
P62495	Eukaryotic peptide chain release factor su	ETF1	49.03	110.79	33.2	13	23	12	1.05	1.073	1.005	1.02	0.965	0.964	0.947	0.983	0.95	0.91	0.91
Q6P996	Pyridoxal-dependent decarboxylase doma	PDXDC1	86.706	119.85	18.9	13	17	13	1.033	1.045	0.999	0.983	1.054	1.021	0.986	0.953	0.95	1.00	0.93
P41227	N-alpha-acetyltransferase 10 OS=Homo s	NAA10	26.458	67.334	28.9	8	13	8	1.025	1.096	1.034	0.989	0.933	1.194	0.854	1.042	0.95	1.00	0.89
O95202	Mitochondrial proton/calcium exchanger	LETM1	83.353	157.51	17.9	10	15	10	1.052	1.038	1.001	0.992	1.004	1.163	0.866	0.933	0.95	1.04	0.86
O15511	Actin-related protein 2/3 complex subuni	ARPC5	16.32	38.694	39.1	5	7	4	1.036	1.029	1.043	0.926	0.983	1.084	0.828	0.976	0.95	1.00	0.87
Q9BXY0	Protein MAK16 homolog OS=Homo sapi	MAK16	35.368	41.385	19	5	5	5	1.046	0.97	0.967	0.955	1.141	0.971	0.993	1.013	0.95	1.05	1.00
O94953	Lysine-specific demethylase 4B OS=Hon	KDM4B	121.9	12.032	2.7	2	2	2	1.021	0.823	0.7	1.058	1.092	0.891	0.984	1.448	0.95	1.08	1.32
Q8TD55	Pleckstrin homology domain-containing f	PLEKHO2	53.349	76.22	21.8	9	10	9	0.934	0.886	0.751	0.984	1.103	1.104	1.001	1.085	0.95	1.21	1.15
P82664	28S ribosomal protein S10, mitochondria	MRPS10	22.999	14.593	11.4	2	2	2	1.095	0.896	0.965	0.933	1.097	0.855	1.189	0.998	0.95	0.98	1.10
Q12797	Aspartyl/asparaginyl beta-hydroxylase O	ASPH	85.862	125.78	16	10	14	10	0.998	1.053	0.91	1.045	0.963	0.878	1.033	1.045	0.95	0.90	1.01
Q9Y4Z0	U6 snRNA-associated Sm-like protein LS	LSM4	15.35	15.835	15.8	2	4	2	0.992	1.1	0.991	1.003	0.913	0.993	1.088	1.034	0.95	0.91	1.01
Q96PU5	E3 ubiquitin-protein ligase NEDD4-like	NEDD4L	111.93	10.007	1	1	2	1	0.926	1.014	0.844	1.005	1.14	0.998	1.003	1.19	0.95	1.10	1.13
Q9P275	Ubiquitin carboxyl-terminal hydrolase 36	USP36	122.91	30.909	3.5	3	4	3	0.964	0.974	0.884	0.963	1.1	0.979	1.126	1.126	0.95	1.07	1.16
P00519	Tyrosine-protein kinase ABL1 OS=Homo	ABL1	122.87	11.036	2.6	2	2	2	1.09	0.927	0.996	0.926	1.123	0.916	1.025	1.039	0.95	1.01	1.02
Q8NBS9	Thioredoxin domain-containing protein 5	TXNDC5	47.628	105.12	26.4	11	19	11	1.019	1.081	1.028	0.973	0.92	1.233	0.828	0.977	0.95	1.03	0.86
P56385	ATP synthase subunit e, mitochondrial O	ATP5ME	7.9331	24.052	31.9	3	6	3	1.063	0.99	0.984	0.972	1.1	1.242	0.766	1.038	0.95	1.14	0.88
P23229	Integrin alpha-6 OS=Homo sapiens OX=	ITGA6	126.6	249.64	26	22	35	22	1.046	1.004	0.95	1.003	0.967	0.951	1.035	1.082	0.95	0.94	1.03
Q2Q1W2	E3 ubiquitin-protein ligase TRIM71 OS=	TRIM71	93.384	89.825	20.2	13	16	13	1.059	1.012	1.002	0.971	0.957	0.956	1.074	1.03	0.95	0.92	1.02
Q9H9Q2	COP9 signalosome complex subunit 7b (	COPS7B	29.622	42.99	20.1	4	5	4	1.042	1.134	1.065	1.008	0.92	1.107	0.842	0.961	0.95	0.93	0.83
Q13185	Chromobox protein homolog 3 OS=Hom	CBX3	20.811	87.52	36.6	6	19	4	1.028	0.987	0.968	0.951	1.045	1.079	0.952	1	0.95	1.05	0.97
P82970	High mobility group nucleosome-binding	HMGN5	31.524	23.911	8.5	2	2	2	0.991	1.169	1.06	0.997	0.829	0.964	1.102	0.96	0.95	0.83	0.95
Q3ZAQ7	Vacuolar ATPase assembly integral meml	VMA21	11.354	6.5367	11.9	1	1	1	0.951	0.871	0.793	0.942	1.275	0.995	1.345	0.952	0.95	1.25	1.26
Q9Y2X9	Zinc finger protein 281 OS=Homo sapier	ZNF281	96.914	60.175	10.5	7	10	7	1.105	0.981	0.984	1.002	1.005	0.961	0.973	0.889	0.95	0.94	0.89
Q08AD1	Calmodulin-regulated spectrin-associated	CAMSAP2	168.09	83.2	9.1	11	13	11	1.024	1.02	0.961	0.985	0.985	1.004	0.971	0.993	0.95	0.97	0.96
Q13347	Eukaryotic translation initiation factor 3	EIF3I	36.501	114.6	44.9	12	19	12	0.937	1.315	1.138	1.006	0.713	1.33	0.818	0.808	0.95	0.91	0.72
Q9UJ83	2-hydroxyacyl-CoA lyase 1 OS=Homo sa	HACL1	63.728	79.374	18.2	9	11	9	1.004	1.163	1.052	1.011	0.922	0.974	0.769	0.92	0.95	0.87	0.78
P09622	Dihydrolipoyl dehydrogenase, mitochond	DLD	54.177	100.06	26.3	11	17	11	1.01	0.989	0.954	0.949	1.075	0.994	1.078	1.02	0.95	1.04	1.05
Q9GZZ1	N-alpha-acetyltransferase 50 OS=Homo s	NAA50	19.398	68.95	44.4	9	12	9	1.064	1.098	1.119	0.939	0.962	1.288	0.746	0.926	0.95	1.04	0.77
C9JLW8	Mapk-regulated corepressor-interacting p	MCRIP1	10.92	15.192	27.8	2	2	2	0.717	1.527	1.153	0.983	0.731	1.517	0.766	0.919	0.95	1.00	0.75
Q9H2K8	Serine/threonine-protein kinase TAO3 O	TAOK3	105.4	32.951	6.1	5	5	5	0.934	1.164	1.006	0.991	0.855	1.307	0.876	0.996	0.95	1.03	0.89
Q9NWT1	p21-activated protein kinase-interacting p	PAK1IP1	43.963	39.289	15.6	6	8	6	0.985	1.009	0.964	0.934	1.004	0.991	1.159	1.047	0.95	1.00	1.11
O60925	Prefoldin subunit 1 OS=Homo sapiens O	PFDN1	14.21	21.881	23.8	3	8	3	0.982	1.094	0.996	0.98	0.986	1.068	0.928	0.979	0.95	0.99	0.92
P49459	Ubiquitin-conjugating enzyme E2 A OS=	UBE2A	17.315	14.699	17.8	2	2	2	1.026	1.024	0.957	0.994	0.967	1.022	1.017	1.039	0.95	0.97	1.00
Q5T2T1	MAGUK p55 subfamily member 7 OS=H	MPP7	65.523	86.132	14.4	6	7	6	1.028	1.042	0.993	0.977	0.955	1.048	0.998	0.985	0.95	0.97	0.96
P21333	Filamin-A OS=Homo sapiens OX=9606	FLNA	280.74	323.31	43.9	88	166	81	1.028	1.103	1.014	1.014	0.857	0.886	1.12	1.023	0.95	0.82	1.01
P51532	Transcription activator BRG1 OS=Homo	SMARCA4	184.64	80.069	10.1	15	18	8	1.078	1.008	1.042	0.943	1	0.839	1.17	0.921	0.95	0.88	1.00
Q9BXJ9	N-alpha-acetyltransferase 15, NatA auxil	NAA15	101.27	138.13	19.4	16	23	16	1.043	1.059	1.005	0.995	0.987	0.983	0.966	0.952	0.95	0.94	0.91
Q5JRA6	Transport and Golgi organization protein	MIA3	213.7	133.75	10.4	16	16	16	1.02	1.081	1.019	0.98	0.979	0.946	0.978	0.891	0.95	0.92	0.89
P35222	Catenin beta-1 OS=Homo sapiens OX=9	CTNNB1	85.496	177.41	31.8	18	41	14	1.056	0.983	0.944	0.996	1.003	0.861	1.106	1.021	0.95	0.91	1.04
O96011	Peroxisomal membrane protein 11B OS=	PEX11B	28.431	12.919	7.3	2	2	2	0.959	1.057	0.915	1.003	0.936	1.131	0.95	1.151	0.95	1.03	1.04
P19174	1-phosphatidylinositol 4,5-bisphosphate	PLCG1	148.53	104.65	11.3	12	15	12	1.048	1.007	0.977	0.978	1.029	1.053	1.007	0.997	0.95	1.01	0.98
P62487	DNA-directed RNA polymerase II subuni	POLR2G	19.294	49.454	36.6	5	8	5	1.037	1.093	1.072	0.954	0.995	1.053	0.945	0.894	0.95	0.96	0.86
Q8WVB6	Chromosome transmission fidelity protei	CHTF18	107.38	83.637	10.7	9	10	9	0.966	1.019	0.909	0.979	1.103	1.058	0.967	1.098	0.95	1.09	1.04
Q9NQC3	Reticulon-4 OS=Homo sapiens OX=960(	RTN4	129.93	60.945	8.2	7	14	7	0.988	1.157	1.058	0.982	0.848	0.975	0.927	0.945	0.95	0.85	0.87
Q9BQI0	Allograft inflammatory factor 1-like OS=	AIF1L	17.068	21.806	21.3	3	6	3	0.989	1.094	0.982	0.999	1.044	1.266	0.826	0.931	0.95	1.11	0.84
Q9NY12	H/ACA ribonucleoprotein complex subur	GAR1	22.348	11.321	8.3	1	2	1	0.952	1.027	0.864	1.018	0.98	1.017	1.141	1.086	0.95	1.01	1.13
Q9HCI7	E3 ubiquitin-protein ligase MSL2 OS=H	MSL2	62.54	19.574	5.9	3	3	3	1.038	1.043	0.972	1.007	0.984	0.868	1.095	1.017	0.95	0.89	1.01
P51970	NADH dehydrogenase [ubiquinone] 1 alp	NDUFA8	20.105	42.88	29.7	5	6	5	1.072	0.863	0.884	0.956	1.124	1.126	0.803	1.166	0.95	1.16	1.02
P24539	ATP synthase F(0) complex subunit B1,	ATP5F1	28.908	63.419	27.3	7	11	7	0.865	1.287	1.065	0.981	0.893	1.375	0.749	0.862	0.95	1.05	0.75
Q8TCD1	UPF0729 protein C18orf32 OS=Homo sa	C18orf32	8.6693	6.6608	13.2	1	1	1	1.057	0.993	0.975	0.974	1.049	1.189	0.794	1.009	0.95	1.09	0.88
Q8N2H4	Protein SYS1 homolog OS=Homo sapier	SYS1	17.615	6.7541	5.8	1	2	1	0.968	0.997	0.888	0.98	1.047	1.077	1.021	1.126	0.95	1.08	1.09
Q9H0C8	Integrin-linked kinase-associated serine/t	ILKAP	42.906	68.363	24	9	10	9	1.082	1.057	1.072	0.961	0.97	0.991	0.955	0.944	0.95	0.92	0.89
Q96A65	Exocyst complex component 4 OS=Homo	EXOC4	110.5	119.27	14.3	12	13	12	1.052	1.022	0.983	0.988	0.962	0.915	1.127	0.99	0.95	0.91	1.02
O43318	Mitogen-activated protein kinase kinase k	MAP3K7	67.195	6.9674	3.3	1	1	1	1.073	1.019	0.985	1.003	1.01	0.924	0.928	1.063	0.95	0.92	0.95
P40855	Peroxisomal biogenesis factor 19 OS=Hc	PEX19	32.806	72.097	25.8	6	6	6	1.056	1.034	0.981	1.005	0.907	0.955	1.15	1.012	0.95	0.89	1.03
Q9GZM7	Tubulointerstitial nephritis antigen-like (	TINAGL1	52.387	20.201	7.7	3	3	3	1.077	0.992	1.017	0.949	0.887	0.982	1.08	1.03	0.95	0.90	1.02
Q9H501	ESF1 homolog OS=Homo sapiens OX=9	ESF1	98.795	49.242	8	6	7	6	0.996	1.027	0.954	0.968	1.078	1.15	0.872	1.072	0.95	1.10	0.96
Q9UBQ0	Vacuolar protein sorting-associated prote	VPS29	20.505	44.395	29.7	6	7	6	1.079	0.943	0.959	0.962	1.007	1.023	0.992	1.001	0.95	1.00	0.99
O95819	Mitogen-activated protein kinase kinase k	MAP4K4	142.1	38.879	7.3	8	9	6	1.028	1.132	1.07	0.982	1.07	1.206	0.725	0.838	0.95	1.05	0.72
Q99613	Eukaryotic translation initiation factor 3	EIF3C	105.34	157.66	24	17	29	17	1.012	1.104	1.024	0.986	0.937	1.09	0.913	0.93	0.95	0.96	0.87
Q0PNE2	Elongator complex protein 6 OS=Homo s	ELP6	29.793	12.757	7.5	2	2	2	0.987	1.009	0.884	1.012	1.098	1.227	0.848	0.997	0.95	1.16	0.92
O43278	Kunitz-type protease inhibitor 1 OS=Hor	SPINT1	58.397	71.523	21.2	10	13	10	0.982	0.973	0.87	0.987	1.052	1.219	0.879	1.067	0.95	1.16	1.00
P49643	DNA primase large subunit OS=Homo sa	PRIM2	58.805	29.779	8.8	4	5	4	0.969	1.001	0.905	0.966	1.052	1.161	1	1.056	0.95	1.12	1.04
Q8NFH4	Nucleoporin Nup37 OS=Homo sapiens (	NUP37	36.707	62.216	26.7	7	9	7	1.019	1.05	0.944	1.021	0.978	1.011	0.993	0.981	0.95	0.96	0.95
Q9H4L7	SWI/SNF-related matrix-associated actin	SMARCAD	117.4	46.041	5.9	5	5	5	1.024	1.063	0.955	1.027	1.037	0.969	0.949	0.986	0.95	0.96	0.93
P05165	Propionyl-CoA carboxylase alpha chain,	PCCA	80.058	24.008	6.9	4	4	4	1.073	0.994	0.96	1.003	0.981	1.068	0.94	0.982	0.95	0.99	0.93
O95602	DNA-directed RNA polymerase I subunit	POLR1A	194.81	177.16	15.1	18	23	18	1.001	1.023	0.958	0.964	1.042	1.032	0.988	1.046	0.95	1.02	1.00
Q8WUH1	Protein Churchill OS=Homo sapiens OX	CHURC1	16.111	14.195	15.1	2	3	2	1.03	0.993	0.957	0.964	1.007	1.275	0.76	1.088	0.95	1.13	0.91
Q92922	SWI/SNF complex subunit SMARCC1 (	SMARCC1	122.87	160.1	21	20	33	13	1.069	1.049	1.029	0.982	0.952	0.893	1.079	0.955	0.95	0.87	0.96
Q13573	SNW domain-containing protein 1 OS=H	SNW1	61.494	96.613	26.9	11	16	11	1.03	0.989	0.943	0.974	1.036	0.982	0.978	1.034	0.95	1.00	1.00
O00442	RNA 3'-terminal phosphate cyclase OS=I	RTCA	39.336	42.776	9	2	2	2	1.072	1	1.011	0.956	1.007	0.957	0.978	1.058	0.95	0.95	0.98
P78347	General transcription factor II-I OS=Hom	GTF2I	112.42	85.197	13.3	11	19	11	0.996	0.957	0.903	0.951	1.065	1.023	1.045	1.1	0.95	1.07	1.10
P04198	N-myc proto-oncogene protein OS=Homo	MYCN	49.561	38.761	12.9	3	4	3	1.057	1.088	1.19	0.846	0.98	1.042	0.978	0.952	0.95	0.94	0.90
O95229	ZW10 interactor OS=Homo sapiens OX=	ZWINT	31.293	37.504	28.2	5	6	5	1.029	0.938	0.969	0.898	1.124	1.134	1.02	0.998	0.95	1.15	1.03
P30533	Alpha-2-macroglobulin receptor-associat	LRPAP1	41.465	123.73	38.1	13	18	13	1.077	1.04	1.011	0.998	0.97	1.152	0.79	0.902	0.95	1.00	0.80
P46087	Probable 28S rRNA (cytosine(4447)-C(5	NOP2	89.301	201.58	28	19	27	19	0.963	0.949	0.858	0.956	1.124	1.136	1.009	1.155	0.95	1.18	1.13
Q9Y3C8	Ubiquitin-fold modifier-conjugating enzy	UFC1	19.458	11.734	11.4	2	3	2	1.038	1.024	0.974	0.982	1.074	1.049	0.951	0.949	0.95	1.03	0.92
Q86U70	LIM domain-binding protein 1 OS=Homo	LDB1	46.532	21.639	10.9	3	3	3	1.006	0.88	0.789	1	1.082	0.928	1.138	1.236	0.95	1.07	1.26
Q9H6S0	3'-5' RNA helicase YTHDC2 OS=Homo	YTHDC2	160.25	44.026	5.7	7	8	7	0.989	1.032	0.951	0.966	1.022	0.977	1.017	1.08	0.95	0.99	1.04
P05023	Sodium/potassium-transporting ATPase s	ATP1A1	112.89	323.31	39.7	33	76	33	1.041	1.054	0.997	0.99	0.964	1.018	0.94	0.956	0.95	0.95	0.91
Q9BV44	THUMP domain-containing protein 3 OS	THUMPD3	57.002	45.771	13.8	7	8	7	0.997	1.058	0.963	0.986	0.962	1.243	0.804	1.012	0.95	1.07	0.88
O75787	Renin receptor OS=Homo sapiens OX=9	ATP6AP2	39.008	42.259	14.9	4	5	3	1.098	0.956	1.022	0.926	1.005	0.875	1.221	0.999	0.95	0.92	1.08
Q8WWH5	Probable tRNA pseudouridine synthase 1	TRUB1	37.252	28.684	13.8	4	4	4	1.047	1.077	1.04	0.974	1.069	0.804	1.158	0.864	0.95	0.88	0.95
O43824	Putative GTP-binding protein 6 OS=Hom	GTPBP6	56.897	48.177	14.9	7	7	7	0.922	1.279	1.113	0.974	0.939	1.186	0.863	0.823	0.95	0.97	0.77
O14828	Secretory carrier-associated membrane pr	SCAMP3	38.287	42.056	24.5	5	6	5	1.062	1.02	0.961	1.013	1.02	1.003	1.032	1.074	0.95	0.97	1.01
P22307	Non-specific lipid-transfer protein OS=H	SCP2	58.993	79.288	19.2	10	16	10	1.035	1.007	0.975	0.961	1.022	0.982	0.963	1.019	0.95	0.98	0.97
P19338	Nucleolin OS=Homo sapiens OX=9606 (	NCL	76.613	273.85	32.4	27	58	27	1.035	1.026	0.939	1.015	0.989	1.149	0.878	0.988	0.95	1.04	0.91
Q9Y520	Protein PRRC2C OS=Homo sapiens OX	PRRC2C	316.91	217.78	12.5	29	34	29	1.036	1.078	1.009	0.995	0.93	1.097	0.855	0.984	0.95	0.96	0.87
Q9NWU5	39S ribosomal protein L22, mitochondria	MRPL22	23.64	60.704	34.5	7	9	7	1.016	0.981	0.878	1.015	1.013	1.212	0.858	1.126	0.95	1.11	0.99
Q6P1A2	Lysophospholipid acyltransferase 5 OS=I	LPCAT3	56.034	33.874	10.7	5	6	5	1.068	0.98	1.04	0.901	1.098	0.939	1.096	0.954	0.95	0.99	1.00
P48059	LIM and senescent cell antigen-like-conta	LIMS1	37.251	58.005	26.8	7	7	7	1.081	0.983	0.982	0.974	1.043	0.931	1.048	1.082	0.95	0.96	1.03
Q05048	Cleavage stimulation factor subunit 1 OS	CSTF1	48.357	95.748	27.6	8	9	8	1.024	1.076	0.994	0.996	0.965	1.221	0.829	0.998	0.95	1.04	0.87
P04844	Dolichyl-diphosphooligosaccharide--prot	RPN2	69.283	86.663	17.6	8	16	8	1.034	1.039	0.981	0.983	0.972	0.989	1.019	0.971	0.95	0.95	0.96
Q96CB9	5-methylcytosine rRNA methyltransferas	NSUN4	43.088	13.822	5.7	2	3	2	0.996	1.019	0.933	0.976	1.043	1.042	1.017	1.058	0.95	1.03	1.03
Q9BVW5	TIMELESS-interacting protein OS=Hom	TIPIN	34.555	26.797	12.3	4	4	4	0.968	0.778	0.672	0.982	1.347	1.182	0.878	1.296	0.95	1.45	1.25
Q13825	Methylglutaconyl-CoA hydratase, mitoch	AUH	35.608	46.059	5.9	1	1	1	0.942	1.065	1.007	0.894	1.087	1.001	1.156	1.031	0.95	1.04	1.09
P61956	Small ubiquitin-related modifier 2 OS=H	SUMO2	10.871	6.0493	27.4	2	4	1	1.008	1.207	1.068	1.03	0.852	1.174	0.817	0.88	0.95	0.91	0.77
Q7L0Y3	tRNA methyltransferase 10 homolog C O	TRMT10C	47.346	121.53	42.9	14	16	13	0.993	1.044	0.994	0.935	1.039	1.238	0.909	0.969	0.95	1.12	0.92
Q9UGR2	Zinc finger CCCH domain-containing pro	ZC3H7B	111.58	21.44	3.4	3	3	3	1.053	0.984	0.969	0.96	1.087	1.262	0.771	0.966	0.95	1.15	0.85
P20645	Cation-dependent mannose-6-phosphate	M6PR	30.993	37.691	11.6	3	4	3	1.107	1.005	1.013	0.987	1.022	1.226	0.693	0.937	0.95	1.06	0.77
Q15006	ER membrane protein complex subunit 2	EMC2	34.833	15.165	7.4	2	2	2	0.963	1.13	1.012	0.97	0.878	0.928	1.204	1.029	0.95	0.86	1.07
P24928	DNA-directed RNA polymerase II subuni	POLR2A	217.17	167.86	13.2	22	28	22	1.007	1.027	0.938	0.988	1.046	0.976	1.005	0.977	0.95	0.99	0.97
O95251	Histone acetyltransferase KAT7 OS=Hon	KAT7	70.642	14.236	3.8	2	2	2	0.991	1.001	0.828	1.058	0.935	0.973	1.158	1.079	0.95	0.96	1.12
O43181	NADH dehydrogenase [ubiquinone] iron-	NDUFS4	20.108	10.157	8.6	1	1	1	1.022	1.004	0.954	0.964	1.045	1.027	0.974	1.083	0.95	1.02	1.02
P62826	GTP-binding nuclear protein Ran OS=Hc	RAN	24.423	40.811	25.5	6	10	5	1.057	1.018	0.973	0.991	0.991	1.027	0.954	1.002	0.95	0.97	0.94
O00567	Nucleolar protein 56 OS=Homo sapiens (	NOP56	66.049	166.93	30.6	14	23	14	0.995	1.042	0.968	0.96	0.981	1.035	1.098	1.018	0.95	0.99	1.04
Q13907	Isopentenyl-diphosphate Delta-isomerase	IDI1	26.319	43.932	19.8	5	7	5	0.953	0.803	0.751	0.911	1.326	1.291	0.872	1.196	0.95	1.49	1.18
Q8ND30	Liprin-beta-2 OS=Homo sapiens OX=96(	PPFIBP2	98.543	68.262	14.4	10	11	8	1.004	1.059	0.935	1.017	0.926	0.892	1.081	1.112	0.95	0.88	1.06
Q8IVL5	Prolyl 3-hydroxylase 2 OS=Homo sapien	P3H2	80.984	40.71	11	6	5	5	1.041	0.984	0.896	1.02	0.967	1.049	0.976	1.086	0.95	1.00	1.02
P27824	Calnexin OS=Homo sapiens OX=9606 G	CANX	67.567	169.39	25.8	18	36	18	1.052	1.047	0.97	1.016	0.957	1.105	0.933	0.993	0.95	0.98	0.92
P54709	Sodium/potassium-transporting ATPase s	ATP1B3	31.512	125.99	29.4	9	18	9	1.069	1.048	0.997	1.006	0.999	0.91	1.067	0.964	0.95	0.90	0.96
Q96FX7	tRNA (adenine(58)-N(1))-methyltransfer	TRMT61A	31.381	30.031	13.1	3	5	3	1.025	1.016	0.944	0.987	1.072	1.121	0.897	0.988	0.95	1.07	0.92
Q15019	Septin-2 OS=Homo sapiens OX=9606 G	SEPT2	41.487	236.25	64.8	16	31	16	1.054	1.06	0.991	1.009	0.921	1.039	0.921	0.983	0.95	0.93	0.90
Q9BZ95	Histone-lysine N-methyltransferase NSD	NSD3	161.61	19.287	3.1	3	4	3	0.948	0.977	0.899	0.922	1.121	1.161	1.019	1.116	0.95	1.19	1.11
Q86UE4	Protein LYRIC OS=Homo sapiens OX=9	MTDH	63.836	27.179	5.5	3	5	3	1.02	1.053	0.968	0.993	1.088	1.005	0.968	0.953	0.95	1.01	0.93
Q8NBQ5	Estradiol 17-beta-dehydrogenase 11 OS=	HSD17B11	32.935	56.504	34	8	8	8	1.102	1.025	0.984	1.028	0.933	1.028	0.831	0.914	0.95	0.92	0.82
Q9H410	Kinetochore-associated protein DSN1 ho	DSN1	40.067	30.644	11.8	4	5	4	1.035	0.904	0.823	1.011	1.007	1.041	1.042	1.112	0.95	1.06	1.11

Q8NHP8	Putative phospholipase B-like 2 OS=Hor	PLBD2	65.471	53.915	8.1	4	5	4	1.069	1.163	1.105	1.006	0.932	0.943	0.87	0.89	0.95	0.84	0.79
Q9NRG9	Aladin OS=Homo sapiens OX=9606 GN	AAAS	59.573	33.545	8.6	4	5	4	1.07	1.014	1.002	0.969	1.02	1.093	0.923	0.958	0.95	1.01	0.90
P55769	NHP2-like protein 1 OS=Homo sapiens (	SNU13	14.173	46.201	46.9	6	9	6	0.984	1.112	0.994	0.988	0.976	1.11	0.975	0.962	0.95	1.00	0.92
Q15691	Microtubule-associated protein RP/EB fa	MAPRE1	29.999	148.6	28.7	7	19	7	1.029	1.047	0.972	0.991	0.942	0.999	1.011	1.009	0.95	0.93	0.97
O00754	Lysosomal alpha-mannosidase OS=Homo	MAN2B1	113.74	46.491	8.3	7	9	7	1.032	1.08	0.996	1.001	1.056	1.204	0.831	0.889	0.95	1.07	0.81
Q9Y256	CAAX prenyl protease 2 OS=Homo sapi	RCE1	35.832	24.223	8.2	2	3	2	1.041	0.777	0.716	1.003	1.241	0.777	1.136	1.338	0.95	1.11	1.36
Q9Y4E5	E3 SUMO-protein ligase ZNF451 OS=H	ZNF451	121.48	16.938	2.9	3	3	3	1.028	0.955	0.904	0.971	1.069	0.907	1.09	1.051	0.95	1.00	1.08
Q7RTP6	[F-actin]-monooxygenase MICAL3 OS=I	MICAL3	224.29	25.81	2.5	4	5	3	1.024	1.032	0.97	0.974	1.04	1.006	0.988	1.028	0.95	1.00	0.98
Q9Y262	Eukaryotic translation initiation factor 3	EIF3L	66.726	175.78	36.3	17	33	17	1.044	1.093	1.047	0.973	0.936	0.988	0.977	0.931	0.95	0.90	0.89
Q9Y4L1	Hypoxia up-regulated protein 1 OS=Hom	HYOU1	111.33	228.84	27.4	24	42	23	1.022	1.06	0.978	0.99	0.997	0.991	0.985	1	0.95	0.95	0.95
Q6NVY1	3-hydroxyisobutyryl-CoA hydrolase, mit	HIBCH	43.482	50.341	15.8	5	6	4	1.005	1.112	1.05	0.951	0.948	1.184	0.915	0.908	0.95	1.01	0.86
Q14061	Cytochrome c oxidase copper chaperone	COX17	6.9151	22.074	57.1	3	4	3	1.111	1.24	1.229	0.993	0.836	1.011	0.85	0.703	0.95	0.79	0.66
P42704	Leucine-rich PPR motif-containing prote	LRPPRC	157.9	323.31	40.5	46	66	45	1.034	1.058	0.993	0.984	1.017	1.197	0.836	0.911	0.95	1.06	0.84
Q9UJZ1	Stomatin-like protein 2, mitochondrial O	STOML2	38.534	57.001	25.8	6	14	6	0.988	1.013	0.941	0.95	1.048	1.066	0.947	0.99	0.95	1.06	0.97
Q96L58	Beta-1,3-galactosyltransferase 6 OS=Hor	B3GALT6	37.137	12.894	6.7	2	2	2	0.889	1.039	1.027	0.795	1.118	0.892	1.357	1.184	0.95	1.04	1.32
O15042	U2 snRNP-associated SURP motif-conta	U2SURP	118.29	137.94	16.6	14	22	14	1.027	1.112	1.027	0.994	0.954	1.234	0.762	0.933	0.94	1.02	0.79
Q9HB09	Bcl-2-like protein 12 OS=Homo sapiens	BCL2L12	36.821	11.526	7.5	2	2	2	0.953	1.273	1.027	1.076	0.642	1.026	1.146	0.901	0.94	0.75	0.92
P40939	Trifunctional enzyme subunit alpha, mito	HADHA	82.999	255.4	36.7	21	35	21	1.02	1.025	0.948	0.984	0.989	1.016	1.048	1.026	0.94	0.98	1.01
Q9NUD5	Zinc finger CCHC domain-containing pro	ZCCHC3	43.618	26.909	11.9	4	4	4	0.98	0.927	0.849	0.952	1.062	1.106	0.982	1.265	0.94	1.14	1.18
Q9UHW5	GPN-loop GTPase 3 OS=Homo sapiens (	GPN3	32.761	12.857	10.2	2	2	2	0.954	1.312	1.135	1.005	0.689	1.181	0.863	0.967	0.94	0.83	0.81
Q9NQW6	Anillin OS=Homo sapiens OX=9606 GN	ANLN	124.2	150.92	20.8	20	23	20	0.819	0.759	0.638	0.852	1.358	1.315	1.08	1.41	0.94	1.69	1.58
O43491	Band 4.1-like protein 2 OS=Homo sapier	EPB41L2	112.59	113.74	17.8	15	17	12	1.068	1.082	1.028	1.002	0.944	1.05	0.906	0.934	0.94	0.93	0.86
Q8N806	Putative E3 ubiquitin-protein ligase UBR	UBR7	47.998	59.671	23.5	8	9	8	0.978	0.939	0.897	0.913	1.02	1.072	1.153	1.166	0.94	1.09	1.21
Q9H9P8	L-2-hydroxyglutarate dehydrogenase, mit	L2HGDH	50.315	50.769	17.5	6	6	6	1.006	1.05	0.991	0.95	1.068	1.085	0.969	0.973	0.94	1.05	0.94
Q71RC2	La-related protein 4 OS=Homo sapiens C	LARP4	80.595	78.975	17.7	9	10	9	1.018	1.026	0.984	0.945	1.062	1.036	1	0.982	0.94	1.03	0.97
Q4VC31	Coiled-coil domain-containing protein 58	CCDC58	16.62	20.346	19.4	3	5	3	1.091	1.005	0.98	0.998	0.987	0.943	1.02	0.95	0.94	0.92	0.94
Q8ND76	Cyclin-Y OS=Homo sapiens OX=9606 C	CCNY	39.336	18.941	12.9	3	3	3	1.027	1.048	0.982	0.976	0.97	0.973	1.068	1.013	0.94	0.94	1.00
Q9UKU7	Isobutyryl-CoA dehydrogenase, mitochor	ACAD8	45.069	31.426	14.9	5	4	4	1.105	0.963	0.933	1.018	1.001	0.889	0.982	1	0.94	0.91	0.96
P49902	Cytosolic purine 5'-nucleotidase OS=Hor	NT5C2	64.969	13.576	4.5	2	2	2	1.013	0.999	0.925	0.973	1.049	1.044	0.961	1.113	0.94	1.04	1.03
Q96AE7	Tetratricopeptide repeat protein 17 OS=H	TTC17	129.56	17.772	1.1	1	1	1	0.945	1.114	0.873	1.069	0.945	1.144	0.859	1.116	0.94	1.01	0.96
P33981	Dual specificity protein kinase TTK OS=	TTK	97.071	79.183	11.3	7	8	7	1.091	1.002	1.013	0.961	1.039	1.032	0.959	0.873	0.94	0.99	0.88
O95772	STARD3 N-terminal-like protein OS=Ho	STARD3N	26.654	7.5673	4.3	1	1	1	1.042	0.963	0.887	1.004	1.047	0.982	1.023	1.078	0.94	1.01	1.05
Q86WB0	Nuclear-interacting partner of ALK OS=I	ZC3HC1	55.261	45.622	14.3	6	7	6	0.965	1.037	0.933	0.955	1.072	0.945	1.062	1.035	0.94	1.01	1.05
Q99496	E3 ubiquitin-protein ligase RING2 OS=H	RNF2	37.655	43.895	17	4	5	4	1.041	1.078	0.978	1.02	0.911	1.239	0.787	0.963	0.94	1.01	0.83
P51858	Hepatoma-derived growth factor OS=Hor	HDGF	26.788	150.99	63.8	15	28	14	1.063	1.003	0.97	0.978	0.988	0.935	1.051	0.984	0.94	0.93	0.98
P24752	Acetyl-CoA acetyltransferase, mitochond	ACAT1	45.199	109.58	36.1	12	16	11	0.997	1.121	0.987	1.01	0.999	1.128	0.846	1.003	0.94	1.00	0.87
O15258	Protein RER1 OS=Homo sapiens OX=96	RER1	22.958	17.604	14.8	2	3	2	0.935	1.02	0.827	1.016	1.049	0.925	1.049	1.289	0.94	1.01	1.20
P13984	General transcription factor IIF subunit 2	GTF2F2	28.38	43.885	27.3	7	8	7	1.04	1.03	0.944	1.007	1.001	1.068	1.003	0.915	0.94	1.00	0.93
P19447	General transcription and DNA repair fac	ERCC3	89.277	53.653	10.1	7	7	7	1.055	1.014	0.933	1.017	0.976	1.018	0.971	1.08	0.94	0.96	0.99
O95456	Proteasome assembly chaperone 1 OS=H	PSMG1	32.854	16.047	8.3	2	3	2	1.127	0.881	0.901	0.991	1.033	0.838	1.166	1.023	0.94	0.93	1.09
Q9NWH9	SAFB-like transcription modulator OS=H	SLTM	117.15	64.89	8.1	8	8	8	1.008	1.028	0.954	0.964	1.045	0.981	1.092	1.045	0.94	1.00	1.05
O60220	Mitochondrial import inner membrane tra	TIMM8A	10.998	45.892	38.1	3	5	3	1.064	0.902	0.942	0.91	1.089	1.007	1.065	1.054	0.94	1.07	1.08
Q9BT22	Chitobiosyldiphosphodolichol beta-manr	ALG1	52.518	34.001	12.9	5	5	5	1.006	1.097	0.937	1.044	0.98	1.06	0.919	0.981	0.94	0.97	0.90
Q5W0V3	Protein FAM160B1 OS=Homo sapiens (	FAM160B1	86.557	34.956	6.8	5	6	5	1.002	1.011	0.896	1	0.988	0.984	1.123	1.056	0.94	0.98	1.08
Q5JSH3	WD repeat-containing protein 44 OS=Ho	WDR44	101.37	45.207	9	7	7	7	1.008	1.125	0.992	1.017	0.841	1.039	1.046	0.975	0.94	0.88	0.95
Q9Y394	Dehydrogenase/reductase SDR family me	DHRS7	38.298	41.006	14.5	4	5	4	1.025	1.073	1.009	0.967	0.953	1.015	0.956	0.916	0.94	0.94	0.89
Q9UKM9	RNA-binding protein Raly OS=Homo sa	RALY	32.463	75.236	26.8	10	21	10	1.064	1.026	1.002	0.966	1.07	1.03	1.026	0.982	0.94	1.00	0.96
Q6I9Y2	THO complex subunit 7 homolog OS=Ho	THOC7	23.743	29.583	25.5	4	5	4	1.022	1.099	1.048	0.949	0.955	1.023	1.081	0.674	0.94	0.93	0.83
Q92600	CCR4-NOT transcription complex subun	CNOT9	33.631	49.566	22.1	6	8	6	1.031	0.983	0.949	0.947	1.003	1.159	0.937	1.033	0.94	1.07	0.98
Q96NT0	Coiled-coil domain-containing protein 11	CCDC115	19.76	12.478	13.9	2	3	2	0.986	1.127	1.011	0.978	0.986	1.126	0.857	1.024	0.94	1.00	0.89
Q8WVY7	Ubiquitin-like domain-containing CTD p	UBLCP1	36.804	17.818	9.1	3	4	3	1.121	1.023	1.018	1	0.948	1	0.938	0.918	0.94	0.91	0.87
Q9Y3B4	Splicing factor 3B subunit 6 OS=Homo s	SF3B6	14.585	36.823	34.4	4	10	4	1.041	1.068	1.007	0.978	1.003	1.085	0.89	0.938	0.94	0.99	0.87
P13639	Elongation factor 2 OS=Homo sapiens O	EEF2	95.337	323.31	36.2	31	77	30	1.041	1.151	1.047	1.016	0.915	1.14	0.802	0.877	0.94	0.94	0.77
P42025	Beta-centractin OS=Homo sapiens OX=9	ACTR1B	42.293	27.035	15.7	6	8	2	1.081	0.973	0.944	0.989	1.022	0.945	1.025	1.024	0.94	0.96	1.00
A6NDB9	Paralemmin-3 OS=Homo sapiens OX=96	PALM3	71.695	20.1	5.1	3	3	3	1.069	0.488	0.639	0.826	1.492	1.262	0.949	1.416	0.94	1.77	1.52
Q7Z4F1	Low-density lipoprotein receptor-related	LRP10	76.193	16.604	6.5	3	3	3	1.043	0.933	0.947	0.912	1.126	1.063	0.972	1.1	0.94	1.11	1.05
Q9GZP9	Derlin-2 OS=Homo sapiens OX=9606 G	DERL2	27.567	22.93	17.2	3	3	3	0.971	0.985	0.853	0.987	1.033	1.173	0.947	1.153	0.94	1.13	1.07
Q92542	Nicastrin OS=Homo sapiens OX=9606 C	NCSTN	78.41	37.795	5.6	4	5	4	1.034	0.965	0.898	0.982	1.051	0.86	1.186	0.984	0.94	0.96	1.09
Q9GZY8	Mitochondrial fission factor OS=Homo s	MFF	38.464	46.933	31.3	7	8	7	1.009	1.018	0.97	0.936	1.036	1.222	0.883	1.085	0.94	1.11	0.97
P45985	Dual specificity mitogen-activated protein	MAP2K4	44.287	15.514	6.5	2	2	2	0.989	1.052	0.956	0.963	1.077	1.248	0.77	1.048	0.94	1.14	0.89
Q9H0S4	Probable ATP-dependent RNA helicase I	DDX47	50.646	33.567	11.9	5	6	5	1.073	0.946	0.976	0.922	1.064	1.001	1.109	1.058	0.94	1.02	1.07
Q96FJ2	Dynein light chain 2, cytoplasmic OS=Ho	DYNLL2	10.35	59.556	37.1	2	11	1	1.141	0.928	0.977	0.968	1.026	0.819	1.118	1.011	0.94	0.89	1.03
O60832	H/ACA ribonucleoprotein complex subur	DKC1	57.673	66.708	17.5	7	10	7	0.984	1.117	1.004	0.971	0.858	1.233	0.887	1.008	0.94	1.00	0.90
Q02809	Procollagen-lysine,2-oxoglutarate 5-diox	PLOD1	83.549	87.913	13.6	7	9	7	1.047	0.97	0.91	0.986	1.015	0.926	1.12	1.096	0.94	0.96	1.10
Q6SPF0	Atherin OS=Homo sapiens OX=9606 GN	SAMD1	56.051	15.686	2.2	1	1	1	1.034	1.132	1.1	0.936	1.135	0.772	1.114	0.846	0.94	0.88	0.90
Q12906	Interleukin enhancer-binding factor 3 OS	ILF3	95.337	223.96	31.5	23	43	21	1.016	1.048	0.971	0.969	1.002	1.058	0.93	1.02	0.94	1.00	0.94
Q9H4G0	Band 4.1-like protein 1 OS=Homo sapier	EPB41L1	98.502	21.177	5	3	3	2	0.918	0.946	0.751	1.001	0.992	0.898	1.365	1.358	0.94	1.01	1.46
Q7L592	Protein arginine methyltransferase NDUF	NDUFAF7	49.237	49.731	15.9	5	5	5	1.019	1.028	0.92	1.004	0.957	1.031	1.005	1.084	0.94	0.97	1.02
O75131	Copine-3 OS=Homo sapiens OX=9606 C	CPNE3	60.13	47.396	11.4	6	8	5	1.087	0.922	0.903	0.985	1.04	0.946	1.055	1.026	0.94	0.99	1.04
Q96S99	Pleckstrin homology domain-containing f	PLEKHF1	31.194	24.671	12.2	3	6	3	1.018	0.988	0.923	0.962	1.028	1.105	1.066	1.052	0.94	1.06	1.06
Q9BS26	Endoplasmic reticulum resident protein 4	ERP44	46.971	87.031	32.8	10	16	10	1.005	1.083	0.974	0.988	0.948	1.193	0.872	0.974	0.94	1.03	0.88
Q9UPU5	Ubiquitin carboxyl-terminal hydrolase 24	USP24	294.36	183.85	9	20	21	20	1.018	1.051	0.915	1.029	0.942	1.023	0.979	1.039	0.94	0.95	0.98
Q96CW1	AP-2 complex subunit mu OS=Homo sap	AP2M1	49.654	85.082	31.7	10	15	10	1.053	0.981	0.961	0.95	1.033	1.101	1.041	1.041	0.94	1.05	1.02
O94966	Ubiquitin carboxyl-terminal hydrolase 19	USP19	145.65	38.416	4.7	6	7	6	1.044	1.022	1.026	0.915	1.054	1.257	0.796	0.981	0.94	1.12	0.86
Q9UQE7	Structural maintenance of chromosomes p	SMC3	141.54	323.31	31.1	32	45	31	1.008	1.058	0.95	0.991	0.977	1.036	0.971	1.02	0.94	0.97	0.96
Q9HD47	Ran guanine nucleotide release factor OS	RANGRF	20.448	14.51	12.4	2	2	2	0.969	1.14	0.964	1.017	0.953	0.975	0.984	1.077	0.94	0.91	0.98
Q9HCN8	Stromal cell-derived factor 2-like protein	SDF2L1	23.598	78.3	36.7	6	7	6	0.976	1.08	0.967	0.964	1.032	0.906	1.058	1.04	0.94	0.94	1.02
Q9Y4W6	AFG3-like protein 2 OS=Homo sapiens (	AFG3L2	88.583	80.95	16.1	12	13	11	1.024	0.982	0.91	0.974	1.077	1.044	1.014	1.013	0.94	1.06	1.01
P55084	Trifunctional enzyme subunit beta, mitoc	HADHB	51.294	99.336	34	14	19	14	1.038	1.029	0.987	0.954	0.997	1.007	0.987	0.997	0.94	0.97	0.96
Q15645	Pachytene checkpoint protein 2 homolog	TRIP13	48.55	69.348	25.9	9	12	9	1.04	1.107	1.014	1.002	0.962	1.189	0.917	0.935	0.94	1.00	0.86
P35244	Replication protein A 14 kDa subunit OS	RPA3	13.569	57.112	45.5	4	6	4	1.001	0.98	0.915	0.945	1.115	1.126	1.058	1.027	0.94	1.13	1.05
P35579	Myosin-9 OS=Homo sapiens OX=9606 (	MYH9	226.53	323.31	44.7	91	207	71	1.061	1.097	1.019	1.007	0.869	0.869	1.08	0.983	0.94	0.81	0.96
Q92674	Centromere protein I OS=Homo sapiens (	CENPI	86.719	7.3187	1.5	1	1	1	1.046	1.013	0.952	0.981	1.015	1.087	0.972	0.97	0.94	1.02	0.94
Q15366	Poly(rC)-binding protein 2 OS=Homo sa	PCBP2	38.58	75.523	35.3	9	31	4	1.06	1.046	1.028	0.949	1	0.975	1.058	0.943	0.94	0.94	0.95
Q8TAE8	Growth arrest and DNA damage-inducibl	GADD45G	25.384	14.33	9	2	4	2	1.055	1.001	0.948	0.982	1.024	0.966	1.054	1	0.94	0.97	1.00
P54105	Methylosome subunit pICln OS=Homo s	CLNS1A	26.215	32.629	24.1	4	6	4	1.118	1.033	1.037	0.982	0.962	1.058	0.858	0.936	0.94	0.94	0.83
P50281	Matrix metalloproteinase-14 OS=Homo s	MMP14	65.893	111.51	24.2	11	16	11	0.972	0.983	0.881	0.954	1.059	1.051	0.998	1.182	0.94	1.08	1.12
P61289	Proteasome activator complex subunit 3 (	PSME3	29.506	170.9	39.4	10	18	9	1.038	1.095	1.023	0.979	0.953	1.049	0.944	0.941	0.94	0.94	0.88
Q9BSC4	Nucleolar protein 10 OS=Homo sapiens (	NOL10	80.301	46.664	9.2	5	5	5	1.034	0.997	0.916	0.99	1.143	1.002	0.984	0.972	0.94	1.06	0.96
Q9H2C0	Gigaxonin OS=Homo sapiens OX=9606	GAN	67.638	60.59	17.3	8	8	7	0.921	0.914	0.721	1.001	1.056	1.249	0.968	1.426	0.94	1.26	1.30
O00401	Neural Wiskott-Aldrich syndrome protein	WASL	54.826	58.514	21.4	9	11	9	1.096	1.015	0.97	1.011	1.053	1.114	0.975	0.98	0.94	1.03	0.93
Q7Z4V5	Hepatoma-derived growth factor-related p	HDGFL2	74.316	20.416	5.4	4	6	3	1.066	1.009	0.941	1.006	1.089	0.857	1.099	1.065	0.94	0.94	1.04
Q08AM6	Protein VAC14 homolog OS=Homo sapi	VAC14	87.972	114	14.5	9	11	9	1.045	1.028	1.015	0.93	1.074	0.896	1.063	1.025	0.94	0.95	1.01
Q8TCT9	Minor histocompatibility antigen H13 OS	HM13	41.488	68.394	12.2	5	8	5	1.044	1.045	0.999	0.961	0.996	1.12	0.875	0.96	0.94	1.01	0.88
O95298	NADH dehydrogenase [ubiquinone] 1 sul	NDUFC2	14.187	11.842	16.8	2	2	2	1.034	0.99	0.886	1.013	1.049	1.006	0.973	1.074	0.94	1.02	1.01
O43837	Isocitrate dehydrogenase [NAD] subunit	IDH3B	42.183	75.877	18.7	6	8	6	0.999	1.04	0.974	0.939	0.896	1.007	1.097	1.142	0.94	0.93	1.10
Q05513	Protein kinase C zeta type OS=Homo sap	PRKCZ	67.659	50.898	9.5	5	5	4	0.999	1.117	1.03	0.955	1.076	1.378	0.746	0.906	0.94	1.16	0.78
Q13085	Acetyl-CoA carboxylase 1 OS=Homo sap	ACACA	265.55	221.4	14	26	32	26	1.045	1.02	0.956	0.981	1.021	1.057	0.972	1.023	0.94	1.01	0.97
Q9NW81	Distal membrane-arm assembly complex	DMAC2	29.267	27.951	11.7	2	2	2	0.99	1.183	0.724	1.314	1.029	0.745	0.917	0.925	0.94	0.82	0.85
Q5T653	39S ribosomal protein L2, mitochondrial	MRPL2	33.3	41.06	16.7	3	4	3	0.986	1.071	0.982	0.947	1.071	1.224	0.758	1.028	0.94	1.12	0.87
Q7L2H7	Eukaryotic translation initiation factor 3	EIF3M	42.502	178.98	32.9	10	22	10	1.041	1.095	1.015	0.988	0.959	1.016	0.895	0.889	0.94	0.92	0.84
Q4U2R6	39S ribosomal protein L51, mitochondria	MRPL51	15.095	9.243	8.6	1	1	1	0.896	1.126	1.035	0.861	1.025	1.257	0.975	1.082	0.94	1.13	1.02
P53803	DNA-directed RNA polymerases I, II, and	POLR2K	7.0041	8.8234	31	1	1	1	1.093	1.023	1.017	0.967	0.96	0.949	1.001	1.005	0.94	0.90	0.95
P28838	Cytosol aminopeptidase OS=Homo sapie	LAP3	56.166	44.706	15.6	5	5	5	0.998	1.062	0.931	1	0.975	0.966	1.087	1.044	0.94	0.94	1.03
P82930	28S ribosomal protein S34, mitochondria	MRPS34	25.65	23.531	17.9	3	6	3	0.981	1.221	1.066	0.998	0.936	1.096	0.849	0.935	0.94	0.92	0.81
Q9NQX3	Gephyrin OS=Homo sapiens OX=9606 C	GPHN	79.748	60.891	12	8	11	8	1.06	0.998	0.986	0.943	1.062	1.153	0.947	0.977	0.94	1.08	0.93
Q86SQ0	Pleckstrin homology-like domain family	PHLDB2	142.16	26.892	3.8	4	4	4	0.973	1.05	1.024	0.872	1.134	1.237	0.536	1.38	0.94	1.17	0.95
Q9HAV7	GrpE protein homolog 1, mitochondrial (	GRPEL1	24.279	17.906	12.9	2	3	2	0.973	1.049	0.944	0.951	0.987	0.921	1.059	1.055	0.94	0.94	1.05
Q9H8V3	Protein ECT2 OS=Homo sapiens OX=96	ECT2	103.5	45.519	9.2	7	7	6	0.999	0.957	0.922	0.911	1.149	1.162	0.928	1.08	0.94	1.18	1.03
O75394	39S ribosomal protein L33, mitochondria	MRPL33	7.6191	15.767	38.5	2	2	2	1.08	1.023	1.062	0.908	0.995	0.974	1.073	0.948	0.94	0.94	0.96
O60502	Protein O-GlcNAcase OS=Homo sapiens	MGEA5	102.91	140.72	19.4	14	18	14	1.085	1.111	1.026	1.031	0.898	0.932	1.075	0.972	0.94	0.83	0.93
Q96DM3	Regulator of MON1-CCZ1 complex OS=	RMC1	74.974	28.766	6.2	4	6	4	1.031	1.067	0.978	0.987	0.918	0.991	0.931	1.039	0.94	0.91	0.94
Q9UPT5	Exocyst complex component 7 OS=Hom	EXOC7	83.381	95.228	18.8	12	13	12	1.019	1.072	0.972	0.986	0.988	0.92	1.074	0.939	0.94	0.91	0.96
Q96AT1	Uncharacterized protein KIAA1143 OS=I	KIAA1143	17.465	18.84	14.9	2	2	2	0.984	1.133	1.009	0.973	0.954	1.163	0.893	0.99	0.94	1.00	0.89
Q9BQ52	Zinc phosphodiesterase ELAC protein 2 (	ELAC2	92.218	133.94	22.5	14	19	14	1.099	1.015	1.016	0.963	1.003	0.955	1.084	0.973	0.94	0.93	0.97
Q9Y2P4	Long-chain fatty acid transport protein 6	SLC27A6	70.111	82.778	19.1	11	16	11	1.028	0.951	0.927	0.925	1.051	0.959	1.131	1.137	0.94	1.02	1.15
Q5TZA2	Rootletin OS=Homo sapiens OX=9606 (	CROCC	228.52	11.932	1	2	3	2	1.063	1.025	1.03	0.924	1.004	0.985	1.123	0.91	0.94	0.95	0.97
Q9NRK6	ATP-binding cassette sub-family B memb	ABCB10	79.147	79.287	14.5	10	13	9	0.972	1.016	0.898	0.962	1.055	1.063	1.081	1.074	0.94	1.07	1.08
P62805	Histone H4 OS=Homo sapiens OX=9606	HIST1H4A	11.367	66.517	51.5	7	41	6	1.051	0.921	0.966	0.879	1.048	1.168	0.93	1.067	0.94	1.12	1.01
Q96CN9	GRIP and coiled-coil domain-containing	GCC1	87.81	46.284	9.2	6	6	6	1.051	1.059	0.994	0.98	1.023	1.14	0.834	0.956	0.94	1.03	0.85
Q8NEJ9	Neuroguidin OS=Homo sapiens OX=960	NGDN	35.894	26.304	15.2	4	5	4	1.017	0.999	0.925	0.961	0.997	0.995	1.083	1.115	0.94	0.99	1.09
Q02127	Dihydroorotate dehydrogenase (quinone)	DHODH	42.867	42.53	17.2	6	6	6	0.989	1.027	0.871	1.015	0.941	1.114	0.889	1.227	0.94	1.02	1.05
Q9H2G2	STE20-like serine/threonine-protein kina	SLK	142.69	64.268	8	10	10	10	1.039	0.992	0.871	1.029	0.927	0.955	1.032	1.088	0.94	0.93	1.04
Q9Y6M1	Insulin-like growth factor 2 mRNA-bindi	IGF2BP2	66.121	75.931	22	11	17	9	1.012	1.127	0.986	1.015	0.88	0.971	1.07	0.982	0.94	0.87	0.96
Q9UL25	Ras-related protein Rab-21 OS=Homo sa	RAB21	24.347	47.001	33.3	6	7	6	1.034	1.027	0.936	0.992	0.907	0.953	1.068	1.014	0.94	0.90	1.01
P84103	Serine/arginine-rich splicing factor 3 OS=	SRSF3	19.329	79.358	43.3	8	15	7	1.032	1.089	1.017	0.967	0.95	1.208	0.885	0.956	0.94	1.02	0.87
O60341	Lysine-specific histone demethylase 1A (	KDM1A	92.902	161.11	29.1	16	25	16	1.086	1.065	1.033	0.979	0.972	0.944	0.902	0.913	0.94	0.89	0.84
P34931	Heat shock 70 kDa protein 1-like OS=Ho	HSPA1L	70.374	-2	17.2	8	25	1	1.046	1.124	1.034	0.995	0.623	1.174	0.904	1.15	0.94	0.83	0.95
Q9NRG0	Chromatin accessibility complex protein	CHRAC1	14.71	19.168	13.7	1	2	1	1.009	1.052	0.909	1.018	0.922	1.288	0.775	1.079	0.93	1.07	0.90
Q9BUF5	Tubulin beta-6 chain OS=Homo sapiens (	TUBB6	49.857	110.83	42.4	17	33	6	1.027	1.046	0.953	0.985	0.915	0.938	1.084	1.078	0.93	0.89	1.04
P11142	Heat shock cognate 71 kDa protein OS=I	HSPA8	70.897	323.31	57.6	34	151	29	1.019	0.975	0.886	0.978	1.074	0.943	1.064	1.083	0.93	1.01	1.08
Q8IYU8	Calcium uptake protein 2, mitochondrial	MICU2	49.666	31.088	8.8	3	3	3	0.981	1.089	0.944	0.991	1.013	1.146	0.986	0.93	0.93	1.04	0.93
Q86VP1	Tax1-binding protein 1 OS=Homo sapier	TAX1BP1	90.876	52.916	9.4	8	9	8	0.937	0.912	0.781	0.947	1.27	1.12	0.967	1.237	0.93	1.29	1.19
P21912	Succinate dehydrogenase [ubiquinone] ir	SDHB	31.629	92.762	29.6	7	12	7	1.065	0.995	0.966	0.959	1.029	1.147	0.836	1.009	0.93	1.06	0.90
P20810	Calpastatin OS=Homo sapiens OX=9606	CAST	76.572	158.93	27.7	15	22	15	1.038	1.083	0.984	0.998	0.914	0.888	1.096	1.051	0.93	0.85	1.01
Q9HAU5	Regulator of nonsense transcripts 2 OS=I	UPF2	147.81	91.724	12.5	12	18	12	1.009	0.998	0.83	1.045	1.007	0.924	1.101	1.134	0.93	0.96	1.11
Q13111	Chromatin assembly factor 1 subunit A C	CHAF1A	106.91	44.812	8.2	5	5	5	0.992	0.89	0.804	0.954	1.114	1.226	0.876	1.256	0.93	1.24	1.13
Q8WXX5	DnaJ homolog subfamily C member 9 OS	DNAJC9	29.909	80.974	27.3	9	12	9	1.004	0.923	0.872	0.928	1.077	0.992	1.184	1.14	0.93	1.07	1.21
O15320	Endoplasmic reticulum export factor CTA	CTAGE5	90.995	34.729	7.3	5	5	5	1.051	0.967	0.917	0.968	1.192	1.155	0.838	0.953	0.93	1.16	0.89
Q01130	Serine/arginine-rich splicing factor 2 OS=	SRSF2	25.476	11.623	7.2	2	3	2	0.994	1.039	1.033	0.866	1.037	1.008	1.168	1.021	0.93	1.01	1.08
Q6NXT1	Ankyrin repeat domain-containing protein	ANKRD54	32.505	9.8647	5.3	1	1	1	1.052	0.995	0.917	0.995	0.983	0.92	1.108	1.052	0.93	0.93	1.06
Q9NUP9	Protein lin-7 homolog C OS=Homo sapie	LIN7C	21.834	31.593	25.9	4	5	4	1.063	1.035	0.955	1.004	0.99	1.002	0.929	1.023	0.93	0.95	0.93
Q00537	Cyclin-dependent kinase 17 OS=Homo s	CDK17	59.582	20.147	8	4	5	3	1.014	1.068	0.934	1.01	0.923	0.923	1.162	1.007	0.93	0.89	1.04
P61421	V-type proton ATPase subunit d 1 OS=H	ATP6V0D1	40.329	91.239	26.2	8	14	8	1.058	0.978	0.953	0.948	1.031	0.948	0.998	1.013	0.93	0.97	0.99
Q8NEM2	SHC SH2 domain-binding protein 1 OS=	SHCBP1	75.69	67.076	21.6	10	10	9	0.906	0.841	0.73	0.901	1.315	1.207	1.011	1.221	0.93	1.44	1.28
P36776	Lon protease homolog, mitochondrial OS	LONP1	106.49	176.45	23.6	20	29	20	1.032	1.031	0.971	0.955	1.004	0.975	0.965	0.984	0.93	0.96	0.94
P07910	Heterogeneous nuclear ribonucleoprotein	HNRNPC	33.67	123.51	33.7	12	35	12	1.064	1.038	0.964	0.998	0.977	0.985	0.968	0.992	0.93	0.93	0.93
Q7L1T6	Cytochrome b5 reductase 4 OS=Homo sa	CYB5R4	59.473	6.7711	2.5	1	1	1	1.012	1.054	0.918	1.01	1.004	0.984	0.97	1.097	0.93	0.96	1.00
Q9Y2D5	A-kinase anchor protein 2 OS=Homo sap	AKAP2	94.659	68.848	11.3	9	10	9	0.919	1.16	0.946	0.994	0.836	0.725	1.337	1.122	0.93	0.75	1.18
Q9UHX1	Poly(U)-binding-splicing factor PUF60 C	PUF60	59.875	119.34	29	13	20	12	1.02	1.028	0.966	0.945	0.98	1.031	0.979	1.039	0.93	0.98	0.99
O75964	ATP synthase subunit g, mitochondrial O	ATP5L	11.428	31.328	37.9	4	6	4	1.068	0.989	0.928	0.991	1.044	1.271	0.74	1.049	0.93	1.13	0.87
Q14657	EKC/KEOPS complex subunit LAGE3 C	LAGE3	14.804	15.513	16.1	1	2	1	1.016	0.877	0.756	1.01	0.904	0.965	1.105	1.426	0.93	0.99	1.34
Q8N442	Translation factor GUF1, mitochondrial (	GUF1	74.327	27.449	7.3	4	4	4	1.026	1.026	0.915	0.999	1.059	1.102	0.935	0.979	0.93	1.05	0.93
P46060	Ran GTPase-activating protein 1 OS=Hor	RANGAP1	63.541	208.53	32.9	17	31	17	1.055	1.095	1.018	0.987	0.93	0.974	0.945	0.896	0.93	0.89	0.86
P07311	Acylphosphatase-1 OS=Homo sapiens O	ACYP1	11.261	6.4035	10.1	1	1	1	0.991	1.024	0.96	0.919	1.056	1.212	0.845	1.131	0.93	1.13	0.98
P56545	C-terminal-binding protein 2 OS=Homo s	CTBP2	48.944	27.935	10.3	4	6	2	1.051	0.942	0.802	1.056	0.957	0.917	1.137	1.117	0.93	0.94	1.13

Q13595	Transformer-2 protein homolog alpha OS	TRA2A	32.688	51.171	14.5	3	5	3	1.081	1.287	1.03	1.177	0.511	1.17	0.834	0.785	0.93	0.71	0.68
Q96T83	Sodium/hydrogen exchanger 7 OS=Homo	SLC9A7	80.13	6.145	1.2	1	1	1	1.015	0.985	0.961	0.903	0.955	1.009	1.222	1.073	0.93	0.98	1.15
Q9BXS4	Transmembrane protein 59 OS=Homo sa	TMEM59	36.223	6.6433	4	1	2	1	1.011	0.929	0.905	0.903	1.137	1.375	0.753	1.124	0.93	1.29	0.97
Q8NEZ5	F-box only protein 22 OS=Homo sapiens	FBXO22	44.508	47.407	20.3	7	7	5	1.038	1.034	0.941	0.99	1.009	1.108	0.92	1.002	0.93	1.02	0.93
Q12756	Kinesin-like protein KIF1A OS=Homo sa	KIF1A	191.06	12.295	2.5	4	4	2	0.848	1.705	1.26	1.119	0.681	0.892	0.943	0.643	0.93	0.62	0.62
P01111	GTPase NRas OS=Homo sapiens OX=96	NRAS	21.229	16.62	23.3	4	6	2	1.028	1.053	0.953	0.986	1.035	1.004	1.035	0.953	0.93	0.98	0.96
P34896	Serine hydroxymethyltransferase, cytosol	SHMT1	53.082	76.681	15.9	6	10	5	0.992	1.001	0.904	0.953	1.016	1.198	0.881	1.059	0.93	1.11	0.97
Q6UW63	KDEL motif-containing protein 1 OS=Hc	KDELC1	58.042	20.269	4.4	2	3	2	1.065	1.089	1.021	0.986	0.971	0.878	1.052	0.956	0.93	0.86	0.93
P17655	Calpain-2 catalytic subunit OS=Homo sa	CAPN2	79.994	85.208	20.6	11	11	10	1.058	1.008	0.923	1.002	0.908	0.875	1.163	1.09	0.93	0.86	1.09
Q9H1A4	Anaphase-promoting complex subunit 1 (	ANAPC1	216.5	28.888	2.5	4	5	4	0.993	1.043	0.949	0.948	1.051	1.117	0.93	1.08	0.93	1.06	0.99
P16219	Short-chain specific acyl-CoA dehydroge	ACADS	44.297	12.251	6.1	2	2	2	1.04	1.115	0.995	1.012	0.86	1.054	1.016	0.93	0.93	0.89	0.90
Q5TGY3	AT-hook DNA-binding motif-containing	AHDC1	168.35	20.452	2.7	3	3	3	0.985	0.878	0.703	1.032	1.013	1.022	1.063	1.367	0.93	1.09	1.30
P39687	Acidic leucine-rich nuclear phosphoprote	ANP32A	28.585	86.445	21.3	6	19	4	1.058	1.096	1.011	0.995	0.937	0.956	1.091	0.903	0.93	0.88	0.93
P84090	Enhancer of rudimentary homolog OS=H	ERH	12.259	65.085	45.2	4	9	4	1.074	1.045	0.988	0.985	0.942	1.019	0.945	1.037	0.93	0.93	0.94
P30566	Adenylosuccinate lyase OS=Homo sapier	ADSL	54.889	78.655	17.1	6	10	6	1.044	1.132	1.097	0.929	1.008	1.161	0.845	0.916	0.93	1.00	0.81
P55265	Double-stranded RNA-specific adenosine	ADAR	136.06	98.918	13.9	14	18	13	1.08	1.089	1.036	0.983	0.957	1.069	0.884	0.914	0.93	0.93	0.83
Q9H2H8	Peptidyl-prolyl cis-trans isomerase-like 3	PPIL3	18.154	15.335	13.7	2	2	2	1.059	0.936	0.932	0.925	0.978	1.27	0.92	1.057	0.93	1.13	0.99
O75935	Dynactin subunit 3 OS=Homo sapiens O	DCTN3	21.119	26.717	19.4	4	5	4	1.067	1.043	1.035	0.929	0.952	1.079	0.949	0.902	0.93	0.96	0.88
Q9BV38	WD repeat-containing protein 18 OS=Ho	WDR18	47.405	41.321	15.5	6	8	6	1.091	0.887	0.949	0.892	1.131	0.816	0.831	1.132	0.93	0.98	0.99
Q15637	Splicing factor 1 OS=Homo sapiens OX=	SF1	68.329	68.266	14.9	8	11	8	1.04	1.039	0.963	0.972	0.982	1.129	0.859	0.955	0.93	1.02	0.87
Q9Y4D7	Plexin-D1 OS=Homo sapiens OX=9606	PLXND1	212	49.677	3.5	6	6	6	1.05	1.057	0.963	0.998	0.964	1.164	0.835	1.058	0.93	1.01	0.90
Q8IWX8	Calcium homeostasis endoplasmic reticul	CHERP	103.7	134.66	18.9	15	22	14	1.056	1.035	0.984	0.962	1.004	1.04	0.976	0.979	0.93	0.98	0.93
Q9Y426	C2 domain-containing protein 2 OS=Hor	C2CD2	75.532	6.9394	2.4	1	1	1	1.098	0.863	0.909	0.916	1.29	0.944	1.009	1.011	0.93	1.14	1.03
O75326	Semaphorin-7A OS=Homo sapiens OX=!	SEMA7A	74.823	9.7129	2	1	1	1	0.985	1.086	0.918	1.009	0.944	1.076	0.962	1.093	0.93	0.98	0.99
Q8ND82	Zinc finger protein 280C OS=Homo sapi	ZNF280C	83.095	12.102	2.3	2	2	2	1.082	1.044	1.069	0.909	0.997	1.176	0.725	1.074	0.93	1.02	0.85
Q9P0R6	GSK3B-interacting protein OS=Homo sa	GSKIP	15.648	7.7635	7.9	1	1	1	1.183	0.87	0.949	0.961	0.98	1.156	0.885	0.963	0.93	1.04	0.90
P09497	Clathrin light chain B OS=Homo sapiens	CLTB	25.19	28.835	17.5	4	6	4	1.066	1.055	0.945	1.028	0.959	0.926	1.014	1.017	0.93	0.89	0.96
Q9NR09	Baculoviral IAP repeat-containing protei	BIRC6	530.25	130.03	4.7	19	22	19	1.025	1.095	0.966	1.006	0.958	0.979	0.957	1.017	0.93	0.91	0.93
O00161	Synaptosomal-associated protein 23 OS=	SNAP23	23.354	114.63	59.7	8	10	7	1.047	1.029	0.933	0.998	0.939	0.953	1.032	1.056	0.93	0.91	1.01
Q13610	Periodic tryptophan protein 1 homolog O	PWP1	55.827	82.178	18.4	8	10	8	1.001	1.043	0.98	0.921	0.996	1.223	0.892	0.988	0.93	1.09	0.92
Q86W56	Poly(ADP-ribose) glycohydrolase OS=H	PARG	111.11	52.048	6.9	6	7	6	1.093	1.049	1.005	0.987	0.971	0.918	0.965	1.012	0.93	0.88	0.92
P50749	Ras association domain-containing protei	RASSF2	37.79	16.379	12.6	3	2	2	1.069	1.201	1.079	1.032	0.81	1.057	0.84	0.899	0.93	0.82	0.77
Q92544	Transmembrane 9 superfamily member 4	TM9SF4	74.518	24.164	8.4	4	4	4	1.006	1.021	1.009	0.876	1.072	1.065	1.065	1.036	0.93	1.05	1.04
Q9BYD3	39S ribosomal protein L4, mitochondrial	MRPL4	34.919	31.407	14.5	3	4	3	1.079	1.039	1.035	0.934	1.097	1.108	0.873	0.992	0.93	1.04	0.88
P27986	Phosphatidylinositol 3-kinase regulatory	PIK3R1	83.597	30.598	8	5	5	5	1.006	1.094	1.023	0.929	1.025	0.908	0.968	0.925	0.93	0.92	0.90
P14927	Cytochrome b-c1 complex subunit 7 OS=	UQCRB	13.53	29.582	29.7	3	4	3	1.09	0.98	0.939	0.985	1.036	0.958	1.027	0.965	0.93	0.96	0.96
O95363	Phenylalanine--tRNA ligase, mitochondri	FARS2	52.356	23.373	7.5	3	3	3	0.999	1.081	1	0.933	0.961	1.255	0.889	1.002	0.93	1.07	0.91
Q00341	Vigilin OS=Homo sapiens OX=9606 GN	HDLBP	141.45	282.1	22.6	25	40	25	1.052	1.026	0.967	0.964	1.007	0.996	0.97	1.002	0.93	0.96	0.95
Q92508	Piezo-type mechanosensitive ion channel	PIEZO1	286.79	22.365	1.3	3	4	3	1.071	1.021	0.972	0.972	1.071	1.252	0.748	0.921	0.93	1.11	0.80
P48729	Casein kinase I isoform alpha OS=Homo	CSNK1A1	38.914	60.16	23.7	7	11	7	1.047	1.016	0.968	0.949	0.977	1.136	0.847	1.019	0.93	1.02	0.90
Q8TCD5	5'(3')-deoxyribonucleotidase, cytosolic ty	NT5C	23.382	29.072	25.4	4	5	4	1.059	1.125	1.113	0.916	0.973	1.319	0.783	0.788	0.93	1.05	0.72
Q9Y3B7	39S ribosomal protein L11, mitochondria	MRPL11	20.683	66.738	39.1	7	12	7	1.047	1.08	1.04	0.936	1.004	1.213	0.927	0.934	0.93	1.04	0.87
P01116	GTPase KRas OS=Homo sapiens OX=96	KRAS	21.656	54.266	30.2	5	7	3	1.083	1.084	1.006	1.007	0.918	0.868	1.051	0.946	0.93	0.82	0.92
Q9NWK9	Box C/D snoRNA protein 1 OS=Homo s	ZNHIT6	53.918	36.679	18.3	6	6	6	0.973	0.966	0.825	0.976	1.088	0.989	1.016	1.277	0.93	1.07	1.18
P00505	Aspartate aminotransferase, mitochondria	GOT2	47.517	167.96	42.6	15	31	15	1.041	1.006	0.921	0.98	1.043	1.204	0.855	1.013	0.93	1.10	0.91
Q96HS1	Serine/threonine-protein phosphatase PG	PGAM5	32.004	43.389	20.4	5	5	5	0.998	1.062	0.93	0.983	1.023	1.294	0.776	1.015	0.93	1.12	0.87
Q96J01	THO complex subunit 3 OS=Homo sapie	THOC3	38.771	63.335	27.4	8	12	8	1.054	1.116	1.035	0.98	0.935	1.035	0.892	0.892	0.93	0.91	0.82
Q14671	Pumilio homolog 1 OS=Homo sapiens O	PUM1	126.47	104.67	11	10	13	6	1.026	1.156	1.008	1.018	0.904	1.037	0.912	0.943	0.93	0.89	0.85
P04062	Glucosylceramidase OS=Homo sapiens C	GBA	59.716	62.232	11.9	6	9	6	0.992	1.102	0.967	0.977	1.02	0.959	1.124	1.037	0.93	0.95	1.03
Q15528	Mediator of RNA polymerase II transcrip	MED22	22.22	18.292	14.5	2	2	2	1.02	1.003	0.845	1.033	1.103	0.888	1.044	1.085	0.93	0.98	1.05
Q8N5M1	ATP synthase mitochondrial F1 complex	ATPAF2	32.772	21.459	7.6	2	3	2	0.993	1.029	0.926	0.951	0.984	1.222	0.909	1.018	0.93	1.09	0.95
Q9BPX6	Calcium uptake protein 1, mitochondrial	MICU1	54.351	19.249	6.7	3	3	3	1.109	1.024	1.019	0.961	0.966	1.16	0.851	0.916	0.93	1.00	0.83
Q16740	ATP-dependent Clp protease proteolytic	CLPP	30.18	57.487	35.4	7	10	7	1.015	1.03	0.936	0.962	1.027	1.021	1.044	1.01	0.93	1.00	1.00
Q10471	Polypeptide N-acetylgalactosaminyltrans	GALNT2	64.732	65.667	20.3	10	12	9	1.102	1.026	1.016	0.959	1.013	0.914	1.128	0.914	0.93	0.91	0.96
O95260	Arginyl-tRNA--protein transferase 1 OS=	ATE1	59.09	52.918	11.2	4	4	4	1.044	1.056	0.939	1.01	0.934	0.948	1.108	1.077	0.93	0.90	1.04
Q8NBM4	Ubiquitin-associated domain-containing	UBAC2	38.963	24.436	11	4	4	4	1.082	1.072	1.008	0.991	0.972	1.053	0.89	0.955	0.93	0.94	0.86
O95816	BAG family molecular chaperone regulato	BAG2	23.772	45.27	32.7	6	7	6	1.015	1	0.953	0.917	1.005	0.946	1.073	0.969	0.93	0.97	1.01
O75718	Cartilage-associated protein OS=Homo s	CRTAP	46.561	76.524	19.7	7	8	6	1.068	1.055	0.966	1.004	0.957	1.119	0.866	0.941	0.93	0.98	0.85
Q8NI27	THO complex subunit 2 OS=Homo sapie	THOC2	182.77	108.71	9	14	20	14	1.042	1.011	0.948	0.957	1.009	1.01	1.02	1.018	0.93	0.98	0.99
A6NHQ2	rRNA/tRNA 2'-O-methyltransferase fibril	FBLL1	34.803	8.0154	6.6	2	3	1	1.026	0.805	0.734	0.965	1.301	0.69	1.379	1.156	0.93	1.09	1.38
Q9NZ01	Very-long-chain enoyl-CoA reductase OS	TECR	36.034	40.247	17.9	6	10	6	1.082	1.011	0.939	1.003	1.007	0.919	1.041	0.998	0.93	0.92	0.97
P21675	Transcription initiation factor TFIID subu	TAF1	212.67	12.217	0.9	2	2	2	1.109	1.075	1.045	0.981	0.929	0.91	1.007	0.933	0.93	0.84	0.89
Q9NW82	WD repeat-containing protein 70 OS=Ho	WDR70	73.2	76.377	20.2	11	13	11	1.038	1.104	0.984	1.003	0.951	1.148	0.905	1.031	0.93	0.98	0.90
P23497	Nuclear autoantigen Sp-100 OS=Homo s	SP100	100.42	33.887	8.5	7	8	5	0.973	1.099	0.902	1.02	0.91	1.101	0.924	1.154	0.93	0.97	1.00
P06756	Integrin alpha-V OS=Homo sapiens OX=	ITGAV	116.04	100.89	16	13	13	12	1.023	1.002	0.929	0.949	1.079	1.146	0.951	0.976	0.93	1.10	0.95
Q9NY61	Protein AATF OS=Homo sapiens OX=96	AATF	63.132	43.351	12	5	6	5	1.036	1.044	0.965	0.964	1.013	1.041	0.985	0.993	0.93	0.99	0.95
Q9UNX4	WD repeat-containing protein 3 OS=Hon	WDR3	106.1	86.72	14.8	10	12	10	1.032	1.019	0.92	0.982	0.993	1.161	0.876	0.98	0.93	1.05	0.90
Q9NP97	Dynein light chain roadblock-type 1 OS=	DYNLRB1	10.921	6.3651	12.5	1	1	1	1.074	0.99	0.946	0.968	0.967	1.236	0.774	1.084	0.93	1.07	0.90
Q8NFF5	FAD synthase OS=Homo sapiens OX=96	FLAD1	65.265	57.319	16.7	8	8	8	1.05	1.012	0.951	0.961	1.081	1.053	0.965	1.034	0.93	1.03	0.97
Q96MF7	E3 SUMO-protein ligase NSE2 OS=Hon	NSMCE2	27.932	6.2945	3.2	1	1	1	1.007	1.054	0.956	0.955	0.984	0.947	1.154	1.032	0.93	0.94	1.06
Q9BVI4	Nucleolar complex protein 4 homolog OS	NOC4L	58.467	100.39	24	11	14	11	0.945	0.99	0.871	0.923	1.047	1.1	1.146	1.051	0.93	1.11	1.14
P42285	Exosome RNA helicase MTR4 OS=Hom	MTREX	117.8	159.33	20.4	18	23	18	1.049	1.007	0.954	0.952	0.997	1.117	0.969	0.975	0.93	1.03	0.95
P28331	NADH-ubiquinone oxidoreductase 75 kD	NDUFS1	79.467	154.1	30.5	15	22	15	1.041	1.053	0.951	0.99	0.991	1.183	0.797	0.949	0.93	1.04	0.83
O43324	Eukaryotic translation elongation factor 1	EEF1E1	19.81	26.413	13.2	3	4	3	1.091	1.055	0.957	1.032	0.946	0.953	0.935	0.999	0.93	0.88	0.90
P16104	Histone H2AX OS=Homo sapiens OX=9	H2AFX	15.144	13.084	12.6	3	18	2	0.976	0.784	0.645	0.986	1.192	0.846	1.267	1.398	0.93	1.16	1.51
Q13443	Disintegrin and metalloproteinase domain	ADAM9	90.555	54.621	13.6	8	8	7	1.015	1.044	0.979	0.929	0.969	1.15	1.008	1.016	0.93	1.03	0.98
P35268	60S ribosomal protein L22 OS=Homo sa	RPL22	14.787	24.191	28.1	3	5	3	1.023	1.035	0.988	0.919	1.025	1.095	1.061	0.958	0.93	1.03	0.98
Q9NZI8	Insulin-like growth factor 2 mRNA-bindi	IGF2BP1	63.48	194.85	35.9	19	37	17	1.018	1.106	1.003	0.965	0.945	1.005	0.993	0.986	0.93	0.92	0.93
Q08752	Peptidyl-prolyl cis-trans isomerase D OS	PPID	40.763	109.98	42.7	15	20	14	1.059	1.101	1.03	0.971	0.98	1	0.943	0.927	0.93	0.92	0.87
Q8N335	Glycerol-3-phosphate dehydrogenase 1-li	GPD1L	38.418	12.104	4.8	2	2	2	1.007	1.055	0.912	0.998	0.99	0.953	1.056	1.089	0.93	0.94	1.04
O75340	Programmed cell death protein 6 OS=Ho	PDCD6	21.868	46.56	28.3	5	5	5	1.028	1.087	1.014	0.945	1.012	1.234	0.749	0.922	0.93	1.06	0.79
Q99829	Copine-1 OS=Homo sapiens OX=9606 (	CPNE1	59.058	48.336	11.2	7	10	7	1.077	1.078	1.03	0.966	0.968	0.88	0.998	0.971	0.93	0.86	0.91
O94925	Glutaminase kidney isoform, mitochondri	GLS	73.46	144.5	17.5	9	16	9	1.04	1.099	1.003	0.978	0.941	0.955	1.072	0.99	0.93	0.89	0.96
Q96PU4	E3 ubiquitin-protein ligase UHRF2 OS=I	UHRF2	89.984	27.558	5.1	4	4	3	0.977	0.888	0.792	0.935	1.196	1.151	1.003	1.191	0.93	1.26	1.18
Q9H7C9	Mth938 domain-containing protein OS=I	AAMDC	13.332	14.881	21.3	2	2	2	1.042	1.363	1.152	1.075	0.63	0.958	0.909	0.849	0.93	0.66	0.73
O14561	Acyl carrier protein, mitochondrial OS=F	NDUFAB1	17.417	22.939	15.4	3	12	3	1.084	1.037	1.001	0.963	0.979	1.058	0.955	0.92	0.93	0.96	0.88
Q5W111	SPRY domain-containing protein 7 OS=I	SPRYD7	21.666	22.792	19.9	3	3	3	1.045	0.873	0.721	1.055	1.166	0.978	0.931	1.219	0.93	1.12	1.12
P49411	Elongation factor Tu, mitochondrial OS=	TUFM	49.541	191.46	46.5	18	34	18	1.057	1.05	0.978	0.973	1.027	1.161	0.87	0.994	0.93	1.04	0.88
Q96H79	Zinc finger CCCH-type antiviral protein	ZC3HAV1I	32.962	13.9	5.7	2	2	2	1.114	0.897	0.977	0.885	1.042	1.307	0.901	0.931	0.93	1.17	0.91
Q69YN2	CWF19-like protein 1 OS=Homo sapiens	CWF19L1	60.618	54.004	16.7	6	8	6	1.031	1.02	0.95	0.949	1.016	0.896	1.099	1.037	0.93	0.93	1.04
O60716	Catenin delta-1 OS=Homo sapiens OX=9	CTNND1	108.17	308.68	38.3	26	38	26	1.015	1.128	0.979	1.005	0.902	0.981	1.007	1.049	0.93	0.88	0.96
P17066	Heat shock 70 kDa protein 6 OS=Homo s	HSPA6	71.027	16.299	12.3	8	34	2	1.041	0.967	0.955	0.904	1.06	1.044	1.084	1.035	0.93	1.05	1.06
O75717	WD repeat and HMG-box DNA-binding	WDHD1	125.97	145.86	19.8	18	20	18	1.036	0.981	0.914	0.953	1.062	1.007	1.033	1.08	0.93	1.03	1.05
Q5HY92	Fidgetin OS=Homo sapiens OX=9606 G	FIGN	82.145	8.326	1.4	1	1	1	1.091	1.001	0.974	0.962	1.096	0.983	0.935	0.974	0.93	0.99	0.91
Q14683	Structural maintenance of chromosomes p	SMC1A	143.23	294.55	31.2	36	49	36	1.039	1.023	0.939	0.969	0.978	0.995	0.973	1.016	0.93	0.96	0.96
Q9BWD1	Acetyl-CoA acetyltransferase, cytosolic (	ACAT2	41.35	122.62	16.9	5	7	5	0.922	1.058	0.858	0.974	1.045	1.01	1.156	0.963	0.93	1.04	1.07
O75436	Vacuolar protein sorting-associated prote	VPS26A	38.169	94.354	30	7	9	7	1.027	1.097	1.032	0.933	0.919	0.976	1.073	0.95	0.93	0.89	0.95
Q9UN79	Transcription factor SOX-13 OS=Homo s	SOX13	69.228	65.838	21.4	10	11	9	1.004	0.982	0.901	0.936	1.008	1.267	0.929	0.988	0.92	1.15	0.97
Q9P2W9	Syntaxin-18 OS=Homo sapiens OX=960	STX18	38.673	34.238	15.5	5	5	5	1.044	1.075	1.022	0.938	1.063	1.036	0.943	0.977	0.92	0.99	0.91
Q99729	Heterogeneous nuclear ribonucleoprotein	HNRNPAB	36.224	104.28	28.3	10	19	10	1.027	1.07	0.993	0.946	1.008	1.041	0.969	0.955	0.92	0.98	0.92
Q9H2Y7	Zinc finger protein 106 OS=Homo sapien	ZNF106	208.88	20.28	1.6	3	3	3	1.029	1.026	0.914	0.986	0.979	0.823	1.24	1.049	0.92	0.88	1.11
O43805	Sjoegren syndrome nuclear autoantigen 1	SSNA1	13.596	14.45	16	2	4	2	1.073	1.047	0.949	1.011	1.009	0.907	0.91	1.042	0.92	0.90	0.92
O76080	AN1-type zinc finger protein 5 OS=Hom	ZFAND5	23.132	9.814	8.5	1	1	1	1.036	1.123	1.009	0.987	0.989	1.222	0.744	0.938	0.92	1.02	0.78
Q9C0J8	pre-mRNA 3' end processing protein WD	WDR33	145.89	69.213	7.8	9	12	9	1.113	1.031	0.986	0.996	0.986	1.162	0.782	0.889	0.92	1.00	0.78
Q15907	Ras-related protein Rab-11B OS=Homo s	RAB11B	24.488	82.317	46.8	10	17	9	1.047	1.079	0.981	0.984	0.995	0.962	0.967	1.015	0.92	0.92	0.93
Q9NSK0	Kinesin light chain 4 OS=Homo sapiens	KLC4	68.639	14.146	12.3	6	7	2	0.944	1.324	0.981	1.115	0.586	1.286	0.659	1.151	0.92	0.83	0.80
P11021	Endoplasmic reticulum chaperone BiP O	HSPA5	72.332	323.31	55.5	33	104	32	1.044	1.038	0.942	0.982	1.016	0.999	0.974	0.996	0.92	0.97	0.95
Q86SQ9	Dehydrodolichyl diphosphate synthase cc	DHDDS	38.657	20.7	8.7	3	3	3	1.036	0.993	0.935	0.94	1.187	0.745	1.232	1	0.92	0.95	1.10
Q96BP2	Coiled-coil-helix-coiled-coil-helix domai	CHCHD1	13.475	16.596	22.9	2	3	2	0.987	1.118	1.023	0.922	1.049	1.307	0.796	0.932	0.92	1.12	0.82
Q15365	Poly(rC)-binding protein 1 OS=Homo sa	PCBP1	37.497	210.96	59.3	13	45	9	0.984	1.081	0.945	0.963	0.941	0.971	1.032	1.03	0.92	0.93	1.00
Q93100	Phosphorylase b kinase regulatory subun	PHKB	124.88	82.266	12.7	12	14	11	1.045	1.032	0.948	0.971	0.948	0.967	0.945	1.028	0.92	0.92	0.95
Q6P3X3	Tetratricopeptide repeat protein 27 OS=F	TTC27	96.631	11.692	3.1	2	2	2	1.087	1.041	0.978	0.988	1.042	0.947	1.037	0.873	0.92	0.93	0.90
P60228	Eukaryotic translation initiation factor 3	EIF3E	52.22	145.23	36.2	14	24	14	1.067	1.083	1	0.986	0.943	0.999	0.965	0.978	0.92	0.90	0.90
P51812	Ribosomal protein S6 kinase alpha-3 OS	RPS6KA3	83.735	32.57	10.7	5	4	4	1.074	1.022	0.883	1.053	0.918	0.843	1.103	1.067	0.92	0.84	1.04
O43665	Regulator of G-protein signaling 10 OS=	RGS10	20.236	14.676	12.7	2	2	2	1.002	0.976	0.812	1.015	0.998	0.989	1.101	1.159	0.92	1.00	1.14
Q14694	Ubiquitin carboxyl-terminal hydrolase 10	USP10	87.133	102.99	21.8	13	16	13	1.05	1.045	0.939	0.996	0.983	1.099	0.867	0.976	0.92	0.99	0.88
Q86XP3	ATP-dependent RNA helicase DDX42 O	DDX42	102.97	239.78	27.5	20	27	20	1.021	1.074	0.957	0.978	0.965	0.977	0.949	0.978	0.92	0.93	0.92
Q8IWB7	WD repeat and FYVE domain-containing	WDFY1	46.323	55.543	20	7	11	7	1.023	1.098	1.001	0.958	0.96	1.032	0.975	0.979	0.92	0.94	0.92
Q9H5Q4	Dimethyladenosine transferase 2, mitoch	TFB2M	45.348	21.083	9.3	3	3	3	1.111	0.93	0.925	0.96	0.996	0.898	1.151	0.97	0.92	0.93	1.04
Q13418	Integrin-linked protein kinase OS=Homo	ILK	51.419	83.647	22.3	9	15	9	1.029	1.075	0.983	0.96	0.975	1.003	1.054	1.009	0.92	0.94	0.98
Q14103	Heterogeneous nuclear ribonucleoprotein	HNRNPD	38.434	168.29	33.5	13	28	13	1.054	1.086	0.994	0.982	0.933	1.081	0.953	0.948	0.92	0.94	0.89
P04183	Thymidine kinase, cytosolic OS=Homo s	TK1	25.468	49.651	26.9	7	7	7	0.895	0.709	0.598	0.883	1.303	0.977	1.265	1.592	0.92	1.42	1.78
Q9NVX2	Notchless protein homolog 1 OS=Homo s	NLE1	53.32	97.45	26.2	10	10	10	0.999	1.038	0.899	0.981	0.993	1.115	1.052	0.962	0.92	1.03	0.99
P50213	Isocitrate dehydrogenase [NAD] subunit	IDH3A	39.591	52.013	19.4	6	10	6	1.051	1.048	0.99	0.947	1.028	1.19	0.943	1.011	0.92	1.06	0.93
P31153	S-adenosylmethionine synthase isoform t	MAT2A	43.66	84.449	36.7	10	15	10	0.977	1.062	0.906	0.975	0.959	1.047	0.972	0.974	0.92	0.98	0.95
P62837	Ubiquitin-conjugating enzyme E2 D2 OS	UBE2D2	16.735	27.235	19.7	2	4	1	1.073	1.103	0.978	1.029	0.909	1.17	0.78	0.946	0.92	0.96	0.79
Q6ZNA5	Ferric-chelate reductase 1 OS=Homo sap	FRRS1	66.114	12.034	2.7	2	3	2	0.999	0.906	0.716	1.041	1.026	1.287	0.768	1.304	0.92	1.21	1.09
Q8IYH5	ZZ-type zinc finger-containing protein 3	ZZZ3	102.02	6.0798	1.3	1	1	1	0.967	1.194	1.078	0.915	0.952	0.979	1.106	0.96	0.92	0.89	0.96
Q96PC3	AP-1 complex subunit sigma-3 OS=Hom	AP1S3	18.28	6.1832	6.5	1	1	1	1.083	0.999	0.907	1.013	1.031	0.978	1.093	0.872	0.92	0.96	0.94
Q8WVK2	U4/U6.U5 small nuclear ribonucleoprotei	SNRNP27	18.86	15.548	19.4	2	2	2	0.951	1.09	0.977	0.905	1.125	1.411	0.802	0.912	0.92	1.24	0.84
O95239	Chromosome-associated kinesin KIF4A (	KIF4A	139.88	199.31	22.4	25	32	16	1.037	1.097	1.003	0.964	0.958	0.976	0.943	0.927	0.92	0.91	0.88
Q04724	Transducin-like enhancer protein 1 OS=I	TLE1	83.2	31.123	10.9	6	6	4	1.029	1.027	0.863	1.032	0.991	1.027	0.946	1.165	0.92	0.98	1.03
Q9P0V3	SH3 domain-binding protein 4 OS=Homo	SH3BP4	107.49	63.872	10.4	8	9	8	1.102	1.117	1.028	1.017	0.972	0.917	1.033	0.912	0.92	0.85	0.88
P57772	Selenocysteine-specific elongation factor	EEFSEC	65.304	45.47	10.7	5	6	5	1.065	1.038	0.984	0.954	0.994	1.13	0.854	1.067	0.92	1.01	0.91
P50395	Rab GDP dissociation inhibitor beta OS=	GDI2	50.663	236.72	44.7	19	42	14	1.075	1.013	0.951	0.973	0.963	0.925	1.083	0.997	0.92	0.90	1.00
P47897	Glutamine--tRNA ligase OS=Homo sapie	QARS	87.798	178.02	27.9	19	26	17	1.064	1.074	0.985	0.985	0.987	0.967	0.972	0.96	0.92	0.91	0.90
Q96EY7	Pentatricopeptide repeat domain-containi	PTCD3	78.549	115.15	21.3	13	18	13	1.058	1.052	1.007	0.937	0.999	1.153	0.86	0.981	0.92	1.02	0.87
P78345	Ribonuclease P protein subunit p38 OS=	RPP38	31.834	40.845	20.8	6	7	6	1.054	0.992	0.959	0.926	1.067	0.926	1.113	1.069	0.92	0.97	1.07
Q13393	Phospholipase D1 OS=Homo sapiens OX	PLD1	124.18	24.241	4	3	3	3	1.006	1	0.984	0.864	1.096	0.924	1.141	1.144	0.92	1.01	1.14
Q96HR8	H/ACA ribonucleoprotein complex non-c	NAF1	53.716	7.3279	2	1	2	1	1.033	0.997	0.921	0.949	0.974	0.924	1.207	1.068	0.92	0.93	1.12
Q07817	Bcl-2-like protein 1 OS=Homo sapiens C	BCL2L1	26.049	6.0235	3.9	1	1	1	0.969	0.896	0.733	0.985	1.055	0.901	1.198	1.368	0.92	1.05	1.38
Q12907	Vesicular integral-membrane protein VIP	LMAN2	40.228	108.57	34	10	17	10	1.025	1	0.903	0.962	0.99	0.996	1.08	1.04	0.92	0.98	1.05
Q9NXC5	GATOR complex protein MIOS OS=Hor	MIOS	98.583	34.73	5.9	5	5	5	1.089	0.986	0.907	1.004	0.99	0.995	0.959	1.061	0.92	0.96	0.97
Q16513	Serine/threonine-protein kinase N2 OS=I	PKN2	112.03	51.076	8.8	7	7	7	1.062	0.974	0.931	0.944	1.069	1.141	0.857	1.017	0.92	1.09	0.92
P38117	Electron transfer flavoprotein subunit bet	ETFB	27.843	108.61	51.4	12	23	11	1.057	0.975	0.9	0.971	1.07	1.137	0.867	1.04	0.92	1.09	0.94
O43676	NADH dehydrogenase [ubiquinone] 1 be	NDUFB3	11.402	15.169	21.4	2	3	2	1.047	1.093	1.01	0.96	0.961	1.126	0.843	0.942	0.92	0.98	0.83
Q9NZD8	Maspardin OS=Homo sapiens OX=9606	SPG21	34.96	55.489	26.9	6	5	5	1.047	1.089	0.984	0.982	0.836	1.011	0.926	1.055	0.92	0.86	0.93
Q9NQH7	Xaa-Pro aminopeptidase 3 OS=Homo sa	XPNPEP3	57.033	24.764	8.7	3	4	3	0.966	0.979	0.826	0.964	1.071	0.929	1.105	1.248	0.92	1.03	1.21
O95999	B-cell lymphoma/leukemia 10 OS=Homo	BCL10	26.251	12.889	8.6	2	3	2	1.104	1.04	1.024	0.949	1.091	0.886	1.029	0.888	0.92	0.92	0.89
P41743	Protein kinase C iota type OS=Homo sap	PRKCI	68.262	86.44	22.7	9	9	7	1.005	1.154	0.986	1	0.886	0.982	0.962	1.005	0.92	0.87	0.91
P09417	Dihydropteridine reductase OS=Homo sa	QDPR	25.789	78.613	36.9	6	10	6	1.072	1.036	0.974	0.965	0.971	0.918	0.996	0.957	0.92	0.90	0.93
Q99569	Plakophilin-4 OS=Homo sapiens OX=96	PKP4	131.87	24.317	3.5	4	4	4	1.004	1.012	0.862	0.992	1.054	0.974	1.132	1.053	0.92	1.01	1.08
Q8IYA6	Cytoskeleton-associated protein 2-like O	CKAP2L	83.586	11.919	3.1	2	2	2	0.941	0.912	0.855	0.849	1.057	1.075	1.268	1.271	0.92	1.15	1.37
P62987	Ubiquitin-60S ribosomal protein L40 OS	UBA52	14.728	-2	43.8	5	20	1	0.951	0.86	0.741	0.924	1.279	0.753	1.226	1.429	0.92	1.12	1.47
P62745	Rho-related GTP-binding protein RhoB (	RHOB	22.123	25.651	27.6	4	5	3	1.052	0.957	0.89	0.957	1.064	0.984	0.99	1.09	0.92	1.02	1.04
Q9H147	Deoxynucleotidyltransferase terminal-int	DNTTIP1	37.013	35.775	14	3	4	3	1.031	1.164	1.015	1.003	0.874	1.075	0.865	0.91	0.92	0.89	0.81

Q9UBP9	PTB domain-containing engulfment adap	GULP1	34.49	23.858	13.5	4	4	4	1.02	1.075	0.969	0.957	1.028	1.223	0.874	0.931	0.92	1.07	0.86
P51649	Succinate-semialdehyde dehydrogenase, r	ALDH5A1	57.214	39.203	8.4	3	3	3	0.946	1.244	1.076	0.937	0.908	1.132	1.008	0.903	0.92	0.93	0.87
Q9P287	BRCA2 and CDKN1A-interacting protei	BCCIP	35.979	65.667	22.6	5	5	5	1.075	1.016	0.961	0.961	0.937	0.968	1.105	0.965	0.92	0.91	0.99
O75794	Cell division cycle protein 123 homolog (	CDC123	39.134	33.335	10.7	3	4	3	1.114	0.964	0.949	0.961	1.084	0.904	0.974	1.052	0.92	0.96	0.97
P00403	Cytochrome c oxidase subunit 2 OS=Hor	MT-CO2	25.565	30.346	20.3	4	6	4	1.083	1.092	0.986	1.013	0.986	1.027	0.901	0.897	0.92	0.93	0.83
O75616	GTPase Era, mitochondrial OS=Homo sa	ERAL1	48.349	61.247	15.6	5	6	5	1.084	1.115	1.044	0.977	1.017	1.388	0.62	0.771	0.92	1.09	0.63
O75414	Nucleoside diphosphate kinase 6 OS=Ho	NME6	21.142	14.294	11.8	2	3	2	1.003	1.072	0.935	0.972	0.978	1.232	0.86	0.94	0.92	1.07	0.87
O15143	Actin-related protein 2/3 complex subuni	ARPC1B	40.949	89.335	30.6	9	15	9	1.027	1.059	0.878	1.039	0.969	1.048	0.948	1.018	0.92	0.97	0.94
P46940	Ras GTPase-activating-like protein IQGA	IQGAP1	189.25	323.31	39.9	57	120	53	1.028	1.041	0.933	0.968	0.956	0.966	1.082	1.059	0.92	0.93	1.03
O60573	Eukaryotic translation initiation factor 4E	EIF4E2	28.362	41.36	30.2	5	5	5	1.069	1.143	1.016	1.016	0.895	0.912	0.925	0.879	0.92	0.82	0.82
Q92879	CUGBP Elav-like family member 1 OS=I	CELF1	52.063	83.226	18.7	9	13	8	1.063	1.099	1.009	0.977	0.961	1.031	0.941	1.016	0.92	0.92	0.91
Q9UGM6	Tryptophan--tRNA ligase, mitochondrial	WARS2	40.146	17.323	10.3	3	3	3	1.018	1.13	0.939	1.034	1.103	1.018	0.925	1.122	0.92	0.99	0.95
P24468	COUP transcription factor 2 OS=Homo s	NR2F2	45.571	14.563	4.6	2	2	2	1.012	1.098	0.929	1.009	1.067	0.843	1.076	1.009	0.92	0.91	0.99
Q96G74	OTU domain-containing protein 5 OS=H	OTUD5	60.625	19.139	8.4	3	3	3	1.082	1.089	1.021	0.973	0.946	0.902	1.061	0.942	0.92	0.85	0.92
Q13243	Serine/arginine-rich splicing factor 5 OS=	SRSF5	31.263	16.36	9.6	2	4	2	1.037	0.986	0.957	0.901	1.026	0.919	1.1	1.005	0.92	0.96	1.04
P54819	Adenylate kinase 2, mitochondrial OS=H	AK2	26.477	86.596	40.6	8	15	8	1.034	1.049	0.952	0.961	0.963	1.029	0.969	0.999	0.92	0.96	0.94
P22033	Methylmalonyl-CoA mutase, mitochondr	MUT	83.134	71.853	14.5	9	12	9	1.025	1.03	0.926	0.961	1.032	1.284	0.815	0.98	0.92	1.13	0.87
Q13948	Protein CASP OS=Homo sapiens OX=96	CUX1	77.454	45.983	8.8	5	5	5	1.016	0.956	0.833	0.977	1.149	1.056	0.921	1.163	0.92	1.12	1.06
Q9P2X0	Dolichol-phosphate mannosyltransferase	DPM3	10.094	7.3025	10.9	1	1	1	1.039	0.917	0.854	0.941	1.16	0.947	1.177	1.033	0.92	1.08	1.13
Q9Y6R4	Mitogen-activated protein kinase kinase k	MAP3K4	181.68	15.024	1.4	2	3	2	0.971	0.948	0.666	1.095	0.971	0.969	1.059	1.346	0.92	1.01	1.25
Q8N766	ER membrane protein complex subunit 1	EMC1	111.76	135.24	18	14	19	14	1.051	1.05	0.92	1.008	1.019	0.945	1.026	1.08	0.92	0.93	1.00
Q9UBW7	Zinc finger MYM-type protein 2 OS=Ho	ZMYM2	154.91	63.384	7.7	10	12	10	1.061	1.209	1.058	1.025	0.894	1.113	0.709	0.879	0.92	0.88	0.70
O00541	Pescadillo homolog OS=Homo sapiens C	PES1	68.002	71.917	18.5	10	14	10	0.987	1.039	0.923	0.936	1.063	1.047	1.007	1.006	0.92	1.04	0.99
P0DPI2	Glutamine amidotransferase-like class 1 c	GATD3A	28.17	212.4	40.7	9	13	9	1.072	1.134	1.064	0.96	0.975	1.161	0.839	0.863	0.92	0.97	0.77
Q9Y2D4	Exocyst complex component 6B OS=Hor	EXOC6B	94.2	66.804	11.2	8	11	8	1.039	1.009	0.948	0.931	1.054	0.887	1.143	1.072	0.92	0.95	1.08
Q14444	Caprin-1 OS=Homo sapiens OX=9606 G	CAPRIN1	78.365	102.16	22	10	14	10	1.048	1.108	1.004	0.974	0.912	0.959	1.024	0.935	0.92	0.87	0.91
Q13630	GDP-L-fucose synthase OS=Homo sapier	TSTA3	35.892	25.951	10.3	2	2	2	1.124	1.175	1.05	1.059	0.885	0.953	0.801	0.875	0.92	0.80	0.73
Q8TCS8	Polyribonucleotide nucleotidyltransferase	PNPT1	85.95	119.4	24.1	15	17	15	1.07	1.056	0.999	0.951	0.969	0.958	0.929	0.937	0.92	0.91	0.88
P82921	28S ribosomal protein S21, mitochondria	MRPS21	10.688	33.202	29.9	2	6	2	1.028	1.012	0.977	0.894	1.096	0.965	1.03	1.047	0.92	1.01	1.02
P07741	Adenine phosphoribosyltransferase OS=I	APRT	19.608	33.094	40	5	10	4	1.075	1.032	0.96	0.972	0.96	1.011	0.887	1.048	0.92	0.94	0.92
Q9NQS7	Inner centromere protein OS=Homo sapie	INCENP	105.43	59.358	9.8	7	9	7	0.966	1.008	0.866	0.944	1.041	1.323	0.916	1.029	0.92	1.20	0.99
P12532	Creatine kinase U-type, mitochondrial OS	CKMT1A	47.036	188.55	23.7	8	15	8	1.02	1.057	0.946	0.958	1.079	1.186	0.923	0.944	0.92	1.09	0.90
Q13555	Calcium/calmodulin-dependent protein k	CAMK2G	62.606	18.646	9	4	5	3	1.139	0.994	1.009	0.946	0.965	0.832	1.087	0.966	0.92	0.84	0.96
Q9H1D9	DNA-directed RNA polymerase III subun	POLR3F	35.684	7.9049	3.8	1	2	1	1.182	0.995	0.989	1.006	0.946	1.178	0.745	0.874	0.92	0.98	0.74
P98194	Calcium-transporting ATPase type 2C me	ATP2C1	100.58	39.978	7.5	5	6	5	1.046	1.15	1.046	0.966	0.913	1.049	0.907	0.908	0.92	0.89	0.83
O75691	Small subunit processome component 20	UTP20	318.38	101.24	5.6	12	15	12	0.994	0.912	0.832	0.914	1.131	1.112	0.983	1.217	0.92	1.18	1.15
Q96GC5	39S ribosomal protein L48, mitochondria	MRPL48	23.934	40.964	30.7	6	7	6	1.068	1.047	1.004	0.933	0.986	1.21	0.805	0.976	0.92	1.04	0.84
Q12788	Transducin beta-like protein 3 OS=Homo	TBL3	89.034	126.86	28.3	15	16	14	1.053	1.059	0.952	0.982	1.079	1.072	0.845	0.92	0.92	1.02	0.84
Q9NS69	Mitochondrial import receptor subunit TO	TOMM22	15.521	22.228	16.2	2	4	2	0.976	1.205	0.938	1.059	1.107	0.93	1.049	0.839	0.92	0.93	0.87
Q00325	Phosphate carrier protein, mitochondrial	SLC25A3	40.094	88.174	28.2	9	13	9	1.093	1.04	1.016	0.937	1.016	1.229	0.8	0.912	0.92	1.05	0.80
P04049	RAF proto-oncogene serine/threonine-pro	RAF1	73.051	20.052	5.2	3	3	2	0.998	1.014	0.92	0.922	1.094	0.935	1.134	1.106	0.92	1.01	1.11
Q13098	COP9 signalosome complex subunit 1 OS	GPS1	55.536	63.77	15.3	8	9	8	1.02	1.038	0.921	0.963	0.997	1.14	0.853	0.982	0.92	1.04	0.89
Q9BTE6	Alanyl-tRNA editing protein Aarsd1 OS=	AARSD1	45.479	27.62	17.5	5	4	4	1.045	0.976	0.959	0.891	1.06	1.17	0.901	0.897	0.92	1.10	0.89
P05141	ADP/ATP translocase 2 OS=Homo sapie	SLC25A5	32.852	52.496	34.2	11	21	4	1.027	1.041	0.935	0.958	1.054	1.223	0.889	0.97	0.92	1.10	0.90
Q01085	Nucleolysin TIAR OS=Homo sapiens OX	TIAL1	41.59	42.508	24	7	13	5	1	1.044	0.912	0.959	1.019	1.02	1.008	0.948	0.92	1.00	0.96
Q9Y5K8	V-type proton ATPase subunit D OS=Ho	ATP6V1D	28.262	76.85	31.6	6	7	6	1.003	1.088	0.958	0.956	0.991	0.889	1.157	0.988	0.92	0.90	1.03
Q4ZIN3	Membralin OS=Homo sapiens OX=9606	TMEM259	67.888	20.116	6	2	2	2	1.073	0.997	0.85	1.044	1.093	0.871	0.935	1.111	0.91	0.95	0.99
P55268	Laminin subunit beta-2 OS=Homo sapier	LAMB2	195.98	90.928	7.6	11	11	11	1.049	1.05	0.966	0.954	0.963	1.062	0.901	0.947	0.91	0.96	0.88
O00178	GTP-binding protein 1 OS=Homo sapien	GTPBP1	72.453	47.662	11.1	6	7	6	1.042	1.028	1.023	0.87	0.921	1.008	0.995	1.106	0.91	0.93	1.01
Q5T0N5	Formin-binding protein 1-like OS=Homo	FNBP1L	70.065	59.793	16.2	8	8	8	1.054	1.165	0.976	1.053	0.841	0.96	0.956	0.979	0.91	0.81	0.87
P11387	DNA topoisomerase 1 OS=Homo sapiens	TOP1	90.725	181.63	26.9	19	35	18	1.04	1.023	0.942	0.944	1.089	1.021	0.937	1.048	0.91	1.02	0.96
Q6P587	Acylpyruvase FAHD1, mitochondrial OS	FAHD1	24.843	23.95	15.2	3	3	3	0.975	1.098	0.981	0.914	1	1.046	1.036	1.104	0.91	0.99	1.03
Q13371	Phosducin-like protein OS=Homo sapien	PDCL	34.281	12.411	7.3	2	2	2	1.277	0.854	0.925	1.023	1.185	0.821	0.839	0.896	0.91	0.94	0.81
P62273	40S ribosomal protein S29 OS=Homo sa	RPS29	6.6767	19.026	33.9	2	4	2	1.041	1.199	1.088	0.959	0.867	1.325	0.707	0.839	0.91	0.98	0.69
Q9UPN9	E3 ubiquitin-protein ligase TRIM33 OS=	TRIM33	122.53	110.66	14.4	14	19	14	1.041	1.081	0.976	0.963	0.966	1.144	0.891	0.98	0.91	0.99	0.88
Q6UWE0	E3 ubiquitin-protein ligase LRSAM1 OS	LRSAM1	83.593	105.69	16.9	10	11	10	1.001	1.12	0.953	0.985	0.985	0.941	0.87	0.975	0.91	0.91	0.87
Q86Y56	Dynein assembly factor 5, axonemal OS=	DNAAF5	93.52	63.559	11.9	8	8	8	1.128	1.071	1.059	0.95	1.039	0.96	0.895	0.915	0.91	0.91	0.82
P47813	Eukaryotic translation initiation factor 1A	EIF1AX	16.46	7.5183	33.3	5	8	1	1.076	1.047	1.011	0.928	0.893	1.19	0.887	1.031	0.91	0.98	0.90
Q9NVD7	Alpha-parvin OS=Homo sapiens OX=96(	PARVA	42.243	32.135	13.2	4	6	4	1.102	1.037	0.97	0.982	0.932	0.945	1.083	0.949	0.91	0.88	0.95
Q9H3P7	Golgi resident protein GCP60 OS=Homo	ACBD3	60.593	58.951	15.9	7	9	6	1.023	1.126	0.938	1.023	0.905	1.289	0.638	1.067	0.91	1.02	0.79
P82650	28S ribosomal protein S22, mitochondria	MRPS22	41.28	72.349	31.4	10	15	10	1.064	1.026	0.928	0.979	1.007	1.214	0.854	0.982	0.91	1.06	0.88
Q5C9Z4	Nucleolar MIF4G domain-containing pro	NOM1	96.256	41.197	6.7	5	5	5	0.979	0.856	0.752	0.922	1.231	0.997	1.12	1.309	0.91	1.21	1.32
Q9Y5J7	Mitochondrial import inner membrane tra	TIMM9	10.378	31.033	38.2	3	7	3	1.053	1.006	0.891	0.987	1.03	1.161	0.791	1.045	0.91	1.06	0.89
P49773	Histidine triad nucleotide-binding protein	HINT1	13.802	28.177	34.1	4	5	4	1.094	1.021	0.924	1.005	1.004	1.174	0.845	0.837	0.91	1.03	0.80
Q6PIW4	Fidgetin-like protein 1 OS=Homo sapien	FIGNL1	74.076	20.017	5.5	3	3	3	1.028	0.984	0.909	0.926	1.065	1.161	0.96	1.066	0.91	1.11	1.01
Q92615	La-related protein 4B OS=Homo sapiens	LARP4B	80.551	69.709	15.4	10	12	10	1.03	1.024	0.914	0.959	1.027	1.005	0.966	0.993	0.91	0.99	0.95
Q9Y2V7	Conserved oligomeric Golgi complex sub	COG6	73.278	36.921	8.2	5	5	5	1.056	1.008	0.906	0.976	1.025	1.129	0.921	1.018	0.91	1.04	0.94
Q7Z3B4	Nucleoporin p54 OS=Homo sapiens OX=	NUP54	55.435	91.863	20.9	9	12	9	1.037	1.037	0.921	0.97	1.009	1.099	0.894	1.006	0.91	1.02	0.92
Q15738	Sterol-4-alpha-carboxylate 3-dehydrogen	NSDHL	41.9	118.14	24.9	7	12	7	0.999	1.108	0.971	0.95	1.059	1.152	0.941	1.039	0.91	1.05	0.94
Q9UK39	Nocturnin OS=Homo sapiens OX=9606	NOCT	48.195	20.949	9.3	3	3	3	1.018	1.29	1.09	1.014	0.866	0.666	1.102	0.992	0.91	0.66	0.91
Q12965	Unconventional myosin-Ie OS=Homo sap	MYO1E	127.06	56.484	8.4	8	10	8	1.036	1.071	0.933	0.987	1.013	1.146	0.883	0.998	0.91	1.02	0.89
O14981	TATA-binding protein-associated factor	BTAF1	206.89	26.16	2.4	4	4	4	1.095	1.021	0.96	0.968	1.009	0.933	1.067	0.954	0.91	0.92	0.96
Q16822	Phosphoenolpyruvate carboxykinase [GT	PCK2	70.698	109.59	23.9	12	23	12	0.994	0.989	0.838	0.968	1.067	1.009	1.179	1.186	0.91	1.05	1.19
O14908	PDZ domain-containing protein GIPC1 (	GIPC1	36.049	32.507	18.3	4	4	4	1.05	1.179	0.961	1.069	0.835	0.872	0.973	1.027	0.91	0.77	0.90
O00743	Serine/threonine-protein phosphatase 6 c	PPP6C	35.144	39.775	21.3	5	7	5	1.066	1.084	0.97	0.988	0.9	1.015	1.02	1.01	0.91	0.89	0.94
Q8NC51	Plasminogen activator inhibitor 1 RNA-b	SERBP1	44.965	136.38	42.6	14	22	14	1.046	1.077	0.99	0.943	0.983	1.057	0.986	0.998	0.91	0.96	0.93
Q9Y3D7	Mitochondrial import inner membrane tra	PAM16	13.825	28.209	27.2	2	2	2	0.944	0.999	0.778	0.991	1.055	0.88	1.068	1.412	0.91	1.00	1.28
O75051	Plexin-A2 OS=Homo sapiens OX=9606	PLXNA2	211.1	44.18	3.9	6	7	6	1.003	1.06	0.955	0.923	1.022	0.967	1.019	1.046	0.91	0.96	1.00
P12955	Xaa-Pro dipeptidase OS=Homo sapiens (	PEPD	54.548	82.459	21.3	9	11	9	1.032	1.12	1.035	0.924	0.948	1.03	0.963	0.979	0.91	0.92	0.90
Q99661	Kinesin-like protein KIF2C OS=Homo sa	KIF2C	81.312	141.25	28.4	15	18	14	0.986	0.953	0.896	0.869	1.145	1.319	0.881	1.099	0.91	1.27	1.02
P16930	Fumarylacetoacetase OS=Homo sapiens (	FAH	46.374	35.593	13.1	5	7	5	1.144	1.083	1.102	0.925	0.954	1.166	0.673	0.868	0.91	0.95	0.69
P78318	Immunoglobulin-binding protein 1 OS=H	IGBP1	39.221	35.455	13.6	4	5	4	1.075	1.084	0.919	1.046	0.869	0.863	1.126	1.063	0.91	0.80	1.01
Q9BYT8	Neurolysin, mitochondrial OS=Homo sap	NLN	80.651	113.32	21.2	13	20	13	1.069	1.032	0.981	0.931	0.944	0.95	1.096	0.988	0.91	0.90	0.99
Q6RFH5	WD repeat-containing protein 74 OS=Ho	WDR74	42.441	70.759	20.3	5	5	5	0.966	1.054	0.909	0.929	0.988	1.297	0.793	1.004	0.91	1.13	0.89
Q8NCC3	Group XV phospholipase A2 OS=Homo	PLA2G15	46.657	33.246	12.1	4	5	4	1.044	1.096	0.98	0.967	1.033	1.1	0.869	0.964	0.91	1.00	0.86
Q9UKA9	Polypyrimidine tract-binding protein 2 O	PTBP2	57.49	24.542	10.7	3	3	3	1.055	0.985	0.911	0.945	1.077	0.9	1.242	0.933	0.91	0.97	1.07
Q8TEX9	Importin-4 OS=Homo sapiens OX=9606	IPO4	118.71	134.6	16.8	14	21	14	1.037	1.091	0.974	0.962	1.039	0.995	0.901	0.899	0.91	0.96	0.85
Q9BVP2	Guanine nucleotide-binding protein-like	GNL3	61.992	128.79	22.2	11	18	11	1.102	0.998	0.977	0.933	1.088	1.332	0.718	0.856	0.91	1.15	0.75
Q6UXN9	WD repeat-containing protein 82 OS=Ho	WDR82	35.079	28.953	14.7	4	4	4	1.026	1.106	0.927	1.012	0.912	1.266	0.89	0.97	0.91	1.02	0.87
Q9NRP0	Oligosaccharyltransferase complex subun	OSTC	16.829	23.43	8.1	1	2	1	1.026	1.194	0.976	1.043	0.777	0.965	1.029	1.006	0.91	0.78	0.92
Q15054	DNA polymerase delta subunit 3 OS=Ho	POLD3	51.4	32.388	12	5	5	5	0.922	0.998	0.747	0.999	1.022	1.239	0.808	1.371	0.91	1.18	1.13
Q9NZB8	Molybdenum cofactor biosynthesis prote	MOCS1	70.104	18.426	5	3	3	3	1.115	1.08	1.036	0.96	1.05	0.819	1.054	0.88	0.91	0.85	0.88
Q9UKR5	Probable ergosterol biosynthetic protein	ERG28	15.864	11.016	12.9	2	2	2	0.932	0.942	0.731	0.973	1.028	1.206	0.853	1.496	0.91	1.19	1.25
O94826	Mitochondrial import receptor subunit TO	TOMM70	67.454	151.95	35.4	15	25	15	1.038	1.077	0.95	0.973	0.986	0.978	0.947	1.006	0.91	0.93	0.92
P47985	Cytochrome b-c1 complex subunit Riesk	UQCRFS1	29.668	47.916	14.2	5	9	5	1.014	1.122	1.009	0.933	0.985	1.223	0.828	0.842	0.91	1.03	0.78
Q9NQ29	Putative RNA-binding protein Luc7-like	LUC7L	43.727	18.605	24.3	8	19	3	1.088	1.111	1.046	0.953	0.982	1.219	0.736	0.895	0.91	1.00	0.74
Q9HCE1	Putative helicase MOV-10 OS=Homo sap	MOV10	113.67	156.35	23.7	19	28	19	1.077	1.043	0.974	0.953	0.985	1.061	0.926	0.982	0.91	0.97	0.90
Q9BY44	Eukaryotic translation initiation factor 2A	EIF2A	64.989	206.75	37.1	15	22	15	1.051	1.09	0.941	1.005	0.956	0.969	0.969	0.991	0.91	0.90	0.92
Q676U5	Autophagy-related protein 16-1 OS=Hom	ATG16L1	68.264	25.354	8.7	4	5	4	1.108	1.021	1.001	0.934	0.992	1.059	1.004	0.905	0.91	0.96	0.90
Q53HL2	Borealin OS=Homo sapiens OX=9606 G	CDCA8	31.323	40.962	20.4	4	5	3	1.019	0.989	0.916	0.909	1.137	1.224	0.805	1.063	0.91	1.18	0.93
P17568	NADH dehydrogenase [ubiquinone] 1 be	NDUFB7	16.402	18.574	21.2	3	4	3	1.016	1.078	0.922	0.981	1.134	1.01	1.189	0.948	0.91	1.02	1.02
P06576	ATP synthase subunit beta, mitochondria	ATP5F1B	56.559	275	47.1	18	44	17	1.029	1.163	1.029	0.963	0.962	1.184	0.929	0.936	0.91	0.98	0.85
P51553	Isocitrate dehydrogenase [NAD] subunit	IDH3G	42.794	22.064	7.6	3	3	3	0.984	1.042	0.897	0.944	0.983	1.399	0.814	1.064	0.91	1.18	0.93
P62318	Small nuclear ribonucleoprotein Sm D3 (	SNRPD3	13.916	89.02	31.7	3	10	3	1.001	1.101	0.979	0.931	1.048	1.048	0.922	1.016	0.91	1.00	0.92
P55347	Homeobox protein PKNOX1 OS=Homo	PKNOX1	47.606	12.723	3.9	2	3	2	1.03	1.157	0.974	1.013	0.954	1.032	0.952	0.917	0.91	0.91	0.85
P38606	V-type proton ATPase catalytic subunit A	ATP6V1A	68.303	117.74	27.9	13	24	13	1.066	1.119	0.997	0.988	0.868	0.922	1.012	0.949	0.91	0.82	0.90
P19404	NADH dehydrogenase [ubiquinone] flavo	NDUFV2	27.391	95.165	42.6	9	16	9	1.071	1.047	0.943	0.981	1.066	0.985	0.949	0.999	0.91	0.97	0.92
P82663	28S ribosomal protein S25, mitochondria	MRPS25	20.116	10.102	11.6	1	2	1	1.02	1.128	0.991	0.96	0.918	0.971	1.089	1	0.91	0.88	0.97
Q8N954	G patch domain-containing protein 11 OS	GPATCH11	33.277	13.78	8.1	2	3	2	1.077	1.016	0.971	0.93	1.077	1.226	0.743	1.019	0.91	1.10	0.84
P82933	28S ribosomal protein S9, mitochondrial	MRPS9	45.834	78.463	29.8	9	15	9	1.045	1.088	0.99	0.947	0.977	1.12	0.874	1.014	0.91	0.98	0.89
Q9UBI6	Guanine nucleotide-binding protein G(I)/	GNG12	8.0061	47.814	56.9	3	8	3	1.037	1.028	0.903	0.972	0.929	0.96	1.031	1.047	0.91	0.91	1.01
Q96MU7	YTH domain-containing protein 1 OS=H	YTHDC1	84.699	21.002	4.4	3	3	3	1.103	1.057	0.938	1.023	1.017	1.153	0.768	0.904	0.91	1.00	0.77
O95155	Ubiquitin conjugation factor E4 B OS=H	UBE4B	146.18	64.998	8.8	10	11	10	1.041	1.073	0.93	0.989	0.91	1.188	0.755	0.996	0.91	0.99	0.83
P15735	Phosphorylase b kinase gamma catalytic	PHKG2	46.442	25.44	10.6	4	6	4	1.043	1.025	0.955	0.922	1.045	0.959	1.119	0.973	0.91	0.97	1.01
Q5VZF2	Muscleblind-like protein 2 OS=Homo sa	MBNL2	40.517	27.003	9.9	4	4	3	0.997	1.015	0.944	0.882	1.133	0.986	1.143	1.05	0.91	1.05	1.09
Q8TDD1	ATP-dependent RNA helicase DDX54 O	DDX54	98.594	80.357	15.1	11	13	11	1.008	1.045	0.982	0.881	1.108	1.232	0.811	1.084	0.91	1.14	0.92
Q9BPX5	Actin-related protein 2/3 complex subuni	ARPC5L	16.941	13.11	13.7	2	3	1	1.01	1.01	0.839	0.994	1	1.028	1.004	1.182	0.91	1.00	1.08
O14777	Kinetochore protein NDC80 homolog OS	NDC80	73.912	48.809	12.1	8	9	8	1.08	1.111	0.983	1.005	1.007	1.011	0.923	0.947	0.91	0.92	0.85
Q9P015	39S ribosomal protein L15, mitochondria	MRPL15	33.419	27.93	15.9	4	5	4	1.093	1.086	0.98	0.997	0.991	1.227	0.678	0.896	0.91	1.02	0.72
Q96QE5	Transcription elongation factor, mitochor	TEFM	41.676	13.497	5.8	2	2	2	0.97	1.142	1.032	0.884	1.106	1.284	0.891	0.859	0.91	1.13	0.83
P13995	Bifunctional methylenetetrahydrofolate d	MTHFD2	37.895	64.567	24.3	6	8	6	0.964	0.997	0.808	0.971	1.069	0.956	1.168	1.28	0.91	1.03	1.25
O60313	Dynamin-like 120 kDa protein, mitochon	OPA1	111.63	105.29	15.9	14	15	14	1.072	1.025	0.928	0.974	1	0.999	0.972	1.028	0.91	0.95	0.95
Q16799	Reticulon-1 OS=Homo sapiens OX=960	RTN1	83.617	11.818	2.3	2	2	2	1.098	1.042	1.03	0.911	0.936	0.93	1.13	0.973	0.91	0.87	0.98
Q9BZV2	Thiamine transporter 2 OS=Homo sapien	SLC19A3	55.664	17.813	12.3	2	2	1	0.981	1.264	1.063	0.973	0.711	1.129	1.074	0.907	0.91	0.82	0.88
Q16891	MICOS complex subunit MIC60 OS=Ho	IMMT	83.677	205.91	37.1	24	28	23	1.054	1.051	0.958	0.951	1.014	1.204	0.899	1.032	0.91	1.05	0.92
Q07666	KH domain-containing, RNA-binding, si	KHDRBS1	48.227	70.792	19.2	9	17	9	0.999	1.105	0.984	0.924	0.927	1.153	0.904	1.026	0.91	0.99	0.92
Q11206	CMP-N-acetylneuraminate-beta-galactosa	ST3GAL4	38.045	11.625	6.9	2	2	2	1.078	1.055	0.982	0.952	0.992	1.016	1.017	0.942	0.91	0.94	0.92
Q9H2H9	Sodium-coupled neutral amino acid trans	SLC38A1	54.047	24.337	8.2	4	4	4	1.05	1.264	1.098	1	0.658	0.94	1.089	0.93	0.91	0.69	0.87
P18669	Phosphoglycerate mutase 1 OS=Homo sa	PGAM1	28.804	250.18	42.1	10	38	10	0.991	1.305	1.081	1	0.712	1.158	0.961	0.904	0.91	0.81	0.81
P49917	DNA ligase 4 OS=Homo sapiens OX=96	LIG4	103.97	8.5708	1.5	1	2	1	1.006	0.966	0.758	1.029	1.042	1.081	1.072	1.08	0.91	1.08	1.09
Q8IWV7	E3 ubiquitin-protein ligase UBR1 OS=H	UBR1	200.21	32.307	2.5	5	6	5	1.033	1.066	0.948	0.954	0.998	0.994	1.061	1.015	0.91	0.95	0.99
Q9NVP1	ATP-dependent RNA helicase DDX18 O	DDX18	75.406	214.67	38.7	19	25	16	1.011	0.992	0.894	0.921	1.01	1.216	0.929	1.104	0.91	1.11	1.01
Q9NZJ0	Denticleless protein homolog OS=Homo	DTL	79.467	10.991	2.9	2	2	2	0.872	0.678	0.507	0.897	1.201	1.116	1.159	1.835	0.91	1.49	1.93
P34949	Mannose-6-phosphate isomerase OS=Ho	MPI	46.655	19.861	9.7	3	3	3	1.095	1.08	0.962	1.008	0.877	1.161	0.8	1.006	0.91	0.94	0.83
Q9H3H5	UDP-N-acetylglucosamine--dolichyl-pho	DPAGT1	46.089	6.4842	2	1	2	1	1.006	1.084	0.918	0.975	0.95	1.01	1.101	1.034	0.91	0.94	1.02
Q13438	Protein OS-9 OS=Homo sapiens OX=96	OS9	75.561	112.38	24	11	13	11	1.058	1.114	0.978	0.989	0.93	1.056	0.874	0.927	0.91	0.91	0.83
Q7Z2T5	TRMT1-like protein OS=Homo sapiens (	TRMT1L	81.746	54.215	11.7	7	8	7	0.945	1.21	0.94	1.011	0.89	1.069	1.004	1.022	0.91	0.91	0.94
Q9P2N5	RNA-binding protein 27 OS=Homo sapie	RBM27	118.72	59.667	8.4	9	11	9	1.05	1.031	0.929	0.955	1.033	1.094	0.957	1.001	0.91	1.02	0.94
P82914	28S ribosomal protein S15, mitochondria	MRPS15	29.842	14.281	8.6	2	3	2	1.055	1.019	0.962	0.915	1.107	1.155	0.92	0.945	0.91	1.09	0.90
Q14141	Septin-6 OS=Homo sapiens OX=9606 G	SEPT6	49.716	116.21	35.3	11	22	5	1.039	1.242	1.045	1.019	0.799	1.163	0.816	0.964	0.90	0.86	0.78
Q8NI36	WD repeat-containing protein 36 OS=Ho	WDR36	105.32	84.046	12.4	10	12	10	1.092	0.998	0.941	0.95	1.048	1.044	0.918	0.983	0.90	1.00	0.91
Q07866	Kinesin light chain 1 OS=Homo sapiens	KLC1	65.309	134.88	28.4	15	20	8	1.075	1.141	0.941	1.064	0.809	0.927	1.005	0.996	0.90	0.78	0.90
P82932	28S ribosomal protein S6, mitochondrial	MRPS6	14.226	33.817	32.8	4	4	4	1.044	1.008	0.928	0.928	1.153	1.155	0.882	0.949	0.90	1.12	0.89
P24723	Protein kinase C eta type OS=Homo sapi	PRKCH	77.828	18.991	4	3	3	3	1.087	1.003	0.945	0.945	1.049	1.09	0.925	0.991	0.90	1.02	0.92
P61586	Transforming protein RhoA OS=Homo sa	RHOA	21.768	43.214	33.2	5	10	2	1.088	1.054	0.972	0.965	0.936	1.129	0.854	1	0.90	0.96	0.87
Q9UBX3	Mitochondrial dicarboxylate carrier OS=	SLC25A10	31.282	27.398	14.3	3	3	3	1.006	1.041	0.871	0.98	0.956	1.359	0.802	1.067	0.90	1.13	0.91
A6NFI3	Zinc finger protein 316 OS=Homo sapier	ZNF316	108.44	12.805	1.8	2	2	2	1.099	1.037	1.014	0.917	1.03	1.211	0.731	1.016	0.90	1.05	0.82
Q96IZ0	PRKC apoptosis WT1 regulator protein (	PAWR	36.567	47.313	25.9	6	8	6	1.075	0.967	0.911	0.935	1.04	0.811	1.148	1.107	0.90	0.91	1.10
Q96Q89	Kinesin-like protein KIF20B OS=Homo s	KIF20B	210.63	24.273	2.1	4	4	4	1.09	1.093	0.976	0.997	0.92	1.053	0.852	1.019	0.90	0.90	0.86
P35251	Replication factor C subunit 1 OS=Homo	RFC1	128.25	111.3	11.8	10	9	9	1.027	0.916	0.851	0.905	1.086	1.024	1.094	1.059	0.90	1.09	1.11
Q92667	A-kinase anchor protein 1, mitochondrial	AKAP1	97.34	27.885	6.9	4	4	4	1.035	1.074	0.937	0.969	0.951	1.003	1.075	1.013	0.90	0.93	0.99
O96008	Mitochondrial import receptor subunit TO	TOMM40	37.893	113.02	21.6	5	12	5	1.06	1.08	0.957	0.977	1.012	1.217	0.87	0.941	0.90	1.04	0.85
Q9H6T0	Epithelial splicing regulatory protein 2 O	ESRP2	78.4	34.057	9.4	6	8	5	1.031	1.263	1.01	1.063	0.741	1.158	0.953	1.042	0.90	0.83	0.87
Q7Z6J0	E3 ubiquitin-protein ligase SH3RF1 OS=	SH3RF1	93.128	8.4661	1.4	1	1	1	0.881	1.379	1.069	0.973	0.884	0.813	1.285	0.888	0.90	0.75	0.96
Q12905	Interleukin enhancer-binding factor 2 OS	ILF2	43.062	135.14	30	9	13	9	1.083	0.98	0.913	0.951	1.003	1.281	0.81	1.06	0.90	1.11	0.91
P40692	DNA mismatch repair protein Mlh1 OS=	MLH1	84.6	63.29	13.1	10	10	9	1.002	1.042	0.88	0.966	0.984	1.122	0.98	1.097	0.90	1.03	1.02

Q9GZT3	SRA stem-loop-interacting RNA-binding	SLIRP	12.349	44.572	62.4	6	12	6	1.106	1.072	0.936	1.031	1.066	1.064	0.894	1.026	0.90	0.98	0.88
Q9UMY1	Nucleolar protein 7 OS=Homo sapiens O	NOL7	29.426	28.595	10.9	4	6	4	1.04	1.003	0.9	0.945	1.125	1.065	1.04	0.984	0.90	1.07	0.99
O43149	Zinc finger ZZ-type and EF-hand domain	ZZEF1	331.07	63.714	3.3	10	11	10	0.996	1.206	0.949	1.039	0.966	1.125	0.95	0.953	0.90	0.95	0.86
P49407	Beta-arrestin-1 OS=Homo sapiens OX=9	ARRB1	47.065	79.778	20.8	8	9	8	1.051	1.036	0.895	0.989	0.958	0.899	1.129	1.072	0.90	0.89	1.05
O75330	Hyaluronan mediated motility receptor O	HMMR	84.099	115.59	22.7	14	17	14	1.069	1.068	0.952	0.977	1.018	1.285	0.67	0.903	0.90	1.08	0.74
Q6IQ23	Pleckstrin homology domain-containing f	PLEKHA7	127.13	17.184	2.9	3	3	3	1.084	1.237	1.131	0.964	0.749	0.976	0.925	0.968	0.90	0.74	0.82
Q6ZXV5	Transmembrane and TPR repeat-containi	TMTC3	104.01	95.554	14.5	10	13	10	1.045	1.07	0.921	0.988	0.957	0.953	0.962	1.074	0.90	0.90	0.96
Q14289	Protein-tyrosine kinase 2-beta OS=Homo	PTK2B	115.87	26.889	3.8	4	4	4	1.078	1.094	0.961	0.999	0.95	0.916	1	1.007	0.90	0.86	0.92
Q9HAV4	Exportin-5 OS=Homo sapiens OX=9606	XPO5	136.31	95.226	11.8	13	21	13	1.061	1.057	0.92	0.991	0.957	1.029	0.944	1.005	0.90	0.94	0.92
P09496	Clathrin light chain A OS=Homo sapiens	CLTA	27.076	34.155	12.1	5	5	5	1.071	1.077	0.952	0.986	1.067	1.143	0.873	1.05	0.90	1.03	0.90
O95625	Zinc finger and BTB domain-containing	ZBTB11	119.38	19.431	2.8	3	3	2	1.098	0.987	0.955	0.926	1.074	1.185	0.875	0.94	0.90	1.08	0.87
P36954	DNA-directed RNA polymerase II subuni	POLR2I	14.523	12.302	19.2	2	2	2	0.883	1.408	0.994	1.072	0.794	1.072	0.743	1.156	0.90	0.81	0.83
Q92665	28S ribosomal protein S31, mitochondria	MRPS31	45.318	63.419	24.8	8	10	8	1.037	1.101	0.958	0.97	1.007	1.158	0.741	0.937	0.90	1.01	0.78
Q14687	Genetic suppressor element 1 OS=Homo	GSE1	136.16	65.993	8.5	9	13	9	1.049	1.16	0.987	1.005	0.963	1.279	0.729	1.007	0.90	1.01	0.79
P35232	Prohibitin OS=Homo sapiens OX=9606	PHB	29.804	94.714	45.6	11	16	11	1.043	1.094	0.972	0.955	1.035	1.107	0.861	0.984	0.90	1.00	0.86
P35544	Ubiquitin-like protein FUBI OS=Homo s	FAU	7.7598	8.6942	25.7	1	1	1	1.136	1.007	0.967	0.965	0.976	1.172	0.805	0.958	0.90	1.00	0.82
P62979	Ubiquitin-40S ribosomal protein S27a O	RPS27A	17.965	155.03	56.4	8	28	4	1.115	0.961	0.867	1.004	1.015	1.004	0.94	1.095	0.90	0.97	0.98
O43175	D-3-phosphoglycerate dehydrogenase OS	PHGDH	56.65	174.34	36.6	17	28	17	1.067	1.17	1.036	0.98	0.88	0.872	1.095	0.959	0.90	0.78	0.92
Q9NZJ9	Diphosphoinositol polyphosphate phospl	NUDT4	20.306	25.294	27.2	3	4	1	1.08	0.944	0.953	0.871	1.168	1.174	0.757	1.158	0.90	1.16	0.95
Q8N2K0	Monoacylglycerol lipase ABHD12 OS=H	ABHD12	45.096	43.598	15.6	6	8	6	1.013	1.1	0.92	0.984	0.955	0.934	1.042	1.11	0.90	0.89	1.02
Q9Y483	Metal-response element-binding transcrip	MTF2	67.089	14.861	4.6	2	2	2	1.041	0.879	0.787	0.943	1.186	1.133	0.946	1.16	0.90	1.21	1.10
Q92538	Golgi-specific brefeldin A-resistance gua	GBF1	206.44	199.34	16	22	28	22	1.049	1.027	0.932	0.938	1.033	0.916	1.061	1.035	0.90	0.94	1.01
Q15067	Peroxisomal acyl-coenzyme A oxidase 1	ACOX1	74.423	155.96	20.2	9	14	9	1.046	1.291	1.074	1.031	0.798	0.939	0.958	0.844	0.90	0.74	0.77
P51398	28S ribosomal protein S29, mitochondria	DAP3	45.566	49.554	16.8	6	8	6	1.082	1.062	1.007	0.924	1.034	1.114	0.897	0.889	0.90	1.00	0.83
Q9NRL3	Striatin-4 OS=Homo sapiens OX=9606 (	STRN4	80.595	18.999	3.1	2	2	2	1.012	1.209	0.974	1.026	0.85	1.053	0.974	0.938	0.90	0.86	0.86
Q9P021	Cysteine-rich PDZ-binding protein OS=H	CRIPT	11.216	13.213	18.8	2	2	2	1.028	1.002	0.872	0.956	0.973	1.285	0.797	1.175	0.90	1.11	0.97
O75909	Cyclin-K OS=Homo sapiens OX=9606 C	CCNK	64.239	14.923	4.1	2	2	2	1.099	0.981	0.946	0.927	1.062	1.314	0.714	1.001	0.90	1.14	0.82
Q49A26	Putative oxidoreductase GLYR1 OS=Ho	GLYR1	60.556	50.577	10.3	5	8	5	1.12	1.004	0.975	0.937	0.964	1.194	0.845	0.979	0.90	1.02	0.86
Q02252	Methylmalonate-semialdehyde dehydroge	ALDH6A1	57.839	173.34	27.7	9	12	9	1.109	1.043	0.94	0.997	1.015	1.058	0.899	0.956	0.90	0.96	0.86
P07339	Cathepsin D OS=Homo sapiens OX=960	CTSD	44.552	132.65	40.8	13	25	13	1.063	1.097	0.976	0.968	1.06	1.066	0.853	0.935	0.90	0.98	0.83
Q9UKF7	Cytoplasmic phosphatidylinositol transfe	PITPNC1	38.387	6.8548	2.4	1	1	1	1.067	1.04	0.973	0.923	1.053	1.132	0.923	0.957	0.90	1.04	0.89
P54198	Protein HIRA OS=Homo sapiens OX=96	HIRA	111.83	17.098	3.1	3	3	3	0.992	1.164	1.036	0.904	1.054	1.255	0.775	0.965	0.90	1.07	0.81
P22234	Multifunctional protein ADE2 OS=Homo	PAICS	47.079	135.88	37.4	14	19	14	1.06	1.113	0.983	0.972	0.929	1.008	0.971	1.009	0.90	0.89	0.91
Q99747	Gamma-soluble NSF attachment protein	NAPG	34.746	63.993	26.6	9	11	9	1.073	1.05	0.953	0.957	0.96	0.929	0.999	0.986	0.90	0.89	0.93
Q9NX20	39S ribosomal protein L16, mitochondria	MRPL16	28.449	16.936	10.4	2	3	2	0.976	1.026	0.872	0.929	1.16	1.11	1.077	1	0.90	1.13	1.04
Q969S9	Ribosome-releasing factor 2, mitochondri	GFM2	86.6	23.316	4.5	3	3	3	1.078	1.053	0.949	0.968	0.924	0.98	1.05	1.026	0.90	0.89	0.97
Q8WV22	Non-structural maintenance of chromoso	NSMCE1	30.855	20.843	10.2	3	3	3	0.998	1.089	0.979	0.898	1.062	1.203	0.913	0.999	0.90	1.09	0.92
Q99848	Probable rRNA-processing protein EBP2	EBNA1BP	34.852	52.66	26.8	7	12	7	1.059	1.016	0.897	0.969	0.993	0.952	1.032	1.069	0.90	0.94	1.01
P52630	Signal transducer and activator of transcr	STAT2	97.915	63.191	7.1	5	6	5	1.091	1.061	0.92	1.015	0.939	1.024	0.971	1.023	0.90	0.91	0.93
P30260	Cell division cycle protein 27 homolog O	CDC27	91.866	46.817	9.5	6	6	6	1.01	1.052	0.881	0.973	1.067	1.294	0.711	1.042	0.90	1.15	0.85
Q7Z7F7	39S ribosomal protein L55, mitochondria	MRPL55	15.128	17.367	24.2	2	3	2	0.979	1.023	0.85	0.95	1.242	1.274	0.871	1.037	0.90	1.26	0.95
P52597	Heterogeneous nuclear ribonucleoprotein	HNRNPF	45.671	207.9	42.7	13	40	11	1.035	1.095	0.945	0.97	0.974	0.964	1.065	1.006	0.90	0.91	0.97
O00303	Eukaryotic translation initiation factor 3	EIF3F	37.563	108.26	30.5	9	9	8	0.998	1.239	1.011	1	0.922	1.267	0.728	0.951	0.90	0.98	0.75
P22392	Nucleoside diphosphate kinase B OS=Ho	NME2	17.298	20.694	50	9	25	3	0.97	1.494	1.238	0.977	0.801	1.278	0.745	0.651	0.90	0.84	0.57
Q7L5N7	Lysophosphatidylcholine acyltransferase	LPCAT2	60.207	35.468	10.5	5	6	5	1.028	1.168	0.969	1.005	0.882	1.079	0.887	1.028	0.90	0.89	0.87
P06493	Cyclin-dependent kinase 1 OS=Homo sa	CDK1	34.095	86.391	36.7	9	23	7	1.107	1.106	0.993	0.996	0.937	0.95	1.007	0.933	0.90	0.85	0.88
Q6WKZ4	Rab11 family-interacting protein 1 OS=H	RAB11FIP	137.17	121.88	13.6	15	19	13	1.027	1.146	0.965	0.988	0.938	1.072	0.909	0.942	0.90	0.92	0.85
Q9BZ29	Dedicator of cytokinesis protein 9 OS=H	DOCK9	236.44	39.639	2.5	5	5	5	1.071	1.082	0.968	0.967	0.914	0.83	0.998	0.979	0.90	0.81	0.92
Q14160	Protein scribble homolog OS=Homo sapi	SCRIB	174.88	122.83	12.6	17	19	13	1.019	1.041	0.79	1.061	0.936	0.961	0.977	1.156	0.90	0.92	1.04
O96005	Cleft lip and palate transmembrane protei	CLPTM1	76.096	104.03	21.1	11	16	10	1.119	0.999	0.934	0.969	1.023	1.049	0.752	0.99	0.90	0.98	0.82
Q9H9Y6	DNA-directed RNA polymerase I subunit	POLR1B	128.23	67.909	8	8	11	8	1.033	0.935	0.852	0.916	1.162	0.989	1.035	0.989	0.90	1.09	1.03
Q99623	Prohibitin-2 OS=Homo sapiens OX=960	PHB2	33.296	67.181	33.1	9	15	9	1.042	1.103	0.981	0.946	1.025	1.1	0.89	0.947	0.90	0.99	0.86
Q9UBB4	Ataxin-10 OS=Homo sapiens OX=9606	ATXN10	53.488	152.29	30.1	12	18	12	1.093	1.129	0.973	1.023	0.958	0.993	0.949	0.893	0.90	0.88	0.83
Q00536	Cyclin-dependent kinase 16 OS=Homo s	CDK16	55.715	7.3187	3.8	2	2	1	1.076	1.083	0.956	0.983	1.005	0.839	1.113	0.957	0.90	0.85	0.96
A0AV96	RNA-binding protein 47 OS=Homo sapi	RBM47	64.098	94.203	21.6	10	14	9	1.059	1.031	0.894	0.983	1.003	0.951	1.113	1.01	0.90	0.93	1.02
O95786	Probable ATP-dependent RNA helicase I	DDX58	106.6	41.631	6.7	6	7	6	1.094	1.143	0.965	1.044	0.821	0.789	1.123	0.977	0.90	0.72	0.94
P53007	Tricarboxylate transport protein, mitocho	SLC25A1	34.012	45.117	17.7	5	7	5	1.041	1.028	0.893	0.965	1.039	1.09	0.939	1.012	0.90	1.03	0.94
P19367	Hexokinase-1 OS=Homo sapiens OX=96	HK1	102.48	137.75	21.5	17	22	16	1.035	1.082	0.933	0.968	0.961	1.003	0.991	1.039	0.90	0.93	0.96
Q9Y5J6	Mitochondrial import inner membrane tra	TIMM10B	11.586	17.09	24.3	2	2	2	1.008	1.049	0.913	0.934	1.116	0.904	1.143	1.033	0.90	0.98	1.06
Q7Z2E3	Aprataxin OS=Homo sapiens OX=9606	APTX	40.74	30.765	20.2	5	5	5	0.98	1.075	0.93	0.915	1.093	1.086	1.02	0.957	0.90	1.06	0.96
Q13310	Polyadenylate-binding protein 4 OS=Ho	PABPC4	70.782	75.663	33.2	19	35	10	1.06	1.08	0.967	0.954	0.988	1.046	0.915	0.964	0.90	0.95	0.88
O95471	Claudin-7 OS=Homo sapiens OX=9606	CLDN7	22.418	8.8389	8.1	1	1	1	1.042	1.135	0.998	0.956	0.942	1.289	0.737	0.973	0.90	1.02	0.79
P37837	Transaldolase OS=Homo sapiens OX=96	TALDO1	37.54	122.98	40.1	15	36	14	1.035	1.062	0.923	0.959	0.974	1.064	0.971	1.018	0.90	0.97	0.95
P42574	Caspase-3 OS=Homo sapiens OX=9606	CASP3	31.608	6.6799	4	1	1	1	0.934	1.307	1.099	0.912	0.748	1.46	0.779	0.956	0.90	0.99	0.77
Q2VPB7	AP-5 complex subunit beta-1 OS=Homo	AP5B1	93.948	21.202	5.5	3	3	3	1	1.161	1.001	0.938	0.982	0.875	1.148	1.004	0.90	0.86	1.00
Q8IYT4	Katanin p60 ATPase-containing subunit	KATNAL2	61.252	11.15	2.2	1	2	1	1.092	1.059	1.016	0.914	0.961	0.971	1.025	1.02	0.90	0.90	0.95
P42338	Phosphatidylinositol 4,5-bisphosphate 3-	PIK3CB	122.76	18.455	2.6	3	3	3	1.114	1.017	0.928	0.984	1.038	0.778	1.115	1.006	0.90	0.85	1.00
Q96GC9	Vacuole membrane protein 1 OS=Homo	VMP1	46.237	12.136	3.9	1	1	1	0.924	1.443	1.145	0.978	0.64	1.375	0.785	0.863	0.90	0.85	0.70
O60869	Endothelial differentiation-related factor	EDF1	16.368	49.721	35.1	6	18	6	1.094	1.019	0.947	0.948	1.022	1.095	0.885	1.009	0.90	1.00	0.90
P13073	Cytochrome c oxidase subunit 4 isoform	COX4I1	19.576	50.79	32.5	7	15	6	1.07	1.06	0.981	0.929	0.99	1.171	0.86	0.911	0.90	1.01	0.83
Q7L2Z9	Centromere protein Q OS=Homo sapiens	CENPQ	30.595	14.033	9	2	2	2	0.872	0.773	0.626	0.849	1.29	1.162	1.239	1.475	0.90	1.49	1.65
Q9Y2X3	Nucleolar protein 58 OS=Homo sapiens	NOP58	59.578	102.24	32.3	13	19	13	1.006	1.025	0.879	0.942	1.052	1.023	1.036	1.096	0.90	1.02	1.05
P10619	Lysosomal protective protein OS=Homo	CTSA	54.465	79.066	13.8	6	8	6	1.104	1.067	0.991	0.955	0.98	0.998	0.875	0.857	0.90	0.91	0.80
P60953	Cell division control protein 42 homolog	CDC42	21.258	36.431	25.7	4	11	4	1.099	1.091	1.004	0.959	0.914	0.983	1.007	1.066	0.90	0.87	0.95
P54727	UV excision repair protein RAD23 homo	RAD23B	43.171	77.766	28.6	12	21	9	1.064	1.261	1.051	1.033	0.882	1.007	0.901	0.925	0.90	0.81	0.79
P57105	Synaptojanin-2-binding protein OS=Hom	SYNJ2BP	15.928	16.08	24.1	3	3	2	1.084	1.034	0.949	0.949	0.965	0.962	1.067	1.026	0.90	0.91	0.99
Q96EY1	DnaJ homolog subfamily A member 3, mi	DNAJA3	52.488	96.027	11.9	4	5	4	1.009	1.041	0.894	0.943	0.989	1.152	0.919	1.074	0.90	1.04	0.97
Q14728	Major facilitator superfamily domain-con	MFSD10	48.339	12.774	4.8	2	2	2	1.011	1.337	1.136	0.968	0.757	0.769	1.188	0.913	0.90	0.65	0.89
P06753	Tropomyosin alpha-3 chain OS=Homo sa	TPM3	32.95	95.968	35.1	13	21	6	1.106	1.065	0.96	0.985	0.92	0.9	1.116	1.078	0.90	0.84	1.01
Q96RP9	Elongation factor G, mitochondrial OS=H	GFM1	83.471	122.64	20	11	16	11	1.067	1.036	0.937	0.947	0.958	0.987	1.011	1.023	0.90	0.92	0.97
Q9H0B6	Kinesin light chain 2 OS=Homo sapiens	KLC2	68.934	33.979	17.4	10	12	5	1.094	1.087	1.054	0.899	1.027	0.909	1.08	0.91	0.90	0.89	0.91
P16220	Cyclic AMP-responsive element-binding	CREB1	36.688	22.855	15.2	3	4	2	1.055	0.971	0.766	1.048	1.035	1.165	0.845	1.105	0.90	1.09	0.96
O15231	Zinc finger protein 185 OS=Homo sapien	ZNF185	73.525	33.389	7.8	5	5	5	0.995	1.001	0.879	0.908	0.972	1.046	1.06	1.179	0.90	1.01	1.12
Q9P289	Serine/threonine-protein kinase 26 OS=H	STK26	46.528	87.894	28.6	12	16	6	1.173	1.004	0.922	1.027	0.943	0.873	1.033	1.028	0.90	0.83	0.95
Q9UN86	Ras GTPase-activating protein-binding p	G3BP2	54.12	35.84	13.5	6	11	5	1.066	1.053	0.905	0.992	1.024	0.932	1.018	1.076	0.90	0.92	0.99
P40926	Malate dehydrogenase, mitochondrial OS	MDH2	35.503	236.65	56.2	16	56	16	1.057	1.061	0.936	0.96	0.982	1.183	0.842	0.982	0.90	1.02	0.86
P61619	Protein transport protein Sec61 subunit a	SEC61A1	52.264	36.957	10.5	5	9	5	1.049	1.039	0.909	0.96	0.965	1.212	0.908	1.016	0.90	1.04	0.92
Q99519	Sialidase-1 OS=Homo sapiens OX=9606	NEU1	45.467	13.943	5.8	2	3	2	0.983	1.084	0.953	0.897	1.126	1.121	0.935	1.03	0.90	1.09	0.95
Q02750	Dual specificity mitogen-activated protei	MAP2K1	43.439	33.087	18.8	7	12	3	1.016	1.145	0.974	0.96	0.931	0.907	1.097	0.959	0.89	0.85	0.95
Q9BXW9	Fanconi anemia group D2 protein OS=H	FANCD2	164.13	85.918	9.3	12	14	12	0.993	0.92	0.792	0.92	1.165	1.064	1.102	1.171	0.89	1.17	1.19
Q8TF05	Serine/threonine-protein phosphatase 4 r	PPP4R1	107	23.613	3.9	3	3	3	1.058	1.038	0.888	0.987	0.911	1.067	0.996	1.087	0.89	0.94	0.99
Q8TC12	Retinol dehydrogenase 11 OS=Homo sap	RDH11	35.386	45.303	10.7	3	4	3	1.092	1.063	0.919	1.008	0.948	0.898	1.059	0.89	0.89	0.86	0.90
Q9Y421	Protein FAM32A OS=Homo sapiens OX	FAM32A	13.178	11.951	16.1	2	2	2	1.071	1.062	0.946	0.96	1.012	1.252	0.754	0.986	0.89	1.06	0.82
P16278	Beta-galactosidase OS=Homo sapiens O	GLB1	76.074	50.668	9.9	5	7	5	1.114	1.111	1.001	0.987	0.964	0.991	1.014	0.857	0.89	0.88	0.84
Q96RE7	Nucleus accumbens-associated protein 1	NACC1	57.258	17.944	5.3	2	2	2	1.14	0.774	0.703	1.007	1.257	0.786	1.023	1.255	0.89	1.07	1.19
Q9NX24	H/ACA ribonucleoprotein complex subu	NHP2	17.201	38.394	31.4	3	5	3	1.012	0.909	0.825	0.891	1.025	0.917	1.083	1.035	0.89	1.01	1.10
Q7Z406	Myosin-14 OS=Homo sapiens OX=9606	MYH14	227.87	17.672	4.2	8	23	2	1.076	1.284	1.099	1.009	0.672	0.912	1.073	0.99	0.89	0.67	0.87
Q9BSY9	Deubiquitinase DESI2 OS=Homo sapien	DESI2	21.444	11.078	8.8	1	1	1	1.017	1.154	0.912	1.027	0.999	0.965	0.908	1.052	0.89	0.90	0.90
Q96HR3	Mediator of RNA polymerase II transcrip	MED30	20.277	14.9	15.2	2	2	2	1.048	1.074	0.943	0.952	1.023	0.885	1.092	1.042	0.89	0.90	1.01
P30519	Heme oxygenase 2 OS=Homo sapiens O	HMOX2	36.032	61.291	30.1	8	9	8	1.044	1.105	0.951	0.968	0.996	1.092	0.954	0.977	0.89	0.97	0.90
Q92979	Ribosomal RNA small subunit methyltra	EMG1	26.72	52.905	38.5	7	9	7	1.055	0.959	0.865	0.933	1.038	1.045	0.999	1.147	0.89	1.03	1.07
Q9P2M7	Cingulin OS=Homo sapiens OX=9606 G	CGN	136.38	179.4	13.9	13	13	13	1.07	1.165	1.002	0.993	0.853	0.81	1.097	1.092	0.89	0.74	0.98
Q3B726	DNA-directed RNA polymerase I subunit	TWISTNB	37.432	21.029	11.8	3	3	3	1.098	1.098	0.901	1.059	0.984	0.926	0.902	0.973	0.89	0.87	0.85
O95817	BAG family molecular chaperone regulat	BAG3	61.594	168.92	31.5	11	14	11	1.076	1.045	0.97	0.923	0.946	0.945	0.997	1.075	0.89	0.89	0.98
Q14781	Chromobox protein homolog 2 OS=Hom	CBX2	56.08	12.848	3.9	2	2	2	1.045	1.062	0.914	0.966	0.999	0.927	1.147	0.988	0.89	0.91	1.01
Q86WJ1	Chromodomain-helicase-DNA-binding p	CHD1L	100.98	80.628	11.1	8	8	8	1.046	1.07	0.902	0.986	0.99	0.921	1.134	0.955	0.89	0.90	0.99
O43166	Signal-induced proliferation-associated 1	SIPA1L1	200.03	50.632	4.7	7	8	7	1.04	1.084	0.96	0.935	0.912	0.926	1.054	1.035	0.89	0.87	0.98
Q9BSV6	tRNA-splicing endonuclease subunit Sen	TSEN34	33.652	47.737	19.4	4	6	4	0.939	1.031	0.87	0.887	1.056	1.113	1.116	1.204	0.89	1.10	1.18
Q00403	Transcription initiation factor IIB OS=H	GTF2B	34.833	50.445	22.2	5	7	5	1.125	0.983	0.918	0.962	0.995	0.986	0.931	0.982	0.89	0.94	0.91
Q8IXB1	DnaJ homolog subfamily C member 10 O	DNAJC10	91.079	38.209	7.8	6	6	6	1.11	0.728	0.657	0.982	1.183	0.772	1.206	1.354	0.89	1.06	1.39
P10515	Dihydrolipoyllysine-residue acetyltransfe	DLAT	68.996	100	20.4	13	21	13	1.051	1.058	0.946	0.934	1.015	1.049	0.938	0.982	0.89	0.98	0.91
Q8IUH3	RNA-binding protein 45 OS=Homo sapi	RBM45	53.502	16.446	5.3	2	2	2	1.134	0.99	0.913	0.98	0.937	0.964	0.979	1.081	0.89	0.90	0.97
Q8N983	39S ribosomal protein L43, mitochondria	MRPL43	23.431	19.52	10.2	2	4	2	1.031	1.157	0.973	0.977	1	1.251	0.781	0.887	0.89	1.03	0.76
L0R6Q1	SLC35A4 upstream open reading frame p	SLC35A4	11.133	40.761	32	2	2	2	1.116	1.067	1.031	0.914	0.989	0.971	0.986	0.963	0.89	0.90	0.89
Q9UJW0	Dynactin subunit 4 OS=Homo sapiens O	DCTN4	52.337	99.832	25.9	9	11	9	1.048	1.167	0.986	0.987	0.922	1.06	0.895	1.01	0.89	0.89	0.86
Q9UJM3	ERBB receptor feedback inhibitor 1 OS=	ERRFI1	50.559	12.499	4.3	2	2	2	1.022	1.063	0.893	0.964	1.037	1.26	0.755	1.09	0.89	1.10	0.88
O75319	RNA/RNP complex-1-interacting phosph	DUSP11	43.71	29.335	11.1	3	3	3	0.96	0.988	0.864	0.87	1.087	1.171	1.073	1.185	0.89	1.16	1.16
Q8IUI8	Cytokine receptor-like factor 3 OS=Hom	CRLF3	49.765	37.736	10	4	4	4	1.054	0.939	0.781	0.993	1.091	0.866	1.191	1.167	0.89	0.98	1.18
Q06481	Amyloid-like protein 2 OS=Homo sapien	APLP2	86.955	32.742	7.6	4	5	4	1.061	1.05	0.961	0.918	1.103	0.809	1.143	1.028	0.89	0.91	1.03
Q96L92	Sorting nexin-27 OS=Homo sapiens OX=	SNX27	61.264	40.172	12.4	6	8	6	1.064	1.065	0.862	1.033	0.907	0.894	1.037	1.062	0.89	0.85	0.99
P35052	Glypican-1 OS=Homo sapiens OX=9606	GPC1	61.68	13.614	4.3	2	2	2	0.925	1.211	1.048	0.853	1.118	0.915	1.093	1.071	0.89	0.95	1.01
Q08722	Leukocyte surface antigen CD47 OS=Ho	CD47	35.213	21.971	8.7	3	6	3	1.029	1.063	0.923	0.938	1.045	0.831	1.114	1.031	0.89	0.90	1.03
P63092	Guanine nucleotide-binding protein G(s)	GNAS	45.664	39.39	20.6	7	14	6	1.079	1.084	0.955	0.969	0.999	1.181	0.833	0.922	0.89	1.01	0.81
Q96NC0	Zinc finger matrin-type protein 2 OS=Ho	ZMAT2	23.612	42.94	29.1	5	5	5	1.037	1.016	0.896	0.93	0.971	0.904	1.131	1.111	0.89	0.91	1.09
P40925	Malate dehydrogenase, cytoplasmic OS=	MDH1	36.426	147.83	30.5	11	22	10	1.059	1.118	0.97	0.966	0.898	0.957	1.005	0.955	0.89	0.85	0.90
O60701	UDP-glucose 6-dehydrogenase OS=Hom	UGDH	55.023	161.1	33.6	13	15	13	1.092	1.081	0.976	0.956	0.956	0.979	0.958	1.001	0.89	0.89	0.90
O75503	Ceroid-lipofuscinosis neuronal protein 5	CLN5	41.496	28.436	10.6	4	5	4	1.047	1.098	0.959	0.948	1.045	1.241	0.831	0.91	0.89	1.07	0.81
P62330	ADP-ribosylation factor 6 OS=Homo sap	ARF6	20.082	58.561	32.6	4	5	4	1.091	1.133	0.996	0.981	0.957	0.939	0.943	0.923	0.89	0.85	0.84
Q15084	Protein disulfide-isomerase A6 OS=Hom	PDIA6	48.121	174.8	44.1	14	28	14	1.074	1.059	0.95	0.946	0.989	0.876	1.106	0.972	0.89	0.87	0.97
Q15041	ADP-ribosylation factor-like protein 6-in	ARL6IP1	23.362	13.398	8.9	2	4	2	1.02	1.436	1.06	1.123	0.647	1.177	0.67	0.83	0.89	0.74	0.61
Q8IYL3	UPF0688 protein C1orf174 OS=Homo sa	C1orf174	25.977	20.889	13.2	2	2	2	0.781	1.782	1.358	0.92	0.614	1.797	0.495	0.562	0.89	0.94	0.41
P07948	Tyrosine-protein kinase Lyn OS=Homo s	LYN	58.573	7.5932	3.7	2	3	1	0.995	1.016	0.825	0.962	1.024	1.062	1.034	1.183	0.89	1.04	1.10
Q9Y5P4	Collagen type IV alpha-3-binding protein	COL4A3BI	70.834	84.391	16.2	8	7	7	1.131	1.067	0.954	0.999	0.908	0.779	1.119	0.93	0.89	0.77	0.93
O96006	Zinc finger BED domain-containing prot	ZBED1	78.155	45.813	11.5	7	8	7	0.963	0.91	0.751	0.913	1.15	1.183	0.929	1.243	0.89	1.25	1.16
P52434	DNA-directed RNA polymerases I, II, and	POLR2H	17.143	22.659	24	3	4	3	1.113	1.181	1.01	1.028	0.827	1.07	0.906	0.818	0.89	0.83	0.75
P51617	Interleukin-1 receptor-associated kinase 1	IRAK1	76.536	7.5168	1.7	1	2	1	1.046	1.337	1.109	1.008	0.716	1.198	0.682	0.94	0.89	0.80	0.68
Q8NCD3	Holliday junction recognition protein OS	HJURP	83.538	12.992	3.9	2	2	2	1.07	1.122	0.998	0.949	0.959	1.064	0.954	0.93	0.89	0.92	0.86
P49642	DNA primase small subunit OS=Homo sa	PRIM1	49.901	36.615	11.4	5	7	5	0.97	0.855	0.703	0.918	1.171	1.058	1.104	1.322	0.89	1.22	1.33
Q9UJS0	Calcium-binding mitochondrial carrier pr	SLC25A13	74.175	236.19	38.1	23	29	23	1.059	1.043	0.902	0.965	1.008	1.04	0.951	1.046	0.89	0.97	0.95
Q86UY6	N-alpha-acetyltransferase 40 OS=Homo	NAA40	27.194	15.151	8.9	2	5	2	1.074	1.035	0.948	0.925	1.009	1.039	0.967	1.037	0.89	0.97	0.95
O43795	Unconventional myosin-Ib OS=Homo sap	MYO1B	131.98	140.07	17.5	19	25	19	1.031	1.005	0.859	0.949	1.02	0.979	1.094	1.117	0.89	0.98	1.09
Q9UBC2	Epidermal growth factor receptor substrat	EPS15L1	94.254	170.48	25.1	17	23	17	1.081	1.044	0.935	0.952	1.002	0.961	1.086	0.953	0.89	0.92	0.96
Q9ULC3	Ras-related protein Rab-23 OS=Homo sa	RAB23	26.659	48.127	21.1	3	3	3	1.114	1.252	1.121	0.98	0.859	1.022	0.811	0.834	0.89	0.80	0.70
P23284	Peptidyl-prolyl cis-trans isomerase B OS	PPIB	23.742	56.169	31.9	6	14	6	1.08	1.06	0.948	0.952	0.934	1.006	0.975	1.026	0.89	0.91	0.94
Q9H160	Inhibitor of growth protein 2 OS=Homo	ING2	32.808	7.0702	4.3	1	1	1	0.959	1	0.689	1.05	0.998	1.187	0.868	1.323	0.89	1.12	1.12
Q9GZM5	Protein YIPF3 OS=Homo sapiens OX=9	YIPF3	38.247	7.9866	2.6	1	2	1	1.007	1.182	0.979	0.964	0.9	1.049	1.006	1	0.89	0.89	0.92
Q8IWA4	Mitofusin-1 OS=Homo sapiens OX=960	MFN1	84.159	41.22	9.3	6	7	6	1.04	0.95	0.882	0.884	1.004	0.96	1.304	1.093	0.89	0.99	1.20
Q9UIV1	CCR4-NOT transcription complex subun	CNOT7	32.745	21.312	11.9	3	4	3	1.048	1.093	0.908	0.992	1.006	0.934	1.072	0.976	0.89	0.91	0.96
P62308	Small nuclear ribonucleoprotein G OS=H	SNRPG	8.496	13.472	26.3	2	3	2	1.114	1.009	0.939	0.945	1.034	0.928	0.948	1.104	0.89	0.92	0.97
P51148	Ras-related protein Rab-5C OS=Homo sa	RAB5C	23.482	97.976	54.2	9	11	6	1.13	1.035	0.967	0.954	0.985	1.042	0.996	0.833	0.89	0.94	0.84
Q96DV4	39S ribosomal protein L38, mitochondria	MRPL38	44.596	58.919	29.7	8	9	8	1.003	1.248	1.09	0.907	0.851	1.159	0.863	1	0.89	0.89	0.83
Q96SZ6	CDK5 regulatory subunit-associated prot	CDK5RAP	67.688	31.145	9.2	4	4	4	0.967	0.982	0.765	0.964	1.083	1.073	0.989	1.305	0.89	1.11	1.18
Q9UNE7	E3 ubiquitin-protein ligase CHIP OS=Ho	STUB1	34.856	45.542	20.5	6	11	6	1.063	1.062	0.949	0.936	1.008	1.29	0.757	1.031	0.89	1.08	0.84
Q9NV70	Exocyst complex component 1 OS=Hom	EXOC1	101.98	38.358	5.6	5	6	5	1.016	1.25	1.09	0.92	1.003	0.808	1.263	0.992	0.89	0.80	1.00
P63272	Transcription elongation factor SPT4 OS	SUPT4H1	13.193	7.6836	7.7	1	3	1	1.111	1.012	0.999	0.884	1.09	0.966	1.111	0.88	0.89	0.97	0.94
O75367	Core histone macro-H2A.1 OS=Homo sa	H2AFY	39.617	174.72	34.7	11	19	11	1.031	0.976	0.877	0.903	1.07	0.937	1.101	0.998	0.89	1.00	1.05

Q8TBM8	DnaJ homolog subfamily B member 14 O	DNAJB14	42.515	17.794	7.7	3	3	3	1.047	0.88	0.649	1.06	1.045	0.923	1.052	1.333	0.89	1.02	1.24
P10606	Cytochrome c oxidase subunit 5B, mitocl	COX5B	13.696	25.075	24	3	9	3	1.039	1.064	0.913	0.952	1.006	1.17	0.874	1.029	0.89	1.03	0.90
O75369	Filamin-B OS=Homo sapiens OX=9606	FLNB	278.16	323.31	57	108	203	101	1.035	1.156	0.959	0.984	0.887	0.921	1.099	1.049	0.89	0.83	0.98
O95619	YEATS domain-containing protein 4 OS	YEATS4	26.499	14.898	7.9	2	2	2	1.092	0.824	0.683	1.016	1.179	0.937	0.938	1.316	0.89	1.10	1.18
Q14257	Reticulocalbin-2 OS=Homo sapiens OX=	RCN2	36.876	66.581	31.5	9	14	9	1.072	1.107	0.968	0.964	0.941	1.262	0.757	0.982	0.89	1.01	0.80
Q5ST30	Valine--tRNA ligase, mitochondrial OS=	VARS2	118.49	21.923	3.5	2	2	2	1.014	1.12	0.956	0.936	0.933	0.996	1.109	1.04	0.89	0.90	1.01
Q9BRJ7	Tudor-interacting repair regulator protein	NUDT16L1	23.338	33.978	25.6	5	6	4	1.018	0.99	0.872	0.908	0.952	0.997	0.942	1.031	0.89	0.97	0.98
Q96DB5	Regulator of microtubule dynamics prote	RMDN1	35.808	28.901	14.6	4	6	4	1.036	1.13	0.976	0.944	0.983	1.098	0.913	1.05	0.89	0.96	0.91
Q9NVT9	Armadillo repeat-containing protein 1 OS	ARMC1	31.28	14.485	9.2	2	2	2	1.059	1.113	1.049	0.876	0.981	0.958	1.153	0.917	0.89	0.89	0.95
Q9Y6Y8	SEC23-interacting protein OS=Homo sap	SEC23IP	111.08	55.667	7.5	7	9	6	1.076	1.155	0.971	1.006	0.873	0.923	1.078	0.916	0.89	0.81	0.89
P49821	NADH dehydrogenase [ubiquinone] flavo	NDUFV1	50.817	87.454	25	12	16	12	1.071	1.107	0.942	0.988	0.953	1.169	0.898	0.916	0.89	0.97	0.83
Q92552	28S ribosomal protein S27, mitochondria	MRPS27	47.611	51.783	20.3	7	9	7	1.082	1.121	1.015	0.937	0.977	1.177	0.871	0.972	0.89	0.98	0.84
Q5BKY9	Protein FAM133B OS=Homo sapiens OX	FAM133B	28.385	18.855	14.6	3	3	3	1.059	1.046	0.894	0.971	1.004	1.045	1.007	1.01	0.89	0.97	0.96
P61009	Signal peptidase complex subunit 3 OS=	SPCS3	20.313	32.663	17.8	3	12	3	1.095	1.062	0.935	0.976	1.017	0.937	0.968	0.974	0.89	0.91	0.90
Q9Y5W7	Sorting nexin-14 OS=Homo sapiens OX=	SNX14	110.18	13.012	2.6	2	2	2	0.95	1.101	0.843	0.974	1.015	1.027	0.962	1.264	0.89	1.00	1.09
Q96JM3	Chromosome alignment-maintaining phos	CHAMP1	89.098	95.251	13.9	10	12	10	0.939	0.927	0.693	0.96	1.083	0.974	1.202	1.261	0.89	1.10	1.32
Q9NWT8	Aurora kinase A-interacting protein OS=	AURKAIP1	22.354	6.8052	5	1	1	1	0.98	1.105	0.815	1.032	1.023	1.033	1.007	1.062	0.89	0.99	0.99
Q13617	Cullin-2 OS=Homo sapiens OX=9606 G	CUL2	86.982	97.512	20.4	13	22	13	1.072	1.036	0.925	0.942	0.994	0.999	1.007	1.021	0.89	0.95	0.96
Q68CZ6	HAUS augmin-like complex subunit 3 OS	HAUS3	69.65	23.604	6.1	3	3	3	1.006	1.1	0.908	0.957	0.934	1.031	1.058	1.102	0.89	0.93	1.03
Q9BZE1	39S ribosomal protein L37, mitochondria	MRPL37	48.117	110.9	31	9	15	9	1.078	1.073	0.954	0.95	0.998	1.091	0.965	0.97	0.89	0.97	0.90
Q9NXU5	ADP-ribosylation factor-like protein 15 (	ARL15	22.876	25.107	19.1	3	4	3	1.043	1.012	0.92	0.899	1.083	0.963	1.092	1.063	0.89	1.00	1.05
Q712K3	Ubiquitin-conjugating enzyme E2 R2 OS	UBE2R2	27.166	38.409	14.7	3	4	3	1.109	1.1	0.944	1.011	0.9	1.167	0.75	0.989	0.89	0.94	0.79
Q9UBM7	7-dehydrocholesterol reductase OS=Hom	DHCR7	54.489	36.861	9.1	3	4	3	1.082	1.056	0.978	0.914	1.037	1.303	0.767	0.917	0.88	1.09	0.79
Q01974	Tyrosine-protein kinase transmembrane r	ROR2	104.76	24.495	4.7	3	3	3	0.939	0.763	0.564	0.942	1.097	0.757	1.427	1.682	0.88	1.09	1.83
P51572	B-cell receptor-associated protein 31 OS	BCAP31	27.991	92.541	33.3	10	14	9	1.093	1.077	0.963	0.957	0.924	0.843	1.036	1.006	0.88	0.81	0.94
Q9NPA0	ER membrane protein complex subunit 7	EMC7	26.47	26.263	16.1	3	4	3	1.075	1.19	0.969	1.035	0.828	1.067	0.903	0.955	0.88	0.84	0.82
P11511	Aromatase OS=Homo sapiens OX=9606	CYP19A1	57.882	68.162	17.7	8	11	8	1.018	1.29	1.031	1.011	0.745	0.889	1.026	0.992	0.88	0.71	0.87
P02794	Ferritin heavy chain OS=Homo sapiens O	FTH1	21.225	113.3	52.5	7	10	7	1.043	1.166	0.996	0.958	0.88	0.888	1.029	0.957	0.88	0.80	0.90
P62310	U6 snRNA-associated Sm-like protein LS	LSM3	11.845	10.188	11.8	1	2	1	1.133	0.998	0.973	0.912	1.07	0.877	1.078	0.978	0.88	0.91	0.96
P16435	NADPH--cytochrome P450 reductase OS	POR	76.689	139.08	24.4	12	17	12	1.073	1.049	0.971	0.906	0.963	1.274	0.83	1.064	0.88	1.05	0.89
O75431	Metaxin-2 OS=Homo sapiens OX=9606	MTX2	29.763	26.672	18.6	4	4	4	0.958	1.119	0.897	0.94	0.993	0.991	1.005	1.057	0.88	0.96	0.99
Q96GF1	E3 ubiquitin-protein ligase RNF185 OS=	RNF185	20.459	6.754	4.7	1	1	1	0.919	1.397	1.044	1.004	0.716	0.899	1.1	1.056	0.88	0.70	0.93
P83916	Chromobox protein homolog 1 OS=Hom	CBX1	21.418	25.388	30.3	4	13	3	1.108	0.955	0.887	0.937	1.008	0.967	0.995	1.075	0.88	0.96	1.00
Q8NC60	Nitric oxide-associated protein 1 OS=Ho	NOA1	78.457	47.466	11.6	6	8	6	1.127	1.074	1.068	0.878	1	1.106	0.703	0.935	0.88	0.96	0.74
O76003	Glutaredoxin-3 OS=Homo sapiens OX=9	GLRX3	37.432	99.738	39.1	11	22	11	1.051	1.141	0.959	0.979	0.942	1.031	0.944	1.013	0.88	0.90	0.89
Q9Y4D1	Disheveled-associated activator of morph	DAAM1	123.47	27.524	3.7	4	4	4	1.062	1.048	0.966	0.899	1.098	0.965	1.009	1.052	0.88	0.98	0.98
O00217	NADH dehydrogenase [ubiquinone] iron-	NDUFS8	23.705	31.505	21.9	4	6	4	1.064	1.043	0.964	0.898	0.889	1.02	0.919	1.119	0.88	0.91	0.97
Q9Y277	Voltage-dependent anion-selective chann	VDAC3	30.658	78.003	35.7	7	12	7	1.024	1.101	0.933	0.944	0.972	1.244	0.793	0.967	0.88	1.04	0.83
P11274	Breakpoint cluster region protein OS=Ho	BCR	142.82	28.271	4.9	5	6	4	1.02	1.242	1.061	0.937	0.909	1.223	0.764	0.95	0.88	0.94	0.76
Q2NKX8	DNA excision repair protein ERCC-6-lik	ERCC6L	141.1	27.951	4.6	4	4	4	0.991	1.064	0.881	0.934	1.015	1.143	0.937	1.165	0.88	1.05	1.02
Q9HCK8	Chromodomain-helicase-DNA-binding pr	CHD8	290.52	59.629	4.5	9	8	7	1.155	1.053	1.021	0.929	1.045	1.289	0.673	0.832	0.88	1.06	0.68
Q02978	Mitochondrial 2-oxoglutarate/malate carr	SLC25A11	34.061	45.424	23.9	7	8	6	1.065	1.023	0.929	0.915	1.023	1.065	0.991	1.057	0.88	1.00	0.98
Q96I59	Probable asparagine--tRNA ligase, mitoc	NARS2	54.089	12.101	5.7	2	2	2	1.114	0.965	0.875	0.961	1.065	0.873	1.153	0.99	0.88	0.93	1.03
P32929	Cystathionine gamma-lyase OS=Homo sa	CTH	44.508	70.202	27.4	8	8	7	0.973	1.223	0.947	0.992	0.814	0.891	0.986	0.961	0.88	0.78	0.89
P05386	60S acidic ribosomal protein P1 OS=Hor	RPLP1	11.514	95.952	51.8	2	7	2	1.046	1.167	0.969	0.985	0.988	1.261	0.757	0.909	0.88	1.02	0.75
Q5JTZ9	Alanine--tRNA ligase, mitochondrial OS	AARS2	107.34	111.36	9.7	8	10	8	1.125	1.014	0.962	0.926	1.023	0.967	0.963	0.967	0.88	0.93	0.90
Q9H6Z4	Ran-binding protein 3 OS=Homo sapiens	RANBP3	60.209	31.675	6.7	3	4	3	1.07	1.108	0.915	1.007	0.923	0.971	0.962	1.052	0.88	0.87	0.92
P50148	Guanine nucleotide-binding protein G(q)	GNAQ	42.142	37.515	22	7	8	4	1.08	1.053	0.907	0.975	0.928	0.97	1.002	1.059	0.88	0.89	0.97
O75506	Heat shock factor-binding protein 1 OS=	HSBP1	8.5435	76.458	47.4	3	8	3	1.107	1.136	1.003	0.976	0.892	0.889	1.063	0.943	0.88	0.79	0.89
Q9NZM1	Myoferlin OS=Homo sapiens OX=9606	MYOF	234.71	323.31	33.4	56	67	55	1.026	1.003	0.854	0.936	1.005	0.964	1.127	1.175	0.88	0.97	1.13
Q9Y294	Histone chaperone ASF1A OS=Homo sa	ASF1A	22.968	7.4985	7.4	1	2	1	1.047	1.049	0.921	0.928	0.998	1.404	0.757	0.982	0.88	1.15	0.83
P17844	Probable ATP-dependent RNA helicase I	DDX5	69.147	171.05	46.9	29	56	21	1.059	1.043	0.911	0.943	1.06	1.02	0.957	0.996	0.88	0.99	0.93
P60866	40S ribosomal protein S20 OS=Homo sa	RPS20	13.373	31.611	19.3	2	8	2	0.996	1.252	1.007	0.975	0.751	1.101	1	0.897	0.88	0.82	0.84
O00115	Deoxyribonuclease-2-alpha OS=Homo sa	DNASE2	39.58	8.4661	3.3	1	2	1	1.053	1.217	1.004	0.997	0.943	1.157	0.843	0.807	0.88	0.93	0.73
O15269	Serine palmitoyltransferase 1 OS=Homo	SPTLC1	52.743	70.822	20.7	10	12	10	1.102	1.134	1.005	0.966	0.967	1.171	0.86	0.896	0.88	0.96	0.79
P32455	Guanylate-binding protein 1 OS=Homo s	GBP1	67.93	96.215	15	8	10	5	1.095	1.156	1.024	0.96	0.795	0.785	1.263	1.02	0.88	0.70	1.01
P09493	Tropomyosin alpha-1 chain OS=Homo sa	TPM1	32.708	61.419	37.7	12	25	5	1.072	1.068	0.909	0.977	0.823	0.834	1.179	1.094	0.88	0.77	1.06
Q03701	CCAAT/enhancer-binding protein zeta O	CEBPZ	120.97	122.18	16.7	14	19	14	1.024	1.046	0.923	0.901	1.013	1.037	1.121	1.054	0.88	0.99	1.05
Q8WXH0	Nesprin-2 OS=Homo sapiens OX=9606	SYNE2	796.43	323.31	16.9	95	106	93	1.048	1.06	0.916	0.941	1.003	1.008	0.977	1.025	0.88	0.95	0.95
Q9UHA4	Ragulator complex protein LAMTOR3 O	LAMTOR3	13.623	53.206	30.6	2	4	2	0.998	1.336	1.002	1.054	0.693	1.327	0.693	0.936	0.88	0.87	0.70
P57735	Ras-related protein Rab-25 OS=Homo sa	RAB25	23.495	28.117	18.8	4	4	3	1.064	1.102	0.903	1.005	1.062	0.866	1.093	0.904	0.88	0.89	0.92
Q13686	Nucleic acid dioxygenase ALKBH1 OS=	ALKBH1	43.831	13.979	5.4	2	2	2	1.104	1.085	0.986	0.942	1.109	1.294	0.673	0.831	0.88	1.10	0.69
Q9ULX3	RNA-binding protein NOB1 OS=Homo s	NOB1	46.674	46.215	20.6	6	6	6	1.186	0.933	0.972	0.894	1.095	0.81	0.96	0.891	0.88	0.90	0.87
P49406	39S ribosomal protein L19, mitochondria	MRPL19	33.535	50.076	24.3	6	7	6	1.059	1.093	0.991	0.904	0.992	1.039	0.923	0.955	0.88	0.94	0.87
Q8IV63	Inactive serine/threonine-protein kinase V	VRK3	52.881	22.086	8	3	3	3	1.005	0.827	0.681	0.932	1.087	1.24	0.786	1.319	0.88	1.27	1.15
O76061	Stanniocalcin-2 OS=Homo sapiens OX=9	STC2	33.248	18.885	7	2	2	2	1.058	0.837	0.659	1.009	1.072	0.931	1.164	1.286	0.88	1.06	1.29
Q5VTU8	ATP synthase subunit epsilon-like protei	ATP5EP2	5.8068	10.888	31.4	2	2	2	1.095	1.142	1.027	0.942	0.927	0.991	0.922	0.991	0.88	0.86	0.86
O14732	Inositol monophosphatase 2 OS=Homo s	IMPA2	31.321	28.405	13.5	4	6	4	0.971	0.972	0.749	0.961	1.01	0.957	1.217	1.398	0.88	1.01	1.35
P36957	Dihydrolipoyllysine-residue succinyltrans	DLST	48.755	52.135	15.7	5	11	5	1.092	1.076	0.928	0.979	0.985	1.212	0.742	1.006	0.88	1.01	0.81
P61803	Dolichyl-diphosphooligosaccharide--prot	DAD1	12.497	14.097	17.7	2	2	2	0.958	1.056	0.727	1.044	0.971	0.907	1.064	1.349	0.88	0.93	1.20
P11171	Protein 4.1 OS=Homo sapiens OX=9606	EPB41	97.016	25.508	3.6	2	2	2	1.001	1.269	0.998	0.998	0.785	1.143	0.913	0.965	0.88	0.85	0.83
Q9C0E2	Exportin-4 OS=Homo sapiens OX=9606	XPO4	130.14	38.381	5.6	5	5	5	0.972	1.351	1.118	0.924	0.688	1.445	0.789	0.868	0.88	0.92	0.71
P15328	Folate receptor alpha OS=Homo sapiens	FOLR1	29.819	22.692	12.8	3	9	3	1	0.86	0.673	0.962	1.028	0.927	1.303	1.361	0.88	1.05	1.43
Q8N697	Solute carrier family 15 member 4 OS=H	SLC15A4	62.033	13.823	4.3	2	3	2	1.062	0.87	0.744	0.954	1.026	1.064	1.048	1.29	0.88	1.08	1.21
P00167	Cytochrome b5 OS=Homo sapiens OX=9	CYB5A	15.33	40.657	33.6	4	5	4	1.035	1.127	0.921	0.979	0.9	1.098	0.891	1.165	0.88	0.92	0.95
Q8IWR0	Zinc finger CCCH domain-containing pro	ZC3H7A	110.54	34.635	5.6	5	5	5	1.077	1.085	0.92	0.98	0.984	0.835	1.073	1.007	0.88	0.84	0.96
O14578	Citron Rho-interacting kinase OS=Homo	CIT	231.43	59.738	4.7	9	10	9	0.961	1.011	0.797	0.936	1.037	1.167	1.028	1.212	0.88	1.12	1.14
Q9BUE6	Iron-sulfur cluster assembly 1 homolog, r	ISCA1	14.179	29.972	40.3	4	5	4	1.079	1.115	0.98	0.948	0.976	0.919	0.976	1.052	0.88	0.86	0.92
Q92766	Ras-responsive element-binding protein 1	RREB1	181.42	92.019	8.1	10	11	10	1.123	1.12	0.976	0.995	0.91	1.078	0.838	0.946	0.88	0.89	0.80
O15488	Glycogenin-2 OS=Homo sapiens OX=96	GYG2	55.183	11.986	3.6	2	2	2	1.072	1.047	0.921	0.941	0.957	0.942	1.082	1.093	0.88	0.90	1.03
Q12849	G-rich sequence factor 1 OS=Homo sapie	GRSF1	53.126	94.216	26.2	12	13	12	1.096	1.082	0.955	0.958	0.949	1.104	0.907	0.972	0.88	0.94	0.86
P48147	Prolyl endopeptidase OS=Homo sapiens	PREP	80.699	137.29	28	15	20	15	1.051	1.168	0.974	0.975	0.903	0.991	0.954	0.995	0.88	0.85	0.88
O15067	Phosphoribosylformylglycinamidine synt	PFAS	144.73	183	20.8	19	22	18	1.073	1.072	0.962	0.922	1.003	0.99	0.992	1.009	0.88	0.93	0.93
P62263	40S ribosomal protein S14 OS=Homo sa	RPS14	16.273	135.83	39.7	8	16	8	1.04	1.081	0.944	0.918	1.015	1.013	1.004	1.075	0.88	0.96	0.98
Q8IYB8	ATP-dependent RNA helicase SUPV3L1	SUPV3L1	87.99	43.438	8.7	6	6	6	1.082	1.006	0.91	0.923	1.042	1.045	0.922	1.042	0.88	1.00	0.94
O75947	ATP synthase subunit d, mitochondrial C	ATP5H	18.491	58.872	52.8	7	12	7	1.038	1.048	0.868	0.963	1.054	1.144	0.957	1.034	0.88	1.05	0.95
P17812	CTP synthase 1 OS=Homo sapiens OX=9	CTPS1	66.69	122.74	25	13	21	12	1.057	1.141	0.952	0.977	0.951	0.932	1.033	0.995	0.88	0.86	0.92
P37108	Signal recognition particle 14 kDa protei	SRP14	14.57	38.507	27.9	5	6	5	1.076	1.054	0.909	0.96	1.04	1.048	0.829	1.129	0.88	0.98	0.92
O14925	Mitochondrial import inner membrane tra	TIMM23	21.943	52.004	16.7	3	4	3	1.067	1.075	0.911	0.968	1.038	0.906	1.139	0.922	0.88	0.91	0.96
P12694	2-oxoisovalerate dehydrogenase subunit a	BCKDHA	50.47	28.237	12.8	4	5	4	1.051	1.103	0.876	1.013	0.943	0.985	0.983	1.063	0.88	0.90	0.95
O94822	E3 ubiquitin-protein ligase listerin OS=H	LTN1	200.55	47.373	5.3	7	7	7	1.048	1.153	0.992	0.938	1.04	1.047	0.985	0.866	0.88	0.95	0.84
Q96P48	Arf-GAP with Rho-GAP domain, ANK re	ARAP1	162.19	13.587	1.9	2	2	2	1.109	1.112	0.924	1.023	0.925	0.912	1.134	0.811	0.88	0.83	0.88
Q5SSJ5	Heterochromatin protein 1-binding protei	HP1BP3	61.206	65.314	18.6	10	12	9	0.966	0.873	0.74	0.872	1.121	0.93	1.278	1.494	0.88	1.12	1.51
Q14318	Peptidyl-prolyl cis-trans isomerase FKBP	FKBP8	44.561	35.72	14.1	4	5	4	1.058	1.087	0.947	0.933	0.888	0.986	1.066	1.014	0.88	0.87	0.97
Q8WXF1	Paraspeckle component 1 OS=Homo sapi	PSPC1	58.743	151.32	30.4	14	22	14	1.083	1.11	0.946	0.976	0.989	1.014	0.891	0.975	0.88	0.91	0.85
P14625	Endoplasmin OS=Homo sapiens OX=96	HSP90B1	92.468	234.59	34.2	25	71	24	1.086	1.131	0.977	0.966	0.944	1.022	0.919	0.952	0.88	0.89	0.84
P01034	Cystatin-C OS=Homo sapiens OX=9606	CST3	15.799	14.454	13.7	2	3	2	1.042	1.021	0.841	0.967	1.106	1.194	0.887	0.997	0.88	1.11	0.91
P20674	Cytochrome c oxidase subunit 5A, mitocl	COX5A	16.762	30.5	10	1	6	1	1.078	1.082	0.916	0.977	0.979	1.237	0.828	1.029	0.88	1.03	0.86
Q9Y3D3	28S ribosomal protein S16, mitochondria	MRPS16	15.345	26.28	24.8	3	6	3	1.076	1.043	0.894	0.963	0.982	1.196	0.851	1.052	0.88	1.03	0.90
Q9NW15	Anoctamin-10 OS=Homo sapiens OX=96	ANO10	76.328	63.291	11.1	6	7	6	1.075	1.044	0.891	0.966	0.939	1.109	0.908	1.051	0.88	0.97	0.92
Q9BTT0	Acidic leucine-rich nuclear phosphoprote	ANP32E	30.692	60.252	10.4	2	4	2	0.992	1.28	0.992	0.999	0.818	1.004	0.967	0.964	0.88	0.80	0.85
Q9HD42	Charged multivesicular body protein 1a C	CHMP1A	21.703	13.904	8.7	2	4	2	1.109	1.065	0.925	0.98	1.01	1.069	0.866	0.971	0.88	0.96	0.84
Q8WU76	Sec1 family domain-containing protein 2	SCFD2	75.126	47.783	9.5	5	5	5	1.074	1.245	1.09	0.942	0.947	1.157	0.794	0.846	0.88	0.91	0.71
Q9UHD8	Septin-9 OS=Homo sapiens OX=9606 G	SEPT9	65.401	119.58	36.3	14	20	14	1.088	1.066	0.927	0.96	0.972	0.965	1.04	0.987	0.88	0.90	0.94
Q14011	Cold-inducible RNA-binding protein OS	CIRBP	18.648	12.391	14.5	2	3	2	1.017	1.18	0.996	0.928	0.901	1.287	0.786	1.02	0.88	1.00	0.82
Q9Y487	V-type proton ATPase 116 kDa subunit a	ATP6V0A2	98.081	29.993	6.4	4	4	4	1.009	1.099	0.864	0.982	0.97	0.944	1.091	1.114	0.88	0.91	1.05
Q9P0S2	Cytochrome c oxidase assembly protein C	COX16	12.293	6.477	13.2	1	1	1	0.95	1.308	0.965	1.012	0.759	1.168	0.867	1.079	0.88	0.85	0.86
Q8N5K1	CDGSH iron-sulfur domain-containing p	CISD2	15.278	27.462	37	4	5	4	1.099	1.094	0.964	0.956	0.975	1.112	0.844	1.023	0.88	0.95	0.85
Q00059	Transcription factor A, mitochondrial OS	TFAM	29.096	40.458	24.4	5	8	5	1.095	1.001	0.908	0.927	0.992	1.012	1.151	1.072	0.88	0.96	1.06
O75390	Citrate synthase, mitochondrial OS=Hom	CS	51.712	71.923	22.5	10	20	10	1.04	1.087	0.946	0.916	1.017	1.061	0.986	0.964	0.88	0.98	0.92
Q9UMS0	NFU1 iron-sulfur cluster scaffold homolo	NFU1	28.462	21.088	16.1	3	3	3	1.065	1.026	0.918	0.912	0.957	1.264	0.876	0.935	0.88	1.06	0.87
O15126	Secretory carrier-associated membrane pr	SCAMP1	37.92	17.682	7.1	2	4	2	1.023	1.092	0.817	1.034	0.924	0.985	0.982	1.171	0.88	0.90	1.02
Q8TEM1	Nuclear pore membrane glycoprotein 210	NUP210	205.11	136.86	9	13	13	13	1.018	1.104	0.877	0.98	0.916	1.032	1.026	1.063	0.88	0.92	0.98
P10809	60 kDa heat shock protein, mitochondrial	HSPD1	61.054	301.74	55.3	24	70	21	1.052	1.11	0.933	0.959	0.96	1.085	0.891	1.004	0.88	0.95	0.88
Q9H902	Receptor expression-enhancing protein 1	REEP1	22.254	9.2636	8.5	1	2	1	1.054	0.963	0.758	1.006	1.006	1.047	1.038	1.148	0.87	1.02	1.08
Q5T5U3	Rho GTPase-activating protein 21 OS=H	ARHGAP2	217.33	46.226	4.1	7	7	7	1.091	1.06	0.97	0.911	1.005	0.952	1.038	1.034	0.87	0.91	0.96
Q9NZL4	Hsp70-binding protein 1 OS=Homo sapie	HSPBP1	39.474	37.359	18.8	5	6	5	1.071	1.116	0.963	0.949	0.985	0.902	1.076	0.978	0.87	0.86	0.94
Q969U7	Proteasome assembly chaperone 2 OS=H	PSMG2	29.396	36.449	16.3	4	6	4	1.092	1.126	1.017	0.922	1	0.97	0.943	0.852	0.87	0.89	0.81
Q5JS37	NHL repeat-containing protein 3 OS=Ho	NHLRC3	38.282	13.565	8.1	2	2	2	1.115	1.046	0.915	0.974	0.988	0.94	1.061	0.948	0.87	0.89	0.93
Q9H3K2	Growth hormone-inducible transmembrar	GHITM	37.205	11.579	5.8	2	2	2	1.085	1.034	0.88	0.972	1.001	0.955	0.992	1.105	0.87	0.92	0.99
Q96P11	Probable 28S rRNA (cytosine-C(5))-meth	NSUN5	46.691	50.823	17.2	6	8	6	1.045	0.953	0.854	0.892	1.145	1.063	0.993	1.109	0.87	1.11	1.05
Q96A35	39S ribosomal protein L24, mitochondria	MRPL24	24.915	12.473	12.5	2	3	2	1.106	1.014	0.951	0.901	1.057	1.136	0.873	1.019	0.87	1.03	0.89
Q7L1Q6	Basic leucine zipper and W2 domain-con	BZW1	48.043	59.335	20	9	15	9	1.067	1.115	0.927	0.979	0.93	1.025	0.922	1.032	0.87	0.90	0.90
Q16658	Fascin OS=Homo sapiens OX=9606 GN	FSCN1	54.529	219.55	45.8	18	32	18	1.155	1.374	1.16	1.049	0.64	0.82	0.956	0.757	0.87	0.58	0.68
Q8TAV0	Protein FAM76A OS=Homo sapiens OX	FAM76A	35.049	12.789	7.2	2	2	2	1.113	1.114	0.977	0.968	0.945	0.828	1.103	0.947	0.87	0.80	0.92
O15145	Actin-related protein 2/3 complex subuni	ARPC3	20.546	20.866	21.3	3	3	3	1.094	1.191	1.009	0.986	0.892	1.006	1.006	0.938	0.87	0.83	0.85
Q9NWS6	Protein FAM118A OS=Homo sapiens OX	FAM118A	40.269	28.493	6.7	3	3	3	1.14	1.149	1.03	0.968	1.052	0.823	1.08	0.72	0.87	0.82	0.79
O43251	RNA binding protein fox-1 homolog 2 O	RBFOX2	41.373	39.067	10.5	2	2	2	0.992	1.257	0.93	1.033	0.709	1.191	0.935	1.01	0.87	0.84	0.86
Q96QK1	Vacuolar protein sorting-associated prote	VPS35	91.706	181.72	27	17	25	17	1.059	1.118	0.956	0.944	0.969	0.962	1.031	1.095	0.87	0.89	0.98
Q9P013	Spliceosome-associated protein CWC15	CWC15	26.624	11.815	3.9	2	3	2	1.054	1.073	0.973	0.883	1.032	1.245	0.831	1.025	0.87	1.07	0.87
Q92841	Probable ATP-dependent RNA helicase I	DDX17	80.272	323.31	47.6	32	64	24	1.051	1.083	0.918	0.944	0.986	1.129	0.903	1	0.87	0.99	0.89
Q96EK5	KIF1-binding protein OS=Homo sapiens	KIF1BP	71.813	22.384	5.5	3	3	3	1.055	1.028	0.805	1.012	1.062	0.829	1.092	1.128	0.87	0.91	1.07
Q9BT17	Mitochondrial ribosome-associated GTPa	MTG1	37.236	21.339	12.3	3	4	3	1.188	0.946	0.948	0.913	1.116	0.869	1.087	0.903	0.87	0.93	0.93
Q9NWZ5	Uridine-cytidine kinase-like 1 OS=Homo	UCKL1	61.14	12.513	3.6	2	3	2	1.032	1.133	1.044	0.843	1.116	0.947	1.042	0.998	0.87	0.95	0.94
Q86X19	Transmembrane protein 17 OS=Homo sa	TMEM17	23.046	6.4159	4	1	1	1	1.057	1.058	0.909	0.934	0.956	0.963	1.098	1.096	0.87	0.91	1.04
Q9BRJ2	39S ribosomal protein L45, mitochondria	MRPL45	35.351	40.507	23.2	6	9	6	1.12	1.119	0.983	0.968	0.987	1.242	0.742	0.899	0.87	1.00	0.73
Q9Y484	WD repeat domain phosphoinositide-inte	WDR45	39.867	21.281	9.4	3	3	3	1.024	0.972	0.795	0.944	1.075	0.979	1.052	1.254	0.87	1.03	1.16
P53582	Methionine aminopeptidase 1 OS=Homo	METAP1	43.215	77.762	26.4	8	9	8	1.022	1.244	0.943	1.031	0.863	1.13	0.868	0.961	0.87	0.88	0.81
Q8IWI9	MAX gene-associated protein OS=Homo	MGA	331.83	40.097	2.5	6	6	6	1.051	1.075	0.929	0.922	0.939	1.226	0.881	0.993	0.87	1.02	0.88
Q9UKD2	mRNA turnover protein 4 homolog OS=H	MRTO4	27.56	31.34	19.7	4	7	4	1.035	1.141	0.924	0.97	0.98	1.232	0.833	1.037	0.87	1.02	0.86
P63098	Calcineurin subunit B type 1 OS=Homo	PPP3R1	19.3	40.719	31.8	4	6	4	1.068	1.207	0.98	1	0.832	0.962	1.06	0.996	0.87	0.79	0.90
P60604	Ubiquitin-conjugating enzyme E2 G2 OS	UBE2G2	18.566	13.129	14.5	2	2	2	1.111	1.084	1.001	0.909	0.993	0.955	0.927	1.072	0.87	0.89	0.91
Q96GX5	Serine/threonine-protein kinase greatwall	MASTL	97.318	42.342	6.5	5	6	5	0.954	0.938	0.68	0.966	1.076	1.297	0.892	1.337	0.87	1.25	1.18
P49247	Ribose-5-phosphate isomerase OS=Homo	RPIA	33.269	25.761	12.5	4	5	4	1.144	1.231	1.116	0.95	0.881	1.141	0.696	0.874	0.87	0.85	0.66
Q8TDM6	Disks large homolog 5 OS=Homo sapien	DLG5	213.87	85.595	7.6	13	13	13	1.094	1.141	0.968	0.975	0.916	0.973	0.952	0.991	0.87	0.85	0.87
P51149	Ras-related protein Rab-7a OS=Homo sa	RAB7A	23.489	165.48	72	15	28	15	1.099	1.066	0.907	0.975	0.907	0.973	1.03	1.014	0.87	0.87	0.94
Q13425	Beta-2-syntrophin OS=Homo sapiens OX	SNTB2	57.949	36.845	11.5	6	5	5	0.988	1.213	0.959	0.954	0.977	1.34	0.726	0.961	0.87	1.05	0.77
Q12770	Sterol regulatory element-binding protein	SCAP	139.73	7.2581	0.8	1	1	1	1.174	1.133	0.956	1.049	0.891	0.909	0.885	0.892	0.87	0.78	0.77
Q99536	Synaptic vesicle membrane protein VAT-	VAT1	41.92	76.786	30.8	9	12	9	1.064	1.339	1.023	1.065	0.77	0.855	1.094	0.999	0.87	0.68	0.87
Q9Y6M4	Casein kinase I isoform gamma-3 OS=Ho	CSNK1G3	51.388	32.348	10.7	5	6	5	1.045	1.143	0.893	1.008	0.978	0.978	0.897	0.954	0.87	0.89	0.85
Q9H490	Phosphatidylinositol glycan anchor biosy	PIGU	50.051	22.465	7.8	3	5	3	1.086	1.129	0.927	0.997	0.924	0.87	1.023	1.044	0.87	0.81	0.93
Q14684	Ribosomal RNA processing protein 1 hor	RRP1B	84.427	81.715	19.1	11	12	10	1.078	0.983	0.899	0.891	1.12	0.936	1.076	0.977	0.87	1.00	1.00
Q9UM47	Neurogenic locus notch homolog protein	NOTCH3	243.63	11.633	0.7	2	2	1	1.163	1.11	0.956	1.018	0.962	0.891	0.903	0.918	0.87	0.82	0.80
P11177	Pyruvate dehydrogenase E1 component su	PDHB	39.233	68.256	20.6	6	13	6	1.102	1.077	0.979	0.913	1.009	1.222	0.922	0.998	0.87	1.02	0.88
O14757	Serine/threonine-protein kinase Chk1 OS	CHEK1	54.433	18.468	6.1	3	3	3	1.015	1.018	0.898	0.867	1.071	1.255	0.968	1.064	0.87	1.14	1.00
O14936	Peripheral plasma membrane protein CAS	CASK	105.12	12.839	2.3	2	2	2	1.107	1.1	0.987	0.929	0.939	0.961	0.985	1.03	0.87	0.86	0.91

P61599	N-alpha-acetyltransferase 20 OS=Homo s	NAA20	20.368	6.1798	5.1	1	1	1	1.043	1.164	0.94	0.975	1.042	1.189	0.711	0.993	0.87	1.01	0.77
P14735	Insulin-degrading enzyme OS=Homo sap	IDE	117.97	153.77	18.7	17	20	17	1.108	0.931	0.816	0.953	1.052	0.941	1.073	1.032	0.87	0.98	1.03
Q14240	Eukaryotic initiation factor 4A-II OS=Ho	EIF4A2	46.402	23.975	28.3	11	31	3	1.059	1.19	0.956	0.995	0.888	0.862	1.047	1.029	0.87	0.78	0.92
P48553	Trafficking protein particle complex subu	TRAPPC1(	142.19	7.6413	0.9	1	1	1	1.018	0.85	0.652	0.968	1.199	0.971	1.134	1.282	0.87	1.16	1.29
Q15070	Mitochondrial inner membrane protein O	OXA1L	48.547	38.088	13.1	6	11	6	1.017	1.092	0.922	0.907	1.074	1.03	0.989	0.976	0.87	1.00	0.93
Q13772	Nuclear receptor coactivator 4 OS=Homo	NCOA4	69.725	62.275	14.5	9	9	9	1.045	1.101	0.917	0.944	1.011	1.282	0.856	0.959	0.87	1.07	0.85
P25705	ATP synthase subunit alpha, mitochondri	ATP5F1A	59.75	263.66	42	21	38	19	1.075	1.114	0.939	0.959	0.941	1.078	0.911	0.991	0.87	0.92	0.87
Q9ULW3	Activator of basal transcription 1 OS=Ho	ABT1	31.079	26.609	17.3	4	4	4	0.988	1.043	0.799	0.962	0.936	1.172	0.933	1.285	0.87	1.04	1.09
P27448	MAP/microtubule affinity-regulating kina	MARK3	84.428	44.498	10.5	6	7	2	1.066	1.014	0.863	0.94	0.988	0.843	1.211	1.133	0.87	0.88	1.13
O95831	Apoptosis-inducing factor 1, mitochondri	AIFM1	66.9	124.23	27.4	14	21	14	1.061	1.111	0.927	0.955	0.94	1.194	0.873	0.931	0.87	0.98	0.83
O60762	Dolichol-phosphate mannosyltransferase	DPM1	29.634	27.453	15	4	7	4	1.054	1.028	0.85	0.954	1.12	1.216	0.831	1.038	0.87	1.12	0.90
P11802	Cyclin-dependent kinase 4 OS=Homo sa	CDK4	33.729	39.185	20.5	5	5	4	1.064	1.002	0.817	0.973	1.08	1.06	0.911	1.13	0.87	1.04	0.99
O75608	Acyl-protein thioesterase 1 OS=Homo sa	LYPLA1	24.669	12.545	8.3	2	3	2	1.087	1.104	1.036	0.862	0.996	1.138	0.922	0.956	0.87	0.97	0.86
O94886	CSC1-like protein 1 OS=Homo sapiens (	TMEM63A	92.125	16.86	3.3	2	3	2	0.995	0.985	0.697	1.018	0.973	1.019	1.08	1.299	0.87	1.01	1.20
Q9BQ39	ATP-dependent RNA helicase DDX50 O	DDX50	82.564	51.725	13.2	9	14	7	1.074	1.003	0.897	0.902	0.981	1.205	0.949	1.034	0.87	1.05	0.95
Q13765	Nascent polypeptide-associated complex	NACA	23.384	51.054	19.5	3	11	3	1.027	1.254	0.972	1.003	0.851	0.968	0.919	0.972	0.87	0.80	0.83
Q9Y5N6	Origin recognition complex subunit 6 OS	ORC6	28.107	25.173	10.7	4	5	4	1.016	1.019	0.841	0.921	1.039	1.075	1.036	1.151	0.87	1.04	1.07
O00471	Exocyst complex component 5 OS=Homo	EXOC5	81.852	93.793	18.1	12	13	12	1.086	1.126	0.936	0.979	0.971	0.964	0.955	0.99	0.87	0.87	0.88
P63244	Receptor of activated protein C kinase 1 (	RACK1	35.076	218.92	61.8	14	38	14	1.049	1.187	0.981	0.954	0.892	1.204	0.805	0.916	0.87	0.94	0.77
Q9UJK0	Ribosome biogenesis protein TSR3 homo	TSR3	33.596	6.3397	2.6	1	1	1	1.043	1.162	0.979	0.929	1.034	1.241	0.711	0.993	0.87	1.03	0.77
Q9NTX5	Ethylmalonyl-CoA decarboxylase OS=Ho	ECHDC1	33.698	65.32	31.3	8	10	8	1.051	1.06	0.861	0.965	1.069	1.115	0.919	0.957	0.86	1.03	0.89
Q99952	Tyrosine-protein phosphatase non-recepto	PTPN18	50.482	7.5492	2.8	1	1	1	1.115	0.988	0.974	0.845	1.123	1.151	0.86	1.037	0.86	1.08	0.90
Q6PJF5	Inactive rhomboid protein 2 OS=Homo sa	RHBDF2	96.685	25.844	4	3	3	3	0.825	1.514	0.962	1.061	0.697	1.36	0.788	0.966	0.86	0.88	0.75
O95336	6-phosphogluconolactonase OS=Homo sa	PGLS	27.547	52.314	33.7	7	9	7	1.101	1.149	0.986	0.96	0.923	1.118	0.873	0.876	0.86	0.91	0.78
P31350	Ribonucleoside-diphosphate reductase su	RRM2	44.877	144.79	40.1	13	23	11	0.735	0.486	0.342	0.714	1.585	1.259	1.477	1.944	0.86	2.33	2.80
O00566	U3 small nucleolar ribonucleoprotein pro	MPHOSPH	78.863	53.283	12.2	6	7	6	1.027	0.961	0.781	0.938	0.975	0.981	1.123	1.076	0.86	0.98	1.11
Q9BX95	Sphingosine-1-phosphate phosphatase 1 (	SGPP1	49.107	27.458	12	4	4	3	1.148	1.032	0.995	0.89	1.04	1.199	0.813	0.904	0.86	1.03	0.79
O43933	Peroxisome biogenesis factor 1 OS=Hom	PEX1	142.87	6.712	0.6	1	1	1	1.071	1.188	1.026	0.927	1.052	0.831	1.146	0.81	0.86	0.83	0.87
Q96RU2	Ubiquitin carboxyl-terminal hydrolase 28	USP28	122.49	22.132	3	3	3	3	0.93	1.048	0.766	0.944	0.995	1.28	0.823	1.399	0.86	1.15	1.12
P08559	Pyruvate dehydrogenase E1 component s	PDHA1	43.295	137.44	43.6	13	19	12	1.061	1.14	0.958	0.944	0.977	1.039	0.919	1.007	0.86	0.92	0.88
Q15904	V-type proton ATPase subunit S1 OS=H	ATP6AP1	52.025	62.139	16.4	6	7	6	1.167	1.061	1.001	0.924	1.016	0.974	0.889	0.94	0.86	0.89	0.82
O14979	Heterogeneous nuclear ribonucleoprotein	HNRNPDL	46.437	61.204	12.1	5	8	5	1.049	1.317	1.021	1.022	0.784	1.067	0.896	0.914	0.86	0.78	0.77
Q5SVZ6	Zinc finger MYM-type protein 1 OS=Ho	ZMYM1	128.72	6.3001	0.9	1	1	1	1.18	0.94	0.86	0.97	1.001	0.871	1.062	1.062	0.86	0.88	1.00
Q9H074	Polyadenylate-binding protein-interacting	PAIP1	53.524	55.483	19.6	8	9	8	1.118	1.184	1.011	0.976	0.845	0.952	1.039	0.906	0.86	0.78	0.84
Q9Y303	N-acetylglucosamine-6-phosphate deacet	AMDHD2	43.747	62.718	23.5	7	9	7	1.142	1.116	0.976	0.973	0.927	0.931	0.923	0.89	0.86	0.82	0.80
P16591	Tyrosine-protein kinase Fer OS=Homo sa	FER	94.637	34.505	7.8	5	6	5	1.078	1.137	0.912	0.999	0.921	0.953	1.01	0.972	0.86	0.85	0.89
P46777	60S ribosomal protein L5 OS=Homo sap	RPL5	34.362	143.8	35.4	13	32	13	1.048	1.074	0.885	0.945	0.989	0.997	0.99	1.103	0.86	0.94	0.99
Q9NRG4	N-lysine methyltransferase SMYD2 OS=	SMYD2	49.688	31.136	11.5	4	4	4	1.023	1.141	0.896	0.97	0.897	0.99	0.961	1.027	0.86	0.87	0.92
Q9BYC5	Alpha-(1,6)-fucosyltransferase OS=Homo	FUT8	66.515	36.548	13.2	6	7	6	1.091	1.057	0.902	0.95	0.953	0.873	1.062	1.162	0.86	0.85	1.04
Q9Y2Q3	Glutathione S-transferase kappa 1 OS=H	GSTK1	25.497	72.1	33.6	6	10	6	1.076	0.985	0.912	0.864	1.107	0.948	1.161	1.161	0.86	1.00	1.13
Q9H9B4	Sideroflexin-1 OS=Homo sapiens OX=9(	SFXN1	35.619	91.836	29.2	9	12	9	1.064	1.08	0.948	0.899	1.056	1.074	0.983	0.984	0.86	0.99	0.92
P16144	Integrin beta-4 OS=Homo sapiens OX=9(	ITGB4	202.16	323.31	22.4	30	42	29	1.101	1.136	0.936	0.991	0.892	0.871	1.053	0.977	0.86	0.79	0.91
P38646	Stress-70 protein, mitochondrial OS=Hom	HSPA9	73.68	323.31	46.1	30	85	30	1.075	1.082	0.929	0.929	0.985	0.993	0.998	1.015	0.86	0.92	0.93
Q8NC26	Zinc finger protein 114 OS=Homo sapien	ZNF114	47.746	25.344	10.3	4	5	3	1.077	1.028	0.85	0.963	1.047	0.821	1.104	1.141	0.86	0.89	1.07
P56182	Ribosomal RNA processing protein 1 hom	RRP1	52.839	79.69	18.4	9	11	9	1.095	1.053	0.881	0.969	1.066	1.05	0.918	1.013	0.86	0.99	0.90
O75534	Cold shock domain-containing protein E1	CSDE1	88.884	127.05	24.4	16	20	15	1.099	1.22	1.008	0.989	0.89	1.137	0.854	0.895	0.86	0.87	0.75
O60870	DNA/RNA-binding protein KIN17 OS=H	KIN	45.373	30.029	14.8	5	6	5	1.011	1.177	0.986	0.898	0.877	1.091	1.105	0.99	0.86	0.90	0.96
Q9BQ48	39S ribosomal protein L34, mitochondria	MRPL34	10.165	11.728	12	2	2	2	0.949	1.045	0.797	0.919	0.948	0.947	1.143	1.435	0.86	0.95	1.29
P21359	Neurofibromin OS=Homo sapiens OX=9	NF1	319.37	96.487	5.1	13	15	13	1.079	1.119	0.934	0.956	0.938	1.011	0.935	0.99	0.86	0.89	0.88
P62857	40S ribosomal protein S28 OS=Homo sa	RPS28	7.8409	28.095	46.4	3	8	3	1.17	0.884	0.8	0.966	1.126	1.053	0.868	1.024	0.86	1.06	0.92
P35221	Catenin alpha-1 OS=Homo sapiens OX=	CTNNA1	100.07	323.31	45.8	37	70	37	1.079	1.133	0.938	0.963	0.901	0.963	0.985	1.004	0.86	0.84	0.90
Q13574	Diacylglycerol kinase zeta OS=Homo sap	DGKZ	124.13	106.85	12.1	9	11	9	1.034	1.37	1.072	0.994	0.723	0.839	1.148	0.972	0.86	0.65	0.88
P05976	Myosin light chain 1/3, skeletal muscle is	MYL1	21.145	30.72	8.2	2	4	2	1.07	1.318	1.059	0.993	0.724	1.058	0.876	0.932	0.86	0.75	0.76
Q9UHY8	Fasciculation and elongation protein zeta	FEZ2	39.666	27.089	13.6	4	4	4	0.968	0.943	0.695	0.947	1.28	0.901	1.146	1.247	0.86	1.14	1.25
Q6P4E1	Protein CASC4 OS=Homo sapiens OX=9	CASC4	48.864	7.4751	2.8	1	1	1	1.041	0.978	0.734	1	1.12	0.741	1.177	1.241	0.86	0.92	1.20
Q86Y39	NADH dehydrogenase [ubiquinone] 1 alp	NDUFA11	14.852	22.643	22.7	2	2	2	1.001	1.172	0.951	0.915	0.927	1.2	0.965	1.001	0.86	0.98	0.90
Q9BWS9	Chitinase domain-containing protein 1 O	CHID1	44.94	15.536	5.1	2	3	2	1.06	1.167	0.95	0.962	0.963	1.257	0.854	0.999	0.86	1.00	0.83
O43865	S-adenosylhomocysteine hydrolase-like p	AHCYL1	58.951	123.06	25.7	13	19	4	1.131	1.131	0.972	0.97	1.02	1.072	0.819	0.885	0.86	0.92	0.75
Q9Y3B3	Transmembrane emp24 domain-containin	TMED7	25.171	24.543	15.2	3	6	3	1.058	1.189	1.033	0.895	0.889	1.093	0.971	0.864	0.86	0.88	0.82
P53350	Serine/threonine-protein kinase PLK1 OS	PLK1	68.254	15.845	4.5	2	2	2	1.156	1.242	1.059	0.998	0.857	1.041	0.794	0.798	0.86	0.79	0.66
P55011	Solute carrier family 12 member 2 OS=H	SLC12A2	131.45	21.442	3.1	3	3	3	1.008	1.024	0.785	0.958	1.068	1.262	0.758	1.241	0.86	1.15	0.98
P46734	Dual specificity mitogen-activated protein	MAP2K3	39.318	55.837	24.5	8	8	5	1.036	1.387	1.049	1.029	0.687	1.168	0.822	0.836	0.86	0.77	0.68
Q96ST2	Protein IWS1 homolog OS=Homo sapien	IWS1	91.954	50.033	7.9	5	7	5	1.054	1.017	0.911	0.865	1.002	0.978	1.174	1.032	0.86	0.96	1.07
Q96EL3	39S ribosomal protein L53, mitochondria	MRPL53	12.107	28.695	41.1	4	7	4	1.119	1.12	0.969	0.951	0.966	1.194	0.829	0.92	0.86	0.96	0.78
Q5VV42	Threonylcarbamoyladenosine tRNA meth	CDKAL1	65.111	46.287	7.6	4	5	4	1.054	1.113	0.913	0.945	0.991	1.137	0.953	0.968	0.86	0.98	0.89
Q96IX5	Up-regulated during skeletal muscle grow	USMG5	6.4575	40.916	44.8	3	5	3	1.131	1.064	0.935	0.947	0.965	1.11	0.867	1.042	0.86	0.95	0.87
Q9NPH2	Inositol-3-phosphate synthase 1 OS=Hom	ISYNA1	61.067	149.41	37.5	13	25	13	1.088	1.14	0.996	0.914	1.002	0.952	0.989	0.943	0.86	0.88	0.87
Q9H9J2	39S ribosomal protein L44, mitochondria	MRPL44	37.535	65.335	24.7	6	8	6	1.012	1.143	0.911	0.936	0.988	1.065	0.979	0.914	0.86	0.95	0.88
Q8N5P1	Zinc finger CCCH domain-containing pro	ZC3H8	33.575	6.2898	4.8	1	1	1	1.024	1.326	0.996	1.018	0.712	1.327	0.668	0.979	0.86	0.87	0.70
P30040	Endoplasmic reticulum resident protein 2	ERP29	28.993	76.351	39.5	10	23	10	1.14	1.112	0.985	0.945	0.921	0.989	0.968	0.925	0.86	0.85	0.84
Q8NB37	Glutamine amidotransferase-like class 1 c	GATD1	23.297	42.063	19.5	3	4	3	1.022	1.2	0.935	0.969	0.983	0.893	1.085	0.981	0.86	0.84	0.93
Q9BXL8	Cell division cycle-associated protein 4 C	CDCA4	26.113	6.0819	3.7	1	1	1	1.137	1.144	0.954	1	0.964	0.902	0.997	0.852	0.86	0.82	0.81
Q9NSY1	BMP-2-inducible protein kinase OS=Hom	BMP2K	129.17	18.103	2.7	2	2	2	1.111	1.14	0.913	1.015	0.952	0.943	0.933	0.959	0.86	0.84	0.84
P19793	Retinoic acid receptor RXR-alpha OS=H	RXRA	50.811	50.571	16.2	7	8	4	1.113	1.095	0.849	1.042	0.937	0.988	0.9	1.023	0.86	0.87	0.87
Q96EA4	Protein Spindly OS=Homo sapiens OX=9	SPDL1	70.171	50.69	14.7	8	9	8	1.157	1.064	0.972	0.93	1.047	0.98	0.951	1.041	0.86	0.91	0.90
P53597	Succinate--CoA ligase [ADP/GDP-formin	SUCLG1	36.249	56.023	18.5	5	10	5	1.072	1.128	0.921	0.963	0.925	1.025	0.882	0.917	0.86	0.89	0.82
Q8IY81	pre-rRNA processing protein FTSJ3 OS=	FTSJ3	96.557	148.18	18.8	12	14	12	1.055	1.003	0.857	0.905	1.086	1.155	0.911	1.087	0.86	1.09	0.97
Q9Y2K2	Serine/threonine-protein kinase SIK3 OS	SIK3	144.85	7.5435	1.3	1	1	1	1.029	1.366	1.087	0.963	0.7	0.942	1.138	0.846	0.86	0.69	0.83
Q9P000	COMM domain-containing protein 9 OS=	COMMD9	21.819	19.257	21.2	3	3	3	1.048	1.176	0.886	1.017	0.9	0.882	1.231	0.864	0.86	0.80	0.94
O94927	HAUS augmin-like complex subunit 5 OS	HAUS5	71.682	7.096	2.1	1	2	1	1.083	1.05	0.938	0.887	1.024	1.289	0.763	1.06	0.86	1.08	0.85
Q9H9Z2	Protein lin-28 homolog A OS=Homo sap	LIN28A	22.743	12.129	17.2	3	3	2	1.131	1.431	1.168	1.024	0.612	0.985	0.82	0.781	0.86	0.62	0.62
Q96G21	U3 small nucleolar ribonucleoprotein pro	IMP4	33.756	14.439	8.2	2	2	2	0.991	0.878	0.636	0.963	1.105	0.986	1.197	1.35	0.86	1.12	1.36
Q9UKK9	ADP-sugar pyrophosphatase OS=Homo s	NUDT5	24.327	92.01	43.4	9	15	9	1.11	1.082	0.92	0.955	0.939	0.946	1.059	0.997	0.86	0.86	0.94
Q9Y2H6	Fibronectin type-III domain-containing pr	FNDC3A	131.85	44.001	7.2	6	5	5	1.1	1.077	0.953	0.909	0.957	1.016	1.036	0.937	0.86	0.91	0.91
P28347	Transcriptional enhancer factor TEF-1 OS	TEAD1	47.945	6.444	9.6	4	6	1	1.102	1.261	0.984	1.037	0.804	1.205	0.732	0.839	0.86	0.85	0.66
Q9NVS2	39S ribosomal protein S18a, mitochondri	MRPS18A	22.184	15.414	13.8	2	2	2	1.076	0.948	0.754	0.977	1.041	1.233	0.612	1.405	0.86	1.12	1.00
Q9Y2X0	Mediator of RNA polymerase II transcrip	MED16	96.792	34.084	5.9	5	5	5	1.011	1.092	0.948	0.85	1.066	0.87	1.17	1.165	0.85	0.92	1.11
O43464	Serine protease HTRA2, mitochondrial C	HTRA2	48.84	28.093	8.1	3	3	3	1.065	1.038	0.88	0.917	1.047	1.209	0.832	1.097	0.85	1.07	0.92
P62072	Mitochondrial import inner membrane tra	TIMM10	10.333	32.7	25.6	3	3	3	1.066	1.112	0.928	0.933	0.925	1.234	0.868	1.004	0.85	0.99	0.86
Q9Y2T2	AP-3 complex subunit mu-1 OS=Homo s	AP3M1	46.939	47.506	21.1	7	8	7	1.05	1.048	0.816	0.976	0.85	0.951	1.064	1.022	0.85	0.86	0.99
Q13614	Myotubularin-related protein 2 OS=Hom	MTMR2	73.38	25.899	5.6	2	2	2	1.137	1.137	0.932	1.01	0.889	0.993	0.918	0.935	0.85	0.83	0.81
P28370	Probable global transcription activator SN	SMARCA1	122.6	45.9	13.6	14	20	6	1.058	1.099	0.913	0.929	1.034	1.167	0.904	1.006	0.85	1.02	0.89
P30626	Sorcin OS=Homo sapiens OX=9606 GN=	SRI	21.676	59.167	36.4	6	8	6	1.056	1.19	0.943	0.975	0.877	0.944	1.06	1.001	0.85	0.81	0.92
O15230	Laminin subunit alpha-5 OS=Homo sapie	LAMA5	399.73	323.31	16.7	46	55	46	1.055	1.143	0.928	0.949	0.939	0.969	0.962	1.025	0.85	0.87	0.90
O15091	Mitochondrial ribonuclease P catalytic su	KIAA0391	67.315	27.72	2.7	1	1	1	1.32	0.792	0.963	0.84	1.22	0.702	1.142	0.94	0.85	0.91	0.99
Q9UNQ2	Probable dimethyladenosine transferase (	DIMT1	35.236	64.731	28.1	8	12	8	1.086	0.971	0.867	0.889	1.109	1.043	0.982	1.126	0.85	1.05	1.02
Q16527	Cysteine and glycine-rich protein 2 OS=H	CSRP2	20.954	53.678	26.4	4	6	4	1	0.981	0.757	0.934	1.026	0.968	1.141	1.392	0.85	1.01	1.28
Q9NR31	GTP-binding protein SAR1a OS=Homo s	SAR1A	22.367	60.269	42.4	9	13	4	1.19	1.072	0.986	0.944	0.867	0.936	0.943	0.993	0.85	0.80	0.86
Q5T4B2	Inactive glycosyltransferase 25 family me	CERCAM	67.591	25.389	7.4	4	4	4	1.163	1.153	0.968	1.008	0.9	1.07	0.847	0.919	0.85	0.85	0.76
Q969M3	Protein YIPF5 OS=Homo sapiens OX=9(	YIPF5	27.989	6.5966	3.5	1	1	1	1.024	1.133	0.948	0.892	1.023	1.071	1.032	1.007	0.85	0.97	0.95
Q9Y289	Sodium-dependent multivitamin transpor	SLC5A6	68.641	45.151	8.5	4	7	4	1.024	1.254	0.961	0.982	0.864	1.088	0.86	1.009	0.85	0.86	0.82
P51003	Poly(A) polymerase alpha OS=Homo sap	PAPOLA	82.842	243.71	40.8	24	41	22	1.114	1.111	0.949	0.948	0.969	1.136	0.895	0.925	0.85	0.95	0.82
Q9UK76	Jupiter microtubule associated homolog 1	JPT1	16.014	79.254	37.7	4	8	4	1.11	1.335	1.042	1.042	0.773	0.888	0.976	0.803	0.85	0.68	0.73
Q9Y3A4	Ribosomal RNA-processing protein 7 ho	RRP7A	32.334	48.561	24.3	6	8	6	0.976	0.994	0.813	0.866	1.156	1.292	0.957	1.135	0.85	1.24	1.06
Q92692	Nectin-2 OS=Homo sapiens OX=9606 G	NECTIN2	57.741	28.895	10.6	4	5	4	1.017	1.093	0.834	0.964	1.025	1.129	0.807	1.224	0.85	1.02	0.96
P98095	Fibulin-2 OS=Homo sapiens OX=9606 (	FBLN2	126.57	22.576	3.3	3	3	3	1.044	1.032	0.888	0.881	1.141	1.269	0.891	1.086	0.85	1.16	0.95
P30044	Peroxiredoxin-5, mitochondrial OS=Hom	PRDX5	22.086	73.79	44.9	8	17	8	1.099	1.1	0.924	0.948	0.949	0.964	0.938	0.977	0.85	0.87	0.87
Q7L7X3	Serine/threonine-protein kinase TAO1 OS	TAOK1	116.07	18.256	3.6	3	3	3	0.826	1.2	0.691	1.033	0.911	1.389	0.781	1.372	0.85	1.14	1.06
Q9Y2R9	28S ribosomal protein S7, mitochondrial	MRPS7	28.134	35.289	18.6	4	6	4	1.139	1.04	0.949	0.905	1.087	1.146	0.813	0.97	0.85	1.02	0.82
P30154	Serine/threonine-protein phosphatase 2A	PPP2R1B	66.213	74.505	28.1	14	16	10	1.064	1.06	0.877	0.93	0.989	0.975	1.06	1.046	0.85	0.92	0.99
Q92522	Histone H1x OS=Homo sapiens OX=960	H1FX	22.487	54.3	23	4	7	4	1.072	1.506	1.244	0.949	0.643	0.916	0.881	0.695	0.85	0.60	0.61
P07947	Tyrosine-protein kinase Yes OS=Homo s	YES1	60.801	13.791	9.2	5	6	2	1.12	1.069	0.903	0.959	0.901	1.207	0.799	1.052	0.85	0.96	0.85
O75381	Peroxisomal membrane protein PEX14 O	PEX14	41.236	39.134	15.9	5	7	5	1.073	1.167	0.925	0.98	0.909	0.947	1.005	0.963	0.85	0.83	0.88
Q92828	Coronin-2A OS=Homo sapiens OX=960	CORO2A	59.763	35.127	9.5	5	7	5	1.145	1.067	0.92	0.961	1.013	1.172	0.713	0.923	0.85	0.99	0.74
Q8IXM3	39S ribosomal protein L41, mitochondria	MRPL41	15.383	20.456	21.2	3	4	3	1.072	1.219	0.951	0.997	0.949	1.191	0.707	0.941	0.85	0.93	0.72
Q9BVK2	Probable dolichyl pyrophosphate Glc1Ma	ALG8	60.087	12.04	4.4	2	2	2	1.084	1.058	0.888	0.933	1.017	1.249	0.848	0.974	0.85	1.06	0.85
Q14766	Latent-transforming growth factor beta-bi	LTBP1	186.79	7.1044	0.8	1	1	1	0.961	0.886	0.749	0.821	1.423	1.144	1.085	1.155	0.85	1.39	1.21
Q9UL54	Serine/threonine-protein kinase TAO2 OS	TAOK2	138.25	6.0517	0.8	1	1	1	1.059	1.141	0.86	1.009	0.964	1.01	1.169	0.784	0.85	0.90	0.89
Q9Y6I3	Epsin-1 OS=Homo sapiens OX=9606 GN	EPN1	60.293	86.525	17.4	7	8	7	1.083	1.183	0.985	0.94	0.835	0.919	1.066	1.003	0.85	0.77	0.91
Q86SX6	Glutaredoxin-related protein 5, mitochon	GLRX5	16.628	55.943	28	3	6	3	1.1	1.106	0.966	0.908	0.949	1.023	0.964	0.986	0.85	0.89	0.88
Q15398	Disks large-associated protein 5 OS=Hor	DLGAP5	95.114	82.765	17	12	15	12	1.111	1.01	0.886	0.915	1.032	1.201	0.863	1.096	0.85	1.05	0.92
O60508	Pre-mRNA-processing factor 17 OS=Hor	CDC40	65.521	35.145	9.8	5	5	5	1.113	1.026	0.838	0.978	1.066	0.983	1.106	1.014	0.85	0.96	0.99
Q92908	Transcription factor GATA-6 OS=Homo	GATA6	60.032	10.885	7.2	2	2	2	1.008	1.104	0.826	0.967	1.057	0.876	1.227	1.011	0.85	0.92	1.06
P11279	Lysosome-associated membrane glycopro	LAMP1	44.882	27.678	10.3	4	5	4	1.029	1.137	0.883	0.955	0.89	1.31	0.808	0.987	0.85	1.02	0.83
Q8NBJ4	Golgi membrane protein 1 OS=Homo sap	GOLM1	45.333	28.768	12	4	5	4	1.122	1.123	0.979	0.926	0.99	1.071	0.896	0.955	0.85	0.92	0.82
Q8TAA5	GrpE protein homolog 2, mitochondrial (	GRPEL2	25.431	21.588	12.4	3	4	3	1.053	1.085	0.901	0.913	1.009	1.04	0.992	1.089	0.85	0.96	0.97
Q8TDN6	Ribosome biogenesis protein BRX1 hom	BRIX1	41.401	46.601	22.9	6	10	6	1.029	1.02	0.871	0.867	0.984	1.244	0.975	1.064	0.85	1.09	1.00
P62993	Growth factor receptor-bound protein 2 (	GRB2	25.206	70.245	47	10	15	10	1.108	1.177	0.98	0.958	0.892	0.98	0.958	0.985	0.85	0.82	0.85
Q14137	Ribosome biogenesis protein BOP1 OS=	BOP1	83.629	138.11	18.8	11	16	11	1.046	0.963	0.75	0.953	1.059	0.982	0.99	1.152	0.85	1.02	1.07
Q9BTA9	WW domain-containing adapter protein v	WAC	70.723	42.741	10.8	5	5	5	1.075	1.085	0.873	0.958	1.029	0.915	1.064	1.037	0.85	0.90	0.97
P05387	60S acidic ribosomal protein P2 OS=Hor	RPLP2	11.665	129.75	76.5	6	19	6	1.097	1.152	0.954	0.952	0.924	1.049	0.943	0.977	0.85	0.88	0.85
P78316	Nucleolar protein 14 OS=Homo sapiens (	NOP14	97.667	111.38	14	11	13	11	1	1.084	0.857	0.909	1.011	1.107	0.994	1.104	0.85	1.02	1.01
Q96EB6	NAD-dependent protein deacetylase sirtu	SIRT1	81.68	58.696	10.6	7	7	7	0.996	0.986	0.706	0.973	1.103	1.047	1.175	1.322	0.85	1.08	1.26
Q96S52	GPI transamidase component PIG-S OS=	PIGS	61.655	26.212	8.5	4	4	4	1.034	1.144	0.899	0.946	0.897	1.211	0.923	1.032	0.85	0.97	0.90
Q8N183	NADH dehydrogenase [ubiquinone] 1 alp	NDUFAF2	19.856	57.626	32	5	6	5	1.154	1.081	0.93	0.963	0.934	1.056	0.847	0.94	0.85	0.89	0.80
P39019	40S ribosomal protein S19 OS=Homo sa	RPS19	16.06	80.879	50.3	8	16	7	1.107	1.061	0.893	0.943	1.007	1.011	0.993	1.075	0.85	0.93	0.95
Q13356	RING-type E3 ubiquitin-protein ligase PP	PPIL2	58.823	74.847	24	10	10	10	1.027	1.068	0.86	0.914	1.014	1.214	0.908	1.043	0.85	1.06	0.93
Q2TBE0	CWF19-like protein 2 OS=Homo sapiens	CWF19L2	103.79	47.072	5.6	4	4	4	1.088	1.157	0.989	0.912	1.035	0.97	0.972	0.937	0.85	0.89	0.85
Q13509	Tubulin beta-3 chain OS=Homo sapiens (	TUBB3	50.432	6.885	33.8	14	52	1	1.051	0.892	0.694	0.951	1.186	1.011	0.988	1.295	0.85	1.13	1.17
Q9UBV2	Protein sel-1 homolog 1 OS=Homo sapie	SEL1L	88.754	58.274	12.3	8	8	8	1.008	1.116	0.849	0.949	1	0.929	1.122	1.184	0.85	0.91	1.09
Q96B97	SH3 domain-containing kinase-binding p	SH3KBP1	73.125	32.332	9.9	5	6	5	1.097	1.254	1.024	0.966	0.765	1.126	0.868	0.922	0.85	0.80	0.76
P14854	Cytochrome c oxidase subunit 6B1 OS=H	COX6B1	10.192	59.001	58.1	5	12	5	1.109	1.11	0.989	0.889	1.047	0.996	0.858	0.969	0.85	0.92	0.82
Q9H869	YY1-associated protein 1 OS=Homo sap	YY1AP1	87.943	12.636	4.1	2	2	2	1.053	1.029	1.11	0.652	1.175	0.968	1.348	0.938	0.85	1.03	1.10
Q7L2E3	Putative ATP-dependent RNA helicase D	DHX30	133.94	130.49	17.1	19	24	19	1.088	1.096	0.932	0.915	0.948	1.166	0.799	1.018	0.85	0.97	0.83
Q9H9A6	Leucine-rich repeat-containing protein 40	LRRC40	68.249	98.29	22.8	11	10	10	1.086	1.161	0.952	0.948	0.926	0.905	1.124	0.926	0.85	0.81	0.91
P62899	60S ribosomal protein L31 OS=Homo sa	RPL31	14.463	38.851	39.2	4	8	4	1.08	1.016	0.9	0.872	0.984	1.14	0.924	1.068	0.85	1.01	0.95
Q695T7	Sodium-dependent neutral amino acid tra	SLC6A19	71.109	10.998	4.9	2	2	2	1.203	1.123	0.917	1.049	0.919	1.169	0.762	0.721	0.85	0.90	0.64
Q01650	Large neutral amino acids transporter sma	SLC7A5	55.01	13.574	2.8	2	2	2	1.04	1.07	0.807	0.976	0.958	0.979	0.976	1.257	0.85	0.92	1.06
Q9UHI5	Large neutral amino acids transporter sma	SLC7A8	58.381	18.476	5.6	3	5	3	1.06	1.153	0.929	0.941	0.933	0.89	0.995	0.997	0.85	0.82	0.90
Q9NRR3	CDC42 small effector protein 2 OS=Hom	CDC42SE2	9.2234	26.907	22.6	1	1	1	1.101	1.032	0.894	0.908	0.932	1.286	0.784	1.131	0.84	1.04	0.90
Q9Y606	tRNA pseudouridine synthase A OS=Hom	PUS1	47.47	35.59	13.1	5	5	5	1.102	1.204	1.044	0.904	1.17	0.885	0.945	0.791	0.84	0.89	0.75
P31641	Sodium- and chloride-dependent taurine	SLC6A6	69.829	12.298	4.2	2	2	2	1.095	1.055	0.92	0.896	1.111	0.818	1.17	0.998	0.84	0.90	1.01
Q9C0B7	Transport and Golgi organization protein	TANGO6	120.75	8.1814	1.4	1	1	1	1.043	0.95	0.612	1.071	1.001	1.276	0.706	1.339	0.84	1.14	1.03
Q9NYJ1	Cytochrome c oxidase assembly factor 4	COA4	10.134	20.88	46	3	3	3	1.04	1.107	0.818	0.995	0.934	0.841	1.127	1.181	0.84	0.83	1.07
Q92930	Ras-related protein Rab-8B OS=Homo sa	RAB8B	23.584	-2	16.4	3	7	1	1.127	1.183	1.031	0.919	0.992	0.91	0.998	0.86	0.84	0.82	0.80
O75152	Zinc finger CCCH domain-containing pro	ZC3H11A	89.13	190.29	25.2	16	22	16	1.116	1.087	0.933	0.926	1	1.014	0.954	0.955	0.84	0.91	0.87

Q15785	Mitochondrial import receptor subunit T(	TOMM34	34.559	132.25	31.1	8	14	8	1.046	1.092	0.852	0.952	0.969	1.23	0.913	1.045	0.84	1.03	0.92
Q9BWT3	Poly(A) polymerase gamma OS=Homo sa	PAPOLG	82.802	18.032	6.9	5	7	3	1.172	1.04	0.906	0.96	1.031	1.148	0.832	0.867	0.84	0.99	0.77
P50851	Lipopolysaccharide-responsive and beige	LRBA	319.1	145.39	8.4	21	22	21	1.114	1.179	1.008	0.926	0.868	0.998	0.924	0.939	0.84	0.81	0.81
Q13469	Nuclear factor of activated T-cells, cytopl	NFATC2	100.14	30.789	5.7	5	5	5	1.082	1.118	0.933	0.922	1.061	0.708	1.214	1.015	0.84	0.80	1.01
P05556	Integrin beta-1 OS=Homo sapiens OX=9(	ITGB1	88.414	159.32	22.7	17	32	17	1.067	1.125	0.892	0.956	0.91	0.928	1.058	1.067	0.84	0.84	0.97
P52292	Importin subunit alpha-1 OS=Homo sapi(	KPNA2	57.861	185.45	31	11	28	11	1.139	1.124	0.948	0.959	0.987	0.984	0.87	0.921	0.84	0.87	0.79
P62873	Guanine nucleotide-binding protein G(I)/	GNB1	37.377	126.03	25.6	8	12	4	1.047	1.226	0.922	0.993	0.835	1.07	0.906	1.004	0.84	0.84	0.84
P03928	ATP synthase protein 8 OS=Homo sapier	MT-ATP8	7.9916	5.9955	13.2	1	2	1	1.04	0.818	0.593	0.972	1.234	0.815	1.157	1.43	0.84	1.10	1.39
P49590	Probable histidine--tRNA ligase, mitocho	HARS2	56.888	31.04	17.4	8	14	4	1	1.022	0.805	0.898	1.084	0.957	1.041	1.347	0.84	1.01	1.18
Q9UJC5	SH3 domain-binding glutamic acid-rich-l	SH3BGRL2	12.326	9.3715	15.9	1	1	1	1.018	0.952	0.671	0.988	1.098	0.976	1.018	1.349	0.84	1.05	1.20
Q9NYQ6	Cadherin EGF LAG seven-pass G-type re	CELSR1	329.48	12.123	1.2	2	2	2	1.099	1.479	1.288	0.883	0.483	1.295	0.518	1.067	0.84	0.69	0.61
Q9GZL7	Ribosome biogenesis protein WDR12 OS	WDR12	47.707	94.066	22.5	7	9	7	0.941	1.604	1.143	1	0.634	1.328	0.725	0.664	0.84	0.77	0.55
Q6PCT2	F-box/LRR-repeat protein 19 OS=Homo	FBXL19	75.706	13.128	2.6	2	2	2	1.04	1.061	0.905	0.864	1.097	1.226	0.936	1.009	0.84	1.11	0.93
O15382	Branched-chain-amino-acid aminotransfe	BCAT2	44.287	78.566	20.9	7	8	7	0.986	1.412	0.994	1.025	0.767	0.944	0.971	1	0.84	0.71	0.82
Q9Y320	Thioredoxin-related transmembrane prote	TMX2	34.037	76.523	22.3	6	7	6	1.092	1.175	0.955	0.953	0.947	0.891	1.015	0.92	0.84	0.81	0.85
Q9H6Y2	WD repeat-containing protein 55 OS=Ho	WDR55	42.07	66.328	20.6	7	10	7	1.042	1.186	0.927	0.948	0.956	1.136	0.821	1.056	0.84	0.94	0.84
P08069	Insulin-like growth factor 1 receptor OS=	IGF1R	154.79	20.234	1.7	2	2	2	1.178	1.011	0.978	0.864	0.904	0.931	1.135	1.026	0.84	0.84	0.99
Q8NB49	Phospholipid-transporting ATPase IG OS	ATP11C	129.48	76.53	9.7	9	11	8	1.102	1.104	0.898	0.958	0.974	0.932	1.036	0.996	0.84	0.86	0.92
A1A4S6	Rho GTPase-activating protein 10 OS=H	ARHGAP1	89.374	52.933	9.9	8	10	8	1.138	1.165	0.988	0.949	0.903	0.909	0.966	0.891	0.84	0.79	0.81
P84101	Small EDRK-rich factor 2 OS=Homo sap	SERF2	6.8998	13.82	30.5	2	3	2	1.111	1.028	0.96	0.838	1.283	0.948	1.075	0.938	0.84	1.04	0.94
Q02878	60S ribosomal protein L6 OS=Homo sap	RPL6	32.728	70.612	26.7	9	16	9	1.165	0.967	0.947	0.845	1.064	1.182	0.727	1.127	0.84	1.05	0.87
Q5T280	Putative methyltransferase C9orf114 OS=	SPOUT1	42.008	28.681	11.4	4	6	4	1.029	1.108	0.868	0.928	1.068	1.219	0.877	0.997	0.84	1.07	0.88
Q8WTT2	Nucleolar complex protein 3 homolog OS	NOC3L	92.547	73.817	13.1	10	13	10	1.049	1.059	0.823	0.948	1.03	1.045	1.067	1.018	0.84	0.98	0.99
Q9BRA2	Thioredoxin domain-containing protein 1	TXNDC17	13.941	13.267	18.7	2	4	2	1.005	1.334	0.933	1.032	0.728	0.925	1.073	1.014	0.84	0.71	0.89
Q93063	Exostosin-2 OS=Homo sapiens OX=960(	EXT2	82.254	12.893	3.5	2	2	2	1.082	1.09	0.899	0.925	1.004	0.834	1.144	1.077	0.84	0.85	1.02
Q53R41	FAST kinase domain-containing protein	FASTKD1	97.41	21.478	4.3	3	3	3	1.083	1.113	0.874	0.97	1.129	1.253	0.696	0.903	0.84	1.08	0.73
Q13636	Ras-related protein Rab-31 OS=Homo sa	RAB31	21.569	46.141	25.3	4	6	4	1.046	1.042	0.827	0.926	1.067	1.074	1.002	1.138	0.84	1.03	1.02
P63220	40S ribosomal protein S21 OS=Homo sa	RPS21	9.1113	21.653	39.8	3	8	3	1.04	1.124	0.901	0.915	0.962	1.258	0.813	1.044	0.84	1.03	0.86
Q14244	Ensconsin OS=Homo sapiens OX=9606	MAP7	84.051	55.136	11.6	9	11	9	1.058	1.118	0.885	0.941	1.095	1.016	0.977	0.996	0.84	0.97	0.91
P0CG12	Chromosome transmission fidelity protei	CHTF8	51.391	23.782	9.2	3	3	3	1.12	1.067	0.942	0.893	1.003	1.072	1.085	1.107	0.84	0.95	1.00
Q12974	Protein tyrosine phosphatase type IVA 2	PTP4A2	19.127	7.4754	7.8	1	1	1	1.076	1.367	1.067	0.982	0.746	1.2	0.769	0.82	0.84	0.80	0.65
Q15059	Bromodomain-containing protein 3 OS=I	BRD3	79.541	30.727	14	10	14	5	1.035	1.152	0.868	0.966	1	1.051	0.924	1.078	0.84	0.94	0.92
Q9BR76	Coronin-1B OS=Homo sapiens OX=960(	CORO1B	54.234	39.99	11.9	6	8	6	1.094	0.969	0.823	0.907	1.015	1.038	1.004	0.976	0.84	1.00	0.96
Q9NPB8	Glycerophosphocholine phosphodiesteras	GPCPD1	76.034	6.9465	1.2	1	1	1	1.119	1.105	0.977	0.888	1.042	0.913	0.909	1.109	0.84	0.88	0.91
O15235	28S ribosomal protein S12, mitochondria	MRPS12	15.173	26.663	31.9	3	4	3	1.028	1.165	0.89	0.949	0.894	1.124	0.863	1.073	0.84	0.92	0.88
P35580	Myosin-10 OS=Homo sapiens OX=9606	MYH10	229	323.31	35.9	65	101	47	1.112	1.099	0.912	0.942	0.917	0.961	1.045	1.045	0.84	0.85	0.95
O75298	Reticulon-2 OS=Homo sapiens OX=960(	RTN2	59.263	25.847	7.9	4	5	4	1.058	1.351	1.077	0.943	0.789	1.087	0.803	0.866	0.84	0.78	0.69
Q9NTJ4	Alpha-mannosidase 2C1 OS=Homo sapie	MAN2C1	115.83	7.4781	1.2	1	2	1	0.917	1.051	0.694	0.956	0.938	0.943	1.289	1.387	0.84	0.96	1.36
O96015	Dynein light chain 4, axonemal OS=Hom	DNAL4	12.009	6.1925	7.6	1	1	1	1.142	1.123	0.932	0.967	0.945	0.99	0.981	0.894	0.84	0.85	0.83
Q8NDD1	Uncharacterized protein C1orf131 OS=H	C1orf131	32.61	25.766	15	4	3	3	1.095	1.114	0.967	0.885	1.02	1.244	0.808	0.962	0.84	1.02	0.80
P30046	D-dopachrome decarboxylase OS=Homo	DDT	12.712	61.458	43.2	5	12	5	1.056	1.169	0.915	0.948	0.979	1.063	0.963	0.97	0.84	0.92	0.87
Q9Y5B8	Nucleoside diphosphate kinase 7 OS=Ho	NME7	42.491	13.495	5.9	2	2	2	1.065	1.116	0.905	0.921	0.896	1.275	0.92	0.98	0.84	1.00	0.87
Q32MZ4	Leucine-rich repeat flightless-interacting	LRRFIP1	89.252	323.31	27.5	19	27	18	1.12	1.31	1.025	1.009	0.701	0.883	1.04	0.97	0.84	0.65	0.83
O95777	U6 snRNA-associated Sm-like protein LS	LSM8	10.403	13.45	19.8	2	2	2	1.032	1.195	0.932	0.932	1.023	1.357	0.713	0.912	0.84	1.07	0.73
Q96KM6	Zinc finger protein 512B OS=Homo sapi	ZNF512B	97.263	41.122	7.1	5	5	5	0.982	1.035	0.798	0.89	1.056	1.019	1.01	1.387	0.84	1.03	1.19
Q15417	Calponin-3 OS=Homo sapiens OX=9606	CNN3	36.413	71.739	39.2	9	13	7	1.072	1.184	0.92	0.968	0.903	1.033	0.965	0.969	0.84	0.86	0.86
P59998	Actin-related protein 2/3 complex subuni	ARPC4	19.667	22.972	17.9	3	6	3	1.13	1.172	1.006	0.92	1.023	1.006	0.948	0.925	0.84	0.88	0.81
P42892	Endothelin-converting enzyme 1 OS=Hor	ECE1	87.163	27.888	5.7	4	4	4	0.998	1.083	0.788	0.953	1.019	0.978	1.073	1.217	0.84	0.96	1.10
O43920	NADH dehydrogenase [ubiquinone] iron-	NDUFS5	12.517	25.27	34.9	3	5	3	1.026	1.011	0.737	0.967	0.96	0.957	1.092	1.327	0.84	0.94	1.19
P30048	Thioredoxin-dependent peroxide reductas	PRDX3	27.692	67.122	35.9	7	21	6	1.099	1.158	0.944	0.944	0.916	1.02	0.978	1.003	0.84	0.86	0.88
O00767	Acyl-CoA desaturase OS=Homo sapiens	SCD	41.522	54.362	10.6	2	3	2	1.173	1.139	1.051	0.883	1.044	1.01	0.926	0.872	0.84	0.89	0.78
P53794	Sodium/myo-inositol cotransporter OS=I	SLC5A3	79.693	10.939	2.8	2	2	2	1.038	0.919	0.747	0.89	1.016	1.23	0.851	1.449	0.84	1.15	1.18
Q6EEV4	DNA-directed RNA polymerase II subuni	POLR2M	15.131	6.0474	17.6	1	1	1	1.084	0.989	0.915	0.819	1.123	0.907	1.246	1.046	0.84	0.98	1.11
Q6IC98	GRAM domain-containing protein 4 OS=	GRAMD4	66.407	25.907	6.2	4	5	4	1.119	1.116	0.893	0.976	0.986	1.002	0.886	1.015	0.84	0.89	0.85
P30101	Protein disulfide-isomerase A3 OS=Hom	PDIA3	56.782	323.31	61	28	77	27	1.076	1.254	0.978	0.97	0.863	1.001	0.941	0.932	0.84	0.80	0.80
P16949	Stathmin OS=Homo sapiens OX=9606 G	STMN1	17.302	73.661	51.7	10	21	9	1.033	1.078	0.779	0.985	0.971	0.964	1.033	1.239	0.84	0.92	1.08
P81877	Single-stranded DNA-binding protein 2 (	SSBP2	37.827	7.1849	2.8	1	1	1	1.131	1.153	0.925	0.983	0.927	0.903	1.007	0.945	0.84	0.80	0.85
Q9UMY4	Sorting nexin-12 OS=Homo sapiens OX=	SNX12	19.73	41.853	29.7	6	15	5	1.106	1.123	0.919	0.943	0.976	1.027	0.906	1.008	0.84	0.90	0.86
P23142	Fibulin-1 OS=Homo sapiens OX=9606 (	FBLN1	77.213	44.475	8.4	5	5	4	1.081	1.073	0.88	0.919	1.016	0.998	1.081	1.145	0.84	0.94	1.03
O94973	AP-2 complex subunit alpha-2 OS=Hom(	AP2A2	103.96	77.123	20.9	15	24	9	1.083	1.186	0.935	0.96	0.883	0.952	1.043	1.028	0.84	0.81	0.91
Q69YH5	Cell division cycle-associated protein 2 (	CDCA2	112.68	23.65	1.6	1	1	1	1.024	1.36	1.026	0.965	0.889	1.268	0.659	0.891	0.84	0.90	0.65
Q9Y5X2	Sorting nexin-8 OS=Homo sapiens OX=9	SNX8	52.569	15.898	5.8	2	6	2	1.009	0.985	0.694	0.971	0.905	0.972	1.116	1.447	0.84	0.94	1.29
O75127	Pentatricopeptide repeat-containing prote	PTCD1	78.855	7.3528	1.4	1	1	1	1.113	1.31	1.061	0.962	0.927	1.01	0.822	0.799	0.83	0.80	0.67
Q9NXW2	DnaJ homolog subfamily B member 12 O	DNAJB12	41.818	31.767	15.7	5	6	5	1.121	1.011	0.885	0.895	1.018	1.083	1.021	1.013	0.83	0.99	0.95
Q9BYD1	39S ribosomal protein L13, mitochondria	MRPL13	20.692	34.422	32.6	5	6	5	1.066	1.204	0.932	0.963	1.023	1.207	0.768	0.934	0.83	0.98	0.75
Q9UEE9	Craniofacial development protein 1 OS=I	CFDP1	33.592	29.842	16.4	4	5	4	1.097	1.206	0.975	0.947	0.858	0.957	1.023	0.968	0.83	0.79	0.86
Q12931	Heat shock protein 75 kDa, mitochondria	TRAP1	80.109	140.55	28	16	21	15	1.105	1.092	0.902	0.931	1.004	1.199	0.933	1.006	0.83	1.00	0.88
Q96T88	E3 ubiquitin-protein ligase UHRF1 OS=I	UHRF1	89.813	11.128	3.9	3	3	2	1.17	1.037	0.935	0.906	0.903	0.992	1.03	1.034	0.83	0.86	0.94
Q7Z3C6	Autophagy-related protein 9A OS=Homo	ATG9A	94.446	30.868	6	5	6	5	1.119	1.079	0.936	0.897	1.019	1.142	0.946	0.909	0.83	0.98	0.84
O75037	Kinesin-like protein KIF21B OS=Homo s	KIF21B	182.66	-2	2	3	3	1	1.087	1.321	1.076	0.932	0.845	1.182	0.835	0.77	0.83	0.84	0.67
P04632	Calpain small subunit 1 OS=Homo sapie	CAPNS1	28.315	27.693	14.2	3	6	3	1.089	1.243	0.987	0.957	0.755	0.85	1.069	1.006	0.83	0.69	0.89
Q9HD45	Transmembrane 9 superfamily member 3	TM9SF3	67.887	48.531	11.4	6	12	5	1.157	1.029	0.915	0.907	0.941	0.965	1.066	0.949	0.83	0.87	0.92
Q14008	Cytoskeleton-associated protein 5 OS=H	CKAP5	225.49	323.31	24.6	45	62	45	1.111	1.139	0.936	0.939	0.939	1.003	0.935	0.978	0.83	0.86	0.85
M5A8F1	Suppressyn OS=Homo sapiens OX=9606	ERVH48-1	18.128	7.9153	9.4	1	1	1	1.114	1.082	0.862	0.968	0.966	1.3	0.683	1.036	0.83	1.03	0.78
O60749	Sorting nexin-2 OS=Homo sapiens OX=9	SNX2	58.47	155.51	42.2	18	36	16	1.117	1.138	0.937	0.942	0.908	1.003	0.953	0.98	0.83	0.85	0.86
Q8TEW0	Partitioning defective 3 homolog OS=Ho	PARD3	151.42	106.63	12.2	12	14	12	1.09	1.206	0.934	0.979	0.799	0.924	1.033	0.949	0.83	0.75	0.86
Q96BK5	PIN2/TERF1-interacting telomerase inhil	PINX1	37.034	22.525	14.9	3	3	3	1.179	0.701	0.653	0.913	1.227	0.848	1.122	1.349	0.83	1.10	1.31
Q16831	Uridine phosphorylase 1 OS=Homo sapie	UPP1	33.934	42.933	19.7	6	7	6	1.013	1.016	0.713	0.977	0.919	0.842	1.286	1.334	0.83	0.87	1.29
Q9UG56	Phosphatidylserine decarboxylase proenz	PISD	46.671	18.165	6.6	3	4	3	1.134	0.857	0.761	0.897	1.208	0.887	1.296	1.21	0.83	1.05	1.26
P17900	Ganglioside GM2 activator OS=Homo sa	GM2A	20.838	79.096	31.1	5	9	5	1.101	1.195	0.943	0.967	0.946	1.19	0.73	0.848	0.83	0.93	0.69
Q15031	Probable leucine--tRNA ligase, mitochon	LARS2	101.97	61.229	12.3	9	9	9	1.113	1.098	0.884	0.955	0.953	1.266	0.747	0.95	0.83	1.00	0.77
O43314	Inositol hexakisphosphate and diphosphc	PPIP5K2	140.41	58.408	6.8	8	9	8	1.058	1.002	0.763	0.95	1.085	0.881	1.129	1.291	0.83	0.95	1.17
P36542	ATP synthase subunit gamma, mitochond	ATP5F1C	32.996	76.864	28.2	9	18	9	1.096	1.105	0.878	0.951	0.998	1.204	0.854	1.045	0.83	1.00	0.86
Q9C091	GREB1-like protein OS=Homo sapiens (	GREB1L	214.35	160.62	11.5	18	21	18	1.077	1.186	0.954	0.926	0.932	0.993	1.005	0.932	0.83	0.85	0.86
Q6UB35	Monofunctional C1-tetrahydrofolate synt	MTHFD1L	105.79	119.7	15	12	16	12	1.06	1.132	0.873	0.947	0.949	1.059	0.959	1.071	0.83	0.92	0.93
Q15018	BRISC complex subunit Abraxas 2 OS=I	ABRAXAS	46.9	30.993	14.5	5	5	5	1.042	1.133	0.943	0.862	0.935	1.064	1.139	1.026	0.83	0.92	1.00
Q9Y5J1	U3 small nucleolar RNA-associated prote	UTP18	62.003	75.272	23.4	9	12	9	1.068	1.142	0.894	0.94	0.937	1.176	0.865	0.96	0.83	0.96	0.83
Q9ULG6	Cell cycle progression protein 1 OS=Hon	CCPG1	87.339	5.9817	1.3	1	1	1	1.07	0.969	0.844	0.848	1.122	1.057	1.173	1.036	0.83	1.07	1.08
Q15120	[Pyruvate dehydrogenase (acetyl-transfer	PDK3	46.938	11.592	4.9	2	2	2	1.035	1.167	0.887	0.94	0.989	0.984	1.132	0.943	0.83	0.90	0.94
A6NJ78	Probable methyltransferase-like protein 1	METTL15	46.121	7.3625	3.4	1	1	1	1.034	1.115	0.828	0.955	0.953	1.127	0.954	1.112	0.83	0.97	0.96
O15260	Surfeit locus protein 4 OS=Homo sapiens	SURF4	30.394	33.301	14.9	4	8	4	1.107	1.124	0.976	0.875	0.99	0.936	1.116	0.92	0.83	0.86	0.91
P29372	DNA-3-methyladenine glycosylase OS=I	MPG	32.868	13.631	8.1	2	2	2	1.126	1.367	0.976	1.092	0.674	1.218	0.727	0.719	0.83	0.76	0.58
Q29865	HLA class I histocompatibility antigen, C	HLA-C	40.932	120.9	34.7	9	12	1	1.091	1.11	0.884	0.94	0.954	1.176	0.881	1.008	0.83	0.97	0.86
A3KMH1	von Willebrand factor A domain-containi	VWA8	214.82	68.49	5.7	10	10	10	1.119	1.08	0.887	0.935	0.947	0.869	1	1.058	0.83	0.83	0.94
P51795	H(+)/Cl(-) exchange transporter 5 OS=H(	CLCN5	83.146	13.663	2.5	2	2	2	1.125	1.055	0.83	0.976	0.94	0.967	0.956	1.147	0.83	0.87	0.96
Q8N128	Protein FAM177A1 OS=Homo sapiens (	FAM177A1	23.757	37.86	34.7	5	5	4	1.074	1.138	0.882	0.95	0.881	1.205	0.887	1.023	0.83	0.94	0.86
Q0JRZ9	F-BAR domain only protein 2 OS=Homo	FCHO2	88.923	55.187	10.1	7	7	7	1.147	1.094	0.968	0.888	0.971	0.907	0.984	0.994	0.83	0.84	0.88
Q13601	KRR1 small subunit processome compon	KRR1	43.664	41.247	10.8	4	6	4	1.036	0.972	0.756	0.907	1.049	1.047	1.025	1.243	0.83	1.04	1.13
Q9UGN5	Poly [ADP-ribose] polymerase 2 OS=Ho	PARP2	66.205	27.114	7	4	4	4	0.944	0.956	0.664	0.909	1.131	1.055	1.178	1.348	0.83	1.15	1.33
P26599	Polypyrimidine tract-binding protein 1 O	PTBP1	57.221	121.01	22.6	8	14	7	1.08	1.382	1.06	0.978	0.708	1.222	0.77	0.881	0.83	0.78	0.67
Q9HAW4	Claspin OS=Homo sapiens OX=9606 GN	CLSPN	151.09	19.276	2.5	3	3	3	0.916	0.97	0.64	0.921	1.125	1.03	1.26	1.333	0.83	1.14	1.37
Q13137	Calcium-binding and coiled-coil domain-	CALCOCO	52.253	30.006	7.2	3	3	3	1.026	1.058	0.783	0.941	1.007	1.065	1.013	1.206	0.83	0.99	1.06
Q9H981	Actin-related protein 8 OS=Homo sapien	ACTR8	70.483	33.244	8.3	5	5	5	1.003	0.98	0.697	0.943	1.195	1.181	0.915	1.195	0.83	1.20	1.06
P14859	POU domain, class 2, transcription factor	POU2F1	76.471	123.6	12.1	6	8	6	1.15	1.152	0.912	0.991	0.978	1.041	0.81	0.945	0.83	0.88	0.76
P62854	40S ribosomal protein S26 OS=Homo sa	RPS26	13.015	46.081	31.3	3	9	3	1.083	1.01	0.812	0.917	1.003	1.035	1.036	0.993	0.83	0.97	0.97
P27797	Calreticulin OS=Homo sapiens OX=960(	CALR	48.141	164.29	53.7	16	35	16	1.116	1.199	0.959	0.952	0.898	0.999	0.938	1.005	0.83	0.82	0.84
Q9Y5Y5	Peroxisomal membrane protein PEX16 O	PEX16	38.628	9.8225	3.9	1	1	1	0.835	1.537	0.955	1.003	0.69	1.369	0.801	1.023	0.83	0.87	0.77
Q8NBJ7	Inactive C-alpha-formylglycine-generatin	SUMF2	33.843	50.966	23.6	7	12	7	1.142	1.074	0.898	0.93	0.987	0.983	1.05	1.054	0.82	0.89	0.95
Q66K79	Carboxypeptidase Z OS=Homo sapiens (	CPZ	73.655	26.731	6.9	4	4	4	1.068	1.141	0.933	0.889	1.016	1.17	0.782	1.109	0.82	0.99	0.86
Q9NWR8	Calcium uniporter regulatory subunit MC	MCUB	39.082	6.4629	3	1	1	1	1.094	1.111	0.937	0.881	1.01	1.065	0.93	1.059	0.82	0.94	0.90
Q96HD1	Cysteine-rich with EGF-like domain prot	CRELD1	45.439	38.496	17.9	4	5	4	1.075	1.208	0.93	0.952	0.949	1.047	0.953	1.002	0.82	0.87	0.86
Q96EU7	C1GALT1-specific chaperone 1 OS=Hon	C1GALT1C	36.382	86.542	20.8	5	7	5	1.075	1.242	0.972	0.938	0.936	0.988	0.954	0.898	0.82	0.83	0.80
A0PJW6	Transmembrane protein 223 OS=Homo s	TMEM223	22.049	13.656	9.9	2	2	2	1.034	1.094	0.851	0.903	1.03	0.946	1.104	1.155	0.82	0.93	1.06
P32519	ETS-related transcription factor Elf-1 OS	ELF1	67.497	17.396	5.3	2	2	2	0.969	1.028	0.66	0.986	1.072	1.215	0.842	1.345	0.82	1.15	1.10
Q9Y3U8	60S ribosomal protein L36 OS=Homo sa	RPL36	12.254	34.836	30.5	5	9	5	1.088	1.051	0.919	0.843	1.077	0.945	1.013	1.114	0.82	0.95	0.99
Q9NYZ3	G2 and S phase-expressed protein 1 OS=	GTSE1	76.644	38.971	9.3	5	5	5	1.026	0.745	0.62	0.838	1.36	1.125	1.057	1.402	0.82	1.40	1.39
Q16643	Drebrin OS=Homo sapiens OX=9606 GN	DBN1	71.428	230.56	31	13	19	13	1.044	1.315	0.945	0.997	0.753	0.893	1.108	1.061	0.82	0.70	0.92
Q04917	14-3-3 protein eta OS=Homo sapiens OX	YWHAH	28.218	84.39	42.3	11	36	9	1.135	1.071	0.852	0.964	0.908	0.837	1.096	1.05	0.82	0.79	0.97
P62312	U6 snRNA-associated Sm-like protein LS	LSM6	9.1275	19.875	35	3	7	3	1.043	1.355	0.981	0.993	0.749	1.194	0.836	0.961	0.82	0.81	0.75
Q96IR7	4-hydroxyphenylpyruvate dioxygenase-lil	HPDL	39.385	79.845	25.1	6	8	6	1.005	1.11	0.778	0.963	1.02	1.33	0.837	1.091	0.82	1.11	0.91
Q99757	Thioredoxin, mitochondrial OS=Homo sa	TXN2	18.383	31.08	9	1	2	1	1.065	1.117	0.837	0.959	0.964	1.275	0.793	1.044	0.82	1.03	0.84
Q8NHQ8	Ras association domain-containing protei	RASSF8	48.326	64.902	17.4	5	5	5	1.097	0.95	0.715	0.969	0.949	0.999	1.015	1.184	0.82	0.95	1.07
Q9Y399	28S ribosomal protein S2, mitochondrial	MRPS2	33.249	25.578	12.5	4	4	4	0.99	1.44	1.114	0.885	0.731	1.424	0.701	0.891	0.82	0.89	0.66
P04040	Catalase OS=Homo sapiens OX=9606 G	CAT	59.755	160.14	36.4	16	20	16	1.155	1.179	0.985	0.935	0.874	0.948	0.947	0.904	0.82	0.78	0.79
Q9H4H8	Protein FAM83D OS=Homo sapiens OX	FAM83D	64.424	47.037	11.6	5	6	5	1.105	1.352	1.039	0.982	0.873	1.148	0.802	0.794	0.82	0.82	0.65
Q15181	Inorganic pyrophosphatase OS=Homo sa	PPA1	32.66	186.31	56.4	13	34	13	1.12	1.177	0.937	0.952	0.898	0.97	0.949	0.943	0.82	0.81	0.82
Q5JTJ3	Cytochrome c oxidase assembly factor 6	COA6	14.116	13.316	21.6	2	3	2	1.145	0.97	0.911	0.828	1.099	1.012	1.075	1.04	0.82	1.00	1.00
Q86WW8	Cytochrome c oxidase assembly factor 5	COA5	8.3755	15.646	35.1	2	2	2	1.076	1.087	0.818	0.96	0.969	1.09	0.928	1.115	0.82	0.95	0.94
P11940	Polyadenylate-binding protein 1 OS=Hor	PABPC1	70.67	217.1	35.7	21	41	12	1.131	1.126	0.961	0.894	0.959	1.184	0.718	0.888	0.82	0.95	0.71
Q5BJF2	Sigma intracellular receptor 2 OS=Homo	TMEM97	20.848	15.81	14.2	2	2	2	1.093	1.057	0.928	0.839	1.074	1.042	1.09	0.987	0.82	0.98	0.97
Q9BUZ4	TNF receptor-associated factor 4 OS=Ho	TRAF4	53.542	22.837	8.5	3	3	3	1.134	1.165	1.001	0.888	0.988	1.294	0.75	0.823	0.82	0.99	0.68
O00483	Cytochrome c oxidase subunit NDUFA4	NDUFA4	9.3697	27.33	46.9	4	8	4	1.256	1.107	1.02	0.921	0.981	0.912	0.88	0.812	0.82	0.80	0.72
P23526	Adenosylhomocysteinase OS=Homo sapi	AHCY	47.716	155.87	44.2	18	34	18	1.058	1.282	0.955	0.967	0.921	1.188	0.722	0.958	0.82	0.90	0.72
Q9P2D3	HEAT repeat-containing protein 5B OS=	HEATR5B	224.3	36.548	2.8	5	5	5	1.15	1.062	0.957	0.859	0.975	0.964	1.064	1.024	0.82	0.88	0.94
Q13464	Rho-associated protein kinase 1 OS=Hor	ROCK1	158.17	120.05	16	20	22	16	1.023	1.361	0.967	0.99	0.762	0.917	0.972	0.989	0.82	0.70	0.82
Q10472	Polypeptide N-acetylgalactosaminyltrans	GALNT1	64.218	10.009	4.1	2	1	1	0.963	1.034	0.649	0.989	1.048	1.024	1.069	1.337	0.82	1.04	1.20
Q9UJQ4	Sal-like protein 4 OS=Homo sapiens OX	SALL4	112.23	49.975	7	6	6	6	1.184	1.152	0.992	0.923	0.943	0.766	1.122	0.891	0.82	0.73	0.86
Q8IUR0	Trafficking protein particle complex subu	TRAPPC5	20.783	11.704	9.6	2	2	2	1.11	1.019	0.863	0.882	1.056	1.197	0.919	1.023	0.82	1.06	0.91
P12235	ADP/ATP translocase 1 OS=Homo sapie	SLC25A4	33.064	96.553	35.2	11	22	3	1.094	1.122	0.896	0.92	0.963	1.109	0.897	0.925	0.82	0.94	0.82
P30084	Enoyl-CoA hydratase, mitochondrial OS=	ECHS1	31.387	50.29	21	6	8	5	1.106	1.196	0.976	0.91	0.975	0.968	0.906	0.893	0.82	0.84	0.78
Q9BUI4	DNA-directed RNA polymerase III subun	POLR3C	60.611	14.104	3.4	2	2	2	1.13	1.087	0.897	0.919	1.012	1.267	0.797	0.916	0.82	1.03	0.77
Q96PY5	Formin-like protein 2 OS=Homo sapiens	FMNL2	123.32	51.717	8	8	9	8	1.136	1.135	0.908	0.952	0.879	0.978	1.03	1.052	0.82	0.82	0.92
Q7KZN9	Cytochrome c oxidase assembly protein (	COX15	46.03	6.0037	2	1	2	1	1.071	1.054	0.833	0.907	0.99	1.047	1.021	1.162	0.82	0.96	1.03
Q99828	Calcium and integrin-binding protein 1 C	CIB1	21.703	6.8391	7.9	1	1	1	0.849	0.944	0.594	0.874	1.477	0.877	1.389	1.264	0.82	1.31	1.48
Q8IYB7	DIS3-like exonuclease 2 OS=Homo sapie	DIS3L2	99.278	17.053	3.5	3	3	3	1.125	1.13	0.92	0.926	0.966	1.117	0.8	1.049	0.82	0.92	0.82
P23919	Thymidylate kinase OS=Homo sapiens O	DTYMK	23.819	70.442	46.2	10	16	10	1.046	1.296	0.957	0.96	0.767	1.138	0.919	0.951	0.82	0.81	0.80
Q96I99	Succinate--CoA ligase [GDP-forming] su	SUCLG2	46.51	72.53	23.1	10	14	10	1.093	1.139	0.893	0.933	0.939	1.013	1.044	1.033	0.82	0.87	0.93
Q12800	Alpha-globin transcription factor CP2 OS	TFCP2	57.255	85.575	12.2	4	7	3	1.088	1.142	0.849	0.974	0.97	1.154	0.798	1.039	0.82	0.95	0.82
Q16706	Alpha-mannosidase 2 OS=Homo sapiens	MAN2A1	131.14	130.15	14.6	14	17	14	1.07	1.132	0.892	0.907	0.975	1.227	0.941	0.996	0.82	1.00	0.88
Q8NEF9	Serum response factor-binding protein 1	SRFBP1	48.633	39.389	15.6	6	8	6	1.084	0.94	0.728	0.925	1.053	1.051	1.141	1.154	0.82	1.04	1.13
P54886	Delta-1-pyrroline-5-carboxylate synthase	ALDH18A1	87.301	177.8	24	15	21	15	1.096	1.094	0.874	0.914	0.948	0.966	0.953	1.047	0.82	0.87	0.91
P18859	ATP synthase-coupling factor 6, mitocho	ATP5J	12.587	19.046	26.9	3	4	2	1.139	1.1	0.875	0.953	1.017	1.237	0.697	0.966	0.82	1.01	0.74
Q92556	Engulfment and cell motility protein 1 OS	ELMO1	83.829	53.972	14	9	10	9	1.179	1.007	0.888	0.896	1.029	0.927	1.172	0.899	0.82	0.89	0.95
Q5UCC4	ER membrane protein complex subunit 1(	EMC10	27.347	15.194	9.5	2	2	2	1.128	1.21	0.96	0.948	0.823	1.085	0.938	0.917	0.82	0.82	0.79
P63173	60S ribosomal protein L38 OS=Homo sa	RPL38	8.2178	16.443	28.6	2	5	2	1.166	1.111	0.924	0.934	0.941	1.185	0.764	0.966	0.82	0.93	0.76
P18206	Vinculin OS=Homo sapiens OX=9606 G	VCL	123.8	323.31	44	42	74	42	1.1	1.166	0.913	0.936	0.868	0.908	1.059	1.063	0.82	0.78	0.94

O15162	Phospholipid scramblase 1 OS=Homo sa	PLSCR1	35.049	31.66	9.4	2	2	2	1.041	1.159	0.894	0.901	1.004	1.001	1.018	1.096	0.82	0.91	0.96
P05388	60S acidic ribosomal protein P0 OS=Hor	RPLP0	34.273	77.915	24.3	6	20	6	1.104	1.16	0.907	0.939	0.954	1	0.944	0.987	0.82	0.86	0.85
P37802	Transgelin-2 OS=Homo sapiens OX=960	TAGLN2	22.391	100.83	56.3	11	26	11	1.147	1.301	1.012	0.983	0.737	0.806	1.03	0.935	0.81	0.63	0.80
P38435	Vitamin K-dependent gamma-carboxylase	GGCX	87.56	29.848	6.3	4	6	4	1.111	1.167	0.903	0.953	0.98	1.218	0.72	0.959	0.81	0.96	0.74
Q15058	Kinesin-like protein KIF14 OS=Homo sa	KIF14	186.49	154.94	14.6	23	25	22	1.123	1.278	0.99	0.966	0.911	1.034	0.857	0.875	0.81	0.81	0.72
Q6UWP7	Lysocardiolipin acyltransferase 1 OS=Ho	LCLAT1	48.92	25.567	10.1	3	4	3	1.139	1.212	0.934	0.981	0.823	0.925	1.002	0.959	0.81	0.74	0.83
Q9Y2Q5	Ragulator complex protein LAMTOR2 O	LAMTOR2	13.507	26.598	24.8	3	4	3	1.153	1.154	0.923	0.955	0.857	0.946	0.96	1.002	0.81	0.78	0.85
Q9UNW1	Multiple inositol polyphosphate phospha	MINPP1	55.051	50.342	12.5	5	7	5	1.066	1.248	0.928	0.955	0.911	1.207	0.921	1.031	0.81	0.92	0.84
Q9NPD8	Ubiquitin-conjugating enzyme E2 T OS=	UBE2T	22.521	48.534	33	5	8	5	0.973	0.98	0.713	0.876	1.108	1.033	1.043	1.341	0.81	1.10	1.22
Q7Z624	Calmodulin-lysine N-methyltransferase C	CAMKMT	36.128	9.4075	4	1	1	1	1.149	1.359	1.047	0.992	0.707	0.89	1.019	0.791	0.81	0.64	0.72
Q9Y616	Interleukin-1 receptor-associated kinase 3	IRAK3	67.766	-2	1.3	1	1	1	1.129	1.248	0.946	0.986	0.881	0.982	0.905	0.9	0.81	0.78	0.76
P61966	AP-1 complex subunit sigma-1A OS=Ho	AP1S1	18.733	19.363	16.5	3	3	3	1.139	1.147	0.913	0.944	0.997	1.016	0.858	0.988	0.81	0.88	0.81
Q969S3	Zinc finger protein 622 OS=Homo sapier	ZNF622	54.271	73.571	18.4	6	8	6	1.025	1.092	0.815	0.903	1.01	1.068	0.993	1.292	0.81	0.98	1.08
Q9UJX6	Anaphase-promoting complex subunit 2 (	ANAPC2	93.827	24.155	4.3	4	4	4	1.006	1.418	0.998	0.969	0.724	1.24	0.75	0.977	0.81	0.81	0.71
Q9Y605	MORF4 family-associated protein 1 OS=	MRFAP1	14.649	51.599	26.8	2	2	2	0.942	1.707	1.224	0.925	0.561	1.473	0.626	0.728	0.81	0.77	0.51
Q5SW79	Centrosomal protein of 170 kDa OS=Hor	CEP170	175.29	12.536	1.9	3	3	2	1.138	1.177	0.933	0.945	0.929	1.006	0.881	0.994	0.81	0.84	0.81
P38159	RNA-binding motif protein, X chromosor	RBMX	42.331	153.33	36.6	15	41	4	1.137	1.13	0.905	0.934	0.959	1.181	0.888	0.946	0.81	0.94	0.81
P61353	60S ribosomal protein L27 OS=Homo sa	RPL27	15.798	72.571	44.9	6	15	6	1.068	1.092	0.918	0.834	1.062	1.061	1.061	1.043	0.81	0.98	0.97
Q99798	Aconitate hydratase, mitochondrial OS=H	ACO2	85.424	313.53	37.1	24	47	24	1.101	1.206	0.941	0.93	0.959	1.159	0.827	0.95	0.81	0.92	0.77
Q9NVU7	Protein SDA1 homolog OS=Homo sapier	SDAD1	79.87	26.049	6.3	4	4	4	1.099	0.89	0.707	0.906	1.224	0.772	1.164	1.296	0.81	1.00	1.24
P35237	Serpin B6 OS=Homo sapiens OX=9606 (	SERPINB6	42.621	12.723	4	1	2	1	0.995	1.045	0.74	0.914	0.946	1.205	0.947	1.362	0.81	1.05	1.13
Q9H3N1	Thioredoxin-related transmembrane prote	TMX1	31.791	43.409	16.1	5	10	5	1.083	1.24	0.935	0.947	0.887	1.016	1.081	0.924	0.81	0.82	0.86
Q13823	Nucleolar GTP-binding protein 2 OS=Ho	GNL2	83.654	50.178	9.3	6	9	6	1.078	1.143	0.913	0.886	0.937	1.074	1.051	0.951	0.81	0.91	0.90
P24588	A-kinase anchor protein 5 OS=Homo sap	AKAP5	47.088	7.6458	3.7	1	1	1	1.113	1.129	0.905	0.91	0.949	0.961	0.999	1.085	0.81	0.85	0.93
P09543	2',3'-cyclic-nucleotide 3'-phosphodiestera	CNP	47.578	46.33	16.6	6	7	6	1.086	1.13	0.822	0.971	0.949	0.919	0.954	1.2	0.81	0.84	0.97
P55327	Tumor protein D52 OS=Homo sapiens O	TPD52	24.327	47.489	37.5	5	8	5	1.025	1.392	0.975	0.98	0.748	0.904	1.093	0.97	0.81	0.68	0.85
P05067	Amyloid-beta A4 protein OS=Homo sapi	APP	86.942	42.445	9.1	6	7	6	1.008	0.906	0.674	0.873	1.186	1.169	0.958	1.291	0.81	1.23	1.18
O00264	Membrane-associated progesterone recep	PGRMC1	21.671	58.592	25.1	7	11	6	1.209	1.169	0.968	0.954	0.916	1.181	0.73	0.896	0.81	0.88	0.68
Q9BQG0	Myb-binding protein 1A OS=Homo sapie	MYBBP1A	148.85	219.6	19.8	21	28	20	0.994	1.116	0.794	0.911	1.015	1.116	0.918	1.166	0.81	1.01	0.99
Q9Y6G3	39S ribosomal protein L42, mitochondria	MRPL42	16.661	17.463	10.6	1	1	1	0.978	1.095	0.683	0.992	0.981	1.039	0.989	1.348	0.81	0.97	1.13
Q71UM5	40S ribosomal protein S27-like OS=Hom	RPS27L	9.4771	8.6736	28.6	2	3	1	0.866	1.479	0.995	0.899	0.666	1.772	0.738	0.847	0.81	1.04	0.68
O96028	Histone-lysine N-methyltransferase NSD	NSD2	152.26	53.694	5.6	7	7	7	0.996	1.041	0.694	0.951	1.021	0.942	1.087	1.333	0.81	0.96	1.19
P25398	40S ribosomal protein S12 OS=Homo sa	RPS12	14.515	105.5	53.8	7	18	7	1.103	1.227	0.914	0.967	0.886	1.081	0.875	0.964	0.81	0.84	0.79
Q9Y3T9	Nucleolar complex protein 2 homolog OS	NOC2L	84.918	61.686	11.7	9	15	9	1.054	1.12	0.807	0.948	1.037	1.149	0.848	1.138	0.81	1.01	0.91
P30050	60S ribosomal protein L12 OS=Homo sa	RPL12	17.818	107.24	55.2	7	15	7	1.062	1.053	0.788	0.919	1.035	0.978	0.971	1.172	0.81	0.95	1.01
Q9H0U3	Magnesium transporter protein 1 OS=Ho	MAGT1	38.036	21.189	8.4	3	4	3	1.022	1.159	0.841	0.919	1.009	1.057	0.949	1.022	0.81	0.95	0.90
P15954	Cytochrome c oxidase subunit 7C, mitoc	COX7C	7.2454	15.188	28.6	2	6	2	1.204	1.038	0.861	0.947	1.021	1.076	0.704	0.995	0.81	0.94	0.76
P18621	60S ribosomal protein L17 OS=Homo sa	RPL17	21.397	71.585	32.1	5	11	5	1.073	1.099	0.912	0.838	0.996	1.033	1.037	1.075	0.81	0.93	0.97
Q5RI15	Cytochrome c oxidase assembly protein (	COX20	13.291	104.93	22	2	3	2	1.028	1.113	0.805	0.92	1.039	0.933	1.107	1.163	0.81	0.92	1.06
Q9H0T7	Ras-related protein Rab-17 OS=Homo sa	RAB17	23.491	21.185	17	3	3	3	1.06	1.034	0.69	0.997	0.97	1.296	0.68	1.314	0.81	1.08	0.95
Q9UBS9	SUN domain-containing ossification facto	SUCO	139.43	7.9425	1.2	1	1	1	1.034	1.13	0.907	0.836	0.965	0.958	1.245	1.088	0.81	0.89	1.08
P48449	Lanosterol synthase OS=Homo sapiens C	LSS	83.308	62.399	14.2	9	10	9	1.091	1.273	0.946	0.958	0.869	1.303	0.755	0.942	0.81	0.92	0.72
Q9H7E9	UPF0488 protein C8orf33 OS=Homo sap	C8orf33	24.992	13.532	11.8	2	2	2	1.046	1.024	0.793	0.874	0.854	1.299	1.098	1.146	0.81	1.04	1.08
Q96DH6	RNA-binding protein Musashi homolog 2	MSI2	35.196	36.716	18.3	5	7	5	1.095	1.241	0.943	0.938	0.868	1.212	0.819	0.977	0.81	0.89	0.77
Q99618	Cell division cycle-associated protein 3 C	CDCA3	28.998	17.238	15.7	3	3	3	1.021	1.118	0.853	0.869	1.094	1.158	1.014	1.022	0.81	1.05	0.95
Q9HC21	Mitochondrial thiamine pyrophosphate ca	SLC25A19	35.511	7.1305	3.1	1	2	1	1.003	1.148	0.878	0.853	1.036	1.068	1.109	1.082	0.80	0.98	1.02
P00846	ATP synthase subunit a OS=Homo sapier	MT-ATP6	24.817	12.607	4.4	1	2	1	1.078	0.944	0.727	0.899	1.31	1.198	0.857	1.066	0.80	1.24	0.95
Q8TCC3	39S ribosomal protein L30, mitochondria	MRPL30	18.546	11.938	11.2	2	3	2	1.157	0.916	0.771	0.896	1.138	1.117	0.909	1.118	0.80	1.09	0.98
P49137	MAP kinase-activated protein kinase 2 O	MAPKAPK	45.567	19.12	6.8	3	3	3	1.061	1.473	0.999	1.038	0.621	0.97	0.915	0.924	0.80	0.63	0.73
Q13505	Metaxin-1 OS=Homo sapiens OX=9606	MTX1	51.462	24.042	10.1	3	4	3	1.075	1.251	0.917	0.952	0.843	0.948	1.049	0.995	0.80	0.77	0.88
Q562F6	Shugoshin 2 OS=Homo sapiens OX=960	SGO2	144.74	8.8076	1.3	1	1	1	1.142	1.025	0.911	0.83	1.06	1.218	0.831	1.073	0.80	1.05	0.88
Q9BXK1	Krueppel-like factor 16 OS=Homo sapier	KLF16	25.43	34.06	14.7	2	2	2	1.094	1.285	0.926	0.985	0.769	1.284	0.798	0.869	0.80	0.86	0.70
Q02952	A-kinase anchor protein 12 OS=Homo sa	AKAP12	191.48	323.31	27.3	33	43	33	1.087	1.346	0.995	0.959	0.717	0.83	1.095	0.959	0.80	0.64	0.84
P61163	Alpha-centractin OS=Homo sapiens OX=	ACTR1A	42.613	67.613	15.7	6	9	2	1.145	1.156	0.909	0.938	0.878	0.994	0.928	0.989	0.80	0.81	0.83
P0DPF3	Neuroblastoma breakpoint family membe	NBPF9	127.75	59.276	7	4	5	4	1.031	1.057	0.791	0.884	1.089	1.205	0.912	1.106	0.80	1.10	0.97
P30049	ATP synthase subunit delta, mitochondria	ATP5F1D	17.49	15.419	13.7	2	4	2	1.091	1.201	0.874	0.962	0.953	1.095	0.84	1.018	0.80	0.89	0.81
P49458	Signal recognition particle 9 kDa protein	SRP9	10.112	39.422	54.7	5	10	5	1.148	1.186	0.921	0.947	0.926	1.156	0.696	0.927	0.80	0.89	0.70
P00918	Carbonic anhydrase 2 OS=Homo sapiens	CA2	29.246	80.291	46.5	9	14	8	1.105	1.365	0.952	1.024	0.691	1.072	0.862	0.928	0.80	0.71	0.72
P54132	Bloom syndrome protein OS=Homo sapi	BLM	159	46.267	4.9	7	7	6	0.899	0.786	0.538	0.809	1.296	1.171	1.167	1.639	0.80	1.46	1.67
P55145	Mesencephalic astrocyte-derived neurotro	MANF	20.7	67.907	32.4	6	10	6	1.088	1.14	0.874	0.907	0.975	0.996	0.994	1.047	0.80	0.88	0.92
Q93045	Stathmin-2 OS=Homo sapiens OX=9606	STMN2	20.828	6.6417	10.6	2	5	1	1.123	1.04	0.823	0.906	0.906	0.988	1.266	0.984	0.80	0.88	1.04
Q15014	Mortality factor 4-like protein 2 OS=Hor	MORF4L2	32.307	35.382	17.7	5	8	4	1.017	1.326	0.909	0.963	0.849	1.247	0.823	0.955	0.80	0.89	0.76
O95674	Phosphatidate cytidylyltransferase 2 OS=	CDS2	51.417	33.056	8.5	2	3	2	1.128	1.354	1.058	0.925	0.775	0.935	1.037	0.81	0.80	0.69	0.74
P54277	PMS1 protein homolog 1 OS=Homo sapi	PMS1	105.83	22.815	4.2	3	4	3	1.174	1.029	0.856	0.904	1.005	1.024	1.016	0.993	0.80	0.92	0.91
Q13501	Sequestosome-1 OS=Homo sapiens OX=	SQSTM1	47.687	260.55	60.5	15	42	15	0.94	0.848	0.594	0.834	1.212	1.081	1.202	1.505	0.80	1.28	1.51
Q12834	Cell division cycle protein 20 homolog O	CDC20	54.722	13.012	6.8	2	2	2	1.154	1.322	0.975	1.001	0.891	1.066	0.702	0.835	0.80	0.79	0.62
P35610	Sterol O-acyltransferase 1 OS=Homo sap	SOAT1	64.734	48.697	13.6	5	8	5	1.155	1.283	0.994	0.95	0.773	1.1	0.853	0.9	0.80	0.77	0.72
P61604	10 kDa heat shock protein, mitochondrial	HSPE1	10.932	79.972	70.6	8	21	8	1.146	1.188	0.926	0.935	0.939	0.999	0.94	0.95	0.80	0.83	0.81
O94952	F-box only protein 21 OS=Homo sapiens	FBXO21	72.269	31.821	4.9	2	2	2	1.115	1.193	0.954	0.886	0.824	1.108	0.91	1.083	0.80	0.84	0.86
Q8WWK9	Cytoskeleton-associated protein 2 OS=H	CKAP2	76.986	44.136	8.1	5	6	5	0.936	0.866	0.577	0.858	1.213	1.357	0.921	1.503	0.80	1.43	1.35
Q13423	NAD(P) transhydrogenase, mitochondrial	NNT	113.89	131.6	16.5	16	21	16	1.114	1.137	0.864	0.928	0.984	1.22	0.74	0.99	0.80	0.98	0.77
O14684	Prostaglandin E synthase OS=Homo sapi	PTGES	17.102	17.756	18.4	3	5	3	1.01	1.172	0.861	0.876	0.821	0.975	1.053	1.117	0.80	0.82	0.99
Q9H6K4	Optic atrophy 3 protein OS=Homo sapier	OPA3	19.996	6.421	5	1	2	1	1.107	1.092	0.846	0.904	1.03	1.264	0.818	0.997	0.80	1.04	0.83
O14965	Aurora kinase A OS=Homo sapiens OX=	AURKA	45.809	65.351	26.8	9	11	9	1.216	1.187	0.962	0.949	0.998	1.266	0.487	0.802	0.80	0.94	0.54
Q9BW19	Kinesin-like protein KIFC1 OS=Homo sa	KIFC1	73.747	119.9	24.7	13	14	13	0.968	0.819	0.587	0.834	1.247	1.158	1.037	1.384	0.80	1.35	1.35
P10586	Receptor-type tyrosine-protein phosphata	PTPRF	212.88	93.683	8.7	11	12	11	1.169	1.203	0.947	0.938	0.946	0.883	0.989	0.971	0.79	0.77	0.83
O75380	NADH dehydrogenase [ubiquinone] iron-	NDUFS6	13.711	26.567	33.1	3	4	3	1.116	1.23	0.945	0.918	0.884	1.195	0.745	1.019	0.79	0.89	0.75
P04181	Ornithine aminotransferase, mitochondria	OAT	48.534	70.284	21.4	8	10	8	0.978	1.042	0.738	0.866	1.069	1	1.256	1.381	0.79	1.02	1.31
P11388	DNA topoisomerase 2-alpha OS=Homo s	TOP2A	174.38	323.31	29.7	42	67	32	1.136	1.136	0.898	0.906	1.006	1.122	0.841	0.941	0.79	0.94	0.78
Q99470	Stromal cell-derived factor 2 OS=Homo s	SDF2	23.026	41.676	28.9	4	6	4	1.082	1.305	0.929	0.966	0.812	1.084	0.718	0.956	0.79	0.79	0.70
P46776	60S ribosomal protein L27a OS=Homo s	RPL27A	16.561	56.433	41.2	7	14	7	1.137	1.05	0.85	0.886	1.089	0.981	0.979	1.092	0.79	0.95	0.95
P08865	40S ribosomal protein SA OS=Homo sap	RPSA	32.854	130.01	44.4	10	25	10	1.112	1.211	0.922	0.921	0.932	1.141	0.843	0.967	0.79	0.89	0.78
Q8IYB5	Stromal membrane-associated protein 1 C	SMAP1	50.386	25.865	6.6	3	5	3	1.172	1.281	0.977	0.969	0.837	0.925	1.022	0.906	0.79	0.72	0.79
Q8NFD5	AT-rich interactive domain-containing pr	ARID1B	236.12	49.164	3.8	8	10	8	1.136	1.089	0.823	0.942	1.022	1.12	0.773	0.995	0.79	0.96	0.79
Q9NZH5	Securin-2 OS=Homo sapiens OX=9606 (	PTTG2	22.301	6.9121	7.4	1	1	1	1.205	1.107	0.91	0.924	1.064	0.785	1.01	0.953	0.79	0.80	0.85
Q93096	Protein tyrosine phosphatase type IVA 1	PTP4A1	19.815	21.554	25.4	3	3	3	1.106	1.36	0.981	0.974	0.837	0.994	0.879	0.876	0.79	0.74	0.71
Q8TCG2	Phosphatidylinositol 4-kinase type 2-beta	PI4K2B	54.744	14.575	4.8	2	2	2	1.029	1.082	0.702	0.971	1.022	1.202	0.747	1.331	0.79	1.05	0.98
P27707	Deoxycytidine kinase OS=Homo sapiens	DCK	30.518	9.7918	11.5	2	1	1	0.973	1.102	0.667	0.977	0.971	0.9	1.028	1.511	0.79	0.90	1.22
Q9BRS2	Serine/threonine-protein kinase RIO1 OS	RIOK1	65.582	12.167	2.8	2	2	2	1.234	0.951	0.875	0.856	1.122	0.819	1.026	1.108	0.79	0.89	0.98
Q9UL33	Trafficking protein particle complex subu	TRAPPC2L	16.145	21.532	22.1	3	5	3	1.115	1.325	0.992	0.941	0.828	0.916	1.01	0.935	0.79	0.71	0.80
Q96HC4	PDZ and LIM domain protein 5 OS=Hon	PDLIM5	63.944	66.89	14.9	7	10	7	1.131	1.362	0.986	0.988	0.757	0.828	1.094	1.058	0.79	0.64	0.86
O15294	UDP-N-acetylglucosamine--peptide N-ac	OGT	116.92	225.76	18.6	14	18	14	1.015	1.372	0.925	0.965	0.732	0.983	1.081	1.043	0.79	0.72	0.89
O00468	Agrin OS=Homo sapiens OX=9606 GN=	AGRN	217.23	323.31	26.2	35	48	35	1.115	1.309	0.956	0.962	0.848	1.04	0.865	0.973	0.79	0.78	0.76
Q6P1L8	39S ribosomal protein L14, mitochondria	MRPL14	15.947	30.785	29.7	3	4	3	1.013	1.224	0.879	0.891	0.988	1.179	0.888	1.084	0.79	0.97	0.88
P98082	Disabled homolog 2 OS=Homo sapiens C	DAB2	82.447	171.26	28.7	16	23	16	1.119	1.335	0.977	0.964	0.747	0.938	0.976	0.937	0.79	0.69	0.78
P62829	60S ribosomal protein L23 OS=Homo sa	RPL23	14.865	64.708	55.7	7	18	7	1.049	1.083	0.798	0.888	1.039	1.074	0.901	1.105	0.79	0.99	0.94
Q9UJF2	Ras GTPase-activating protein nGAP OS	RASAL2	128.56	27.254	3.9	4	4	4	1.16	1.142	0.975	0.844	0.935	1.183	0.673	1.166	0.79	0.92	0.80
P61247	40S ribosomal protein S3a OS=Homo sap	RPS3A	29.945	96.199	37.5	12	28	12	1.096	1.124	0.895	0.859	0.971	1.158	0.886	1.028	0.79	0.96	0.86
P45880	Voltage-dependent anion-selective chann	VDAC2	31.566	187.75	49.7	13	30	12	1.124	1.181	0.884	0.937	0.897	0.955	0.973	0.993	0.79	0.80	0.85
O43819	Protein SCO2 homolog, mitochondrial O	SCO2	29.81	17.153	15.8	3	3	3	1.067	1.075	0.907	0.785	1.075	1.268	0.777	1.24	0.79	1.09	0.94
Q15072	Zinc finger protein OZF OS=Homo sapie	ZNF146	33.307	8.1621	5.1	1	1	1	1.08	0.871	0.668	0.873	1.2	1.175	0.734	1.522	0.79	1.22	1.16
O95573	Long-chain-fatty-acid--CoA ligase 3 OS=	ACSL3	80.419	93.732	21.5	10	14	9	1.116	1.212	0.912	0.926	0.899	0.954	0.988	0.936	0.79	0.80	0.83
Q92520	Protein FAM3C OS=Homo sapiens OX=	FAM3C	24.68	54.989	36.1	6	8	6	1.168	1.147	0.914	0.913	0.865	1.192	0.852	0.877	0.79	0.89	0.75
O75251	NADH dehydrogenase [ubiquinone] iron-	NDUFS7	23.563	6.4331	4.2	1	2	1	1.122	1.221	0.951	0.897	1.019	1.216	0.694	0.933	0.79	0.95	0.69
Q15004	PCNA-associated factor OS=Homo sapie	PCLAF	11.986	59.848	56.8	6	8	6	0.896	0.727	0.502	0.778	1.471	1.57	0.899	1.466	0.79	1.87	1.46
Q96HY7	Probable 2-oxoglutarate dehydrogenase E	DHTKD1	103.08	82.113	16.3	10	10	9	1.039	1.161	0.829	0.906	0.928	1.101	1.053	0.975	0.79	0.92	0.92
P32969	60S ribosomal protein L9 OS=Homo sap	RPL9	21.863	58.08	35.9	7	14	7	1.123	1.138	0.878	0.905	0.943	1.157	0.88	0.99	0.79	0.93	0.83
Q8N5G2	Macoilin OS=Homo sapiens OX=9606 C	MACO1	76.177	13.537	3.3	2	2	2	1.098	1.147	0.87	0.9	1.071	0.883	1.005	1.093	0.79	0.87	0.93
Q96GD4	Aurora kinase B OS=Homo sapiens OX=	AURKB	39.31	20.146	9.9	3	3	3	1.085	1.022	0.807	0.854	1.083	1.151	0.966	1.147	0.79	1.06	1.00
Q9Y653	Adhesion G-protein coupled receptor G1	ADGRG1	77.737	18.619	3.9	2	2	2	0.872	0.946	0.604	0.829	1.209	1.091	1.267	1.187	0.79	1.27	1.35
Q9C0C4	Semaphorin-4C OS=Homo sapiens OX=9	SEMA4C	92.622	118.92	18.5	13	17	13	1.13	1.315	0.942	0.985	0.724	0.892	1.028	0.954	0.79	0.66	0.81
P07099	Epoxide hydrolase 1 OS=Homo sapiens (	EPHX1	52.948	53.835	19.6	6	7	6	1.069	0.967	0.626	0.978	1.022	1.019	1.103	1.289	0.79	1.00	1.17
P15880	40S ribosomal protein S2 OS=Homo sap	RPS2	31.324	92.457	32.8	9	21	9	1.051	1.214	0.866	0.918	0.878	1.244	0.875	1.086	0.79	0.94	0.87
Q5JTH9	RRP12-like protein OS=Homo sapiens C	RRP12	143.7	156.88	15.3	16	18	16	1.104	1.163	0.86	0.925	0.956	1.032	0.992	1.059	0.79	0.88	0.90
Q99943	1-acyl-sn-glycerol-3-phosphate acyltransf	AGPAT1	31.716	25.924	13.4	3	3	3	1.08	1.177	0.795	0.982	1.047	1.021	0.956	0.957	0.79	0.92	0.85
Q8NHQ9	ATP-dependent RNA helicase DDX55 O	DDX55	68.546	22.896	6.8	4	4	4	1.04	0.901	0.693	0.835	1.274	0.963	1.119	1.338	0.79	1.15	1.27
Q13572	Inositol-tetrakisphosphate 1-kinase OS=I	ITPK1	45.621	15.288	5.3	2	2	2	1.124	1.029	0.796	0.898	1.155	0.924	1.047	1.07	0.79	0.97	0.98
Q8WX93	Palladin OS=Homo sapiens OX=9606 Gl	PALLD	150.56	98.23	9.3	10	15	10	1.112	1.355	0.959	0.981	0.695	0.891	1.058	0.943	0.79	0.64	0.81
Q16836	Hydroxyacyl-coenzyme A dehydrogenase	HADH	34.293	35.829	16.9	4	8	4	1.073	0.981	0.686	0.929	1.026	0.998	0.968	1.243	0.79	0.99	1.08
P08195	4F2 cell-surface antigen heavy chain OS=	SLC3A2	67.993	323.31	32.9	17	40	17	1.114	1.246	0.914	0.941	0.866	0.904	1.022	1.086	0.79	0.75	0.89
Q16540	39S ribosomal protein L23, mitochondria	MRPL23	17.781	18.701	18.3	2	2	2	1.135	1.257	0.913	0.967	0.869	1.089	0.857	0.903	0.79	0.82	0.74
P08174	Complement decay-accelerating factor OS	CD55	41.4	54.115	12.6	4	8	4	1.138	1.191	0.958	0.872	0.982	0.968	0.992	0.917	0.79	0.84	0.82
Q99986	Serine/threonine-protein kinase VRK1 O	VRK1	45.476	67.795	22.5	7	9	6	1.048	1.019	0.671	0.953	1.024	0.971	1.09	1.323	0.79	0.97	1.17
Q96CN7	Isochorismatase domain-containing prote	ISOC1	32.236	60.762	21.5	5	9	5	1.128	1.091	0.835	0.908	0.952	1.157	0.767	1.02	0.79	0.95	0.81
P39023	60S ribosomal protein L3 OS=Homo sap	RPL3	46.108	113.98	34.7	13	28	13	1.107	1.155	0.899	0.877	0.947	1.181	0.92	1.018	0.79	0.94	0.86
P42677	40S ribosomal protein S27 OS=Homo sa	RPS27	9.461	15.294	28.6	2	4	1	0.912	1.571	1.034	0.915	0.717	1.372	0.866	0.822	0.78	0.84	0.68
Q7Z6M2	F-box only protein 33 OS=Homo sapiens	FBXO33	62.629	5.9802	1.4	1	1	1	1.005	1.095	0.698	0.95	1.013	0.939	1.117	1.294	0.78	0.93	1.15
Q92685	Dol-P-Man:Man(5)GlcNAc(2)-PP-Dol al	ALG3	50.126	6.484	2.3	1	1	1	1.054	1.192	0.849	0.913	1.178	0.966	0.898	1.041	0.78	0.95	0.86
Q9Y291	28S ribosomal protein S33, mitochondria	MRPS33	12.629	12.938	25.5	2	2	2	0.95	1.142	0.937	0.704	1.293	1.478	0.769	1.063	0.78	1.32	0.88
Q969N2	GPI transamidase component PIG-T OS=	PIGT	65.699	45.956	13.3	7	9	7	1.144	1.223	0.914	0.942	0.943	1.2	0.758	1.045	0.78	0.91	0.76
Q9UGV2	Protein NDRG3 OS=Homo sapiens OX=	NDRG3	41.408	37.189	17.6	5	6	5	1.053	1.368	0.933	0.965	0.758	1.042	0.912	1.033	0.78	0.74	0.80
Q6UXH1	Cysteine-rich with EGF-like domain prot	CRELD2	38.191	48.993	12.5	5	7	5	1.133	1.116	0.774	0.989	0.993	1.211	0.772	1.077	0.78	0.98	0.82
Q99538	Legumain OS=Homo sapiens OX=9606 (	LGMN	49.411	55.553	11.5	4	6	4	1.097	1.452	1.032	0.966	0.738	1.034	0.893	0.776	0.78	0.70	0.65
P56378	6.8 kDa mitochondrial proteolipid OS=H	MP68	6.662	6.7904	15.5	1	4	1	0.983	1.693	1.162	0.934	0.597	1.546	0.668	0.556	0.78	0.80	0.46
P99999	Cytochrome c OS=Homo sapiens OX=96	CYCS	11.749	45.239	32.4	4	7	4	1.108	0.877	0.572	0.982	1.095	0.897	1.104	1.291	0.78	1.00	1.21
Q9UGP8	Translocation protein SEC63 homolog O	SEC63	87.996	77.906	17.8	10	12	10	1.095	1.327	0.934	0.962	0.783	1	0.934	0.956	0.78	0.74	0.78
P14406	Cytochrome c oxidase subunit 7A2, mito	COX7A2	9.3959	15.87	27.7	2	4	2	1.148	1.346	0.991	0.961	0.782	1.148	0.793	0.815	0.78	0.77	0.64
Q71F23	Centromere protein U OS=Homo sapiens	CENPU	47.521	30.463	11.2	4	6	4	1.121	0.843	0.677	0.86	1.275	1.305	0.98	1.087	0.78	1.31	1.05
P00367	Glutamate dehydrogenase 1, mitochondri	GLUD1	61.397	167.57	34.6	16	30	16	1.094	1.135	0.828	0.916	0.911	0.987	0.987	1.079	0.78	0.85	0.93
P62888	60S ribosomal protein L30 OS=Homo sa	RPL30	12.784	68.009	66.1	7	18	7	1.093	1.194	0.899	0.888	0.955	0.993	1.057	1.029	0.78	0.85	0.91
O00291	Huntingtin-interacting protein 1 OS=Hon	HIP1	116.22	37.905	7.7	7	7	5	1.024	1.297	0.875	0.938	0.72	1.176	0.925	1.09	0.78	0.82	0.87
P23378	Glycine dehydrogenase (decarboxylating)	GLDC	112.73	318.16	37.6	27	50	27	1.119	1.201	0.893	0.918	0.927	1.007	0.952	0.96	0.78	0.83	0.82
Q86YH6	Decaprenyl-diphosphate synthase subuni	PDSS2	44.128	16.483	3.3	1	1	1	1.09	1.38	0.973	0.955	0.736	1.261	0.778	0.868	0.78	0.81	0.67
Q99447	Ethanolamine-phosphate cytidylyltransfe	PCYT2	43.835	36.728	14.7	4	5	4	1.093	0.988	0.708	0.916	1.154	1.041	0.968	1.201	0.78	1.05	1.04
P60033	CD81 antigen OS=Homo sapiens OX=96	CD81	25.809	29.333	15.3	2	3	2	1.077	1.252	0.865	0.952	0.884	1.128	0.942	0.948	0.78	0.86	0.81
Q9NRX2	39S ribosomal protein L17, mitochondria	MRPL17	20.05	19.34	16	3	5	3	1.151	1.164	0.913	0.893	0.994	1.212	0.729	1.026	0.78	0.95	0.76
Q9NP72	Ras-related protein Rab-18 OS=Homo sa	RAB18	22.977	70.518	46.6	9	13	9	1.126	1.208	0.875	0.945	0.887	0.995	0.981	1.051	0.78	0.81	0.87
Q4ADV7	RAB6A-GEF complex partner protein 1 (	RIC1	159.3	17.783	2.6	3	4	3	1.16	1.173	0.93	0.889	0.999	0.928	1.015	0.93	0.78	0.83	0.83
Q13206	Probable ATP-dependent RNA helicase I	DDX10	100.89	51.716	9.1	7	8	7	1.02	1.067	0.737	0.89	1.027	1.021	0.966	1.237	0.78	0.98	1.06
P51809	Vesicle-associated membrane protein 7 C	VAMP7	24.935	22.596	9.1	2	3	2	1.14	1.235	0.988	0.863	0.95	1.086	0.904	0.896	0.78	0.86	0.76
P61026	Ras-related protein Rab-10 OS=Homo sa	RAB10	22.541	18.047	26	4	9	3	1.075	1.445	0.99	0.974	0.688	1.247	0.678	0.949	0.78	0.77	0.65
P48651	Phosphatidylserine synthase 1 OS=Homo	PTDSS1	55.527	11.961	3.8	2	2	2	1.074	1.254	0.887	0.927	0.849	0.965	1.1	1.013	0.78	0.78	0.91
Q6Y1H2	Very-long-chain (3R)-3-hydroxyacyl-Co/	HACD2	28.368	20.761	12.2	3	5	3	1.091	1.214	0.891	0.905	0.832	1.203	0.923	1	0.78	0.88	0.83
Q6NUQ4	Transmembrane protein 214 OS=Homo s	TMEM214	77.15	73.912	19	11	15	11	1.037	1.017	0.688	0.912	1.066	1.23	0.95	1.248	0.78	1.12	1.07
Q13309	S-phase kinase-associated protein 2 OS=	SKP2	47.76	37.559	10.8	6	8	6	1.023	1.006	0.66	0.92	1.093	1.042	1.044	1.323	0.78	1.05	1.17
Q99595	Mitochondrial import inner membrane tra	TIMM17A	18.023	7.6444	12.9	1	1	1	1.036	1.36	0.864	1.001	0.733	1.392	0.7	0.969	0.78	0.89	0.70

O75844	CAAX prenyl protease 1 homolog OS=H	ZMPSTE24	54.812	36.242	13.1	5	6	5	1.094	1.231	0.862	0.947	1.028	1.012	0.967	1.018	0.78	0.88	0.85
Q8N0U8	Vitamin K epoxide reductase complex su	VKORC1L	19.835	18.721	15.3	3	4	3	1.192	0.933	0.768	0.885	1.019	1.067	1.009	1.132	0.78	0.98	1.01
Q9Y5L4	Mitochondrial import inner membrane tra	TIMM13	10.5	36.633	52.6	4	6	4	1.083	1.159	0.804	0.94	1.005	1.247	0.781	1.053	0.78	1.00	0.82
Q92503	SEC14-like protein 1 OS=Homo sapiens	SEC14L1	81.249	7.5407	1.5	1	1	1	1.1	1.305	0.917	0.953	0.877	0.773	1.131	0.97	0.78	0.69	0.87
Q07020	60S ribosomal protein L18 OS=Homo sa	RPL18	21.634	61.731	31.4	6	9	6	0.761	0.828	0.58	0.655	1.098	0.779	1.467	2.372	0.78	1.18	2.42
P10109	Adrenodoxin, mitochondrial OS=Homo s	FDX1	19.393	18.585	13.6	3	4	3	1.178	1.158	0.866	0.949	0.908	1.179	0.893	0.965	0.78	0.89	0.80
Q9HD33	39S ribosomal protein L47, mitochondria	MRPL47	29.45	41.614	24	6	7	6	1.213	1.087	0.873	0.914	1.001	1.132	0.804	1.064	0.78	0.93	0.81
O95429	BAG family molecular chaperone regulat	BAG4	49.593	15.656	5.3	2	2	2	1.13	1.221	0.856	0.97	0.814	0.9	1.062	1.039	0.78	0.73	0.89
O15270	Serine palmitoyltransferase 2 OS=Homo	SPTLC2	62.924	8.3151	2.1	1	1	1	0.915	1.281	0.757	0.948	0.677	0.892	1.303	1.421	0.78	0.71	1.24
P98160	Basement membrane-specific heparan sul	HSPG2	468.83	92.822	3.8	13	13	13	0.972	1.031	0.665	0.89	1.136	1.046	1.171	1.292	0.78	1.09	1.23
P54687	Branched-chain-amino-acid aminotransfe	BCAT1	42.966	25.897	10.6	3	3	3	1.129	1.392	1.003	0.954	0.722	0.858	1.06	0.887	0.78	0.63	0.77
P23771	Trans-acting T-cell-specific transcription	GATA3	47.915	29.719	12.9	4	5	2	1.129	1.132	0.814	0.941	0.907	0.954	0.981	1.165	0.78	0.82	0.95
Q7Z2W4	Zinc finger CCCH-type antiviral protein	ZC3HAV1	101.43	155.08	26.5	18	24	18	1.116	1.17	0.87	0.904	0.821	0.93	0.997	1.048	0.78	0.77	0.89
Q9H6R4	Nucleolar protein 6 OS=Homo sapiens O	NOL6	127.59	175.2	16.3	13	20	13	1.088	1.139	0.835	0.893	1.083	0.987	1.122	1.069	0.78	0.93	0.98
Q12824	SWI/SNF-related matrix-associated actin	SMARCB1	44.141	117.84	17.7	4	2	2	1.048	1.18	0.828	0.9	0.885	1.024	1.059	1.192	0.78	0.86	1.01
Q9Y221	60S ribosome subunit biogenesis protein	NIP7	20.462	6.6146	5	1	1	1	1.082	1.114	0.625	1.078	0.906	1.212	0.725	1.22	0.78	0.96	0.89
Q9BZE4	Nucleolar GTP-binding protein 1 OS=Ho	GTPBP4	73.964	194.47	34.2	20	25	20	1.111	1.129	0.829	0.908	1.003	1.044	0.947	1.049	0.78	0.91	0.89
Q8IXM2	Chromatin complexes subunit BAP18 OS	BAP18	17.9	12.361	8.1	1	2	1	1.163	1.343	1.042	0.899	0.689	1.102	0.809	0.982	0.77	0.71	0.71
Q14978	Nucleolar and coiled-body phosphoprote	NOLC1	73.602	70.989	12.3	11	15	11	1.067	1.261	0.849	0.954	0.941	1.051	0.936	1.045	0.77	0.86	0.85
Q96SI9	Spermatid perinuclear RNA-binding prot	STRBP	73.652	86.808	18.9	12	20	10	1.139	1.304	0.934	0.956	0.757	1.089	0.868	0.905	0.77	0.76	0.73
Q6PCB0	von Willebrand factor A domain-containi	VWA1	46.804	13.333	4.5	2	2	2	1.169	1.119	0.822	0.948	0.993	0.982	0.933	1.01	0.77	0.86	0.85
O00418	Eukaryotic elongation factor 2 kinase OS	EEF2K	82.143	18.229	4	2	2	2	1.185	1.26	0.99	0.901	0.933	0.904	0.923	0.901	0.77	0.75	0.75
Q5W0B1	RING finger protein 219 OS=Homo sapie	RNF219	81.116	12.846	2.6	2	2	2	1.129	1.188	0.845	0.947	0.965	0.928	0.932	1.079	0.77	0.82	0.87
Q96BY7	Autophagy-related protein 2 homolog B (	ATG2B	232.76	46.166	3.9	7	7	7	1.099	1.311	0.99	0.873	0.872	0.915	1.088	0.939	0.77	0.74	0.84
O75323	Protein NipSnap homolog 2 OS=Homo s	NIPSNAP2	33.742	14.763	9.1	2	3	2	1.133	1.279	0.923	0.941	0.839	1.183	0.786	0.931	0.77	0.84	0.71
Q9NUL7	Probable ATP-dependent RNA helicase I	DDX28	59.58	19.009	6.9	3	3	3	1.208	1.107	0.935	0.853	0.97	1.256	0.835	0.85	0.77	0.96	0.73
P23396	40S ribosomal protein S3 OS=Homo sap	RPS3	26.688	111.67	53.9	13	33	13	1.143	1.142	0.861	0.903	0.959	0.986	0.937	0.979	0.77	0.85	0.84
P62269	40S ribosomal protein S18 OS=Homo sa	RPS18	17.718	36.501	25	4	10	4	1.125	1.177	0.889	0.888	1.059	0.96	1	0.97	0.77	0.88	0.86
P11474	Steroid hormone receptor ERR1 OS=Hor	ESRRA	45.509	13.857	5.4	2	2	2	1.192	1.089	0.902	0.858	1.015	1.273	0.78	0.916	0.77	1.00	0.74
Q8NEY1	Neuron navigator 1 OS=Homo sapiens O	NAV1	202.47	100.43	8.6	12	13	12	1.072	0.996	0.679	0.916	1.07	0.936	1.115	1.418	0.77	0.97	1.22
Q96T76	MMS19 nucleotide excision repair protei	MMS19	113.29	49.884	7.1	6	9	6	0.974	1.408	0.838	0.996	0.677	1.289	0.79	1.141	0.77	0.83	0.81
Q9BSW7	Synaptotagmin-17 OS=Homo sapiens OX	SYT17	53.848	6.3443	2.1	1	1	1	1.025	1.096	0.695	0.938	1.004	1.172	0.918	1.26	0.77	1.03	1.03
Q9H223	EH domain-containing protein 4 OS=Hor	EHD4	61.174	63.031	20.7	10	12	7	1.155	1.166	0.866	0.92	0.92	0.886	1.091	1.045	0.77	0.78	0.92
Q8N6L1	Keratinocyte-associated protein 2 OS=Ho	KRTCAP2	14.678	8.8733	12.5	1	1	1	1.078	0.993	0.686	0.907	0.918	0.966	1.108	1.439	0.77	0.91	1.23
P62241	40S ribosomal protein S8 OS=Homo sap	RPS8	24.205	106.44	48.1	9	17	9	1.114	1.116	0.818	0.897	0.992	1.007	1.02	1.079	0.77	0.90	0.94
O15213	WD repeat-containing protein 46 OS=Ho	WDR46	68.07	90.214	23.9	9	10	9	1.029	0.993	0.644	0.911	1.06	1.393	0.848	1.268	0.77	1.21	1.05
O14602	Eukaryotic translation initiation factor 1A	EIF1AY	16.442	38.935	33.3	5	8	1	1.032	1.53	1.043	0.926	0.647	1.23	0.735	0.977	0.77	0.73	0.67
Q8IYB3	Serine/arginine repetitive matrix protein 1	SRRM1	102.33	20.386	4.3	3	4	3	1.15	1.099	0.859	0.869	1.023	0.996	1.03	1.156	0.77	0.90	0.97
O75054	Immunoglobulin superfamily member 3 C	IGSF3	135.19	82.147	8.9	10	11	10	1.104	1.264	0.887	0.932	0.825	0.868	1.127	0.998	0.77	0.71	0.90
P08708	40S ribosomal protein S17 OS=Homo sa	RPS17	15.55	102.86	57	9	15	9	1.097	1.208	0.886	0.884	0.892	1.157	0.91	0.999	0.77	0.89	0.83
Q9NZE8	39S ribosomal protein L35, mitochondria	MRPL35	21.514	8.3946	5.3	1	2	1	1.16	0.927	0.616	0.986	1.011	0.88	1.088	1.292	0.77	0.91	1.14
P49454	Centromere protein F OS=Homo sapiens	CENPF	367.76	132.31	6.3	20	20	20	1.132	1.075	0.786	0.907	1.016	0.966	1.061	1.109	0.77	0.90	0.98
Q9NTJ5	Phosphatidylinositide phosphatase SAC1	SACM1L	66.966	46.019	12.8	7	7	7	1.034	1.465	0.893	1.023	0.733	0.993	1.085	0.976	0.77	0.69	0.82
P62906	60S ribosomal protein L10a OS=Homo s	RPL10A	24.831	124.89	38.2	9	20	9	1.005	1.058	0.716	0.864	1.024	1.089	1.025	1.296	0.77	1.02	1.13
O95297	Myelin protein zero-like protein 1 OS=H	MPZL1	29.082	43.891	21.9	6	9	6	1.068	1.088	0.715	0.936	0.973	1.013	0.98	1.286	0.77	0.92	1.05
Q08209	Serine/threonine-protein phosphatase 2B	PPP3CA	58.687	27.77	10	4	3	2	1.146	1.503	1.04	0.988	0.59	0.904	0.977	0.82	0.77	0.56	0.68
Q8N556	Actin filament-associated protein 1 OS=I	AFAP1	80.724	34.001	7.8	5	6	5	1.047	1.416	0.939	0.946	0.695	0.99	0.86	1.065	0.77	0.68	0.78
Q15109	Advanced glycosylation end product-spec	AGER	42.802	6.4463	3.7	1	1	1	1.075	1.162	0.712	1	0.886	0.963	0.912	1.319	0.77	0.83	1.00
P62917	60S ribosomal protein L8 OS=Homo sap	RPL8	28.024	47.019	33.1	5	13	5	0.966	1.025	0.68	0.843	0.939	1.132	1.089	1.373	0.76	1.04	1.24
O95235	Kinesin-like protein KIF20A OS=Homo s	KIF20A	100.28	53.422	9.7	7	9	7	1.36	1.502	1.228	0.958	0.639	0.998	0.685	0.533	0.76	0.57	0.43
O60336	Mitogen-activated protein kinase-binding	MAPKBP1	163.82	7.1299	0.6	1	1	1	1.257	0.969	0.681	1.019	0.976	1.321	0.701	0.928	0.76	1.03	0.73
Q7Z5K2	Wings apart-like protein homolog OS=Ho	WAPL	132.94	20.375	2.6	3	3	3	1.126	1.22	0.862	0.929	0.828	0.912	1.1	1.052	0.76	0.74	0.92
P52732	Kinesin-like protein KIF11 OS=Homo sa	KIF11	119.16	114.28	16.7	15	20	15	1.067	1.024	0.735	0.861	1.034	1.177	0.968	1.25	0.76	1.06	1.06
Q58EX2	Protein sidekick-2 OS=Homo sapiens OX	SDK2	239.39	17.427	0.9	1	1	1	1.076	1.165	0.788	0.922	1.107	1.026	1.044	0.931	0.76	0.95	0.88
Q8N5L8	Ribonuclease P protein subunit p25-like	RPP25L	17.631	7.6811	5.5	1	2	1	1.174	1.159	0.861	0.918	0.953	1.173	0.841	0.918	0.76	0.91	0.75
Q9NVV4	Poly(A) RNA polymerase, mitochondrial	MTPAP	66.171	66.471	15.1	6	7	6	1.082	1.273	0.87	0.925	0.793	1.06	1.013	1.032	0.76	0.79	0.87
P09958	Furin OS=Homo sapiens OX=9606 GN=	FURIN	86.677	12.617	2.1	2	2	2	1.133	1.431	1.09	0.863	0.737	1.264	0.765	0.792	0.76	0.78	0.61
P46778	60S ribosomal protein L21 OS=Homo sa	RPL21	18.565	46.285	33.1	4	6	3	0.994	1.304	0.904	0.846	0.893	1.37	0.857	0.974	0.76	0.98	0.80
Q9P035	Very-long-chain (3R)-3-hydroxyacyl-CoA	HACD3	43.159	37.38	14.9	4	5	4	1.133	1.357	0.979	0.916	0.737	1.098	0.834	0.919	0.76	0.74	0.70
Q9BXJ8	Transmembrane protein 120A OS=Homo	TMEM120	40.61	8.3346	3.2	1	1	1	1.165	1.45	1.099	0.889	0.784	0.982	0.865	0.793	0.76	0.68	0.63
Q9Y570	Protein phosphatase methylesterase 1 OS	PPME1	42.315	61.792	29.5	8	12	8	1.17	1.452	1.007	0.986	0.623	0.791	0.978	0.903	0.76	0.54	0.72
Q9Y3Z3	Deoxynucleoside triphosphate triphospho	SAMHD1	72.2	90.936	21.9	11	14	11	1.164	1.43	1.009	0.962	0.759	0.982	0.899	0.776	0.76	0.67	0.65
P05166	Propionyl-CoA carboxylase beta chain, m	PCCB	58.215	77.49	28	11	12	11	1.041	1.07	0.663	0.941	0.997	0.978	1.16	1.241	0.76	0.94	1.14
Q9BT09	Protein canopy homolog 3 OS=Homo sap	CNPY3	30.748	116.91	34.9	8	8	8	1.06	1.158	0.713	0.972	0.979	0.992	0.903	1.28	0.76	0.89	0.98
O43663	Protein regulator of cytokinesis 1 OS=Ho	PRC1	71.606	65.122	14.8	8	9	8	1.049	1.076	0.711	0.903	1.085	1.181	1.013	1.17	0.76	1.07	1.03
Q5U5X0	Complex III assembly factor LYRM7 OS	LYRM7	11.955	9.4243	16.3	1	1	1	1.209	1.176	0.918	0.893	0.939	1.212	0.719	0.924	0.76	0.90	0.69
P84157	Matrix-remodeling-associated protein 7 (	MXRA7	21.466	15.857	5.4	2	2	2	1.061	1.533	0.99	0.978	0.656	1.184	0.711	0.943	0.76	0.71	0.64
Q5VT79	Annexin A8-like protein 1 OS=Homo sap	ANXA8L1	36.879	8.3616	20.8	5	6	1	1.176	1.416	0.863	1.103	0.64	0.867	0.862	0.935	0.76	0.58	0.69
Q6P4R8	Nuclear factor related to kappa-B-binding	NFRKB	139	26.974	4.9	4	4	4	1.114	1.392	0.901	0.999	0.696	1.259	0.698	0.936	0.76	0.78	0.65
Q6PK04	Coiled-coil domain-containing protein 13	CCDC137	33.231	12.87	8.7	2	2	2	0.984	1.377	0.958	0.832	0.916	1.149	1.049	0.946	0.76	0.87	0.84
Q8N9N2	Activating signal cointegrator 1 complex	ASCC1	45.509	17.268	6.8	3	3	3	1.004	1.087	0.682	0.903	0.997	1.05	1.071	1.358	0.76	0.98	1.16
P13798	Acylamino-acid-releasing enzyme OS=Ho	APEH	81.224	144.17	24.6	14	26	14	1.131	1.37	0.952	0.942	0.697	1.033	0.918	0.913	0.76	0.69	0.73
Q8NBU5	ATPase family AAA domain-containing p	ATAD1	40.744	49.649	20.8	6	8	6	1.115	1.287	0.887	0.931	0.916	1.041	0.86	1.034	0.76	0.81	0.79
Q8N1F8	Serine/threonine-protein kinase 11-intera	STK11IP	120.26	7.1201	1	1	1	1	1.048	0.741		1.354	0.673		0.682	0.858	0.76	0.38	0.86
Q9BV79	Enoyl-[acyl-carrier-protein] reductase, mi	MECR	40.461	8.5678	3.2	1	3	1	1.113	1.118	0.775	0.911	1.065	1	0.976	1.091	0.76	0.93	0.93
P62249	40S ribosomal protein S16 OS=Homo sa	RPS16	16.445	72.437	49.3	9	13	9	1.007	1.039	0.682	0.864	1.043	0.972	1.175	1.354	0.76	0.98	1.24
Q9H1Y0	Autophagy protein 5 OS=Homo sapiens (	ATG5	32.447	15.296	7.6	2	2	2	1.049	0.893	0.627	0.84	1.105	1.005	1.256	1.381	0.76	1.09	1.36
Q86U28	Iron-sulfur cluster assembly 2 homolog, r	ISCA2	16.476	22.688	22.1	3	4	3	1.151	1.261	0.911	0.91	0.943	1.09	0.813	0.944	0.75	0.84	0.73
P52789	Hexokinase-2 OS=Homo sapiens OX=96	HK2	102.38	188.21	25.5	18	28	17	1.131	1.232	0.887	0.897	0.849	0.959	1.009	1.043	0.75	0.77	0.87
Q99797	Mitochondrial intermediate peptidase OS	MIPEP	80.64	51.589	9.7	6	6	6	1.053	1.264	0.855	0.893	0.969	1.23	0.788	0.932	0.75	0.95	0.74
P08236	Beta-glucuronidase OS=Homo sapiens O	GUSB	74.731	6.0199	1.4	1	1	1	1.085	1.434	0.923	0.977	0.708	1.251	0.664	0.994	0.75	0.78	0.66
P51784	Ubiquitin carboxyl-terminal hydrolase 11	USP11	109.82	26.889	4.6	4	4	4	1.217	1.219	0.995	0.841	0.971	0.516	1.32	0.93	0.75	0.61	0.92
Q96LT9	RNA-binding protein 40 OS=Homo sapie	RNPC3	58.574	12.626	3.9	2	2	2	1.015	1.386	0.912	0.895	0.9	1.09	0.973	0.971	0.75	0.83	0.81
Q16595	Frataxin, mitochondrial OS=Homo sapien	FXN	23.135	32.43	24.8	4	5	4	1.063	1.08	0.678	0.934	0.987	0.897	1.18	1.258	0.75	0.88	1.14
Q8TB72	Pumilio homolog 2 OS=Homo sapiens O	PUM2	114.21	7.5858	4.6	5	5	1	1.158	1.114	0.909	0.8	1.034	0.853	1.149	1.078	0.75	0.83	0.98
P45954	Short/branched chain specific acyl-CoA (	ACADSB	47.485	36.946	15	5	4	4	1.098	1.262	0.798	0.977	0.916	0.932	0.927	1.109	0.75	0.78	0.86
P10412	Histone H1.4 OS=Homo sapiens OX=96	HIST1H1E	21.865	58.749	34.7	8	20	3	0.901	0.973	0.702	0.707	1.035	0.685	1.274	2.073	0.75	0.92	1.79
P62081	40S ribosomal protein S7 OS=Homo sap	RPS7	22.127	44.265	22.7	6	9	6	1.113	1.471	0.956	0.986	0.672	1.085	0.903	0.87	0.75	0.68	0.69
Q99439	Calponin-2 OS=Homo sapiens OX=9606	CNN2	33.697	97.933	47.2	10	13	9	1.209	1.22	0.906	0.918	0.861	0.814	1.065	1.013	0.75	0.69	0.86
P60468	Protein transport protein Sec61 subunit b	SEC61B	9.9743	20.256	37.5	3	6	3	1.178	1.174	0.911	0.855	0.984	1.062	0.81	0.978	0.75	0.87	0.76
P16422	Epithelial cell adhesion molecule OS=Ho	EPCAM	34.932	106.13	26.1	5	15	5	1.115	1.345	0.886	0.961	0.686	0.903	1.025	1.024	0.75	0.65	0.83
Q15047	Histone-lysine N-methyltransferase SETI	SETDB1	143.16	17.574	2	3	3	3	1.156	1.219	0.906	0.877	1.03	1.223	0.738	0.891	0.75	0.95	0.69
Q92747	Actin-related protein 2/3 complex subuni	ARPC1A	41.569	40.328	17	6	6	5	1.198	1.241	0.897	0.934	1.038	1.005	0.924	0.961	0.75	0.84	0.77
P62701	40S ribosomal protein S4, X isoform OS	RPS4X	29.597	73.462	36.1	9	23	4	1.094	0.98	0.655	0.901	1.07	0.951	1.14	1.205	0.75	0.97	1.13
Q8IY37	Probable ATP-dependent RNA helicase I	DHX37	129.54	32.919	4.6	5	5	5	1.061	1.167	0.745	0.925	1	1.155	0.843	1.194	0.75	0.97	0.91
Q53FV1	ORM1-like protein 2 OS=Homo sapiens	ORMDL2	17.363	6.0256	5.2	1	1	1	1.069	1.086	0.562	1.052	1.092	0.886	1.064	1.164	0.75	0.92	1.03
P61313	60S ribosomal protein L15 OS=Homo sa	RPL15	24.146	49.687	21.6	5	10	5	1.083	1.071	0.699	0.914	0.946	1.034	1.032	1.295	0.75	0.92	1.08
P25440	Bromodomain-containing protein 2 OS=I	BRD2	88.06	33.471	8.4	6	7	5	1.182	1.364	0.997	0.909	0.826	1.153	0.809	0.754	0.75	0.78	0.61
Q8NFV4	Protein ABHD11 OS=Homo sapiens OX	ABHD11	34.69	88.527	34.3	8	9	8	1.053	1.047	0.668	0.904	1.086	1.028	1.114	1.348	0.75	1.01	1.17
O60488	Long-chain-fatty-acid--CoA ligase 4 OS=	ACSL4	79.187	30.335	10.1	5	6	4	1.076	1.055	0.673	0.921	0.961	0.91	1.118	1.367	0.75	0.88	1.17
Q9UBC3	DNA (cytosine-5)-methyltransferase 3B (	DNMT3B	95.75	97.497	15.8	11	14	9	1.162	1.167	0.863	0.879	0.962	1.204	0.84	1.018	0.75	0.93	0.80
Q9BZX2	Uridine-cytidine kinase 2 OS=Homo sapi	UCK2	29.299	41.326	28	6	7	6	1.081	1.271	0.897	0.861	0.764	1.201	1.011	1.011	0.75	0.84	0.86
Q15561	Transcriptional enhancer factor TEF-3 OS	TEAD4	48.328	12.934	9.4	4	5	2	1.013	1.477	0.838	1.022	0.697	1.305	0.697	1.013	0.75	0.80	0.69
Q5VTB9	E3 ubiquitin-protein ligase RNF220 OS=	RNF220	62.765	16.285	5.3	2	2	2	1.018	1.565	1.104	0.824	0.752	1.113	0.865	0.962	0.75	0.72	0.71
P52815	39S ribosomal protein L12, mitochondria	MRPL12	21.348	42.224	22.2	4	7	4	1.161	0.905	0.622	0.92	1.203	0.959	1.017	1.201	0.75	1.05	1.07
O00629	Importin subunit alpha-3 OS=Homo sapi	KPNA4	57.886	49.323	12.3	6	9	3	0.981	1.15	0.661	0.928	0.875	0.976	1.047	1.318	0.75	0.87	1.11
Q9NRZ9	Lymphoid-specific helicase OS=Homo sa	HELLS	97.073	61.479	9.3	9	9	9	1.065	1.041	0.657	0.913	1.041	1.019	0.992	1.278	0.75	0.98	1.08
P50453	Serpin B9 OS=Homo sapiens OX=9606	SERPINB9	42.403	217.59	54.8	18	32	17	1.154	1.418	0.975	0.942	0.705	0.856	1.009	0.946	0.75	0.61	0.76
P08133	Annexin A6 OS=Homo sapiens OX=960	ANXA6	75.872	204.75	41.2	25	42	24	1.123	1.382	0.924	0.943	0.713	0.971	0.971	0.961	0.75	0.67	0.77
Q6UWY5	Olfactomedin-like protein 1 OS=Homo s	OLFML1	45.95	17.194	6.2	3	3	3	1.13	1.416	0.946	0.949	0.703	0.812	1.015	0.918	0.74	0.60	0.76
P56747	Claudin-6 OS=Homo sapiens OX=9606	CLDN6	23.291	42.634	24.5	5	7	5	1.1	1.159	0.765	0.916	0.896	1.039	0.904	1.151	0.74	0.86	0.91
Q9Y2B0	Protein canopy homolog 2 OS=Homo sap	CNPY2	20.652	44.618	39.6	6	8	6	0.808	1.894	1.029	0.981	0.639	1.684	0.748	0.686	0.74	0.86	0.53
Q93052	Lipoma-preferred partner OS=Homo sapi	LPP	65.746	38.81	10.1	5	6	5	1.18	1.112	0.844	0.861	0.892	0.869	1.032	1.025	0.74	0.77	0.90
Q92614	Unconventional myosin-XVIIIa OS=Hom	MYO18A	233.11	303.52	16.5	32	34	32	1.157	1.342	0.904	0.954	0.739	0.877	0.997	0.962	0.74	0.65	0.78
P12830	Cadherin-1 OS=Homo sapiens OX=9606	CDH1	97.455	101.43	13.9	9	18	9	1.132	1.291	0.894	0.906	0.986	0.952	0.926	0.99	0.74	0.80	0.79
Q86SF2	N-acetylgalactosaminyltransferase 7 OS=	GALNT7	75.388	72.133	13.7	7	9	7	1.204	1.141	0.87	0.872	0.936	0.902	1.099	0.998	0.74	0.78	0.89
Q15392	Delta(24)-sterol reductase OS=Homo sap	DHCR24	60.101	6.3008	1.6	1	2	1	1.156	1.307	0.904	0.925	0.86	1.092	0.869	0.896	0.74	0.79	0.72
P62841	40S ribosomal protein S15 OS=Homo sa	RPS15	17.04	23.067	20	3	4	3	1.098	1.461	0.962	0.938	0.712	1.187	0.93	0.956	0.74	0.74	0.74
O75563	Src kinase-associated phosphoprotein 2 (	SKAP2	41.216	6.9623	3.3	1	1	1	1.109	1.383	0.895	0.955	0.656	0.714	1.206	1.111	0.74	0.55	0.93
P07602	Prosaposin OS=Homo sapiens OX=9606	PSAP	58.112	119.78	27.9	14	36	14	1.147	1.281	0.873	0.929	0.823	0.935	1.069	1.026	0.74	0.72	0.86
Q5XPI4	E3 ubiquitin-protein ligase RNF123 OS=	RNF123	148.51	11.204	2.1	2	2	2	1.076	1.389	0.926	0.903	0.94	0.985	0.943	0.923	0.74	0.78	0.76
Q9BYG3	MKI67 FHA domain-interacting nucleola	NIFK	34.222	129.3	44	10	16	10	1.07	0.998	0.684	0.85	1.18	1.122	0.955	1.234	0.74	1.11	1.06
P84243	Histone H3.3 OS=Homo sapiens OX=96	H3F3A	15.328	7.1832	13.2	2	6	1	1.12	0.922	0.613	0.9	1.026	0.857	1.17	1.456	0.74	0.92	1.29
P55263	Adenosine kinase OS=Homo sapiens OX	ADK	40.545	92.023	30.7	9	15	9	1.008	1.375	0.806	0.959	0.716	0.893	1.09	1.179	0.74	0.68	0.95
Q13576	Ras GTPase-activating-like protein IQGA	IQGAP2	180.58	6.243	1.5	3	5	1	1.24	1.113	0.835	0.907	0.946	1.197	0.736	0.983	0.74	0.91	0.73
Q8N9R8	Protein SCAI OS=Homo sapiens OX=96	SCAI	70.399	13.667	3.1	2	2	2	1.068	1.041	0.665	0.895	1.147	0.96	1.115	1.205	0.74	1.00	1.10
P16035	Metalloproteinase inhibitor 2 OS=Homo	TIMP2	24.399	18.467	17.3	3	4	3	1.021	0.961	0.606	0.86	1.041	1.061	1.194	1.435	0.74	1.06	1.33
Q9P273	Teneurin-3 OS=Homo sapiens OX=9606	TENM3	300.95	47.09	2.4	6	6	6	1.098	1.497	0.967	0.952	0.918	0.765	1.102	0.884	0.74	0.65	0.77
Q7Z7N9	Transmembrane protein 179B OS=Homo	TMEM179	23.55	10.733	11	2	2	2	1.115	1.113	0.69	0.957	0.93	0.926	0.954	1.343	0.74	0.83	1.03
Q9H8H2	Probable ATP-dependent RNA helicase I	DDX31	94.086	25.169	5.9	4	4	4	1.053	1.034	0.707	0.835	1.138	0.981	1.13	1.302	0.74	1.02	1.17
P61513	60S ribosomal protein L37a OS=Homo s	RPL37A	10.275	24.611	38	3	6	3	1.032	1.095	0.704	0.867	1.013	0.987	1.127	1.291	0.74	0.94	1.14
P62913	60S ribosomal protein L11 OS=Homo sa	RPL11	20.252	41.34	22.5	5	11	5	1.182	1.238	0.891	0.896	0.936	1.088	0.739	0.911	0.74	0.84	0.68
P02462	Collagen alpha-1(IV) chain OS=Homo sa	COL4A1	160.61	29.58	3	4	5	4	1.063	1.498	0.989	0.902	0.656	0.961	1.088	0.945	0.74	0.63	0.79
O75962	Triple functional domain protein OS=Ho	TRIO	346.9	87.794	4.6	13	14	13	1.11	1.32	0.867	0.926	0.669	1.012	1.075	0.991	0.74	0.69	0.85
P61769	Beta-2-microglobulin OS=Homo sapiens	B2M	13.714	12.721	16.8	2	2	2	0.879	1.842	1.161	0.846	0.564	1.598	0.753	0.65	0.74	0.79	0.52
P11182	Lipoamide acyltransferase component of	DBT	53.486	7.3452	3.7	1	1	1	1.163	1.142	0.859	0.841	1.028	0.977	0.927	1.132	0.74	0.87	0.89
Q8NDI1	EH domain-binding protein 1 OS=Homo	EHBP1	140.02	88.953	12.3	11	10	10	1.117	1.348	0.879	0.939	0.671	0.901	1.082	1.026	0.74	0.64	0.86
Q71DI3	Histone H3.2 OS=Homo sapiens OX=96	HIST2H3A	15.388	24.907	14	3	7	2	1.471	1.877	1.53	0.938	0.377	0.732	0.532	0.293	0.74	0.33	0.25
Q15050	Ribosome biogenesis regulatory protein h	RRS1	41.193	52.194	21.4	6	9	6	1.08	1.025	0.673	0.878	1.054	1.26	0.957	1.186	0.74	1.10	1.02
O95881	Thioredoxin domain-containing protein 1	TXNDC12	19.206	22.716	22.1	3	6	3	1.167	1.321	0.909	0.924	0.804	1.078	0.833	0.893	0.74	0.76	0.69
Q92626	Peroxidasin homolog OS=Homo sapiens	PXDN	165.27	17.997	2.6	3	3	3	1.205	1.208	0.872	0.903	0.97	0.99	0.838	1	0.74	0.81	0.76
Q9Y3B9	RRP15-like protein OS=Homo sapiens C	RRP15	31.484	14.037	6.7	2	3	2	1.075	1.075	0.753	0.827	1.049	1.039	1.179	1.145	0.73	0.97	1.08
O95470	Sphingosine-1-phosphate lyase 1 OS=Ho	SGPL1	63.523	28.186	8.3	4	4	4	1.14	1.498	0.962	0.975	0.737	0.937	0.838	0.901	0.73	0.63	0.66
Q9Y5X3	Sorting nexin-5 OS=Homo sapiens OX=9	SNX5	46.816	173.78	42.1	17	21	15	1.221	1.313	0.909	0.95	0.92	0.901	1.029	0.908	0.73	0.72	0.76
Q9HCD6	Protein TANC2 OS=Homo sapiens OX=9	TANC2	219.65	28.502	2.5	4	4	4	1.185	1.412	0.934	0.971	0.723	0.92	0.912	0.895	0.73	0.63	0.70
Q96KC8	DnaJ homolog subfamily C member 1 OS	DNAJC1	63.882	73.164	19.1	10	10	10	1.126	1.21	0.796	0.917	0.836	1.121	0.87	1.112	0.73	0.84	0.85
Q15651	High mobility group nucleosome-binding	HMGN3	10.666	6.3772	18.2	2	3	1	1.15	1.453	0.986	0.922	0.677	0.878	1.045	0.906	0.73	0.60	0.75
O75683	Surfeit locus protein 6 OS=Homo sapiens	SURF6	41.45	40.663	16.9	6	6	6	0.995	0.975	0.664	0.78	1.279	0.935	1.146	1.367	0.73	1.12	1.28
Q9Y6M7	Sodium bicarbonate cotransporter 3 OS=	SLC4A7	136.04	12.528	1.8	2	3	2	1.066	1.564	0.971	0.955	0.334	0.59	1.292	1.308	0.73	0.35	0.99
P47914	60S ribosomal protein L29 OS=Homo sa	RPL29	17.752	35.839	14.5	2	3	2	0.995	1.205	0.732	0.879	0.931	1.012	1.093	1.333	0.73	0.88	1.10
P83876	Thioredoxin-like protein 4A OS=Homo s	TXNL4A	16.786	15.793	15.5	2	3	2	1.021	1.081	0.686	0.853	1.015	0.958	1.165	1.399	0.73	0.94	1.22
P62266	40S ribosomal protein S23 OS=Homo sa	RPS23	15.807	51.462	30.1	6	13	6	1.024	1.086	0.676	0.868	1.004	1.281	0.936	1.236	0.73	1.08	1.03
P60891	Ribose-phosphate pyrophosphokinase 1 (	PRPS1	34.834	63.383	18.9	4	5	3	1.21	1.037	0.669	0.975	0.882	0.701	1.148	1.312	0.73	0.70	1.09
Q9NRP2	COX assembly mitochondrial protein 2 h	CMC2	9.4598	8.6417	12.7	1	2	1	1.195	1.114	0.86	0.828	1.149	1.118	0.863	0.915	0.73	0.98	0.77
Q8NF37	Lysophosphatidylcholine acyltransferase	LPCAT1	59.151	29.881	9	4	5	4	1.159	1.444	0.969	0.926	0.79	0.807	1.02	0.859	0.73	0.61	0.72
Q9UBU8	Mortality factor 4-like protein 1 OS=Hor	MORF4L1	41.473	17.951	13.8	4	6	3	1.082	1.179	0.712	0.934	0.912	0.742	1.095	1.418	0.73	0.73	1.11

Q6UVJ0	Spindle assembly abnormal protein 6 hon	SASS6	74.396	17.091	4.1	3	3	3	0.975	0.963	0.506	0.904	1.11	1.063	1.146	1.509	0.73	1.12	1.37
Q02543	60S ribosomal protein L18a OS=Homo s	RPL18A	20.762	62.585	39.8	8	14	7	1.042	0.906	0.541	0.876	1.039	0.974	1.166	1.538	0.73	1.03	1.39
P62910	60S ribosomal protein L32 OS=Homo sa	RPL32	15.86	33.226	24.4	4	15	4	1.105	1.103	0.707	0.898	0.998	1.113	0.846	1.166	0.73	0.96	0.91
Q15404	Ras suppressor protein 1 OS=Homo sapi	RSU1	31.54	40.264	20.2	6	10	6	1.134	1.3	0.881	0.886	0.81	0.919	0.999	1.045	0.73	0.71	0.84
P23434	Glycine cleavage system H protein, mitoc	GCSH	18.884	62.338	30.6	4	12	4	1.183	1.311	0.887	0.922	0.821	1.011	0.936	0.947	0.73	0.73	0.76
Q9Y3D9	28S ribosomal protein S23, mitochondria	MRPS23	21.77	68.08	52.1	8	12	8	1.223	1.248	0.859	0.932	0.99	1.234	0.683	0.875	0.72	0.90	0.63
P20073	Annexin A7 OS=Homo sapiens OX=960	ANXA7	52.739	106.99	25	10	14	10	1.172	1.373	0.899	0.945	0.686	0.956	0.983	0.997	0.72	0.65	0.78
P43361	Melanoma-associated antigen 8 OS=Hon	MAGEA8	35.214	13.746	11	4	4	1	0.966	1.326	0.673	0.987	0.704	1.275	0.782	1.428	0.72	0.86	0.96
Q6NUK4	Receptor expression-enhancing protein 3	REEP3	29.264	6.4762	3.9	1	1	1	1.138	0.986	0.599	0.939	0.946	0.889	1.23	1.287	0.72	0.86	1.19
Q5QJE6	Deoxynucleotidyltransferase terminal-int	DNTTIP2	84.468	143.99	20.5	12	16	12	1.049	1.072	0.693	0.842	1.052	1.201	0.979	1.175	0.72	1.06	1.02
P46783	40S ribosomal protein S10 OS=Homo sa	RPS10	18.898	46.036	37	7	11	7	1.108	1.008	0.649	0.882	1.014	0.922	1.076	1.3	0.72	0.91	1.12
O00622	Protein CYR61 OS=Homo sapiens OX=9	CYR61	42.026	6.136	3.4	1	1	1	0.903	0.886	0.504	0.79	1.054	0.924	1.505	1.758	0.72	1.11	1.82
O43678	NADH dehydrogenase [ubiquinone] 1 alp	NDUFA2	10.921	28.594	28.3	4	5	4	1.143	1.409	0.931	0.914	0.784	0.9	1.05	0.894	0.72	0.66	0.76
Q14807	Kinesin-like protein KIF22 OS=Homo sa	KIF22	73.261	91.135	15.3	7	8	7	1.034	1.026	0.595	0.893	1.064	1.142	0.968	1.285	0.72	1.07	1.09
P50454	Serpin H1 OS=Homo sapiens OX=9606	SERPINH1	46.44	186.22	37.8	14	21	14	1.08	1.429	0.898	0.914	0.759	1.147	0.81	0.963	0.72	0.76	0.71
Q9BXS6	Nucleolar and spindle-associated protein	NUSAP1	49.451	22.129	7.5	3	3	3	1.08	1.444	0.9	0.919	0.824	1.147	0.807	0.968	0.72	0.78	0.70
P17152	Transmembrane protein 11, mitochondria	TMEM11	21.541	13.606	10.4	2	2	2	1.002	1.362	0.719	0.984	0.735	1.212	0.824	1.266	0.72	0.82	0.88
Q9Y2P8	RNA 3'-terminal phosphate cyclase-like p	RCL1	40.842	33.817	12.9	4	7	4	1.072	1.084	0.736	0.817	1.079	1.247	0.931	1.181	0.72	1.08	0.98
Q01196	Runt-related transcription factor 1 OS=H	RUNX1	48.736	6.9063	3.3	1	1	1	1.188	1.349	0.89	0.937	0.685	0.885	1.111	0.929	0.72	0.62	0.80
Q53FT3	Protein Hikeshi OS=Homo sapiens OX=9	HIKESHI	21.627	6.0199	4.6	1	2	1	1.09	1.548	0.926	0.971	0.717	1.197	0.802	0.776	0.72	0.73	0.60
P48047	ATP synthase subunit O, mitochondrial (	ATP5O	23.277	131.41	57.7	10	19	10	1.119	1.169	0.788	0.855	0.994	1.183	0.807	0.913	0.72	0.95	0.75
P16455	Methylated-DNA--protein-cysteine methy	MGMT	21.646	48.609	28.5	4	6	4	1.12	1.411	0.901	0.914	0.745	1.168	0.753	1.043	0.72	0.76	0.71
P62277	40S ribosomal protein S13 OS=Homo sa	RPS13	17.222	41.046	37.7	5	11	5	1.021	1.124	0.692	0.846	1.061	1.014	1.144	1.263	0.72	0.97	1.12
Q99880	Histone H2B type 1-L OS=Homo sapiens	HIST1H2B	13.952	6.9152	41.3	5	40	1	1.478	1.285	1.154	0.826	1.017	1.121	0.479	0.456	0.72	0.77	0.34
Q9BQ75	Protein CMSS1 OS=Homo sapiens OX=	CMSS1	31.884	20.044	11.1	3	4	3	1.109	1.127	0.749	0.853	1.008	1.088	0.997	1.171	0.72	0.94	0.97
P62851	40S ribosomal protein S25 OS=Homo sa	RPS25	13.742	28.783	28.8	4	11	4	1.143	1.081	0.704	0.889	1.009	0.945	1.165	1.204	0.72	0.88	1.07
Q96QD8	Sodium-coupled neutral amino acid trans	SLC38A2	56.025	69.58	4.2	1	1	1	1.159	1.249	0.88	0.843	0.964	1.242	0.757	0.977	0.72	0.92	0.72
P18077	60S ribosomal protein L35a OS=Homo s	RPL35A	12.538	6.8958	8.2	1	5	1	0.731	1.94	1.106	0.804	0.495	1.846	0.633	0.621	0.72	0.88	0.47
Q9P016	Thymocyte nuclear protein 1 OS=Homo s	THYN1	25.697	41.399	26.2	6	7	6	1.088	1.411	0.857	0.93	0.753	0.844	1.18	1.001	0.72	0.64	0.87
Q16651	Prostasin OS=Homo sapiens OX=9606 C	PRSS8	36.431	29.927	13.1	2	3	2	1.068	1.193	0.667	0.949	0.985	0.986	0.996	1.214	0.71	0.87	0.98
Q99643	Succinate dehydrogenase cytochrome b56	SDHC	18.61	19.243	12.4	2	2	2	1.192	1.324	0.874	0.923	0.781	1.304	0.681	0.911	0.71	0.83	0.63
Q01082	Spectrin beta chain, non-erythrocytic 1 O	SPTBN1	274.61	323.31	34	60	89	58	1.165	1.387	0.904	0.918	0.73	0.951	0.914	0.966	0.71	0.66	0.74
Q96C01	Protein FAM136A OS=Homo sapiens O:	FAM136A	15.641	45.195	44.2	6	7	6	1.204	1.155	0.894	0.79	0.994	1.166	0.886	1.066	0.71	0.92	0.83
Q96GQ7	Probable ATP-dependent RNA helicase I	DDX27	89.834	150.65	24.2	19	26	19	1.107	1.058	0.701	0.842	1.115	1.063	0.936	1.201	0.71	1.01	0.99
P98164	Low-density lipoprotein receptor-related	LRP2	521.95	225.12	6.1	22	24	22	1.153	1.283	0.801	0.932	0.828	0.952	0.948	1.045	0.71	0.73	0.82
P62753	40S ribosomal protein S6 OS=Homo sap	RPS6	28.68	74.716	27.3	6	18	6	1.13	1.114	0.746	0.848	1.004	0.99	1.013	1.207	0.71	0.89	0.99
Q13257	Mitotic spindle assembly checkpoint prot	MAD2L1	23.51	35.808	24.4	5	5	5	1.168	1.46	0.943	0.921	0.754	0.994	0.886	0.88	0.71	0.67	0.67
Q13813	Spectrin alpha chain, non-erythrocytic 1 (	SPTAN1	284.54	323.31	39.8	87	121	85	1.171	1.422	0.915	0.924	0.705	0.917	0.971	0.978	0.71	0.63	0.75
Q9NX02	NACHT, LRR and PYD domains-contair	NLRP2	120.51	311.29	32.4	35	71	28	1.213	1.248	0.876	0.868	0.872	0.873	0.995	1.018	0.71	0.71	0.82
P62847	40S ribosomal protein S24 OS=Homo sa	RPS24	15.423	26.089	19.5	2	11	2	1.118	1.059	0.656	0.886	1.022	0.937	1.011	1.3	0.71	0.90	1.06
O95865	N(G),N(G)-dimethylarginine dimethylami	DDAH2	29.644	43.121	22.8	5	6	5	1.052	1.326	0.76	0.924	0.794	0.755	1.211	1.28	0.71	0.65	1.05
P62750	60S ribosomal protein L23a OS=Homo s	RPL23A	17.695	55.477	32.7	6	12	6	1.164	1.202	0.814	0.861	0.97	1.188	0.795	1.095	0.71	0.91	0.80
P32242	Homeobox protein OTX1 OS=Homo sap	OTX1	37.327	6.0418	3.4	1	1	1	1.132	1.173	0.649	0.979	1.183	0.39	0.925	1.569	0.71	0.68	1.08
Q99996	A-kinase anchor protein 9 OS=Homo sap	AKAP9	452.98	41.654	1.7	7	7	7	1.214	1.192	0.789	0.91	0.89	1.182	0.717	0.904	0.71	0.86	0.67
P09668	Pro-cathepsin H OS=Homo sapiens OX=	CTSH	37.393	22.819	10.1	3	4	3	1.226	1.491	0.998	0.918	0.727	0.954	0.847	0.8	0.71	0.62	0.61
Q6P0N0	Mis18-binding protein 1 OS=Homo sapie	MIS18BP1	129.08	11.89	1.6	2	2	2	1.061	1.001	0.55	0.904	0.984	1.079	1.049	1.486	0.71	1.00	1.23
P08473	Neprilysin OS=Homo sapiens OX=9606	MME	85.513	37.426	7.2	6	6	6	1.21	1.116	0.757	0.882	0.864	0.866	1.07	1.241	0.70	0.74	0.99
Q9NV06	DDB1- and CUL4-associated factor 13 C	DCAF13	51.402	74.165	22	7	8	7	1.018	1.16	0.74	0.794	1.069	1.27	0.744	1.087	0.70	1.07	0.84
Q9NRX1	RNA-binding protein PNO1 OS=Homo s	PNO1	27.924	29.344	21.4	4	4	4	1.131	1.08	0.724	0.833	1.006	1.194	0.981	1.337	0.70	1.00	1.05
P50897	Palmitoyl-protein thioesterase 1 OS=Hon	PPT1	34.193	36.279	19.3	5	8	5	1.16	1.377	0.865	0.919	0.807	1.139	0.757	1.018	0.70	0.77	0.70
P50238	Cysteine-rich protein 1 OS=Homo sapien	CRIP1	8.5328	12.73	22.1	2	3	2	1.258	1.26	0.903	0.863	0.9	0.982	0.904	0.928	0.70	0.75	0.73
Q8WW33	Gametocyte-specific factor 1 OS=Homo s	GTSF1	19.266	23.941	22.2	4	3	3	1.134	1.414	0.864	0.921	0.692	1.211	0.853	0.91	0.70	0.75	0.69
Q8NG31	Kinetochore scaffold 1 OS=Homo sapien	KNL1	265.39	40.826	3	6	7	6	1.152	1.026	0.655	0.869	1.121	1.267	0.743	1.23	0.70	1.10	0.91
Q86YM7	Homer protein homolog 1 OS=Homo sap	HOMER1	40.276	62.609	23.7	7	10	7	1.104	1.163	0.712	0.872	0.936	0.964	0.96	1.223	0.70	0.84	0.96
Q9NW13	RNA-binding protein 28 OS=Homo sapie	RBM28	85.737	187.56	23.1	16	19	16	1.005	0.996	0.572	0.826	1.105	1.264	0.921	1.467	0.70	1.18	1.19
Q9ULW0	Targeting protein for Xklp2 OS=Homo sa	TPX2	85.652	117.78	27.4	16	19	16	1.059	1.05	0.659	0.814	1.103	1.079	0.999	1.236	0.70	1.03	1.06
Q6FIF0	AN1-type zinc finger protein 6 OS=Hom	ZFAND6	22.555	20.167	20.7	3	4	3	1.197	1.418	0.921	0.905	0.749	1.21	0.672	0.894	0.70	0.75	0.60
P42224	Signal transducer and activator of transcr	STAT1	87.334	201.97	35.2	21	33	21	1.216	1.456	0.93	0.935	0.683	0.944	0.894	0.888	0.70	0.61	0.67
Q9NYP3	Protein downstream neighbor of Son OS=	DONSON	62.746	18.63	4.9	3	3	3	1.072	1.166	0.684	0.878	0.941	0.941	1.123	1.314	0.70	0.84	1.09
O75915	PRA1 family protein 3 OS=Homo sapien	ARL6IP5	21.614	51.839	10.1	1	4	1	0.966	1.077	0.626	0.799	1.333	1.301	0.957	1.118	0.70	1.29	1.02
P21741	Midkine OS=Homo sapiens OX=9606 G	MDK	15.585	15.85	14	2	2	2	0.962	0.942	0.539	0.789	1.305	1.186	1.137	1.406	0.70	1.31	1.34
Q00577	Transcriptional activator protein Pur-alph	PURA	34.91	20.341	12.4	3	3	3	1.377	0.814	0.58	0.948	1.092	0.747	0.984	1.276	0.70	0.84	1.03
O15347	High mobility group protein B3 OS=Hon	HMGB3	22.98	69.347	29	7	16	7	1.183	1.434	0.908	0.917	0.681	0.902	1.003	0.964	0.70	0.60	0.75
P19525	Interferon-induced, double-stranded RNA	EIF2AK2	62.094	54.586	14.3	7	9	7	1.177	1.4	0.878	0.918	0.716	0.931	1.054	0.963	0.70	0.64	0.78
P12931	Proto-oncogene tyrosine-protein kinase S	SRC	59.834	56.039	16.6	7	8	4	1.201	1.42	0.921	0.904	0.737	1.081	0.881	0.846	0.70	0.69	0.66
Q86X29	Lipolysis-stimulated lipoprotein receptor	LSR	71.438	66.05	10.2	5	7	5	1.167	1.398	0.875	0.911	0.85	0.952	0.948	0.977	0.70	0.70	0.75
Q8NCG7	Sn1-specific diacylglycerol lipase beta O	DAGLB	73.731	25.659	6.4	4	5	4	0.972	1.264	0.584	0.972	0.889	1.235	0.647	1.591	0.70	0.95	1.00
Q9BUN8	Derlin-1 OS=Homo sapiens OX=9606 G	DERL1	28.8	6.5218	3.2	1	1	1	1.168	1.198	0.77	0.875	1.1	1.306	0.732	0.885	0.70	1.02	0.68
P07942	Laminin subunit beta-1 OS=Homo sapier	LAMB1	198.04	323.31	25.5	36	51	36	1.159	1.373	0.836	0.924	0.76	0.915	1.012	1.03	0.70	0.66	0.81
Q3KP66	Innate immunity activator protein OS=Hc	INAVA	72.913	36.239	8.6	5	6	5	1.109	1.261	0.875	0.772	1.095	1.345	0.783	0.92	0.69	1.03	0.72
Q9P219	Protein Daple OS=Homo sapiens OX=96	CCDC88C	228.23	51.828	3.9	8	8	8	1.237	1.481	0.996	0.892	0.681	0.854	1.055	0.801	0.69	0.56	0.68
Q14692	Ribosome biogenesis protein BMS1 hom	BMS1	145.81	130.73	12.8	13	15	12	1.017	1.16	0.684	0.826	1.061	1.267	0.927	1.237	0.69	1.07	0.99
P20248	Cyclin-A2 OS=Homo sapiens OX=9606	CCNA2	48.55	36.369	17.6	5	6	5	0.992	0.916	0.571	0.752	1.153	1.207	1.03	1.499	0.69	1.24	1.33
Q9Y6E2	Basic leucine zipper and W2 domain-con	BZW2	48.162	73.482	26.5	11	17	11	1.19	1.389	0.873	0.915	0.713	0.96	1.034	0.958	0.69	0.65	0.77
P62861	40S ribosomal protein S30 OS=Homo sa	FAU	6.6478	7.2786	16.9	1	4	1	1.328	1.154	1	0.718	1.047	0.866	1.195	0.699	0.69	0.77	0.76
Q9Y3C1	Nucleolar protein 16 OS=Homo sapiens (	NOP16	21.188	40.571	34.3	5	8	5	1.16	1.236	0.796	0.862	0.914	1.035	0.971	1.047	0.69	0.81	0.84
P46781	40S ribosomal protein S9 OS=Homo sap	RPS9	22.591	31.887	19.1	5	11	5	1.011	1.089	0.608	0.845	0.995	0.933	1.277	1.428	0.69	0.92	1.29
P05026	Sodium/potassium-transporting ATPase s	ATP1B1	35.061	18.708	11.2	3	3	3	1.053	1.082	0.62	0.856	1.056	1.117	1.055	1.309	0.69	1.02	1.11
Q9BRK5	45 kDa calcium-binding protein OS=Hon	SDF4	41.806	26.126	13.5	4	4	4	1.196	1.313	0.828	0.904	0.874	1.129	0.833	0.92	0.69	0.80	0.70
P62280	40S ribosomal protein S11 OS=Homo sa	RPS11	18.431	56.021	34.2	7	9	7	1.129	1.034	0.687	0.806	1.056	1.023	1.015	1.359	0.69	0.96	1.10
Q16204	Coiled-coil domain-containing protein 6	CCDC6	53.29	79.291	27.6	10	10	9	1.163	1.485	0.886	0.938	0.701	1.051	0.843	0.912	0.69	0.66	0.66
O60566	Mitotic checkpoint serine/threonine-prot	BUB1B	119.54	41.168	6.1	6	6	6	1.085	1.147	0.615	0.922	0.96	1.153	0.879	1.285	0.69	0.95	0.97
P60602	Reactive oxygen species modulator 1 OS	ROMO1	8.1828	14.262	31.6	2	2	2	1.181	1.721	0.968	1.03	0.502	1.314	0.462	0.75	0.69	0.63	0.42
Q9BRK3	Matrix remodeling-associated protein 8 (	MXRA8	49.131	25.786	10.6	4	5	4	1.204	1.309	0.878	0.851	0.957	1.146	0.748	0.937	0.69	0.84	0.67
Q14118	Dystroglycan OS=Homo sapiens OX=96	DAG1	97.44	103.31	15.6	11	12	11	1.136	1.542	0.921	0.918	0.686	0.964	0.932	0.963	0.69	0.62	0.71
O75477	Erlin-1 OS=Homo sapiens OX=9606 GN	ERLIN1	38.925	46.678	29.2	9	13	6	1.111	1.157	0.686	0.871	0.924	0.905	1.092	1.198	0.69	0.81	1.01
Q13325	Interferon-induced protein with tetratrico	IFIT5	55.846	7.5776	3.3	1	1	1	0.936	1.411	0.678	0.932	0.671	0.854	1.347	1.363	0.69	0.65	1.15
P36404	ADP-ribosylation factor-like protein 2 O:	ARL2	20.878	21.851	16.8	3	3	3	1.18	1.401	0.859	0.911	0.712	1.092	0.829	1.029	0.69	0.70	0.72
Q16625	Occludin OS=Homo sapiens OX=9606 C	OCLN	59.143	11.738	5.9	2	2	2	1.131	1.758	1.023	0.956	0.675	0.837	0.89	0.739	0.69	0.52	0.56
Q9P0V9	Septin-10 OS=Homo sapiens OX=9606 (	SEPT10	52.592	23.646	12.1	4	8	3	1.213	1.231	0.76	0.913	0.889	0.833	1.005	1.135	0.68	0.70	0.88
P42696	RNA-binding protein 34 OS=Homo sapie	RBM34	48.564	42.343	14.9	6	7	6	0.924	0.962	0.52	0.766	1.175	1.095	1.238	1.549	0.68	1.20	1.48
Q16775	Hydroxyacylglutathione hydrolase, mitoc	HAGH	33.805	27.854	15.9	4	4	4	1.192	1.358	0.872	0.863	0.846	1.062	0.89	0.95	0.68	0.75	0.72
P46779	60S ribosomal protein L28 OS=Homo sa	RPL28	15.747	50.28	29.2	6	13	6	1.019	0.986	0.579	0.785	1.168	0.925	1.147	1.448	0.68	1.04	1.29
Q92485	Acid sphingomyelinase-like phosphodies	SMPDL3B	50.813	15.621	8.1	2	2	2	1.087	1.1	0.641	0.846	1.15	1.16	0.881	1.266	0.68	1.06	0.98
O75607	Nucleoplasmin-3 OS=Homo sapiens OX=	NPM3	19.343	20.819	14.6	2	3	2	1.24	1.162	0.75	0.882	0.876	0.869	0.94	1.271	0.68	0.73	0.92
O00762	Ubiquitin-conjugating enzyme E2 C OS=	UBE2C	19.652	64.034	39.1	5	7	5	1.336	1.671	1.091	0.951	0.637	1.092	0.536	0.579	0.68	0.57	0.37
Q96EI5	Transcription elongation factor A protein	TCEAL4	24.647	52.763	48.8	8	9	8	1.191	1.429	0.844	0.935	0.753	1.02	0.881	0.94	0.68	0.68	0.70
Q9NV96	Cell cycle control protein 50A OS=Homo	TMEM30A	40.683	11.758	5	2	2	2	1.23	1.421	0.91	0.889	0.691	0.869	1.03	0.94	0.68	0.59	0.74
Q53S33	BolA-like protein 3 OS=Homo sapiens C	BOLA3	12.114	14.708	22.4	2	2	2	1.173	1.371	0.76	0.965	0.723	0.957	0.966	1.061	0.68	0.66	0.80
Q9NQ55	Suppressor of SWI4 1 homolog OS=Hon	PPAN	53.193	34.665	9.9	4	4	4	0.946	1.009	0.535	0.787	1.197	0.955	1.304	1.553	0.68	1.10	1.46
Q99523	Sortilin OS=Homo sapiens OX=9606 GN	SORT1	92.067	133.48	11.9	7	9	7	1.195	1.445	0.882	0.903	0.686	0.984	0.881	0.898	0.68	0.63	0.67
Q9NXW9	Alpha-ketoglutarate-dependent dioxygena	ALKBH4	33.837	7.8452	9.3	1	1	1	0.703	1.629	0.803	0.773		1.298	0.835	1.286	0.68	0.56	0.91
P42766	60S ribosomal protein L35 OS=Homo sa	RPL35	14.551	21.844	22.8	3	6	3	1.013	1.058	0.64	0.759	1.163	1.059	1.127	1.437	0.68	1.07	1.24
Q9Y2G4	Ankyrin repeat domain-containing protei	ANKRD6	79.97	6.32	1.5	1	1	1	1.221	1.171	0.836	0.779	1.116	0.976	0.981	0.982	0.68	0.87	0.82
O95067	G2/mitotic-specific cyclin-B2 OS=Homo	CCNB2	45.281	25.486	6.8	2	2	2	0.997	1.099	0.54	0.874	1.06	1.03	1.185	1.395	0.67	1.00	1.23
P36578	60S ribosomal protein L4 OS=Homo sap	RPL4	47.697	120.1	27.6	13	42	13	1.099	1.161	0.674	0.847	1.024	0.988	0.999	1.259	0.67	0.89	1.00
Q9H0K6	Pseudouridylate synthase 7 homolog-like	PUS7L	80.7	37.511	7.4	4	4	4	0.977	1.741	0.915	0.914	0.483	1.56	0.508	1.098	0.67	0.75	0.59
Q9NR30	Nucleolar RNA helicase 2 OS=Homo sap	DDX21	87.343	261.23	40.5	29	47	27	1.131	1.137	0.678	0.848	1.028	1.072	1.018	1.186	0.67	0.93	0.97
P20933	N(4)-(beta-N-acetylglucosaminyl)-L-aspa	AGA	37.208	33.527	14.5	5	5	5	1.323	1.151	0.799	0.864	1.051	1.102	0.711	0.922	0.67	0.87	0.66
Q96FQ6	Protein S100-A16 OS=Homo sapiens OX	S100A16	11.801	13.057	23.3	2	2	2	1.105	1.208	0.585	0.969	0.764	0.893	1.107	1.403	0.67	0.72	1.09
Q6DKI1	60S ribosomal protein L7-like 1 OS=Hor	RPL7L1	28.661	23.715	16.3	4	3	3	1.184	0.999	0.676	0.788	1.078	1.238	0.958	1.173	0.67	1.06	0.98
Q9C0K1	Zinc transporter ZIP8 OS=Homo sapiens	SLC39A8	49.63	12.587	4.8	1	3	1	1.206	1.472	0.854	0.94	0.736	0.799	1.004	0.951	0.67	0.57	0.73
Q9Y2I8	WD repeat-containing protein 37 OS=Ho	WDR37	54.665	12.515	4.3	2	2	2	1.111	1.213	0.63	0.926	1.037	1.225	0.692	1.223	0.67	0.97	0.82
P02649	Apolipoprotein E OS=Homo sapiens OX	APOE	36.154	77.509	27.4	7	7	4	1.089	1.148	0.646	0.847	1.036	1.197	0.885	1.266	0.67	1.00	0.96
Q9NP50	SIN3-HDAC complex-associated factor (	SINHCAF	24.852	13.092	7.7	2	2	2	1.33	1.393	0.89	0.924	0.678	0.835	0.974	0.85	0.67	0.56	0.67
P52758	2-iminobutanoate/2-iminopropanoate dea	RIDA	14.494	23.175	27	3	4	3	1.269	1.492	0.93	0.904	0.715	0.925	0.875	0.83	0.66	0.59	0.62
P61916	NPC intracellular cholesterol transporter	NPC2	16.57	63.81	37.7	4	13	4	1.222	1.487	0.857	0.942	0.697	1.137	0.765	0.879	0.66	0.68	0.61
Q96PE7	Methylmalonyl-CoA epimerase, mitochon	MCEE	18.749	16.227	12.5	2	2	2	1.085	1.479	0.897	0.805	0.828	1.269	0.852	0.95	0.66	0.82	0.70
P27635	60S ribosomal protein L10 OS=Homo sa	RPL10	24.604	72.067	28	5	7	5	1.143	1.304	0.713	0.911	0.702	1.057	0.89	1.085	0.66	0.72	0.81
P42771	Cyclin-dependent kinase inhibitor 2A OS	CDKN2A	16.532	30.408	34	4	6	4	1.355	1.248	0.917	0.806	0.934	1.144	0.647	0.897	0.66	0.80	0.59
Q99741	Cell division control protein 6 homolog (	CDC6	62.72	12.382	3.9	2	2	2	0.8	0.69	0.294	0.692	1.493	1.153	1.405	1.95	0.66	1.78	2.25
O43291	Kunitz-type protease inhibitor 2 OS=Hor	SPINT2	28.228	47.571	19	4	4	4	1.141	0.908	0.535	0.82	1.375	0.869	1.126	1.324	0.66	1.10	1.20
Q99963	Endophilin-A3 OS=Homo sapiens OX=9	SH3GL3	39.284	6.9181	4.6	1	1	1	1.131	1.147	0.616	0.889	0.911	0.992	1.088	1.292	0.66	0.84	1.04
P23469	Receptor-type tyrosine-protein phosphata	PTPRE	80.641	8.1854	1.7	1	2	1	1.194	1.359	0.85	0.835	0.879	1.219	0.868	0.843	0.66	0.82	0.67
Q8N8S7	Protein enabled homolog OS=Homo sapi	ENAH	66.509	72.21	16.8	8	8	8	1.245	1.596	0.955	0.917	0.615	0.897	0.893	0.85	0.66	0.53	0.61
P61254	60S ribosomal protein L26 OS=Homo sa	RPL26	17.258	38.83	30.3	6	12	2	1.095	1.082	0.616	0.818	1.002	1.016	1.107	1.413	0.66	0.93	1.16
Q9GZR7	ATP-dependent RNA helicase DDX24 O	DDX24	96.331	122.91	16.3	11	14	11	1.05	1.033	0.552	0.817	1.15	1.285	0.836	1.437	0.66	1.17	1.09
Q9ULM3	YEATS domain-containing protein 2 OS	YEATS2	150.78	18.512	2.5	3	3	3	1.036	1.086	0.619	0.774	1.155	1.415	0.883	1.257	0.66	1.21	1.01
Q9BSG0	Protease-associated domain-containing p	PRADC1	21.042	11.49	11.2	2	2	2	1.628	0.656	0.903	0.594	0.914	0.807	1.409	0.962	0.66	0.75	1.04
Q9H7B2	Ribosome production factor 2 homolog C	RPF2	35.582	25.592	14.7	4	4	4	0.932	0.898	0.383	0.816	1.246	0.936	1.341	1.834	0.66	1.19	1.73
Q96E14	RecQ-mediated genome instability protei	RMI2	15.865	7.6567	8.8	1	1	1	0.978	1.548	0.832	0.822	0.773	1.354	0.827	1.112	0.65	0.84	0.77
Q969E8	Pre-rRNA-processing protein TSR2 hom	TSR2	20.894	12.187	11	2	3	2	1.227	1.486	0.841	0.928	0.703	0.918	0.927	0.931	0.65	0.60	0.68
O43924	Retinal rod rhodopsin-sensitive cGMP 3'.	PDE6D	17.42	16.543	12.7	1	1	1	1.156	1.586	0.978	0.806	0.794	1.189	0.815	0.779	0.65	0.72	0.58
P83881	60S ribosomal protein L36a OS=Homo s	RPL36A	12.441	26.342	26.4	4	6	1	1.116	1.166	0.669	0.815	0.991	1.028	1.024	1.327	0.65	0.88	1.03
Q68D10	Protein SPT2 homolog OS=Homo sapien	SPTY2D1	75.598	24.854	6.3	4	5	4	1.038	1.191	0.668	0.78	1.01	1.386	0.79	1.37	0.65	1.07	0.97
Q9UDR5	Alpha-aminoadipic semialdehyde synthas	AASS	102.13	140.98	20.2	16	15	15	1.281	1.481	0.89	0.899	0.71	1.174	0.655	0.858	0.65	0.68	0.55
P13928	Annexin A8 OS=Homo sapiens OX=960	ANXA8	36.881	36.26	20.8	5	6	1	1.13	1.493	0.775	0.921	0.646	0.913	1.009	1.165	0.65	0.59	0.83
P43155	Carnitine O-acetyltransferase OS=Homo	CRAT	70.857	15.599	3.2	2	2	2	1.296	1.542	0.985	0.847	0.731	1.231	0.508	0.834	0.65	0.69	0.47
Q9H3G5	Probable serine carboxypeptidase CPVL	CPVL	54.163	108.68	27.3	11	22	10	1.275	1.309	0.793	0.866	0.948	1.078	0.815	0.947	0.64	0.78	0.68
Q9NUI1	Peroxisomal 2,4-dienoyl-CoA reductase (	DECR2	30.777	18.993	11.3	3	4	3	1.142	1.372	0.696	0.916	0.87	0.984	0.874	1.189	0.64	0.74	0.82
Q96A59	MARVEL domain-containing protein 3 (	MARVELI	44.91	12.665	4.7	2	2	2	1.166	0.997	0.596	0.789	0.979	1.005	1.08	1.51	0.64	0.92	1.20
Q92484	Acid sphingomyelinase-like phosphodies	SMPDL3A	51.26	15.672	5.5	2	3	2	1.248	1.55	0.83	0.957	0.635	0.882	0.899	0.925	0.64	0.54	0.65
Q96CB8	Integrator complex subunit 12 OS=Homo	INTS12	48.807	24.024	7.1	3	3	3	1.226	1.408	0.875	0.806	0.926	1.234	0.712	0.863	0.64	0.82	0.60
P62244	40S ribosomal protein S15a OS=Homo s	RPS15A	14.839	19.291	24.6	3	7	3	1.137	1.197	0.624	0.865	1.053	1.197	0.839	1.26	0.64	0.96	0.90
P16401	Histone H1.5 OS=Homo sapiens OX=96	HIST1H1B	22.58	28.778	25.2	5	8	4	0.934	1.013	0.569	0.673	1.072	0.812	1.523	2.142	0.64	0.97	1.88
Q6PK18	2-oxoglutarate and iron-dependent oxyge	OGFOD3	35.646	6.8275	2.5	1	1	1	1.301	1.223	0.761	0.848	1.036	1.301	0.638	0.844	0.64	0.93	0.59
Q9Y4C8	Probable RNA-binding protein 19 OS=H	RBM19	107.33	57.581	11	9	10	9	1.059	1.097	0.59	0.779	1.062	1.046	1.15	1.435	0.63	0.98	1.20
Q8N9N8	Probable RNA-binding protein EIF1AD (	EIF1AD	19.053	57.399	42.4	6	8	6	1.144	1.148	0.591	0.863	0.904	1.142	0.939	1.306	0.63	0.89	0.98
O76021	Ribosomal L1 domain-containing protein	RSL1D1	54.972	185.21	39.2	19	28	19	1.006	1.03	0.544	0.746	1.157	1.129	1.126	1.509	0.63	1.12	1.29
Q9NQX7	Integral membrane protein 2C OS=Homo	ITM2C	30.223	7.1983	4.1	1	2	1	0.968	1.441	0.607	0.918	0.774	1.229	0.953	1.294	0.63	0.83	0.93
Q8WVX9	Fatty acyl-CoA reductase 1 OS=Homo sa	FAR1	59.356	53.473	8.3	4	6	4	1.211	1.597	0.936	0.841	0.754	1.001	0.852	0.851	0.63	0.63	0.61
P40429	60S ribosomal protein L13a OS=Homo s	RPL13A	23.577	50.429	33	7	23	7	1.087	1.257	0.654	0.827	1.008	1.21	0.903	1.238	0.63	0.95	0.91
Q9NZV1	Cysteine-rich motor neuron 1 protein OS	CRIM1	113.74	11.459	2.6	2	2	2	1.265	1.568	0.889	0.9	0.696	1.151	0.64	0.848	0.63	0.65	0.53
P48426	Phosphatidylinositol 5-phosphate 4-kina	PIP4K2A	46.224	40.568	12.1	4	6	3	1.125	1.391	0.685	0.903	0.866	0.746	1.152	1.19	0.63	0.64	0.93
Q96BR5	Cytochrome c oxidase assembly factor 7 (	COA7	25.709	12.179	10.4	2	2	2	1.21	1.427	0.799	0.863	0.78	1.332	0.729	0.882	0.63	0.80	0.61
Q08380	Galectin-3-binding protein OS=Homo sa	LGALS3BI	65.33	58.441	11.5	4	7	4	1.194	1.312	0.736	0.843	0.865	0.887	1.147	1.135	0.63	0.70	0.91

P50995	Annexin A11 OS=Homo sapiens OX=96	ANXA11	54.389	55.826	18.4	8	10	8	1.129	1.4	0.71	0.883	0.724	1.013	0.993	1.153	0.63	0.69	0.85
P05161	Ubiquitin-like protein ISG15 OS=Homo	ISG15	17.887	28.551	24.8	4	7	4	1.384	1.406	0.911	0.838	0.666	0.765	0.983	0.826	0.63	0.51	0.65
Q96GA3	Protein LTV1 homolog OS=Homo sapier	LTV1	54.854	27.155	9.3	4	5	4	1.301	1.498	0.956	0.797	0.699	1.182	0.759	0.809	0.63	0.67	0.56
Q9GZR2	RNA exonuclease 4 OS=Homo sapiens C	REXO4	46.671	26.954	9.2	4	5	4	1.137	1.15	0.631	0.8	1.118	1.224	0.892	1.174	0.63	1.02	0.90
P46013	Proliferation marker protein Ki-67 OS=H	MKI67	358.69	323.31	36.7	85	106	84	1.135	1.131	0.613	0.799	1.055	1.099	0.959	1.275	0.62	0.95	0.99
Q92784	Zinc finger protein DPF3 OS=Homo sapi	DPF3	43.084	31.926	10.1	3	4	3	1.174	1.726	1.004	0.799	0.678	0.867	1.056	0.847	0.62	0.53	0.66
P26373	60S ribosomal protein L13 OS=Homo sa	RPL13	24.261	61.112	34.1	7	13	7	1.115	1.108	0.62	0.761	1.093	1.001	1.083	1.373	0.62	0.94	1.10
Q9BV23	Monoacylglycerol lipase ABHD6 OS=Hc	ABHD6	38.33	42.118	22	6	6	6	1.252	1.683	0.92	0.9	0.587	0.999	0.816	0.919	0.62	0.54	0.59
Q8WX94	NACHT, LRR and PYD domains-contair	NLRP7	111.81	87.871	19.1	17	22	10	1.05	1.112	0.524	0.813	0.829	0.786	1.438	1.594	0.62	0.75	1.40
P14543	Nidogen-1 OS=Homo sapiens OX=9606	NID1	136.38	88.287	8.7	8	10	8	1.199	1.511	0.842	0.825	0.79	1.131	0.833	0.942	0.62	0.71	0.65
Q5T1C6	Acyl-coenzyme A thioesterase THEM4 O	THEM4	27.129	14.608	8.3	2	2	2	1.232	1.559	0.893	0.821	0.85	1.066	0.779	0.833	0.61	0.69	0.58
O75113	NEDD4-binding protein 1 OS=Homo sap	N4BP1	100.38	21.329	3.7	3	3	3	1.213	1.806	0.983	0.871	0.644	1.049	0.652	0.804	0.61	0.56	0.48
Q9NYH9	U3 small nucleolar RNA-associated prote	UTP6	70.193	53.036	14.4	8	7	7	1.125	1.332	0.615	0.889	0.882	1.052	0.953	1.226	0.61	0.79	0.89
P02654	Apolipoprotein C-I OS=Homo sapiens O	APOC1	9.3318	7.9913	10.8	1	1	1	1.181	1.519	0.792	0.86	0.765	1.31	0.682	0.947	0.61	0.77	0.60
Q9UI08	Ena/VASP-like protein OS=Homo sapier	EVL	44.619	14.167	7.5	2	2	2	1.251	1.534	0.898	0.802	0.63	1.094	0.888	0.947	0.61	0.62	0.66
P84098	60S ribosomal protein L19 OS=Homo sa	RPL19	23.466	65.804	21.9	5	15	5	1.06	1.265	0.618	0.8	0.935	1.108	0.996	1.311	0.61	0.88	0.99
Q9GZQ8	Microtubule-associated proteins 1A/1B l	MAP1LC3l	14.688	6.5539	11.2	1	1	1	1.211	1.169	0.619	0.831	1.027	1.358	0.66	1.178	0.61	1.00	0.77
Q9UJW3	DNA (cytosine-5)-methyltransferase 3-lik	DNMT3L	43.582	65.542	21.5	6	11	6	1.312	1.747	0.948	0.904	0.603	1.073	0.731	0.751	0.61	0.55	0.48
P50914	60S ribosomal protein L14 OS=Homo sa	RPL14	23.432	30.405	19.1	4	7	4	0.959	0.873	0.444	0.665	0.963	1.028	1.31	1.806	0.61	1.09	1.70
Q96AB3	Isochorismatase domain-containing prote	ISOC2	22.337	18.893	17.6	2	2	2	1.212	1.656	0.85	0.886	0.692	1.388	0.559	0.768	0.61	0.73	0.46
Q9P031	Thyroid transcription factor 1-associated	CCDC59	28.669	12.695	8.3	2	2	2	1.1	1.142	0.647	0.71	1.056	1.31	0.888	1.383	0.61	1.06	1.01
Q8N567	Zinc finger CCHC domain-containing pr	ZCCHC9	30.477	6.7922	4.4	1	2	1	1.06	1.068	0.533	0.747	1.186	1.367	0.84	1.435	0.60	1.20	1.07
Q96EU6	Ribosomal RNA processing protein 36 h	RRP36	29.823	12.447	5.8	1	2	1	1.163	1.018	0.553	0.752	1.28	1.225	0.857	1.294	0.60	1.15	0.99
P49641	Alpha-mannosidase 2x OS=Homo sapien	MAN2A2	130.54	6.1669	1	1	1	1	0.956	1.541	0.67	0.823	0.858	0.983	1.138	1.293	0.60	0.74	0.97
Q9BX67	Junctional adhesion molecule C OS=Hor	JAM3	35.02	46.204	14.2	3	4	3	1.317	1.375	0.733	0.867	0.833	0.98	0.886	1.129	0.59	0.67	0.75
Q01995	Transgelin OS=Homo sapiens OX=9606	TAGLN	22.611	45.319	36.8	7	8	6	1.037	1.279	0.59	0.786	0.676	0.831	1.425	1.596	0.59	0.65	1.30
P25391	Laminin subunit alpha-1 OS=Homo sapie	LAMA1	337.08	323.31	15.6	37	52	36	1.15	1.326	0.615	0.852	0.869	0.946	1.079	1.302	0.59	0.73	0.96
Q14690	Protein RRP5 homolog OS=Homo sapier	PDCD11	208.7	274.23	17.5	29	36	28	1.141	1.153	0.566	0.788	1.018	1.027	1.004	1.301	0.59	0.89	1.00
Q9BVJ6	U3 small nucleolar RNA-associated prote	UTP14A	87.977	82.524	20.8	12	12	12	1.181	1.164	0.575	0.808	1.02	1.22	0.772	1.261	0.59	0.96	0.87
Q9Y324	rRNA-processing protein FCF1 homolog	FCF1	23.369	20.927	10.1	2	2	2	1.144	1.123	0.541	0.793	1.084	1.193	0.92	1.335	0.59	1.00	0.99
Q9BRX8	Redox-regulatory protein FAM213A OS=	FAM213A	25.764	13.924	11.4	2	2	2	1.187	1.464	0.842	0.716	0.669	0.957	1.183	1.14	0.59	0.61	0.88
Q14050	Collagen alpha-3(IX) chain OS=Homo sa	COL9A3	63.615	61.095	5.4	2	3	2	1.306	1.404	0.713	0.876	0.796	1.207	0.572	1.076	0.59	0.74	0.61
Q8N257	Histone H2B type 3-B OS=Homo sapiens	HIST3H2B	13.908	38.946	41.3	5	39	1	1.352	1.989	1.414	0.531	0.456	1.098	0.12	0.235	0.58	0.47	0.11
P54296	Myomesin-2 OS=Homo sapiens OX=960	MYOM2	164.89	9.2056	1.2	2	1	1	1.207	1.529	0.788	0.802	0.794	1.259	0.783	0.913	0.58	0.75	0.62
Q9GZV5	WW domain-containing transcription reg	WWTR1	44.101	12.4	4.2	2	3	2	1.279	1.474	0.803	0.793	0.828	0.98	0.916	0.95	0.58	0.66	0.68
Q9UM01	Y+L amino acid transporter 1 OS=Homo	SLC7A7	55.99	7.561	6.1	1	2	1	1.378	1.587	0.901	0.815	0.623	0.796	0.974	0.853	0.58	0.48	0.62
Q6IA17	Single Ig IL-1-related receptor OS=Homo	SIGIRR	45.679	14.363	4.6	2	2	2	1.322	1.602	0.823	0.864	0.665	1.192	0.59	0.887	0.58	0.64	0.51
Q7L0Q8	Rho-related GTP-binding protein RhoU (	RHOU	28.218	14.531	8.9	2	2	2	1.17	1.411	0.624	0.865	0.697	1.185	0.878	1.239	0.58	0.73	0.82
O60493	Sorting nexin-3 OS=Homo sapiens OX=!	SNX3	18.762	24.122	34.6	5	8	4	1.247	1.593	0.856	0.782	0.713	1.216	0.794	0.812	0.58	0.68	0.57
P49006	MARCKS-related protein OS=Homo sap	MARCKSl	19.529	33.866	14.4	2	4	2	1.245	1.615	0.825	0.82	0.643	0.798	1.052	0.951	0.58	0.50	0.70
P62424	60S ribosomal protein L7a OS=Homo sa	RPL7A	29.995	200.08	45.1	17	31	17	0.974	0.917	0.374	0.712	1.139	0.952	1.293	1.789	0.57	1.11	1.63
Q9HBJ0	Placenta-specific protein 1 OS=Homo sa	PLAC1	23.616	8.113	5.7	1	1	1	1.24	1.614	0.787	0.85	0.729	1.135	0.663	1.005	0.57	0.65	0.58
Q13895	Bystin OS=Homo sapiens OX=9606 GN	BYSL	49.601	50.829	17.4	7	10	7	1.193	1.286	0.569	0.852	0.916	1.077	0.906	1.228	0.57	0.80	0.86
Q9UFN0	Protein NipSnap homolog 3A OS=Homo	NIPSNAP3	28.466	32.426	21.5	5	6	5	0.902	2.253	0.968	0.84	0.572	1.3	0.691	0.749	0.57	0.59	0.46
P83731	60S ribosomal protein L24 OS=Homo sa	RPL24	17.779	55.748	35.7	7	14	7	1.055	1.067	0.509	0.707	1.121	0.959	1.2	1.612	0.57	0.98	1.33
P11047	Laminin subunit gamma-1 OS=Homo sap	LAMC1	177.6	323.31	28.7	35	46	34	1.196	1.456	0.645	0.87	0.698	0.895	1.063	1.187	0.57	0.60	0.85
Q9NZM5	Ribosome biogenesis protein NOP53 OS	NOP53	54.389	46.067	16.9	7	9	7	1.279	1.522	0.785	0.814	0.83	1.254	0.738	1.039	0.57	0.74	0.63
P0DI82	Trafficking protein particle complex subu	TRAPPC2I	16.445	6.712	5.7	1	1	1	1.388	1.291	0.818	0.711	1.098	0.824	0.994	0.859	0.57	0.72	0.69
Q5T9L3	Protein wntless homolog OS=Homo sapi	WLS	62.253	26.14	5	4	5	4	1.392	1.569	0.946	0.743	0.805	1.099	0.606	0.864	0.57	0.64	0.50
P60520	Gamma-aminobutyric acid receptor-assoc	GABARAP	13.667	22.725	29.1	3	3	3	1.31	1.503	0.782	0.816	0.818	1.026	0.795	0.933	0.57	0.66	0.61
Q14651	Plastin-1 OS=Homo sapiens OX=9606 C	PLS1	70.253	67.323	17.6	10	13	7	1.288	1.699	0.867	0.819	0.543	0.842	0.969	0.903	0.56	0.46	0.63
Q15397	Pumilio homolog 3 OS=Homo sapiens O	PUM3	73.584	83.561	19.9	12	16	12	1.185	1.254	0.574	0.797	1.019	1.245	0.902	1.184	0.56	0.93	0.86
Q9BZA8	Protocadherin-11 Y-linked OS=Homo sa	PCDH11Y	146.77	11.086	1.3	2	2	2	1.192	1.481	0.622	0.878	0.7	0.911	1.002	1.255	0.56	0.60	0.84
P40189	Interleukin-6 receptor subunit beta OS=F	IL6ST	103.54	24.976	6	2	2	2	0.994	1.321	0.522	0.777	0.937	0.915	1.218	1.589	0.56	0.80	1.21
P17174	Aspartate aminotransferase, cytoplasmic (	GOT1	46.247	109.44	31.7	11	17	11	1.283	1.576	0.794	0.806	0.71	1.026	0.824	0.981	0.56	0.61	0.63
P14635	G2/mitotic-specific cyclin-B1 OS=Homo	CCNB1	48.337	58.755	18.5	7	8	6	1.144	1.075	0.528	0.712	1.13	1.224	0.944	1.33	0.56	1.06	1.02
Q9NY93	Probable ATP-dependent RNA helicase I	DDX56	61.589	65.04	17.9	9	12	9	1.138	1.15	0.509	0.75	1.073	1.346	0.836	1.267	0.55	1.06	0.92
P78325	Disintegrin and metalloproteinase domair	ADAM8	88.77	20.234	4.5	2	2	2	1.167	1.672	0.695	0.866	0.7	1.23	0.706	1.034	0.55	0.68	0.61
Q9Y580	RNA-binding protein 7 OS=Homo sapier	RBM7	30.503	20.353	12.8	3	4	3	1.331	1.616	0.886	0.733	0.78	1.23	0.712	0.823	0.55	0.68	0.52
P04114	Apolipoprotein B-100 OS=Homo sapiens	APOB	515.6	6.7904	0.5	2	1	1	1.222	1.258	0.564	0.797	1.055	1.398	0.648	1.129	0.55	0.99	0.72
Q9H267	Vacuolar protein sorting-associated prote	VPS33B	70.584	44.201	12.2	7	7	7	1.301	1.394	0.594	0.881	0.665	1.164	0.688	1.276	0.55	0.68	0.73
P46782	40S ribosomal protein S5 OS=Homo sap	RPS5	22.876	26.935	22.1	4	9	3	1.136	1.418	0.593	0.801	0.766	1.158	0.917	1.09	0.55	0.75	0.79
Q92994	Transcription factor IIIB 90 kDa subunit	BRF1	73.839	6.4674	1.2	1	2	1	1.232	1.263	0.524	0.83	1.056	1.165	0.715	1.256	0.54	0.89	0.79
P30405	Peptidyl-prolyl cis-trans isomerase F, mit	PPIF	22.04	22.604	22.7	4	3	3	1.187	1.529	0.603	0.853	0.694	0.911	1.035	1.252	0.54	0.59	0.84
P49207	60S ribosomal protein L34 OS=Homo sa	RPL34	13.293	20.562	22.2	3	8	3	1.062	1.076	0.494	0.647	1.09	0.961	1.244	1.577	0.53	0.96	1.32
Q6NS38	DNA oxidative demethylase ALKBH2 O!	ALKBH2	29.322	11.811	11.9	2	3	2	1.32	1.451	0.699	0.775	0.859	1.056	0.872	0.975	0.53	0.69	0.67
Q9Y6Y0	Influenza virus NS1A-binding protein OS	IVNS1ABF	71.729	37.202	9.5	5	6	5	1.285	1.38	0.596	0.803	0.686	0.852	1.115	1.309	0.52	0.58	0.91
P13796	Plastin-2 OS=Homo sapiens OX=9606 C	LCP1	70.288	323.31	55	28	69	22	1.247	1.398	0.592	0.796	0.812	1.013	0.934	1.205	0.52	0.69	0.81
Q9BVS4	Serine/threonine-protein kinase RIO2 OS	RIOK2	63.282	25.482	6.2	2	3	2	1.307	1.646	0.768	0.773	0.741	1.188	0.673	0.933	0.52	0.65	0.54
P09758	Tumor-associated calcium signal transdu	TACSTD2	35.709	38.294	22.6	5	6	5	1.25	1.524	0.628	0.807	0.709	0.879	1.06	1.22	0.52	0.57	0.82
O75817	Ribonuclease P protein subunit p20 OS=	POP7	15.651	37.249	17.9	2	2	2	1.038	1.73	0.409	0.982	0.427	1.641	0.355	1.543	0.50	0.75	0.69
O95864	Fatty acid desaturase 2 OS=Homo sapien	FADS2	52.259	52.355	9	3	5	3	1.293	1.531	0.633	0.775	0.737	1.185	0.862	1.059	0.50	0.68	0.68
Q14CB8	Rho GTPase-activating protein 19 OS=H	ARHGAP1	55.756	7.4863	2.8	1	1	1	1.168	1.586	0.517	0.853	0.647	1.348	0.717	1.257	0.50	0.72	0.72
P17693	HLA class I histocompatibility antigen, al	HLA-G	38.224	103.42	33.1	9	17	9	1.469	1.685	0.743	0.805	0.606	0.773	0.881	0.93	0.49	0.44	0.57
P07305	Histone H1.0 OS=Homo sapiens OX=96(	H1F0	20.863	21.954	14.9	3	4	3	1.012	1.081	0.345	0.68	0.998	0.868	1.322	2.093	0.49	0.89	1.63
Q9NUK0	Muscleblind-like protein 3 OS=Homo sa	MBNL3	38.531	7.6946	5.4	2	3	1	1.158	2.002	0.73	0.81	0.683	1.602	0.408	0.731	0.49	0.72	0.36
Q4KWH8	1-phosphatidylinositol 4,5-bisphosphate	PLCH1	189.22	6.8495	0.5	1	4	1	1.413	1.705	0.757	0.739	0.729	1.168	0.639	0.887	0.48	0.61	0.49
Q8NEY4	V-type proton ATPase subunit C 2 OS=F	ATP6V1C2	48.758	26.533	10.3	4	4	4	1.261	1.363	0.544	0.709	1.005	1.249	0.84	1.137	0.48	0.86	0.75
P18124	60S ribosomal protein L7 OS=Homo sap	RPL7	29.225	147.05	48.8	13	27	12	0.945	1.336	0.334	0.73	0.941	0.94	1.265	1.828	0.47	0.82	1.36
P38571	Lysosomal acid lipase/cholesteryl ester h	LIPA	45.418	8.7319	4	1	2	1	1.303	1.612	0.53	0.792	0.668	1.261	0.692	1.171	0.45	0.66	0.64
P0DPB3	Schwannomin-interacting protein 1 OS=I	SCHIP1	53.479	6.9406	1.6	1	1	1	1.325	1.396	0.512	0.719	1.138	0.933	0.899	1.124	0.45	0.76	0.74
Q96P44	Collagen alpha-1(XXI) chain OS=Homo	COL21A1	99.367	30.799	4.7	4	4	4	1.357	1.62	0.523	0.822	0.631	1.162	0.681	1.144	0.45	0.60	0.61
Q99715	Collagen alpha-1(XII) chain OS=Homo s	COL12A1	333.14	110.08	7.1	15	16	15	1.345	1.657	0.601	0.746	0.704	0.917	0.924	1.045	0.45	0.54	0.66
Q9Y3A2	Probable U3 small nucleolar RNA-associ	UTP11	30.446	30.401	20.9	5	5	5	1.115	1.128	0.4	0.6	1.304	1.068	1.06	1.42	0.45	1.06	1.11
P98155	Very low-density lipoprotein receptor OS	VLDLR	96.098	61.873	11.7	9	10	9	1.343	1.657	0.54	0.771	0.644	1.101	0.808	0.874	0.44	0.58	0.56
O95478	Ribosome biogenesis protein NSA2 hom	NSA2	30.065	44.967	20.8	5	5	5	1.241	1.597	0.509	0.704	0.789	1.368	0.749	1.187	0.43	0.76	0.68
Q96L50	Leucine-rich repeat protein 1 OS=Homo	LRR1	46.722	21.428	7.7	3	3	3	1.332	1.278	0.462	0.594	1.203	1.307	0.806	1.158	0.40	0.96	0.75
Q02487	Desmocollin-2 OS=Homo sapiens OX=9	DSC2	99.961	6.5566	1.1	1	1	1	1.672	2.14	0.79	0.729	0.462	0.876	0.476	0.642	0.40	0.35	0.29
O60911	Cathepsin L2 OS=Homo sapiens OX=96	CTSV	37.329	182.27	38.9	10	19	9	1.632	2.372	0.555	0.613	0.573	0.944	0.57	0.726	0.29	0.38	0.32
Q9Y625	Glypican-6 OS=Homo sapiens OX=9606	GPC6	62.735	14.451	4.5	2	2	2	1.6	2.249	0.368	0.655	0.511	0.64	0.873	1.022	0.27	0.30	0.49
P33121	Long-chain-fatty-acid--CoA ligase 1 OS=	ACSL1	77.942	46.028	9.9	6	6	6	1.706	2.205	0.434	0.571	0.551	0.992	0.602	0.834	0.26	0.39	0.37

Table S2

up-regulated in JEG3/MTX	down-regulated in JEG3/MTX	up-regulated in JEG3/5-Fu	down-regulated in JEG3/5-Fu	up-regulated in JEG3/VP16	down-regulated in JEG3/VP16	shared (up)	shared (down)
SLAMF1	ADD3	CENPC	JAM3	SLAMF1	CCNB2	SLAMF1	SINHCAF
TTN	SORT1	TTN	ANXA6	TTN	RPL4	TTN	EVL
GRIA2	AKAP9	SLAMF1	LTV1	UBIAD1	PUS7L	GRIA2	DPF3
SOX8	MAD2L1	UBIAD1	MYH14	SOX8	DDX21	SOX8	COL12A1
AKAP6	KRT1	GRIA2	SAMHD1	GRIA2	AGA	AKAP6	SMPDL3A
EYS	PTBP1	C10orf67	MAD2L1	AKAP6	S100A16	EYS	ISG15
DHFR	MXRA8	PARL	NOCT	CENPE	RPL7L1	UBIAD1	LIPA
UBIAD1	PTPRE	SOX8	LIPA	HERPUD1	SLC39A8	PARL	GOT1
PARL	SINHCAF	CES1	CCDC6	C10orf67	WDR37	C10orf67	PLS1
C10orf67	PTCD1	EYS	LAMB1	PARL	APOE	SCN4A	RIDA
SCN4A	EIF1AY	CENPE	SEMA4C	EYS	SINHCAF	SERPINA10	SLC7A7
SERPINA10	STAT1	CXCR2	BOLA3	SERPINA10	RIDA	MVP	GABARAPL2
MVP	ALKBH2	AKAP6	AAMDC	MAP7D3	NPC2	METTL7A	ENAH
METTL7A	KIF21B	ZFYVE26	NDUFA2	SCN4A	MCEE	MAP7D3	COL21A1
MAP7D3	COCH	SERPINA10	SPTBN1	KIF26B	RPL10	ARHGDIB	FAR1
ARHGDIB	PDSS2	TFCP2L1	WWTR1	MTMR14	CDKN2A	CENPE	ABHD6
KIF26B	TEAD1	DPP4	GABARAPL2	CENPC	CDC6	FHL1	PLAC1
CENPE	PLK1	FHL1	RIOK2	METTL7A	SPINT2	DPP4	HLA-G
FHL1	CCDC6	METTL7A	PLAC1	ABCG2	SH3GL3	GPX3	VLDLR
DPP4	RPIA	MAP7D3	CRIM1	MAGEA4	PTPRE	CES1	LTV1
GPX3	COX17	ARHGDIB	LRRFIP1	CES1	ENAH	CXCR2	RIOK2
RRM2	AGA	RCCD1	DDAH2	ARHGDIB	RPL26	HMOX1	CRIM1
TYMS	SGPL1	MVP	TAGLN	DPP4	DDX24	AKR1C1	SIGIRR
CES1	SRC	COCH	MFSD10	MVP	YEATS2	C1QTNF6	WLS
CXCR2	EVL	SCN4A	IFIT5	MAGEB2	PRADC1	HCLS1	GPC6
HMOX1	GUSB	HERPUD1	DGKZ	PEG3	RPF2	INPP4A	PLCH1
AKR1C1	DPF3	AKR1C1	BZW2	CDKN1A	RMI2	HIST1H1D	DNMT3L
RPL18	COL12A1	CCDC120	TENM3	INPP4A	TSR2	GSN	N4BP1
C1QTNF6	MRPS2	MAGEB2	MYO18A	AKR1C1	PDE6D	PFN2	NIPSNAP3A
HCLS1	NID1	B4GALT7	EPCAM	CXCR2	RPL36A	GPX1	ACSL1
INPP4A	LGMN	MTMR14	ANXA7	GPX3	SPTY2D1	B4GALT7	CTSV
HIST1H1D	DLX3	ASF1B	WLS	TP53	AASS	ARPIN	DSC2
ASF1B	NFRKB	ABCG2	PALLD	GPX1	ANXA8	ANXA3	HIST3H2BB
CDC6	SMPDL3A	RRM2	PIP4K2A	B4GALT7	CRAT	NFIL3	
GSN	PTP4A2	DNAJB4	EIF2AK2	PAGE4	CPVL	PEG3	
ABCC1	CDCA2	GPX3	THYN1	ANXA3	DECR2	MAGEB2	
PFN2	FAM83D	FADD	EHBP1	FN1	MARVELD3	GRHL3	
GPX1	ISG15	PEG3	CAMKMT	LGALS1	SMPDL3A	TTC5	
B4GALT7	SAMHD1	TYMS	AKAP12	PTGR2	INTS12	S100P	
ARPIN	RAB10	INPP4A	PDLIM5	ARPIN	RPS15A	SRXN1	
SFN	COX7A2	MAGEA4	SIGIRR	C1QTNF6	HIST1H1B	ABCG2	
ANXA3	LIPA	GRHL3	SGPL1	COCH	OGFOD3	E2F6	
NFIL3	PSMF1	GPX1	TANC2	ZFYVE26	RBM19	PTGR2	
RGN	MXRA7	SYNE3	SORT1	FHL1	EIF1AD	STK38	
PEG3	SLC6A19	CRIP2	COL4A1	FADD	RSL1D1	MAPK1	
MAGEB2	NOP53	L1RE1	TAGLN2	HCLS1	ITM2C	TP53	
GRHL3	TMEM120A	ANXA3	MAPKAPK2	DNAJB4	FAR1	ASS1	
TTC5	SDHC	SFN	BCAT1	ASS1	RPL13A	SETX	
DTL	ERAL1	PCLAF	ROMO1	GSN	CRIM1	SELENOF	
LGALS1	GID8	SRXN1	SPTAN1	ATR	PIP4K2A	L1RE1	
S100P	GOT1	E2F6	FAR1	DSC3	COA7	HEBP1	
SRXN1	MRPS23	NFIL3	F13A1	AFG1L	LGALS3BP	DNAJB4	
ABCG2	PLS1	ZNF385A	LIN28A	S100P	ANXA11		
HIST1H1B	LIN28A	MGST1	EVL	TTC5	ISG15		
E2F6	KIF1A	CDCA5	RUNX1	PFN2	LTV1		
PTGR2	CDC20	CDC6	CTSH	HMOX1	REXO4		
CDCA5	MYOM2	PALM3	KIF1A	HIST1H1D	MKI67		
SFXN4	RIDA	STK38	DAG1	HEBP1	DPF3		
ATP5IF1	SURF2	CEP78	LPCAT1	PPP1R12B	RPL13		
ISM2	SLC7A7	ASS1	FAM213A	L1RE1	ABHD6		
ROR2	CELSR1	FBXO30	USP11	NFIL3	NLRP7		
CYR61	GABARAPL2	BIN3	STAT1	RCCD1	NID1		
PAGE4	BRD2	HCLS1	PLCH1	E2F6	THEM4		
GSTM2	ENAH	SH3GLB1	GOT1	GRHL3	N4BP1		
HMGN4	COL21A1	S100P	SERPINB9	DCP2	UTP6		
STK38	ADAM8	C1QTNF6	HMGB3	CHD6	APOC1		
MAPK1	H1FX	TP53	H1FX	STK38	EVL		
HIST1H1E	COA7	ANLN	PCDH11Y	SOD1	RPL19		
TK1	ARL6IP1	PHLDA3	COL21A1	FMO1	MAP1LC3B		
BIN3	COL9A3	CHAF1B	LAMC1	RPRD1A	DNMT3L		
TP53	FURIN	ARPIN	TSR2	SRXN1	RPL14		
RPF2	NPC2	POMK	HMGN3	TRPV2	ISOC2		
C19orf53	FAR1	EPS8L1	OLFML1	SETX	CCDC59		
LRRC14	CTSH	HMOX1	ANXA8	COTL1	ZCCHC9		
GRAMD2B	APOC1	HIST1H1D	RIDA	CCDC120	RRP36		
ASS1	ZFAND6	DSC3	NIPSNAP3A	GBE1	MAN2A2		
RPL14	HIKESHI	ZCCHC7	PPIF	SMIM1	JAM3		
DCP2	INTS12	TP53I3	TMEM30A	SELENOF	TAGLN		
SETX	TMEM143	C16orf58	VLDLR	ABCC1	LAMA1		
GRK6	TPT1	CENPS	ANXA8L1	DR1	PDCD11		
EPS8L1	CDKN2A	SSFA2	FSCN1	ATP5IF1	UTP14A		
BLM	MTCH2	SELENOF	IVNS1ABP	GNL3L	FCF1		
RASGRP2	ABHD6	HEXIM1	UBE2C	PHLDA3	FAM213A		
SPAG7	PUS7L	CXXC1	SLC39A8	C16orf58	COL9A3		
CENPQ	OGFOD3	PSMB10	TACSTD2	RABL3	HIST3H2BB		
GALK1	PLAC1	SFXN4	KIF20A	TP53I3	MYOM2		
PLIN2	PDE6D	TTC5	CCDC88C	SMAD9	WWTR1		
KCTD9	MPG	SDCBP	PPP3CA	EPS8L2	SLC7A7		
MED15	THEM4	PFN2	N4BP1	ZNF385A	SIGIRR		
AFG1L	HLA-G	TRPV2	ALKBH4	MAPK1	RHOU		
COTL1	NME2	TMEM263	SINHCAF	TMEM263	SNX3		
H1F0	SNX3	RARS2	SKAP2	PNPLA2	MARCKSL1		
ATR	OCLN	HERC2	DNMT3L	PLIN2	RPL7A		
RPL7A	VLDLR	MAPK1	SMPDL3A	TUBGCP4	PLAC1		
SOD1	LTV1	GDPD3	ABHD6	KRT1	BYSL		
PPP1R12B	AASS	PAK6	COL12A1	ABCD1	NIPSNAP3A		
TGM1	WDR12	SETX	PPME1	GNG10	RPL24		
CYSTM1	RIOK2	GSN	IFRD1	HMGN2	LAMC1		
SELENOF	AURKA	STOM	DPF3	PSMB10	NOP53		
SMAD9	CNPY2	PPM1D	ENAH	HSPA4L	TRAPPC2B		
L1RE1	CRIM1	PTGR2	OCLN	GALK1	WLS		
IGF2	SMYD5	GBE1	ISG15	TFCP2L1	GABARAPL2		
APOBEC2	RBM7	HEBP1	MARCKSL1	PPP6R1	PLS1		
ANKRD24	B2M	ERI1	SLC7A7	MGST1	PUM3		
HEBP1	MRFAP1	ISM2	HIST3H2BB	CASTOR2	PCDH11Y		
ANLN	SIGIRR	MAZ	PLS1		IL6ST		
FN1	WLS	FAM98C	HLA-G		GOT1		
PPM1D	GPC6	STON2	ACSL1		CCNB1		
TPBG	PLCH1	IGF2	CTSV		DDX56		
CHD6	MGST3		STK11IP		ADAM8		
DNAJB4	DNMT3L		SLC4A7		RBM7		
CCDC117	N4BP1		DSC2		APOB		
FHL2	CRAT		HIST2H3A		VPS33B		
EGFR	RPL35A		GPC6		RPS5		
CENPO	ISOC2				BRF1		
SMIM1	MP68				PPIF		

LENG8	NIPSNAP3A	RPL34
PALM3	KIF20A	ALKBH2
H2AFX	ROMO1	IVNS1ABP
SQSTM1	GNG5	LCP1
DCPS	C1orf174	RIOK2
GAB2	UBE2C	TACSTD2
RAB27A	CCNL2	POP7
SGO1	ACSL1	FADS2
CCK	MBNL3	ARHGAP19
HP1BP3	TUBB8	HLA-G
	HIST1H2BL	H1F0
	CTSV	MBNL3
	DSC2	PLCH1
	HIST2H3A	ATP6V1C2
	HIST3H2BB	RPL7
		LIPA
		SCHIP1
		COL21A1
		COL12A1
		UTP11
		VLDLR
		NSA2
		LRR1
		DSC2
		CTSV
		GPC6
		ACSL1
